# Supplementary material for: Reporting inconsistency between published conference abstracts and article abstracts of randomised controlled trials in prosthodontics presented at IADR general sessions
Source: PeerJ. 2023 May 5;11:e15303. doi: 10.7717/peerj.15303 (PMC10166077; doi:10.7717/peerj.15303)
Supplement: Supplemental Information 1 [file peerj-11-15303-s001.pdf]

2002

Comparing The Time to Fabricate Complete Dentures Using Two Different Methods

Comparing The Time to Fabricate Complete Dentures Using Two Different Methods

Objectives: The traditional method for denture fabrication (traditional-T) taught to dental students is not routinely employed by dentists after graduation (Murphy et al., 1971), perhaps because it is time-consuming and costly. Some authors assume that this lowers the quality of care, but this has never been proven. Therefore, we decided to compare the T method of fabrication with a simpler method (modified-M), that appears to be standard practice for generalists in both North America and the UK (Gauthier G et al., 1992; Hyde and McCord, 1999). **Methods:** A randomized controlled clinical trial was carried out with 53 edentulous patients (21 male and 32 female; mean age=61yrs.). The subjects were stratified by oral condition, then randomly allocated into two groups (traditional and modified) and treated by one experienced prosthodontist. Twenty-seven were treated with the traditional method and 26 were treated with the modified method. Patient satisfaction with their dentures was measured with questionnaires and external examiners blind to treatment also assessed the quality of dentures. In this presentation, we compare chair time for each procedure measured with a stopwatch. **Results:** There were no significant differences between the groups for age, gender and oral condition. A Mann-Whitney test was used to compare between-group differences for median time (min.) of final impressions (T 57, M 18; P=0.001), inter-occlusal records (T 34, M 30; P=0.04), try-in (T 27, M 22; P=0.62), delivery (T 24, M 31; P=0.01) and total time of fabrication (T 203, M 112; P=0.001). **Conclusions:** The results reveal that significantly less chair time is needed when a modified method is used to fabricate conventional dentures.
ykawai@med.mcgill.ca

Division: IADR/AADR/CADR General Session
Meeting: 2002 IADR/AADR/CADR General Session (San Diego, California)
Location: San Diego, California
Year: 2002
Final Presentation ID: 266

Authors

- Kawai Yasuhiko (Nihon University School of Dentistry at Matsudo, Matsudo, N/A, Japan)
- Penrod John R (McGill University, Montreal, QC, Canada)
- Murakami H (Nihon University School of Dentistry at Matsudo, Matsudo, N/A, Japan)
- Takanashi Y (McGill University, Montreal, QC, Canada)
- Lund James Percy (McGill University, Montreal, QC, Canada)
- Feine Jocelyne S (McGill University, Montreal, QC, Canada)

SESSION INFORMATION
Oral Session
Implant Prostheses and Complete Dentures: Clinical Evaluations
03/07/2002

Kawai Yasuhiko (Nihon University School of Dentistry at Matsudo, Matsudo, N/A, Japan)
Penrod John R (McGill University, Montreal, QC, Canada)
Murakami H (Nihon University School of Dentistry at Matsudo, Matsudo, N/A, Japan)
Takanashi Y (McGill University, Montreal, QC, Canada)
Lund James Percy (McGill University, Montreal, QC, Canada)
Feine Jocelyne S (McGill University, Montreal, QC, Canada)

Comparing The Time to Fabricate Complete Dentures Using Two Different Methods

Do traditional techniques produce better conventional complete dentures than simplified techniques?

Kawai Y[1], Murakami H, Shariati B, Klemetti E, Blomfield JV, Billette L, Lund JP, Feine JS.

Author information

1 Department of Gnatho-Oral Prosthetic Rehabilitation, Nihon University School of Dentistry at Matsudo, Chiba, Japan. ykawai@mascat.nihon-u.ac.jp

Abstract

OBJECTIVES: To compare the quality of conventional complete dentures fabricated with two different techniques. A randomized controlled clinical trial was conducted to compare traditional (T) and simplified (S) methods of making complete conventional dentures on patients' ratings of satisfaction, comfort and function at 3 and 6 months following delivery. The quality of the prostheses was rated by prosthodontists at 6 months.

MATERIALS AND METHODS: One hundred twenty-two male and female edentulous individuals, aged 45-75 years, were randomly allocated into groups that received dentures made with either T or S methods. Following delivery, patients' ratings of several denture-related factors were measured using 100mm visual analogue scales, and denture quality was assessed by blinded prosthodontists using ratings on a validated quantitative scale.

RESULTS: There were no significant differences between the two groups in patient ratings for overall satisfaction (3 months: mean T = 83 mm, mean S = 83 mm, P = 0.97; 6 months: mean T = 79 mm, mean S = 79 mm, P = 0.96) or in prosthodontists' ratings of denture quality (T = 66, S = 63; P = 0.38).

CONCLUSION: These results show that the quality of complete dentures does not suffer when manufacturing techniques are simplified to save time and materials. Dental educators should consider these findings when re-designing prosthodontic training programs.

Comment in

Simple complete denture techniques can provide patient satisfaction. [Evid Based Dent. 2006]

PMID: 16139697 DOI: 10.1016/j.jdent.2005.01.005

Does It Take More Time to Provide Mandibular 2-implant Overdentures Than Conventional Dentures?

Does It Take More Time to Provide Mandibular 2-implant Overdentures Than Conventional Dentures?

Objectives: In order to provide low cost implant treatment to the large number of edentulous individuals who are poor, clinicians must know what costs are involved and how they are distributed. The aim of this study is to compare the time and number of visits needed by an oral surgeon and a prosthodontist to provide and maintain mandibular 2-implant overdentures and conventional dentures. **Methods:** Sixty edentulous patients (aged 65 to 75 years) completed a randomized clinical trial. All received new maxillary conventional dentures and either a mandibular conventional denture (CD, n=30) or a 2-implant overdenture on ball attachments (ID, n=30). The time and number of visits - both scheduled and unscheduled visits - were recorded for each patient from the preliminary examination to 6 months after delivery. Data from the two groups were compared using Mann-Whitney U tests. **Results:** The mean number of visits for ID and CD were 16.3 and 10.8, respectively. The oral surgeon spent a mean time of 112 min for ID and examined one CD patient (total 6 min). The prosthodontist also relined the dentures of the ID patients during the surgical phase (mean time - 29 min). Large and significant differences between the two groups were found both for total mean clinician's time (Δ=171 min, P<0.05) and number of appointments (Δ=5.5 visits, P<0.05). However, the differences in mean time and in number of visits with the prosthodontist for the two types of prostheses from preliminary impressions to 6 months recall were very similar (ID - 296 min; 10.1 visits, CD - 282 min; 10.8 visits) and not significant (P>0.05). **Conclusions:** Although mandibular 2-implant overdentures require more time to provide than conventional dentures, this is accounted for in the surgical phase; no additional time is needed during the prosthodontic phase.

Division: IADR/AADR/CADR General Session
Meeting: 2002 IADR/AADR/CADR General Session (San Diego, California)
Location: San Diego, California
Year: 2002
Final Presentation ID: 340

Authors

- Takanashi Yoshiaki (McGill University, Montreal, QC, Canada)
- Penrod John R (McGill University, Montreal, QC, Canada)
- Chehade Antoine (McGill University, Montreal, QC, Canada)
- Klemetti Esa (McGill University, Montreal, QC, Canada)
- Savard Andre (McGill University, Montreal, QC, Canada)
- Lund James P. (McGill University, Montreal, QC, Canada)
- Feine Jocelyne S. (McGill University, Montreal, QC, Canada)

SESSION INFORMATION
Poster Session
Removable Prostheses: Acceptance, Outcomes, Clinical Evaluations
03/07/2002

Takanashi Yoshiaki (McGill University, Montreal, QC, Canada)
Penrod John R (McGill University, Montreal, QC, Canada)
Chehade Antoine (McGill University, Montreal, QC, Canada)
Klemetti Esa (McGill University, Montreal, QC, Canada)
Savard Andre (McGill University, Montreal, QC, Canada)
Lund James P. (McGill University, Montreal, QC, Canada)
Feine Jocelyne S. (McGill University, Montreal, QC, Canada)

Does It Take More Time to Provide Mandibular 2-implant Overdentures Than Conventional Dentures?

Does a prosthodontist spend more time providing mandibular two-implant overdentures than conventional dentures?
(PMID:12170856)

Abstract | Citations | Related Articles | Data | BioEntities | External Links

Takanashi Y, Penrod JR, Chehade A, Klemetti E, Savard A, Lund JP, Feine JS

The International Journal of Prosthodontics [01 Jul 2002, 15(4):397-403]

Type: Comparative Study, Clinical Trial, Research Support, Non-U.S. Gov't, Randomized Controlled Trial, Journal Article

Abstract

PURPOSE: In this article, the time taken by a prosthodontist to fabricate and maintain mandibular overdentures retained by two implants and conventional dentures is compared. MATERIALS AND METHODS: Sixty edentulous patients between the ages of 65 and 75 completed a randomized clinical trial. All received new maxillary conventional dentures and either a mandibular conventional denture (n = 30) or a two-implant overdenture on ball attachments (n = 30). The time spent by the prosthodontist and the number of visits required for treatment, including both scheduled and unscheduled visits, were recorded for each patient from preliminary impressions to 6 months following delivery. Data from the two groups were compared using Mann-Whitney U tests. RESULTS: The prosthodontist spent a mean total time of 296 minutes in treating an implant overdenture patient and 282 minutes on a conventional denture patient during the period from preliminary impressions to the 6-month follow-up. The mean numbers of appointments were 10.1 (implant group) and 10.8 (conventional group). These differences were not significant. CONCLUSION: Although additional knowledge is required to treat patients with implant prostheses, the time required by the prosthodontist to provide two-implant mandibular overdentures with ball attachments was not significantly different than the time needed for conventional denture treatment.

Effect of Occlusal Appliance Therapy on TMD Treatment Need in a Non-Patient Sample

Effect of Occlusal Appliance Therapy on TMD Treatment Need in a Non-Patient Sample

Objectives: Occlusal splints may be beneficial in the treatment of TMD, but the evidence based on randomized, controlled trials (RCTs) is scarce (Forssell et al.1999). As part of a study concerning secondary otalgia and TMD we wanted to test the effect of a stabilization splint in subjects with TMD treatment need in a double-blind study set-up. **Methods:** Altogether 36 subjects diagnosed as having active treatment need for TMD (Kuttila 1998) including muscular, arthrogenous or combined signs and symptoms, gave their consent to participate in the study. None of the subjects had earlier received treatment for TMD. The subjects were randomly assigned into a treatment group (stabilization splint) or a control group (non-occluding palatal splint, Ekberg et al.1998). Two subjects interrupted the trial. Of the remaining 34 subjects, 18 subjects with a stabilization splint (5 men, 13 women, mean age 45 years) and 16 subjects with a control splint (10 men and 6 women, mean age 48 years) continued throughout the 10 weeks´ test period with the splint in night-time use. At the end of the treatment period the subjects were re-examined blindly and TMD treatment need assessed. **Results:** In the stabilization splint group 13 out of 18 were no longer in need of treatment for TMD, while the corresponding figures for the control splint group were 6 out of 16. The difference between the groups was statistically significant (p=0.045, Fisher´s exact test). The mean number of muscles tender to palpation as well as the number of subjects with four or more tender muscles decreased statistically significantly in the stabilization splint group but not in the control group. **Conclusion:** In a short-term perspective, the use of a stabilization splint reduces signs and symptoms of TMD more effectively than a palatal, non-occluding control splint.

Division: IADR/AADR/CADR General Session
Meeting: 2002 IADR/AADR/CADR General Session (San Diego, California)
Location: San Diego, California
Year: 2002
Final Presentation ID: 2337

Authors

- Le Bell Yrsa (University of Turku, Turku, N/A, Finland)
- Kuttila Marjaana (Otonhammas Oy, Jyväskylä, N/A, Finland)
- Savolainen-niemi Elina (Otonhammas Oy, Jyväskylä, N/A, Finland)
- Kuttila Seppo (Otonhammas Oy, Jyväskylä, N/A, Finland)
- Alanen Pentti (University of Turku, Turku, N/A, Finland)

Le Bell Yrsa (University of Turku, Turku, N/A, Finland)
Kuttila Marjaana (Otonhammas Oy, Jyväskylä, N/A, Finland)
Savolainen-niemi Elina (Otonhammas Oy, Jyväskylä, N/A, Finland)
Kuttila Seppo (Otonhammas Oy, Jyväskylä, N/A, Finland)
Alanen Pentti (University of Turku, Turku, N/A, Finland)

SESSION INFORMATION
Poster Session
TMD - Treatment and Pathophysiology
03/08/2002

Effect of Occlusal Appliance Therapy on TMD Treatment Need in a Non-Patient Sample

Acta Odontol Scand. 2002 Aug;60(4):248-54.

Efficiency of occlusal appliance therapy in secondary otalgia and temporomandibular disorders.

Kuttila M[1], Le Bell Y, Savolainen-Niemi E, Kuttila S, Alanen P.

Author information

1 marjaana.kuttila@otonhammmas.fi

Abstract

In clinical practice, it is commonly assumed that occlusal splints have therapeutic value in the treatment of temporomandibular disorders CTMD), but the evidence based on randomized controlled trials is scarce. This study evaluated the short-term (10-week) efficacy of a stabilization splint in subjects with recurrent secondary otalgia and active TMD treatment need using a randomized, controlled, double-blind design. Thirty-six subjects were randomly allocated to the two treatment groups: the stabilization splint and the control splint group. After 10 weeks' treatment, the intensity of secondary otalgia, measured on a VAS scale (from 0 to 100 mm), decreased statistically significantly in the stabilization splint group (t 2.12; P 0.006), but not in the control group. Improvement in active TMD treatment need in subjects showing moderate or severe signs and symptoms of TMD was reported significantly more often in the stabilization splint group than in the control splint group (chi2 5.71; P.017). A statistically significant decrease in the Helkimo clinical dysfunction index was seen in the subjects with stabilization splint (Z-2.63; P.009), but not in the subjects with control splint. The results indicate that the use of a stabilization splint is beneficial with regard to secondary otalgia and active TMD treatment need.

PMID: 12222651

[Indexed for MEDLINE]

Effectiveness of Habit Awareness Training for Temporomandibular Disorder Pain

Effectiveness of Habit Awareness Training for Temporomandibular Disorder Pain

Objectives: This study tested the hypothesis that a behaviorally-oriented habit awareness program emphasizing awareness and reduction of parafunctional activities would be as effective in the treatment of temporomandibular disorder pain as a standard dental intervention. **Methods:** A randomized trial design was employed. Eight TMD patients diagnosed with myofascial pain and/or arthralgia (according to the Research Diagnostic Criteria) were assigned to either an interocclusal appliance therapy group or to a behavioral intervention group. Patients assigned to the interocclusal appliance therapy group received a flat plane splint, adjusted for comfort. Patients were initially instructed to wear the splint during the day and at night. They were further instructed to taper their use of the splint during the day as treatment progressed. Patients assigned to the behavioral intervention group were instructed on the impact that parafunctional clenching has on pain. They were also given a demonstration of the impact of tooth contact or "jaw setting" on the activity of the masticatory muscles. They were then given a pager and instructed to check tooth position and masticatory muscle tension when they were paged. If they detected contact or tension at this or any other time, they were instructed to relax the masticatory muscles. The pagers contacted these patients approximately every two hours during the day, but not at night. The initial phase of treatment lasted one month. Follow-up evaluations were performed by a trained, blinded examiner. **Results:** Self-report measures of pain decreased significantly for both groups, $p < .05$, but pain levels did not differ between groups. **Conclusions:** <u>Behavioral treatment techniques emphasizing awareness and control of parafunctional activity and masticatory muscle tension may be effective treatments for TMD-related pain</u>.

Division: IADR/AADR/CADR General Session
Meeting: 2002 IADR/AADR/CADR General Session (San Diego, California)
Location: San Diego, California
Year: 2002
Final Presentation ID: 3739

Authors

- Kim Nellie (University of Missouri-Kansas City, Kansas City, MO, USA)
- Glaros Alan (University of Missouri-Kansas City, Kansas City, MO, USA)
- Lausten Leonard (University of Missouri - Kansas City, Kansas City, MO, USA)

SESSION INFORMATION
Poster Session
TMD - Epidemiology, Pathophysiology and Diagnosis
03/09/2002

Kim Nellie (University of Missouri-Kansas City, Kansas City, MO, USA)
Glaros Alan (University of Missouri-Kansas City, Kansas City, MO, USA)
Lausten Leonard (University of Missouri - Kansas City, Kansas City, MO, USA)

Effectiveness of Habit Awareness Training for Temporomandibular Disorder Pain

Appl Psychophysiol Biofeedback. 2007 Dec;32(3-4):149-54. Epub 2007 Jun 15.

Comparison of habit reversal and a behaviorally-modified dental treatment for temporomandibular disorders: a pilot investigation.

Glaros AG[1], Kim-Weroha N, Lausten L, Franklin KL.

Author information

1 Kansas City University of Medicine and Biosciences, 1750 Independence Ave, Kansas City, MO 64106, USA. aglaros@kcumb.edu

Abstract

This study tested the hypothesis that a habit reversal program emphasizing awareness and reduction of masticatory muscle activity would significantly reduce pain in patients diagnosed with chronic temporomandibular disorder (TMD) and would be a competitive alternative to a behaviorally-modified dental intervention. Eight individuals diagnosed with TMD were randomly assigned to a splint therapy or habit reversal group. Patients in the splint group received an interocclusal appliance (splint) fabricated from acrylic and were instructed to wear the splint day and night up to a maximum of 20 h per day. Patients in the habit reversal group were given a pager and instructed to check tooth position and masticatory muscle tension when paged. Paging occurred approximately once every 2 h during the day, but not at night. Both groups were instructed to avoid tooth contact and relax the masticatory muscles during the 4 weeks of active treatment. Outcome data were collected at 1 month and 1 year post-treatment intervals. Pain decreased significantly for both groups and did not differ between groups. Habit reversal may be as effective as a behaviorally-modified splint therapy for TMD-related pain.

PMID: 17570054 DOI: 10.1007/s10484-007-9039-5

[Indexed for MEDLINE]

Giomer Composite and Microfilled Composite in Clinical Double Blind Study

Giomer Composite and Microfilled Composite in Clinical Double Blind Study

A new restorative that the manufacturers call a giomer composite has been introduced. **Objective:** The objective of this study is to determine retention (RT), anatomical form (F), caries (CR), restoration stained (RS), marginal discoloration (MD), marginal adaptation (MA), surface roughness (SR) and sensitivity (ST) of giomer compared to microfilled composites. **Methods:** All patients received a dental prophylaxis at least two weeks before placement of restorations. Forty sets of restorations were placed randomly in cuspids and bicuspids in vivo using Beautifil, a giomer composite, with Shofu Fluorobond adhesive and Silux Plus, a microfilled composite, with Scotchbond Multi-Purpose Plus adhesive in erosion/abrasion/abfraction Class 5 lesions that have not been altered with a rotary instruments and according to manufacturer's recommendations. Restorations were evaluated using modified USPH criteria at baseline, eighteen months and three years by two calibrated examiners, independently. If differences existed forced consensus was required. **Results:** There were no differences at baseline, which were evaluated two weeks after placement. At three years the Beautifil and Silux Plus treatments did not have significantly different retention ($p=0.56$), anatomical form ($p=0.65$), caries ($p=0.37$), restoration stained ($p=1.0$), marginal discoloration ($p=0.26$), marginal adaptation ($p=0.06$), surface roughness ($p=0.37$) or sensitivity ($p=0.17$). **Conclusions:** Both products meet clinical portion of the Acceptance Program Guidelines for Dentin and Enamel Adhesives materials the ADA requires. Beautifil ranked higher in five areas, Silux Plus ranked higher in two areas and they were equal in one area.

Division: IADR/AADR/CADR General Session
Meeting: 2002 IADR/AADR/CADR General Session (San Diego, California)
Location: San Diego, California
Year: 2002
Final Presentation ID: 437

Authors

- Matis, Bruce A. (Indiana University School of Dentistry, Indianapolis, IN, USA)
- Cochran, Michael A. (Indiana University School of Dentistry, Indianapolis, IN, USA)
- Carlson, Timothy J. (Indiana University School of Dentistry, Indianapolis, IN, USA)
- Eckert, George J. (Indiana University School of Dentistry, Indianapolis, IN, USA)
- Kulapongs, Kultip Jane (Indiana University School of Dentistry, Indianapolis, IN, USA)

SESSION INFORMATION
Poster Session
Clinical Evaluations of Adhesives, Composites, Ormocers, Giomers
03/07/2002

Matis, Bruce A. (Indiana University School of Dentistry, Indianapolis, IN, USA)
Cochran, Michael A. (Indiana University School of Dentistry, Indianapolis, IN, USA)
Carlson, Timothy J. (Indiana University School of Dentistry, Indianapolis, IN, USA)
Eckert, George J. (Indiana University School of Dentistry, Indianapolis, IN, USA)
Kulapongs, Kultip Jane (Indiana University School of Dentistry, Indianapolis, IN, USA)

Giomer Composite and Microfilled Composite in Clinical Double Blind Study

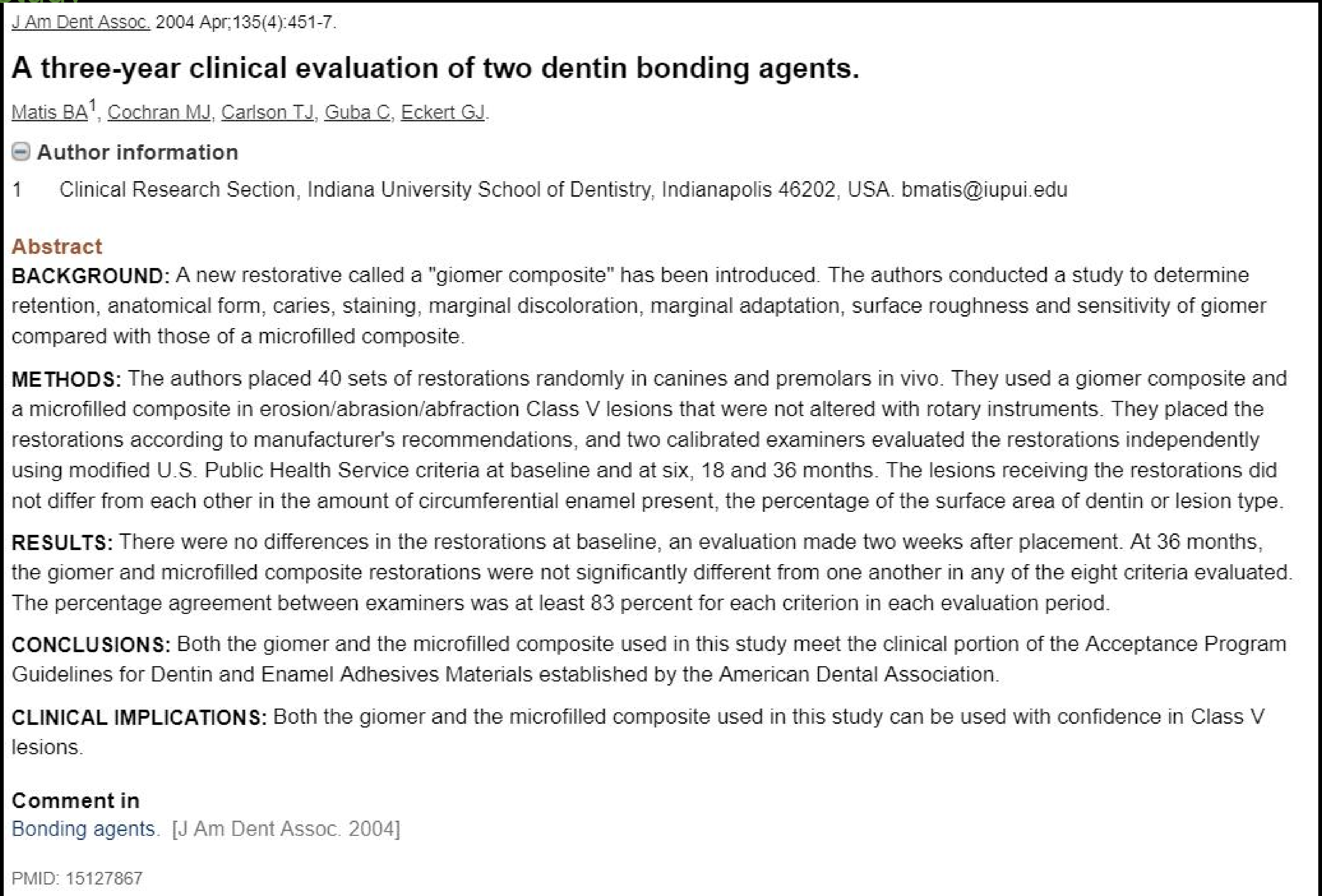

J Am Dent Assoc. 2004 Apr;135(4):451-7.

A three-year clinical evaluation of two dentin bonding agents.

Matis BA[1], Cochran MJ, Carlson TJ, Guba C, Eckert GJ.

Author information

1 Clinical Research Section, Indiana University School of Dentistry, Indianapolis 46202, USA. bmatis@iupui.edu

Abstract

BACKGROUND: A new restorative called a "giomer composite" has been introduced. The authors conducted a study to determine retention, anatomical form, caries, staining, marginal discoloration, marginal adaptation, surface roughness and sensitivity of giomer compared with those of a microfilled composite.

METHODS: The authors placed 40 sets of restorations randomly in canines and premolars in vivo. They used a giomer composite and a microfilled composite in erosion/abrasion/abfraction Class V lesions that were not altered with rotary instruments. They placed the restorations according to manufacturer's recommendations, and two calibrated examiners evaluated the restorations independently using modified U.S. Public Health Service criteria at baseline and at six, 18 and 36 months. The lesions receiving the restorations did not differ from each other in the amount of circumferential enamel present, the percentage of the surface area of dentin or lesion type.

RESULTS: There were no differences in the restorations at baseline, an evaluation made two weeks after placement. At 36 months, the giomer and microfilled composite restorations were not significantly different from one another in any of the eight criteria evaluated. The percentage agreement between examiners was at least 83 percent for each criterion in each evaluation period.

CONCLUSIONS: Both the giomer and the microfilled composite used in this study meet the clinical portion of the Acceptance Program Guidelines for Dentin and Enamel Adhesives Materials established by the American Dental Association.

CLINICAL IMPLICATIONS: Both the giomer and the microfilled composite used in this study can be used with confidence in Class V lesions.

Comment in

Bonding agents. [J Am Dent Assoc. 2004]

PMID: 15127867

Mandibular Ridge Height and Perceptions of Conventional and Overdentures

Mandibular Ridge Height and Perceptions of Conventional and Overdentures

Objectives: Previously (JDR:80:752:2001) we reported that treatment with mandibular implant-supported overdentures (IOD) improves masticatory performance only in persons with resorbed mandibular ridges. This study compares the effects of mandibular conventional dentures (CD) and implant-retained overdentures (IOD) on patient perception in persons with low, moderate and high mandibular ridge heights.
Methods: A total of 63 participants with a mandibular symphyseal height of 21 mm or less (low group), between 21mm and 28mm (moderate group), or 28mm or greater (high group) were selected from participants who had received new study dentures with either a mandibular conventional denture (CD) or an implant-retained overdenture (IOD) in a previous randomized clinical trial. This provided groups of low (n=6), moderate (n=8) and high (n=11) ridge height in the CD group and low (n=11) moderate (n=14) and high (n=13) in the IOD group. A 13 item questionnaire evaluating patient perceptions of the chewing function, speaking ability, social life, denture hygiene, self-confidence, and overall satisfaction was given to assess original dentures at entry and study dentures at 6-months after treatment.

Results: Significant differences 6-months after study denture treatment between CD and IOD occurred only for chewing comfort and speech in the moderate bone height group (Mann-Whitney's U test, P<0.02). The only significant difference seen between denture treatments in change scores (6-month minus entry), was for food choices in the moderate ridge height group (Mann-Whitney's U test, P<0.04). No significant differences were found for change scores in the low and high ridge height groups.

Conclusions: The results indicate that patients have improvements in perceptions of dentures following treatment with either a mandibular CD or IOD and these perceptions are not dependent on the bone height of the mandibular ridge. (Supported by NIDCR Grant DE-09085 and DVA Medical Research Service)

Division: IADR/AADR/CADR General Session
Meeting: 2002 IADR/AADR/CADR General Session (San Diego, California)
Location: San Diego, California
Year: 2002
Final Presentation ID: 337

Authors

- Kimoto K (UCLA School of Dentistry, Weintraub Center, Los Angeles, CA, USA)
- Garrett Nr (UCLA School of Dentistry, Weintraub Center, VA GLAHS, , N/A, USA)
- Roumanas Eleni (UCLA School of Dentistry, Weintraub Center, Los Angeles, CA, USA)
- Hamada Michael (UCLA School of Dentistry, Los Angeles, CA, USA)
- Kapur Krishan (UCLA School of Dentistry, Weintraub Center, Los Angeles, CA, USA)

Kimoto K (UCLA School of Dentistry, Weintraub Center, Los Angeles, CA, USA)
Garrett Nr (UCLA School of Dentistry, Weintraub Center, VA GLAHS, , N/A, USA)
Roumanas Eleni (UCLA School of Dentistry, Weintraub Center, Los Angeles, CA, USA)
Hamada Michael (UCLA School of Dentistry, Los Angeles, CA, USA)
Kapur Krishan (UCLA School of Dentistry, Weintraub Center, Los Angeles, CA, USA)

SESSION INFORMATION
Poster Session
Removable Prostheses: Acceptance, Outcomes, Clinical Evaluations
03/07/2002

Mandibular Ridge Height and Perceptions of Conventional and Overdentures

Effect of mandibular ridge height on patients' perceptions with mandibular conventional and implant-assisted overdentures

Article *in* The International journal of oral & maxillofacial implants 20(5):762-8 · September 2005 *with* 18 Reads

Source: PubMed

Cite this publication

Katsuhiko Kimoto

Neal R Garrett

Abstract

This study assessed the impact of mandibular ridge height on patients' perceptions of dentures following treatment with a mandibular conventional denture (CD) or an implant-assisted overdenture (IOD). Evaluation of patient satisfaction in 63 participants was made with original complete dentures and 6 months after treatment completion with new dentures. Twenty-five patients received a new mandibular CD and 38 received a new mandibular IOD. The subjects were divided into 3 subgroups according to ridge height (low, moderate, or high). Two questionnaires with categorical responses were administered. Questionnaire 1 had 13 questions to determine patients' assessment of their original dentures at entry and of their study dentures at 6 months after treatment completion. Questionnaire 2, which was given at 6 months after treatment completion, had 11 questions assessing the change perceived by patients with new dentures compared to their original dentures. No significant differences between the 2 groups were found for most of the variables in Questionnaire 1 at either time point or in regard to the difference between time points. The retrospective questionnaire 2 showed the IOD group to have significantly better perceptions than the CD group for improvement in chewing comfort, ability to eat hard foods, eating enjoyment, and denture security. The only effect of ridge height was an interaction with denture treatment for eating enjoyment, where mean improvement with the study denture was significantly less for the moderate ridge height group with the CD. The results indicate that patients in all ridge height groups had similar improvement in perceptions of dentures following treatment with either a mandibular CD or IOD and that these perceptions were not dependent on the bone height of the mandibular ridge.

Photocolorimetric Measurements for Clinical Shade Matching of Porcelain Crowns

Photocolorimetric Measurements for Clinical Shade Matching of Porcelain Crowns

The ability to select an acceptable shade match is a great concern in esthetic restorative dentistry. **Objectives:** The purpose of this study was to compare the accuracy of clinical shade selection and a photocolorimetric technique. **Methods:** Patients (n=40) treatment planned to receive porcelain fused to metal (PFM) or all-porcelain crowns were used in this study. These patients were randomly divided into 2 groups for shade selection: conventional visual assessment and photocolorimetric analysis. At the preparation appointment, a photograph was taken of the target tooth along with four shade guide tabs selected by the 2 visual observers. The crown was fabricated by the either visual selection or by the lowest ΔE values determined from the photographs and a spectrophotometer. The same dental laboratory fabricated all forty restorations. At the cementation appointment, clinical criteria were used to evaluate anatomy/contour, surface texture, and the amount of glaze as it relates to color perception before the restoration was cemented. **Results:** Analysis of data showed that the observers and colorimetric technique were perfect (ΔE=0) 41% of the time and they varied (ΔE=0.1 or higher) 59% of the time. Data collected further showed no significant difference between shade selection methods and clinical criteria. **Conclusion:** These results provide evidence that there is no significant difference in shade selection using the conventional visual assessment or the the photocolorimetric technique.

Division: IADR/AADR/CADR General Session
Meeting: 2002 IADR/AADR/CADR General Session (San Diego, California)
Location: San Diego, California
Year: 2002
Final Presentation ID: 2543

Authors

- Dancy Wm. Kevin (University of Michigan, Ann Arbor, MI, USA)
- Yaman P. (University of Michigan, Ann Arbor, MI, USA)
- Dennison J.b. (University of Michigan, Ann Arbor, MI, USA)
- O'brien W.j. (University of Michigan, Ann Arbor, MI, USA)
- Razzoog M.e. (University of Michigan, Ann Arbor, MI, USA)
- O'kray H. (University of Michigan, Ann Arbor, MI, USA)

Molin Margareta K (Umeå University, Umea, N/A, Sweden)
Karlsson Stig L (Goteborg University, Goteborg, N/A, Sweden)

SESSION INFORMATION
Poster Discussion Session
Computerized Color Analysis and Fabrication of Ceramic Prostheses
03/08/2002

Photocolorimetric Measurements for Clinical Shade Matching of Porcelain Crowns

J Esthet Restor Dent. 2003;15(2):114-21; discussion 122.

Color measurements as quality criteria for clinical shade matching of porcelain crowns.

Dancy WK[1], Yaman P, Dennison JB, O'Brien WJ, Razzoog ME.

Author information

Abstract

STATEMENT OF PROBLEM: The ability of a dentist to select and communicate an acceptable shade match to a dental laboratory may be the most important factor in esthetic restorative dentistry.

PURPOSE: The purpose of this study was to evaluate the use of instrumental color measurement in clinical shade matching of porcelain-fused-to-metal (PFM) and all-porcelain crowns. The relative effects of clinical and laboratory factors related to shade matching for PFM and all-porcelain crowns were also evaluated.

MATERIALS AND METHOD: Forty patients treatment planned to receive PFM or all-porcelain crowns made up the study population. The patients were randomly divided into two groups for shade selection: conventional visual assessment and photocolorimetric analysis. At the preparation appointment, a photograph was taken of the target tooth along with four shade guide tabs selected by the two visual observers. The crown was fabricated by either visual selection or by the lowest E* values determined from the photographs and a spectrophotometer. The same dental laboratory fabricated all 40 restorations. At the cementation appointment, clinical criteria were used to evaluate anatomy/contour, surface texture, and the amount of glaze as it relates to color perception before the restoration was cemented.

RESULTS: The mean E* between the reference tooth before preparation and the crown before cementation in the visual assessment group was 10.49 (+/- 14.6), whereas the mean E* in the photocolorimetric group was 8.99 (+/- 5.7). Analysis of data showed that the observers and the colorimetric technique were perfect (E* = 0) 41% of the time and varied (E* = 0.1 or higher) 59% of the time. Data collected further showed no significant difference or correlation between shade selection methods and the evaluated clinical criteria.

CONCLUSIONS: These results provide evidence that there is no significant difference in shade selection using the conventional visual assessment by two experienced clinicians or the photocolorimetric technique.

CLINICAL SIGNIFICANCE: The use of photocolorimetric analysis in shade selection can serve as a reliable alternative to conventional visual shade selection. This method is useful for clinicians who have difficulty with shade selection.

PMID: 12762475

Pulsed Radio Frequency Energy in Treatment of Temporomandibular Joint Pain

Pulsed Radio Frequency Energy in Treatment of Temporomandibular Joint Pain

This randomized controlled double-blind study was designed to evaluate the effectiveness of Pulsed Radio Frequency Energy Therapy (PRFE) in patients with temporomandibular joint arthralgia. Forty subjects, 9 males and 31 females, were enrolled in the study (age range 22 to 55 y) with the diagnosis of temporomandibular joint arthralgia according to Research Diagnostic Criteria for Temporomandibular Joint Disorders. Subjects were randomly assigned into two equal groups: (1) experimental group received PRFE using the Energex unit (Orthosonix, Inc. Tappan NJ) and (2) control group received PRFE placebo treatment using a sham device. During each treatment visit both groups received six applications (15 seconds each) applied to TMJ area and this procedure was repeated 6-times over a period of 2 weeks. Joint pain was measured by a 10-point Numerical Rating Scale (NRS). Mandibular range of motion was measured in mm. Both were recorded before and after each application. ANOVA for repeated measured analyzed four time periods pre-experimentally, visit number 4, first and second week follow-up. ANOVA for NRS scores showed that there was a main effect for pain over time ($F_{df=3}$ =8.8, $P < 0.0001$). The experimental group decreased significantly (mean pain=6.1 to 3.0) while there was no significant effect for time in control group $F_{df=3}$ =1.9. $P > 0.05$ (mean pain=5.3 to 4.2). The experimental group showed a significant increase in mouth opening over time $F_{df=3}$ =4.7, P=0.0005) mean=35.3 mm to 41.7 mm. There was no significant change in the control group for mouth opening, ($F_{df=3}$ =0.3, $P > 0.05$) mean=38.5 mm to 39.6 mm. Similarly right and left lateral movement showed a significant increase in the experimental but not the control group. There were no side effects during or 2 weeks after the study. <u>The results suggest strongly that PRFE is effective in reducing and eliminating TMJ arthralgia and increasing mandibular range of motion. The results also indicate that the PRFE is a safe treatment.</u> This study was supported by a grant from Orthosonix, Inc. Tappan, NJ.

Division: IADR/AADR/CADR General Session
Meeting: 2002 IADR/AADR/CADR General Session (San Diego, California)
Location: San Diego, California
Year: 2002
Final Presentation ID: 2329

Authors

- Al-badawi E. (Tufts University School of Dental Medicine, Boston, MA, USA)
- Mehta N. (Tufts University School of Dental Medicine, Boston, MA, USA)
- Forgione A. (Tufts University School of Dental Medicine, Boston, MA, USA)
- Lobo S. (Tufts University School of Dental Medicine, Boston, MA, USA)
- Zawawi K. (Tufts University School of Dental Medicine, Boston, MA, USA)

SESSION INFORMATION
Poster Session
TMD - Treatment and Pathophysiology
03/08/2002

Al-badawi E. (Tufts University School of Dental Medicine, Boston, MA, USA)
Mehta N. (Tufts University School of Dental Medicine, Boston, MA, USA)
Forgione A. (Tufts University School of Dental Medicine, Boston, MA, USA)
Lobo S. (Tufts University School of Dental Medicine, Boston, MA, USA)
Zawawi K. (Tufts University School of Dental Medicine, Boston, MA, USA)

Pulsed Radio Frequency Energy in Treatment of Temporomandibular Joint Pain

Cranio. 2004 Jan;22(1):10-20.

Efficacy of pulsed radio frequency energy therapy in temporomandibular joint pain and dysfunction.

Al-Badawi EA[1], Mehta N, Forgione AG, Lobo SL, Zawawi KH.

⊕ Author information

Abstract

This randomized double-blind study evaluated the effectiveness of pulsed radio frequency energy therapy (PRFE) in patients with temporomandibular joint arthralgia. Forty subjects (age range 22 to 55 yrs.) were assigned randomly into two equal groups: (1) Experimental group received PRFE using the Energex unit (Energex, Inc. Emerson, New Jersey) and (2) Control group received PRFE placebo treatment using a sham device. Both groups received six applications to the TMJ area over two weeks. Data were analyzed for the following times: baseline, first and second follow-up visits. Numerical Rating Scale scores for TMJ pain showed a significant reduction over time for the experimental group (mean = 6.13 to 3.05, $p < 0.001$). There was also a significant effect for the controls (mean = 5.35 to 4.20, $p = 0.01$). The effect for experimental subjects was a mean reduction of 3.07 versus 1.15 for controls. The significant reduction in controls was attributed to the placebo effect. The experimental group showed a significant increase in mouth opening (mean = 34.95 to 41.70 mm, $p = 0.002$), right lateral movement (mean = 7.85 to 10.80 mm, $p = 0.001$) and left lateral movement (mean = 7.65 to 10.85 mm, $p < 0.0001$). No significant ($p > 0.1$) change in the control group occurred for mouth opening (mean = 38.50 to 39.65 mm), right lateral movement (mean = 8.60 to 8.75 mm) and left lateral movement (mean = 8.50 to 8.80 mm). No side effects were reported during the treatment and the two week follow-up. These results suggest strongly that PRFE is a safe and effective treatment for TMJ arthralgia as well as for increasing mandibular range of motion.

PMID: 14964334 DOI: 10.1179/crn.2004.003

[illegible]

Real-Time Assessment of Coping Processes in TMD Treatment

Real-Time Assessment of Coping Processes in TMD Treatment

Objectives: To examine the role of coping skills in cognitive-behavioral treatment (CBT) for TMD. **Methods:** Thirty men and women reporting jaw pain for at least 3 months were recruited from the community and randomly assigned to either a standard care group (STD; n=15) or to standard care plus cognitive-behavioral treatment (STD+CBT; n=15). Both treatments lasted one hour per week for 6 weeks. STD consisted of placement of a flat plane splint plus prescription of nonsteroidal anti-inflammatory medication and soft diet. CBT added relaxation training, stress management, and cognitive restructuring to promote self-efficacy. Follow-ups occurred at posttreatment (6 weeks), and at 12, 18, and 24 weeks. Experience-sampling via handheld computer was used to record momentary jaw pain, mood state, self-efficacy, coping actions taken, and catastrophization four times per day for 7 days prior to and following treatment. **Results:** Analysis of daily aggregated pain scores recorded on the handheld computer showed that the STD+CBT condition resulted in a significantly greater decrease in pain from pre- to posttreatment than did the STD group [$F(1,60)=6.59$; $p < .05$]. As hypothesized, ANOVAs on momentary recordings indicated that the STD+CBT group recorded significantly greater increases in use of active coping than did the STD group [$F(1,60)=4.41$; $p < .05$], greater increases in use of distraction [$F(1,60)=3.97$; $p < .05$], and greater decreases in catastrophization [$F(1,60)=3.45$; $p < .05$]. Mixed model random effects regression (HLM) indicated that momentary pain in the week after treatment was predicted by baseline pain level, baseline coping self-efficacy, and by tendency to catastrophize measured at baseline. Two momentary measures also contributed to momentary pain: coping self-efficacy and catastrophization. **Conclusions:** CBT can result in increased self-efficacy, increased use of coping skills, and decreased catastrophizing. These changes are linked to treatment outcome.

Division: IADR/AADR/CADR General Session
Meeting: 2002 IADR/AADR/CADR General Session (San Diego, California)
Location: San Diego, California
Year: 2002
Final Presentation ID: 2409

Authors

- Litt Mark D. (University of Connecticut Health Center, Farmington, CT, USA)
- Napolitano Christopher (University of Connecticut Health Center, Farmington, CT, USA)
- Shafer David (University of Connecticut Health Center, Farmington, CT, USA)
- Goupil Michael (University of Connecticut Health Center, Farmington, CT, USA)

Litt Mark D. (University of Connecticut Health Center, Farmington, CT, USA)
Napolitano Christopher (University of Connecticut Health Center, Farmington, CT, USA)
Shafer David (University of Connecticut Health Center, Farmington, CT, USA)
Goupil Michael (University of Connecticut Health Center, Farmington, CT, USA)

SESSION INFORMATION
Oral Session
Orofacial Pain, TMD, and Xerostomia
03/08/2002

Real-Time Assessment of Coping Processes in TMD Treatment

Momentary pain and coping in temporomandibular disorder pain: Exploring mechanisms of cognitive behavioral treatment for chronic pain

来自Elsevier

收藏 引用 批量引用 报错 分享

作者 MD Litt , DM Shafer , CR Ibanez , DL Kreutzer , Z Tawfik-Yonkers

摘要 The purpose of this study was to determine whether cognitive鈥揵ehavioral treatment (CBT) operates by effecting changes in cognitions, affects, and coping behaviors in the context of painful episodes. Patients were 54 men and women with temporomandibular dysfunction-related orofacial pain (TMD) enrolled in a study of brief (6weeks) standard conservative treatment (STD) or standard treatment plus CBT (STD+CBT). Momentary affects, pain, and coping processes were recorded on a cell phone keypad four times per day for 7days prior to treatment, and for 14days after treatment had finished, in an experience sampling paradigm. Analyses indicated no treatment effects on general retrospective measures of pain, depression, or pain-related interference with lifestyle at post-treatment. However, mixed model analyses on momentary pain and coping recorded pre- and post-treatment indicated that STD+CBT patients reported greater decreases in pain than did STD patients, significantly greater increases in the use of active cognitive and behavioral coping, and significantly decreased catastrophization. Analyses of experience sampling data indicated that post-treatment momentary pain was negatively predicted by concurrent active coping, self-efficacy, perceived control over pain, and positive-high arousal affect. Concurrent catastrophization was strongly predictive of pain. Active behavioral coping and self-efficacy reported at the prior time point (about 3h previously) were also protective, while prior catastrophization and negative-high arousal mood were predictive of momentary pain. The results suggest that CB treatment for TMD pain can help patients alter their coping behaviors, and that these changes translate into improved outcomes. 收起

出版源 《Pain》 , 2009 , 145 (1-2) :160-168

被引量 43

Success of Hydroxyapatite vs. Titanium Implants: 5 year results

Success of Hydroxyapatite vs. Titanium Implants: 5 year results

Objectives: The purpose of this study was to compare the success of hydroxyapatite (HA) and titanium (Ti) coated implants in a 5 year randomized controlled clinical trial conducted in 2 centers.
Methods: Each of 120 edentulous patients received HA threaded, HA cylindrical, and Ti threaded implants in a randomized split-plot design using 5 or 6 implants per patient. Digital radiography provided yearly measurements of bone loss; calibrated clinicians measured, mobility, gingival index, plaque index, and recession.

Results: There was no significant difference in the gingival index, plaque index, or mobility at 5 years for HA and Ti coated implants. At 5 years, mean bone loss was significantly lower for HA cylinders (0.01±0.10 mm) and HA threaded (0.03±0.17 mm) than Ti threaded (0.5±0.20) ($p<.02$). A Kaplan-Meier analysis was used to compare the proportion of ailing implants, using the strict criterion that successful implants must lose less than 2 mm of bone support over 5 years. This analysis revealed that 95.2% of Ti threaded implants, 97.92% of HA coated threaded implants and 99.0% of HA cylinder implants were successful by this criterion ($p<0.06$).

Conclusions: These results indicate that all types of implants placed in this study had high success rates above 95%. Over 5 years the success rate tended to favor HA coated implants.

Division: IADR/AADR/CADR General Session
Meeting: 2002 IADR/AADR/CADR General Session (San Diego, California)
Location: San Diego, California
Year: 2002
Final Presentation ID: 74

Authors

- Jeffcoat Marjorie K. (University of Alabama at Birmingham, Birmingham, AL, USA)
- Mcglumphy Edwin A. (Ohio State University, Columbus, OH, USA)
- Reddy Michael S. (University of Alabama at Birmingham, Birmingham, AL, USA)
- Geurs Nico C. (University of Alabama at Birmingham, Birmingham, AL, USA)
- Proskin Howard M. (University of Alabama at Birmingham, Birmingham, AL, USA)

SESSION INFORMATION
Oral Session
Implant Periodontics I
03/06/2002

Jeffcoat Marjorie K. (University of Alabama at Birmingham, Birmingham, AL, USA)
Mcglumphy Edwin A. (Ohio State University, Columbus, OH, USA)
Reddy Michael S. (University of Alabama at Birmingham, Birmingham, AL, USA)
Geurs Nico C. (University of Alabama at Birmingham, Birmingham, AL, USA)
Proskin Howard M. (University of Alabama at Birmingham, Birmingham, AL, USA)

Success of Hydroxyapatite vs. Titanium Implants: 5 year results

Int J Oral Maxillofac Implants. 2003 May-Jun;18(3):406-10.

A comparison of hydroxyapatite (HA) -coated threaded, HA-coated cylindric, and titanium threaded endosseous dental implants.

Jeffcoat MK[1], McGlumphy EA, Reddy MS, Geurs NC, Proskin HM.

Author information

Abstract

PURPOSE: The purpose of this study was to compare the success of hydroxyapatite (HA) -coated and machined titanium (Ti) implants in a 5-year randomized, controlled clinical trial conducted at 2 centers.

MATERIALS AND METHODS: Each of 120 edentulous patients received HA-coated threaded, HA-coated cylindric, and machined Ti threaded implants in a randomized design using 5 or 6 implants. Digital radiographs allowed for yearly measurements of bone loss. Calibrated clinicians also measured mobility, Gingival Index, Plaque Index, probing depth, and recession. A Kaplan-Meier analysis was used to compare the proportion of ailing implants (defined as less than 2 mm of alveolar bone loss over 5 years) for each type of implant design. The criteria employed to assess implant outcome included the need for successful implants to lose less than 2 mm of bone support over the 5 years following placement of the prosthesis.

RESULTS: This analysis revealed that 95.2% of machined Ti threaded implants and 97.92% of HA-coated threaded implants were successful, while 99.0% of HA-coated cylindric implants experienced less than 2 mm of bone loss ($P < .06$).

DISCUSSION: All types of implants placed in this study had success rates above 95%.

CONCLUSION: Over 5 years, the success rate tended to favor HA-coated implants.

PMID: 12814316

The Effects of a Denture Adhesive on Masticatory Function

The Effects of a Denture Adhesive on Masticatory Function

Objectives: The objective of this study was to examine whether a denture adhesive is useful for the improvement of masticatory function.**Methods:**Sixteen edentulous subjects wearing well-fitting complete dentures volunteered to participate in this study. According to Kapur's classification for the adequacy of denture-bearing tissues, eight subject were categorized into "poor" group and the others into "fair or good" group. All the subjects were instructed to apply a denture adhesive (Correct , Shionogi, Osaka) onto maxillary and mandibular dentures according to manufacturer's direction. Masticatory performance was measured using the sieving method with peanuts. Maximum biting forces were recorded with maximal voluntary clench using a hand-held occlusal force meter (Model GM10, Nagano, Tokyo) unilaterally and bilaterally in the molar region. During the two-week test period, subjects wore dentures with a denture adhesive for half period and without it for another half. This order was randomly assigned. Experiments were carried out at the end of each half period.Whether the use of a denture adhesive and the adequacy of denture-bearing tissues influenced on masticatory performances and maximum biting force were tested using repeated measured two-way ANOVA. In addition, the improvements of masticatory for both groups were compared using t-test.**Results:**Both the use of a denture adhesive and the adequacy of denture-bearing tissues showed significant positive effects for all variables ($p<0.05$). The improvement of masticatory function by using denture adhesive was greater for subjects with poor denture-bearing tissues than with good or fair ($p<0.05$).**Conclusions:**It was concluded that the use of a denture adhesive showed the positive effect for masticatory function of complete denture wearers.

Division: IADR/AADR/CADR General Session
Meeting: 2002 IADR/AADR/CADR General Session (San Diego, California)
Location: San Diego, California
Year: 2002
Final Presentation ID: 1819

Authors

- Fujimori Takuto (Tokyo Medical and Dental University, Tokyo, N/A, Japan)
- Hirano Shigezo (Tokyo Medical and Dental University, Tokyo, N/A, Japan)
- Hayakawa Iwao (Tokyo Medical and Dental University, Tokyo, N/A, Japan)

SESSION INFORMATION
Poster Session
Denture Liners and Adhesives
03/08/2002

Fujimori Takuto (Tokyo Medical and Dental University, Tokyo, N/A, Japan)
Hirano Shigezo (Tokyo Medical and Dental University, Tokyo, N/A, Japan)
Hayakawa Iwao (Tokyo Medical and Dental University, Tokyo, N/A, Japan)

The Effects of a Denture Adhesive on Masticatory Function

Effects of a denture adhesive on masticatory functions for complete denture wearers--consideration for the condition of denture-bearing tissues--.

来自Europe PMC

收藏　引用　批量引用　报错　分享

作者　T Fujimori ， S Hirano ， I Hayakawa

摘要　Abstract The purpose of this study was to examine effects of a denture adhesive on masticatory functions for complete denture wearers considering the condition of denture-bearing tissues. Sixteen edentulous subjects wearing well-fitting complete dentures volunteered to participate in this study. According to the condition of denture-bearing tissues, subjects were divided into two groups; "good group" and "poor group". Maximum biting forces, masticatory performance, and electromyography of the masseter muscle during mastication were recorded with and without a denture adhesive. Durations of chewing burst and cycle, and coefficients of variation for these variables were calculated using electromyography recordings. Data were analyzed by using two-way repeated-measured ANOVA and paired t-test in order to assess the effect of the use of a denture adhesive. The use of the denture adhesive increased maximum biting force and provided rhythmic masseter muscle activity during mastication for both groups. Masticatory performance was improved and duration of chewing burst was decreased only for "poor group". It was concluded that the effects of the denture adhesive on masticatory functions were observed overall for both groups, and more significant for denture wearers with poor denture-bearing tissues than with good denture-bearing tissues.

收起

出版源　《J Med Dent Sci》 , 2016 , 49 (4) :151-156

[illegible]

Use of muscle exercise device in treatment of TMD

Use of muscle exercise device in treatment of TMD

Objectives: A muscle exercise device called the Bite Assist Exerciser (BAE) was evaluated with the objective of determining if it reduces muscle pain while increasing muscle function in patients with oral muscle dysfunction and /or muscle pain associated with specific temporomandibular joint disorders. **Methods:** 45 consecutive eligible patients who presented to the Brotman Facial Pain Center for treatment were asked to participate in the study. Patients were randomly assigned to one of three treatment groups. Group 1: any state of the art patient treatment normally rendered except for the use of the exercise device. Group 2: same as in group 1 except for the addition of the muscle exercise device. Group 3: home care instructions, home physiotherapy, use of the exercise device, and the use of pain medication, if needed. **Results:** There were no statistically significant differences found between the groups, but there was a trend noted toward the efficacy of the exercice device. Clinical measurements (interincisal opening, lateral movements, joint noises, etc.) demonstrated the least amount of differences between groups. Subjective pain levels were more reduced in groups using the exercise device but the differences did not reach statistical significance ($p=.08$). Similarly, the groups using the exercise device were more positive about their overall wellness and functioning levels during and after treatment but again not to a level that reached statistical significance($p=.1$). **Conclusions:** Muscle exercise devices could be useful in the treatment of TMD with muscular components. This was a small study and recommendations are made for future research.

Division: IADR/AADR/CADR General Session
Meeting: 2002 IADR/AADR/CADR General Session (San Diego, California)
Location: San Diego, California
Year: 2002
Final Presentation ID: 2331

Authors

- Grace Edward George (University of Maryland, Baltimore, MD, USA)
- Sarlani Eleni (University of Maryland, Baltimore, MD, USA)

SESSION INFORMATION
Poster Session
TMD - Treatment and Pathophysiology
03/08/2002

Grace Edward George (University of Maryland, Baltimore, MD, USA)
Sarlani Eleni (University of Maryland, Baltimore, MD, USA)

Use of muscle exercise device in treatment of TMD

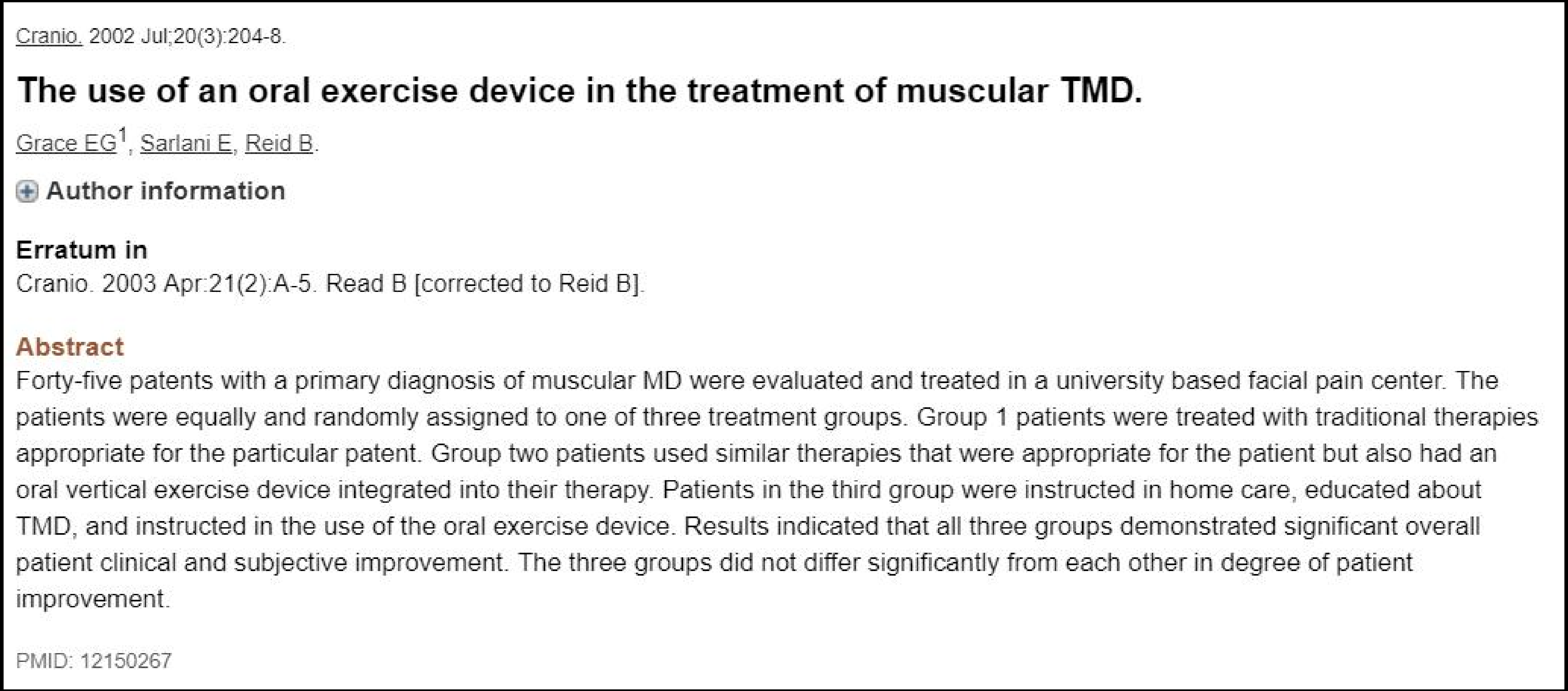

Cranio. 2002 Jul;20(3):204-8.

The use of an oral exercise device in the treatment of muscular TMD.

Grace EG[1], Sarlani E, Reid B.

Author information

Erratum in

Cranio. 2003 Apr;21(2):A-5. Read B [corrected to Reid B].

Abstract

Forty-five patents with a primary diagnosis of muscular MD were evaluated and treated in a university based facial pain center. The patients were equally and randomly assigned to one of three treatment groups. Group 1 patients were treated with traditional therapies appropriate for the particular patent. Group two patients used similar therapies that were appropriate for the patient but also had an oral vertical exercise device integrated into their therapy. Patients in the third group were instructed in home care, educated about TMD, and instructed in the use of the oral exercise device. Results indicated that all three groups demonstrated significant overall patient clinical and subjective improvement. The three groups did not differ significantly from each other in degree of patient improvement.

PMID: 12150267

2003

Accuracy of One- and Two-stage Impression Techniques - A Randomized Controlled Trial

Accuracy of One- and Two-stage Impression Techniques - A Randomized Controlled Trial

Objectives: The determination of complex three-dimensional alterations of the reproduction of prepared tooth by different impression techniques in-vivo is possible by a newly developed procedure using extraoral digitizing of master casts. This method overcomes the limitations of common methods, which are limited to a two-dimensional analysis. The aim of this study was to determine the three-dimensional accuracy of the reproduction of the finishing line of teeth prepared for single crowns by different dental impressions techniques (monophase, one-stage, two-stage) dependent on clinical variables (PI, GI, BOP, Probing Depth, location of the finishing line). Methods: For 48 patients with teeth prepared for single crowns a monophase-, one-stage- and two-stage-impression were taken in a randomized order. Master-casts were manufactured according to the Zeiser-technique using a type IV die stone for any impression and then optically digitized. The digitized data sets of the monophase- and two-stage-impression were compared with the CAD-surface model of the one-stage impression chosen as the reference. Differences between the CAD-surface-model and the digitized data set were analyzed quantitatively and qualitatively using Surfacer" (Imageware Inc., USA). The statistical analysis was performed by calculating linear regression models for repeated measures. Results: The finishing has been reproduced most exactly by the one-stage-putty and wash technique. Variables of significant influence for the reproduction of the finishing line were the impression technique (p= 0.0248), adhesion of blood at the impression (0.0349) and the probing depth (0.0358). Conclusion: The two-stage putty and wash impression showed significantly reduced accuracy compared with the one-stage techniques. This study was supported in part by the 3M ESPE AG, Seefeld, Germany.

Division: IADR/PER General Session
Meeting: 2003 IADR/PER General Session (Goteborg, Sweden)
Location: Goteborg, Sweden
Year: 2003
Final Presentation ID: 2637

Authors

- Luthardt Ralph G. (Dresden University of Technology, Dresden, N/A, Germany)

Luthardt Ralph G. (Dresden University of Technology, Dresden, N/A, Germany)

SESSION INFORMATION
Poster
Clinical Evaluations: Indirect Resins, Fixed Prosthodontics, Endodontics, Orthodontics
06/28/2003

Accuracy of One- and Two-stage Impression Techniques - A Randomized Controlled

Int J Prosthodont. 2009 May-Jun;22(3):296-302.

Randomized controlled clinical study on the accuracy of two-stage putty-and-wash impression materials.

Haim M[1], Luthardt RG, Rudolph H, Koch R, Walter MH, Quaas S.

Author information

1 Department of Prosthetic Dentistry, Dresden University of Technology, University Hospital Carl Gustav Carus Dresden, Dental School, Dresden, Germany.

Abstract

PURPOSE: The accuracy of dental impression taking is one major factor influencing the fit of crowns and fixed partial dentures. The aim of this study was to determine the accuracy of three-dimensional (3-D) tooth surface and subgingival tooth surface reproduction using three different silicone materials and the two-stage putty-and-wash technique.

MATERIALS AND METHODS: From 24 probands, three impressions each were taken with Express STD Putty/Wash (3M ESPE), Optosil/Xantopren L (Heraeus Kulzer), and an experimental ultralight body/putty material (3M ESPE) in a randomized order. The preliminary impression was cut according to established procedures for the two-stage putty-and-wash technique. Master casts were manufactured with a standardized procedure and optically digitized. The 3-D accuracy was analyzed with a computer-aided procedure. The Express STD putty-and-wash impressions were used as a reference. Linear models were used for the statistical analysis.

RESULTS: Mean deviations of 27.0 microm and -23.6 microm were found for Optosil/Xantopren L and 26.5 microm and -22.6 microm for the experimental material when analyzing 3-D surface reproduction. The tooth surface (buccal/oral) significantly influenced the accuracy of the surface reproduction. Optosil/Xantopren L showed a more complete reproduction of the subgingival tooth surface than either the experimental or reference materials.

CONCLUSION: The accuracy of the 3-D tooth surface reproduction as well as the reproduction of the subgingival tooth surface was not favorably influenced when the ultralight wash material was used with established cutting procedures for the preliminary impression.

PMID: 19548415

Association between Sleep Bruxism and Gastroesophageal Reflux

Association between Sleep Bruxism and Gastroesophageal Reflux

Objective: Sleep bruxism, defined as a stereotyped movement disorder or parasomnia, often causes abnormal tooth wear, masticatory muscle discomfort, and/or pain. Recently, several common features have been found separately on sleep bruxism and gastroesophageal reflux. The purpose of this study was to investigate the relationship between sleep bruxism and this reflux. Methods: Eight bruxism patients and 8 age- and sex- matched normal subjects participated in this study. Firstly, we measured the masticatory muscle activity (EMG), orofacial movements, and pH in the lower esophagus during sleep. Using the EMG and audiovisual data, we then scored bruxism episodes according to the research criteria of Lavigne et al. Acid reflux episodes were automatically scored. Secondly, we performed a randomized double-blind test to examine the effect of a secretory inhibitor of peptic regurgitation on sleep bruxism. The mean difference between groups was statistically analyzed. Results: Bruxism patients with a longer duration and higher frequency of bruxism episodes showed a significantly longer duration of acid reflux episodes than the normal subjects. The majority of bruxism episodes in all subjects occurred when the esophageal pH had decreased due to acid reflux. The frequency and duration of bruxism episodes after medication with a secretory inhibitor of peptic regurgitation were significantly lower and shorter, respectively, than those with the placebo medication. In fact, both parameters showed normal values after the secretory inhibitor of peptic regurgitation had been given. Conclusion: The results suggest that the occurrence of sleep bruxism is closely associated with acid reflux.

Division: IADR/PER General Session
Meeting: 2003 IADR/PER General Session (Goteborg, Sweden)
Location: Goteborg, Sweden
Year: 2003
Final Presentation ID: 2284

Miyawaki, Shouichi (Okayama University Graduate School of Medicine and Dentistry, Okayama, N/A, Japan)
Tanimoto, Yuko (Okayama University Graduate School of Medicine and Dentistry, Okayama, N/A, Japan)
Araki, Yoshiko (Okayama University Graduate School of Medicine and Dentistry, Okayama, N/A, Japan)
Katayama, Akira (Okayama University Graduate School of Medicine and Dentistry, Okayama, N/A, Japan)
Fujii, Akihito (Okayama University Graduate School of Medicine and Dentistry, Okayama, N/A, Japan)
Takano-yamamoto, Teruko (Okayama University Graduate School of Medicine and Dentistry, Okayama, N/A, Japan)

Authors

- Miyawaki, Shouichi (Okayama University Graduate School of Medicine and Dentistry, Okayama, N/A, Japan)
- Tanimoto, Yuko (Okayama University Graduate School of Medicine and Dentistry, Okayama, N/A, Japan)
- Araki, Yoshiko (Okayama University Graduate School of Medicine and Dentistry, Okayama, N/A, Japan)
- Katayama, Akira (Okayama University Graduate School of Medicine and Dentistry, Okayama, N/A, Japan)
- Fujii, Akihito (Okayama University Graduate School of Medicine and Dentistry, Okayama, N/A, Japan)
- Takano-yamamoto, Teruko (Okayama University Graduate School of Medicine and Dentistry, Okayama, N/A, Japan)

SESSION INFORMATION

Poster

Sensory-Motor Systems

06/27/2003

Association between Sleep Bruxism and Gastroesophageal Reflux

Sleep. 2003 Nov 1;26(7):888-92.

Association between nocturnal bruxism and gastroesophageal reflux.

Miyawaki S[1], Tanimoto Y, Araki Y, Katayama A, Fujii A, Takano-Yamamoto T.

Author information

1 Department of Orthodontics and Dentofacial Orthopedics, Okayama University Graduate School of Medicine and Dentistry, Okayama, Japan.

Abstract

STUDY OBJECTIVE: To examine the relationship between nocturnal bruxism and gastroesophageal reflux.

DESIGN: Controlled descriptive study and double-blind, placebo-controlled, clinical study.

SETTING: Portable pH monitoring, electromyography, and audio-video recordings were conducted during the night in the subjects' home.

PARTICIPANTS: Ten patients with bruxism and 10 normal subjects were matched for height, weight, age, and sex. They did not have symptoms of gastroesophageal reflux disease.

INTERVENTION: Medication with a proton pump inhibitor (ie, a gastric-acid-inhibiting drug).

MEASUREMENTS AND RESULTS: The bruxism group showed a significantly higher frequency of nocturnal rhythmic masticatory muscle activity (RMMA) episodes (mean +/- SD: 6.7 +/- 2.2 times per hour) and a higher frequency and percentage of time of gastroesophageal reflux episodes with a pH less than 4.0 and 5.0 (0.5 +/- 0.9 and 3.6 +/- 1.6 times per hour and 1.3% +/- 2.5% and 7.4% +/- 12.6%, respectively) than the control group (RMMA episodes: 2.4 +/- 0.9 times per hour; gastroesophageal reflux episodes: 0.0 +/- 0.0 and 0.1 +/- 0.3 times per hour and 0.0% +/- 0.0% and 0.0% +/- 0.0%, respectively). In the bruxism group, 100% of the gastroesophageal reflux episodes with a pH less than 3.0 and 4.0 included both an RMMA episode and an electromyographic burst, the duration of which was approximately 0.5 to 1.0 seconds, probably representing swallowing of saliva. The majority of gastroesophageal reflux episodes with a pH of 4.0 to 5.0 also included both an RMMA episode and an electromyographic burst in the control and bruxism groups (100% +/- 0.0% vs 70.7% +/- 16.5%), again probably due to swallowing of saliva. The remaining minority of gastroesophageal reflux episodes with a pH of 4.0 to 5.0 contained only an electromyographic burst (swallowing of saliva). The frequency of RMMA episodes after the release of the medication from the proton pump inhibitor, which increased the gastric and esophageal pH, was significantly lower than that after administration of the placebo in the control and bruxism groups (1.0 +/- 0.6 vs 1.9 +/- 3.2 times per hour, and 3.7 +/- 1.9 vs. 6.0 +/- 2.2 times per hour, respectively).

CONCLUSIONS: Nocturnal bruxism may be secondary to nocturnal gastroesophageal reflux, occurring via sleep arousal and often together with swallowing. The physiologic link between bruxism and the increase in salivation needs to be investigated.

Clinical and Radiographic Evaluation of Soft- and Hard-tissue Changes around Implants

Clinical and Radiographic Evaluation of Soft- and Hard-tissue Changes around Implants

Objective: The aim of this study was to evaluate the clinical and radiographic changes in the perimplantar tissues around one-stage implants with different smooth neck portion lengths previous and after functional prosthetic loading. Methods: Twelve one-stage implants were placed in adult patients with bilateral edentulous posterior mandibular ridges. The sites were randomly assigned into group I: treated with 2.8 mm smooth neck portion implants (n=6) and group II: treated with 1.8 mm smooth neck portion implants (n=6). The parameters Plaque Index (PlI), Bleeding Index (BI), Probing Depth (PD), Gingival Margin Level (GML), relative Clinical Attachment Level (r-CAL) and Optic Density (OD) were measured in the loading (4 months) and 12 months after implant placement. The radiographic parameter Osseous Level (OL) was measured in the implant placement, in the loading and 12 months after. Analysis of variance and the paired Student-t test were applied to detect difference over time and between groups. Results: The results showed significant differences ($P < 0.05$) for both groups between the evaluation periods for PD, r-CAL and OL. There were significant differences between groups I and II ($P < 0.05$) for r-CAL, however, no significant differences were found for PlI, BI, PD, GML, OD and OL. Conclusion: Bony loss occurred previous loading to support the soft tissues and maintain the biologic width irrespective of the smooth portion length.

Division: IADR/PER General Session
Meeting: 2003 IADR/PER General Session (Goteborg, Sweden)
Location: Goteborg, Sweden
Year: 2003
Final Presentation ID: 1621

Authors

- Joly Julio Cesar (University of Campinas - School of Dentistry at Piracicaba, Piracicaba, N/A, Brazil)
- Lima Afm (University of Campinas - School of Dentistry at Piracicaba, Piracicaba, N/A, Brazil)
- Silva Rc (University of Campinas - School of Dentistry at Piracicaba, Piracicaba, N/A, Brazil)

Joly Julio Cesar (University of Campinas - School of Dentistry at Piracicaba, Piracicaba, N/A, Brazil)
Lima Afm (University of Campinas - School of Dentistry at Piracicaba, Piracicaba, N/A, Brazil)
Silva Rc (University of Campinas - School of Dentistry at Piracicaba, Piracicaba, N/A, Brazil)

SESSION INFORMATION

Poster

Human Implant Studies

06/27/2003

Clinical and Radiographic Evaluation of Soft- and Hard-tissue Changes around Implants

J Periodontol. 2003 Aug;74(8):1097-103.

Clinical and radiographic evaluation of soft and hard tissue changes around implants: a pilot study.

Joly JC[1], de Lima AF, da Silva RC.

Author information

Abstract

BACKGROUND: The aim of this study was to evaluate the clinical and radiographic changes in the peri-implant tissues around one-stage implants with different smooth neck portion lengths before and after functional prosthetic loading.

METHODS: Twelve one-stage implants were placed in adult patients with bilateral edentulous posterior mandibular ridges. The sites were randomly assigned into two groups of six each: group 1: 2.8 mm neck implants and group 2: neck implants. The parameters plaque index (PI), gingival index (GI), probing depth (PD), gingival margin level (GML), relative clinical attachment level (r-CAL), and optical density (OD) were measured at loading (4 months) and 12 months after implant placement. The radiographic parameter osseous level (OL) was measured at implant placement, loading, and at 12 months. Analysis of variance and the paired Student t test were used to detect difference over time and between groups.

RESULTS: The results showed significant differences ($P<0.05$) for both groups for PD, r-CAL, and OL for intragroup comparisons over time. However, no significant differences were found for PI, GI, PD, GML, OD, and OL for between-group comparisons.

CONCLUSION: Bony loss occurred before loading, supporting the soft tissues and maintaining the biologic width irrespective of the smooth portion length.

PMID: 14514222 DOI: 10.1902/jop.2003.74.8.1097

Clinical Evaluation of CAD/CAM-generated Composite Inlays: Three-year

Clinical Evaluation of CAD/CAM-generated Composite Inlays: Three-year Report

Objective: To evaluate the clinical performance of a composite material compared to a porcelain material for CAD/CAM-generated adhesive inlays. This is the three year report of a randomized clinical trial.
Experimental Methods: Two trained clinicians placed 40 porcelain (Vita Mark II/Vita) and 40 composite (Paradigm MZ100/3M-ESPE) CAD/CAM inlays, in 43 patients, divided between 37 molars and 43 bicuspids. A CAD/CAM unit (Cerec 2/Sirona) was used to fabricate all restorations using COS 1.21 software. Following computergraphic design of the inlay, the prefabricated block to be used for the specific restoration was randomly assigned. Both restorative materials were cemented with a total etch technique using Single Bond (3M-ESPE) and a dual cured resin cement (RelyX-ARC Resin Cement/3M-ESPE).

Results: Tooth sensitivity was evaluated by report to cold stimulus (number of teeth): 1 week: Composite (0), Porcelain (1), with no sensitivity reported in either group after 2 weeks, 6 months, 1 year, 2 years, and 3 years. All restorations were evaluated by two examiners using a modified USPHS rating. The percentage alpha scores (baseline, 6 months, 1 year, 2 years, 3 years) were: margin adaptation - Composite (100, 97, 91, 68, 66), Porcelain (97, 92, 76, 73, 65); inlay fracture - Composite (100, 100, 100, 97, 100), Porcelain (97, 94, 89, 91, 88); color match - Composite (100, 97, 97, 91, 91), Porcelain (85, 58, 65, 58, 59); margin discoloration – Composite (100, 100, 91, 91, 88), Porcelain (100, 100, 100, 91, 91). The percentage of alpha scores for anatomic form, tooth fracture, caries, and surface finish were 95% or greater for both materials at all recall intervals.

Conclusions: The composite inlays performed equally as well as the porcelain inlays at 3 years in all categories with clinical advantages noted in inlay fracture and color match.
Supported by 3M/ESPE.

Division: IADR/PER General Session
Meeting: 2003 IADR/PER General Session (Goteborg, Sweden)
Location: Goteborg, Sweden
Year: 2003
Final Presentation ID: 68

Authors

- Fasbinder D.j. (University of Michigan, Ann Arbor, MI, USA)
- Dennison J.b. (University of Michigan, Ann Arbor, MI, USA)
- Heys D (University of Michigan, Ann Arbor, MI, USA)
- Lampe K. (University of Zurich, Zurich, N/A, Switzerland)

Fasbinder D.j. (University of Michigan, Ann Arbor, MI, USA)
Dennison J.b. (University of Michigan, Ann Arbor, MI, USA)
Heys D (University of Michigan, Ann Arbor, MI, USA)
Lampe K. (University of Zurich, Zurich, N/A, Switzerland)

SESSION INFORMATION
Oral
Clinical Evaluations of Ceramics/Indirect Metallic Materials
06/25/2003

Clinical Evaluation of CAD/CAM-generated Composite Inlays: Three-year Report

J Am Dent Assoc. 2005 Dec;136(12):1714-23.

The clinical performance of CAD/CAM-generated composite inlays.

Fasbinder DJ[1], Dennison JB, Heys DR, Lampe K.

Author information

Abstract

BACKGROUND: The authors conducted a study to evaluate the longitudinal clinical performance of a resin-based composite (Paradigm, 3M ESPE, St. Paul, Minn.) for computer-aided design/computer-aided manufacturing (CAD/CAM)-generated adhesive inlays.

METHODS: The researchers used a CAD/CAM unit (CEREC 2, Sirona Dental Systems, Bensheim Germany) to fabricate 40 porcelain (Vita Mark II, Vita Zahnfabrik, Bad Säckingen, Germany) and 40 resin-based composite (Paradigm, 3M ESPE) inlays. Both restorative materials were cemented with a total-etch technique using Single Bond dental adhesive (3M ESPE) and a dual-cured resin cement (RelyX ARC Adhesive Resin Cement, 3M ESPE). Two examiners evaluated the inlays using modified U.S. Public Health Service (PHS) criteria at six months, one year, two years and three years.

RESULTS: No sensitivity was reported for either material at any recall period. There was no significant difference between the two materials relative to margin adaptation at three years. Margin adaptation initially was very good for both materials, with an increase in margin detection due to apparent wear of the resin-based composite luting agent. There was a significant difference in color match between the two materials at three years, with 91.4 percent of the resin-based composite inlays and 58.8 percent of the porcelain inlays rated Alfa.

CONCLUSIONS AND CLINICAL IMPLICATIONS: The resin-based composite inlays had a significantly better color match at three years than did the porcelain inlays. Resin-based composite CAD/CAM inlays performed as well as porcelain CAD/CAM inlays after three years of clinical service.

PMID: 16383055

Comparison of Habit Awareness Training and Interocclusal Appliances as Treatments for TMD Pain

Comparison of Habit Awareness Training and Interocclusal Appliances as Treatments for TMD Pain

Objectives: This study tested the hypothesis that a behaviorally-oriented habit awareness program emphasizing awareness and reduction of parafunctional activities would be as effective in the treatment of temporomandibular disorder pain as splint therapy. Methods: A randomized trial design was employed. Thirteen TMD patients diagnosed with myofascial pain and/or arthralgia (according to the Research Diagnostic Criteria) were assigned to either an interocclusal appliance therapy group or to a behavioral intervention group. Patients assigned to the interocclusal appliance therapy group received a flat plane splint, adjusted for comfort. Patients were instructed to avoid contact with the splint. Patients wore the splint during the day and at night and were instructed to taper their use of the splint during the day as treatment progressed. Patients assigned to the behavioral intervention group were instructed on the impact that parafunctional clenching has on pain. They were also given a demonstration of the impact of tooth contact or "jaw setting" on the activity of the masticatory muscles. They were then given a pager and instructed to check tooth position and masticatory muscle tension when they were paged. If they detected contact or tension at this or any other time, they were instructed to relax the masticatory muscles. The pagers contacted these patients approximately every two hours during the day, but not at night. The initial phase of treatment lasted one month, and follow-up measures were collected approximately 9 months after the end of treatment. Results: Self-report measures of pain decreased significantly for both groups, $p < .001$, but pain levels did not differ between groups. Conclusions: Behavioral treatment techniques emphasizing awareness and control of parafunctional activity and masticatory muscle tension may be effective treatments for TMD-related pain. This project was conducted with the support of grant DE13563 from the National Institute of Dental and Craniofacial Research

Division: IADR/PER General Session
Meeting: 2003 IADR/PER General Session (Goteborg, Sweden)
Location: Goteborg, Sweden
Year: 2003
Final Presentation ID: 783

Authors

- Glaros Alan G. (University of Missouri - Kansas City School of Dentistry, Kansas City, MO, USA)
- Lausten Leonard (University of Missouri - Kansas City School of Dentistry, Kansas City, MO, USA)
- Kim Nellie (University of Missouri - Kansas City School of Dentistry, Kansas City, MO, USA)
- Horrigan Kellylora (University of Missouri - Kansas City School of Dentistry, Kansas City, MO, USA)

Glaros Alan G. (University of Missouri - Kansas City School of Dentistry, Kansas City, MO, USA)
Lausten Leonard (University of Missouri - Kansas City School of Dentistry, Kansas City, MO, USA)
Kim Nellie (University of Missouri - Kansas City School of Dentistry, Kansas City, MO, USA)
Horrigan Kellylora (University of Missouri - Kansas City School of Dentistry, Kansas City, MO, USA)

SESSION INFORMATION
Oral
TMD - Pathophysiology, Diagnosis and Treatment
06/26/2003

Comparison of Habit Awareness Training and Interocclusal Appliances as Treatments for TMD Pain

Appl Psychophysiol Biofeedback. 2007 Dec;32(3-4):149-54. Epub 2007 Jun 15.

Comparison of habit reversal and a behaviorally-modified dental treatment for temporomandibular disorders: a pilot investigation.

Glaros AG[1], Kim-Weroha N, Lausten L, Franklin KL.

Author information

Abstract

This study tested the hypothesis that a habit reversal program emphasizing awareness and reduction of masticatory muscle activity would significantly reduce pain in patients diagnosed with chronic temporomandibular disorder (TMD) and would be a competitive alternative to a behaviorally-modified dental intervention. Eight individuals diagnosed with TMD were randomly assigned to a splint therapy or habit reversal group. Patients in the splint group received an interocclusal appliance (splint) fabricated from acrylic and were instructed to wear the splint day and night up to a maximum of 20 h per day. Patients in the habit reversal group were given a pager and instructed to check tooth position and masticatory muscle tension when paged. Paging occurred approximately once every 2 h during the day, but not at night. Both groups were instructed to avoid tooth contact and relax the masticatory muscles during the 4 weeks of active treatment. Outcome data were collected at 1 month and 1 year post-treatment intervals. Pain decreased significantly for both groups and did not differ between groups. Habit reversal may be as effective as a behaviorally-modified splint therapy for TMD-related pain.

PMID: 17570054 DOI: 10.1007/s10484-007-9039-5

Cost-effectiveness of Mandibular Conventional and Two-implant Overdentures

Cost-effectiveness of Mandibular Conventional and Two-implant Overdentures

Economic considerations often dictate choice of therapeutic options in restorative dentistry. Therefore, it is important to measure cost of providing treatments along with efficacy in clinical trials so that the two can be compared directly. Results of such cost-effectiveness studies provide patients and third-party payers, including governments, with the necessary information they need to make appropriate health-care choices. Objective: To determine the cost-effectiveness of mandibular 2-implant overdentures and conventional dentures opposed by conventional maxillary dentures. Methods: In a randomized clinical trial, we measured direct treatment costs (e.g. materials and labor) and oral health-related quality of life using the OHIP-20 in edentulous seniors (65-75 years; n=30) who received a maxillary denture and either a mandibular conventional denture (CD) or a two-implant overdenture with ball attachments (IOD) up to one year post-treatment. Data for subsequent years were estimated from values obtained from published data and a panel of experts. Results: Using an average life expectancy of 17.9 years, the equalized annual costs were CAD$ 398.60 for CD and $ 528.40 for IOD (p<0.001). The equalized annual values for the OHIP-20 outcome were 47.01 units for CD and 31.29 for IOD treatment (p<0.05), indicating that oral health-related quality of life was significantly better (by 33%) in the IOD group. The additional annual cost needed to achieve this level of oral health-related quality of life benefit over conventional treatment is approximately $131 CAD. Conclusion: The initial cost of mandibular two-implant overdentures is significantly more than conventional dentures, but the former provide a much better oral health-related quality of life. By comparing costs and benefits over the expected lifetime of the subjects, it can be seen that quality of life can be greatly improved by IOD treatment for a relatively modest annual investment. Supported by the Canadian Institutes for Health Research and Straumann Canada Limited.

Division: IADR/PER General Session
Meeting: 2003 IADR/PER General Session (Goteborg, Sweden)
Location: Goteborg, Sweden
Year: 2003
Final Presentation ID: 1554

Authors

- Feine Jocelyne (McGill University, Montreal, QC, Canada)
- Heydecke Guido (Albert-Ludwigs-University, D-79106 Freiburg, N/A, Germany)
- Takanashi Yoshiaki (McGill University, Montreal, QC, Canada)
- Thomason J Mark (University of Newcastle, Newcastle upon Tyne, N/A, England)
- Lund James P. (McGill University, Montreal@, QC, Canada)
- Penrod John R (McGill University, Montreal, QC, Canada)

SESSION INFORMATION
Poster
Oral Health Care, Epidemiology, Quality of Life, Oral Function and Morphology in Old Age
06/27/2003

Feine Jocelyne (McGill University, Montreal, QC, Canada)
Heydecke Guido (Albert-Ludwigs-University, D-79106 Freiburg, N/A, Germany)
Takanashi Yoshiaki (McGill University, Montreal, QC, Canada)
Thomason J Mark (University of Newcastle, Newcastle upon Tyne, N/A, England)
Lund James P. (McGill University, Montreal·@, QC, Canada)
Penrod John R (McGill University, Montreal, QC, Canada)

Cost-effectiveness of Mandibular Conventional and Two-implant Overdentures

J Dent Res. 2005 Sep;84(9):794-9.

Cost-effectiveness of mandibular two-implant overdentures and conventional dentures in the edentulous elderly.

Heydecke G[1], Penrod JR, Takanashi Y, Lund JP, Feine JS, Thomason JM.

Author information

Abstract

Implementation of new therapies is usually governed by financial considerations, so efficacy studies should also include cost comparisons. The cost and effectiveness of mandibular conventional dentures (CD, n = 30) and two-implant overdentures (IOD, n = 30) were compared in elderly subjects. Effectiveness (Oral Health Impact Profile, OHIP-20) and cost were measured up to one year post-treatment. Data for subsequent years were estimated by the Delphi method. Using an average life expectancy of 17.9 years, the equalized annual costs (in Canadian dollars) were dollar 399 for CD and dollar 625 for IOD ($p < 0.001$), and the equalized annual values for the outcome (OHIP-20) were 47.0 for CD and 31.3 for IOD treatment ($p < 0.05$). These values translate into a yearly additional cost for IOD treatment of dollar 14.41 per OHIP-20 point. These results are key to the implementation of programs to provide this form of therapy for edentulous adults.

PMID: 16109986 DOI: 10.1177/154405910508400903

Effect of Wearing Complete Dentures on Body Equilibrium as the Dynamic Body

Effect of Wearing Complete Dentures on Body Equilibrium as the Dynamic Body Balance

Objectives: It has been suggested that change of occlusion affects the postural reflexes and various muscle functions of the head and neck, resulting in changes of the head posture. Furthermore, these changes may have an impact on the body posture. This study examined the influence of wearing complete dentures on body equilibrium in edentulous elderly under dynamic condition.
Materials and Methods: Gait analysis was performed in nineteen edentulous volunteers (five men and ten women, 63 to 82y old, mean age 73.0y) who have been treated at the dental hospital of Tokyo Medical and Dental University for new complete dentures and volunteered to participate in this study. 10 meters of steady walking was recorded with a telemetric measuring device and a speedometer. The measurements were randomly carried out three times each when wearing and not wearing dentures. A paired t test was performed to compare the gate cycle, gate velocity, stride length, and the coefficient variation for the gate cycle with and without dentures with a 0.05 level of significance.

Results: When wearing dentures, the gait cycle was significantly reduced compared to that when not wearing dentures (p=0.0015). The gait velocity and stride length significantly increased when wearing dentures (p=0.0007 and p=0.0015, respectively). The coefficient variation of the gait cycle significantly decreased when wearing dentures (p=0.0041), demonstrating that when wearing denture, the walking rhythm became more stable.

Conclusion: Gait analysis was performed with nineteen edentulous patients to detect an influence of wearing complete dentures to body equilibrium. 1) The gait cycle was stabilized and reduced when wearing dentures. 2) The stride length and the gait velocity were increased when wearing dentures. It was suggested that body equilibrium of edentulous elderly may be maintained well by wearing complete dentures.

Division: IADR/PER General Session
Meeting: 2003 IADR/PER General Session (Goteborg, Sweden)
Location: Goteborg, Sweden
Year: 2003
Final Presentation ID: 312

Authors

- Watanabe Ikki (Tokyo Medical and Dental University, Tokyo, N/A, Japan)
- Fujinami Yukiko (Tokyo Medical and Dental University, Tokyo, N/A, Japan)
- Hirano Shigezo (Tokyo Medical and Dental University, Tokyo, N/A, Japan)
- Keh Ensheng (Tokyo Medical and Dental University, Tokyo, N/A, Japan)
- Hayakawa Iwao (Tokyo Medical and Dental University, Tokyo, N/A, Japan)

SESSION INFORMATION
Poster
Removable Prostheses: Clinical Outcomes and Assessment
06/26/2003

Watanabe Ikki (Tokyo Medical and Dental University, Tokyo, N/A, Japan)
Fujinami Yukiko (Tokyo Medical and Dental University, Tokyo, N/A, Japan)
Hirano Shigezo (Tokyo Medical and Dental University, Tokyo, N/A, Japan)
Keh Ensheng (Tokyo Medical and Dental University, Tokyo, N/A, Japan)
Hayakawa Iwao (Tokyo Medical and Dental University, Tokyo, N/A, Japan)

Effect of Wearing Complete Dentures on Body Equilibrium as the Dynamic Body Balance

J Prosthodont Res. 2010 Jan;54(1):42-7. doi: 10.1016/j.jpor.2009.09.002. Epub 2009 Oct 9.

Effect of complete dentures on body balance during standing and walking in elderly people.

Okubo M[1], Fujinami Y, Minakuchi S.

Author information

Abstract

PURPOSE: To investigate the influence of wearing complete dentures on postural control in standing and walking.

METHODS: Thirty-four edentulous patients participated in this study. All the subjects were wearing complete dentures, and the dentures were adjusted or replaced with new dentures when necessary. Measurements were performed under two conditions: wearing dentures and not wearing dentures. Standing stability was evaluated by the locus of center of mass, and gait stability was evaluated by the gait velocity, stride and gait cycle. In addition, gait stability was also evaluated by the maximum acceleration, maximum angle rate, lateral equilibrium, root mean square and harmonic ratio with a tri-axial accelerometer at a sampling rate of 66 Hz. Differences for the locus of center of mass, gait velocity, gait cycle and stride length were assessed with the paired t test ($P<0.05$). Other outcomes were compared with the Wilcoxon signed-rank test ($P<0.05$).

RESULTS: With denture wear, the locus of center of mass was significantly shortened, and the gait velocity and harmonic ratio of the vertical angle rate were significantly increased; though other parameters showed no differences. Complete dentures produced an effect on the stability of edentulous patients under both static and dynamic conditions.

CONCLUSIONS: These results indicate that wearing complete dentures may be an effective aid to maintain and improve balance and control for elderly people.

PMID: 19819207 DOI: 10.1016/j.jpor.2009.09.002

Effects of Occlusal Splint on Salivary Flow Rate, Acid Reflux, and Sleep Bruxism

Effects of Occlusal Splint on Salivary Flow Rate, Acid Reflux, and Sleep Bruxism

Objective: Various occlusal splints have been extensively used to control sleep bruxism, but their mechanisms of action remain controversial. Recently, we reported that sleep bruxism may be closely associated with saliva swallowing and acid reflux. The purpose of this study was to investigate the effects of an occlual splint on salivary flow rate, acid reflux, and sleep bruxism. Methods: The subjects were 7 bruxism patients and 7 age- and sex- matched normal subjects. Firstly, we measured the salivary flow rate with or without the splint. These subjects were classified into two groups again according to changes in salivary flow after splint application. Secondly, we measured the masticatory muscle activity (EMG) and pH in the lower esophagus during sleep with or without the occlusal splint. The order of measurements made with and without the splint was randomly determined. Using EMG and audiovisual data, we scored bruxism episodes according to the research criteria of Lavigne et al. Acid reflux episodes were automatically scored. The mean difference between groups was statistically analyzed. Results: Bruxism patients showed a significantly longer duration of acid reflux episodes, and higher frequency and longer duration of bruxism episodes during sleep than the normal subjects. In the group with a higher rate of increase in salivary flow after the splint application, the duration of acid reflux and bruxism episodes per hour of sleep after splint application were significantly shorter than without the splint application. Conclusion: The results suggest that the increase in salivary flow rate caused by splint application may be an important treatment mechanism for sleep bruxism in relation to acid reflux.

Division: IADR/PER General Session
Meeting: 2003 IADR/PER General Session (Goteborg, Sweden)
Location: Goteborg, Sweden
Year: 2003
Final Presentation ID: 2285

Katayama, Akira (Okayama University Graduate School of Medicine and Dentistry, Okayama, N/A, Japan)
Miyawaki, Shouichi (Okayama University Graduate School of Medicine and Dentistry, Okayama, N/A, Japan)
Tanimoto, Yuko (Okayama University Graduate School of Medicine and Dentistry, Okayama, N/A, Japan)
Araki, Yoshiko (Okayama University Graduate School of Medicine and Dentistry, Okayama, N/A, Japan)
Fujii, Akihito (Okayama University Graduate School of Medicine and Dentistry, Okayama, N/A, Japan)
Takano-yamamoto, Teruko (Okayama University Graduate School of Medicine and Dentistry, Okayama, N/A, Japan)

Authors

- Katayama, Akira (Okayama University Graduate School of Medicine and Dentistry, Okayama, N/A, Japan)
- Miyawaki, Shouichi (Okayama University Graduate School of Medicine and Dentistry, Okayama, N/A, Japan)
- Tanimoto, Yuko (Okayama University Graduate School of Medicine and Dentistry, Okayama, N/A, Japan)
- Araki, Yoshiko (Okayama University Graduate School of Medicine and Dentistry, Okayama, N/A, Japan)
- Fujii, Akihito (Okayama University Graduate School of Medicine and Dentistry, Okayama, N/A, Japan)
- Takano-yamamoto, Teruko (Okayama University Graduate School of Medicine and Dentistry, Okayama, N/A, Japan)

SESSION INFORMATION
Poster
Sensory-Motor Systems
06/27/2003

Effects of Occlusal Splint on Salivary Flow Rate, Acid Reflux, and Sleep Bruxism

Am J Orthod Dentofacial Orthop. 2004 Sep;126(3):367-70.

Salivary flow rates during relaxing, clenching, and chewing-like movement with maxillary occlusal splints.

Miyawaki S[1], Katayama A, Tanimoto Y, Araki Y, Fujii A, Yashiro K, Takano-Yamamoto T.

Author information

1 Department of Orthodontics and Dentofacial Orthopedics, Okayama University Graduate School of Medicine and Dentistry, Japan.

Abstract

The purpose of this study was to test the hypothesis that the application of occlusal splints increases the diurnal salivary flow rate both in bruxism patients and in normal subjects. Salivary flow rates in 16 adult volunteers (8 bruxism patients and 8 sex- and age-matched control subjects) were measured with the spitting method. There was no significant difference in the salivary flow rate with or without splints between the control and bruxism groups. In all subjects, the salivary flow rates with splints were significantly higher than those without splints during relaxing, clenching, and chewing-like movement. The salivary flow rate during the chewing-like movement was significantly higher than that during relaxing and clenching, irrespective of splint application. The results suggest that maxillary occlusal splints might stimulate salivary secretion, particularly during chewing-like movement, in both bruxism patients and normal subjects.

PMID: 15356502 DOI: 10.1016/S0889540604004871

Is a Stabilization Appliance Effective in the Treatment of Tension-type Headache in Patients with TMD of Myogenous Origin?

Is a Stabilization Appliance Effective in the Treatment of Tension-type Headache in Patients with TMD of Myogenous Origin?

Objective: To evaluate tension-type headache before and after treatment with a stabilisation and a control appliance both in a short and a long-term perspective.
Methods: 60 patients with TMD of mainly myogenous origin were studied. The patients were selected from those referred to the Department of Stomatognathic Physiology, Faculty of Odontology, Malmö University during a period of approximately 2 years. The study was performed as a randomised controlled trial evaluating the treatment effect on tension-type headache after 10 weeks, 6 and 12 months. The patients were randomly assigned to at treatment (T) group, given a stabilisation appliance and a control (C) group, given a control appliance. Patients who reported a negative treatment outcome of overall subjective symptoms, had any discomfort associated with the appliances, or both at the 10 weeks follow-up either had their appliance readjusted (1 patient from the T-group) or were given another appliance (17 patients from the C-group) creating a mixed M-group.

Results: At start of the study 88 % of all patients reported a frequency of headache from rarely up top daily. In the T-group 73% and in the C-group 70% of the patients reported headache at least once a week or more before treatment. At the 10-weeks follow-up, a statistically significant improvement of reported tension-type headache between T and C-group and within T-group regarding frequency of headache was found. The number of patients with headache once a week or more decreased significantly in the T-group at the 6- months as well as the 12 months follow-ups.

Conclusion: The stabilisation appliance seems to have a positive effect on tension-type headache both in a short and a long-term perspective in patients with TMD of mainly myogenous origin.

Division: IADR/PER General Session
Meeting: 2003 IADR/PER General Session (Goteborg, Sweden)
Location: Goteborg, Sweden
Year: 2003
Final Presentation ID: 2865

Authors

- Ekberg Ewacarin (Malmö University, Malmö, N/A, Sweden)
- Nilner Maria (Malmö University, Malmö, N/A, Sweden)

Ekberg Ewacarin (Malmö University, Malmö, N/A, Sweden)
Nilner Maria (Malmö University, Malmö, N/A, Sweden)

SESSION INFORMATION
Poster
TMD - Pathophysiology, Psychophysiology and Treatment
06/28/2003

Is a Stabilization Appliance Effective in the Treatment of Tension-type Headache in Patients with TMD of M[illegible]

Acta Odontol Scand. 2004 Dec;62(6):343-9.

Treatment outcome of appliance therapy in temporomandibular disorder patients with myofascial pain after 6 and 12 months.

Ekberg E[1], Nilner M.

⊕ **Author information**

Abstract

AIM: To compare the long-term effect of treatment with a stabilization appliance (group T) and treatment with a control appliance (group C) in temporomandibular disorder (TMD) patients with myofascial pain.

METHODS: In this controlled trial, 60 patients (mean age 29 years) with myofascial pain were evaluated after 10 weeks of treatment with either a stabilization appliance or a control appliance. All 60 patients were then assigned to 1 of 3 groups according to demand for treatment. Seventeen patients from group C requested another appliance and were given a stabilization appliance, thus creating a mixed group (group M).

RESULTS: A significant difference in improvement of overall subjective symptoms in an intent-to-treat analysis between groups T and C was found at the follow-ups. In a survival analysis of treatment compliance, a significant difference was found between groups T and C. At the 6- and 12-month follow-ups, a significant reduction in myofascial pain, as measured on a visual analog scale, was found in all three groups. A significant decrease in frequency and intensity of myofascial pain was found in group T at the follow-ups. A significant decrease in number of tender sites on the masticatory muscles was found in group T at the follow-ups.

CONCLUSION: The results support the conclusion that the positive treatment outcome obtained by use of a stabilization appliance to alleviate the signs and symptoms in patients with myofascial pain persisted after 6 and 12 months. Most patients in groups T and M reported positive changes in overall subjective symptoms in this trial. We therefore recommend use of the stabilization appliance in the treatment of TMD patients with myofascial pain.

PMID: 15848979 DOI: 10.1080/00016350410010063

Selected Characteristics of a New Polyvinylsiloxane Impression Material: A Randomized Clinical Trial

Selected Characteristics of a New Polyvinylsiloxane Impression Material: A Randomized Clinical Trial

Purpose: This study evaluated the ability of a new polyvinylsiloxane (PVS) impression material (Affinis, Coltene Whaledent) to achieve satisfactory final impressions for indirect fixed restorations when used by inexperienced clinicians (3rd year dental students) as compared to a widely used PVS impression material (Express, 3M ESPE). The Null-Hypothesis was tested: there is no difference between impression materials. Methods: 115 patients treated in the LSU School of Dentistry Junior Student Clinic for indirect fixed restorations and meeting the inclusion criteria were randomly assigned to either one of two treatment groups. Two calibrated examiners evaluated the first impression of prepared posterior teeth at a magnification of x10 for acceptability (no voids or bubbles). Position of tooth, type of preparation, preparation finish line, and gingival bleeding score were recorded. All statistical tests were performed with the level of significance set at .05. Results: Fisher-Freeman-Halton test was used to test for associations between material and type of preparation, preparation finish line, and gingival bleeding score. Significant association was found between type of material (p=0.0385). Logistic regression was used to determine the effect of material on success of the impression (acceptable/unacceptable). Material was highly significant in the logistic model (p<0.001) with the odds favoring acceptable impression seven and a half times greater for impressions made with Affinis as compared to Express (OR=7.481; 95% CI for OR: 2.574, 21.747). 92% of the impressions made with Affinis were acceptable; as compared to 60 % of the impressions made with Express. Conclusions: Within this study's limitations, the new PVS impression material resulted in a significantly higher rate of acceptable impressions than the reference PVS material. This study was sponsored by Coltene Whaledent.

Division: IADR/PER General Session
Meeting: 2003 IADR/PER General Session (Goteborg, Sweden)
Location: Goteborg, Sweden
Year: 2003
Final Presentation ID: 2638

Authors

- Blatz Markus B. (Louisiana State University School of Dentistry, New Orleans, LA, USA)
- Burgess J. O. (Louisiana State University School of Dentistry, New Orleans, LA, USA)
- Sadan Avishai (Louisiana State University School of Dentistry, New Orleans, LA, USA)
- Mercante D (Louisiana State University School of Dentistry, New Orleans, LA, USA)
- Chiche G. J. (Louisiana State University School of Dentistry, New Orleans, LA, USA)

SESSION INFORMATION
Poster
Clinical Evaluations: Indirect Resins, Fixed Prosthodontics, Endodontics, Orthodontics
06/28/2003

Blatz Markus B. (Louisiana State University School of Dentistry, New Orleans, LA, USA)
Burgess J. O. (Louisiana State University School of Dentistry, New Orleans, LA, USA)
Sadan Avishai (Louisiana State University School of Dentistry, New Orleans, LA, USA)
Mercante D (Louisiana State University School of Dentistry, New Orleans, LA, USA)
Chiche G. J. (Louisiana State University School of Dentistry, New Orleans, LA, USA)

Selected Characteristics of a New Polyvinylsiloxane Impression Material: A Randomized [illegible]

Quintessence Int. 2005 Feb;36(2):97-104.

Selected characteristics of a new polyvinyl siloxane impression material--a randomized clinical trial.

Blatz MB[1], Sadan A, Burgess JO, Mercante D, Hoist S.

Author information

Abstract

OBJECTIVES: This study evaluated the ability of a new polyvinyl siloxane impression material (Affinis, Coltène/Whaledent, material A) to obtain final impressions free of bubbles and voids for indirect fixed cuspal-coverage restorations. The results were compared to a control polyvinyl siloxane impression material (material B). Both materials were handled by inexperienced clinicians (undergraduate dental students) in student clinics.

METHOD AND MATERIALS: One-hundred and thirty patients who were treated in the Louisiana State University School of Dentistry Junior Student Clinic for indirect fixed cuspal-coverage restorations and who met the inclusion criteria were randomly assigned to either one of two treatment groups, group A (n = 65) or group B (n = 65). Two calibrated examiners evaluated the first impression of prepared posterior teeth at a magnification of 10x for acceptability (no voids or bubbles). Position of tooth, type of preparation, preparation finish line (Class I-V), and gingival bleeding scores were recorded. All statistical tests were performed with the level of significance set at .05.

RESULTS: The Fisher-Freeman-Halton test did not reveal significant associations between material and gingival bleeding score (P = .492). Significant differences in the location of the preparation finish line between materials were observed (P = .0096); material A was more frequently used in cases where the preparation finish line was located at least 2 mm subgingivally. Logistic regression was used to assess the effect of the material on the success of the impression (acceptable/ unacceptable). Material was highly significant in the logistic model (P < .001) with an odds in favor of an acceptable impression being eight times higher with material A than with material B (odds ratio = 8.00; 95% confidence index for odds ratio: 2.832, 22.601). The 60/65 (92.3%) impressions made with material A and 39/65 (60%) impressions made with material B were rated "acceptable."

CONCLUSION: The new polyvinyl siloxane impression material provided a significantly higher proportion of impressions free of bubbles and voids than the control polyvinyl siloxane material.

PMID: 15732545

Sensitivity of the Human Jaw-stretch Reflex: Effects of TMJ Anesthesia and of Jaw Gape

Sensitivity of the Human Jaw-stretch Reflex: Effects of TMJ Anesthesia and of Jaw Gape

Objective: To study the roles of afferent sensory inputs from the temporomandibular joint (TMJ) and of muscle length in the modulation of the jaw-stretch reflex in humans. Methods: Reflexes were evoked in both the masseter muscles and the temporalis muscles under standardized conditions in 11 young women (mean age ± SD = 25.2 ± 4.1 yrs). The study was performed in two sessions; experimental conditions were jaw gape and injection of local anesthetics. For jaw gape, 4 mm, 14 mm, and 24 mm were used in random order. One-ml TMJ injections (carbocaine, 10 mg/ml, versus isotonic saline, 0.9%) were given in a randomized, double blind manner. When a participant received carbocaine during the first session, isotonic saline was injected during the second one. In every participant, a total of 480 reflexes was evoked at pre-stimulus EMG activity levels of 15% of the maximum voluntary contraction level, and by jaw displacements of 1 mm with a rise time of 10 ms. Results: No significant differences in normalized peak-to-peak amplitude were found between carbocaine and isotonic saline. ANOVA and post-hoc paired t-tests did show, however, a significant effect of jaw gape for the left masseter and anterior temporalis muscles, with the 14-mm gape having the highest amplitude. Conclusion: Blocking the afferent sensory input (including the mechanoreceptors) from the TMJ seem to have no influence on the sensitivity of the human jaw-stretch reflex. Instead, muscle spindles are the most likely receptors to be responsible for the reflex modulation that was observed in the present study. Supported by the Danish National Research Foundation and the Netherlands Institute of Dental Sciences (IOT).

Division: IADR/PER General Session
Meeting: 2003 IADR/PER General Session (Goteborg, Sweden)
Location: Goteborg, Sweden
Year: 2003
Final Presentation ID: 2442

Authors

- Lobbezoo F. (Academic Centre for Dentistry Amsterdam, Amsterdam, N/A, Netherlands)
- Wang K. (Aalborg University, Aalborg, N/A, Denmark)
- Aartman I.h.a. (Academic Centre for Dentistry Amsterdam, Amsterdam, N/A, Netherlands)
- Svensson P. (Aarhus University, Aarhus, N/A, Denmark)

Lobbezoo F. (Academic Centre for Dentistry Amsterdam, Amsterdam, N/A, Netherlands)
Wang K. (Aalborg University, Aalborg, N/A, Denmark)
Aartman I.h.a. (Academic Centre for Dentistry Amsterdam, Amsterdam, N/A, Netherlands)
Svensson P. (Aarhus University, Aarhus, N/A, Denmark)

SESSION INFORMATION
Oral
TMD and Orofacial Sensory-Motor Function
06/28/2003

Sensitivity of the Human Jaw-stretch Reflex: Effects of TMJ Anesthesia and of Jaw Gape

Clin Neurophysiol. 2003 Sep;114(9):1656-61.

Effects of TMJ anesthesia and jaw gape on jaw-stretch reflexes in humans.

Lobbezoo F[1], Wang K, Aartman IH, Svensson P.

Author information

Abstract

OBJECTIVE: To study the roles of afferent sensory inputs in the temporomandibular joint (TMJ) and of muscle length in the modulation of the jaw-stretch reflex in humans.

METHODS: Reflexes were evoked in both the masseter and temporalis muscles under standardized conditions in 11 young women. The study was performed in two sessions; experimental conditions were jaw gape and injection of local anesthetics. For jaw gape, 4, 14, and 24 mm were used in random order. One milliliter TMJ injections (carbocaine, 10 mg/ml, versus isotonic saline, 0.9%) were given in a randomized, double blind manner. When a participant received carbocaine during the first session, isotonic saline was injected during the second one. A total of 480 reflexes were evoked in every participant.

RESULTS: No significant differences were found between carbocaine and isotonic saline. ANOVA and post hoc paired t tests did show, however, a significant effect of jaw gape for the left masseter and anterior temporalis muscles, with the 14 mm gape having the highest amplitude.

CONCLUSIONS: Blocking the afferent sensory input (including the mechanoreceptors) from the TMJ seems to have no influence on the sensitivity of the human jaw-stretch reflex. Instead, muscle spindles are the most likely receptors to be responsible for the reflex modulation that was observed in the present study.

PMID: 12948794

Speech Errors with Maxillary Fixed and Removable Implant

Speech Errors with Maxillary Fixed and Removable Implant Prostheses

One of the primary functions of the oral cavity in humans is the production of speech. Unretentive conventional maxillary dentures are often replaced by implant-supported prostheses, but this sometimes reduces the quality of speech.
Objective: The aim of this study was to determine if the quality of speech is dependent on the design of maxillary implant prostheses.

Materials & Methods: Data were gathered in a two-arm parallel, randomized within-subject crossover trial. Four to six maxillary implants were inserted into 26 patients who had previously received mandibular implant prostheses. In the first arm, we compared maxillary implant retained fixed prostheses (FP) with long-bar implant overdentures without palates (LBO1): five subjects received the FP first and eight the LBO1. In the second arm, seven subjects first received maxillary long-bar overdentures with palates (LBOP) and six were given overdentures without palatal coverage (LBO2). After two months, the prostheses within each trial arm were changed and the second prosthesis was also worn for two months. Audio recordings of test words from a French language speech battery were made after each subject had been wearing each prosthesis for at least 2 months. Two lay judges rated the % of stops, fricatives and vowels that were correctly produced with each prosthesis.

Results: In the FP-LBO1 comparison, ratings indicated that participants were able to produce a significantly higher % of sounds correctly with the LBO1. Between treatment differences were significant for stops and fricatives ($p<0.01$, t-tests), but there was no significant difference for vowels. In the LBOP-LBO2 comparison, there were no significant differences for any of the three classes of sounds.

Conclusion: Maxillary implant overdentures with and without palates enable patients to produce more intelligible speech than fixed prostheses.

Supported by the Canadian Institutes of Health Research and NobelBiocare Canada.

Division: IADR/PER General Session
Meeting: 2003 IADR/PER General Session (Goteborg, Sweden)
Location: Goteborg, Sweden
Year: 2003
Final Presentation ID: 1361

Authors

- Heydecke Guido (School of Dentistry, University of Freiburg Medical Center, Freiburg, N/A, Germany)
- Mcfarland David (Faculté de Médecine, Université de Montréal, Montréal, QC, Canada)
- Lund James P. (McGill University, Montreal, QC, Canada)
- Feine Jocelyne S. (McGill University, Montreal, QC, Canada)

SESSION INFORMATION
Oral
Maxillofacial Treatment, Masticatory Performance and Forces, Speech
06/27/2003

Heydecke Guido (School of Dentistry, University of Freiburg Medical Center, Freiburg, N/A, Germany)
Mcfarland David (Faculté de Médecine, Université de Montréal, Montréal, QC, Canada)
Lund James P. (McGill University, Montreal, QC, Canada)
Feine Jocelyne S. (McGill University, Montreal, QC, Canada)

Speech Errors with Maxillary Fixed and Removable Implant Prostheses

J Dent Res. 2004 Mar;83(3):236-40.

Speech with maxillary implant prostheses: ratings of articulation.

Heydecke G[1], McFarland DH, Feine JS, Lund JP.

Author information

Abstract

Speech is often perturbed after placement of maxillary implant-retained prostheses. We tested the hypothesis that the rate of speech errors varies with prosthetic design. Thirty edentulous subjects with mandibular implant prostheses entered two within-subject crossover trials. Subjects wore maxillary fixed prostheses and removable long-bar overdentures (Trial 1), or overdentures with and without palates (Trial 2). Test words from a French language speech battery were recorded after each prosthesis had been worn for two months. The percentages of stops, fricatives, and vowels correctly perceived by lay judges were calculated. Subjects produced a significantly higher percentage of sounds correctly with overdentures than with fixed prostheses. Between-treatment differences were significant for stops and fricatives ($p < 0.01$), but not for vowels. There were no significant differences in error rates between the two overdentures. In conclusion, maxillary implant overdentures with and without palates enable patients to produce more intelligible speech than fixed prostheses.

PMID: 14981126 DOI: 10.1177/154405910408300310

Two-year Clinical Effectiveness of a Self-etch Adhesive in Cervical Lesions

Two-year Clinical Effectiveness of a Self-etch Adhesive in Cervical Lesions

Objectives: The purpose of this double-blind, split-mouth, randomized controlled clinical trial was to test the hypothesis that using a self-etch adhesive additional acid-etching of enamel is beneficial in terms of retention and marginal adaptation of Class-V restorations. **Methods:** 28 patients received two or four restorations randomly following two experimental protocols: (1) A 'mild' self-etch adhesive (Clearfil SE, Kuraray) was applied following a self-etch approach on both enamel and dentin (C-SE non-etch). (2) Similar application of Clearfil SE, but including beforehand selective acid-etching of the enamel cavity margins with 40% phosphoric acid (C-SE etch). Clearfil AP-X (Kuraray) was used as restorative composite for all 96 restorations. The clinical effectiveness was recorded in terms of retention (R), perfect marginal integrity (MI), absence of small incisal marginal defects (I-MI), absence of small cervical marginal defects (C-MI), absence of clinical microleakage (CM), absence of caries recurrence (CR) and absence of post-operative sensitivity (PS) after 2 years of clinical service. **Results:** No restoration losses were recorded. Clinical microleakage was rarely observed. Almost no differences between both groups were found for the diverse parameters evaluated except for the number of small marginal defects (mainly incisal), which was significantly higher in the C-SE non-etch group (McNemar p=0.0156).

	R	MI	I-MI	C-MI	CM	CR	PS
**C-SE non-etch**	100%	55%	71%	81%	94%	100%	97%
**C-SE etch**	100%	77%	87%	87%	97%	100%	97%

Conclusion: The clinical effectiveness of the mild two-step self-etch adhesive Clearfil SE was excellent after 2 years of clinical service. Although in general no difference in clinical performance was recorded when Clearfil SE was applied following either of the experimental protocols, a tendency towards more marginal defects at the enamel side was noticed when enamel was not beforehand etched with phosphoric acid. However, these defects were small and of clinical negligible relevance.

Division: IADR/PER General Session
Meeting: 2003 IADR/PER General Session (Goteborg, Sweden)
Location: Goteborg, Sweden
Year: 2003
Final Presentation ID: 911

Authors

- Peumans, Marleen (Catholic University of Leuven, Leuven, N/A, Belgium)
- Van Meerbeek, Bart (Catholic University of Leuven, Leuven, N/A, Belgium)
- De Munck, Jan (Catholic University of Leuven, Leuven, N/A, Belgium)
- Lambrechts, Paul (Catholic University of Leuven, Leuven, N/A, Belgium)

SESSION INFORMATION
Poster
Clinical Evaluations of Dentine/Enamel Adhesives
06/26/2003

Peumans, Marleen (Catholic University of Leuven, Leuven, N/A, Belgium)
Van Meerbeek, Bart (Catholic University of Leuven, Leuven, N/A, Belgium)
De Munck, Jan (Catholic University of Leuven, Leuven, N/A, Belgium)
Lambrechts, Paul (Catholic University of Leuven, Leuven, N/A, Belgium)

Two-year Clinical Effectiveness of a Self-etch Adhesive in Cervical Lesions

Eur J Oral Sci. 2005 Dec;113(6):512-8.

Three-year clinical effectiveness of a two-step self-etch adhesive in cervical lesions.

Peumans M[1], Munck J, Van Landuyt K, Lambrechts P, Van Meerbeek B.

Author information

1 Leuven BIOMAT Research Cluster, Department of Conservative Dentistry, School of Dentistry, Oral Pathology and Maxillo-facial Surgery, Catholic University of Leuven, Leuven, Belgium. marleen.peumans@med.kuleuven.ac.be

Abstract

A 3-yr randomized, controlled prospective study evaluated the clinical effectiveness of a mild two-step self-etch adhesive, Clearfil SE, in Class-V non-carious lesions. The hypothesis tested was that prior selective etching of enamel with phosphoric acid does not affect the 3-yr clinical performance of this adhesive. A total of 100 lesions in 29 patients were randomly restored in one or two pairs, according to two experimental protocols: (i) application of Clearfil SE according to the instructions of the manufacturer (C-SE non-etch); and (ii) similar application of Clearfil SE with prior etching of enamel cavity margins with phosphoric acid (C-SE etch). Clearfil AP-X was used as a restorative material. At 3 yr, 90% of the restorations were examined for retention, marginal integrity, marginal discoloration, caries recurrence, postoperative sensitivity, and preservation of tooth vitality. An excellent retention rate (100%) was noted after 3 yr of clinical functioning. Only one restoration of the C-SE etch group was clinically unacceptable owing to the presence of a severe cervical marginal defect. A pairwise comparison between both groups showed a significant difference only in the number of small marginal defects at the enamel side, which was higher in the C-SE non-etch group. These incisal defects were small and clinically irrelevant. Superficial marginal discoloration increased slightly in the C-SE non-etch group and was related to the higher frequency of small incisal marginal defects. In this latter group, localized marginal discoloration was observed significantly more in smokers. In conclusion, the clinical performance of the mild two-step self-etch adhesive, Clearfil SE, remained excellent after 3 yr of clinical functioning. Additional etching of the enamel cavity margins was not critical for its clinical performance.

PMID: 16324142 DOI: 10.1111/j.1600-0722.2005.00256.x

2004

3-year Clinical Evaluation of Experimental Glass-Ceramic Crowns: In-vivo Elemental Analysis

3-year Clinical Evaluation of Experimental Glass-Ceramic Crowns: In-vivo Elemental Analysis

The clinical performance of the newly developed all-ceramic systems on the posterior teeth and the mechanism of failure are relatively unknown. ***Objectives***: This study reports the results of prospective clinical evaluation and elemental analysis of three ceramic systems {non-layered, experimental hot-pressed ceramic (EC)[1], Procera-AllCeram (PA)[2] and metal ceramic (PFM)[3]} over 3-year period. ***Methods***: A total of 90 posterior crowns were randomized into three groups equally in 48 patients and assessed over 3 years using modified USPHS criteria. Clinical images were taken after using staining dye to highlight surface changes. The data were analyzed using Kruskal-Wallis non-parametric statistical test and subsidiary follow up Mann-Whitney was performed with Bonferroni correction. Crowns that developed cracks were removed and the surfaces analyzed. Quantitative and qualitative element analysis was performed on contact and non-contact areas using SEM, back-scattered imaging and energy-dispersive spectroscopy. ***Results***: USPHS evaluation showed visible roughness, wear and deformity in PA crowns at the region of occlusal contact after 36 months. A number of PA showed chipping of the layering material, whereas EC and PFM showed less changes clinically. One PA crown was rated Delta and removed due to fracture while one EC was rated Charlie and removed due to crack propagation after 36 months. Kruskal-Wallis showed a highly significant difference ($p<0.0005$) in alpha scores in between the three crown systems. Mann-Whitney showed all the groups were significantly different. Elemental analysis showed a difference in chemical composition between contact and non-contact areas in EC and PA with disappearance and change in the weight % of some elements after 3 years. ***Conclusions:*** EC showed a comparable clinical performance with PA crowns, but improved durability according to USPHS criteria. Chemical analysis suggests that there is preferential elemental loss in EC and changes in weight % for PA and EC.

1: Ivoclar-Vivadent, 2: NobelBiocare/Ducera, 3: Panadent/Ivoclar.

Division: IADR/AADR/CADR General Session
Meeting: 2004 IADR/AADR/CADR General Session (Honolulu, Hawaii)
Location: Honolulu, Hawaii
Year: 2004
Final Presentation ID: 197

Authors

- Etman Maged Kamal (Guy's, King's and St. Thomas's Dental Institute, London, N/A, United Kingdom)
- Woolford M. J. (Guy's, King's and St. Thomas's Dental Institute, London, N/A, United Kingdom)
- Watson T.f. (Guy's, King's and St. Thomas's Dental Institute, London, N/A, United Kingdom)

SESSION INFORMATION
Oral Session
Arthur R. Frechette Research Award Finalists
03/10/2004

Etman Maged Kamal (Guy's, King's and St. Thomas's Dental Institute, London, N/A, United Kingdom)
Woolford M. J. (Guy's, King's and St. Thomas's Dental Institute, London, N/A, United Kingdom)
Watson T.f. (Guy's, King's and St. Thomas's Dental Institute, London, N/A, United Kingdom)

3-year Clinical Evaluation of Experimental Glass-Ceramic Crowns: In-vivo Elemental Analysis

J Prosthet Dent. 2010 Feb;103(2):80-90. doi: 10.1016/S0022-3913(10)60010-8.

Three-year clinical evaluation of two ceramic crown systems: a preliminary study.

Etman MK[1], Woolford MJ.

Author information

Abstract

STATEMENT OF PROBLEM: The clinical performance and failure mechanisms of recently introduced ceramic crown systems used to restore posterior teeth have not been adequately examined.

PURPOSE: The purpose of this prospective clinical study was to evaluate and compare the clinical performance of 2 new ceramic crown systems with that of metal ceramic crowns using modified United States Public Health Services (USPHS) criteria.

MATERIAL AND METHODS: Ninety posterior teeth requiring crown restorations in 48 patients were randomized into 3 equal groups (n=30) for which different crown systems were used: an experimental hot-pressed glass ceramic based on a modified lithium disilicate ceramic (IPS e.max Press), an alumina-coping-based ceramic (Procera AllCeram), and a metal ceramic (Simidur S 2 veneered with IPS Classic Porcelain). The crowns were assessed over 3 years using the modified USPHS criteria. Crowns that developed visible cracks were sectioned and removed, and the surfaces were analyzed using a scanning electron microscope (SEM). The data were analyzed using the Kruskal-Wallis nonparametric statistical test, followed by the Mann-Whitney test with Bonferroni correction (alpha=.05).

RESULTS: USPHS evaluation showed that the IPS e.max Press and metal ceramic crowns experienced fewer clinical changes than Procera AllCeram. Visible roughness, wear, and deformity were noticed in occlusal contact areas of Procera AllCeram crowns. SEM images showed well defined wear facets in both ceramic crown systems. Kruskal-Wallis tests showed a significant difference ($P<.05$) in Alpha scores among the 3 crown systems. Mann-Whitney tests showed significant differences among groups.

CONCLUSIONS: IPS e.max Press crowns demonstrated clinical behavior comparable to Procera AllCeram and metal ceramic crowns, but the wear resistance of this crown type was superior to the Procera AllCeram crowns, according to modified USPHS criteria.

PMID: 20141812 DOI: 10.1016/S0022-3913(10)60010-8

Clinical Comparison of Proximal Contacts of Class II Composite Restorations

Clinical Comparison of Proximal Contacts of Class II Composite Restorations

Objectives: To investigate clinically the differences in proximal contact strengths of Class II posterior composite resin restorations (PCR's) placed with three different techniques.

Methods: 86 Patients (61 female, 25 male) from two dental practices volunteered after informed consent. Patients were randomly assigned to one of three groups: Group 1) Pre-wedging + Tofflemire (Produits Dentaire SA) + 1001c matrix (Hawe Neos), including pressure by hand-instrument during polymerization, Group 2) Pre-wedging + Palodent sectional matrix + Bi-Tine separation ring (Dentsply), Group 3) Pre-wedging + Contact Matrix System sectional matrix + separation ring (Danville Materials). Proximal contact strength (PCS) was measured in Newton using a modified Tooth Pressure Meter as described by Dšrfer et al. (2000) directly before (PCSB) and after (PCSA) restoration placement. Data were statistically analyzed using ANOVA at p=0.05 using Bonferroni correction for multiple testing.

Results: Differences in PCS (PCSA-PCSB) were observerd between the three techniques. The Tofflemire system produced statistically significant larger PCS reduction compared to the Palodent system (p<0.01).

	PCSA-PCSB (N)	95% CI	Contact Matrix	Tofflemire
Palodent	0.54	[-1.062; 2.14]	p= 0.36	p< 0.01
Contact Matrix	-0.73	[-2.294; 0.84]		p= 0.21
Tofflemire	-2.17	[-3.364; -0.98]		

Conclusion: Class II posterior composite resin restorations inserted using a sectional matrix system combined with separation rings demonstrated the strongest proximal contacts compared to the circumferential matrix system. This study was supported by Danville Materials, San Ramon, CA and Dentsply Caulk, Milford, DE.

Division: IADR/AADR/CADR General Session
Meeting: 2004 IADR/AADR/CADR General Session (Honolulu, Hawaii)
Location: Honolulu, Hawaii
Year: 2004
Final Presentation ID: 1549

Authors

- Loomans, Bas A.c. (University Medical Centre Nijmegen, Nijmegen, N/A, Netherlands)
- Opdam, Niek J.m. (University Medical Centre Nijmegen, Nijmegen, N/A, Netherlands)
- Roeters, F.j.m. (University Medical Centre Nijmegen, Nijmegen, N/A, Netherlands)
- Bronkhorst, Ewald M. (University Medical Centre Nijmegen, Nijmegen, N/A, Netherlands)
- Dörfer, Christof E. (Ruprecht-Karls-University Heidelberg, Heidelberg, N/A, Germany)

Loomans, Bas A.c. (University Medical Centre Nijmegen, Nijmegen, N/A, Netherlands)
Opdam, Niek J.m. (University Medical Centre Nijmegen, Nijmegen, N/A, Netherlands)
Roeters, F.j.m. (University Medical Centre Nijmegen, Nijmegen, N/A, Netherlands)
Bronkhorst, Ewald M. (University Medical Centre Nijmegen, Nijmegen, N/A, Netherlands)
Dörfer, Christof E. (Ruprecht-Karls-University Heidelberg, Heidelberg, N/A, Germany)

SESSION INFORMATION
Oral Session
Special Topics
03/12/2004

Oper Dent. 2006 Nov-Dec;31(6):688-93.

Comparison of proximal contacts of Class II resin composite restorations in vitro.

Loomans BA[1], Opdam NJ, Roeters FJ, Bronkhorst EM, Burgersdijk RC.

Author information

1 Department of Preventive and Restorative Dentistry, Radboud University Nijmegen Medical Centre, College of Dental Sciences, Nijmegen, The Netherlands. b.loomans@dent.umcn.nl

Abstract

This study investigated the tightness of the proximal contact when placing posterior resin composite restorations with circumferential and sectional matrix systems in an in vitro model using a special measuring device (Tooth Pressure Meter). A manikin model was used with an artificial first molar in which an MO-preparation was ground, simulating the clinical situation of an amalgam replacement. This preparation was duplicated, resulting in 160 identically prepared teeth. These teeth were divided into 8 groups (n=20). In 2 groups, circumferential matrix bands (flat or contoured) in a Tofflemire retainer were applied. In the remaining 6 groups, 3 different separation rings were combined with 2 types of sectional matrix bands. All the cavities were restored using Clearfil Photo Bond and Clearfil AP-X. The tightness of the proximal contact was measured using the Tooth Pressure Meter. Data were statistically analyzed using SPSS 12. ANOVA was used to find differences in proximal contact tightness between the groups. Tukey tests were used to find differences between the homogeneous subgroups. The use of sectional matrices combined with separation rings resulted in tighter proximal contacts compared to when circumferential systems were used ($p<0.001$). The use of these devices is therefore recommended when posterior resin composite restorations are placed.

PMID: 17153978 DOI: 10.2341/05-133

Computerized Shade Selection in Matching Anterior Metal-Ceramic Crowns

Computerized Shade Selection in Matching Anterior Metal-Ceramic Crowns

Efficacy of a computerized shade selection systemin matching anterior metal-ceramic crowns. A double-blind clinical pilot study.

A.J. Raigrodski and G.J. Chiche: Louisiana State University, New Orleans, LA

Objectives: To determine whether anterior crowns fabricated using a computerized shade selection system (ShadeScan,™ Cynovad) (experimental procedure) match adjacent teeth better than anterior crowns fabricated using conventional shade prescription and slides with Vita Lumin shade guide (control). **Methods:** Five subjects, requiring a crown on a maxillary central incisor, were assigned to the study. Two crowns were fabricated for each subject. Shade for either the first or second crown was selected randomly using either the experimental procedure or the control. The ceramist fabricated the first crown using data obtained from experimental procedure, and the second using the control. The duration of each shade selection procedure was recorded as well. Each crown was tried-in in a double-blind manner and subjectively evaluated for match to the adjacent teeth with modified Ryge criteria. Crowns were rated using an ordinal scale as either Romeo (excellent), Sierra (acceptable), Tango (unacceptable), or Victor (failure). **Results:** The table demonstrates ratings:

Data was analyzed within each subject at the significance level of alpha=0.05.Fisher's exact test showed no statistically significant difference in color matching between the experimental and control procedures. The duration of the control procedure was 14.4 ± 5 minutes, and the experimental procedure was 5.2 ± 3.3 minutes. A paired t-test showed the difference was significant (p=0.0045). **Conclusion:** Matching level of crowns fabricated with the ShadeScan system was not different from crowns fabricated using the control. It took significantly less time to record the shade with the ShadeScan. Further studies are required using an adequate sample.

Supported by a research grant from Cynovad.

Division: IADR/AADR/CADR General Session
Meeting: 2004 IADR/AADR/CADR General Session (Honolulu, Hawaii)
Location: Honolulu, Hawaii
Year: 2004
Final Presentation ID: 1477

Authors

- Raigrodski Ariel J. (University of Washington, Seattle, WA, USA)
- Chiche Gerard J. (Louisiana State University, New Orleans, LA, USA)

SESSION INFORMATION
Oral Session
Assessment of Color and Esthetics
03/11/2004

Raigrodski Ariel J. (University of Washington, Seattle, WA, USA)
Chiche Gerard J. (Louisiana State University, New Orleans, LA, USA)

Computerized Shade Selection in Matching Anterior Metal-Ceramic Crowns

Quintessence Int. 2006 Nov-Dec;37(10):793-802.

Efficacy of a computerized shade selection system in matching the shade of anterior metal-ceramic crowns--a pilot study.

Raigrodski AJ[1], Chiche GJ, Aoshima H, Spiekerman CF.

Author information

Abstract

OBJECTIVE: The purpose of this study was to determine whether anterior crowns fabricated using a computerized shade selection system (ShadeScan, Cynovad) (experimental procedure) match adjacent teeth better than anterior crowns fabricated using conventional shade prescription and clinical slides (control).

METHOD AND MATERIALS: Five subjects who required a crown to restore a maxillary central incisor were selected. Two metal-ceramic crowns were fabricated for each incisor, 1 using the experimental procedure and 1 using the control method. The shade selection method to be used for the first and second crowns was randomly assigned. The duration of each procedure was recorded. Each restoration was tried-in in a double-blind manner and evaluated for its level of match to adjacent teeth using modified Ryge criteria. Data were analyzed within each subject using descriptive statistics and paired t test (alpha = .05).

RESULTS: In 40% of the cases, both procedures did equally well. In the remaining 60% of the cases the control procedure (two-thirds of the cases) performed better than the experimental procedure (one-third of the cases). Duration of the control procedure was 14.4 +/- 5 minutes, and the experimental procedure was 5.2 +/- 3.3 minutes. A paired t test showed the difference was significant (P = .0045).

CONCLUSION: The level of matching of crowns fabricated with the ShadeScan system was not different from crowns fabricated using the control. However, it took significantly less time to record the shade with the ShadeScan system.

PMID: 17078278

Design of Multicenter Phase III Clinical Trial for Maxillofacial Prosthetics

Design of Multicenter Phase III Clinical Trial for Maxillofacial Prosthetics

Chlorinated polyethylene (CPE) was developed by us in the 1980s as a low-cost alternative skin replacement material for costly silicone rubber maxillofacial prostheses. CPE is thermoplastic, molded in gypsum at 110°C, followed by surface coloration and lamination. A Phase II clinical trial, conducted by Gulf South Research Institute at Charity Hospital/New Orleans, showed equivalency to the silicone control material.
Objectives: A Phase III multicenter clinical trial grant has been awarded to the University of Louisville, the University of Texas-Houston Dental Branch, and 3 cancer centers: J.G. Brown Cancer Center (Louisville), M.D. Anderson Cancer Center (Houston), and Toronto Sunnybrook Regional Cancer Centre (Toronto). Hypothesis: determine non-inferiority of the CPE (experimental) versus silicone (control) material, based on functional/subjective characteristics and quality of life of the patient.

Methods: Outcome measures are: a) anaplastologist/ dental laboratory technician; b) 2 independent prosthodontists; c) failure evaluation, and d) the patient for functional/subjective details, and e) the Toronto Outcomes Measure of Craniofacial Prostheses for quality of life. Longevity is estimated after the crossover trial; patients wear the better prosthesis as long as they wish.

Results: Guided by 3 power analyses from previous studies (patient satisfaction: 71; frayed margin length: 65; Toronto Quality-of-Life: 71 patients), the trial treats 100 extraoral maxillofacial prosthetic patients in a randomized single-crossover double-blind design to determine non-inferiority. Control prostheses of Medical Silastic Adhesive A/MDX4-4210 are compared with low-cost CPE; both prostheses are made to the same sculpture, using the same skin adhesive. Each prosthesis is randomly and alternately delivered to the patient, worn 4 months, with outgoing and incoming evaluations performed.

Conclusions: A unique clinical trial design for maxillofacial prosthetics has been devised, in this case to compare facial materials. Other variables are possible in facial prosthetics for clinical trials using this experimental design.

Supported by cooperative agreement 1-U01-DE014543-01A1 from the NIDCR, NIH.

Division: IADR/AADR/CADR General Session
Meeting: 2004 IADR/AADR/CADR General Session (Honolulu, Hawaii)
Location: Honolulu, Hawaii
Year: 2004
Final Presentation ID: 1625

Authors
- Gettleman Lawrence (University of Louisville, Louisville, KY, USA)
- Kiat-amnuay Sudarat (University of Texas - Houston/Health Science Center, Houston, TX, USA)
- Johnston Dennis A. (Baylor University, Waco, TX, USA)
- Anderson James D. (University of Toronto, Toronto, ON, Canada)
- Jacob Rhonda F. (University of Texas - Houston/Health Science Center, Houston, TX, USA)

Gettleman Lawrence (University of Louisville, Louisville, KY, USA)
Kiat-amnuay Sudarat (University of Texas - Houston/Health Science Center, Houston, TX, USA)
Johnston Dennis A. (Baylor University, Waco, TX, USA)
Anderson James D. (University of Toronto, Toronto, ON, Canada)
Jacob Rhonda F. (University of Texas - Houston/Health Science Center, Houston, TX, USA)

SESSION INFORMATION
Oral Session
Removable/Maxillofacial Prosthodontics and Articulators
03/12/2004

Design of Multicenter Phase III Clinical Trial for Maxillofacial Prosthetics

Int J Prosthodont. 2010 May-Jun;23(3):263-70.

Clinical trial of chlorinated polyethylene for facial prosthetics.

Kiat-amnuay S[1], Jacob RF, Chambers MS, Anderson JD, Sheppard RA, Johnston DA, Haugh GS, Gettleman L.

⊕ Author information

Abstract

PURPOSE: Extraoral maxillofacial prostheses have been fabricated with silicone elastomer for 50 years with few improvements. The objective of this controlled, randomized, prospective, double-blind, single-crossover, multicenter, phase III clinical trial was to determine the noninferiority of chlorinated polyethylene elastomer (CPE) to silicone elastomer for fabricating prostheses.

MATERIALS AND METHODS: Forty-two patients were randomly assigned to wear a custom-made prosthesis fabricated from both materials for 4 months and asked to rate their satisfaction (0 = not satisfied, 10 = completely satisfied). Many other measures of prosthesis performance were investigated (see online appendices).

RESULTS: Of the 28 patients who completed the study, 68% had used silicone prostheses previously. Overall, patients rated the silicone prosthesis higher than CPE (difference: 2.2, 95% confidence interval [CI]: 0.9 to 3.6, P = .017). Previous users had a stronger preference for silicone (difference: 3.3, 95% CI: 1.7 to 4.9, P = .001), while the 9 new users rated the two materials similarly (difference: 0.0, 95% CI: -2.1 to 2.1, P = 1.00).

CONCLUSIONS: The noninferiority of CPE could not be established because of the early termination of the trial. Previous users of silicone prostheses preferred those made of silicone. However, new users expressed no preference between prostheses fabricated with the low-cost CPE or silicone. The authors have developed original clinical trial methodology for assessing extraoral maxillofacial prostheses.

TRIAL REGISTRATION: ClinicalTrials.gov NCT00123097.

PMID: 20552094

Evaluation of Two Packable Composites at 18 months

Evaluation of Two Packable Composites at 18 months

Objective: Packable Composites have been proposed as amalgam alternatives for posterior restorations. A double blind, randomized clinical trial is ongoing to compare two packable composites; with and without a flowable composite liner. The hypothesis that liners prevent marginal staining was tested at the 5% significance level. This report includes 18-month results for margin staining and observations in five other performance categories. Methods: 52 subjects needing moderate to large Class II or complex Class I restorations were selected. After informed consent was obtained, 26 Alert Restorations (Jeneric/Pentron, Wallingford, CT), with (Aw) and without (Aw/o) surface sealant; and 26 SureFil Restorations (Dentsply Caulk, Milford, DE), with (Sw) and without (Sw/o) surface sealant were placed (n=13 for all groups). Only groups Aw & Aw/o included a flowable composite (manufacturer's recommendation). For all restorations, each increment placed was < 4.0 mm thick, and cured for 40 s (QTH light). Two independent, calibrated investigators evaluated each subject using Ryge Criteria in a forced consensus model. Results: Eight restorations were unavailable for recall evaluation. Of the remaining 44, only two were rated Charlie in one category each- secondary caries (Sw), and proximal contact (Aw/o). The rest were rated Alpha or Bravo in every category. For Marginal Staining, there was no significant difference between materials (Chi Square; p = 0.84), or for sealed versus non-sealed restorations (Chi square; p = 0.64). In the other evaluation categories; for groups Aw/o, Aw, Sw/o, & Sw respectively, the Alpha % were: Color Match- 55%, 60%, 70%, 54%; Margin Continuity- 91%, 100%, 90%, 92%; Surface Smoothness- 82%, 50%, 90%, 100%; Proximal Contacts- 91%, 92%, 100%, 91%, Secondary Caries- 100%, 100%, 100%, 92 %. Conclusion: Within the limits of this trial, restorations placed with a flowable composite liner did not have significantly less marginal staining than those without one.

Division: IADR/AADR/CADR General Session
Meeting: 2004 IADR/AADR/CADR General Session (Honolulu, Hawaii)
Location: Honolulu, Hawaii
Year: 2004
Final Presentation ID: 548

Authors

- Frazier, Kevin B. (Medical College of Georgia, School of Dentistry, Augusta, GA, USA)
- Browning, Wd (Medical College of Georgia, School of Dentistry, Augusta, GA, USA)
- Callan, Rs (Medical College of Georgia, School of Dentistry, Augusta, GA, USA)
- Blalock, Js (Medical College of Georgia, School of Dentistry, Augusta, GA, USA)
- Brackett, Mg (Medical College of Georgia, School of Dentistry, Augusta, GA, USA)

Frazier, Kevin B. (Medical College of Georgia, School of Dentistry, Augusta, GA, USA)
Browning, Wd (Medical College of Georgia, School of Dentistry, Augusta, GA, USA)
Callan, Rs (Medical College of Georgia, School of Dentistry, Augusta, GA, USA)
Blalock, Js (Medical College of Georgia, School of Dentistry, Augusta, GA, USA)
Brackett, Mg (Medical College of Georgia, School of Dentistry, Augusta, GA, USA)

SESSION INFORMATION
Poster Session
Adhesives, Composites, Sealants
03/11/2004

Evaluation of Two Packable Composites at 18 months

Article Citation:
W. W. Brackett, W. D. Browning, M. G. Brackett, R. S. Callan, and J. S. Blalock (*2007*) Effect of Restoration Size on the Clinical Performance of Posterior "Packable" Resin Composites Over 18 Months. Operative Dentistry: June 2007, Vol. 32, No. 3, pp. 212-216.

https://doi.org/10.2341/06-87

CLINICAL RESEARCH

Effect of Restoration Size on the Clinical Performance of Posterior "Packable" Resin Composites Over 18 Months

W. W. Brackett*, W. D. Browning, M. G. Brackett, R. S. Callan, and J. S. Blalock

William W Brackett, DDS, MSD, associate professor, Department of Oral Rehabilitation, School of Dentistry, Medical College of Georg[illegible]

Williar[illegible]
Medic[illegible]

Marth[illegible]
GA, U[illegible]

Rick S[illegible]
GA, U[illegible]

John [illegible]
GA, U[illegible]

*Repr[illegible]

SUMMARY

Fifty predominantly moderate or large Class II or multiple-surface Class I resin composite restorations were placed in molars under rubber dam isolation. The restorative systems used were: Alert Condensable (Jeneric/Pentron) and SureFil (Dentsply/Caulk). The restorations were classified according to size, with 7 small, 25 moderate and 18 large, of which 8 were cusp replacement restorations. Baseline, 6, 12 and 18-month double-blinded clinical evaluations were carried out using modified USPHS criteria. The independent variables: restorative material, restoration size and three other clinical factors, were tested using a Multiple Logistic Regression procedure to determine if any were predictive of failure. Of the 50 restorations, four failed by the 18-month recall, three failed due to fracture of the restoration and one due to secondary caries. Both restorative systems demonstrated a 92% success rate. No association between restoration size (p=0.99) or restorative material (p=0.65) and failure was found. Similarly, the additional variables, occlusal contact type, presence of occlusal wear facets and first or second molar, were not predictive of failure.

Five-year Clinical Evaluation of Two Adhesives in Non-carious Cervical Lesions

Objectives: This randomized controlled clinical trial evaluated the 5-year clinical performance of a self-etching primer system with selective acid-etching of enamel and a single-bottle adhesive system. Methods: Seventy-two non-carious cervical lesions in 8 patients (4 men and 4 women) with a mean age of 61.3 years (range 45-78) were involved for the study. Enamel bevel was placed and dentin walls were lightly ground, and restored with Clearfil Liner Bond II (LB) or Single Bond (SB) in conjunction with a hybrid resin composite (Clearfil AP-X). In the case of LB, enamel wall was pretreated with 37% phosphoric acid for a few seconds. Each patient received both restorative groups randomly. All restorations (37 restorations for LB and 35 restorations for SB) were placed by one dentist. The restorations were evaluated double blind after 5 years using modified USPHS criteria. The data were statistically analyzed using the Chi-square test. Results: All but one restoration that was replaced by a crown after the 2-year recall visit were evaluated after 5 years. 100% retention rates were recorded for both restorative groups. No recurrent caries was detected on any restorations. The only minor problem was marginal discoloration. Superficial and localized marginal discoloration occurred 18% of the restorations, and mainly at the dentin margin of the restoration. There were no significant differences in the marginal integrity between LB and SB. Conclusions: Restorative techniques used in this study demonstrated a good clinical effectiveness in the restoration of non-carious cervical lesions. Supported by Grant-in-Aid for Scientific Research from JSPS, Japan (A 09307046, A 12307043, A 12307045, C 10671793 and C 13672004).

Division: IADR/AADR/CADR General Session
Meeting: 2004 IADR/AADR/CADR General Session (Honolulu, Hawaii)
Location: Honolulu, Hawaii
Year: 2004
Final Presentation ID: 539

Kubo, Shisei (Nagasaki University Graduate School of Biomedical Sciences, Nagasaki, N/A, Japan)
Yokota, Hiroaki (Nagasaki University Graduate School of Biomedical Sciences, Nagasaki, N/A, Japan)
Kawasaki, Koji (Nagasaki University Hospital, Nagasaki, N/A, Japan)
Hayashi, Yoshihiko (Nagasaki University Graduate School of Biomedical Sciences, Nagasaki, N/A, Japan)

Authors

- Kubo, Shisei (Nagasaki University Graduate School of Biomedical Sciences, Nagasaki, N/A, Japan)
- Yokota, Hiroaki (Nagasaki University Graduate School of Biomedical Sciences, Nagasaki, N/A, Japan)
- Kawasaki, Koji (Nagasaki University Hospital, Nagasaki, N/A, Japan)
- Hayashi, Yoshihiko (Nagasaki University Graduate School of Biomedical Sciences, Nagasaki, N/A, Japan)

SESSION INFORMATION
Poster Session
Adhesives, Composites, Sealants
03/11/2004

Five-year Clinical Evaluation of Two Adhesives in Non-carious Cervical Lesions

J Dent. 2006 Feb;34(2):97-105. Epub 2005 Jun 22.

Five-year clinical evaluation of two adhesive systems in non-carious cervical lesions.

Kubo S[1], Kawasaki K, Yokota H, Hayashi Y.

Author information

1 Division of Cariology, Department of Developmental and Reconstructive Medicine, Course of Medical and Dental Sciences, Nagasaki University Graduate School of Biomedical Sciences, 1-7-1, Sakamoto, Nagasaki 852-8588, Japan.

Abstract

OBJECTIVE: This controlled clinical trial evaluated the 5-year clinical performance of a self-etching primer system including selective enamel-etching with phosphoric acid and a one-bottle adhesive system.

METHODS: Seventy-two non-carious cervical lesions in 8 patients (4 male and 4 female) with a mean age of 61.3 years (range 45-78) participated in the study. An enamel bevel was placed and dentin lightly ground, and cavities restored with clearfil liner bond II (LB) or single bond (SB) in conjunction with a hybrid resin composite (Clearfil AP-X). In the case of 27 cavities for LB, the enamel was pretreated with 37% phosphoric acid for 10 s. Each patient received both types of restoration, which were distributed on a random basis. All restorations (37 restorations for LB and 35 restorations for SB) were placed by one dentist. The restorations were evaluated blind after 5 years using modified USPHS criteria. The data were statistically analyzed using the Fisher's exact test.

RESULTS: All but one restoration (which was replaced by a crown after the 2-year recall) were evaluated after 5 years. 100% retention rates were recorded for both restorative groups. No caries was detected in association with any restorations. The only minor problem was marginal discoloration; superficial and localized marginal discoloration occurred around 18% of the restorations, and mainly at the dentin margin. There were no significant differences in the marginal integrity between the LB and SB groups.

CONCLUSIONS: Restorative materials used in this study demonstrated a good clinical effectiveness in the restoration of non-carious cervical lesions for 5 years.

PMID: 15978714 DOI: 10.1016/j.jdent.2005.04.003

Intramuscular Injection of Granisetron in TMD Patients and Healthy Subjects

Intramuscular Injection of Granisetron in TMD Patients and Healthy Subjects

Objectives: Serotonin (5-HT) is present in the human masseter muscle and seems to mediate pain and allodynia/hyperalgesia by the 5-HT_3 receptor. The level of 5-HT in the masseter muscle was accordingly reported to be increased in patients with chronic myalgia, and associated with pain and allodynia/hyperalgesia. The aim of this study was to investigate if local administration of the 5-HT_3 antagonist granisetron (Kytril®) influences the pain level and pressure pain threshold (PPT) of the masseter muscle in patients with temporomandibular disorders (TMD) of muscular origin and healthy subjects. **Methods**: Eighteen patients with TMD including bilateral localized myalgia of the masseter muscle and 24 healthy subjects participated in the study. The patients assessed the degree of pain in the masseter region for each side separately on a visual analogue scale. The most tender point of the masseter muscle on both sides was chosen for injection of test substances in the patients, while standardized points in the center of the muscles were chosen for the healthy subjects. PPT was assessed over these points before and after intramuscular injection of granisetron on one side and isotonic saline on the other side. This was performed in a randomized and double blind order. **Results**: The intensity of pain from the masseter muscle decreased after injection of granisetron, but increased after saline. The difference between substances was significant ($P < 0.05$, Wilcoxon). Granisetron increased the PPT in both patients and healthy subjects, while there were no changes after saline. The difference between substances was significant in both groups ($P < 0.05$, dependent t-test). **Conclusion**: The results of this study indicate that local administration of the 5-HT_3 antagonist granisetron reduces pain and allodynia/hyperalgesia of the masseter muscle in patients with TMD of muscular origin and increases the pressure pain threshold in healthy subjects.

Division: IADR/AADR/CADR General Session
Meeting: 2004 IADR/AADR/CADR General Session (Honolulu, Hawaii)
Location: Honolulu, Hawaii
Year: 2004
Final Presentation ID: 1596

Authors

- Christidis Nikolaos (Karolinska Institutet, Huddinge, N/A, Sweden)
- Nilsson Anna (Karolinska Institutet, Stockholm, N/A, Sweden)
- Kopp Sigvard (Karolinska Institutet, Huddinge, N/A, Sweden)
- Ernberg Malin (Karolinska Institutet, Huddinge, N/A, Sweden)

Christidis Nikolaos (Karolinska Institutet, Huddinge, N/A, Sweden)
Nilsson Anna (Karolinska Institutet, Stockholm, N/A, Sweden)
Kopp Sigvard (Karolinska Institutet, Huddinge, N/A, Sweden)
Ernberg Malin (Karolinska Institutet, Huddinge, N/A, Sweden)

SESSION INFORMATION
Oral Session
Experimental Muscle Pain
03/12/2004

Intramuscular Injection of Granisetron in TMD Patients and Healthy Subjects

Clin J Pain. 2007 Jul-Aug;23(6):467-72.

Intramuscular injection of granisetron into the masseter muscle increases the pressure pain threshold in healthy participants and patients with localized myalgia.

Christidis N[1], Nilsson A, Kopp S, Ernberg M.

Author information

Abstract

OBJECTIVES: The aims of this study were to experimentally investigate whether an intramuscular injection of the 5-HT(3) antagonist granisetron into the masseter muscle increases the mechanical pain threshold in healthy participants and reduces masseter muscle pain or allodynia in patients with craniofacial myalgia.

METHODS: Eighteen patients with bilateral localized myalgia of the masseter muscle and 24 healthy participants participated in this randomized, double-blind study, in which granisetron was injected on one side and isotonic saline on the other side. Pain (Visual Analog Scale) and pressure pain threshold (PPT) were recorded before and during 30 minutes after injections and the changes from baseline were analyzed with analysis of variance.

RESULTS: In both groups, the PPT increased after injection of granisetron whereas it decreased after saline. The difference between substances was significant (patients: $P=0.016$; healthy participants: $P=0.029$). In the healthy participants there was also a significant time effect ($P<0.001$) and an interaction between time and substance ($P=0.022$). The post-hoc test showed that the difference between substances was significant 0 to 15 minutes after injections (Bonferroni t test; $P<0.05$). The pain intensity from the masseter muscle did not differ between substances, but there was a significant time effect ($P<0.001$) and an interaction between time and substance ($P<0.001$). The post-hoc test showed significantly lower pain intensity on the granisetron side 0 to 2 minutes after injections (Bonferroni t test; $P<0.05$).

CONCLUSIONS: This study indicates that intramuscular injection of granisetron into the masseter muscle increases the PPT in healthy participants and in patients with craniofacial myalgia.

PMID: 17575485 DOI: 10.1097/AJP.0b013e318058abb1

Patients preference of major connector design of removable partial dentures

Patientsf preference of major connector design of removable partial dentures

Objectives: Ti-6Al-7Nb alloy has many advantageous properties over Co-Cr alloy and, therefore, has a potential to be applied to removable partial dentures (RPD). However, since its elastic modulus is approximately half of Co-Cr, the improvement of rigidity by reinforcement design is necessary for the application to major connectors (MC). Our preliminarily study showed 4 potential strengthening designs for Ti-6Al-7Nb MC, which had comparable rigidity with a conventional Co-Cr MC (0.8mm thick x 10mm width). Those included; A (0.8mm x 16mm); B (1mm x 10mm); C and D (0.8mm x 10mm, with reinforcements at the middle and at the anterior and posterior borders). The aim of this study was to examine if there is patients' preference of specific MC design.
Methods: Seven patients, (1 male and 6 females; 61.9 +/- 9.5 years) with Kennedy class I or II maxillary RPD participated in this study. Four trial major connectors (TMC) were fabricated with light cure tray resin and inlay wax for each subject. The subjects were given 2 TMC to wear, each one for 30 seconds and, were asked to select the preferred one. This was repeated 6 times in a random order and every combination was tested. The construction of the TMC and the administration of the test were performed following standardized protocols.

Results: The preference test took approximately 25-30 minutes in the clinical set up. The rank of the patients' preference was significantly different among 4 TMC (Friedman test, $P<0.05$). Designs A, C and D were preferred to design B (Bonferroni post-hoc, $P<0.05$ for each comparison).

Conclusions: The patients' preference of the MC design could be tested by this method in the clinical setup without any substantial difficulty. Thinner designs, although wider or with reinforcements, tended to be preferred to thicker design in our study subjects.

Division: IADR/AADR/CADR General Session
Meeting: 2004 IADR/AADR/CADR General Session (Honolulu, Hawaii)
Location: Honolulu, Hawaii
Year: 2004
Final Presentation ID: 418

Authors

- Pallegama Ranjith (Tokyo Medical & Dental University, Tokyo, N/A, Japan)
- Aridome Kumiko (Tokyo Medical & Dental University, Tokyo, N/A, Japan)
- Baba Kazuyoshi (Tokyo Medical & Dental University, Tokyo, N/A, Japan)
- Ohyama Takashi (Tokyo Medical & Dental University, Tokyo, N/A, Japan)

SESSION INFORMATION
Poster Session
Clinical Outcomes of Prosthodontic Treatment
03/11/2004

Pallegama Ranjith (Tokyo Medical & Dental University, Tokyo, N/A, Japan)
Aridome Kumiko (Tokyo Medical & Dental University, Tokyo, N/A, Japan)
Baba Kazuyoshi (Tokyo Medical & Dental University, Tokyo, N/A, Japan)
Ohyama Takashi (Tokyo Medical & Dental University, Tokyo, N/A, Japan)

Patients preference of major connector design of removable partial dentures

J Med Dent Sci. 2005 Jun;52(2):123-8.

Patients' preference for acrylic resin major connector analogues formulated for titanium alloy removable partial dentures.

Pallegama RW[1], Aridome K, Baba K, Ohyama T.

Author information

Abstract

PURPOSE: Aim of this study was to determine patients' preference to acrylic resin major connector analogues (MCA) that simulated strengthened major connector designs formulated for Ti-6Al-7Nb alloy.

MATERIALS AND METHODS: Four MCA namely wide design (Wide), design with 2 strengthening ridges (2SR), design with 1 strengthening ridge (1SR), and thick design (Thick) were fabricated using light-polymerizing acrylic resin for 10 patients with Kennedy Class I or II partially edentulous maxillary arches. They were asked to wear each MCA in the mouth for 30 seconds in 6 pairs, and to report their preference for each pair. Using these data the 4 MCA were ranked in a descending preference order for each patient. A within-subject comparison of preferences was performed with the Friedman test and multiple comparisons with Wilcoxon Signed Ranks test.

RESULTS: A statistically significant preference order was revealed: Wide, 1SR, 2SR, and Thick ($P < 0.008$). The wide design ($P < 0.004$) and the 1SR ($P < 0.01$) were significantly preferred to the thick design. However, individual data showed that the first preference varied depending upon the subject.

CONCLUSION: Thinner designs tended to be preferred to the thicker design by the subjects, while none of the designs tested were consistently selected as the best design.

PMID: 16187618

Peri-implant outcome of 2-implant mandibular overdentures: a 10-year randomized study

Peri-implant outcome of 2-implant mandibular overdentures: a 10-year randomized study

Objectives: long-term data on peri-implant tissue response to splinted versus unsplinted implants retaining mandibular overdentures (OD) remains scarce. Aim of the study was to evaluate the efficiency of two, splinted and unsplinted, implants in the OD therapy over a 10-year period.
Methods: 36 full edentulous patients (mean age: 63.7) were selected. Two implants (Brånemark System, Nobelbiocare, Sweden) were installed at the interforaminal area and connected 3 to 5 m. later with standard abutments. The 36 patients were randomly allocated in one of the 3 overdenture groups with different attachment systems: Magnets or Balls (test) or Bars (control). Patients were followed for 4, 12, 60 and 120 m. post-abutment connection. Group means (PROC MIXED) as well as linear regression (PROC REG) were fitted with attachment type and time as classification variables and corrected for simultaneous testing (Tukey). The overall p-value (a) was set 0.05.

Results: after 10 years, 9 patients had died and one was severely ill. None of the implants did fail. Mean plaque and bleeding index (BI), change in attachment level (AL), Periotest values and marginal bone level (MBL) at year 10 were not significant different between the groups. The implant stability quotient (ISQ/Osstell device) rated significantly higher for the Ball group (p=0.04) compared to the others. No correlation was found between BI and MBL.

Conclusions: the fact that no implants failed and that the marginal bone loss was limited to 0.05 mm/year, excluding the first months of remodelling, offers the implants in the 2-implant mandibular overdenture-concept an excellent prognosis, irrespective of the attachment system used.

Acknowledgements: Nobelbiocare, Sweden and Dyna, The Netherlands both are thanked for providing the ball and magnet attachments respectively.

Division: IADR/AADR/CADR General Session
Meeting: 2004 IADR/AADR/CADR General Session (Honolulu, Hawaii)
Location: Honolulu, Hawaii
Year: 2004
Final Presentation ID: 2708

Authors

- Naert Ignace (Catholic University of Leuven, Leuven, N/A, Belgium)
- Alsaadi Ghada (Catholic University Leuven, Leuven, N/A, Belgium)
- Coucke Wim (Independent statistician, Heverlee, N/A, Belgium)
- Van Steenberghe Daniel (Catholic University of Leuven, Leuven, N/A, Belgium)
- Quirynen Marc (Catholic University of Leuven, Leuven, N/A, Belgium)

Naert Ignace (Catholic University of Leuven, Leuven, N/A, Belgium)
Alsaadi Ghada (Catholic University Leuven, Leuven, N/A, Belgium)
Coucke Wim (Independent statistician, Heverlee, N/A, Belgium)
Van Steenberghe Daniel (Catholic University of Leuven, Leuven, N/A, Belgium)
Quirynen Marc (Catholic University of Leuven, Leuven, N/A, Belgium)

SESSION INFORMATION
Oral Session
Human Clinical Implant Investigations
03/12/2004

Peri-implant outcome of 2-implant mandibular overdentures: a 10-year randomized study

Int J Oral Maxillofac Implants. 2004 Sep-Oct;19(5):695-702.

A 10-year randomized clinical trial on the influence of splinted and unsplinted oral implants retaining mandibular overdentures: peri-implant outcome.

Naert I[1], Alsaadi G, van Steenberghe D, Quirynen M.

Author information

Abstract

PURPOSE: This randomized controlled clinical trial aimed to evaluate the efficacy of splinted implants versus unsplinted implants in overdenture therapy over a 10-year period.

MATERIALS AND METHODS: The study sample comprised 36 completely edentulous patients, 17 men and 19 women (mean age 63.7 years). In each patient, 2 implants (Brånemark System, Nobel Biocare, Göteborg, Sweden) were placed in the interforaminal area. Three to 5 months after placement, they were connected to standard abutments. The patients were then rehabilitated with ball-retained overdentures, magnet-retained overdentures, or bar-retained overdentures (the control group). Patients were followed for 4, 12, 60, and 120 months post-abutment connection. Group means as well as linear regression models were fitted with attachment type and time as classification variables and corrected for simultaneous testing (Tukey).

RESULTS: After 10 years, 9 patients had died and 1 was severely ill. Over 10 years, no implants failed. Mean Plaque Index, Bleeding Index, change in attachment level, Periotest values, and marginal bone level at the end of the follow-up period were not significantly different among the groups.

DISCUSSION: The annual marginal bone loss, excluding the first months of remodeling, was comparable with that found around healthy natural teeth.

CONCLUSION: The fact that no implants failed and that overall marginal bone loss after the first year of bone remodeling was limited suggested that implants in a 2-implant mandibular overdenture concept have an excellent prognosis in this patient population, irrespective of the attachment system used.

PMID: 15508985

Postoperative Sensitivity: Comparison of Two Dentin Bonding Agents

Self-etching, self-priming dentin bonding agents are reputed to reduce postoperative sensitivity. Objectives: This research compares sensitivity in two groups of people. Methods: The study is a community-based, double-blind clinical trial. To date 52 people have given written informed consent, and completed treatment. Teeth were restored due to caries, restoration failure and, predominately, to satisfy patients' esthetic preferences. Teeth were asymptomatic. Assignment to treatment group (TX) or control group (CN) was random. Restorations were fabricated using Z250 and SingleBond (CN) or AdperPrompt (TX). Restorations were placed according to manufacturer's instruction. Using a 100mm Visual Analog Scale (VAS), pain level was recorded immediately following a standardized cold water stimulus. Evaluations were pre-operatively, and at one and 13 weeks. The experimental unit was the person. Participants received two, three or four restorations. People with two or four restorations received an equal number of TX and CN restorations. For those with three restorations the unpaired restoration was randomly assigned to TX or CN. The final outcome was an average of the VAS scores. Results: 69 TX restorations were placed, 13 Class 1 and 56 Class 2. For CN it was 66, 11 and 55, Class 1s & 2s respectively. For the pre-op, 1-wk and 13-wk evaluations respectively, the mean TX score was 28.8, 22.3 & 21.0. The mean CN score was 27.0, 20.5 & 17.8. A Repeated Measures Two-Factor ANOVA was conducted. There was no significant difference between TX and CN groups at any evaluation. There was a significant difference between the three recall periods ($p < 0.001$). For both groups, compared to the pre-op evaluation, the mean VAS scores at the 1-week and 13-week evaluation was significantly lower. Conclusion: Both bonding agents were associated with reduced post-operative sensitivity. This project was supported by 3M Dental.

Division: IADR/AADR/CADR General Session
Meeting: 2004 IADR/AADR/CADR General Session (Honolulu, Hawaii)
Location: Honolulu, Hawaii
Year: 2004
Final Presentation ID: 536

Authors

- Browning, William D. (Medical College of Georgia, Augusta, GA, USA)
- Frazier, Kb (Medical College of Georgia, Augusta, GA, USA)
- Chan, Dcn (Medical College of Georgia, Augusta, GA, USA)
- Pohjola, Rm (Medical College of Georgia, Augusta, GA, USA)
- Myers, Ml (Medical College of Georgia, Augusta, GA, USA)

Browning, William D. (Medical College of Georgia, Augusta, GA, USA)
Frazier, Kb (Medical College of Georgia, Augusta, GA, USA)
Chan, Dcn (Medical College of Georgia, Augusta, GA, USA)
Pohjola, Rm (Medical College of Georgia, Augusta, GA, USA)
Myers, Ml (Medical College of Georgia, Augusta, GA, USA)

SESSION INFORMATION
Poster Session
Adhesives, Composites, Sealants
03/11/2004

Postoperative Sensitivity: Comparison of Two Dentin Bonding Agents

Oper Dent. 2007 Mar-Apr;32(2):112-7.

Postoperative sensitivity: a comparison of two bonding agents.

Browning WD[1], Blalock JS, Callan RS, Brackett WW, Schull GF, Davenport MB, Brackett MG.

Author information

1 Department of Oral Rehabilitation, School of Dentistry, Medical College of Georgia, Augusta, GA, USA. wbrownin@mail.mcg.edu

Abstract

Historically, postoperative pain associated with temperature was considered a thermal conduction problem. More recently, pulpal hydrodynamics has been used to explain this sensitivity. Relative to restorations placed with dentin bonding agents that require a separate etching step, agents that include an acidic primer are believed to result in a better seal of the dentinal tubules. This study compared pain associated with a standardized cold stimulus in two groups of restorations. One group was placed with a self-priming resin that required a separate etch step, the other with a self-etching, self-priming dentin bonding agent. This was a community-based, randomized, double-blind clinical trial. Two hundred and nine restorations were placed for 76 participants. All teeth were asymptomatic at the start of the trial. Immediately following application of a standardized cold stimulus, participants rated the pain for each restored tooth using a Visual Analog Scale (VAS). For each group of restorations, VAS scores at 13 weeks were compared to preoperative scores. In addition, the preoperative score was subtracted from the 13-week score, and the two groups of restorations were compared. For both groups of restorations, the median scores were significantly reduced at 13 weeks. This decrease in the VAS score reflects a reduction in sensitivity below that which existed preoperatively. There was no significant difference between the two groups of restorations in terms of change in sensitivity at 13 weeks.

PMID: 17427818 DOI: 10.2341/06-58

TMD therapy using two occlusal splint designs

TMD therapy using two occlusal splint designs

Objectives: Compare occlusal splint therapy in TMD patients using two splint designs.
Methods: In a double blind randomized parallel trial, 40 consenting patients were selected from the dental faculty pool of TMD patients. Two splint designs were made; an ordinary stabilization (Michigan type, type “a”) or a NTI (Nociceptiv trigeminal inhibition, type “b”) splint. All patients were treated by one operator. A separate examiner assessed joint and muscle tenderness by palpation and bite opening prior to splint therapy, and after 2 and 6 weeks and 3 months splint use. Additionally, the patients reported on a VAS scale headache and TMD-related pain and comfort of splint use. Commentaries were also invited. The differences in splint design were not described to the patients and their allocated splint design was unknown to the examiner.

Results: Eighteen patients have been observed over 3 months and form the basis of these preliminary findings. Muscle tenderness and self-reported pain was less with the type a splint ($p < .05$, Wilcoxon sign rank test), but not for the type b splint. A reduction of headache was reported for the type b splint ($p = .04$), but not for the type a splint. There were no changes for joint pain and bite opening.

Few differences between the two splints have been noted. The patients with a type a splint reported less pain at 14 days ($p = .03$, Mann-Whitney U test), headache at 6 weeks ($p = .02$) and had less tender muscles at 3 months ($p = .009$). Comfort of use was identical for both splint designs.

Conclusion: The preliminary observations indicate that splint treatment using the Michigan type and the NTI splint types were comparable over 3 months.

Division: IADR/AADR/CADR General Session
Meeting: 2004 IADR/AADR/CADR General Session (Honolulu, Hawaii)
Location: Honolulu, Hawaii
Year: 2004
Final Presentation ID: 1192

Authors

- Mo Arild (University of Oslo, Oslo, N/A, Norway)
- Jokstad Asbjorn (University of Oslo, Oslo, N/A, Norway)
- Schie Krogstad Berit (University of Oslo, Oslo, N/A, Norway)
- Dahl Bjorn L (University of Oslo, Oslo, N/A, Norway)

SESSION INFORMATION
Poster Session
Orofacial Pain - Diagnosis and Treatment
03/11/2004

Mo Arild (University of Oslo, Oslo, N/A, Norway)
Jokstad Asbjorn (University of Oslo, Oslo, N/A, Norway)
Schie Krogstad Berit (University of Oslo, Oslo, N/A, Norway)
Dahl Bjorn L (University of Oslo, Oslo, N/A, Norway)

TMD therapy using two occlusal splint designs

Acta Odontol Scand. 2005 Aug;63(4):218-26.

Clinical comparison between two different splint designs for temporomandibular disorder therapy.

Jokstad A[1], Mo A, Krogstad BS.

Author information

Abstract

OBJECTIVE: To compare splint therapy in temporomandibular disorder (TMD) patients using two splint designs.

MATERIAL AND METHODS: In a double-blind randomized parallel trial, 40 consenting patients were selected from the dental faculty pool of TMD patients. Two splint designs were produced: an ordinary stabilization (Michigan type) and a NTI (Nociceptiv trigeminal inhibition). The differences in splint design were not described to the patients. All patients were treated by one operator. A separate, blinded, examiner assessed joint and muscle tenderness by palpation and jaw opening prior to splint therapy, and after 2 and 6 weeks' and 3 months' splint use during night-time. The patients reported headache and TMD-related pain on a visual analog scale before and after splint use, and were asked to describe the comfort of the splint and invited to comment.

RESULTS: Thirty-eight patients with mainly myogenic problems were observed over 3 months. A reduction of muscle tenderness upon palpation and self-reported TMD-related pain and headache and an improved jaw opening was seen in both splint groups ($p < 0.05$; paired t-test and Wilcoxon signed-ranks tests). There were no changes for TM joint tenderness upon palpation. No differences were noted between the two splint designs after 3 months for the chosen criteria of treatment efficacy ($p > 0.05$; Mann-Whitney U-test).

CONCLUSION: No differences in treatment efficacy were noted between the Michigan and the NTI splint types when compared over 3 months.

PMID: 16040444 DOI: 10.1080/00016350510019982

Treatment of Peri-implantitis with an Er:YAG Laser

Treatment of Peri-implantitis with an Er:YAG Laser

Objectives:The aim of this controlled, parallel design clinical study was to compare the effectiveness of an Er:YAG laser to that of mechanical debridement using plastic curets and antiseptic therapy for nonsurgical treatment of peri-implantitis; Methods: Twenty patients with moderate to advanced peri-implantitis lesions were randomly treated with either (1) an Er:YAG laser (KEY3, KaVo, Germany) using a cone-shaped glass fiber tip at an energy setting of 100 mJ/pulse and 10 pps (ERL), or (2) mechanical debridement using plastic curets and antiseptic therapy with chlorhexidine digluconate (0.2 %) (C). The following clinical parameters were measured at baseline, 3 and 6 months after treatment by one blinded and calibrated examiner: Plaque index (PI), bleeding on probing (BOP), probing depth (PD), gingival recession (GR) and clinical attachment level (CAL). Results: At the baseline examination, there were no statistically significant differences in any of the investigated parameters. Mean value of BOP decreased in the ERL group from 83% at baseline to 31% after 6 months ($P<0.001$) and in the C group from 80% at baseline to 58% after 6 months ($P<0.001$). The difference between both groups was statistically significant ($P<0.05$). The sites treated with ERL demonstrated a mean CAL change from 5.8±1.0 mm at baseline to 5.1±1.1 mm ($P<0.01$) after 6 months. The C sites demonstrated a mean CAL change from 6.2±1.5 mm at baseline to 5.6±1.6 mm ($P<0.001$) after 6 months. After 6 months, the difference between both groups was statistically non significant ($P>0.05$). Conclusion: Within the limits of the present study, it was concluded that (i) at six months following treatment both therapies led to significant improvements of the investigated clinical parameters, and (ii) ERL resulted in a statistically significant higher reduction of BOP than C.

Division: IADR/AADR/CADR General Session
Meeting: 2004 IADR/AADR/CADR General Session (Honolulu, Hawaii)
Location: Honolulu, Hawaii
Year: 2004
Final Presentation ID: 1571

Authors

- Schwarz Frank (Heinrich Heine University, Düsseldorf, N/A, Germany)
- Rothamel Daniel (Heinrich Heine University, Düsseldorf, N/A, Germany)
- Sculean Anton (Johannes Gutenberg-University, Mainz, N/A, Germany)
- Latz Tilo (Heinrich Heine University, Düsseldorf, N/A, Germany)
- Schwenzer Katja (University Basel, Basel, N/A, Switzerland)
- Becker Jürgen (Heinrich Heine University, Düsseldorf, N/A, Germany)

SESSION INFORMATION
Oral Session
Guided Tissue Regeneration
03/12/2004

Schwarz Frank (Heinrich Heine University, Düsseldorf, N/A, Germany)
Rothamel Daniel (Heinrich Heine University, Düsseldorf, N/A, Germany)
Sculean Anton (Johannes Gutenberg-University, Mainz, N/A, Germany)
Latz Tilo (Heinrich Heine University, Düsseldorf, N/A, Germany)
Schwenzer Katja (University Basel, Basel, N/A, Switzerland)
Becker Jürgen (Heinrich Heine University, Düsseldorf, N/A, Germany)

Treatment of Peri-implantitis with an Er:YAG Laser

Clin Oral Implants Res. 2005 Feb;16(1):44-52.

Clinical evaluation of an Er:YAG laser for nonsurgical treatment of peri-implantitis: a pilot study.

Schwarz F[1], Sculean A, Rothamel D, Schwenzer K, Georg T, Becker J.

Author information

Abstract

The aim of this controlled, parallel design clinical study was to compare the effectiveness of an Er:YAG laser (ERL) to that of mechanical debridement using plastic curettes and antiseptic therapy for nonsurgical treatment of peri-implantitis. Twenty patients with moderate to advanced peri-implantitis lesions were randomly treated with either (1) an ERL using a cone-shaped glass fiber tip at an energy setting of 100 mJ/pulse and 10 pps (ERL), or (2) mechanical debridement using plastic curettes and antiseptic therapy with chlorhexidine digluconate (0.2%) (C). The following clinical parameters were measured at baseline, 3 and 6 months after treatment by one blinded and calibrated examiner: Plaque index (PI), bleeding on probing (BOP), probing depth (PD), gingival recession (GR) and clinical attachment level (CAL). At the baseline examination, there were no statistically significant differences in any of the investigated parameters. Mean value of BOP decreased in the ERL group from 83% at baseline to 31% after 6 months ($P < 0.001$) and in the C group from 80% at baseline to 58% after 6 months ($P < 0.001$). The difference between the two groups was statistically significant ($P < 0.001$, respectively). The sites treated with ERL demonstrated a mean CAL change from 5.8 +/- 1 mm at baseline to 5.1 +/- 1.1 mm ($P < 0.01$) after 6 months. The C sites demonstrated a mean CAL change from 6.2 +/- 1.5 mm at baseline to 5.6 +/- 1.6 mm ($P < 0.001$) after 6 months. After 6 months, the difference between the two groups was statistically not significant ($P > 0.05$). Within the limits of the present study, it was concluded that (i) at 6 months following treatment both therapies led to significant improvements of the investigated clinical parameters, and (ii) ERL resulted in a statistically significant higher reduction of BOP than C.

PMID: 15642030 DOI: 10.1111/j.1600-0501.2004.01051.x

[illegible]

2005

Aftercare and Cost-Analysis with 3-types of Mandibular Implant-retained Overdentures

Aftercare and Cost-Analysis with 3-types of Mandibular Implant-retained Overdentures

Accepting that an implant-retained overdenture is treatment of choice, it is of interest which modality of implant overdenture treatment is most cost-efficient. Economic considerations may influence or even dictate that choice, but this is only reasonable if they are based on long term data.
Objectives: To determine the total direct costs for the total treatment of placing the implants and insertion of the overdenture with retentive system and, over a period of 8 years, aftercare and maintenance.

Methods: In a randomized controlled clinical trial, 1/3 of the patients received an implant-retained overdenture on two implants with ball attachments, 1/3 on two implants with a single bar and 1/3 on four implants with a triple bar. We measured direct treatment costs and costs of aftercare and maintenance.

Results: After 8,3 yrs 103 patients (94%) out of 110 patients, who were included at the start of the trial, participated in the evaluation. The initial costs are 71-79% of the total costs. The results show that there is no significant difference in time and costs in the prosthetic aftercare for the three treatment groups. There is a significant difference between the three groups for the number of checkups with a simple treatment. There is an increased demand for aftercare in the group with the ball attachments for simple readjustments like activating the matrices. Conclusion: This study shows that the total direct costs of aftercare and maintenance do not differ for the 3 treatment strategies over an evaluation period of 8,3 yrs. The initial costs make the differences and make up ¾ of the total costs. The group with the ball attachments needs the highest number of prosthodontist-patient aftercare contacts. This doesn't make a significant difference in the total time consumed by providing aftercare, but surely more patient contact moments are more demanding.

Division: IADR/AADR/CADR General Session
Meeting: 2005 IADR/AADR/CADR General Session (Baltimore, Maryland)
Location: Baltimore, Maryland
Year: 2005
Final Presentation ID: 1418

Authors

- Stoker, Geert (ACTA - Vrije Universiteit, Simonshaven, N/A, Netherlands)

Stoker, Geert (ACTA - Vrije Universiteit, Simonshaven, N/A, Netherlands)

SESSION INFORMATION
Oral Session
Keynote Address and Implant Prosthodontics and Diagnosis
03/11/2005

Aftercare and Cost-Analysis with 3-types of Mandibular Implant-retained Overdentures

Journal of Dental Research

IADR International Association for Dental Research

AADR® American Association for Dental Research

Journal Home Browse Journal Submit Paper About Subscribe

Close

PDF

An Eight-year Follow-up to a Randomized Clinical Trial of Aftercare and Cost-analysis with Three Types of Mandibular Implant-retained Overdentures

G.T. Stoker[123], D. Wismeijer[2], M.A.J. van Waas[1],

First Published March 1, 2007 | Other

https://doi.org/10.1177/154405910708600315

Abstract

Mandibular implant overdentures increase satisfaction and the quality of life of edentulous individuals. Long-term aftercare and costs may depend on the type of overdentures. One hundred and ten individuals received one of 3 types of implant-retained overdentures, randomly assigned, and were evaluated with respect to aftercare and costs. The follow-up time was 8 years, with only seven drop-outs. No significant differences (Kruskal-Wallis test) were observed for direct costs of aftercare ($p = 0.94$). The initial costs constituted 75% of the total costs and were significantly higher in the group with a bar on 4 implants, compared with the group with a bar on 2 implants and the group with ball attachments on 2 implants ($p = 0.018$). The last group needed a significantly higher number of prosthodontist-patient aftercare contacts, mostly for re-adjustment of the retentive system. It can be concluded that an overdenture with a bar on 2 implants might be the most efficient in the long term.

Keywords

randomized controlled clinical trial, edentulism, cost-analysis, aftercare, implant-retained overdentures

Candida Albicans Recolonization in Denture Stomatitis Patients After LLLT Treatment

Candida Albicans Recolonization in Denture Stomatitis Patients After LLLT Treatment

OBJECTIVES: The optimal treatment modality for denture stomatitis (DS) has not yet being found since the recurrence of the disease is quite often. Low level laser therapy (LLLT) showed significant antifungal effect in the treatment of DS. The aim of this study was to investigate the recolonization of Candida albicans on dentures and palatal mucosa by patients with DS, one week after treatment with LLLT has been completed. METHODS: Patients with clinical evidence of DS (n=70) participated in this parallel, single blind and placebo, controlled study.The subjects were randomly assigned to four treatment regimens:1)685 nm laser irradiation group (30 mW; 3.0 J/cm2; 10 minutes); 2)830 nm laser irradiation group (60 mW;3.0 J/cm2;5 minutes).A semiconductor diode laser was used in both treatment cases for five consecutive days,using continuous working mode;3)placebo group(sham irradiation of palatal mucosa and denture base);4)antimicotic group(self treatment with myconazole oral gel and antiseptic solution for the dentures).Swabs from palatal mucosa and dentures were cultured on Sabouraud's dextrose agar immediately after the treatment and at follow-up checking,one week after the last treatment in antimicotic and laser groups. Candida albicans growth was assessed using semi-quantitative methods. RESULTS:The recolonization of Candida albicans on palatal mucosa and dentures occurred in a significant number of patients from all three of the treated groups, one week after the treatment. There were no statistically significant differences between laser and the antimicotic group (χ^2=2.226;p=0.329;palate);(χ^2=2.566; p=0.463;denture).However,fungal growth in all three treatment groups was mainly unchanged or decreased at follow ups, when compared to the initial findings,also without statistically significant differences between the groups (χ^2=11.043;p=0.087; palate);(χ^2=2.144; p=0.709;denture). CONCLUSION:There were no significant differences in therapeutic effect between LLLT and antimicotic oral gel.In order to achieve long term antimicotic effect it is necessary to eliminate predisposing factors and improve hygiene habits individually.

Division: IADR/AADR/CADR General Session
Meeting: 2005 IADR/AADR/CADR General Session (Baltimore, Maryland)
Location: Baltimore, Maryland
Year: 2005
Final Presentation ID: 1619

Authors

- Jerolimov, Vjekoslav (School of Dental Medicine University of Zagreb, Zagreb, N/A, Croatia)
- Maver-biscanin, Mirela (Clinical Hospital Centre Zagreb, Zagreb, N/A, Croatia)
- Mravak-stipetic, Marinka (School of Dental Medicine University of Zagreb, Zagreb, N/A, Croatia)

Jerolimov, Vjekoslav (School of Dental Medicine University of Zagreb, Zagreb, N/A, Croatia)
Maver-biscanin, Mirela (Clinical Hospital Centre Zagreb, Zagreb, N/A, Croatia)
Mravak-stipetic, Marinka (School of Dental Medicine University of Zagreb, Zagreb, N/A, Croatia)

SESSION INFORMATION

Poster Session

Complete Denture and Overdenture Research

03/11/2005

Candida Albicans Recolonization in Denture Stomatitis Patients After LLLT Treatment

Recurrence of Candida Albicans Colonization in Denture Stomatitis Patients Treated with Low Level Laser Therapy

来自hrcak.srce.hr

收藏 引用 批量引用 报错 分享

作者 M Maverbišćanin , M Mravakstipetić , V Jerolimov , B Klaić , Žarković, Damir , ...

摘要 U radu je prikazano djelovanje terapijskog lasera na gljivicu Candidu albicans uzročnika protetskog stomatitisa.

出版源 《Acta Stomatologica Croatica》 , 2007 , 41 (3) :225-232

Abstract

An optimal treatment modality for denture stomatitis (DS) hasn't been yet found since the disease recurrs quite often. The aim of this paper is to investigate the occurrence of recolonization of the yeast Candida albicans one week after laser irradiation treatment in patients with DS. 70 patients with clinical evidence of denture stomatitis participated in this study. The subjects were randomly assigned to one of four different treatment regimens: 1) irradiation with a 685 nm wavelength laser for 10 minutes (30 mW); 2) irradiation with a 830 nm wavelength laser for 5 minutes (60 mW); 3) placebo control group: sham irradiation of patients; 4) antimycotic control group: self treatment of patients' palatal mucosa with an antifungal oral gel and the use of an antiseptic solution for their dentures. A semiconductor diode laser, BTL-2000 (BTL-2 Dravotnicka Techika, Prague, Czech Republic), was used in both treatment cases using an energy density of 3.0 J/cm2 and a continuous working mode for five consecutive days. Swabs from patients palates and dentures were taken prior to initial treatment (1st day), immediately after the treatment was finished (5th day) and at a follow-up visit (12th day). We found that recolonization of Candida albicans, one week after treatment, occurred in a significant number of patients regardless of the treatment method. In order to achieve a long-term antimycotic effect, it is necessary to eliminate all predisposing factors individually, to incorporate the use of antifungal agents into the patients' regime, and to have the patients improve their oral hygiene habits.

Clinical Success Rates for Polyvinylsiloxane and Polyether Dual-Viscosity Impressions

Clinical Success Rates for Polyvinylsiloxane and Polyether Dual-Viscosity Impressions

OBJECTIVE: Conduct a randomized, controlled, prospective clinical trial to compare success rates of polyvinylsiloxane and polyether impression systems.

METHODS: Dual-viscosity systems were a polyvinylsiloxane (Dentsply-Caulk, Aquasil Ultra Monophase/Aquasil Ultra XLV) and a polyether (3M-ESPE, Impregum Penta Soft HB/Impregum Garant Soft LB). Only the first impression of a case was evaluated by a prosthodontist with the dental laboratory quality control team. Primary outcome was impression success or failure using developed criteria. Fifty senior dental students participated where their sequence of impression systems alternated for each new case. A full-arch perforated plastic (COE-Disposable Tray) or a plastic dual-arch impression tray (Tri-Bite, Direct Dental) was used based on selection guidelines. To compare first impression success rates, a Wald test was used based on a logistic regression fitted using the method of generalized estimating equations.

RESULTS: Inter-rater agreement for detecting critical defects was 92% (finish line) and 94% (axial surface). 191 impressions were evaluated and the success rate and confidence interval for each system is given below.

Impression System	Number	First Impression Success Rate	95% CI
Polyvinylsiloxane	n=103	61%	50% – 71%
Polyether	n=88	54%	43% – 65%

Wald Chi-square test = 0.7 and P-value = 0.39. Additional regression analyses, adjusted for potential confounders, did not indicate a difference between the two systems. Dual-arch trays were used for 61% of the impressions. Most common critical defect was located on the finish line (94%). Most common operator error was "inadequate gingival deflection" (15%).

CONCLUSION: Statistically, a difference could not be shown for rates of success between these two impression systems, thus one system is not superior to the other. However a trend was evident with 7% greater impression success in the dental school environment when the polyvinylsiloxane system was used. Success rates may differ and are likely higher with experienced practitioners. Supported by Dentsply Caulk.

Division: IADR/AADR/CADR General Session
Meeting: 2005 IADR/AADR/CADR General Session (Baltimore, Maryland)
Location: Baltimore, Maryland
Year: 2005
Final Presentation ID: 362

Authors

- Johnson, Glen H. (University of Washington, Seattle, WA, USA)
- Mancl, Lloyd A. (University of Washington, Seattle, WA, USA)
- Schwedhelm, E. Ricardo (University of Washington, Seattle, WA, USA)
- Verhoef, Douglas R. (University of Washington, Seattle, WA, USA)
- Junge, Thomas (University of Washington, Seattle, WA, USA)
- Lepe, Xavier (University of Washington, Seattle, WA, USA)

Johnson, Glen H. (University of Washington, Seattle, WA, USA)
Mancl, Lloyd A. (University of Washington, Seattle, WA, USA)
Schwedhelm, E. Ricardo (University of Washington, Seattle, WA, USA)
Verhoef, Douglas R. (University of Washington, Seattle, WA, USA)
Junge, Thomas (University of Washington, Seattle, WA, USA)
Lepe, Xavier (University of Washington, Seattle, WA, USA)

SESSION INFORMATION
Oral Session
Clinical Outcomes of Prosthodontic Treatment
03/10/2005

Clinical Success Rates for Polyvinylsiloxane and Polyether Dual-Viscosity Impressions

The Journal of Prosthetic Dentistry
Volume 103, Issue 1, January 2010, Pages 13-22

Clinical trial investigating success rates for polyether and vinyl polysiloxane impressions made with full-arch and dual-arch plastic trays

Glen H. Johnson DDS, MS [a], Lloyd A. Mancl PhD [b], E. Ricardo Schwedhelm DDS, MSD [c], Douglas R. Verhoef DDS [d], Xavier Lepe DDS, MS [e]

⊞ **Show more**

https://doi.org/10.1016/S0022-3913(09)60208-0 Get rights and content

Statement of problem

Success rates for making fixed prosthodontic impressions based on material and tray selection are not known.

Purpose

The purpose of this clinical study was to compare first impression success rates for 2 types of impression material and 2 impression tray systems.

Material and methods

Dual-viscosity impressions were made with a vinyl polysiloxane (VPS) (Aquasil Ultra Monophase/Aquasil Ultra XLV) and a polyether (PE) (Impregum Penta Soft HB/Impregum Garant Soft LB) impression material. The first impression made was evaluated for success or failure using developed criteria. Fifty senior dental students participated. The type of impression material alternated for each new patient. A full-arch perforated plastic (President Tray) or a plastic dual-arch impression tray (Tri-Bite) was used based on clinical guidelines. Impression success rates were compared using logistic regression, fitted using the method of generalized estimating equations (α=.05).

Results

One hundred ninety-one impressions were evaluated, and the overall success rate was 61% for VPS and 54% for PE (*P*=.39). Additional regression analyses, adjusted for potential confounders, did not indicate a difference between the 2 systems (*P*=.35). There was little difference in success rates between the 2 materials when a full-arch tray was used (50% versus 49% success, *P*=.89), whereas a larger difference was apparent with the use of dual-arch trays (70% success with VPS versus 58% success with PE, *P*=.21). The most common critical defect was located on the preparation finish line (94%), and the most common operator error was inadequate gingival displacement (15%).

Conclusions

There was little difference in success rates between VPS and PE when full-arch impression trays were used, but there was greater success when using VPS with dual-arch trays. For single teeth, the trend favored VPS, but when more than one prepared tooth per impression was involved, the success rate was higher for PE.

Clinical Trial on Health Effects from Removal of Amalgam Restorations

Clinical Trial on Health Effects from Removal of Amalgam Restorations

Concerns over adverse effects of mercury released from amalgam restorations sometimes lead patients to request removal of their amalgam restorations. Several studies report a decrease of symptoms and improvement of subjective health after removal of amalgam restorations, but no randomized controlled trials have been published on changes of symptom intensity after removal of amalgam restorations. Objectives: The aim of the present project is to conduct a clinical trial on changes in symptom load after removal of all amalgam restorations in patients with symptoms related to amalgam. At this time baseline characteristics are presented. Methods: Patients previously referred to a specialty unit from dentists and physicians for symptoms related to amalgam restorations were included in the study. At the examination, no causal relationship between amalgam exposure and symptoms was established. Thus, the patients were not recommended removal of amalgam restorations. Patients who still had amalgam restorations at follow-up 18 months to 7 years later, and fulfilling the inclusion criteria, were randomized into treatment group (n=20; removal of all amalgam restorations and replacement with other dental restorative materials) and reference group (n= 20; no treatment). Outcome variables were data from questionnaires (including MMPI-2) and laboratory data. Results: At examination, the treatment group was similar to the reference group regarding age, gender distribution, number of amalgam surfaces and concentration of mercury in blood and urine ($p>0.15$). At baseline, both groups had similar symptom intensity regarding intraoral, orofacial and general symptoms. Results from MMPI-2 from the treatment group were in agreement with results reported for a comparable group of patients (Dalen et al 2003). Conclusion: Randomized clinical trials will have a potential to provide important information regarding changes in symptom load after removal of amalgam restorations in patients with symptoms related to amalgam.
Acknowledgement: The project is funded by the Norwegian Ministry of Health.

Division: IADR/AADR/CADR General Session
Meeting: 2005 IADR/AADR/CADR General Session (Baltimore, Maryland)
Location: Baltimore, Maryland
Year: 2005
Final Presentation ID: 1192

Authors

- Björkman, Lars (UNIFOB/University of Bergen, Bergen, N/A, Norway)
- Dalen, Knut (University of Bergen, Bergen, N/A, Norway)
- Helland, Vigdis (UNIFOB/University of Bergen, Bergen, N/A, Norway)

Björkman, Lars (UNIFOB/University of Bergen, Bergen, N/A, Norway)
Dalen, Knut (University of Bergen, Bergen, N/A, Norway)
Helland, Vigdis (UNIFOB/University of Bergen, Bergen, N/A, Norway)

SESSION INFORMATION
Poster Session
Oral Effects/Delivery/Abrasion
03/10/2005

Clinical Trial on Health Effects from Removal of Amalgam Restorations

Long term changes in health complaints after removal of amalgam restorations

来自Taylor & Francis

收藏 引用 批量引用 报错 分享

作者 L BjÃ¶Rkman , TT Sjursen , K Dalen , GB Lygre , TLL Berge , ...

摘要 Objective: Concerns over adverse effects of mercury released from dental amalgam sometimes lead patients to request removal of their amalgam restorations. Several studies report improvement of subjective health after removal of amalgam restorations, but the mechanisms are unclear. The aim of this paper is to present data on long term changes in intensity of health complaints after amalgam removal in a group of patients with health complaints self-attributed to dental amalgam. Data from the five years follow-up in a clinical trial are presented and related to potential determinants of change.

收起

出版源 《Acta Odontologica Scandinavica》 , 2017 , 75 (3) :208-219

被引量 0

Correlation of Jaw Function Assessments with TMJ Closed-Lock Pain

Correlation of Jaw Function Assessments with TMJ Closed-Lock Pain

Jaw-function assessment in persons with painful TMJ disc displacement without reduction, with limited opening (closed-lock), is performed by self-report and clinical examination. It is uncertain which method more strongly correlates with jaw-pain symptoms. Objective: To compare the correlation of self-report and clinical examination jaw-function assessment instruments, with jaw-pain symptoms, in a population of subjects diagnosed with closed-lock. Methods: 106 subjects (92 women, 8 men), confirmed by MRI to have a closed-lock TMJ diagnosis, were randomly assigned to: 1) medical management; 2) comprehensive non-surgical treatment; 3) arthroscopic surgery; or 4) disc repair/plication or discectomy surgery. At baseline, jaw function was assessed by self-report by having subjects complete the Mandibular Function Impairment Questionnaire (MFIQ). Clinical examination measurements of jaw function were determined using the Dysfunction Index (DI), a sub-index of the Craniomandibular Index (CMI), and performed by a calibrated examiner (PAL). Jaw pain levels were assessed using the Symptom Severity Index (anchored to the jaw joint, SSIJT). A correlation analysis and a partial correlation analysis (PROC CANCORR, SAS Institute) were performed to assess the independent correlation between self-report (MFIQ) and jaw pain (SSIJT) (after the effect of DI was removed), and clinical examination (DI) and jaw pain (SSIJT) (after the effect of MFIQ was removed). Results: Mean SSIJT at baseline was 0.70 ± 0.21 (range: 0.12 to 1.00). The correlation between MFIQ and SSIJT was 0.412 (p = 0.0001); the independent canonical correlation was 0.358 (p = 0.0002). The correlation between DI and SSIJT was 0.312 (p = 0.001); the independent canonical correlation, 0.219 (p = 0.025). The correlation between the MFIQ and DI was 0.291 (p = 0.003). Conclusion: Self-report jaw function assessment may be more strongly correlated with jaw pain symptoms than clinical examination measures of jaw function. This study was supported by NIH Grants #R29DEO8668, R01DE1342 and P30-DEO9737.

Division: IADR/AADR/CADR General Session
Meeting: 2005 IADR/AADR/CADR General Session (Baltimore, Maryland)
Location: Baltimore, Maryland
Year: 2005
Final Presentation ID: 1393

Authors

- Look, John O. (University of Minnesota, Minneapolis, MN, USA)
- Schiffman, Eric (University of Minnesota, Minneapolis, MN, USA)
- Lenton, Patricia A. (University of Minnesota, Minneapolis, MN, USA)
- Fricton, James R. (University of Minnesota, Minneapolis, MN, USA)

Look, John O. (University of Minnesota, Minneapolis, MN, USA)
Schiffman, Eric (University of Minnesota, Minneapolis, MN, USA)
Lenton, Patricia A. (University of Minnesota, Minneapolis, MN, USA)
Fricton, James R. (University of Minnesota, Minneapolis, MN, USA)

SESSION INFORMATION

Oral Session

TMJ Structure and Function

03/11/2005

Correlation of Jaw Function Assessments with TMJ Closed-[illegible]

Journal of Dental Research

IADR International Association for Dental Research

AADR® American Association for Dental Research

Journal Home | Browse Journal | Submit Paper | About | Subscribe | Search: [illegible]

Close

PDF

Randomized Effectiveness Study of Four Therapeutic Strategies for TMJ Closed Lock

E.L. Schiffman[1]*, J.O. Look[1], J.S. Hodges[2], more...

First Published January 1, 2007 | Other

https://doi.org/10.1177/154405910708600109

Article information

A correction has been published

Abstract

For individuals with temporomandibular joint (TMJ) disc displacement without reduction with limited mouth opening (closed lock), interventions vary from minimal treatment to surgery. In a single-blind trial, 106 individuals with TMJ closed lock were randomized among medical management, rehabilitation, arthroscopic surgery with post-operative rehabilitation, or arthroplasty with post-operative rehabilitation. Evaluations at baseline, 3, 6, 12, 18, 24, and 60 months used the Craniomandibular Index (CMI) and Symptom Severity Index (SSI) for jaw function and TMJ pain respectively. Using an intention-to-treat analysis, we observed no between-group difference at any follow-up for CMI ($p \geq 0.33$) or SSI ($p \geq 0.08$). Both outcomes showed within-group improvement ($p < 0.0001$) for all groups. The findings of this study suggest that primary treatment for individuals with TMJ closed lock should consist of medical management or rehabilitation. The use of this approach will avoid unnecessary surgical procedures.

Keywords

temporomandibular joint, closed lock, randomized clinical trial, effectiveness study

Do simplified complete denture fabrication techniques produce more unscheduled consultations?

Do simplified complete denture fabrication techniques produce more unscheduled consultations?

Objectives: To find out if simplified techniques for making conventional complete dentures result in more unscheduled consultations (UCs) than traditional techniques. Methods: A randomized controlled trial was carried out in which 119 edentulous subjects received complete dentures fabricated either by traditional (T: n=58, six fabrication and five control scheduled visits) or simplified (S: n=61, five fabrication and five control scheduled visits) techniques. The overall numbers of UCs were counted and Wilcoxon rank-sum test were carried out to test the difference between fabrication techniques. In addition, the number of subjects who needed a second try-in, occlusal recording or reline were counted, then a Chi squared test and relative risk (RR) were calculated to estimate the association of "re-do procedures" and fabrication techniques. Results: There were no significant differences in the number of the UCs between T and S Groups (T: 61visits, S: 75 visits, p=0.26). Furthermore, the number of patients requiring a 2nd try-in (9T, 6S p=0.35, RR: 0.76, 95%CI: 0.40 to 1.44), relining (3T, 6S, p=0.34, RR: 1.33, 95%CI: 0.81 to 2.19), or 2nd occlusal registration (1T, 3S, p=0.33 RR: 1.49, 95%CI: 0.82 to 2.70) did not depend on treatment. Conclusion: The use of a simplified method to produce conventional dentures does not lead to an increase in the number of re-does or unscheduled consultations.

Division: IADR/AADR/CADR General Session
Meeting: 2005 IADR/AADR/CADR General Session (Baltimore, Maryland)
Location: Baltimore, Maryland
Year: 2005
Final Presentation ID: 1359

Authors

- Kawai, Yasuhiko (Nihon University, Matsudo, Chiba, N/A, Japan)
- Murakami, Hiroshi (Nihon University, Matsudo, Chiba, N/A, Japan)
- Lund, James P. (McGill University, Montreal, QC, Canada)
- Feine, Jocelyne (McGill University, Montreal, QC, Canada)

Kawai, Yasuhiko (Nihon University, Matsudo, Chiba, N/A, Japan)
Murakami, Hiroshi (Nihon University, Matsudo, Chiba, N/A, Japan)
Lund, James P. (McGill University, Montreal, QC, Canada)
Feine, Jocelyne (McGill University, Montreal, QC, Canada)

SESSION INFORMATION
Oral Session
Clinical Research and Interventions
03/11/2005

Do simplified complete denture fabrication techniques produce more unscheduled co[illegible]

Journal of Prosthodontics
Implant, Esthetic, and Reconstructive Dentistry

Your smile. Our specialty.

Full Access

Efficient Resource Use in Simplified Complete Denture Fabrication

Yasuhiko Kawai DDS, MMedSci, PhD, Hiroshi Murakami DDS, PhD, Yoshiaki Takanashi DDS, PhD, James P. Lund BDS, PhD, Jocelyne S. Feine DDS, HDR

First published: 16 August 2010 | https://doi.org/10.1111/j.1532-849X.2010.00628.x | Cited by: 21

Correspondence
Yasuhiko Kawai, Nihon University, 2-870-1 Sakaecho-Nishi Matsudo, Chiba 2718587, Japan. E-mail: kawai.yasuhiko@nihon-u.ac.jp

Abstract

Purpose: Conventional dentures will remain the only treatment available to most edentulous people for the foreseeable future. In this study, we compared the efficiency of two methods of making complete conventional dentures—the traditional academic standard (T) and a simplified technique (S) used in private practice. We have previously shown that they produce similar levels of patient satisfaction and denture quality.

Materials and Methods: Data were gathered during a randomized controlled clinical trial of 122 subjects from initial examination until 6-month follow-up. For this report, the direct costs of providing one set of conventional complete dentures by T or S techniques were estimated. All materials used were recorded and their cost was calculated in Canadian dollars (CAN$). The costs of fabrication in an outside laboratory were added. Clinician's labor time was recorded for every procedure. Between-group comparisons for each clinical procedure were carried out with independent *t*-tests. The number of patients in each group who needed postdelivery treatment was compared with Chi-square tests. The effect of group assignment and of treatment difficulty on outcomes was analyzed with multiple regression analysis.

Results: The mean total cost of the T method was significantly greater than S (CAN$166.3; $p < 0.001$), and clinicians spent 90 minutes longer ($p < 0.001$) on clinical care. The difficulty of the case had no significant influence on outcomes.

Conclusions: The results indicate that the S method is the more cost-efficient method and that there are no negative consequences that detract from the cost savings.

Does occupational therapy improve manual performance in tooth/denture

Does occupational therapy improve manual performance in tooth/denture brushing?

Objectives: Occupational therapy focuses on the promotion of the autonomy in the execution of ADL. The aim of the present study was to investigate a new way in teaching and monitoring tooth and denture brushing activities in LTC residents by employing occupational therapy techniques and evaluate the learning effect on manual performance in the individual tooth and denture brushing movements.
Methods: 61 residents, 44 women and 17 men, with an average age of 85.7 ± 6.6 years (range 72-97 years) living in a LTC in Geneva were enrolled in a randomized controlled trial. At baseline a Mini Mental State was taken and brushing movements were assessed both for denture cleaning and tooth brushing step by step by an occupational therapist. Denture and dental plaque scores were evaluated within the context of a comprehensive clinical assessment. Based on those results both the experimental and the control group were divided into an "assisted" (IA) and an "independent" (II) sub-group. In the EG tooth brushing was only initially taught whereas in the IA additionally monitored and re-educated once a week. The CG-IA received a weekly placebo activity such as manicure by the same person.

Results: Both, the occupational therapy and the placebo activity led to a significant improvement in oral ($p<0.01$; $p<0.05$) but even more denture hygiene ($p<0.001$; $p<0.05$). The monitoring of the individual brushing movements revealed that the IA sub-groups learned best to clean their dentures ($p<0.05$). For the remaining movements the learning effect was not significant. Oral and denture hygiene was most improved in patient suffering from cognitive impairment ($p<0.001$; $p<0.05$).

Conclusions: Despite the limited improvement of motor skills and a marked placebo effect, weekly occupational therapy seems to help especially demented patients in improving their oral and denture hygiene and thus promoting their autonomy in ADL.

Division: IADR/AADR/CADR General Session
Meeting: 2005 IADR/AADR/CADR General Session (Baltimore, Maryland)
Location: Baltimore, Maryland
Year: 2005
Final Presentation ID: 3155

Authors

- Bellomo, Fleurange (University of Geneva, Geneva, N/A, Switzerland)
- Julien, Noëlle (University of Geneva, Geneva, N/A, Switzerland)
- De Preux, Fréderique (University of Geneva, Geneva, N/A, Switzerland)
- Chung, Jean-pierre (University of Geneva, Geneva, N/A, Switzerland)
- Budtz-jorgensen, Ejvind (University of Geneva, Geneva, N/A, Switzerland)
- Müller, Frauke (University of Geneva, Geneva, N/A, Switzerland)

SESSION INFORMATION
Poster Session
Clinical Interventions and Services
03/12/2005

Bellomo, Fleurange (University of Geneva, Geneva, N/A, Switzerland)
Julien, Noëlle (University of Geneva, Geneva, N/A, Switzerland)
De Preux, Fréderique (University of Geneva, Geneva, N/A, Switzerland)
Chung, Jean-pierre (University of Geneva, Geneva, N/A, Switzerland)
Budtz-jorgensen, Ejvind (University of Geneva, Geneva, N/A, Switzerland)
Müller, Frauke (University of Geneva, Geneva, N/A, Switzerland)

Does occupational therapy improve manual performance in tooth/denture

Gerodontology. 2005 Mar;22(1):24-31.

The advantages of occupational therapy in oral hygiene measures for institutionalised elderly adults.

Bellomo F[1], de Preux F, Chung JP, Julien N, Budtz-Jørgensen E, Müller F.

Author information

Abstract

OBJECTIVE: To investigate a new method in teaching and supervising tooth and denture brushing activities by employing occupational therapy techniques.

MATERIALS AND METHODS: Sixty-one residents, 44 women and 17 men, with an average age of 85.7 +/- 6.6 years (range 72-97 years) living in a Long-Term Care home (LTC) in Geneva were enrolled in a randomised controlled trial. They were divided at random into experimental (EG) and control groups (CG) with matched age and sex distribution. Two subjects passed away during the 3-month experimental period. Following medical history, plaque scores and tooth brushing habits were evaluated within the context of a comprehensive clinical assessment. Furthermore, a Mini Mental State and a vision test were taken. Based on the results of these health assessments both the EG and the CG were divided into an 'assisted' (IA) and an 'independent' (II) subgroup. In the EG, tooth brushing was initially taught and in the IA monitored and re-educated once a week by an occupational therapist. In contrast, the CG-IA group received a weekly placebo activity such as manicure by the same person.

RESULTS: From the individual movements taught and monitored by the occupational therapist, opening a tube of toothpaste (n.s.) and denture brushing ($p < 0.05$) were performed more independently after 3 months. Both the occupational therapy and the placebo activity led to a significant improvement in oral ($p < 0.01$ and 0.05) and in denture hygiene ($p < 0.001$ and 0.05). From all participants, the EG-IA subgroup presented the most significant amelioration in plaque ($p < 0.01$) and denture hygiene scores ($p < 0.001$). This group consisted mostly of subjects with an impaired cognitive state.

CONCLUSIONS: Despite the marked placebo effect, the results indicate that occupational therapy is particularly useful to improve the oral and denture hygiene in dependent and cognitively impaired LTC residents and may promote their autonomy in the execution of activities of daily life such as denture brushing.

PMID: 15747895

Five Year Survival following Restoration of Shortened Lower Dental Arches

Five Year Survival following Restoration of Shortened Lower Dental Arches

Removable partial dentures used to restore the shortened lower dental arch may adversely affect the remaining natural teeth and are associated with a low prevalence of use. Objective: To report the findings for prosthesis survival 5 years after restoration of lower shortened arches with bilateral cantilever resin-bonded bridges (RBBs) and conventional partial dentures (RPDs). Design: Randomised controlled trial. Setting: Secondary care. Methods: 25 male and 35 female subjects of median age 67 years were randomly allocated to RBB and RPD treatment groups of 30 patients each matched for age and sex. Interventions: Bilateral cantilever RBBs restoring one occlusal unit, up to but not beyond the second premolar, and conventional free-end saddle RPDs with cast metal frameworks. Using defined criteria, survival or failure of prostheses was noted at each review (3m and then yearly) or as and when problems arose. The unilateral failure of one RBB indicated treatment failure for that patient. Results: 21 subjects (12 RBB and 9 RPD) were lost to follow up at 5 years (5 deceased; 9 withdrew through illness and 7 for personal or other reasons). Survival analysis (modified Kaplan-Meier) indicted mean survival times of 51.3m (95% CI 42.0, 60.5m) and 42.2m (95% CI 33.3, 51.3m) for RBB and RPD groups respectively. Bridges had a slightly lower hazard rate (and were thus more likely to survive) than dentures but the difference was not statistically significant (hazard ratio = 0.62 with 95% CI: 0.28, 1.34). Conclusion: In terms of survival at 5 years, RBBs are at least as effective as RPDs for the restoration of shortened lower dental arches. Supported by the NHS Executive, UK.

Division: IADR/AADR/CADR General Session
Meeting: 2005 IADR/AADR/CADR General Session (Baltimore, Maryland)
Location: Baltimore, Maryland
Year: 2005
Final Presentation ID: 367

Authors

- Jepson, Nicholas John (University of Newcastle upon Tyne, Newcastle upon Tyne, N/A, United Kingdom)
- Moynihan, Paula (University of Newcastle upon Tyne, Newcastle upon Tyne, N/A, United Kingdom)
- Thomason, J. Mark (University of Newcastle upon Tyne, Newcastle, N/A, England, Uk)

Jepson, Nicholas John (University of Newcastle upon Tyne, Newcastle upon Tyne, N/A, United Kingdom)
Moynihan, Paula (University of Newcastle upon Tyne, Newcastle upon Tyne, N/A, United Kingdom)
Thomason, J. Mark (University of Newcastle upon Tyne, Newcastle, N/A, England, Uk)

SESSION INFORMATION
Oral Session
Clinical Outcomes of Prosthodontic Treatment
03/10/2005

Five Year Survival following Restoration of Shortened Lower Dental Arches

Journal of Dental Research

IADR International Association for Dental Research

AADR® American Association for Dental Research

Journal Home Browse Journal Submit Paper About Subscribe Search:

Close

ad PDF

Time to Survival for the Restoration of the Shortened Lower Dental Arch

J.M. Thomason[1]*, P.J. Moynihan[1], N. Steen[2], N.J.A. Jepson[1]

First Published July 1, 2007 | Other

https://doi.org/10.1177/154405910708600712

Abstract

Removable partial dentures may adversely affect remaining tissues and have a low prevalence of use. This randomized controlled trial was designed to compare the time to survival of cantilever resin-bonded fixed partial dentures and conventional removable partial dentures to restore shortened lower dental arches. We randomly allocated 25 male and 35 female patients (median age, 67 years) to fixed or removable partial denture groups of 30 persons, matched for age and sex. Survival of the prostheses was assessed, based on listed criteria, at each review or when problems arose. Although the removable partial denture group required rather more maintenance visits, the difference in survival rates was not statistically significant (hazard ratio = 0.59, with 95% CI 0.27, 1.29). In the absence of significant differences in five-year survival, the reported advantages of fixed partial dentures, including reduced maintenance frequency, offer positive support for the use of resin-bonded fixed partial dentures.

Keywords

denture, partial fixed, resin-bonded, removable, randomized clinical trial, survival

Hybrid Bond and XenoIII in cervical lesions: 6 month results

Hybrid Bond and XenoIII in cervical lesions: 6 month results

Objective: Self etching adhesives are commonly used in restorative dentistry for bonding resin composite to Class V lesions. The aim of the study was to evaluate the clinical performance of adhesive Class V resin composite restorations utilized with two different self etching adhesives (Hybrid Bond, Sun Medical Co., Ltd.; Xeno III, Dentsply/DeTrey) according to the Ryge/CDA-criteria. Method: In accordance to a split mouth study design, 50 patients (57.3 SD±13.5a) received two comparable Class V restorations. The cavities were randomly assigned to the two adhesives: Hybrid Bond lot GV2 and Xeno III lot 0310000129. To obtain comparability, Filtek Supreme was used as the only restorative material in both cases. VL-curing was conducted incrementally for 40 s each (independent of the restorative material). After half a year, 90 % of the restorations were available for a first follow up investigation. Results: After 6 months the results [%] of the Ryge/CDA-evaluation for the two groups Hybrid Bond /Xeno III were: Marginal Adaptation: Alfa: 96/93, Bravo: 4/4, Charlie:0/0, Delta: 0/2; Anatomic Form: Alfa: 91/89, Bravo: 9/7, Charlie 0/4; Secondary Caries: Alfa: 100/100, Bravo: 0/0; Marginal Discoloration: Alfa: 89/93, Bravo: 11/7, Charlie: 0/0; Surface: Romeo: 84/84, Sierra: 13/13, Tango: 0/0, Victor: 2/2; Color Match: Oscar: 44/60, Alfa: 49/38, Bravo: 7/2, Charlie: 0/0; Tooth Vitality: Alfa: 100/96, Bravo: 0/4; Postoperative Sensitivity: Alfa 1: 100/98, Alfa 2: 0/0, Bravo: 0/0, Charlie: 0/2, Delta: 0/0; Integrity of Tooth: Alfa 1: 96/98, Alfa 2: 4/2, Bravo: 0/0, Charlie: 0/0, Delta: 0/0. One restoration where Xeno III was utilized was lost in part. Conclusion: After 6 months, all the restorations - except one - retained and showed clinically acceptable results. No significant difference could be found between both adhesives investigated. This study was supported by Sun Medical Co, Ltd., Japan.

Division: IADR/AADR/CADR General Session
Meeting: 2005 IADR/AADR/CADR General Session (Baltimore, Maryland)
Location: Baltimore, Maryland
Year: 2005
Final Presentation ID: 1786

Authors

- Ernst, Claus-peter (Johannes Gutenberg-University Mainz, Mainz, N/A, Germany)
- Brandenbusch, Mathias (Johannes Gutenberg-University Mainz, Mainz, N/A, Germany)
- Meyer, Gerrit (Johannes Gutenberg-University Mainz, Mainz, N/A, Germany)
- Canbek, Kerem (Johannes Gutenberg-University Mainz, Mainz, N/A, Germany)
- Werling, Ute (Johannes Gutenberg-University Mainz, Mainz, N/A, Germany)
- Willershausen, Brita (Johannes Gutenberg-University Mainz, Mainz, N/A, Germany)

Ernst, Claus-peter (Johannes Gutenberg-University Mainz, Mainz, N/A, Germany)
Brandenbusch, Mathias (Johannes Gutenberg-University Mainz, Mainz, N/A, Germany)
Meyer, Gerrit (Johannes Gutenberg-University Mainz, Mainz, N/A, Germany)
Canbek, Kerem (Johannes Gutenberg-University Mainz, Mainz, N/A, Germany)
Werling, Ute (Johannes Gutenberg-University Mainz, Mainz, N/A, Germany)
Willershausen, Brita (Johannes Gutenberg-University Mainz, Mainz, N/A, Germany)

SESSION INFORMATION

Poster Session

Dental Adhesive Systems and Sealants

03/11/2005

Hybrid Bond and XenoIII in cervical lesions: 6 month results

Clin Oral Investig. 2008 Sep;12(3):225-32. doi: 10.1007/s00784-008-0193-9. Epub 2008 Mar 28.

Two-year clinical performance of two one-step self-etching adhesives in the restoration of cervical lesions.

Schattenberg A[1], Werling U, Willershausen B, Ernst CP.

Author information

1 Clinic for Restorative Dentistry, Johannes Gutenberg-University of Mainz, Augustusplatz 2, 55131 Mainz, Germany. schattan@uni-mainz.de

Abstract

The aim of the study was to evaluate the clinical performance of two different one-step self-etching adhesives (Hybrid Bond/Sun Medical, Xeno III/Dentsply) in adhesive cervical resin composite restorations. In accordance with a split-mouth study design, 50 patients (57.3+/-13.5) received at least one pair of restorations. In each of two comparable cervical cavities, either the adhesive systems Hybrid Bond or Xeno III was used with the resin composite Filtek Supreme (3M ESPE). After 6, 12 and 24 months, the restorations were scored according to the Ryge and California Dental Association criteria. After 2 years, the resulting scores (percent) of the Ryge evaluation for the groups Hybrid Bond/ Filtek Supreme and Xeno III/ Filtek Supreme were marginal integrity, Alpha (92/78), Bravo (8/2), Charlie (0/0) and Delta (0/10); anatomic form, Alpha (92/82), Bravo (8/8) and Charlie (0/10); secondary caries, Alpha (100/100) and Bravo (0/0); marginal discoloration, Alpha (80/84), Bravo (20/12), Charlie (0/0) and not available (0/4); color match, Oscar (39/47), Alpha (51/45), Bravo (10/4), Charlie (0/0) and not available (0/4); surface, Romeo (78/69), Sierra (22/22), Tango (0/0) and Victor (0/10); tooth vitality, Alpha (98/94), Bravo (2/6); and integrity of tooth, alpha 1 (96/96) and alpha 2 (4/4). After 2 years, all Hybrid Bond restorations were retained and showed clinically acceptable results, while five Xeno III restorations were lost in part or in toto. For marginal integrity, anatomic form and surface, significant differences ($p<0.05$) were found but did not prove statistically significant after Bonferroni adjustment.

PMID: 18369669 DOI: 10.1007/s00784-008-0193-9

Outcomes from a Randomised Controlled Trial of Implant Overdentures

Outcomes from a Randomised Controlled Trial of Implant Overdentures

Implant overdentures offer the possibility of overcoming some of the limitations of conventional dentures. Objective: To conduct a randomised clinical trial of implant retained overdentures in an sample of older UK adults. Method: Edentulous adults attending Newcastle Dental Hospital, UK were randomly assigned into two treatment groups, one provided with an implant retained overdenture in the mandible and conventional maxillary denture (IG, n=45) and a group provided with conventional dentures (DG, n=47). The groups were balanced by gender and operator. The "intention to treat" principle was used to deal with subjects who refused implants. Both groups completed the Oral Health Impact Profile pre- and three months post treatment, and a validated denture satisfaction scale with a response range of 1 (totally satisfied) – 5 (very dissatisfied). Summary OHIP scores were calculated by adding the response codes (ranging from 0-4) across the 49 OHIP statements. Results: In the implant group, 33 subjects had implants, and 12 subjects were provided with complete dentures. There were no significant pre-treatment differences between the groups for denture satisfaction, with both groups reporting a high level of dissatisfaction with their dentures [mean satisfaction: IG = 4.6; DG = 4.5]. Pre-treatment OHIP scores were higher for IG subjects (99.1) than DG subjects (78.6) [P=0.02, unpaired t-test]. Following treatment, both groups reported a large improvement in satisfaction (IG = 2.5; DG = 2.9) and OHRQoL (IG=44.7; DG = 39.0). Between group differences were not significant for either post treatment or change scores. Inclusion of refusers in the IG masked between group differences reported by the subjects who accepted implants. Conclusion: It was concluded that, when following the intention to treat principle, the implant group and conventional denture group reported similar impact on oral health related quality of life and satisfaction in this group of older adults.

Division: IADR/AADR/CADR General Session
Meeting: 2005 IADR/AADR/CADR General Session (Baltimore, Maryland)
Location: Baltimore, Maryland
Year: 2005
Final Presentation ID: 1364

Authors

- Allen, Patrick Finbarr (National University of Ireland - Cork, Cork, N/A, Ireland)
- Thomason, J. Mark (University of Newcastle upon Tyne, Newcastle, N/A,)
- Jepson, Nicholas John (University of Newcastle upon Tyne, Newcastle upon Tyne, N/A, United Kingdom)
- Nohl, Francis (University of Newcastle upon Tyne, Newcastle upon Tyne, N/A, United Kingdom)
- Smith, David (University of Newcastle upon Tyne, , N/A, United Kingdom)
- Ellis, Janice (University of Newcastle upon Tyne, Newcastle upon Tyne, N/A, United Kingdom)

Allen, Patrick Finbarr (National University of Ireland - Cork, Cork, N/A, Ireland)
Thomason, J. Mark (University of Newcastle upon Tyne, Newcastle, N/A,)
Jepson, Nicholas John (University of Newcastle upon Tyne, Newcastle upon Tyne, N/A, United Kingdom)
Nohl, Francis (University of Newcastle upon Tyne, Newcastle upon Tyne, N/A, United Kingdom)
Smith, David (University of Newcastle upon Tyne, , N/A, United Kingdom)
Ellis, Janice (University of Newcastle upon Tyne, Newcastle upon Tyne, N/A, United Kingdom)

SESSION INFORMATION
Oral Session
Clinical Research and Interventions
03/11/2005

Outcomes from a Randomised Controlled Trial of Implant

Journal of Dental Research

IADR International Association for Dental Research

AADR® American Association for Dental Research

Journal Home | Browse Journal | Submit Paper | About | Subscribe

Close

d PDF

le

ON

& Tables

A Randomized Controlled Trial of Implant-retained Mandibular Overdentures

P.F. Allen[1]*, J.M. Thomason[2], N.J.A. Jepson[2], F. Nohl[2], D.G. Smith[2], J. Ellis[2]

Show less

First Published June 1, 2006 | Other

https://doi.org/10.1177/154405910608500613

Article information

Article Information

Volume: 85 issue: 6, page(s): 547-551

Issue published: June 1, 2006

P.F. Allen[1], *, J.M. Thomason[2], N.J.A. Jepson[2], F. Nohl[2], D

[1]National University of Ireland, Cork, Ireland; and

[2]University of Newcastle upon Tyne, UK

Corresponding Author: *corresponding author, University D

f.allen@ucc.ie

Abstract

Evidence from randomized clinical trials of implant-retained overdentures is very limited at the present time. The aim of this study was to compare implant-retained mandibular overdentures and conventional complete dentures in a randomized controlled trial (RCT). Our *a priori* hypothesis was that implant-retained mandibular overdentures would be significantly better than conventional complete dentures. Edentulous patients ($n = 118$) were randomly allocated to either an Implant Group ($n = 62$) or a Denture Group ($n = 56$). Patients completed the Oral Health Impact Profile (OHIP) and a denture satisfaction scale pre-treatment and three months post-treatment. Upon completion of treatment, both groups reported improvement ($p < 0.001$, Wilcoxon Ranks Sum test) in oral-health-related quality of life and denture satisfaction. There were no significant post-treatment differences between the groups, but a treatment effect may be masked by application of “intention to treat” analysis. The OHIP change scores were significantly greater for patients receiving implants than for those who refused them.

Keywords

implants, overdentures, quality of life, randomized controlled trial

Simple versus advanced fabrication of complete dentures: Chewing

Simple versus advanced fabrication of complete dentures: Chewing ability

For the fabrication of complete dentures, a comprehensive procedure involving a facebow transfer, intraoral tracings for the identification of centric relation and the use of semi-anatomic teeth is deemed to have a positive influence on occlusion and chewing ability. Objectives: To determine if patient ratings of their ability to chew are dependent on the method of complete denture fabrication. Methods: Ten patients (mean age 70.5 yrs, SD 7.8) participated in a randomized within-subject crossover trial. Each participant received two sets of new complete dentures. One pair (Gerber Prosthesis, GEP) was manufactured based on an intraoral tracing of centric relation with a registration plate and facebow transfer. Semi-anatomic teeth with a reduced occlusal pattern were chosen (Condylofom®). The other pair was made using a simplified procedure without facebow transfer. Jaw relations were recorded with wax occlusion rims and anatomic teeth (Merz®) were selected (Gysi Prosthesis, GYP). The dentures were delivered in randomized order, and each was worn for three months. Patients' ratings of chewing ability of seven index foods were measured on visual analog scales (VAS) with the original prostheses, and three months after the insertion of each new prosthesis, respectively. Between-group comparisons were carried out using t-tests; paired t-tests were used for pre- and post-treatment comparisons. Results: In the pre-/post-treatment comparison, participants rated their ability to chew hard cheese, carrots, hard sausages, steaks, and apples significantly better than with their original prostheses ($p<0.05$, paired t-tests). There were no improvements for white bread and salad. There were also no statistically significant differences between the GEP and GYP treatments. Conclusion: Comprehensive measures for the fabrication of complete dentures, when compared to more simple procedures, do not appear to influence the perceived chewing ability.
This study was supported by Merz Dental GmbH (Lütjenburg, Germany)

Division: IADR/AADR/CADR General Session
Meeting: 2005 IADR/AADR/CADR General Session (Baltimore, Maryland)
Location: Baltimore, Maryland
Year: 2005
Final Presentation ID: 1617

Authors

- Vogeler, Michael (University Hospital Freiburg, Freiburg, N/A, Germany)
- Heydecke, Guido (University Hospital Freiburg, Freiburg, N/A, Germany)
- Türp, Jens C (University of Basel, Basel, N/A, Switzerland)
- Strub, Joerg R (University Hospital Freiburg, Freiburg, N/A, Germany)

SESSION INFORMATION
Poster Session
Complete Denture and Overdenture Research
03/11/2005

Vogeler, Michael (University Hospital Freiburg, Freiburg, N/A, Germany)
Heydecke, Guido (University Hospital Freiburg, Freiburg, N/A, Germany)
Türp, Jens C (University of Basel, Basel, N/A, Switzerland)
Strub, Joerg R (University Hospital Freiburg, Freiburg, N/A, Germany)

Simple versus advanced fabrication of complete dentures: Chewing

Gerodontology. 2007 Jun;24(2):77-86.

Patient ratings of chewing ability from a randomised crossover trial: lingualised vs. first premolar/canine-guided occlusion for complete dentures.

Heydecke G[1], Akkad AS, Wolkewitz M, Vogeler M, Türp JC, Strub JR.

Author information

1 Department of Prosthodontics, School of Dentistry, University Hospital, Freiburg, Germany. guido.heydecke@uniklinik-freiburg.de

Abstract

BACKGROUND: Complex procedures involving a facebow transfer and the use of lingualised teeth are deemed to have a positive influence on the chewing ability with complete dentures.

OBJECTIVES: To determine if patients' ratings of their ability to chew depend on the method of complete denture fabrication.

METHODS: Edentulous patients (n = 20) participated in a within-subject crossover trial. Each patient received two sets of new complete dentures. One pair was manufactured based on intraoral tracing of centric relation and facebow transfer; semi-anatomical teeth with lingualised occlusion denture (LOD) were chosen. The second pair was made using a simplified procedure without facebow transfer; jaw relations were recorded with wax occlusion rims, and anatomical teeth with a first premolar/canine-guidance (CGD) were selected. The dentures were delivered in randomised order, and each was worn for 3 months. Three months after delivery, patients' ratings of each new prosthesis were recorded on visual analogue scales for their ability to chew seven index foods. Repeated measurements analysis of variance was performed to investigate possible carry-over effects accounting for confounding by treatment period.

RESULTS: When comparing the two treatments, participants rated their ability to chew in general, to masticate carrots, hard sausage, steak and raw apple in particular, was significantly better with the CGD (anatomical teeth) than with the LOD ($p < 0.05$).

CONCLUSION: Comprehensive methods for the fabrication of complete dentures including semi-anatomical lingualised teeth and a full registration do not seem to influence the perceived chewing ability, when compared with more simple procedures. Chewing ability for tough foods appears to benefit from the use of anatomical teeth.

PMID: 17518954 DOI: 10.1111/j.1741-2358.2007.00153.x

Simple versus advanced fabrication of complete dentures: patient

Simple versus advanced fabrication of complete dentures: patient satisfaction

It is unclear if comprehensive measures for the fabrication of complete dentures, including facebow transfer, intraoral tracings for the determination of centric relation, and semi-anatomic teeth lead to greater patient acceptance. Objectives: The aim of this clinical trial was to compare patient satisfaction of new complete dentures manufactured with either simple or advanced methods. Methods: Ten patients (♀: 5, ♂: 5; mean age 70.5 yrs) participated in a randomized within-subject crossover trial. They received two sets of new complete dentures. One pair (Gerber Prosthesis, GEP) was manufactured based on an intraoral recording of centric relation with a registration plate and facebow transfer. Semi-anatomic teeth with a reduced occlusal pattern were chosen. The other pair was made using a simplified procedure without facebow transfer. Vertical and horizontal jaw relations were recorded with wax occlusion rims; anatomic teeth (Merz®) were selected (Gysi Prosthesis, GYP). The dentures were delivered in randomized order, and each was worn for three months. Patients' ratings of general satisfaction, comfort, speech, stability, esthetics, and ease of cleaning were measured with 100 mm visual analog scales (VAS). Ratings were obtained with the original prostheses, and three months after the insertion of the first and second prosthesis. Between-group comparisons were carried out using t-tests; paired t-tests were used for pre- and post-treatment comparisons. Results: In the pre- and post-treatment comparison, participants rated their general satisfaction, comfort, and stability significantly better than with their original prostheses ($p<0.05$). There were no improvements for speech, esthetics, or ease of cleaning. There were also no significant differences between GEP and GYP treatments. Conclusion: For the fabrication of complete dentures, comprehensive measures, when compared to more simple procedures, do not seem to have advantages on patient ratings of general satisfaction, comfort, speech, stability, esthetics, and ease of cleaning.

Supported by Merz Dental GmbH (Lütjenburg, Germany)

Division: IADR/AADR/CADR General Session
Meeting: 2005 IADR/AADR/CADR General Session (Baltimore, Maryland)
Location: Baltimore, Maryland
Year: 2005
Final Presentation ID: 2769

Authors

- Heydecke, Guido (University Hospital Freiburg, Freiburg, N/A, Germany)
- Vogeler, Michael (University Hospital Freiburg, Freiburg, N/A, Germany)
- Türp, Jens C (University of Basel, Basel, N/A, Switzerland)
- Strub, Joerg R (University Hospital Freiburg, Freiburg, N/A, Germany)

Heydecke, Guido (University Hospital Freiburg, Freiburg, N/A, Germany)
Vogeler, Michael (University Hospital Freiburg, Freiburg, N/A, Germany)
Türp, Jens C (University of Basel, Basel, N/A, Switzerland)
Strub, Joerg R (University Hospital Freiburg, Freiburg, N/A, Germany)

SESSION INFORMATION

Oral Session

Removable and Maxillofacial Prosthodontics

03/12/2005

Simple versus advanced fabrication of complete dentures: patient

Quintessence Int. 2008 Feb;39(2):107-16.

Simplified versus comprehensive fabrication of complete dentures: patient ratings of denture satisfaction from a randomized crossover trial.

Heydecke G[1], Vogeler M, Wolkewitz M, Türp JC, Strub JR.

Author information

Abstract

OBJECTIVE: To determine if patient ratings of their ability to chew are dependent on the method of complete denture fabrication.

METHOD AND MATERIALS: Twenty edentulous patients participated in a randomized within-subject crossover trial. Each participant received 2 sets of new complete dentures. One pair (Gerber prosthesis) was manufactured based on tracings (to determine centric relation) and facebow transfer; semianatomic teeth with a lingualized and balanced occlusal pattern were used. The other set of complete dentures was made using a simplified procedure without facebow transfer; jaw relations were recorded with wax occlusion rims, and anatomic teeth were set with a canine and premolar guidance (Gysi prosthesis). The 2 dentures were delivered in randomized order, and each was worn for 3 months. Three months after insertion, patients' ratings of each new prosthesis were obtained on visual analog scales for general satisfaction, comfort, ability to speak, stability, esthetics, ease of cleaning, and ability to chew.

RESULTS: Patients rated their general satisfaction, stability, and esthetic appearance significantly better for the Gysi prostheses ($P < .05$). No significant differences between the 2 denture treatment methods were detected for ability to speak, comfort, chewing ability, and the ease of cleaning the dentures.

CONCLUSION: A comprehensive method for the fabrication of complete dentures using lingualized teeth does not appear to positively influence patient ratings of denture satisfaction when compared to a simple procedure with anatomic teeth.

PMID: 18560649

Sleep Bruxism Reduction by Double Arch Device and Occlusal Splint

Sleep Bruxism Reduction by Double Arch Device and Occlusal Splint

A recent study suggested that sleep bruxism (SB) is significantly reduced by use of a single upper occlusal splint (Dubé et al., JDR, 2004). Objectives: To compare the efficacy of a double arch device (DAD) vs. a single upper occlusal splint (OS).
Methods: A group of 13 patients with a sleep diagnosis of SB participated in this randomized controlled crossover study. Subjects were recorded in the sleep laboratory for 6 nights. The DAD (Silencer Custom II, BC, Canada), designed to treat sleep apnea, was used in 3 configurations: without rod between arches (freedom of movement) and in slightly ($<40\%$) and pronounced ($>75\%$) lower arch advancement positions. Masticatory muscle activity was evaluated in SB episodes per hour and SB bursts per hour and statistically analyzed (repeated measures ANOVA followed by paired comparisons). Pain felt during the previous night was scored on a 1-5 scale and analyzed with Friedman two-way ANOVA.

Results: A statistically significant reduction in the number of SB episodes per hour (decrease of 42%, $p=0.002$) and SB bursts per hour (decrease of 51%, $p<0.0001$) was observed between baseline night and night with OS. Comparisons between night with OS and nights with DAD revealed a further reduction in all configurations (all p's<0.05). However, 8 patients over 13 reported an increase in oro-dental pain when baseline night was compared with nights with DAD both in slightly and pronounced advancement positions ($p=0.02$).

Conclusion: The exact cause of the reduction of SB with DAD remains to be explained: thickness of device, pain, reduction of movement freedom or change in airway patency.

Supported by Canadian CIHR and Québec FRSQ. DAD were provided free.

Division: IADR/AADR/CADR General Session
Meeting: 2005 IADR/AADR/CADR General Session (Baltimore, Maryland)
Location: Baltimore, Maryland
Year: 2005
Final Presentation ID: 204

Authors

- Landry, Marie-lou (Universite de Montreal, Montreal, QC, Canada)
- Rompré, Pierre H (Universite de Montreal, Montreal, QC, Canada)
- Manzini, Christiane (Universite de Montreal, Montreal, QC, Canada)
- Guitard, Francine (Universite de Montreal, Montreal, QC, Canada)
- De Grandmont, Pierre (Universite de Montreal, Montreal, QC, Canada)
- Lavigne, Gilles J (Universite de Montreal, Montreal, QC, Canada)

Landry, Marie-lou (Universite de Montreal, Montreal, QC, Canada)
Rompré, Pierre H (Universite de Montreal, Montreal, QC, Canada)
Manzini, Christiane (Universite de Montreal, Montreal, QC, Canada)
Guitard, Francine (Universite de Montreal, Montreal, QC, Canada)
De Grandmont, Pierre (Universite de Montreal, Montreal, QC, Canada)
Lavigne, Gilles J (Universite de Montreal, Montreal, QC, Canada)

SESSION INFORMATION
Oral Session
Keynote Address and TMD Diagnosis and Treatment
03/10/2005

Sleep Bruxism Reduction by Double Arch Device and Occlusal Splint

Int J Prosthodont. 2006 Nov-Dec;19(6):549-56.

Reduction of sleep bruxism using a mandibular advancement device: an experimental controlled study.

Landry ML[1], Rompré PH, Manzini C, Guitard F, de Grandmont P, Lavigne GJ.

Author information

1 Faculté de Médecine Dentaire, Université Laval, Canada.

Abstract

PURPOSE: The objective of this experimental study was to compare the effect on sleep bruxism and tooth-grinding activity of a double-arch temporary custom-fit mandibular advancement device (MAD) and a single maxillary occlusal splint (MOS).

MATERIALS AND METHODS: Thirteen intense and frequent bruxors participated in this short-term randomized crossover controlled study. All polygraphic recordings and analyses were made in a sleep laboratory. The MOS was used as the active control condition and the MAD was used as the experimental treatment condition. Designed to temporarily manage snoring and sleep apnea, the MAD was used in 3 different configurations: (1) without the retention pin between the arches (full freedom of movement), (2) with the retention pin in a slightly advanced position (< 40%), and (3) with the retention pin in a more advanced position (> 75%) of the lower arch. Sleep variables, bruxism-related motor activity, and subjective reports (pain, comfort, oral salivation, and quality of sleep) were analyzed with analysis of variance and the Friedman test.

RESULTS: A significant reduction in the number of sleep bruxism episodes per hour (decrease of 42%, P < .001) was observed with the MOS. Compared to the MOS, active MADs (with advancement) also revealed a significant reduction in sleep bruxism motor activity. However, 8 of 13 patients reported pain (localized on mandibular gums and/or anterior teeth) with active MADs.

CONCLUSIONS: Short-term use of a temporary custom-fit MAD is associated with a remarkable reduction in sleep bruxism motor activity. To a smaller extent, the MOS also reduces sleep bruxism. However, the exact mechanism supporting this reduction remains to be explained. Hypotheses are oriented toward the following: dimension and configuration of the appliance, presence of pain, reduced freedom of movement, or change in the upper airway patency.

PMID: 17165292

TMD Pain Reduction in Splint Therapy Associated with Reduced Parafunctions

TMD Pain Reduction in Splint Therapy Associated with Reduced Parafunctions

Objectives: Interocclusal splints may be an effective modality in the management of temporomandibular disorders (TMD), but there is little evidence regarding the mechanism by which splints work. This study tested the hypothesis that pain reduction produced by splints is associated with reduction in parafunctional activity. Methods: This was a two-group, single-blinded randomized clinical trial. Ten individuals diagnosed with myofascial pain or myofascial pain and arthralgia according to the Research Diagnostic Criteria completed the study. All patients received full coverage hard maxillary stabilization splints. Six patients received instructions to maintain contact with the splint, and four were instructed to avoid contact. Experience sampling methodology was used to collect data on pain and parafunctional behaviors for one week prior to the start of treatment and during the final week of treatment. Patients were reminded approximately every two hours to maintain or avoid contact with the splint. Parafunctional activity was assessed as the mean intensity of tooth contact measured on a four-point scale. Degree of change in parafunctional activity was expressed as the slope of a best fit regression line. Facial and jaw pain were assessed on an 11-point interval scale. Results: Pain diminished significantly for all patients during treatment, $F(1,8) = 8.07$, $p < .01$, and there were no significant group or group by time interactions. Three additional patients, two from the Avoid Contact and one from the Maintain Contact, terminated treatment early. The amount of change in tooth contact accounted for a significant proportion of the variance in pain change scores, $F(1,7) = 5.85$, $p < .05$. Reductions in tooth contact were associated with greater relief from pain. Conclusions: Reduction of parafunctional tooth contact results in diminished pain. Splints may produce therapeutic effects by reducing parafunctional activities associated with TMD pain. Supported by a grant from National Institutes of Health, DE13563.

Division: IADR/AADR/CADR General Session
Meeting: 2005 IADR/AADR/CADR General Session (Baltimore, Maryland)
Location: Baltimore, Maryland
Year: 2005
Final Presentation ID: 203

Authors

- Glaros, Alan G. (Kansas City University of Medicine and Biosciences, Kansas City, MO, USA)
- Owais, Zaidoon R. (University of Missouri -Kansas City, Kansas City, MO, USA)
- Lausten, Leonard L. (University of Missouri -Kansas City, Kansas City, MO, USA)

Glaros, Alan G. (Kansas City University of Medicine and Biosciences, Kansas City, MO, USA)
Owais, Zaidoon R. (University of Missouri -Kansas City, Kansas City, MO, USA)
Lausten, Leonard L. (University of Missouri -Kansas City, Kansas City, MO, USA)

SESSION INFORMATION
Oral Session
Keynote Address and TMD Diagnosis and Treatment
03/10/2005

TMD Pain Reduction in Splint Therapy Associated with Reduced Parafunctions

Journal of Oral Rehabilitation

Full Access

Reduction in parafunctional activity: a potential mechanism for the effectiveness of splint therapy

A. G. GLAROS, Z. OWAIS, L. LAUSTEN

First published: 28 November 2006 | https://doi.org/10.1111/j.1365-2842.2006.01660.x | Cited by: 19

Dr Alan G. Glaros, Associate Dean, Kansas City University of Medicine and Biosciences, 1750 Independence Ave. Kansas City, MO 64106.
E-mail: aglaros@kcumb.edu

Abstract

SUMMARY Interocclusal splints may be an effective modality in the management of temporomandibular disorders (TMD), but there is little evidence regarding the mechanism by which splints work. This study tested the hypothesis that pain reduction produced by splints is associated with reduction in parafunctional activity. In a two-group, single-blinded randomized clinical trial, patients diagnosed with myofascial pain received full coverage hard maxillary stabilization splints. Patients were instructed to maintain or avoid contact with the splint for the 6 weeks of active treatment. Patients who decreased the intensity of tooth contact were expected to show the greatest alleviation of pain, and those who maintained or increased contact were expected to report lesser reductions in pain. Experience-sampling methodology was used to collect data on pain and parafunctional behaviours at pre-treatment and during the final week of treatment. Patients were reminded approximately every 2 h by pagers to maintain/avoid contact with the splint. The amount of change in intensity of tooth contact accounted for a significant proportion of the variance in pain change scores. Patients who reduced tooth contact intensity the most reported greater relief from pain. Splints may produce therapeutic effects by reducing parafunctional activities associated with TMD pain.

2006
