# Supplementary material for: Reporting inconsistency between published conference abstracts and article abstracts of randomised controlled trials in prosthodontics presented at IADR general sessions
Source: PeerJ. 2023 May 5;11:e15303. doi: 10.7717/peerj.15303 (PMC10166077; doi:10.7717/peerj.15303)
Supplement: Supplemental Information 2 [file peerj-11-15303-s002.pdf]

A Randomised Trial of Food Choices Made By Edentulous Adults

A Randomised Trial of Food Choices Made By Edentulous Adults

Tooth loss is associated with poor diet, and edentulous adults tend to have a diet which is dominated by carbohydrates and low in fibre. There is limited evidence to show that food choices of patients provided with implant retained prostheses (IRPs) are better than those provided with conventional dentures (CDs). Objective:The objective of the study was to conduct a randomised clinical trial to compare food choices of edentulous adults provided with IRPs and CDs. Method: Edentulous patients awaiting conventional dentures at Newcastle Dental Hospital, UK, were randomly allocated to two treatment groups, an implant group (IG) and a denture group (DG). IG subjects (n=45) were provided with a conventional denture in the maxilla, and an implant retained overdenture in the mandible. Subjects who refused implants were retained in IG using the "intention to treat" principle. DG subjects (n=46) were provided with conventional dentures. All subjects were asked to indicate whether they consumed any of the following foods: bread, cheese, carrot, apple, bacon, nuts and lettuce. They were also asked to indicate their level of difficulty (ranging from "no difficulty" to "extreme difficulty") chewing these foods. Data were collected pre-treatment and three months post-treatment. Results: IG subjects reported increased consumption of carrots and apples (McNemar's test, $p<0.05$), but no other significant differences were detected. Within - groups, IG subjects reported decreased difficulty chewing all foods except bread, whereas DG subjects reported decreased difficulty chewing carrot, apple, bacon and nuts only (Wilcoxon test, $p<0.05$). Between-group differences were only detected for nuts, which DG subjects found easier to chew than IG subjects ($p=0.002$, Mann Whitney U test). Conclusion: It was concluded that both implant retained overdentures and conventional dentures reduced perceived difficulty chewing foods. The food choices made by subjects in both groups did not alter to a major degree following either treatment.

Division: IADR General Session
Meeting: 2006 IADR General Session (Brisbane, Australia)
Location: Brisbane, Australia
Year: 2006
Final Presentation ID: 546

Authors

- Allen, Patrick Finbarr (National University of Ireland - Cork, Cork, N/A, Ireland)
- Thomason, J. Mark (University of Newcastle upon Tyne, Newcastle upon Tyne, N/A, United Kingdom)
- Jepson, Nicholas (University of Newcastle upon Tyne, Newcastle upon Tyne, N/A, United Kingdom)
- Nohl, F (University of Newcastle upon Tyne, Newcastle upon Tyne, N/A, United Kingdom)
- Smith, D (University of Newcastle upon Tyne, Newcastle upon Tyne, N/A, United Kingdom)
- Ellis, Janice (University of Newcastle upon Tyne, Newcastle upon Tyne, N/A, United Kingdom)

Allen, Patrick Finbarr (National University of Ireland - Cork, Cork, N/A, Ireland)
Thomason, J. Mark (University of Newcastle upon Tyne, Newcastle upon Tyne, N/A, United Kingdom)
Jepson, Nicholas (University of Newcastle upon Tyne, Newcastle upon Tyne, N/A, United Kingdom)
Nohl, F (University of Newcastle upon Tyne, Newcastle upon Tyne, N/A, United Kingdom)
Smith, D (University of Newcastle upon Tyne, Newcastle upon Tyne, N/A, United Kingdom)
Ellis, Janice (University of Newcastle upon Tyne, Newcastle upon Tyne, N/A, United Kingdom)

SESSION INFORMATION
Oral Session
Gerodontology Quality of Life/Caries Epidemiology
06/29/2006

A Randomised Trial of Food Choices Made By Edentulous Adu[illegible]

CLINICAL ORAL IMPLANTS RESEARCH WILEY

Full Access

A randomized-controlled trial of food choices made by edentulous adults

J. S. Ellis, J. M. Thomason, N. J. Jepson, F. Nohl, D. G. Smith, P. F. Allen

First published: 20 February 2008 | https://doi.org/10.1111/j.1600-0501.2007.01488.x | Cited by: 25

Correspondence to:
J. S. Ellis
School of Dental Sciences Newcastle University
Framlington Place
Newcastle upon Tyne
NE2 4BW
UK
e-mail: j.s.ellis@ncl.ac.uk

Ctrl+Shift+X
尺寸:1874 x 920
RGB:(255,255,255)
双击可完成复制

Abstract

Aim: The aim of this study was to conduct a randomized-controlled trial to compare food choices of edentulous adults provided with implant-supported mandibular overdentures and conventional dentures.

Methods: Edentulous patients were randomly allocated to an implant group (IG) or a denture group (DG). IG subjects (n=49) were provided with conventional maxillary dentures and implant-retained mandibular overdentures. Subjects in this group refusing implants were retained using the 'intention-to-treat principle' and provided with conventional dentures. DG subjects (n=48) were provided with conventional dentures.

Subjects indicated whether they consumed any of seven test foods and the level of chewing difficulty experienced. Data were collected pre-treatment and 3 months post treatment.

Results: IG subjects reported increased consumption of carrots, apples and nuts post-treatment ($P<0.05$) and decreased post-treatment difficulty in chewing apples and nuts. DG subjects reported decreased post-treatment difficulty in chewing carrots, bacon and nuts ($P<0.05$). Between group differences for chewing difficulty were detected for nuts that DG subjects found easier to chew than IG subjects ($P=0.002$).

Conclusion: Food selection and perceived chewing difficulty improved in both groups, with no significant differences between groups.

Successful rehabilitation may not result in different food selection, which may require concurrent tailored dietary interventions, but may increase available food choices.

A Randomized Clinical Trial on Cusp-Replacing Composite Restorations: One-

A Randomized Clinical Trial on Cusp-Replacing Composite Restorations: One-Year Results

Objectives: To assess the one-year clinical performance of direct and indirect cusp-replacing resin composite restorations.
Methods: In this randomized clinical trial, 1 year after placing 106 cusp-replacing resin composite restorations in 94 patients, 79 patients could be recalled with 90 restorations made (85%). Restorations were made by two operators in upper premolars with Class II cavities with a missing buccal or palatal cusp, following a strict protocol. Treatment technique and operator were randomly assigned. Seventy-nine patients with 47 direct (Clearfil AP-X, Kuraray) and 43 indirect (Estenia, Kuraray) restorations could be retrieved for one-year evaluation. Evaluation included presence of occlusal and proximal contacts. The patients were interviewed at baseline and after one year to record post-operative sensitivity.

Results: Eighty-nine restorations (99%) were rated as clinically acceptable. One premolar with a direct restoration was re-restored four months after treatment due to persistent post-operative sensitivity. Directly after treatment occlusal contacts were present in 92% of direct and in 98% of indirect restorations; after one year this was 94% and 98% respectively (both Chi-square, P>0.05). Proximal contacts changed from 98% present directly after treatment to 94% after one year for direct restorations; for indirect restorations no change was found (95% present) (both Chi-square, P>0.05). Post-operative sensitivity directly after treatment was reported for 9% of direct restorations and for 10% of indirect restorations (Chi-square, P>0.05) and decreased to 0% for both techniques after 1 year.

Conclusions: Over a one-year period direct and indirect resin composite restorations are equally effective in the treatment of premolars with a missing cusp.

Division: IADR General Session
Meeting: 2006 IADR General Session (Brisbane, Australia)
Location: Brisbane, Australia
Year: 2006
Final Presentation ID: 2018

Authors

- Fennis, Willem Maarten Maria (University of Nymegen, Nijmegen, N/A, Netherlands)
- Kuijs, Ruud H. (University of Nymegen, Nijmegen, N/A, Netherlands)
- Kreulen, Cees M. (University of Nymegen, Nijmegen, N/A, Netherlands)
- Roeters, F. Joost M. (University of Nymegen, Nijmegen, N/A, Netherlands)
- Burgersdijk, Rob C.w. (University of Nymegen, Nijmegen, N/A, Netherlands)
- Creugers, Nico H.j. (University of Nymegen, Nijmegen, N/A, Netherlands)

Fennis, Willem Maarten Maria (University of Nymegen, Nijmegen, N/A, Netherlands)
Kuijs, Ruud H. (University of Nymegen, Nijmegen, N/A, Netherlands)
Kreulen, Cees M. (University of Nymegen, Nijmegen, N/A, Netherlands)
Roeters, F. Joost M. (University of Nymegen, Nijmegen, N/A, Netherlands)
Burgersdijk, Rob C.w. (University of Nymegen, Nijmegen, N/A, Netherlands)
Creugers, Nico H.j. (University of Nymegen, Nijmegen, N/A, Netherlands)

SESSION INFORMATION
Oral Session
Clinical Investigations
06/30/2006

A Randomized Clinical Trial on Cusp-Replacing Composite Restorations: One-Year Results

Journal of Dental Research

IADR International Association for Dental Research

AADR® American Association for Dental Research

Close

PDF

Randomized Control Trial of Composite Cuspal Restorations

Five-year Results

W.M. Fennis*, R.H. Kuijs, F.J. Roeters, N.H. Creugers, C.M. Kreulen

Show less

First Published October 23, 2013 | Research Article | Check for updates

https://doi.org/10.1177/0022034513510946

Article information

Abstract

The objective of this randomized control trial was to compare the five-year clinical performance of direct and indirect resin composite restorations replacing cusps. In 157 patients, 176 restorations were made to restore maxillary premolars with Class II cavities and one missing cusp. Ninety-two direct and 84 indirect resin composite restorations were placed by two operators, following a strict protocol. Treatment technique and operator were assigned randomly. Follow-up period was at least 4.5 yrs. Survival rates were determined with time to reparable failure and complete failure as endpoints. Kaplan-Meier five-year survival rates were 86.6% (SE 0.27%) for reparable failure and 87.2% (SE 0.27%) for complete failure. Differences between survival rates of direct and indirect restorations [89.9% (SE 0.34%) *vs.* 83.2% (SE 0.42%) for reparable failure and 91.2% (SE 0.32%) *vs.* 83.2% (SE 0.42%) for complete failure] were not statistically significant (p = .23 for reparable failure; p = .15 for complete failure). Mode of failure was predominantly adhesive. The results suggest that direct and indirect techniques provide comparable results over the long term (trial registration number: ISRCTN29200848).

Clinical outcomes and esthetic results of non-submerged immediate implants

Clinical outcomes and esthetic results of non-submerged immediate implants

Objectives: To (i) evaluate healing of grafted marginal defects, and (ii) assess the soft tissue and radiographic outcomes over a period up to 4 years in immediate transmucosal implants. Methods: 30 immediate transmucosal implants in maxillary anterior extraction sites of 30 patients were treated randomly with BioOssTM (BG), BioOssTM and resorbable collagen membrane (BG+M) and a non-grafted control group (C). Following surgical re-entry after six months, patients were followed clinically and radiographically for 3-years following completion of the implant crown. Results: All successfully integrated. Vertical defect height (VDH) reductions of 81.2 ± 5.04%, 70.45 ± 17.4% and 68.2 ± 16.6%, and horizontal defect depth (HDD) reductions of 71.7 ± 34.3%, 81.7 ±. 33.7% and 55.0 ± 28.4% were observed for BG, BG+M and C groups respectively with no significant differences between groups. Horizontal resorption was significantly greater in the C group (48.3% ± 9.48) when compared to BG (15.8 ± 16.9%) and BG+M (20.0 ± 21.9%) groups (p=0.000). Ten sites (33.3%) showed recession of the marginal mucosa after 6 months. Recession was significantly associated with buccally positioned implants (p=0.032) which had an initial HDD of 1.1 ± 0.30 mm compared to 2.3 ± 0.55 mm for implants that were lingually placed (p=0.000). In 19 out of 30 patients available for follow-up (mean post-placement period of 4.0 ± 0.65 years), the marginal soft tissues and radiographic bone levels remained stable. Conclusion: The use of BioOssTM significantly reduced the horizontal resorption of the bone. Immediate implant placement carries a risk of marginal mucosal recession and adverse esthetic outcomes. However the marginal mucosa and bone remains stable after restoration. To reduce the risk of tissue recession, implants should not be placed too buccally in maxillary anterior sockets and should be placed to maintain an HDD of at least 2mm to the implant shoulder.

Division: IADR General Session
Meeting: 2006 IADR General Session (Brisbane, Australia)
Location: Brisbane, Australia
Year: 2006
Final Presentation ID: 2574

Authors

- Darby, Ivan B. (University of Melbourne, Melbourne, N/A, Australia)
- Chen, Stephen (University of Melbourne, Melbourne, N/A, Australia)

Darby, Ivan B. (University of Melbourne, Melbourne, N/A, Australia)
Chen, Stephen (University of Melbourne, Melbourne, N/A, Australia)

SESSION INFORMATION
Oral Session
Implant Prosthodontics & Materials
07/01/2006

Clinical outcomes and esthetic results of non-submerged immediate impl[illegible]

CLINICAL ORAL IMPLANTS RESEARCH WILEY

Full Access

A prospective clinical study of non-submerged immediate implants: clinical outcomes and esthetic results

Stephen T. Chen, Ivan B. Darby, Eric C. Reynolds

First published: 30 June 2007 | https://doi.org/10.1111/j.1600-0501.2007.01388.x | Cited by: 155

Correspondence to:
Dr S. T. Chen
c/o Ms K Fletcher
Centre for Oral Health Science
School of Dental Science
The University of Melbourne
720 Swanston Street
Melbourne, Vic., 3010
Australia
Tel.: +61 3 9341 1547
Fax:+61 3 9341 1596
e-mail: schen@mira.net

Abstract

Objectives: To evaluate healing of marginal defects in immediate transmucosal implants grafted with anorganic bovine bone, and to assess mucosal and radiographic outcomes 3–4 years following restoration.

Material and methods: Thirty immediate transmucosal implants in maxillary anterior extraction sites of 30 patients randomly received BioOss™ (*N*=10; BG), BioOss™ and resorbable collagen membrane (*N*=10; BG+M) or no graft (*N*=10; control).

Results: Vertical defect height (VDH) reductions of 81.2±5%, 70.5±17.4% and 68.2±16.6%, and horizontal defect depth (HDD) reductions of 71.7±34.3%, 81.7±33.7% and 55±28.4% were observed for BG, BG+M and control groups, respectively, with no significant inter-group differences. Horizontal resorption was significantly greater in control group (48.3±9.5%) when compared with BG (15.8±16.9%) and BG+M (20±21.9%) groups (*P*=0.000). Ten sites (33.3%) exhibited recession of the mucosa after 6 months; eight (26.7%) had an unsatisfactory esthetic result post-restoration due to recession. Mucosal recession was significantly associated (*P*=0.032) with buccally positioned implants (HDD 1.1±0.3 mm) when compared with lingually positioned implants (HDD 2.3±0.6 mm). In 19 patients followed for a mean of 4.0±0.7 years, marginal mucosa and bone levels remained stable following restoration.

Conclusion: BioOss™ significantly reduced horizontal resorption of buccal bone. There is a risk of mucosal recession and adverse soft tissue esthetics with immediate implant placement. However, this risk may be reduced by avoiding a buccal position of the implant in the extraction socket.

Clinical Evaluation of CAD/CAM-Generated Composite Inlays: Six-Year Report

Clinical Evaluation of CAD/CAM-Generated Composite Inlays: Six-Year Report

Objective: Porcelain materials have demonstrated clinical reliability with a direct placement CAD/CAM technique. The purpose of this study is to evaluate the clinical performance after six years of a composite and a porcelain material for CAD/CAM-generated, adhesive inlays.
Methods: Two trained clinicians placed 40 porcelain (P) (Vita Mark II/Vita) and 40 composite (C) (Paradigm MZ100/3M-ESPE) CAD/CAM inlays, in 43 patients. A CAD/CAM unit (CEREC 2/Sirona) was used to fabricate all restorations using COS 1.21 software. Following computergraphic design of the inlay, a prefabricated block was randomly assigned for each restoration. Both restorative materials were cemented with a total etch technique using Single Bond (3M-ESPE) and dual cured resin cement (RelyX-ARC/3M-ESPE). Restorations were evaluated by two examiners using modified USPHS criteria.

Results: At six years, there was no significant difference in margin adaptation or margin discoloration between the two materials, but the composite showed a significantly better color match (Chi-Square, $p < 0.05$). The porcelain inlays had a significant increase in margin detectability from baseline at 1, 2, 3 and 6 years and the composite inlays at 2, 3, and 6 years (Wilcoxon SRT, $p < 0.05$). Both materials showed a significant increase in margin discoloration at 6 years (WSRT, $p < 0.05$). The composite resin inlays showed no significant difference in color match at any recall period, while the porcelain inlays had a significant decrease in color match at 6 months (WSRT, $p < 0.05$), with no significant color change between 6 months and 6 years. Ratings for anatomic form, surface finish and recurrent caries were > 95% alpha for both materials at all recalls.

Conclusions: The composite inlays performed equally as well as the porcelain inlays after 6 years in all categories with significantly better color match and less bulk inlay fracture.

This study was supported by 3M-ESPE.

Division: IADR General Session
Meeting: 2006 IADR General Session (Brisbane, Australia)
Location: Brisbane, Australia
Year: 2006
Final Presentation ID: 543

Authors

- Fasbinder, Dennis J. (University of Michigan, Ann Arbor, MI, USA)
- Dennison, Joseph B. (University of Michigan, Ann Arbor, MI, USA)
- Heys, Donald R. (University of Michigan, Ann Arbor, MI, USA)
- Lampe, Katherin (University of Zurich Dental School, Zurich, N/A, Switzerland)

Fasbinder, Dennis J. (University of Michigan, Ann Arbor, MI, USA)
Dennison, Joseph B. (University of Michigan, Ann Arbor, MI, USA)
Heys, Donald R. (University of Michigan, Ann Arbor, MI, USA)
Lampe, Katherin (University of Zurich Dental School, Zurich, N/A, Switzerland)

SESSION INFORMATION
Oral Session
Keynote Address and Clinical Trials
06/29/2006

Clinical Evaluation of CAD/CAM-Generated Composite Inlays: Six-Year Report

Clinical Performance of CAD/CAM-Generated Composite Inlays After 10 Years

来自ResearchGate

收藏 引用 批量引用 报错 分享

作者 DJ Fasbinder

摘要 A number of porcelain materials have demonstrated clinical reliability with a chairside placement CAD/CAM technique (e.g., CEREC). The purpose of this clinical study was to evaluate the longitudinal clinical performance of a composite resin material (Paradigm) compared to a porcelain material (Vita Mark II) for chairside CAD/CAM-generated adhesive inlays. The inlays were evaluated at six months, one year, two years, three years, six years, and 10 years. Composite resin CAD/CAM inlays performed equally as well as porcelain CAD/CAM inlays after 10 years of clinical service, with clinical advantages noted favoring composite inlays for fracture resistance and better color match to the tooth.

收起

出版源 《Current Opinion in Cosmetic Dentistry》, 2013 , 28 (4) :134

被引量 4

Levobupivicaine for postoperative pain after iliac bone harvesting for

Levobupivicaine for postoperative pain after iliac bone harvesting for implantology

Objectives: The aim of our study was to investigate whether the postoperative experience of patients following iliac bone harvesting could be improved by reducing the pain experienced using continuous local anaesthetic wound infusion with levobupivicaine.
Methods: Patients scheduled for iliac bone harvesting for the reconstruction of the mandible or maxilla under general anaesthesia were randomised to receive levobupivicaine 2.5mg/ml (provided by Abbott Laboratories) or saline started at wound closure and continued for 24h at a rate of 6.5ml/hour (equivalent to 390mg/24h levobupivicaine).

Pain intensity (Visual Analogue Scale, 0=no pain, 10=worst pain imaginable) was recorded preoperatively and hourly postoperatively for 6 hours, then twice daily until discharge and at one week. Additionally, gait disturbance was recorded whilst an inpatient and then at the clinic appointment one week later. The research nurse undertaking outcome measures was blind to the study group.

Results: 46 ASA I or II patients were eligible for inclusion in the study. VAS pain scores were significantly reduced in the levobupivicaine group ($p<0.05$). Gait disturbance was greater in the saline placebo group ($p<0.05$). Four placebo group and no levobupivicaine group patients had there discharge extended beyond the usual two nights because of poor pain control.

Conclusion: The 24h wound infusion of levobupivicaine provided statistically and clinically significant reduction in postoperative pain after iliac bone harvesting for oral and maxillofacial surgery under general anaesthesia. Although, local infusions have been advocated as a safe and effective method of reducing pain from the graft donor site, the efficacy has not previously been studied in a controlled fashion. Our recommendation is that would infiltration with local anaesthetic (levobupivicaine 2.5mg/ml) should be used in clinical practice.

Division: IADR General Session
Meeting: 2006 IADR General Session (Brisbane, Australia)
Location: Brisbane, Australia
Year: 2006
Final Presentation ID: 1775

Coulthard, Paul (The University of Manchester, Manchester, N/A, United Kingdom)
Jackson-leech, Debbie (Central Manchester and Manchester Childrens University Hospitals, Manchester, N/A, United Kingdom)
Oliver, Richard John (The University of Manchester, Manchester, N/A, United Kingdom)

Authors

- Coulthard, Paul (The University of Manchester, Manchester, N/A, United Kingdom)
- Jackson-leech, Debbie (Central Manchester and Manchester Childrens University Hospitals, Manchester, N/A, United Kingdom)
- Oliver, Richard John (The University of Manchester, Manchester, N/A, United Kingdom)

SESSION INFORMATION
Poster Session
Dental Anesthesiology Research
06/30/2006

Levobupivicaine for postoperative pain after iliac bone harvesting for implantology

Int J Surg. 2008 Feb;6(1):57-63. Epub 2007 Jul 24.

The efficacy of local anaesthetic for pain after iliac bone harvesting: a randomised controlled trial.

Coulthard P[1], Oliver R, Khan Afridi KA, Jackson-Leech D, Adamson L, Worthington H.

Author information

1 Department of Oral and Maxillofacial Surgery, School of Dentistry, The University of Manchester, Higher Cambridge Street, Manchester, M15 6FH, UK. paul.coulthard@manchester.ac.uk

Abstract

INTRODUCTION: Autogenous bone grafting is commonly used in reconstructive surgery but postoperative pain from the donor site can be severe, delaying early mobilisation and preventing discharge from hospital.

METHOD: An RCT of levobupivacaine infusion (16.25mg/h for 24h) of iliac crest wounds versus placebo. Postoperative pain was recorded immediately on returning to the ward, then at 1, 2, 3, 4, 5, 6h, morning and evening on subsequent days until discharge, and at the 7-day clinic appointment. Mobility was recorded twice daily and at 7days.

RESULTS: Of 46 evaluable patients, 25 were randomised to levobupivacaine and 21 to placebo. Mean pain scores for (i) average pain from initial assessment to 6h; (ii) 1day in the morning; (iii) 1day in the evening; (iv) at 2days; and (v) follow-up were all statistically significant in favour of lower pain scores in the levobupivicaine group (p-values all <0.01). Comparison between the study groups for mobility found 6 patients unable to get out of bed in the placebo group and none in the local anaesthetic group at the initial assessment (Fisher's exact test p-value=0.005), and 2 patients at 24h. Patients in the local anaesthetic group were always more mobile and this was statistically significant even at 7days for gait disturbance, limp, deviation of gait and unequalness of stride. There were no complications relating to the infusion system.

CONCLUSIONS: Local anaesthetic significantly reduced postoperative pain and improved mobility. We recommend that surgeons use a local anaesthetic infusion to improve the postoperative experience for their patients undergoing iliac crest grafting.

PMID: 17869596 DOI: 10.1016/j.ijsu.2007.07.002

2007

3-Year Clinical Evaluation of an All-In-One Self-Etching Dental Adhesive

3-Year Clinical Evaluation of an All-In-One Self-Etching Dental Adhesive

Objective: The purpose of this randomized clinical trial was to evaluate the clinical performance of an all-in-one self-etching dental adhesive (iBond, Heraeus Kulzer) versus that of a multi-step total-etch dental adhesive (Gluma Solid Bond, Heraeus Kulzer) when used to restore non-carious cervical lesions. Methods: Lesions were characterized preoperatively relative to height, width, depth, percent of margin in enamel, internal angle, and degree of sclerosis. Fifty-five non-carious cervical lesions were randomly assigned to two treatment groups according to the adhesive used: iBond (n=28) or Gluma Solid Bond (n=27). The exposed walls of the lesion were roughened with a diamond instrument. No retentive grooves or bevels were used. Lesions were restored with Durafill VS (Heraeus Kulzer). Adhesives and composite were applied according to manufacturer's directions and light-cured using Translux Energy (Heraeus Kulzer). The restorations were evaluated at baseline, 6 and 18 months, and 3 years post-insertion for retention, secondary caries, marginal adaptation/integrity, and marginal discoloration using modified USPHS criteria for clinical evaluation of dental restorations. Data were analyzed using Fisher's Exact Test (p=0.05) for significant differences between treatments. Results: Overall lesion characteristics were similar for both treatment groups, and all baseline scores were alfa for restorations in both treatment groups. The 6-month, 18-month and 3-year recall rates were 100%, 95% and 86%, respectively. No retention failures or secondary caries were observed at any of the evaluation visits. No significant differences were detected between iBond and Gluma Solid Bond regarding marginal adaptation/integrity (p=0.09). iBond had significantly more marginal discoloration than Gluma Solid Bond at 18 months and 3 years (p=0.003). Conclusions: The all-in-one self-etching dental adhesive had a higher incidence of marginal discoloration than the total-etch dental adhesive at 18 months and 3 years post-insertion. No clinical failures were observed as determined by modified USPHS criteria. Supported by Heraeus Kulzer. rittera@dentistry.unc.edu

Division: IADR/AADR/CADR General Session
Meeting: 2007 IADR/AADR/CADR General Session (New Orleans, Louisiana)
Location: New Orleans, Louisiana
Year: 2007
Final Presentation ID: 899

Authors

- Ritter, Andre V. (University of North Carolina, Chapel Hill, NC, USA)
- Heymann, Harald (University of North Carolina, Chapel Hill, NC, USA)
- Pereira, Patricia (University of North Carolina, Chapel Hill, NC, USA)
- Sturdevant, John (University of North Carolina, Chapel Hill, NC, USA)
- Swift, Edward J. (University of North Carolina, Chapel Hill, NC, USA)
- Wilder, Al (University of North Carolina, Chapel Hill, NC, USA)

SESSION INFORMATION
Poster Session
Clinical Trials: Dentin/Enamel Adhesives
03/22/2007

Ritter, Andre V. (University of North Carolina, Chapel Hill, NC, USA)
Heymann, Harald (University of North Carolina, Chapel Hill, NC, USA)
Pereira, Patricia (University of North Carolina, Chapel Hill, NC, USA)
Sturdevant, John (University of North Carolina, Chapel Hill, NC, USA)
Swift, Edward J. (University of North Carolina, Chapel Hill, NC, USA)
Wilder, Al (University of North Carolina, Chapel Hill, NC, USA)

3-Year Clinical Evaluation of an All-In-One Self-Etching Dental Adhesive

OPERATIVE DENTISTRY

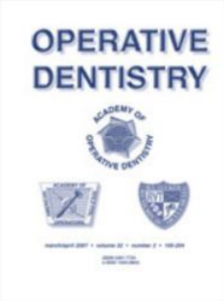

Article Citation:
A. V. Ritter, H. O. Heymann, E. J. Swift Jr, J. R. Sturdevant, and A. D. Wilder Jr (*2008*) Clinical Evaluation of an All-in-one Adhesive in Non-Carious Cervical Lesions with Different Degrees of Dentin Sclerosis. Operative Dentistry: July 2008, Vol. 33, No. 4, pp. 370-378.

https://doi.org/10.2341/07-128

Clinical Research

Clinical Evaluation of an All-in-one Adhesive in Non-Carious Cervical Lesions with Different Degrees of Dentin Sclerosis

A. V. Ritter*, H. O. Heymann, E. J. Swift Jr, J. R. Sturdevant, and A. D. Wilder Jr

SUMMARY

This randomized clinical trial compared the performance of an all-in-one adhesive (iBond) applied in sclerotic and non-sclerotic non-carious cervical lesions with that of a three-step etch-prime-bond adhesive (Gluma Solid Bond, SB). One-hundred and five lesions were randomly assigned to four groups according to adhesive, sclerosis scale and technique: 1) SB applied to lesions with sclerosis scale 1 and 2 (n=26); 2) iBond applied to lesions with sclerosis scale 1 and 2 (n=28); 3) iBond applied to lesions with sclerosis scale 3 and 4 (n=25) and 4) iBond applied with prior acid-etching to lesions with sclerosis scale 3 and 4 (n=26). A microfilled composite (Durafill VS) was used as the restorative material. The restorations were evaluated for retention, color match, marginal adaptation, anatomic form, cavosurface margin discoloration, secondary caries, pre- and post-operative sensitivity, surface texture and fracture at insertion (baseline), 6, 18 months and at 3 years using modified USPHS evaluation criteria (Alfa=excellent; Bravo=clinically acceptable; Charlie=clinically unacceptable). There was a high percentage of Bravo scores for marginal adaptation (4%–32%) and marginal discoloration (18%–60%) in Groups 2, 3 and 4, but all groups had <5% Charlie scores at 6 months and <10% Charlie scores at 18 months for retention and marginal discoloration, respectively. However, it should be noted that 13% of the restorations in Group 4 were not retained at three years.

Clinical Outcomes of Immediately Loaded Implant-Supported Mandibular Fixed Prostheses

Objectives: To test the null hypothesis that a procedure whereupon dental implants in the edentulous mandible are immediately loaded with a denture has no benefits compared to a traditional loading procedure in terms of implant survival, clinical function, short-term prognosis and patient quality of life. Methods: A parallel randomized controlled trial with 2 study arms was approved by the University of Toronto ethical committee and initiated in 2006. Forty participants are being recruited amongst patients seeking implant-supported prosthetics treatment at the Implant Prosthodontic Unit (IPU), University of Toronto. Four TiUnite dental implants (NobelBiocare®, Gothenburg, Sweden) are placed following the standard surgical protocol that has been used for more than two decades at IPU. Immediately after surgery, the allocation code, provided by a third independent part that conducted the randomization, was opened to determine which arm the patient should enter. In the experimental group, the denture was modified into a fixed bridge on the same day of the surgery. The permanent FPD was fabricated 2 weeks later. In the control group, the implants remained submerged for 3 months. The clinical evaluations were done by an independent blinded investigator. Further outcomes will be measures of quality of life and patient mediated cost effectiveness analysis of treatment. Results: Early clinical results show minor difference in prevalence of osseointegration and patient satisfaction between the two groups. Implant survival is slightly higher in the control group. However, the immediate loading group reported better quality of life. Both groups reported more satisfaction with implant treatment compared to conventional complete dentures. Conclusion: Implant survival, clinical function, short-term prognosis and quality of life profile of the fixed prostheses in the mandibles are comparable in patients treated with immediate loading or conventional loading techniques. Acknowledgment: This study has been funded by NobelBiocare®, Gothenburg, Sweden.

Division: IADR/AADR/CADR General Session
Meeting: 2007 IADR/AADR/CADR General Session (New Orleans, Louisiana)
Location: New Orleans, Louisiana
Year: 2007
Final Presentation ID: 53

Authors

- Alfadda, Sara (University of Toronto, Toronto, ON, Canada)
- Chvartszaid, David (University of Toronto, Toronto, ON, Canada)
- Preston, Kirk (University of Toronto, Toronto, ON, Canada)
- David, Lesley (University of Toronto, Toronto, ON, Canada)
- Jokstad, Asbjorn (University of Toronto, Toronto, ON, Canada)

Alfadda, Sara (University of Toronto, Toronto, ON, Canada)
Chvartszaid, David (University of Toronto, Toronto, ON, Canada)
Preston, Kirk (University of Toronto, Toronto, ON, Canada)
David, Lesley (University of Toronto, Toronto, ON, Canada)
Jokstad, Asbjorn (University of Toronto, Toronto, ON, Canada)

SESSION INFORMATION
Oral Session
Implant Survival Studies: Clinical Outcomes
03/21/2007

Clin Implant Dent Relat Res. 2014 Dec;16(6):806-16. doi: 10.1111/cid.12057. Epub 2013 Mar 18.

A randomized controlled clinical trial of edentulous patients treated with immediately loaded implant-supported mandibular fixed prostheses.

Alfadda SA[1].

Author information

Abstract

PURPOSE: A 1-year blinded two-arm parallel randomized controlled clinical trial was conducted to test the null hypothesis that immediate loading of four dental implants between the mental foramina with a fixed prosthesis has no benefits compared with the conventional loading technique in terms of implant success and clinical function.

MATERIALS AND METHODS: Forty-five patients, completely edentulous in the mandibles seeking implant-supported prostheses at the Faculty of Dentistry, University of Toronto, were recruited. Four TiUnite dental implants (NobelBiocare®, Göteborg, Sweden) were placed following the one-stage surgical protocol. Immediately after surgery, the patients were randomly assigned to either study arms by a third independent party. In the experimental arm (EA), existing mandibular denture was converted into an interim implant-supported fixed bridge (ISFB) on the same day of surgery. In the control arm (CA), the mandibular denture was hollowed out and relined with a soft tissue reline. The implants were loaded with the permanent ISFB at least 3 months postsurgery. Patients were assessed by a calibrated independent investigator at 2, 6, and 12 months following completion of treatment.

RESULTS: A total of one hundred sixty implants were placed. Due to anatomical limitations, one patient was excluded from the study. Four patients in the EA did not receive intervention as allocated and were transferred to the CA. Implant success rate was comparable between the two arms and exceeded 96%. Marginal bone loss was statistically significantly more in the immediate loading arm, -0.296 mm versus -0.037 mm (intention to treat: p = .002; per protocol: p = .021). The relatively early intervention and insertion of the final prosthesis in the immediate arm, when bone healing and remodeling process had not yet been completed, might explain the difference in the amount of bone loss.

CONCLUSION: Immediate loading of four dental implants with a fixed prosthesis in the edentulous mandible is a feasible treatment option and leads to a substantial improvement in perceived oral health status.

© 2013 Wiley Periodicals, Inc.

KEYWORDS: edentulous mandible; fixed implant prosthesis; immediate loading; implant; randomized controlled trial

PMID: 23506407 DOI: 10.1111/cid.12057

Color match of crowns with zirconia and metal

Color match of crowns with zirconia and metal frameworks

Objectives: The purpose of this study was to analyze the color match of veneered crowns with three different framework materials.
Methods: Six patients each with one central maxillary incisor to be restored and the other one unrestored (control) were included. For each patient one metal (M), one glass-ceramic (G) and two zirconia (Zi) frameworks were made. The veneering was performed by 4 dental technicians in a standardized manner. The following veneering ceramics were included and masked to eliminate bias: A: Creation Classic, Klema; B: IPS e.max Ceram (with zirconia frameworks), Ivoclar; C: Creation ZI, Klema; D: IPS e.max Ceram (with glass-ceramic fameworks). The 24 reconstructions were randomly distributed to the technicians. The color of the crowns and the control teeth was captured using spectrophotometric analysis (SpectroShade, Metalor) and the color difference ΔE was calculated (objective assessment). Furthermore, reconstructions were compared to control teeth and rated best to worst by 11 unbiased observers (subjective assessment). Statistical analysis was performed with a Student's paired t-test, Chi-Square-test and correlation coefficient.

Results: Spectrophotometric analysis revealed a visible color deviation between test crowns and control teeth without statistical significance (ΔE MA 3.9±0.8, ΔE GD 5.0±1.1, ΔE ZiB 4.2±1.1, ΔE ZiC 5.2±1.6). In contrast, the subjective assessment showed a significantly higher preference for crowns with zirconia frameworks ($p<0.01$, Chi Square). Out of all combinations, zirconia frameworks veneered with B were ranked best match in the majority of judgings (MA 25.8%, GD 22.7%, ZiB 38%, ZiC 13.6%, $p<0.01$, Chi Square). Glass-ceramic crowns veneered with the same ceramic, however, were only best match in 22.7% (n=15). Metal-ceramic crowns were judged to be worst in 33.3% (n=22, $p<0.01$, Chi Square). No correlation was found between objective and subjective analysis.

Conclusion: Veneered zirconia frameworks exhibited a comparable or better color match as veneered glass-ceramic and metal frameworks.

Division: IADR/AADR/CADR General Session
Meeting: 2007 IADR/AADR/CADR General Session (New Orleans, Louisiana)
Location: New Orleans, Louisiana
Year: 2007
Final Presentation ID: 291

Authors

- Sailer, Irena (University of Zurich, Zurich, N/A, Switzerland)
- Holderegger, Claudia (University of Zurich, Zurich, N/A, Switzerland)
- Jung, Ronald Ernst (University of Zurich, Zurich, N/A, Switzerland)
- Zembic, Anja (University of Zurich, Zurich, N/A, Switzerland)
- Hammerle, Christoph Hans Franz (University of Zurich, Zurich, N/A, Switzerland)

Sailer, Irena (University of Zurich, Zurich, N/A, Switzerland)
Holderegger, Claudia (University of Zurich, Zurich, N/A, Switzerland)
Jung, Ronald Ernst (University of Zurich, Zurich, N/A, Switzerland)
Zembic, Anja (University of Zurich, Zurich, N/A, Switzerland)
Hammerle, Christoph Hans Franz (University of Zurich, Zurich, N/A, Switzerland)

SESSION INFORMATION
Oral Session
Crowns and Fixed Partial Dentures
03/22/2007

Color match of crowns with zirconia and metal

Int J Prosthodont. 2007 May-Jun;20(3):263-9.

Clinical study of the color stability of veneering ceramics for zirconia frameworks.

Sailer I[1], Holderegger C, Jung RE, Suter A, Thiévent B, Pietrobon N, Gebhard-Achilles W, Hämmerle CH.

Author information

1 Clinic for Fixed and Removable Prosthodontics and Dental Materials, School of Dentistry, University of Zürich, Plattenstr 11, 8032 Zürich, Switzerland. irena.sailer@zzmk.unizh.ch

Abstract

PURPOSE: The purpose of this study was to compare 3 veneering ceramics for zirconia frameworks regarding color stability and predictability of the esthetic result.

MATERIALS AND METHODS: Six patients with 1 maxillary central incisor to be restored were enrolled in the study. The contralateral incisor had to be nonrestored and vital to serve as a reference tooth. For each patient, 4 single crowns with zirconia frameworks were fabricated. Three veneering ceramics were assessed and masked to eliminate bias. Choice of the veneering ceramics was done at random. The veneering was performed by 4 dental technicians. Three veneering ceramics were compared: ceramic A (Initial, GC), ceramic B (Triceram, Espridént), and ceramic C (Cercon Ceram S, DeguDent). The color of the crowns and reference teeth was captured using spectrophotometric analysis (SpectroShade, MHT), and the color difference (deltaE) was calculated (objective method). In addition, the crowns and reference teeth were compared subjectively by 11 observers blind to the ceramic used for veneering. Statistical analysis was performed with analysis of variance (ANOVA).

RESULTS: Regardless of the veneering ceramic used, all crowns showed a high color deviation from the reference teeth when applying the objective analysis (deltaE(A) 6.8 +/- 2.5, deltaE(B) 5.6 +/- 1.2, deltaE(C) 5.7 +/- 2.1). In addition, no significant differences were found between the deltaE of crowns and teeth for the 3 ceramics. In the framework-supported area, ceramic B showed a significantly lower difference in value (deltaL) compared to the reference teeth than the other 2 ceramics (deltaL(A) 4.9 +/- 2.3, deltaL(B) 1.1 +/- 2.1, deltaL(C) 4.1 +/- 1.5; P < .01 ANOVA). When performing the subjective analysis, ceramic B was chosen as the best match by a majority of observers (> 60%) in 4 of 6 patients.

CONCLUSIONS: All 3 ceramics met the esthetic demands only to a limited extent. Ceramic B allowed for the most predictable result in terms of color stability.

PMID: 17580458

Detecting erosions in temporomandibular joint RA by a cone-

Detecting erosions in temporomandibular joint RA by a cone-beam CT

Objectives: To investigate the reproducibility of detection of bone tissue erosive changes in the temporomandibular joint (TMJ) by a cone-beam computer tomography (CBCT) using a new scoring system as well as their association with TMJ pain, c-reactive protein (CRP) and plasma levels of the NMDA receptor agonist glutamate (Glu).
Methods: Forty-eight patients with recent diagnosis of RA was included. Presence or absence of erosive changes was recorded in six regions of the temporal part and condyle of each TMJ using CBCT images. The radiographic score for each patient could range from 0 to 24. Ten joints were randomly selected for assessment of reproducibility of readings. The patients were examined for TMJ pain intensity, which was assessed on a numeric rating scale (0-100). Venous blood was analyzed for CRP to assess systemic inflammatory activity as well as for Glu. Nonparametric statistical methods were used.

Results: The relative reproducibility of detection of erosive changes varied between 70 and 100% for the different areas where the medial part of the anterior frontal section of the condyle showed the lowest degree of reproducibility and regions on the temporal part the highest. A total of 71% of the patients had erosive changes in the TMJ and 26% of these patients had TMJ pain. Presence of erosive changes in the TMJ was neither significantly associated with CRP (n=40), nor plasma level of Glu (n=46).

Conclusions: This study shows that detection of bony erosions in the TMJ using CBCT and our scoring system has acceptable reproducibility. Further that TMJ erosive changes are frequent in early RA but seemingly not related to TMJ pain nor CRP or Glu in blood. This suggests a difference in the modulation of pain and bone tissue destruction in the TMJ.

Supported by NIDCR grant De15420

Division: IADR/AADR/CADR General Session
Meeting: 2007 IADR/AADR/CADR General Session (New Orleans, Louisiana)
Location: New Orleans, Louisiana
Year: 2007
Final Presentation ID: 260

Authors

- Hajati, Anna-kari (Karolinska Institutet, Huddinge, N/A, Sweden)
- Nasstrom, Karin (Karolinska Institutet, Huddinge, N/A, Sweden)
- Alstergren, Per (Karolinska Institutet, Huddinge, N/A, Sweden)
- Bratt, Johan (Karolinska Institutet, Stockholm, N/A, Sweden)
- Kopp, Sigvard (Karolinska Institutet, Huddinge, N/A, Sweden)

Hajati, Anna-kari (Karolinska Institutet, Huddinge, N/A, Sweden)
Nasstrom, Karin (Karolinska Institutet, Huddinge, N/A, Sweden)
Alstergren, Per (Karolinska Institutet, Huddinge, N/A, Sweden)
Bratt, Johan (Karolinska Institutet, Stockholm, N/A, Sweden)
Kopp, Sigvard (Karolinska Institutet, Huddinge, N/A, Sweden)

SESSION INFORMATION
Oral Session
Diagnosis and genetics of TMD/orofacial pain
03/22/2007

Detecting erosions in temporomandibular joint RA by a cone-beam CT

J Oral Maxillofac Surg. 2009 Sep;67(9):1895-903. doi: 10.1016/j.joms.2009.04.056.

Endogenous glutamate in association with inflammatory and hormonal factors modulates bone tissue resorption of the temporomandibular joint in patients with early rheumatoid arthritis.

Hajati AK[1], Alstergren P, Näsström K, Bratt J, Kopp S.

⊕ **Author information**

Abstract

PURPOSE: The aim of this study was to investigate the relation between plasma level of glutamate and extent of radiographic bone erosion of the temporomandibular joint (TMJ) in patients with early rheumatoid arthritis (RA) in relation to inflammatory disease activity as well as estradiol and testosterone.

MATERIALS AND METHODS: A total of 47 patients (29 women and 18 men) of whom 24 were seropositive were included shortly after being diagnosed with RA. Radiographic signs of bone tissue resorption (erosions) in the TMJ were recorded by cone-beam CT images, and an erosion score (0 to 24) was calculated for each patient. Venous blood was analyzed for rheumatoid factor, C-reactive protein, erythrocyte sedimentation rate, leukocyte particle count, glutamate, estradiol, and testosterone. Nonparametric statistical methods were used in the analysis.

RESULTS: Resorptive changes of the TMJ were found in a major part of the patients. There was a significant positive correlation between plasma level of glutamate and extension of radiographic erosions that was strongest in the patients with low levels of C-reactive protein, estradiol, or testosterone. By contrast, erosions were correlated with C-reactive protein in patients with high levels of estradiol. The highest levels of glutamate were found in patients with low levels of C-reactive protein and estradiol.

CONCLUSIONS: This study shows that a majority of patients with early RA presents radiographic signs of bone tissue resorption of the TMJ and that circulating glutamate is associated with the extent of these changes. The relationship between glutamate and bone resorption seems to be influenced by systemic inflammatory activity as well as estradiol and testosterone levels.

PMID: 19686927 PMCID: PMC2771262 DOI: 10.1016/j.joms.2009.04.056

Duplicating the number of adhesive coats: an 18-month clinical trial

Duplicating the number of adhesive coats: an 18-month clinical trial

Objectives: A 18-month randomized, controlled prospective study evaluated, in an intra-individual comparison, the clinical performance of a self-etch and an etch-and-rinse adhesive in non-carious cervical lesions applied as recommended and after duplicating the number of adhesive coats. Methods: Twenty-nine patients, with at least two pairs of similar sized non-carious cervical lesions participated in this study. One hundred sixteen restorations were placed, according to one of the following groups: OS2: phosphoric acid + 2 coats of One Step Plus; OS4: phosphoric acid + 4 coats of One Step Plus; TY2: Tyrian SPE + 2 coats of One Step Plus and TY4: Tyrian SPE + 4 coats of One Step Plus. The restorations were evaluated at baseline and after 18 months according to the modified USPHS criteria. Statistical analysis was made with Friedman repeated measures analysis of variance by rank and Wilcoxon sign-ranked test for significance at each pair (alfa=0.05). Results: One hundred eight restorations were evaluated at 18 months. The retention rate of the group TY2 after 18 month (55.5%) was statistically lower than groups OS2 (70.4%), OS4 (88.9%) and TY4 (77.8%). Marginal discoloration occurred in all groups, being statistically worse in group TY2, which was scored as beta in 26.9% of the cases. Conclusions: Tyrian SPE + One Step Plus applied according manufacturer's recommendations showed a very low retention rate after 18 months. The use of four coats of the One Step Plus in the self-etch approach can improve its clinical performance.

Division: IADR/AADR/CADR General Session
Meeting: 2007 IADR/AADR/CADR General Session (New Orleans, Louisiana)
Location: New Orleans, Louisiana
Year: 2007
Final Presentation ID: 227

Authors
- Reis, Alessandra (University of Oeste de Santa Catarina, Joaçaba - SC, N/A, Brazil)
- Loguercio, Alessandro Dourado (University of Oeste de Santa Catarina, Joaçaba - SC, N/A, Brazil)
- Bauer, José Roberto Oliveira (Universidade Bandeirantes, São Paulo - SP, N/A, Brazil)

Reis, Alessandra (University of Oeste de Santa Catarina, Joaçaba - SC, N/A, Brazil)
Loguercio, Alessandro Dourado (University of Oeste de Santa Catarina, Joaçaba - SC, N/A, Brazil)
Bauer, José Roberto Oliveira (Universidade Bandeirantes, São Paulo - SP, N/A, Brazil)

SESSION INFORMATION
Oral Session
Keynote Address and Clinical Trials: New Resin Composites and Adhesives
03/22/2007

Duplicating the number of adhesive coats: an 18-month clinical trial

J Am Dent Assoc. 2008 Jan;139(1):53-61.

Application of a dental adhesive using the self-etch and etch-and-rinse approaches: an 18-month clinical evaluation.

Loguercio AD[1], Reis A.

Author information

1 Department of Dental Materials and Operative Dentistry, Universidade do Oeste de Santa Catarina, Joaçaba, Brazil. aloguercio@hotmail.com

Abstract

BACKGROUND: Laboratory investigations have demonstrated that the application of multiple adhesive coats can increase adhesive infiltration, thereby increasing bond strength values. The authors conducted an 18-month, randomized, controlled prospective study to evaluate the clinical performance of a self-etch and an etch-and-rinse adhesive in noncarious cervical lesions.

METHODS: Twenty-nine patients with at least two pairs of similarly sized lesions participated in this study. The authors placed 116 restorations in one of four groups: OS2 (phosphoric acid and dental adhesive [One-Step Plus, Bisco, Schaumburg, Ill.], following the manufacturer's recommendation [two coats]); OS4 (phosphoric acid and One-Step Plus, with four coats); TY2 (Tyrian SPE [Bisco] and One-Step Plus, following the manufacturer's recommendation [two coats]); and TY4 (Tyrian SPE and One-Step Plus, with four coats). The authors evaluated the restorations at baseline and at six, 12 and 18 months, according to modified U.S. Public Health Service criteria. (Eight of the 116 restorations were unavailable for follow-up.)

RESULTS: The retention rate for the TY2 group (55.5 percent) was statistically lower than that for the TY4 (77.8 percent) and OS4 (88.9 percent) groups. Only teeth in OS4 exhibited a retention rate at 18 months that was similar to that observed at baseline. Marginal discoloration occurred in all groups, and it was statistically significantly worse in TY2.

CONCLUSIONS: Multiple adhesive coats significantly improved retention rates.

CLINICAL IMPLICATIONS: Applying multiple coats of adhesive with the etch-and-rinse or self-etch approach can improve retention rates of Class V resin-based composite restorations, although not to the level of the American Dental Association's guidelines for dentin and adhesive materials.

PMID: 18167386

Effect of Physiotherapy on Bruxism and Head Posture

Effect of Physiotherapy on Bruxism and Head Posture

Objective: The aim was to evaluate the effectiveness of a physiotherapeutic intervention to improve the head posture and reduce the signs of bruxism in a group of subjects with bruxist behaviour. Materials and Methods: A blind randomized clinical trial was performed. All the subjects were three to six year old, were healthy, had normal facial morphology and class I occlusion. The individuals were included in the study when their guardians reported bruxism, they presented high level of anxiety in the Conners' Parents Rating Scales (CPRS), two or more signs of temporomandibular disorders (TMD) according to Bernal and Tsamtsouris and dental wear visually evident. For each child, a clinical, photographic and radiographic evaluation of the head and cervical posture were realized with standardized techniques. The dental wear was drawn in dental casts, and processed in digital format. The children were randomized in an experimental (n=13) and a control (n=13) group. A physiotherapeutic intervention was applied to the children of the experimental group once a week, until 13 sessions were completed. Afterwards, the cephalogram, the clinical and photographic evaluation of the head posture and the dental wear, were measured. Also the anxiety, the TMD and the report of bruxism by the guardians were evaluated again. The data were analyzed with the t-test and the Wilcoxon rank sum test. Results: All the children presented a downward head posture and kyphotic position of the cervical spine at the beginning of the study. The subjects of the experimental group showed statistically significant improvement in the natural head posture (p value: 0.013). The anxiety and the guardian's report of bruxism reduced as well. The TMD did not reduce. Conclusion: The physiotherapeutic intervention showed to be efficient to reduce the signs of bruxism and to improve the head posture in the studied children.

Division: IADR/AADR/CADR General Session
Meeting: 2007 IADR/AADR/CADR General Session (New Orleans, Louisiana)
Location: New Orleans, Louisiana
Year: 2007
Final Presentation ID: 721

Authors

- Restrepo, Claudia C. (Instituto de Ciencias de la Salud, Medellín, N/A, Colombia)
- Quintero, Yasmy (Instituto de Ciencias de la Salud, Medellín, N/A, Colombia)
- Tamayo, Martha (Instituto de Ciencias de la Salud, Medellín, N/A, Colombia)
- Tamayo, Veronica (CINDE, Medellín, N/A, Colombia)
- Gallego, Gabriel (Instituto de Ciencias de la Salud, Medellín, N/A, Colombia)
- Velez, Ana Lucia (Instituto de Ciencias de la Salud, Medellín, N/A, Colombia)
- Pelaez-vargas, Alejandro (Instituto de Ciencias de la Salud, Medellín, N/A, Colombia)
- Alvarez, Emery (Instituto de Ciencias de la Salud, Medellín, N/A, Colombia)

Restrepo, Claudia C. (Instituto de Ciencias de la Salud, Medellín, N/A, Colombia)
Quintero, Yasmy (Instituto de Ciencias de la Salud, Medellín, N/A, Colombia)
Tamayo, Martha (Instituto de Ciencias de la Salud, Medellín, N/A, Colombia)
Tamayo, Veronica (CINDE, Medellín, N/A, Colombia)
Gallego, Gabriel (Instituto de Ciencias de la Salud, Medellín, N/A, Colombia)
Velez, Ana Lucia (Instituto de Ciencias de la Salud, Medellín, N/A, Colombia)
Pelaez-vargas, Alejandro (Instituto de Ciencias de la Salud, Medellín, N/A, Colombia)
Alvarez, Emery (Instituto de Ciencias de la Salud, Medellín, N/A, Colombia)

SESSION INFORMATION
Poster Session
TMD/orofacial pain epidemiology and treatment
03/22/2007

Effect of Physiotherapy on Bruxism and Head Posture

Journal of Oral Rehabilitation

Full Access

Effect of awareness through movement on the head posture of bruxist children

Y. QUINTERO, C.C. RESTREPO, V. TAMAYO, M. TAMAYO, A. L. VÉLEZ, G. GALLEGO, A. PE

First published: 29 December 2008 | https://doi.org/10.1111/j.1365-2842.2008.01906.x

Dr Claudia Restrepo, Carrera 38 No. 6 B Sur 25. Apartamento 1002 Medellín, Colombi
martinezrestrepo@une.net.co

All authors have made substantive contribution to this study and/or manuscript, and all t
final paper prior to its submission.

Abstract

Summary The aim of this study was to evaluate the effectiveness of physiotherapy to improve the head posture and reduce the signs of bruxism in a group of bruxist children. A single-blind randomized clinical trial was performed. All the subjects were 3- to 6-year old, had complete primary dentition, dental and skeletal class I occlusion and were classified as bruxist according to the minimal criteria of the ICSD for bruxism. For each child, a clinical, photographic and radiographic evaluation of the head and cervical posture were realized with standardized techniques. The children were randomized in an experimental ($n = 13$) and a control ($n = 13$) group. A physiotherapeutic intervention was applied to the children of the experimental group once a week, until 10 sessions were completed. Afterwards, the cephalogram and the clinical and photographic evaluation of the head posture were measured again. The data were analysed with the *t*-test and Mann–Whitney test. The subjects of the experimental group showed statistically significant improvement in the natural head posture. The physiotherapeutic intervention showed to be efficient to improve the head posture at the moment of measurement in the studied children. The relationship between bruxism and head posture, if exists, seems to be worthwhile to examine.

Effectiveness of Low-Level Laser Therapy in Treatment of Temporomandibular

Effectiveness of Low-Level Laser Therapy in Treatment of Temporomandibular

Objectives: The purpose of this study was to evaluate the effectiveness of low-level laser therapy (LLLT) in patients presenting temporomandibular disorder (TMD) in a random and placebo-controlled research design. Methods: The sample consisted of 40 patients, divided into experimental group (G1) and placebo group (G2). The treatment was done with infrared laser (830 nm, 500 mW, 20s, 4 J/point) at the painful points, once a week during four consecutive weeks. The patients were evaluated before and after the treatment through Visual Analogue Scale (VAS) and Craniomandibular Index (CMI). Results: the baseline and post-therapy values of VAS and CMI were compared by the paired T-test, separately for the placebo and laser groups. A significant difference was observed between initial and final values (p<0.05) in both groups. Baseline and post-therapy values of pain and Craniomandibular Index were compared in the therapy groups by the two-sample T-test, no significant differences were observed regarding VAS and CMI (p>0.05). Conclusion: After either placebo or laser therapy, pain and temporomandibular symptoms were significantly lower. There was no significant difference between groups, although laser therapy presented better outcomes than placebo.

Division: IADR/AADR/CADR General Session
Meeting: 2007 IADR/AADR/CADR General Session (New Orleans, Louisiana)
Location: New Orleans, Louisiana
Year: 2007
Final Presentation ID: 737

Authors

- Firoozmand, Leily Macedo (Universidade Est. Paulista Julio Mesquita, São José dos Campos, N/A, Brazil)
- Camargo, Samira Esteves Afonso (Universidade Est. Paulista Julio Mesquita, São José dos Campos, N/A, Brazil)
- Cunha, Lia Alves (Universidade Est. Paulista Julio Mesquita, Sao José dos Campos, N/A, Brazil)
- Silva, Andressa Pereira (Universidade Estadual Paulista, São Paulo, N/A, Brazil)
- Oliveira, Wagner De (Universidade Est. Paulista Julio Mesquita, São José Campos, N/A, Brazil)

Firoozmand, Leily Macedo (Universidade Est. Paulista Julio Mesquita, São José dos Campos, N/A, Brazil)
Camargo, Samira Esteves Afonso (Universidade Est. Paulista Julio Mesquita, São José dos Campos, N/A, Brazil)
Cunha, Lia Alves (Universidade Est. Paulista Julio Mesquita, Sao José dos Campos, N/A, Brazil)
Silva, Andressa Pereira (Universidade Estadual Paulista, São Paulo, N/A, Brazil)
Oliveira, Wagner De (Universidade Est. Paulista Julio Mesquita, São José Campos, N/A, Brazil)

SESSION INFORMATION
Poster Session
TMD/orofacial pain epidemiology and treatment
03/22/2007

Effectiveness of Low-Level Laser Therapy in Treatment of Temporomandibular

Int Dent J. 2008 Aug;58(4):213-7.

Efficacy of low-level laser therapy in the treatment of temporomandibular disorder.

da Cunha LA[1], Firoozmand LM, da Silva AP, Camargo SE, Oliveira W.

Author information

Erratum in

Int Dent J. 2008 Oct;58(5):230. Esteves, Samira Afonso [corrected to Camargo, Samira Esteves Afonso]..

Abstract

AIMS: To evaluate the effectiveness of low-level laser therapy (LLLT) in patients presenting with temporomandibular disorder (TMD) in a random and placebo-controlled research design.

METHODS: The sample consisted of 40 patients, divided into an experimental group (G1) and a placebo group (G2). The treatment was done with an infrared laser (830nm, 500mW, 20s, 4J/point) at the painful points, once a week for four consecutive weeks. The patients were evaluated before and after the treatment through a Visual Analogue Scale (VAS) and the Craniomandibular Index (CMI).

RESULTS: The baseline and posttherapy values of VAS and CMI were compared by the paired T-test, separately for the placebo and laser groups. A significant difference was observed between initial and final values ($p < 0.05$) in both groups. Baseline and post-therapy values of pain and CMI were compared in the therapy groups by the two-sample T-test, yet no significant differences were observed regarding VAS and CMI ($p > 0.05$).

CONCLUSION: After either placebo or laser therapy, pain and temporomandibular symptoms were significantly lower, although there was no significant difference between groups. The low-level laser therapy was not effective in the treatment of TMD, when compared to the placebo.

PMID: 18783114

Healing of immediate transmucosal implants in the esthetic zone

Healing of immediate transmucosal implants in the esthetic zone

Objectives: -to compare the clinical outcomes of standard, cylindrical, screw- shaped to novel tapered, transmucosal implants immediately placed into extraction sockets. Material and methods: In this randomized controlled clinical trial, outcomes were evaluated over a 3-year observation period. This report deals with the need for bone augmentation, healing events, implant stability and patient- centered outcomes up to 3 months only. Nine centres contributed a total of 208 immediate implant placements. All surgical and post-surgical procedures and the evaluation parameters were discussed with representatives of all centres during a calibration meeting. Following careful luxation of the designated tooth,allocation of the devices was randomly performed by a central study registrar. If the extraction socket was ≥1mm larger than the implant, guided bone regeneration was performed simultaneously (Bio Oss® and BioGide®). The flaps were then sutured. At surgery, the need for augmentation and the degree of wound closure was verified. Implant stability was assessed clinically and by means of resonance frequency analysis (RFA) at surgery and after 3 months. Wound healing was evaluated after 1, 2, 6 and 12 weeks postoperatively. Results: The demographic data did not show any differences between the patients receiving either standard cylindrical or tapered implants. All implants yielded uneventful healing with 15 % wound dehiscences after 1 week. After 2 weeks 93%, after 6 weeks 96% and after 12 weeks 100% of the flaps were closed. 90% of both implant designs required bone augmentation. Immediately after implantation RFA values were 55.8 and 56.7 and at 3 months 59.4 and 61.1 for cylindrical and tapered implants, respectively. Patient- centered outcomes did not differ between the two implant designs. Conclusions: This RCT has demonstrated that tapered or standard cylindrical implants yielded clinically equivalent short- term outcomes after immediate implant placement into the extraction socket. Supported by ITI Foundation, Basel, Switzerland

Division: IADR/AADR/CADR General Session
Meeting: 2007 IADR/AADR/CADR General Session (New Orleans, Louisiana)
Location: New Orleans, Louisiana
Year: 2007
Final Presentation ID: 51

Authors

- Lang, Niklaus P. (University of Berne, School of Dental Medicine, Berne, N/A, Switzerland)
- Tonetti, Maurizio (University of Berne, Switzerland, Berne, N/A, Switzerland)
- Behalf Of Ergo Perio, O. N. (University of Bern, Berne, N/A, Switzerland)

Lang, Niklaus P. (University of Berne, School of Dental Medicine, Berne, N/A, Switzerland)
Tonetti, Maurizio (University of Berne, Switzerland, Berne, N/A, Switzerland)
Behalf Of Ergo Perio, O. N. (University of Bern, Berne, N/A, Switzerland)

SESSION INFORMATION
Oral Session
Implant Survival Studies: Clinical Outcomes
03/21/2007

Healing of immediate transmucosal implants in the esthetic zone

CLINICAL ORAL IMPLANTS RESEARCH WILEY

Full Access

Immediate implant placement with transmucosal healing in areas of aesthetic priority: A multicentre randomized-controlled clinical trial I. Surgical outcomes

Niklaus P. Lang, Maurizio S. Tonetti, Jean E. Suvan, Jean Pierre Bernard, Daniele Botticelli, Ioannis Fourmousis, Marlene Hallund, Ronald Jung, Lars Laurell, Giovanni E. Salvi, ... See all authors

First published: 24 January 2007 | https://doi.org/10.1111/j.1600-0501.2006.01371.x | Cited by: 71

Correspondence to:
Niklaus P. Lang
Universität Bern, Zahnmedizinische Kliniken
Tel.: +41 316 32 2577
Fax: +41 316 32 4915
e-mail: perio@zmk.unibe.ch

Read the full text > PDF TOOLS SHARE

Abstract

Objectives: To compare the clinical outcomes of standard, cylindrical, screw-shaped to novel tapered, transmucosal (Straumann Dental®) implants immediately placed into extraction sockets.

Material and methods: In this randomized-controlled clinical trial, outcomes were evaluated over a 3-year observation period. This report deals with the need for bone augmentation, healing events, implant stability and patient-centred outcomes up to 3 months only. Nine centres contributed a total of 208 immediate implant placements. All surgical and post-surgical procedures and the evaluation parameters were discussed with representatives of all centres during a calibration meeting. Following careful luxation of the designated tooth, allocation of the devices was randomly performed by a central study registrar. The allocated SLA titanium implant was installed at the bottom or in the palatal wall of the extraction socket until primary stability was reached. If the extraction socket was ≥1 mm larger than the implant, guided bone regeneration was performed simultaneously (Bio Oss® and BioGide®). The flaps were then sutured. During non-submerged transmucosal healing, everything was done to prevent infection. At surgery, the need for augmentation and the degree of wound closure was verified. Implant stability was assessed clinically and by means of resonance frequency analysis (RFA) at surgery and after 3 months. Wound healing was evaluated after 1, 2, 6 and 12 weeks post-operatively.

Results: The demographic data did not show any differences between the patients receiving either standard cylindrical or tapered implants. All implants yielded uneventful healing with 15% wound dehiscences after 1 week. After 2 weeks, 93%, after 6 weeks 96%, and after 12 weeks 100% of the flaps were closed. Ninety percent of both implant designs required bone augmentation. Immediately after implantation, RFA values were 55.8 and 56.7 and at 3 months 59.4 and 61.1 for cylindrical and tapered implants, respectively. Patient-centred outcomes did not differ between the two implant designs. However, a clear preference of the surgeon's perception for the appropriateness of the novel-tapered implant was evident.

Conclusions: This RCT has demonstrated that tapered or standard cylindrical implants yielded clinically equivalent short-term outcomes after immediate implant placement into the extraction socket.

Inferior Jonit Space Injection Therapy in Patients with TMD

Inferior Jonit Space Injection Therapy in Patients with TMD

Aims: To compare the outcome of inferior joint space and superior joint space injection of sodium hyaluronate in patients with anterior disc displacement without reduction of the temporomandibular joint (TMJ). Methods: 120 patients were randomized to two groups, one group receiving superior joint space injections of sodium hyaluronate and the other receiving inferior joint space injections of sodium hyaluronate. To evaluate the efficacy of both procedures based on the diagnosis of clinical signs and symptoms of anterior disc displacement without reduction in the 3 and 6 months follow-up after the last of the three injection therapies by maximal mouth opening (MMO), pain intensity on a visual analogue scale(VAS), and modified Helkimo's clinical dysfunction index . Results: In the superior joint space therapy, 50 patients follow up for 3 months and 6 months and in the inferior joint space therapy, 54 patients follow up for 3 months and 6 months. MMO, VAS and Helkimo's index improved in both the superior joint space therapy and the inferior joint space therapy at 3 months and 6 months follow up. There was a significant reduction in TMJ pain in the inferior joint injection group at 3 months follow-up as compare to the superior joint injection group ($p<0.001$), but the results of increasing MMO and TMJ function was almost the same in both groups at 3 months follow-up. There was a significant difference between the inferior joint injection group and superior joint injection group in MMO ($p<0.005$), VAS ($p<0.001$) and Helkimo's index ($p<0.001$) at 6 months follow-up. Conclusion: This study demonstrated that the inferior joint space injection with sodium hyaluronate is a valid method of treating the anterior disc displacement without reduction of TMJ.

Division: IADR/AADR/CADR General Session
Meeting: 2007 IADR/AADR/CADR General Session (New Orleans, Louisiana)
Location: New Orleans, Louisiana
Year: 2007
Final Presentation ID: 723

Authors

- Long, Xing (Wuhan University Hospital of Stomatology, Wuhan, N/A, China)
- Deng, Mohong (Wuhan University Hospital of Stomatology, Wuhan, N/A, China)
- Cheng, Yong (Wuhan University Hospital of Stomatology, Wuhan, N/A, China)
- Cai, Hengxing (Wuhan University Hospital of Stomatology, Wuhan, N/A, China)

Long, Xing (Wuhan University Hospital of Stomatology, Wuhan, N/A, China)
Deng, Mohong (Wuhan University Hospital of Stomatology, Wuhan, N/A, China)
Cheng, Yong (Wuhan University Hospital of Stomatology, Wuhan, N/A, China)
Cai, Hengxing (Wuhan University Hospital of Stomatology, Wuhan, N/A, China)

SESSION INFORMATION
Poster Session
TMD/orofacial pain epidemiology and treatment
03/22/2007

Inferior Jonit Space Injection Therapy in Patients with TMD

ELSEVIER

Journal of Oral and Maxillofacial Surgery

Volume 67, Issue 2, February 2009, Pages 357-361

Basic and patient-oriented research

A Randomized Controlled Trial of Superior and Inferior Temporomandibular Joint Space Injection With Hyaluronic Acid in Treatment of Anterior Disc Displacement Without Reduction

Xing Long DDS, PhD *, Guoxin Chen MD †, Andrew Hua an Cheng BDS ‡, Yong Cheng DDS, PhD §, Mohong Deng DDS, PhD ‖, Hengxing Cai DDS, PhD ¶, Qinggong Meng DDS, MD #

⊞ Show more

https://doi.org/10.1016/j.joms.2008.09.014

Get rights and content

Purpose

To compare the outcome of inferior and superior joint space injection of sodium hyaluronate in patients with disc displacement without reduction of the temporomandibular joint (TMJ).

Materials and Methods

One hundred twenty patients with disc displacement without reduction of TMJ were randomized into 2 experimental groups. One group of patients received superior joint space injections of sodium hyaluronate and the other group was treated with inferior joint space injections. Patient's TMJ status and clinical symptoms were evaluated at the 3 and 6 month follow-up appointments. The clinical parameters recorded were maximal mouth opening (MMO), pain intensity on a visual analog scale (VAS), and modified Helkimo's clinical dysfunction index and analyzed with ANCOVA.

Results

Fifty of the superior and 54 of the inferior joint space injection therapy group returned for the 3 and 6 month evaluations; 86.67% of the patients were retained in the follow-up. MMO, VAS, and Helkimo's index of both groups improved at the 3 and 6 month follow-ups. The results of MMO changes and TMJ function were almost the same in both groups at 3 month follow-up. However, there was a significant reduction in TMJ pain in the inferior joint injection group at 3 month follow-up compared with the superior joint injection group ($P < .001$). There were also significant differences between the inferior joint injection group and superior joint injection group in MMO ($P < .005$), VAS ($P < .001$), and Helkimo's index ($P < .001$) at 6 month follow-up.

Conclusion

This study showed that inferior joint space injection with sodium hyaluronate is a valid method of treating disc displacement without reduction of TMJ and a long-term study will be needed to assess the effect of inferior joint injection on the morphologic changes of the TMJ.

Randomized Clinical Study of Initial Treatment for Temporomandibular Disorder ADDWOR

Objectives: A various treatment modalities have been proposed for patients with temporomandibular disorders (TMD) anterior disc displacement without reduction (ADDWOR). This study compared the effectiveness of splint and physical therapy for ADDWOR patients in a prospective randomized manner. Methods: From consecutive series of 856 TMD new patients who visited TMJ clinic at Tokyo Medical and Dental University from January to June 2006, 26 subjects (female: 23, male: 3, age: 37.3 ± 15.1) were selected according to the following inclusion criteria: over 18 years old, having pain on mouth opening with TMJ affected side, over 2 weeks after an onset of locking, mouth opening range < 40mm, and ADDWOR confirmed with MRI. Subjects were randomly assigned to one of two treatment groups: a splint treatment; a joint mobilization exercise. The splint group subjects were treated with flat occlusal appliance wearing while sleeping. The joint mobilization exercise group subjects were instructed to perform manual jaw opening exercise by themselves four times a day. The measured four outcome variables were maximum mouth opening range, visual analogue scale (VAS) for present pain intensity and food intake difficulty, and limitation of daily activities. The effect of these treatments was evaluated at a 4-week time point. For statistical analysis, the paired t-test was used to compare the outcome variables before and after treatment in each group (P<.05). Results: After 4-week follow up, significant improvement was observed in the joint mobilization exercise group with maximum mouth opening range, VAS for present pain intensity and food intake difficulty (P=.00), but no significant changes in splint group. Variable of limitation of daily activities significantly improved in both groups (P<.03). Conclusion: Applying a self joint mobilization exercise for TMD ADDWOR patients proved to be effective for better improvement of TMJ function and impairment of daily life.

Division: IADR/AADR/CADR General Session
Meeting: 2007 IADR/AADR/CADR General Session (New Orleans, Louisiana)
Location: New Orleans, Louisiana
Year: 2007
Final Presentation ID: 720

Authors

- Haketa, Tadasu (Tokyo Medical & Dental University, Tokyo, N/A, Japan)
- Kino, Koji (Tokyo Medical & Dental University, Tokyo, N/A, Japan)
- Sato, Fumiaki (Tokyo Medical & Dental University, Tokyo, N/A, Japan)
- Ishikawa, Takayuki (Tokyo Medical & Dental University, Tokyo, N/A, Japan)
- Sugisaki, Masashi (Jikei University School of Medicine, Tokyo, N/A, Japan)

SESSION INFORMATION
Poster Session
TMD/orofacial pain epidemiology and treatment
03/22/2007

Haketa, Tadasu (Tokyo Medical & Dental University, Tokyo, N/A, Japan)
Kino, Koji (Tokyo Medical & Dental University, Tokyo, N/A, Japan)
Sato, Fumiaki (Tokyo Medical & Dental University, Tokyo, N/A, Japan)
Ishikawa, Takayuki (Tokyo Medical & Dental University, Tokyo, N/A, Japan)
Sugisaki, Masashi (Jikei University School of Medicine, Tokyo, N/A, Japan)

Randomized Clinical Study of Initial Treatment for Temporomandibular Disorder ADDWOR

Journal of Dental Research
IADR International Association for Dental Research
AADR® American Association for Dental Research

Journal Home | Browse Journal | Submit Paper | About | Subscribe

Close
PDF

Randomized Clinical Trial of Treatment for TMJ Disc Displacement

T. Haketa*, K. Kino, M. Sugisaki, M. Takaoka, T. Ohta

Show less

First Published August 25, 2010 | Research Article
https://doi.org/10.1177/0022034510378424

Article information

Article Information
Volume: 89 issue: 11, page(s): 1259-1263
Article first published online: August 25, 2010; Issue published: November 1, 2010

T. Haketa[1]*, K. Kino[1], M. Sugisaki[2], M. Takaoka[1], T. Ohta[1]
[1]Temporomandibular Joint and Occlusion, Graduate School, Tokyo Medical and Denta
Yushima, Bunkyo-ku, Tokyo, 113–8549, Japan
[2]Department of Dentistry, Jikei University School of Medicine, Tokyo, Japan

Corresponding Author: *haketa-mvrk@polka.ocn.ne.jp

Abstract

Of the various conservative treatment modalities available for temporomandibular disorders, we believe that therapeutic exercise has a good prognosis, especially for anterior disc displacement without reduction. Since its effectiveness has not been extensively evaluated, we conducted a comparative study to verify the hypothesis that treatment efficacy would not differ for exercise and occlusal splints. Fifty-two individuals with anterior disc displacement without reduction were randomly assigned to a splint or a joint mobilization self-exercise treatment group. Four outcome variables were evaluated: (i) maximum mouth-opening range without and (ii) with pain, (iii) current maximum daily pain intensity, and (iv) limitation of daily functions. All outcome variables significantly improved after 8 weeks of treatment in both groups. In particular, the mouth opening range increased more in the exercise group than in the splint group. This result demonstrates that therapeutic exercise brings earlier recovery of jaw function compared with splints.

Keywords

temporomandibular disorders, anterior disc displacement without reduction, randomized controlled clinical trial, physical therapy, splint

The impact of mandibular 2-implant overdentures on denture stomatitis

The impact of mandibular 2-implant overdentures on denture stomatitis

The recent literature has provided strong evidence for the superiority of mandibular 2-implant overdentures over conventional dentures in terms of health related quality of life, patient satisfaction and chewing ability. However, little attention has been given to the effect of mandibular implant overdentures on oral mucosal health and, specifically, denture stomatitis.
Objectives: This study aimed to assess and compare the frequency of denture stomatitis in an elderly edentulous population wearing mandibular two-implant overdentures and conventional dentures.

Methods: Two hundred twenty-two (222) edentulous elders (men and women; 65 years and over) enrolled in a randomized clinical study were followed over a one-year period. They randomly received either mandibular overdentures retained by ball attachments on two implants (ITI, Straumann, Waldenburg, Switzerland) or conventional dentures, both opposed by new conventional maxillary dentures. Data were derived from oral clinical examinations of the maxillary mucosa carried out by two calibrated examiners. Diagnosis of denture stomatitis was based on a modified Newton classification.

Results: Inter-rater reliability was good (kappa=0.81). Furthermore, a preliminary analysis (n=80) showed that a significantly higher proportion of subjects wearing mandibular conventional dentures had denture stomatitis (78%) compared to those with implant overdentures (48%, p=0.01 Fisher's exact test). Participants wearing conventional dentures were almost 4 times more likely to have denture stomatitis than those wearing mandibular 2-implant overdentures (Odds ratio=3.8, 95% C.I.=1.4-10.2).

Conclusion: The results of this study suggest that, in edentulous elders, better maxillary oral mucosal health may result when mandibular dentures are supported by two implants.

Division: IADR/AADR/CADR General Session
Meeting: 2007 IADR/AADR/CADR General Session (New Orleans, Louisiana)
Location: New Orleans, Louisiana
Year: 2007
Final Presentation ID: 959

Authors
- Emami, Elham (Université de Montréal, Montreal, QC, Canada)
- Rompré, Pierre H. (Université de Montréal, Montreal, QC, Canada)
- De Grandmont, Pierre (Université de Montréal, Montreal, QC, Canada)
- Pan, Shao-xia (McGill University, Montreal, QC, Canada)
- Feine, Jocelyne (McGill University, Montreal, QC, Canada)

Emami, Elham (Université de Montréal, Montreal, QC, Canada)
Rompré, Pierre H. (Université de Montréal, Montreal, QC, Canada)
De Grandmont, Pierre (Université de Montréal, Montreal, QC, Canada)
Pan, Shao-xia (McGill University, Montreal, QC, Canada)
Feine, Jocelyne (McGill University, Montreal, QC, Canada)

SESSION INFORMATION
Poster Session
Morita Awards
03/22/2007

The impact of mandibular 2-implant overdentures on denture stomatitis

ELSEVIER

Journal of Dentistry

Volume 38, Issue 3, March 2010, Pages 232-236

Better oral health related quality of life: Type of prosthesis or psychological robustness?

E. Emami [a], P.J. Allison [b], P. de Grandmont [a], P.H. Rompré [c], J.S. Feine [b, d, e]

[a] Département de dentisterie de restauration, Faculté de Médecine dentaire, Université de Montréal, Montréal, Canada

[b] Faculty of Dentistry, McGill University, Montreal, Canada

[c] Département de stomatologie, Faculté de Médecine dentaire, Université de Montréal, Montréal, Canada

[d] Department of Epidemiology and Biostatistics, Faculty of Medicine, Faculty of Dentistry, McGill University, Montreal, Canada

[e] Department of Oncology, Faculty of Medicine, Faculty of Dentistry, McGill University, Montreal, Canada

Received 18 September 2009, Revised 7 November 2009, Accepted 11 November 2009, Available online 18 November 2009.

⊟ Show less

https://doi.org/10.1016/j.jdent.2009.11.002 Get rights and content

Abstract

Sense of coherence (SOC) is an individual-based coping characteristic and believed to influence a person's ability to adapt to life stressors, such as edentulism and using complete denture. Thus, SOC may mediate the effect of prosthetic treatment on quality of life.

Objectives

1. To simultaneously test the effect of type of treatment and sense of coherence on oral health related quality of life (OHRQoL) in edentate elders and to identify any interaction. 2. To report the level of sense of coherence among a sample of edentate elders.

Methods

Data were collected and analysed cross-sectionally at a 1-year follow-up from 173 edentulous elders who had randomly received mandibular-implant overdentures or conventional dentures, both opposed by new conventional maxillary dentures. The dependent outcome variable, oral health related quality of life, was measured using the Oral Health Impact Profile (OHIP-20). Independent variables included SOC and prosthesis type, as well as socio-demographic variables. SOC was evaluated using the 13-item likert scale of The Orientation to Life questionnaire.

Results

The group mean SOC score was 70.28 (SD = 9.6). Married or coupled people had significantly higher SOC scores than those who were separated, single or divorced ($p = 0.04$). General linear model analyses demonstrated that there was a statistically significant main effect for type of prosthesis, $F(1.169) = 0.71$, $p = 0.008$, with no interaction with SOC.

Conclusion

The results of this study suggest that, in edentulous elders, SOC does not mediate the effect of the type of prosthetic treatment on oral health related quality of life.

2008

12-Year Clinical Evaluation of a Dual-Cured Hydrophilic Dental Adhesive

12-Year Clinical Evaluation of a Dual-Cured Hydrophilic Dental Adhesive

Objectives: The purpose of this randomized clinical trial was to evaluate the performance of a fluoride-releasing dual-cured hydrophilic dental adhesive used with or without dentin acid-etching to restore non-carious cervical lesions. Methods: One hundred lesions were characterized preoperatively relative to height, width, depth, volume, occlusion, enamel margin (%), internal angle, and degree of sclerosis. The exposed walls of the lesion were roughened with a diamond instrument. No retentive grooves or bevels were used. Lesions were randomly assigned to two treatment groups (max.3/group/subject): In Group A (enamel etch only), the enamel was etched with 37% phosphoric acid for 30 seconds; in Group B (enamel and dentin etch), the enamel was etched as in Group A, and the dentin was etched with 37% phosphoric acid for 15 seconds. After acid treatment, all lesions were treated with a light-cured primer and a dual-cured adhesive (OptiBond, Kerr). The preparations were restored with a light-cured composite (Herculite XRV, Kerr), and evaluated by masked evaluators at baseline and at 12 years post-insertion for interfacial staining, secondary caries, marginal adaptation, post-operative sensitivity, and retention using modified USPHS criteria (Alfa=excellent, Bravo=clinically acceptable, Charlie=clinically unacceptable). Data were analyzed using two-sample t-tests and Pearson's Chi-Square (p=0.05). Results: Lesion characteristics were similar at baseline. All baseline scores were Alfa for both treatment groups. At 12 years, the recall rates were 54% (27 restorations) for Group A and 38% (19 restorations) for Group B. Two retention failures were noted in Group A, and three in Group B, for an overall retention rate of 89%. No statistically significant differences were detected between the two treatment groups for retention or any of the other evaluation criteria. Conclusion: The 12-year clinical performance, including retention, of a dual-cured hydrophilic dental adhesive was excellent and was not affected by dentin acid etching. Supported by Kerr.

Division: IADR/CADR General Session
Meeting: 2008 IADR/CADR General Session (Toronto, Ontario, Canada)
Location: Toronto, Ontario, Canada
Year: 2008
Final Presentation ID: 239

Authors

- Wilder, Aldridge D. (University of North Carolina, Chapel Hill, NC, USA)
- Ritter, Andre V. (University of North Carolina, Chapel Hill, NC, USA)
- Heymann, Harald (University of North Carolina, Chapel Hill, NC, USA)
- Sturdevant, John (University of North Carolina, Chapel Hill, NC, USA)
- Swift, Ed (University of North Carolina, Chapel Hill, NC, USA)
- Bayne, Stephen C. (University of Michigan, Ann Arbor, MI, USA)

SESSION INFORMATION
Oral Session
Clinical Performance of Resin Composite Restorations
07/03/2008

Wilder, Aldridge D. (University of North Carolina, Chapel Hill, NC, USA)
Ritter, Andre V. (University of North Carolina, Chapel Hill, NC, USA)
Heymann, Harald (University of North Carolina, Chapel Hill, NC, USA)
Sturdevant, John (University of North Carolina, Chapel Hill, NC, USA)
Swift, Ed (University of North Carolina, Chapel Hill, NC, USA)
Bayne, Stephen C. (University of Michigan, Ann Arbor, MI, USA)

12-Year Clinical Evaluation of a Dual-Cured Hydrophilic Dental Adhesive

Clinical Evaluation of a Dual-Cured Hydrophilic Dentin Adhesive "Kerr OptiBond Study" Report of 12Year Recall Evaluation

来自ResearchGate

收藏　<> 引用　批量引用　报错　分享

作者　HO Heymann ， AV Ritter ， JR Sturdevant ， EJ Swift

摘要　ABSTRACT The objective of this project was to evaluate the clinical
dentin adhesive (Kerr OptiBond). One hundred caries-free Class V n
mechanical retention (retentive grooves) were used. The cavosurfac
beveled. The cavity preparations were divided into two groups for ac
A were treated with 37% phosphoric acid gel for 30 seconds. Both th

出版源　《Test De》 , 2012

被引量　0

ABSTRACT

The objective of this project was to evaluate the clinical performance of a dual-cured hydrophilic dentin adhesive (Kerr OptiBond). One hundred caries-free Class V non-carious cervical lesions without macro-mechanical retention (retentive grooves) were used. The cavosurface margins of the cavity preparations were not beveled. The cavity preparations were divided into two groups for acid treatment. Only the enamel walls of Group A were treated with 37% phosphoric acid gel for 30 seconds. Both the enamel and dentin walls of Group B were treated with 37% phosphoric acid.

The enamel walls were treated for 30 seconds as in Group A. The dentin walls were treated for 15 seconds only. After acid treatment, the enamel and dentin walls of all cavity preparations were primed with a light-cured primer (Kerr T-Sealer). A fluoride-releasing adhesive dual-cured liner (Kerr OptiBond) was applied to the primed surface. The preparations were restored with a light-cured composite resin filling material (Kerr Herculite XRV). The restorations were evaluated directly at insertion (baseline), 1 year and 12 years.

At 12 years, the recall rate for Group A was 54% (27 restorations), and for Group B it was 38% (19 restorations). The recall rate at 12 years was 46% (46 restorations) for Groups A and B combined. Two retention failures were noted in Group A, and three were noted in Group B. At 12 years, the retention of adhesively bonded OptiBond/Herculite XRV Class V restorations was 93% in Group A and 84% in Group B, or 89% for the combined groups. Except for marginal discoloration in both Groups and retention in Group B, all of the direct clinical evaluation categories in both groups of restorations were rated 88% alfa or higher.

Clinical Comparison of Proximal Contacts Obtained with Different Matrix Systems

Clinical Comparison of Proximal Contacts Obtained with Different Matrix Systems

Objectives: The aim of this clinical randomized single blind study was to investigate in vivo the proximal contact tightness (PCT) of direct posterior composite resin restorations obtained with two different matrix systems before, directly after treatment and after a period of two weeks.
Methods: 85 consecutive patients without periodontitis being in need of a Class II direct resin restoration were randomly assigned to two groups. Group I (n = 45) was treated with a circumferential matrix system (Tofflemire, Produits Dentaire SA, Vevey, Switzerland). Group II (n = 40) with a sectional matrix system (Palodent Matrix System, Dentsply International Inc. Milford, DE). Proximal contact tightness was measured in Newton using a modified Tooth Pressure Meter (Dörfer et al. (2000)) directly before (T1), after (T2) restoration placement and after a period of two weeks (T3) at the site of treatment as well as at corresponding teeth in the contralateral quadrant as control. Differences over time were tested by ANOVA followed by the post hoc Scheffé-test and differences between the two groups by the T-test for independent variables (p=0.05).

Results: Group I effected an average reduction of PCT from 4.41 N ± 3.06 N (T1) to 2.95 N ± 1.66 N (T2) and 3.47 N ± 1.71 N (T3) ($p<0.05$), whereas Group II lead to an average increase of PCS from 4.31 ± 2.60 N (T1) to 5.65 N ± 2.63 N (T2) and 5.14 ± 2.05 N (T3) ($p<0.05$). Differences of PCT between the groups were statistically significant directly after treatment as well as two weeks later ($p<0.05$).

Conclusions: Use of traditional circumferential matrix systems without a separation ring will lead to a significant loss in PCT, whereas the use of sectional matrix systems combined with separation rings will lead to an increase of PCT.

Division: IADR/CADR General Session
Meeting: 2008 IADR/CADR General Session (Toronto, Ontario, Canada)
Location: Toronto, Ontario, Canada
Year: 2008
Final Presentation ID: 2860

Authors

- Wirsching, Eva (Christian-Albrechts-University Kiel, Kiel, N/A, Germany)
- Loomans, Bas A.c. (Radboud University Nijmegen Medical Centre, Nijmegen, N/A, Netherlands)
- Staehle, H. J. (University of Heidelberg, Heidelberg, N/A, Germany)
- Dorfer, Christof E. (Christian-Albrechts-University Kiel, Kiel, N/A, Germany)

SESSION INFORMATION
Oral Session
Assessment of Devices and Dentifrices
07/05/2008

Wirsching, Eva (Christian-Albrechts-University Kiel, Kiel, N/A, Germany)
Loomans, Bas A.c. (Radboud University Nijmegen Medical Centre, Nijmegen, N/A, Netherlands)
Staehle, H. J. (University of Heidelberg, Heidelberg, N/A, Germany)
Dorfer, Christof E. (Christian-Albrechts-University Kiel, Kiel, N/A, Germany)

Clinical Comparison of Proximal Contacts Obtained with Different Matrix Systems

J Dent. 2011 May;39(5):386-90. doi: 10.1016/j.jdent.2011.03.001. Epub 2011 Mar 21.

Influence of matrix systems on proximal contact tightness of 2- and 3-surface posterior composite restorations in vivo.

Wirsching E[1], Loomans BA, Klaiber B, Dörfer CE.

Author information

1 Department of Conservative Dentistry and Periodontology, Julius-Maximilians-University of Würzburg, Würzburg, Germany. Wirsching_e@klinik.uni-wuerzburg.de

Abstract

OBJECTIVES: To investigate the influence of cavity preparation (MO/DO/MOD) and type of matrix system on proximal contact tightness of direct posterior composite restorations.

MATERIALS AND METHODS: 85 patients in need of a two- or three surface Class II direct composite restoration were randomly divided into two treatment groups. Group 1 was treated with a sectional matrix system combined with a separation ring (Palodent); Group 2 was treated with a circumferential matrix system in combination with a retainer (Tofflemire). Proximal contact tightness was recorded before treatment and directly after finishing the restoration.

RESULTS: For the two-surface cavities use of the separation ring resulted in a statistically significantly tighter proximal contacts at both the mesial and distal site (MO: 2.51±0.81 N; DO: 2.82±1.14 N) compared to the use of the circumferential (MO: -1.08±1.04 N; DO: -0.22±0.87 N) (p=0.01). Regarding the three-surface (MOD) cavities no statistically significant differences were found between the mesial and distal site, nor was there an effect of the used matrix system. No statistically significant influence of cavity design (mesially/distally) was recorded for all cavities (MO, DO and MOD).

CONCLUSIONS: Use of the sectional matrix system in two-surface Class II cavities resulted in statistically significantly tighter proximal contacts than the use of the circumferential matrix system. For the three-surface no statistically significant differences in contact tightness were found between the different matrix systems. Location of the cavity (mesially or distally) did not show to have any statistically significant effect on the obtained proximal contact tightness.

Copyright © 2011 Elsevier Ltd. All rights reserved.

PMID: 21414384 DOI: 10.1016/j.jdent.2011.03.001

Converting one-step into two-step self-etch system improves its clinical performance

Converting one-step into two-step self-etch system improves its clinical performance

Objectives: A 18-month randomized, controlled prospective study evaluated, in an intra-individual comparison, the clinical performance of two one-step self-etch adhesives in non-carious cervical lesions [NCCL] applied as recommended or with an extra layer of hydrophobic adhesive. Methods: Thirty patients, with at least four similar sized NCCL participated in this study. After sample size calculation, 120 restorations were placed, according to one of the following groups: Clearfil S3 Bond [CS] and iBond [IB] applied according to manufacturer's instructions and Clearfil S3 Bond [CSB] and iBond [iBB] applied as recommended plus an extra layer of hydrophobic resin layer [Bond, Scotchbond Multi Purpose]. The restorations were placed incrementally using a composite resin. The restorations were evaluated at baseline and after 18 months according to the modified USPHS criteria. Statistical analysis was made with Friedman repeated measures analysis of variance by rank and Wilcoxon sign-ranked test for significance at each pair (alfa=0.05). Results: All restorations were evaluated at 18 months. The retention rate of the group IB after 18 months (60%) was statistically lower than groups CS (77.3%), IBB (83.4%) and CSB (93.4%). Although the application of an extra layer of a hydrophobic resin improved the retention rate of both adhesives, this was only statistically significant for iBond. Marginal discoloration occurred in all groups, being statistically worse in group IB, which was scored as beta in 94.4% of the cases. Conclusions: iBond applied according to the manufacturer's recommendations showed a very low retention rate after 18 months. The use of a bond layer of Scotchbond Multi Purpose associated with iBond adhesive can improve its clinical performance.

Division: IADR/CADR General Session
Meeting: 2008 IADR/CADR General Session (Toronto, Ontario, Canada)
Location: Toronto, Ontario, Canada
Year: 2008
Final Presentation ID: 1781

Authors

- Reis, Alessandra (Ponta Grossa State University, Ponta Grossa, N/A, Brazil)
- Matté, Karina (University of Oeste de Santa Catarina, Joaçaba, PA, Brazil)
- Meurer, Roberto (University of Oeste de Santa Catarina, Joaçaba, N/A, Brazil)
- Geraldeli, Saulo (University of Iowa, Iowa City, IA, USA)
- Loguercio, Alessandro Dourado (Universidade Estadual de Ponta Grossa, Ponta Grossa - PR, N/A, Brazil)

Reis, Alessandra (Ponta Grossa State University, Ponta Grossa, N/A, Brazil)
Matté, Karina (University of Oeste de Santa Catarina, Joaçaba, PA, Brazil)
Meurer, Roberto (University of Oeste de Santa Catarina, Joaçaba, N/A, Brazil)
Geraldeli, Saulo (University of Iowa, Iowa City, IA, USA)
Loguercio, Alessandro Dourado (Universidade Estadual de Ponta Grossa, Ponta Grossa - PR, N/A, Brazil)

SESSION INFORMATION
Poster Session
Clinical Performance of Dental Adhesives
07/04/2008

Converting one-step into two-step self-etch system improves its clinical performance

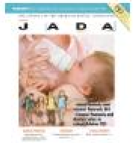

ELSEVIER

The Journal of the American Dental Association

Volume 140, Issue 7, July 2009, Pages 877-885

Improving Clinical Retention of One-Step Self-Etching Adhesive Systems With an Additional Hydrophobic Adhesive Layer

Alessandra Reis DDS, PhD [1], Tatiana Moroz Leite DDS [2], Karina Matte DDS [3], Roberto Michels DDS [4], Roberto C. Amaral DDS, MS [5], Saulo Geraldeli DDS, MS, PhD [6], Alessandro Dourado Loguercio DDS, MS, PhD [7]

ABSTRACT

JADA CE CONTINUING EDUCATION ARTICLE 2

Background

The durability of restorations bonded with one-step self-etching (OSSE) adhesive systems is inferior compared with that of restorations bonded with conventional adhesives. The authors conducted an 18-month randomized clinical study to evaluate the clinical performance of two OSSE systems in noncarious cervical lesions applied as recommended or with an extra layer of hydrophobic adhesive layer after 18 months of clinical service.

Methods

Thirty participants, each of whom had at least two pairs of similar-sized noncarious cervical lesions, took part in this study. The authors placed 120 restorations, 30 in each of four groups: Clearfil S^{3} Bond (Kuraray, Osaka, Japan) (CS) and iBond Gluma inside (Heraeus Kulzer, Hanau, Germany) (IB), and Clearfil S^{3} Bond (CSB) and iBond Gluma inside (IBB) with an extra layer of hydrophobic adhesive applied on top of them. They placed the restorations incrementally, using a resin-based composite. The authors evaluated the restorations at baseline and at 18 months following modified U.S. Public Health Service criteria.

Results

At 18 months, the retention rate for the IB group was statistically lower than those for the CS, IBB and CSB groups. Marginal discoloration occurred in all groups and was statistically worse in the IB group.

Conclusions

The conversion of the iBond Gluma inside and Clearfil S^{3} Bond adhesive systems into two-step systems by means of applying an extra hydrophobic adhesive layer improved the clinical performance of these materials after 18 months of clinical service.

Clinical Implications

The application of an extra hydrophobic adhesive layer over OSSE adhesive systems, layers improved the OSSE systems clinical performance, mainly in terms of retention rate.

Cost-efficiency of Two Methods of Fabrication of Conventional Dentures

Cost-efficiency of Two Methods of Fabrication of Conventional Dentures

We carried out a randomized controlled clinical trial to compare traditional (T) and simplified (S) methods of fabricating conventional dentures for 118 edentate subjects (Kawai et al, 2005). The T method includes additional steps: final impressions, face-bow registration, mounting on an adjustable articulator, and occlusal adjustment on the articulator, that are not used in the S method. We showed that the method of fabrication had no significant effect on patent satisfaction or on denture quality assessed by prosthodontists. In this paper, we report on cost-efficiency for clinicians by computing earning per hour using the T and S methods. The relationship between patient ratings of satisfaction and fabrication method was also investigated.
Objectives: To compare the cost-efficiency of providing maxillary and mandibular conventional dentures using T and S methods.

Methods: We measured the direct material costs and laboratory costs in Canadian $, time spent by the prosthodontists on treatment for up to 6 months post-delivery, and patient's ratings of general satisfaction (0-100). Gross income was calculated by deducting total costs from the suggested fee (Quebec Association of Dentists fee guide) for one set of complete dentures.

Results: Mean gross income per hour was significantly higher ($p<0.001$) with S (\$278) than with the T technique (\$170). There was also a low, but significant positive association between income per hour and patient general satisfaction (S: $r=0.34$, $p<0.01$, T: $r=0.48$, $p<0.01$).

Conclusion: The S method is more cost-efficient than the T method for fabrication of conventional dentures.

Division: IADR/CADR General Session
Meeting: 2008 IADR/CADR General Session (Toronto, Ontario, Canada)
Location: Toronto, Ontario, Canada
Year: 2008
Final Presentation ID: 2438

Authors

- Kawai, Yasuhiko (Nihon University, Matsudo, Chiba, N/A, Japan)
- Murakami, H. (Nihon University, Matsudo, Chiba, N/A, Japan)
- Lund, James P. (McGill University, Montreal, QC, Canada)
- Feine, Jocelyne (McGill University, Montreal, QC, Canada)

SESSION INFORMATION
Poster Session
Health Services Research II
07/04/2008

Kawai, Yasuhiko (Nihon University, Matsudo, Chiba, N/A, Japan)
Murakami, H. (Nihon University, Matsudo, Chiba, N/A, Japan)
Lund, James P. (McGill University, Montreal, QC, Canada)
Feine, Jocelyne (McGill University, Montreal, QC, Canada)

Cost-efficiency of Two Methods of Fabrication of Conventional Dentures

J Prosthodont. 2010 Oct;19(7):512-6. doi: 10.1111/j.1532-849X.2010.00628.x. Epub 2010 Aug 16.

Efficient resource use in simplified complete denture fabrication.

Kawai Y[1], Murakami H, Takanashi Y, Lund JP, Feine JS.

Author information

1 Nihon University, Sakaecho-Nishi Matsudo, Chiba, Japan. kawai.yasuhiko@nihon-u.ac.jp

Abstract

PURPOSE: Conventional dentures will remain the only treatment available to most edentulous people for the foreseeable future. In this study, we compared the efficiency of two methods of making complete conventional dentures-the traditional academic standard (T) and a simplified technique (S) used in private practice. We have previously shown that they produce similar levels of patient satisfaction and denture quality.

MATERIALS AND METHODS: Data were gathered during a randomized controlled clinical trial of 122 subjects from initial examination until 6-month follow-up. For this report, the direct costs of providing one set of conventional complete dentures by T or S techniques were estimated. All materials used were recorded and their cost was calculated in Canadian dollars (CAN$). The costs of fabrication in an outside laboratory were added. Clinician's labor time was recorded for every procedure. Between-group comparisons for each clinical procedure were carried out with independent t-tests. The number of patients in each group who needed postdelivery treatment was compared with Chi-square tests. The effect of group assignment and of treatment difficulty on outcomes was analyzed with multiple regression analysis.

RESULTS: The mean total cost of the T method was significantly greater than S (CAN$166.3; $p < 0.001$), and clinicians spent 90 minutes longer ($p < 0.001$) on clinical care. The difficulty of the case had no significant influence on outcomes.

CONCLUSIONS: The results indicate that the S method is the more cost-efficient method and that there are no negative consequences that detract from the cost savings.

© 2010 by The American College of Prosthodontists.

PMID: 20723019 DOI: 10.1111/j.1532-849X.2010.00628.x

Does mandibular condition affect prosthetic treatment success in edentulous elders?

Does mandibular condition affect prosthetic treatment success in edentulous elders?

Objectives: The aim of this study is to determine whether mandibular condition affects patients' ratings of satisfaction and function with mandibular implant overdentures and conventional dentures.
Methods: 214 edentulous elders were randomly allocated into 2 groups and treated with maxillary conventional dentures and either mandibular overdentures supported by two implants with ball attachments or mandibular conventional dentures. Classification of mandibular bone height and alveolar resorption was carried out on panoramic radiographs using 4 published methods. Maxillomandibular relationship, mandibular ridge form and soft tissue quality were also recorded. At baseline and at 6 months after delivery, all participants rated their satisfaction with their prostheses using the McGill Denture Satisfaction Instrument. Independent t-tests and a linear multivariable regression model were used.

Results: Mandibular condition has no effect on ratings of general satisfaction with prostheses, nor on satisfaction with ability to chew, stability, comfort, aesthetics and ability to speak at 6 months ($p>0.05$, linear regression). There were significant between-treatment differences in ratings of general satisfaction in all mandibular condition categories, with greater general satisfaction ratings assigned to implant overdentures. These differences were also seen for comfort, stability and ability to chew ($p<0.01$, t-tests). For general satisfaction, as well as satisfaction with ability to chew, stability, comfort, aesthetics and ability to speak, results confirm that, at 6 months after delivery, treatment with implant overdentures contributes to higher satisfaction ratings ($p<0.001$, linear regression).

Conclusion: The results of this study do not support the assumption that the condition of the mandible has an effect on elderly edentulous patients' satisfaction with their prostheses. Therefore, the condition of the mandible may not be an appropriate criterion for choice of prosthesis type. No matter what mandibular condition, elderly patients will experience greater satisfaction with implant overdentures than with conventional dentures.

Division: IADR/CADR General Session
Meeting: 2008 IADR/CADR General Session (Toronto, Ontario, Canada)
Location: Toronto, Ontario, Canada
Year: 2008
Final Presentation ID: 1900

Authors

- Pan, Shao Xia (McGill University, Montreal, QC, Canada)
- Dagenais, Marie (McGill University, Montreal, QC, Canada)
- Thomason, J.m. (Newcastle University, Newcastle upon Tyne, N/A, United Kingdom)
- Awad, Manal (University of Sharjah, Sharjah, N/A, United Arab Emirates)
- Emami, Elham (Université de Montréal, McGill University, Montreal, QC, Canada)
- Kimoto, Suguru (Nihon University, Matsudo, N/A, Japan)
- Feine, Jocelyne (McGill University, Montreal, QC, Canada)

SESSION INFORMATION
Poster Session
Topical Issues in Geriatric Oral Research
07/04/2008

Pan, Shao Xia (McGill University, Montreal, QC, Canada)
Dagenais, Marie (McGill University, Montreal, QC, Canada)
Thomason, J.m. (Newcastle University, Newcastle upon Tyne, N/A, United Kingdom)
Awad, Manal (University of Sharjah, Sharjah, N/A, United Arab Emirates)
Emami, Elham (Université de Montréal, McGill University, Montreal, QC, Canada)
Kimoto, Suguru (Nihon University, Matsudo, N/A, Japan)
Feine, Jocelyne (McGill University, Montreal, QC, Canada)

Does mandibular condition affect prosthetic treatment success in edentulous elders?

J Dent. 2010 Nov;38(11):899-907. doi: 10.1016/j.jdent.2010.08.002. Epub 2010 Aug 11.

Does mandibular edentulous bone height affect prosthetic treatment success?

Pan S[1], Dagenais M, Thomason JM, Awad M, Emami E, Kimoto S, Wollin SD, Feine JS.

Author information

1 Department of Prosthodontics, Peking University, School and Hospital of Stomatology, Beijing, China.

Abstract

OBJECTIVES: The aim of this study is to determine whether mandibular bone height affects patients' ratings of satisfaction and function with mandibular 2-implant overdentures (IODs) and conventional dentures (CDs).

METHODS: 214 edentulous elders were randomly allocated into 2 groups and treated with maxillary CDs and either mandibular CDs or IODs. Classifications of mandibular bone height were carried out on panoramic radiographs using 4 published methods. At baseline and 6 months after delivery, all participants rated their satisfaction with their prostheses using the McGill Denture Satisfaction Instrument. Independent t-tests and a linear multivariable regression model were used for statistical analyses.

RESULTS: Mandibular bone height has no effect on patients' ratings of general satisfaction, nor on ratings of ability to chew, stability, comfort, aesthetics and ability to speak at 6 months ($p>0.05$, linear regression). There were significant between treatment differences in ratings of general satisfaction, comfort, stability and ability to chew from all mandibular bone height categories, with higher ratings assigned to IODs ($p<0.01$, t-tests). Linear regression analyses confirmed that, for general satisfaction, as well as ability to chew, stability, comfort, aesthetics and ability to speak, treatment with IODs contributes to higher satisfaction ratings ($p<0.001$), while mandibular bone height does not.

CONCLUSIONS: The evidence demonstrates that mandibular bone height has no effect on patients' satisfaction with the function, chewing ability and comfort of their prostheses. Furthermore, no matter how much mandibular bone, these results suggest that edentulous elders will benefit more from mandibular IODs than from CDs.

Copyright © 2010 Elsevier Ltd. All rights reserved.

PMID: 20708062 DOI: 10.1016/j.jdent.2010.08.002

Effect of a Titrable Mandibular Advancement Appliance on Sleep Bruxism

Objectives: Assess the efficacy and safety of a reinforced titratable mandibular advancement appliance (MAA) on sleep bruxism (SB) activity in comparison to a mandibular occlusal splint (MOS), in order to offer an alternative to patients presenting concomitant SB and respiratory disorders during sleep.
Methods: Twelve subjects (mean age ± SEM: 26.0±1.5) with frequent SB participated in a short term (3 blocs of 2 weeks each) randomized crossover controlled study. Subjects were recorded in the sleep laboratory for 5 nights. After habituation and baseline nights, 3 more nights were spent with a MAA (Silencer Professional) either in a slightly (25%) or pronounced (75%) mandibular advancement positions, or with the control condition, the MOS. Patient self reports of pain intensity and oral appliance comfort were evaluated on 100 mm visual analogue scale (VAS). Appliance preference was also noted. Repeated measures ANOVA, Friedman and Wilcoxon signed rank tests were used for statistics.

Results: The mean number of SB episodes/hr was reduced by 39% and 47% from baseline values with the MAA at a protrusion of 25% and 75% respectively ($p<0.04$). No difference between the 2 MAA positions was noted. The MOS slightly reduced the number of SB episodes/hr without reaching statistical significance (34%, $p=0.07$). None of the SB subjects experienced any MAA breakage. With both types of oral appliance, transient tooth sensitivity to bite pressure was reported in the morning by half of the participants. All SB subjects preferred the MOS over the MAA and one subject was unable to tolerate the MAA at the 75% protrusion.

Conclusions: Short term use of MAA is associated with a significant reduction of SB motor activity without any appliance breakage in SB patients. Reinforced MAA design may be an alternative to patients with concomitant tooth grinding and snoring or apnea during sleep.

Division: IADR/CADR General Session
Meeting: 2008 IADR/CADR General Session (Toronto, Ontario, Canada)
Location: Toronto, Ontario, Canada
Year: 2008
Final Presentation ID: 71

Authors

- Rompré, Pierre H. (Université de Montréal, Montreal, QC, Canada)
- Landry-schönbeck, Anaïs (Université de Montréal, Montreal, QC, Canada)
- De Grandmont, Pierre (Université de Montréal, Montreal, QC, Canada)
- Lavigne, Gilles J. (Université de Montréal, Montreal, QC, Canada)

SESSION INFORMATION
Oral Session
Bruxism: Associations, Measurement, and Treatment
07/02/2008

Rompré, Pierre H. (Université de Montréal, Montreal, QC, Canada)
Landry-schönbeck, Anaïs (Université de Montréal, Montreal, QC, Canada)
De Grandmont, Pierre (Université de Montréal, Montreal, QC, Canada)
Lavigne, Gilles J. (Université de Montréal, Montreal, QC, Canada)

Effect of a Titrable Mandibular Advancement Appliance on Sleep Bruxism

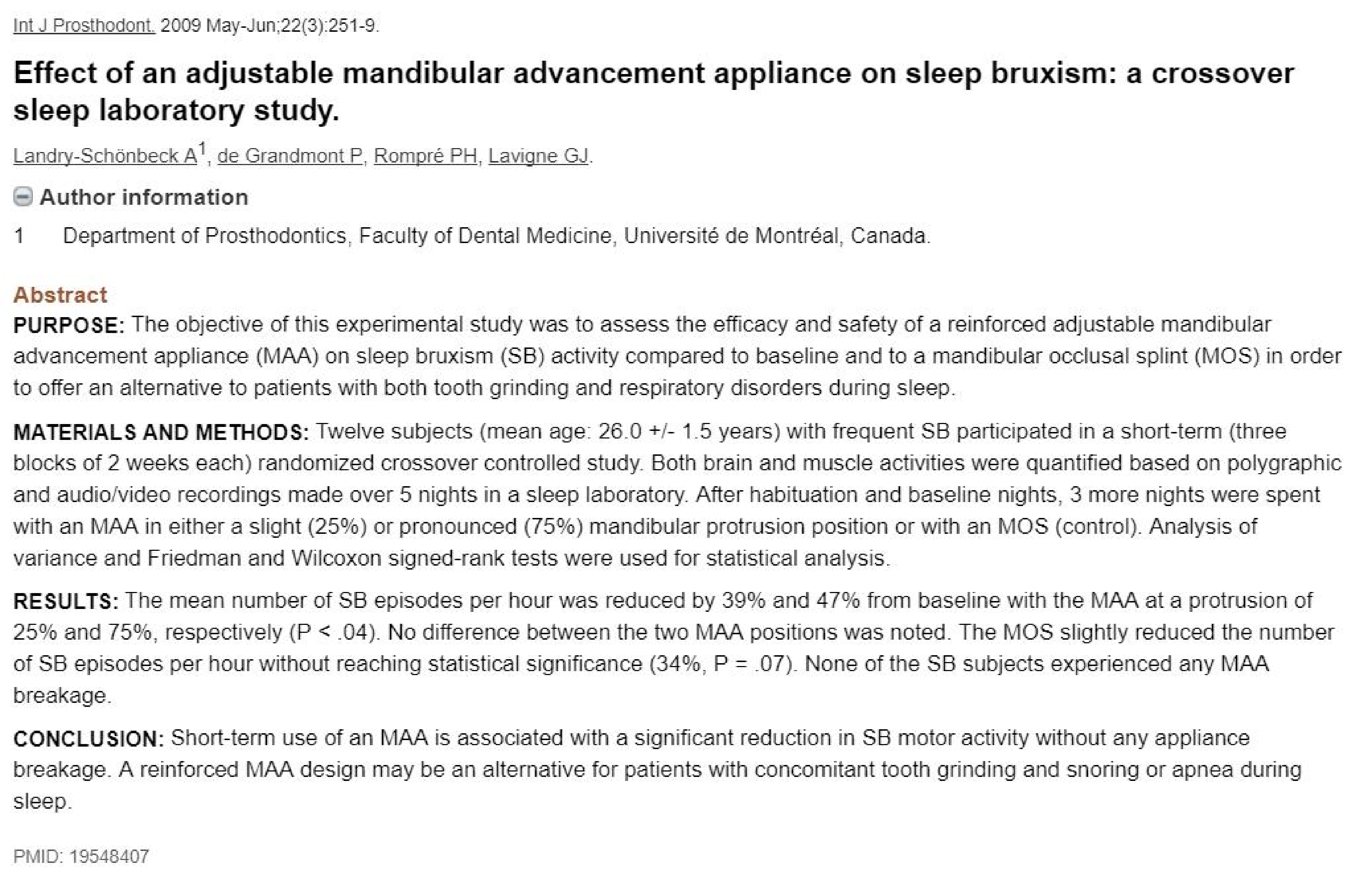

Int J Prosthodont. 2009 May-Jun;22(3):251-9.

Effect of an adjustable mandibular advancement appliance on sleep bruxism: a crossover sleep laboratory study.

Landry-Schönbeck A[1], de Grandmont P, Rompré PH, Lavigne GJ.

Author information

1 Department of Prosthodontics, Faculty of Dental Medicine, Université de Montréal, Canada.

Abstract

PURPOSE: The objective of this experimental study was to assess the efficacy and safety of a reinforced adjustable mandibular advancement appliance (MAA) on sleep bruxism (SB) activity compared to baseline and to a mandibular occlusal splint (MOS) in order to offer an alternative to patients with both tooth grinding and respiratory disorders during sleep.

MATERIALS AND METHODS: Twelve subjects (mean age: 26.0 +/- 1.5 years) with frequent SB participated in a short-term (three blocks of 2 weeks each) randomized crossover controlled study. Both brain and muscle activities were quantified based on polygraphic and audio/video recordings made over 5 nights in a sleep laboratory. After habituation and baseline nights, 3 more nights were spent with an MAA in either a slight (25%) or pronounced (75%) mandibular protrusion position or with an MOS (control). Analysis of variance and Friedman and Wilcoxon signed-rank tests were used for statistical analysis.

RESULTS: The mean number of SB episodes per hour was reduced by 39% and 47% from baseline with the MAA at a protrusion of 25% and 75%, respectively ($P < .04$). No difference between the two MAA positions was noted. The MOS slightly reduced the number of SB episodes per hour without reaching statistical significance (34%, $P = .07$). None of the SB subjects experienced any MAA breakage.

CONCLUSION: Short-term use of an MAA is associated with a significant reduction in SB motor activity without any appliance breakage. A reinforced MAA design may be an alternative for patients with concomitant tooth grinding and snoring or apnea during sleep.

PMID: 19548407

Effect of microwave disinfection in the treatment of denture stomatitis

Effect of microwave disinfection in the treatment of denture stomatitis

Objective: The aim of this in-vivo study was to identify the most effective frequency of microwave disinfection to reduce *Candida* colonization on complete dentures from patients with denture stomatitis. **Methods:** Twenty nine patients with denture stomatitis were instructed to scrub their dentures with coconut soap and water four times a day and soak it in water overnight. Patients were randomly divided into three groups. Control group (CG): patients received topical antifungal medication (Nystatin oral suspension 100, 000 UI/mL) 4x/daily for 14-days. Test group 1 (TG1): patients had their maxillary denture immersed in water and microwaved (650W/3min) 1x/week for 14-days. Test group 2 (TG2): patients had their maxillary denture immersed in water and microwaved (650W/3min) 3x/week for 14-days. Mycological samples were taken from the tissue side of the upper dentures for each patient before treatment (day-0), after starting treatment (15-days) and follow-up (30, 60, 90-days). Samples were cultured in CHROMagar′ s plates and incubated (30°C, 5-days). *Candida* spp. colonies were quantified (cfu/mL) and submitted to identification by micro-cultivation in lamina, hypertonic Sabouraud broth and bioMérieux 32C. Data were analyzed using Kruskall-Wallis test (α=0.05). **Results:** Patients in CG, TG1 and TG2 presented a decrease in colonization of *Candida* species on complete dentures after treatment, when compared with pretreatment results. The mean percentage reductions of *Candida* colonization in CG, TG1 and TG2 were 64.61, 67.77, 69.75%, respectively. There were no significant differences in the reduction of *Candida* counts in the follow-up (30, 60, 90-days) treatment of denture stomatitis when the three groups were compared. There were no significant changes in the species of *Candida* that colonized complete dentures before treatment, after starting treatment and follow-up. **Conclusion:** Microwaving dentures appeared to be useful for decreasing the *Candida* spp. colonies present in complete dentures from patients with denture stomatitis.

Grant: FAPESP 05/03211-6 and 05/04695-7.

Location: Toronto, Ontario, Canada
Year: 2008
Final Presentation ID: 1276

Authors

- Silva, Mariana Montenegro (Araraquara Dental School, Sao Paulo State University-UNESP, Araraquara, N/A, Brazil)
- Vergani, Carlos Eduardo (Araraquara Dental School, Sao Paulo State University-UNESP, Araraquara, N/A, Brazil)
- Mima, Ewerton Garcia Oliveira (Araraquara Dental School, Sao Paulo State University-UNESP, Araraquara, N/A, Brazil)
- Pavarina, Ana Cláudia (Araraquara Dental School, Sao Paulo State University-UNESP, Araraquara, N/A, Brazil)
- Machado, Ana Lúcia (Araraquara Dental School, Sao Paulo State University-UNESP, Araraquara, N/A, Brazil)
- Giampaolo, Eunice Teresinha (Araraquara Dental School, Sao Paulo State University-UNESP, Araraquara, N/A, Brazil)

SESSION INFORMATION
Poster Session
Periodontal Health/Plaque Development and Plaque Control
07/03/2008

Silva, Mariana Montenegro (Araraquara Dental School, Sao Paulo State University-UNESP, Araraquara, N/A, Brazil)
Vergani, Carlos Eduardo (Araraquara Dental School, Sao Paulo State University-UNESP, Araraquara, N/A, Brazil)
Mima, Ewerton Garcia Oliveira (Araraquara Dental School, Sao Paulo State University-UNESP, Araraquara, N/A, Brazil)
Pavarina, Ana Cláudia (Araraquara Dental School, Sao Paulo State University-UNESP, Araraquara, N/A [illegible]

Effect of microwave disinfection in the treatment of denture stomatitis

Oral Surg Oral Med Oral Pathol Oral Radiol. 2012 Oct;114(4):469-79. doi: 10.1016/j.oooo.2012.05.006.

Comparison of denture microwave disinfection and conventional antifungal therapy in the treatment of denture stomatitis: a randomized clinical study.

Silva MM[1], Mima EG, Colombo AL, Sanitá PV, Jorge JH, Massucato EM, Vergani CE.

Author information

1 School of Dentistry, CESMAC University Center, Maceió, Brazil.

Abstract

OBJECTIVE: The aim of this study was to compare the effectiveness of denture microwave disinfection and antifungal therapy on treatment of denture stomatitis.

STUDY DESIGN: Sixty denture wearers with denture stomatitis (3 groups; n = 20 each), were treated with nystatin or denture microwave disinfection (1 or 3 times/wk) for 14 days. Mycologic samples from palates and dentures were quantified and identified with the use of Chromagar, and clinical photographs of palates were taken. Microbiologic and clinical data were analyzed with the use of a series of statistical tests ($\alpha = .05$).

RESULTS: Both treatments similarly reduced clinical signs of denture stomatitis and growth on palates and dentures at days 14 and 30 ($P > .05$). At sequential appointments, the predominant species ($P < .01$) isolated was C. albicans (range 98%-53%), followed by C. glabrata (range 22%-12%) and C. tropicalis (range 25%-7%).

CONCLUSIONS: Microwave disinfection, at once per week for 2 treatments, was as effective as topical antifungal therapy for treating denture stomatitis.

Copyright © 2012 Elsevier Inc. All rights reserved.

PMID: 22986242 DOI: 10.1016/j.oooo.2012.05.006

Glutamate-Induced Temporomandibular Joint Pain is Partially Mediated by NMDA

Glutamate-Induced Temporomandibular Joint Pain is Partially Mediated by NMDA Receptors

Objectives:

To determine if injection of glutamate into the healthy human temporomandibular joint (TMJ) evokes pain through peripheral NMDA receptors and if sex or sex-steroid hormones influences this pain.

Methods:

Fifty-one healthy individuals were included; 15 males (median age = 27 years) and 36 females (age = 26 years). Double-blind intraarticular injections of 0.2 mL glutamate (1.0 mol/L) or 0.2 mL glutamate + ketamine (10 mmol/L) were performed in a randomized order into the TMJ. Maximum resting TMJ pain intensity, pain duration and area under the pain curve were assessed on a continuous electronic visual analogue scale (0-10) before and during 25 min after each injection. Venous blood samples were obtained and analyzed for serum levels of estradiol, progesterone and testosterone.

Results:

Glutamate evoked a TMJ resting pain of a median duration of 8 min. The median maximum resting pain intensity was 4.6 and the median area under the curve was 59 AU. Females reported a significantly higher maximum pain intensity than males ($p = 0.048$). There was no significant influence of gender or sex-steroid hormone levels.

In the males, co-injection of the NMDA antagonist ketamine significantly reduced the area under the pain curve to 57% ($p = 0.024$) whereas in the females, co-injection with ketamine significantly reduced the maximum pain intensity, duration and area under the curve to 90%, 65% and 54%, respectively ($p = 0.026$, $p = 0.010$ and $p < 0.001$). The effect of ketamine was not significantly related to sex-steroid hormone levels in either gender.

Conclusions:

Glutamate evokes pain in the TMJ, partially via the peripheral NMDA receptor. The degree of NMDA receptor modulation of glutamate-induced pain seems to be larger in females although no sex hormone-related effect was found. Supported by National Institute of Dental and Craniofacial Research Grant 1 R01 DE15420-01.

Division: IADR/CADR General Session
Meeting: 2008 IADR/CADR General Session (Toronto, Ontario, Canada)
Location: Toronto, Ontario, Canada
Year: 2008
Final Presentation ID: 169

Authors

- Alstergren, Per (Karolinska Institutet, Huddinge, N/A, Sweden)
- Ernberg, Malin (Karolinska Institutet, Huddinge, N/A, Sweden)
- Hajati, Anna-kari (Karolinska Institutet, Huddinge, N/A, Sweden)
- Nilsson, Mikael (Karolinska Institutet, Huddinge, N/A, Sweden)
- Sessle, Barry (University of Toronto, Toronto, ON, Canada)
- Kopp, Sigvard (Karolinska Institutet, Huddinge, N/A, Sweden)

Alstergren, Per (Karolinska Institutet, Huddinge, N/A, Sweden)
Ernberg, Malin (Karolinska Institutet, Huddinge, N/A, Sweden)
Hajati, Anna-kari (Karolinska Institutet, Huddinge, N/A, Sweden)
Nilsson, Mikael (Karolinska Institutet, Huddinge, N/A, Sweden)
Sessle, Barry (University of Toronto, Toronto, ON, Canada)

SESSION INFORMATION

Oral Session

Keynote Address and Thirty Years of Orofacial Pain Research

07/03/2008

Glutamate-Induced Temporomandibular Joint Pain is Partially Mediated by NMDA Receptors

J Orofac Pain. 2010 Spring;24(2):172-80.

Glutamate-induced temporomandibular joint pain in healthy individuals is partially mediated by peripheral NMDA receptors.

Alstergren P[1], Ernberg M, Nilsson M, Hajati AK, Sessle BJ, Kopp S.

Author information

Abstract

AIM: To determine if glutamate injected into the healthy temporomandibular joint (TMJ) evokes pain through peripheral N-methyl-D-aspartate (NMDA) receptors and if such pain is influenced by sex or sex steroid hormones.

METHODS: Sixteen healthy men and 36 healthy women were included and subjected to two randomized and double-blind intra-articular injections of the TMJ. Experimental TMJ pain was induced by injection of glutamate (1.0 mol/L) and NMDA block was achieved by co-injection of the NMDA antagonist ketamine (10 mmol/L). The TMJ pain intensity in the joint before and during a 25-minute postinjection period was continuously recorded on an electronic visual analog scale (0 to 10). Estradiol, progesterone, and testosterone levels in serum were analyzed.

RESULTS: Glutamate-induced pain showed a median (25/75 percentile) duration of 8.3 (5.2/12.2) minutes. The peak pain intensity was 6.1 (4.2/8.2), the time to peak was 50 (30/95) seconds, and the area under the curve was 59 (29/115) arbitrary units. The women reported higher maximum pain intensity than the men and shorter time to peak. The sex hormone levels were not significantly related to the glutamate-induced TMJ pain. NMDA block significantly reduced the glutamate-induced TMJ pain, mainly in the women. There were no significant correlations between sex hormone levels and the effects of NMDA block for any pain variable.

CONCLUSION: Glutamate evokes immediate pain in the healthy human TMJ that is partly mediated by peripheral NMDA receptors in the TMJ.

RCT of a HEMA-free all-in-one adhesive in non-carious cervical lesions

RCT of a HEMA-free all-in-one adhesive in non-carious cervical lesions

Objectives: One-step self-etch adhesives are the most recent generation of adhesives introduced onto the market. The objective of this randomized controlled clinical trial was to test the hypothesis that a one-step self-etch adhesive performs equally well as a conventional three-step etch&rinse adhesive (gold standard).
Methods: Two-hundred and sixty-seven non-carious cervical lesions in fifty-two patients were restored with Gradia Direct Anterior (GC, Tokyo, Japan). These composite restorations were either bonded with the HEMA-free 'all-in-one' adhesive G-Bond (GC, Tokyo, Japan) or with the three-step etch&rinse adhesive Optibond FL (Kerr, CA, USA). The restorations so far have been evaluated after 6 and 12 months clinical service regarding retention, marginal adaptation, microleakage, caries occurrence and sensitivity. Retention loss, severe marginal defects and/or discoloration that needed intervention (repair or replacement) and occurrence of caries were considered as clinical failures. A Logistic regression analysis with generalized estimating equations (GEE) was applied to factor in the clustered data (multiple lesions per patient).

Results: The recall rate at 1 year was 98%. The statistical analysis revealed a relatively low patient factor, indicating that supplementary information could be obtained from the additional restorations per patient. The retention rate for G-Bond was 98.5% compared to 99.3% for Optibond FL due to loss of two and one restoration, respectively. There were no significant differences between both adhesives regarding the evaluated parameters except for the higher occurrence of incisal marginal defects with G-Bond. These defects, however, were small and clinically irrelevant.

Conclusions: Regarding short-term performance, it was concluded that the simplified one-step adhesive G-Bond and the three-step Optibond FL are clinically equally successful, even though both were characterized by progressive degradation of marginal adaptation and G-Bond exhibited more small enamel marginal defects.

This study was supported by the Toshio Nakao Chair. The author was granted a PhD-fellowship of the Research Foundation–Flanders.

Division: IADR/CADR General Session
Meeting: 2008 IADR/CADR General Session (Toronto, Ontario, Canada)
Location: Toronto, Ontario, Canada
Year: 2008
Final Presentation ID: 1779

Authors

- Van Landuyt, Kirsten L. (Catholic University of Leuven, Leuven, N/A, Belgium)
- Fieuws, Steffen (Catholic University of Leuven, Leuven, N/A, Belgium)
- Peumans, Marleen (Catholic University of Leuven, Leuven, N/A, Belgium)
- De Munck, Jan (Catholic University of Leuven, Leuven, N/A, Belgium)
- Cardoso, Marcio Vivan (Catholic University of Leuven, Leuven, N/A, Belgium)
- Ermis, R. Banu (Suleyman Demirel University, Faculty of Dentistry, Isparta, N/A, Turkey)
- Lambrechts, Paul (Leuven BIOMAT Research Cluster, Catholic University of Leuven, Leuven, N/A, Belgium)
- Van Meerbeek, Bart (Catholic University of Leuven, Leuven, N/A, Belgium)

Van Landuyt, Kirsten L. (Catholic University of Leuven, Leuven, N/A, Belgium)
Fieuws, Steffen (Catholic University of Leuven, Leuven, N/A, Belgium)
Peumans, Marleen (Catholic University of Leuven, Leuven, N/A, Belgium)
De Munck, Jan (Catholic University of Leuven, Leuven, N/A, Belgium)
Cardoso, Marcio Vivan (Catholic University of Leuven, Leuven, N/A, Belgium)
Ermis, R. Banu (Suleyman Demirel University, Faculty of Dentistry, Isparta, N/A, Turkey)
Lambrechts, Paul (Leuven BIOMAT Research Cluster, Catholic University of Leuven, Leuven, N/A, Belgium)
Van Meerbeek, Bart (Catholic University of Leuven, Leuven, N/A, Belgium)

SESSION INFORMATION
Poster Session
Clinical Performance of Dental Adhesives
07/04/2008

RCT of a HEMA-free all-in-one adhesive in non-carious cervical lesions

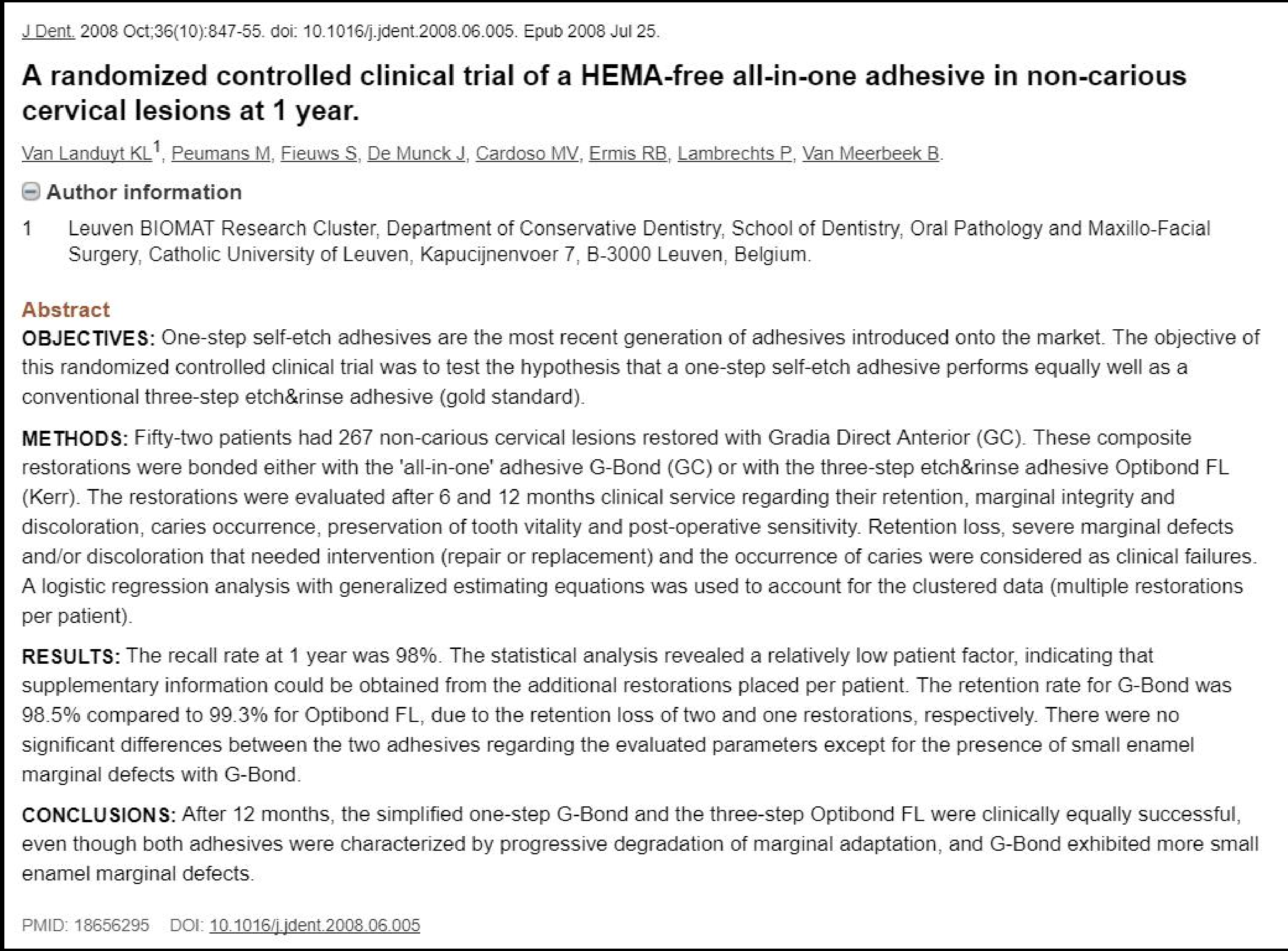
J Dent. 2008 Oct;36(10):847-55. doi: 10.1016/j.jdent.2008.06.005. Epub 2008 Jul 25.

A randomized controlled clinical trial of a HEMA-free all-in-one adhesive in non-carious cervical lesions at 1 year.

Van Landuyt KL[1], Peumans M, Fieuws S, De Munck J, Cardoso MV, Ermis RB, Lambrechts P, Van Meerbeek B.

Author information

1 Leuven BIOMAT Research Cluster, Department of Conservative Dentistry, School of Dentistry, Oral Pathology and Maxillo-Facial Surgery, Catholic University of Leuven, Kapucijnenvoer 7, B-3000 Leuven, Belgium.

Abstract

OBJECTIVES: One-step self-etch adhesives are the most recent generation of adhesives introduced onto the market. The objective of this randomized controlled clinical trial was to test the hypothesis that a one-step self-etch adhesive performs equally well as a conventional three-step etch&rinse adhesive (gold standard).

METHODS: Fifty-two patients had 267 non-carious cervical lesions restored with Gradia Direct Anterior (GC). These composite restorations were bonded either with the 'all-in-one' adhesive G-Bond (GC) or with the three-step etch&rinse adhesive Optibond FL (Kerr). The restorations were evaluated after 6 and 12 months clinical service regarding their retention, marginal integrity and discoloration, caries occurrence, preservation of tooth vitality and post-operative sensitivity. Retention loss, severe marginal defects and/or discoloration that needed intervention (repair or replacement) and the occurrence of caries were considered as clinical failures. A logistic regression analysis with generalized estimating equations was used to account for the clustered data (multiple restorations per patient).

RESULTS: The recall rate at 1 year was 98%. The statistical analysis revealed a relatively low patient factor, indicating that supplementary information could be obtained from the additional restorations placed per patient. The retention rate for G-Bond was 98.5% compared to 99.3% for Optibond FL, due to the retention loss of two and one restorations, respectively. There were no significant differences between the two adhesives regarding the evaluated parameters except for the presence of small enamel marginal defects with G-Bond.

CONCLUSIONS: After 12 months, the simplified one-step G-Bond and the three-step Optibond FL were clinically equally successful, even though both adhesives were characterized by progressive degradation of marginal adaptation, and G-Bond exhibited more small enamel marginal defects.

PMID: 18656295 DOI: 10.1016/j.jdent.2008.06.005

Relationships Between Facial Form and Alveolar Ridge Height

OBJECTIVES: The purpose of this study was to determine the relationships between facial form and mandibular and maxillary edentulous alveolar ridge height.
METHODS: A randomized controlled clinical trial was undertaken to treat patients with either mandibular conventional (CD) or implant-assisted overdentures (IOD). Post-insertion cephalometric radiographs were assessed for 25 subjects with CD and 44 with IOD. The radiographs were evaluated using Dolphin software (Dolphin 10.0-Dolphin Imaging, Chatsworth CA.). Selected reference values were used for determination of facial form and skeletal classification.

Maxillary alveolar ridge height was determined by the perpendicular bisection of the palatal plane (ANS-PNS) with the most inferior point on the maxillary anterior ridge. Mandibular alveolar ridge height was determined by the perpendicular bisection of the mandibular plane (Go-Gn) with the uppermost point on the symphysis. The ratio of maxillary to mandibular height was calculated. Multivariate analysis of variance (MANOVA) was used to compare the ridge height measures between the 3 facial form categories. Additional univariate analyses and post-hoc tests were performed following the omnibus test.

RESULTS: Significant differences in ridge height were found between facial forms (MANOVA, $F=5.79$; $p<0.001$). Univariate analyses showed significant difference in maxillary ridge height ($F=13.83$, $p<0.0001$), but no significant differences were found for mandibular ridge height or the calculated ratio of maxillary to mandibular heights ($p>0.05$). Post-hoc comparisons (REGW) indicated the maxillary ridge height for the brachyfacial group ($p<0.05$) was significantly less than for mesofacial and dolicofacial groups. No difference in maxillary ridge height was found between mesofacial and dolicofacial groups ($p>0.05$).

CONCLUSIONS: The maxillary alveolar resorptive ridge pattern was found to be significantly reduced for edentulous patients with a diagnosed brachyfacial facial profile as compared to patients with a mesofacial or dolicofacial facial form. Further evaluation of these relationships is indicated for clinical impact and relationship with available treatment modalities.

Division: IADR/CADR General Session
Meeting: 2008 IADR/CADR General Session (Toronto, Ontario, Canada)
Location: Toronto, Ontario, Canada
Year: 2008
Final Presentation ID: 912

Authors

- Nakamura, Camille (University of Southern California, Los Angeles, CA, USA)
- Ochiai, Kent T. (UCLA School of Dentistry, Santa Ana, CA, USA)
- Ikeda, Hideki (Baylor College of Dentistry, Dallas, TX, USA)
- Garrett, Neal (UCLA School of Dentistry, Los Angeles, CA, USA)

Nakamura, Camille (University of Southern California, Los Angeles, CA, USA)
Ochiai, Kent T. (UCLA School of Dentistry, Santa Ana, CA, USA)
Ikeda, Hideki (Baylor College of Dentistry, Dallas, TX, USA)
Garrett, Neal (UCLA School of Dentistry, Los Angeles, CA, USA)

SESSION INFORMATION
Poster Session
Removable Prosthodontics
07/03/2008

J Prosthet Dent. 2011 Apr;105(4):256-65. doi: 10.1016/S0022-3913(11)60041-3.

Impact of facial form on the relationship between conventional or implant-assisted mandibular dentures and masticatory function.

Ochiai KT[1], Hojo S, Nakamura C, Ikeda H, Garrett NR.

Author information

Abstract

STATEMENT OF PROBLEM: It is not clear if the interaction of craniofacial form with type of prosthetic restoration (conventional or implant-assisted) is related to masticatory function in complete denture patients.

PURPOSE: The purpose of this study was to investigate the relationships among facial form, skeletal class, alveolar residual ridge heights and masticatory function in subjects treated with implant-assisted or conventional mandibular dentures with lateral cephalometric evaluation.

MATERIAL AND METHODS: Data from a previously reported randomized controlled clinical trial were accessed to compare treatment success rates, functional and perceptual outcomes, dietary intake, and craniofacial relationships between mandibular complete dentures and implant overdentures in edentulous diabetic subjects. Evaluation of the digitized post-insertion lateral cephalometric radiographs provided measures of facial form (mesocephalic "medium", brachycephalic "broad and square", dolichocephalic "vertical and long"), skeletal class (Class I, Class II, Class III), and alveolar ridge height (mm), which were compared to results of standardized masticatory tests as evaluated using MANOVA and REGW post-hoc evaluation ($\alpha=.05$).

RESULTS: Masticatory performance on the preferred side was slightly reduced in the dolichocephalic group, compared to brachycephalic and mesocephalic groups ($P=.085$). Swallowing threshold performance was significantly less in skeletal Class II subjects compared to Class I ($P=.034$). Maxillary residual alveolar ridge height was significantly less in the brachycephalic group compared to the dolichocephalic group ($P<.001$). No differences in mandibular ridge height were seen associated with facial form or skeletal class groups.

CONCLUSIONS: Facial form may be related to masticatory function with conventional and implant-assisted mandibular dentures, but larger controlled studies are needed to confirm this relationship. Alveolar ridge height is reduced in edentulous subjects with a brachycephalic facial form.

Copyright © 2011 The Editorial Council of the Journal of Prosthetic Dentistry. Published by Mosby, Inc. All rights reserved.

PMID: 21458651 DOI: 10.1016/S0022-3913(11)60041-3

Results with Overdentures Retained by One or Two Implants

Results with Overdentures Retained by One or Two Implants

Objectives: This randomized clinical trial tested the hypothesis that there is no difference in patient satisfaction with mandibular overdentures retained by one or two implants. We also compared component costs, treatment time and maintenance time.
Methods: The study was supported by CIHR (Grant #58954), ITI Foundation (Grant #222) and Straumann Canada. Subjects wearing complete dentures were randomly assigned to receive either a single midline implant or two implants (Solid Screw, SLA surface, Straumann Canada) in the anterior mandible. They indicated satisfaction with their dentures on visual analogue scales (VAS) at baseline and one year after their lower dentures were relined to incorporate implant retention (Straumann ITI Gold Matrix). We recorded surgical and prosthodontic times, as well as component costs.

Results: We enrolled 86 subjects (39 women and 37 men, mean age 67), one of whom withdrew after receiving two implants. At one-year follow-up, median satisfaction was 93 in the single-implant group (VAS max=100) and 94 with two implants (Wilcoxon-Mann-Whitney test, $p > 0.5$). Median improvement in satisfaction in both groups was similar (about 44 points) and significant (signed-rank test, $p < 0.001$). Time for prosthodontic maintenance was also similar for both groups ($p > 0.1$). On the other hand, the single-implant group had lower component costs, and lower mean and median times for surgery, post-surgical denture maintenance, and denture reline for implants; these differences were statistically significant (each $p < 0.02$) and clinically important (post-surgical maintenance time averaged 186 vs. 268 minutes). Three implants failed, all before denture reline and all in the two-implant group.

Conclusion: One-year results indicate comparable satisfaction and maintenance time, with lower component costs and treatment times, when mandibular overdentures are retained by a single implant. This procedure should be considered as an alternative to the standard two-implant overdenture for maladaptive denture patients.

Division: IADR/CADR General Session
Meeting: 2008 IADR/CADR General Session (Toronto, Ontario, Canada)
Location: Toronto, Ontario, Canada
Year: 2008
Final Presentation ID: 2973

Authors

- Walton, Joanne N. (University of British Columbia, Vancouver, BC, Canada)
- Macentee, Michael I. (University of British Columbia, Vancouver, BC, Canada)

Walton, Joanne N. (University of British Columbia, Vancouver, BC, Canada)
Macentee, Michael I. (University of British Columbia, Vancouver, BC, Canada)

SESSION INFORMATION
Oral Session
Clinical Studies
07/05/2008

Results with Overdentures Retained by One or Two Implants

Int J Prosthodont. 2009 Jul-Aug;22(4):331-9.

A randomized clinical trial comparing patient satisfaction and prosthetic outcomes with mandibular overdentures retained by one or two implants.

Walton JN[1], Glick N, Macentee MI.

Author information

1 Department of Oral Health Sciences, University of British Columbia, Vancouver, British Columbia, Canada. jnwalton@interchange.ubc.ca

Abstract

PURPOSE: This randomized clinical trial tested hypotheses that there are no differences in patient satisfaction, component costs, or treatment and maintenance times when mandibular overdentures are retained by one or two implants.

MATERIALS AND METHODS: Subjects wearing conventional complete dentures were randomized to receive either one midline or two bilateral mandibular implants followed by a mandibular denture reline to incorporate implant retention. They indicated on a visual analog scale satisfaction with their dentures before implants and at 2 months and 1 year after implant retention. Satisfaction outcomes between the two groups were compared using the Wilcoxon/Mann-Whitney nonparametric rank test, while changes within each group were analyzed using signed-rank tests. Component costs and times for surgery, prosthodontic treatment, and maintenance were compared using nonparametric and t tests.

RESULTS: Eighty-six subjects enrolled in this study and 85 completed the 1-year follow-up, at which median satisfaction was 93 (maximum 100) in the single-implant group and 94 in the two-implant group ($P > .5$). Within each group, median improvement in satisfaction was similarly dramatic (approximately 44) and significant ($P < .001$). Prosthodontic maintenance time was similar for both groups ($P > .37$), but the single-implant group had significantly lower component costs ($P < .001$) and lower times for surgery ($P = .002$), postsurgical denture maintenance ($P = .021$), and denture reline ($P < .001$). Five implants failed in four subjects, all in the two-implant group and all before denture reline.

CONCLUSION: Lower component costs and treatment times, with comparable satisfaction and maintenance time over the first year, indicate that a mandibular overdenture retained by a single midline implant may be an alternative to the customary two-implant overdenture for maladaptive denture patients.

PMID: 19639067

Rigid vs. flexible endodontic posts: 5-year results of a RCT

Rigid vs. flexible endodontic posts: 5-year results of a RCT

Objective: to compare the clinical survival rate of 'rigid' titanium and more 'flexible' glass fiber reinforced endodontic posts. All posts were adhesively placed with a self-adhesive resin cement.
Methods: 98 patients with a treatment need of postendodontic restoration were assessed for eligibility. 91 patients met the selection criteria, were randomized and allocated to two intervention groups. 45 patients were treated using titanium posts, and 46 patients received glass fiber posts. All posts had a diameter of 1.4mm and a length of 13mm, were cemented with self-adhesive universal resin cement and composite core build-ups were placed. A circumferential ferrule of 2mm was prepared in all cases, if necessary by means of surgical crown lengthening. Patients were observed at 3, 6, 12 month after post placement, and annually thereafter for a total of 5 years.

Results: Half of all restored teeth received single cast crowns, while in 23% fixed partial dentures and in 11% combined fixed-removable partial dentures were placed. 10% of all restored teeth were molars. One patient was excluded from the analysis since no final restoration was placed. One tooth was extracted due to changes of prosthetic treatment planning. One tooth was extracted for periodontal reasons. One patient was lost to follow-up. One abutment tooth for a cantilever bridge fractured where a glass fiber post was inserted. An intention-to-treat analysis revealed no statistically significant differences between groups (p=0.553, Fisher's Exact test).

Conclusion: Adhesively luted titanium and glass fiber reinforced endodontic posts may have similarly low failure rates over 5 years of clinical service if a 2mm ferrule is provided.

Division: IADR/CADR General Session
Meeting: 2008 IADR/CADR General Session (Toronto, Ontario, Canada)
Location: Toronto, Ontario, Canada
Year: 2008
Final Presentation ID: 1607

Authors

- Naumann, M. (CC3, CBF, Charité-Universitätsmedizin Berlin, Berlin, N/A, Germany)
- Franke, Alexandra (CC3, CBF, Charité-Universitätsmedizin Berlin, Berlin, N/A, Germany)
- Dietrich, Thomas (The School of Dentistry, University of Birmingham, Birmingham, N/A, United Kingdom)
- Sterzenbach, Guido (CC3, CBF, Charité-Universitätsmedizin Berlin, Berlin, N/A, Germany)

SESSION INFORMATION
Oral Session
Survival and Clinical Performance of Restorations including Endodontic Posts
07/04/2008

Naumann, M. (CC3, CBF, Charité-Universitätsmedizin Berlin, Berlin, N/A, Germany)
Franke, Alexandra (CC3, CBF, Charité-Universitätsmedizin Berlin, Berlin, N/A, Germany)
Dietrich, Thomas (The School of Dentistry, University of Birmingham, Birmingham, N/A, United Kingdom)
Sterzenbach, Guido (CC3, CBF, Charité-Universitätsmedizin Berlin, Berlin, N/A, Germany)

Rigid vs. flexible endodontic posts: 5-year results of a RCT

J Endod. 2012 Dec;38(12):1557-63. doi: 10.1016/j.joen.2012.08.015.

Rigid versus flexible dentine-like endodontic posts--clinical testing of a biomechanical concept: seven-year results of a randomized controlled clinical pilot trial on endodontically treated abutment teeth with severe hard tissue loss.

Sterzenbach G[1], Franke A, Naumann M.

Author information

1 Department of Prosthodontics, Geriatric Dentistry and Craniomandibular Disorders, Charité-Universitätsmedizin Berlin, Berlin, Germany. guido.sterzenbach@charite.de

Abstract

INTRODUCTION: This is the first clinical long-term pilot study that tested the biomimetic concept of using more flexible, dentine-like (low Young modulus) glass fiber-reinforced epoxy resin posts (GFREPs) compared with rather rigid, stiff (higher Young modulus) titanium posts (TPs) in order to improve the survival rate of severely damaged endodontically treated teeth.

METHODS: Ninety-one subjects in need of postendodontic restorations in teeth with 2 or less remaining cavity walls were randomly assigned to receive either a tapered TP (n = 46) or a tapered GFREP (n = 45). The posts were adhesively luted using self-adhesive resin cement. The composite core build-ups were prepared ensuring a circumferential 2-mm ferrule. The primary endpoint was a loss of restoration for any reason. To study group differences, the log-rank test was calculated ($P < .05$). Hazard plots were constructed.

RESULTS: After 84 months of observation (mean = 71.2 months), 7 restorations failed (ie, 4 GFREPs and 3 TPs). The failure modes were as follows: GFREP:root fracture (n = 3), core fracture (n = 1) and TP:endodontic failure (n = 3). No statistical difference was found between the survival rates (GFREPs = 90.2%, TPs = 93.5%, P = .642). The probability of no failure was comparable for both post materials (risk ratio; 95% confidence interval, 0.965-0.851/1.095).

CONCLUSIONS: When using self-adhesive luted prefabricated posts in severely destroyed abutment teeth with 2 or less cavity walls and a 2-mm ferrule, postendodontic restorations achieved high long-term survival rates irrespective of the post material used (ie, glass fiber vs titanium).

Copyright © 2012 American Association of Endodontists. Published by Elsevier Inc. All rights reserved.

PMID: 23146637 DOI: 10.1016/j.joen.2012.08.015

Self-Adhesive Composite Versus Zinc-Oxide-Phosphate Luting Cements: A Prospective

Self-Adhesive Composite Versus Zinc-Oxide-Phosphate Luting Cements: A Prospective Clinical Trial

Objectives: to compare the clinical outcomes of metal-based fixed partial dentures luted conventionally with zinc-phosphate or self-adhesive resin cement.
Materials and methods: Forty-nine patients (mean age 54 +/-13 years) received 49 metal-based fixed partial dentures randomly luted using zinc-phosphate (ZPC) (Richter&Hoffmann, G) or self-adhesive resin cement (Rely X Unicem (RXU) encapsulated, 3M Espe, G) at the University Medical Center of Regensburg. There were two onlays, 42 posterior and 5 anterior crowns. Forty-seven restorations were made of precious alloys, 2 of non-precious alloys. The restorations were examined immediately after cementation and then every year. The clinical performance was checked according plaque (0-5; PI, Quigley -Hein)- bleeding- (0-4; PBI; Mühlemann) and attachment scores. The examination included pulp vitality and pain percussion tests. Statistics: means of scores, std.dev. cumulative survival and complication rates were calculated using life tables.

Results: The mean observation time was 3.16 +/-0.6 years (min: 2.0; max: 4.5 years). During that time no restoration was lost, no re-cementation became necessary. One endodontic treatment was performed at the self-adhesive composite group after 2.9 years. Bleeding (1.44 RXU vs. 1.25 ZOC) and plaque (1.64 RXU vs. 1.0 ZPC) scores showed no statistically significant difference. Pulp vitality and pain percussion tests were not different.

Conclusion: The self-adhesive- resin cement RelyX Unicem performed clinically as well and can be used as easily as zinc phosphate cement to retain metal-based restorations over a 38-month observation period.

Division: IADR/CADR General Session
Meeting: 2008 IADR/CADR General Session (Toronto, Ontario, Canada)
Location: Toronto, Ontario, Canada
Year: 2008
Final Presentation ID: 2346

Authors

- Wimmer, Jutta (University of Regensburg, Regensburg, N/A, Germany)
- Behr, Michael (University of Regensburg, Regensburg, N/A, Germany)
- Rosentritt, Martin (University of Regensburg, Regensburg, N/A, Germany)
- Kolbeck, Carola (University of Regensburg, Regensburg, N/A, Germany)
- Handel, Gerhard (University of Regensburg, Regensburg, N/A, Germany)

SESSION INFORMATION
Poster Session
Clinical Evaluation of Ceramic Restorations, Crown Restorations, and Endodontic Treatment
07/04/2008

Wimmer, Jutta (University of Regensburg, Regensburg, N/A, Germany)
Behr, Michael (University of Regensburg, Regensburg, N/A, Germany)
Rosentritt, Martin (University of Regensburg, Regensburg, N/A, Germany)
Kolbeck, Carola (University of Regensburg, Regensburg, N/A, Germany)
Handel, Gerhard (University of Regensburg, Regensburg, N/A, Germany)

Self-Adhesive Composite Versus Zinc-Oxide-Phosphate Luting Cements: A Prospective Clinical Trial

Dent Mater. 2009 May;25(5):601-4. doi: 10.1016/j.dental.2008.11.003. Epub 2008 Dec 18.

Self-adhesive resin cement versus zinc phosphate luting material: a prospective clinical trial begun 2003.

Behr M[1], Rosentritt M, Wimmer J, Lang R, Kolbeck C, Bürgers R, Handel G.

Author information

1 Department of Prosthodontics, University Medical Center Regensburg, 93042 Regensburg, Germany. michael.behr@klinik.uni-regensburg.de

Abstract

OBJECTIVES: The literature demonstrates that conventional luting of metal-based restorations using zinc phosphate cements is clinically successful over 20 years. This study compared the clinical outcomes of metal-based fixed partial dentures luted conventionally with zinc phosphate and self-adhesive resin cement.

METHODS: Forty-nine patients (mean age 54+/-13 years) received 49 metal-based fixed partial dentures randomly luted using zinc phosphate (Richter & Hoffmann, Berlin, Germany) or self-adhesive resin cement (RelyX Unicem Aplicap, 3M ESPE, Germany) at the University Medical Center Regensburg. The core build-up material was highly viscous glass ionomer; the finishing line was in dentin. The study included 42 posterior, 5 anterior crowns and two onlays. Forty-seven restorations were made of precious alloys, 2 of non-precious alloys. The restorations were clinically examined every year. The clinical performance was checked for plaque (0-5; PI, Quigley-Hein), bleeding (0-4; PBI; Mühlemann) and attachment scores. The examination included pulp vitality and percussion tests.

STATISTICS: Means of scores, standard deviation, cumulative survival and complication rates were calculated using life tables.

RESULTS: The mean observation time was 3.16+/-0.6 years (min: 2.0; max: 4.5 years). During that time no restoration was lost, no recementation became necessary. One endodontic treatment was performed in the self-adhesive composite group after 2.9 years. At study end bleeding (1.44 RelyX Unicem vs. 1.25 zinc phosphate) and plaque (1.64 RelyX Unicem vs. 1.0 zinc phosphate) scores showed no statistically significant difference.

SIGNIFICANCE: The self-adhesive resin cement performed clinically as well and can be used as easily as zinc phosphate cement to retain metal-based restorations over a 38-month observation period.

PMID: 19100611 DOI: 10.1016/j.dental.2008.11.003

Twenty-six-Year Assessment of Anterior Resin Restorations -Two Restorative

Twenty-six-Year Assessment of Anterior Resin Restorations -Two Restorative Procedures

Objectives: The aim of this randomized clinical study was to compare the long-term quality and longevity of anterior resin restorations completed with two restorative procedures.

Methods: The material consisted of 52 pairs of Class III restorations made in 35 patients in the microfilled resin Silar® using two modifications of the conventional acid-etch restorative procedure: A) bevelling of cavity margin, and B) cavity treatment with a dentin adhesive NPG-GMA in ethanol and re-etching and coating of the finished restorations with low viscous resin. The restorations were examined at baseline and after 2, 4, 6, 11 and 26 years. Information concerning repair and replacement of restorations were collected from the patients' records through their general practitioners. Data were processed by nonparametric statistics for paired observations and Kaplan-Meier survival analyses.

Results: After 26 years were 54 restorations clinically controlled, and 28 restorations were assessed through the dental records. 7 patients had died, and 2 patients did not want to participate in the final folllow-up. 25 A and 21 B restorations had been replaced ($p>0.05$).

The remaining restorations showed one or more failures, but none of them were in need of replacement. The cumulative survival rates were almost congruent during the whole observation period with a median survival time of 23 years for A restorations and 25 years for B restorations ($p>0.05$).

Conclusions: The study showed that it was possible to perform Class III restorations with a high clinical quality and a long durability using the materials and methods available in the beginning of the 1980's, and that the restorative procedures had an insignificant influence on the clinical quality and longevity of the restorations.

Support: The restorative materials were provided by the manufacturers.

Division: IADR/CADR General Session
Meeting: 2008 IADR/CADR General Session (Toronto, Ontario, Canada)
Location: Toronto, Ontario, Canada
Year: 2008
Final Presentation ID: 1767

Authors

- Bakhshandeh, Azam (University of Copenhagen, Copenhagen, N/A, Denmark)
- Qvist, Vibeke (University of Copenhagen, Copenhagen, N/A, Denmark)
- Strøm, Carsten (N/A, Copenhagen, N/A, Denmark)

Bakhshandeh, Azam (University of Copenhagen, Copenhagen, N/A, Denmark)
Qvist, Vibeke (University of Copenhagen, Copenhagen, N/A, Denmark)
Strøm, Carsten (N/A, Copenhagen, N/A, Denmark)

SESSION INFORMATION
Poster Session
Clinical Evaluation of Resin Composite and ART Restorations
07/04/2008

Twenty-six-Year Assessment of Anterior Resin Restorations -Two Restorative Procedures

Two-year assessment of anterior resin restorations inserted with two acid-etch restorative procedures

来自Wiley

收藏　引用　批量引用　报错　分享

作者 V Qvist , C Ström , A Thylstrup

摘要 Abstract Fifty-two pairs of Class III restorations in a microfilled resin Silar were inserted in acid-etched cavities. A bevel preparation was performed along the margin of one cavity (A) whereas the other cavity was treated with the surface-active comonomer NPG-GMA before filling (B). After finishing of the restorations, the surface of the type B filling was re-etched and covered with a layer of non-composite, low-viscous resin. Following a 2-yr observation period 65 restorations were classified as "good", 33 were "adequate" and 4 were "unsatisfactory" or were replaced during the study period. The only significant difference between type A and type B restorations was marginal discolorations which most frequently were observed along the beveled type A restorations. The occurrence of such failures was increased by marginal deficiencies and occlusion/articulation on the restorations. 收起

出版源 《European Journal of Oral Sciences》 , 2010 , 93 (4) :343-350

被引量 26

2009

A Prospective, Randomized Study of Medialized vs Conventional Implant Abutments

A Prospective, Randomized Study of Medialized vs Conventional Implant Abutments

Objectives: Crestal bone loss following the restoration of a dental implant is a documented phenomenon. Recent observations suggest that less bone loss may result from placing a more medialized abutment on the implant (platform switching). The aim of this study was to measure crestal bone loss and biologic width in medialized versus same diameter abutments with the same subject.

Methods: Ten subjects had 2-4 implants placed within the same mandibular fixed partial denture. At placement implants were randomly assigned conventional or medialized (platform switched) abutments. Standardized occlusal stents were fabricated for peri-apical radiographs taken at placement, six weeks, two months, six months, one year, and two years. The radiographs were analyzed using image analysis software to measure crestal bone loss and biologic width. Data were analyzed for image analysis reproducibility and compared using a t-test and a regression model.

Results: A statistically significant difference in crestal bone loss over 2 years was observed between the conventional and the switched implants (p=0.0368; t-test), with a mean bone loss of 1.19±0.58mm and 0.99±0.53mm respectively. After a regression analysis adjusting for implant length and tooth position, a significant difference was also observed (p=<0.0001). Analyzing biologic width showed no significant difference between the conventional and switched implants (p=0.7419; t-test), with a mean biologic width of 1.53±0.78mm and 1.57±0.72mm respectively. After adjusting for implant length and tooth position, the biologic width showed no significant difference between the two groups (p=0.1996).

Conclusion: These findings suggest that crestal bone loss is less in a platform switched dental implant. The crestal bone may be preserved in platform switched implants due to the formation of a consistent biologic width which allows more space laterally for the connective tissue attachment. Future studies should include more subjects and multiple clinical centers.

Supported by NIH Grant 5 T35 HL007473

Division: IADR/AADR/CADR General Session
Meeting: 2009 IADR/AADR/CADR General Session (Miami, Florida)
Location: Miami, Florida
Year: 2009
Final Presentation ID: 186

Authors

- Trammell, Kyle H. (University of Alabama, Birmingham, AL, USA)
- Reddy, Michael S. (University of Alabama, Birmingham, AL, USA)
- Geurs, Nicolaas C. (University of Alabama, Birmingham, AL, USA)
- O'neal, Jean, S. (University of Alabama, Birmingham, AL, USA)
- Liu, Perng-ru (University of Alabama, Birmingham, AL, USA)
- Hsu, Yung Tsung (University of Alabama, Birmingham, AL, USA)
- Mcneal, Sandre F. (University of Alabama, Birmingham, AL, USA)

SESSION INFORMATION
Oral Session
Implant Prosthodontics and Immediate Loading
04/02/2009

Trammell, Kyle H. (University of Alabama, Birmingham, AL, USA)
Reddy, Michael S. (University of Alabama, Birmingham, AL, USA)
Geurs, Nicolaas C. (University of Alabama, Birmingham, AL, USA)
O'neal, Jean, S. (University of Alabama, Birmingham, AL, USA)
Liu, Perng-ru (University of Alabama, Birmingham, AL, USA)
Hsu, Yung Tsung (University of Alabama, Birmingham, AL, [illegible]

A Prospective, Randomized Study of Medialized vs Conventional Implant Abutments

Int J Periodontics Restorative Dent. 2009 Dec;29(6):599-605.

A prospective, randomized, controlled comparison of platform-switched and matched-abutment implants in short-span partial denture situations.

Trammell K[1], Geurs NC, O'Neal SJ, Liu PR, Haigh SJ, McNeal S, Kenealy JN, Reddy MS.

Author information

1 University of Alabama at Birmingham School of Dentistry, Birmingham, Alabama 35294-0007, USA.

Abstract

Recent observations suggest that less bone loss may result from placing a more medialized abutment on an implant (platform switching). The objective of this study was to measure the radiographic crestal bone loss and biologic width around conventional and platform-switched implants. Implants were randomly assigned into conventional or switched categories within the same prosthesis. Twenty-five implants were placed and observed in the mandibles of 10 patients for 2 years. A regression analysis demonstrated a significant difference between groups (P < or = .0001). These findings suggest that less crestal bone loss occurs around a platform-switched dental implant versus a conventional implant.

PMID: 20072737

A Study of Denture Adhesive in Well-Fitting Dentures

A Study of Denture Adhesive in Well-Fitting Dentures

Objectives:
The study aim was to compare objective and subjective performance measures of well fitting dentures with and without denture adhesive.

Methods:

This was a single center, randomized, blinded, 4 arm crossover study to compare 3 denture adhesive formulas (Super Poligrip® Original, Super Poligrip® Free, Super Poligrip® Comfort Seal Strips, GSK Consumer Healthcare, LP) with no adhesive. Thirty-six subjects with well fitting and well made full upper and lower conventional dentures completed the study. The determination of fit was made using Kapur criteria (Olshan Modification, Retention and Stability Index Sum Score $\geqslant$6). Dentures were judged to be well made using criteria that included vertical dimension, freeway space, occlusal relationships and border extensions. Standard test methods for bite force, food occlusion and masticatory efficacy were used. Subjects also rated confidence, comfort, satisfaction and denture wobble in conjunction with the food occlusion and mastication tests. Mixed models were used in the analysis of these parameters with period and treatment as fixed effects and subject as a random effect. Pair-wise Dunnett adjustment of multiple comparisons were performed at 5% level to compare each of the three denture adhesive formulas to no adhesive.

An oral soft tissue exam was performed at baseline and after all study procedures were completed.

Results:

The 3 denture adhesives significantly ($p<0.05$) improved bite force, masticatory efficacy and retention and stability of well fitting dentures. Significant ($p<0.05$) increases in subjective ratings of confidence, comfort and decreases in denture wobble were associated with use of adhesive. There was significant ($p<0.05$) improvement in satisfaction ratings for cream adhesives. All adhesives were well tolerated.

Conclusions:

[illegible]ures. The results of this study are in agreement with published studies that also demonstrate the advantages

Division: IADR/AADR/CADR General Session
Meeting: 2009 IADR/AADR/CADR General Session (Miami, Florida)
Location: Miami, Florida
Year: 2009
Final Presentation ID: 422

Authors

- Gendreau, Linda (GlaxoSmithKline Consumer Healthcare, Parsippany, NJ, USA)
- Shanga, Gilbert (GlaxoSmithKline Consumer Healthcare, Parsippany, NJ, USA)
- Munoz, Carlos (State University of New York - Buffalo, Buffalo, NY, USA)
- Durocher, John (GlaxoSmithKline Consumer Healthcare, Parsippany, NJ, USA)
- Fernandez, Patricia (GlaxoSmithKline Consumer Healthcare, Parsippany, NJ, USA)
- Loewy, Zvi (GlaxoSmithKline Consumer Healthcare, Parsippany, NJ, USA)

SESSION INFORMATION
Poster Session
Removable Prosthodontics
04/02/2009

Gendreau, Linda (GlaxoSmithKline Consumer Healthcare, Parsippany, NJ, USA)
Shanga, Gilbert (GlaxoSmithKline Consumer Healthcare, Parsippany, NJ, USA)
Munoz, Carlos (State University of New York - Buffalo, Buffalo, NY, USA)
Durocher, John (GlaxoSmithKline Consumer Healthcare, Parsippany, NJ, USA)
Fernandez, Patricia (GlaxoSmithKline Consumer Healthcare, Parsippany, NJ, USA)

A Study of Denture Adhesive in Well-Fitting Dentures

J Prosthodont. 2012 Feb;21(2):123-9. doi: 10.1111/j.1532-849X.2011.00795.x. Epub 2011 Nov 6.

A clinical study to evaluate denture adhesive use in well-fitting dentures.

Munoz CA[1], Gendreau L, Shanga G, Magnuszewski T, Fernandez P, Durocher J.

Author information

1 University at Buffalo School of Dental Medicine (SUNY), Buffalo, NY, USA.

Abstract

PURPOSE: The objective of this study was the assessment of retention and stability and functional benefits of denture adhesive applied to well-fitting and well-made dentures.

MATERIALS AND METHODS: This was a randomized, crossover study to compare two marketed denture adhesives (test cream, Super Poligrip® Free, and test strip, Super Poligrip® Comfort Seal Strips) and an unmarketed cream adhesive (GlaxoSmith Kline Consumer Healthcare) with no adhesive as the negative control. Thirty-six subjects completed the study. One hour after the application of denture adhesive, retention and stability were measured using the Kapur Index and maxillary incisal bite force. Two hours after application, functional tests were used to assess denture movement and peanut particle migration under the denture. Subjects also rated confidence, comfort, satisfaction with dentures, and denture wobble in conjunction with the functional tests.

RESULTS: Denture adhesives significantly ($p < 0.05$) improved retention and stability of well-fitting dentures. Subjects experienced significantly ($p < 0.05$) fewer dislodgements while eating an apple after adhesive was applied to dentures. Significant ($p < 0.05$) increases in subjective ratings of confidence and comfort as well as decreases in denture wobble were associated with the use of adhesive. There was significant ($p < 0.05$) improvement in satisfaction ratings for cream adhesives. A single application of each denture adhesive was well tolerated.

CONCLUSION: The results of this study provide evidence that use of Super Poligrip® denture adhesives can enhance aspects of performance of complete well-fitting dentures as well as provide increased comfort, confidence, and satisfaction with dentures.

© 2011 by the American College of Prosthodontists.

PMID: 22054033 DOI: 10.1111/j.1532-849X.2011.00795.x

Clinical Evaluation of a Self-Etching Adhesive After Two Years

Objectives: The purpose of this prospective randomized clinical study was to compare the clinical performance of the new self-etching adhesive system AdheSE One in combination with the composite Tetric Evo Ceram and the influence of the additional application of the flowable resin composite Tetric Flow after two years. Methods: In 50 patients 25 class I and 75 class II cavities were placed with at least two restorations per patient. The adhesive system AdheSE One was used for all the restorations. In one of the two fillings in each patient, an additional layer of the flowable resin composite Tetric Flow was applied in the entire cavity and separately light-cured. The fillings were placed under rubber dam. All materials were used as recommended by the manufacturer. Two clinicians evaluated the restorations at baseline, two week following placement, and at the six month recall visit according to the modified clinical criteria of Ryge. For this sensitivity, hypersensitivity, marginal discoloration, marginal adaption, recurrent caries, surface, color match, proximal contact and filling integrity were considered. All data were analyzed by Man-Whitney-U-test. Results: After two years 44 patients could be re-examined. All teeth remained vital and did not show any signs of postoperative sensitivity. Marginal adaption code Bravo could be evaluated in seven fillings (four with flowable liner, three without). In three teeth a filling integrity was scored as Bravo (two with and one without fowable liner). None of the teeth showed signs of secondary caries. Statistical analysis showed no significant difference between techniques for any of the evaluation criteria ($p>0.05$, Man-Whitney-U-test). Conclusions: After two years the use of a flowable composite showed no significant impact on the clinical performance of class-I and -II restorations. The self-etch adhesive AdheSE One might be a promising alternative to other systems. This study was supported by Ivoclar Vivadent, Germany.

Division: IADR/AADR/CADR General Session
Meeting: 2009 IADR/AADR/CADR General Session (Miami, Florida)
Location: Miami, Florida
Year: 2009
Final Presentation ID: 550

Authors

- Kobler, Annett (Martin-Luther-University Halle-Wittenberg, Halle, N/A, Germany)
- Schaller, Hans-guenter (Martin-Luther-University Halle-Wittenberg, Halle, N/A, Germany)
- Gernhardt, Christian Ralf (Martin-Luther-University Halle-Wittenberg, Halle, N/A, Germany)

SESSION INFORMATION
Poster Session
Adhesives
04/02/2009

Kobler, Annett (Martin-Luther-University Halle-Wittenberg, Halle, N/A, Germany)
Schaller, Hans-guenter (Martin-Luther-University Halle-Wittenberg, Halle, N/A, Germany)
Gernhardt, Christian Ralf (Martin-Luther-University Halle-Wittenberg, Halle, N/A, Germany)

Clinical Evaluation of a Self-Etching Adhesive After Two Years

International Poster Journal of Dentistry and Oral M
QUINTESSENCE PUBLISHING

Int Poster J Dent Oral Med 12 (2010), No. 1 15. Mar. 2010

Int Poster J Dent Oral Med 2010, Vol 12 No 1, Poster 478

Clinical Evaluation of a Self-Etching Adhesive After Two Years

Language: English

Authors:
Dr. Annett Kobler, Prof. Dr. Hans-Guenter Schaller, PD Dr.Christian Ralf Gernhardt,
Department of Operative Dentistry and Periodontology, Martin-Luther-University Halle-Wittenl

Introduction

Due to the characteristic polymerization shrinkage of resin-based composites, clinical success with composite restorative materials is dependent on effective and durable adhesion to enamel and dentin (1). Flowable resin composites have been reported to adapt well to the cavity wall (2). This optimal adaption may result in an improvement of the adhesive performance of resin composites (2-4). Moreover, a number of new self-etch adhesives have been developed to simplify clinical bonding procedure. The efficiency of these simplified bonding systems is still controversia (5).

Objectives

The purpose of this prospective randomized clinical study was to compare the clinical performance of the new self-etching adhesive system AdheSE One (Fig. 1) in combination with the composite Tetric Evo Ceram (Fig. 2) and the influence of the additional application of the flowable resin composite Tetric Flow (Fig. 2) after two years.

Material and Methods

In 50 patients 32 class I and 68 class II cavities were placed with at least two restorations per patient. The adhesive system AdheSE One was used for all the restorations: An adequate amount of AdheSE One was directly applied to the cavity. Starting with the enamel portion, all cavity surfaces were thoroughly coated for 30 seconds. Excess amounts of AdheSE One were dispersed with a strong stream of air until there was no longer any movement of the material. Then, AdheSE One was polymerized for 10 seconds at a light intensity of more than 500 mW/cm2 (bluephase, Ivoclar Vivadent)(Fig. 3-7).
In one of the two fillings in each patient, an additional layer of the flowable resin composite Tetric Flow was applied in the entire cavity and separately light-cured. The fillings were placed under rubber dam. All materials were used as recommended by the manufacturer. Two clinicians evaluated the restorations at baseline, two week following placement, and at the six month, one and two year recall visit according to the modified clinical criteria of Ryge (sensitivity, hypersensitivity, marginal discoloration, marginal adaption, recurrent caries, surface, color match, proximal contact, filling integrity).
For each of the criteria, Alpha was used to indicate the highest degree of clinical acceptability; Beta to Delta were used to indicate progressively lessening degrees of clinical acceptability. The thermic test for sensibility was done by using a cold stimulus (Endofrost, Roeko, Langenau,Germany). In addition, each restoration was photographed at each recall. Statistical analysis was based on Man-Whitney-U-test using SPSS 12.0. The test was carried out at 95% confidence level.

Results

After two years 44 patients could be re-examined. All teeth remained vital and did not show any signs of postoperative sensitivity. Marginal adaption code Bravo could be evaluated in seven fillings (four with flowable liner, three without). In three teeth a filling integrity was scored as Bravo (two with and one without fowable liner). None of the teeth showed signs of secondary caries. Statistical analysis showed no significant difference between techniques for any of the evaluation criteria ($p>0.05$, Man-Whitney-U-test)(Fig. 8-15).

Conclusions

After two years the use of a flowable composite showed no significant impact on the clinical performance of class-I and -II restorations. The self-etch adhesive AdheSE One might be a promising alternative to other systems.

Clinical performance of cervical restoration with potassium oxalate-based desensitizing

Clinical performance of cervical restoration with potassium oxalate-based desensitizing

Objectives: to evaluate the clinical performance and post-operative sensitivity of non-carious cervical restoration with and without the use of a potassium oxalate-based desensitizing agent after 18 months. Methods: Upon IRB approval and informed consent 144 non-carious cervical lesions were selected and randomly divided in four groups: in Group I (GI) the teeth were restored using a potassium oxalate-based desensitizing agent (BisBlock, Bisco) after acid etching and before the application of Adper Single Bond 2 (3M ESPE). In Group II (GII) the teeth were restored using the same adhesive system used in GI, without the use of desensitizing agent. Group III (GIII) was restored in the same way of GI, but using One-Step (Bisco) as the adhesive system. Group IV (GIV) was restored with the same adhesive of GIII, but without the application of BisBlock. Filtek Supreme (3M ESPE) was used to restore GI and GII, and Aelite (Bisco) was used to restore GIII and GIV. All 144 restorations were evaluated after 1 week, 2, 6, 12 and 18 months according to the modified USPHS criteria. McNemar test was used to identify anterations in the alfa index within groups throughout the evaluated periods. Qui-square test was used to detect interactions among groups in each period. Results: revealed no statistical differences between groups restored with or without the use of a desensitizing agent regarding induced and reported sensitivity. After 18 months, retention rates showed to be statistically lower for One-Step than Adper SingleBond 2.
Conclusion: the use of potassium oxalate-based desensitizing agent did not decreased post-operative sensitivity neither improved the clinical performance.

Division: IADR/AADR/CADR General Session
Meeting: 2009 IADR/AADR/CADR General Session (Miami, Florida)
Location: Miami, Florida
Year: 2009
Final Presentation ID: 1880

Authors

- Sartori, Neimar (Universidade Federal De Santa Catarina, Florianópolis, N/A, Brazil)
- Lopes, Gc (Universidade Federal De Santa Catarina, Florianópolis, N/A, Brazil)
- Vieira, L. C. C. (Universidade Federal De Santa Catarina, Florianópolis, N/A, Brazil)

Sartori, Neimar (Universidade Federal De Santa Catarina, Florianópolis, N/A, Brazil)
Lopes, Gc (Universidade Federal De Santa Catarina, Florianópolis, N/A, Brazil)
Vieira, L. C. C. (Universidade Federal De Santa Catarina, Florianópolis, N/A, Brazil)

SESSION INFORMATION
Poster Session
Caries, Sealants, Desensitizers, Special Topics
04/03/2009

Clinical performance of cervical restoration with potassium oxalate-based desensitizing

Clinical Performance of Cervical Restorations with Desensitizing Agents: 18-month Clinical Trial

Neimar Sartori[a] / Guilherme Carpena Lopes[b] / Luiz Clovis Cardoso Vieira[b]

Purpose: To evaluate the clinical performance and postoperative sensitivity of noncarious Class V restorations with and without the use of a potassium oxalate-based desensitizing agent over a period of 18 months.

Materials and Methods: One hundred forty cervical lesions (40 patients) were selected and randomly divided into four groups: group 1 (G1) – teeth restored with the application of a potassium oxalate-based desensitizing agent (BisBlock) after acid etching and before the application of the adhesive Adper Single Bond 2; group 2 (G2) – teeth restored using the same adhesive system used in G1, without the use of any desensitizing agent; group 3 (G3) – similar to G1, but using the adhesive One-Step; group 4 (G4) – similar to G3, but without the application of BisBlock. All restorations were evaluated (double blind) after 1 week and 2, 6, 12, and 18 months according to the modified USPHS criteria. The McNemar and chi-square tests were used to analyze the results ($p \leq 0.05$).

Results: There were no statistical differences between groups restored with or without the use of a desensitizing agent for postoperative sensitivity. After 18 months, retention rates showed to be statistically significantly lower for One-Step than Adper Single Bond 2.

Conclusion: The use of potassium oxalate-based desensitizing agent did not decrease postoperative sensitivity when it was used under composite resin restorations.

Keywords: clinical evaluation, dentin desensitizer, potassium oxalate, dentin hypersensitivity, noncarious cervical lesions.

J Adhes Dent 2011;13:xxpages *Submitted for publication: ????; accepted for publication: ???*

Color stability of reline resin after microwave disinfection: clinical evaluation

Color stability of reline resin after microwave disinfection: clinical evaluation

Objective: The purpose of this study was to evaluate the effect of microwave disinfection on the color stability of hard chairside reline resin after 180 days service period. Methods: Forty adult patients, who required denture reline treatment, participated in this study. Tokuyama Rebase II was used to reline complete maxillary dentures. Adaptation of each denture was examined and the surface to be relined was reduced by means of a rotary cutting instrument. The relining material was spatulated, poured into the relining area, inserted and adjusted after setting. The edentulous subjects were randomly divided into 2 groups (n=20) and dentures were cleansed according to two methods: CG (control group)- brushing with coconut soap and soft toothbrush; DG (disinfection group)- brushing according to previous methods and microwave disinfection for 3min at 650W. Color parameters in L*a*b* were recorded with a spectrophotometer. Each relined denture was subjected to base line evaluation and after 7, 15, 30, 90 and 180 days. Based on the L*a*b* values the color difference (ΔE) was calculated. Data were analyzed using Mann-Whitney and Kruskal-Wallis tests (P=0.05). Results: The reline material showed color changes (ΔE) from 0.64 to 3.21. The results demonstrated that the color change values of disinfection group were significantly lower than those of control group after each one of the time periods tested (P<0.05). When comparing the results of the control group, it can be seen that the color change values were not significantly different after all use periods of the complete denture (P>0.05). The results also revealed that no significant differences were found among the periods of use of relined denture for disinfection group (P>0.05). Conclusion: The color of Tokuyama Rebase II was not affected after 180 days observation period. The microwave disinfection produced higher color stability for the tested material.

Grants: FAPESP-05/02194-0 and 05/04693-4

Division: IADR/AADR/CADR General Session
Meeting: 2009 IADR/AADR/CADR General Session (Miami, Florida)
Location: Miami, Florida
Year: 2009
Final Presentation ID: 1755

Authors

- Ribeiro, Roberta Chuqui (São Paulo State University (UNESP), Araraquara Dental School, Araraquara, N/A, Brazil)
- Izumida, Fernanda Emiko (São Paulo State University (UNESP), Araraquara Dental School, Araraquara, N/A, Brazil)
- Moffa, Eduardo Buozi (São Paulo State University (UNESP), Araraquara Dental School, Araraquara, N/A, Brazil)
- Basso, Michael Frederico Manzolli (São Paulo State University (UNESP), Araraquara Dental School, Araraquara, N/A, Brazil)
- Giampaolo, Eunice Teresinha (São Paulo State University (UNESP), Araraquara Dental School, Araraquara, N/A, Brazil)
- Vergani, Carlos Eduardo (São Paulo State University (UNESP), Araraquara Dental School, Araraquara, N/A, Brazil)

SESSION INFORMATION
Poster Session
Color/Restorative Materials
04/03/2009

Ribeiro, Roberta Chuqui (São Paulo State University (UNESP), Araraquara Dental School, Araraquara, N/A, Brazil)
Izumida, Fernanda Emiko (São Paulo State University (UNESP), Araraquara Dental School, Araraquara, N/A, Brazil)
Moffa, Eduardo Buozi (São Paulo State University (UNESP), Araraquara Dental School, Araraquara, N/A, Brazil)
Basso, Michael Frederico Manzolli (São Paulo State University (UNESP), Araraquara Dental School, [illegible]

Am J Dent. 2011 Aug;24(4):200-4.

Color stability of chemically activated reline resin after microwave disinfection: a 1-year clinical trial.

Ribeiro RC[1], Giampaolo ET, Izumida FE, Pavarina AC, Moffa EB, Vergani CE.

Author information

1 Araraquara Dental School - UNESP, R. Humaitá, no 1680, CEP 14801-903 Araraquara, São Paulo, Brazil. robertachuqui@yahoo.com.br

Abstract

PURPOSE: To evaluate the effect of microwave disinfection on the color stability of a hard chairside reline resin after a 1-year service period.

METHODS: 40 adult patients aged between 30-75 years, who required denture reline treatment, participated in this study. Tokuyama Rebase II was used to reline complete maxillary dentures. The edentulous subjects were randomly divided into two groups (n=20) and dentures were cleansed according to two methods: CG (control group) - brushing with coconut soap and soft toothbrush; DG (disinfection group) - brushing according to previous methods and microwave disinfection once a week for 3 minutes at 650W. Color parameters in L*a*b* were recorded by spectrophotometer immediately after the reline, at 7 and 15 days, 1, 3, 6 and 9 months and 1 year post-placement. Data were analyzed by two-way repeated-measures ANOVA and Tukey tests (alpha = 0.05).

RESULTS: Color alteration values of DG were significantly lower than those of CG (P<0.05). Color changes observed after 15 days were greater than values obtained at 7 days recall (P<0.05). All color changes observed for the CG were considered noticeable (between 1.5 and 3.0 NBS). In DG, color change was slight (between 0.5 and 1.5 NBS). There were statistically significant differences between L* values obtained initially and after 3 months, between 15 days and 3 months and between 15 days and 1 year (P<0.05). No significant differences were observed between group and time for the parameters a* and b*.

PMID: 22016912

Evaluation of Packable and Conventional Hybrid Composite Resins in Restorations

Evaluation of Packable and Conventional Hybrid Composite Resins in Restorations

Objectives: to compare the clinical performance of a packable and a conventional hybrid composite resins in Class I restorations for a period of 3 years using a randomized controlled double-blind clinical trial with self-matching design.
Methods: A total of 50 pairs of Class I restorations were placed in 32 adult patients in a self-macthing prospective clinical trial. The paired teeth were divided into Synergy Compact/One Coat (SC) group and TPH Spectrum/XenoIII (TS) group according to random number table. Application of the materials followed the manufacture's instructions. The restorations were evaluated by two independent evaluators using U.S. Public Health Service (USPHS)-Ryge modified criteria. Statistical analysis was performed using Wilcoxon rank sum test.

Results: After 3 years, 40 pairs of restorations were available for evaluation. Four TS and two SC restorations failed due to fracture. Only one TS restored tooth showed postoperative sensitivity at baseline and the symptom disappeared a week later. Alfa ratings of TS vs that of SC restorations were as follows: 95% vs 98% for color match, 85% vs 88% for marginal integrity, 88% vs 90% for anatomical form, 85% vs 83% for marginal discoloration, 88% vs 93% for occlusal contact. For both materials, Alfa ratings were 88% for surface texture. There were no statistically significant differences between the two restorative materials for any parameters evaluated (P>0.05).

Conclusion: The 3-year clinical performances of the two restorative materials were satisfactory and not significantly different for each of the parameters evaluated.

Clinical Trial Registration Number: 20050218013

Division: IADR/AADR/CADR General Session
Meeting: 2009 IADR/AADR/CADR General Session (Miami, Florida)
Location: Miami, Florida
Year: 2009
Final Presentation ID: 2291

Authors

- Chen, Zhi (School and Hospital of Stomatology,Wuhan University, Wuhan, N/A, China)
- Shi, Lu (School and Hospital of Stomatology,Wuhan University, Wuhan, N/A, China)
- Wang, Xuefei (School and Hospital of Stomatology,Wuhan University, Wuhan, N/A, China)
- Zhao, Qinwen (School and Hospital of Stomatology,Wuhan University, Wuhan, N/A, China)
- Zhang, Yan (School and Hospital of Stomatology,Wuhan University, Wuhan, N/A, China)
- Ji, Wei (School and Hospital of Stomatology,Wuhan University, Wuhan, N/A, China)

SESSION INFORMATION
Poster Session
Outcomes Studies and Biological Prosthodontic Research
04/03/2009

Chen, Zhi (School and Hospital of Stomatology,Wuhan University, Wuhan, N/A, China)
Shi, Lu (School and Hospital of Stomatology,Wuhan University, Wuhan, N/A, China)
Wang, Xuefei (School and Hospital of Stomatology,Wuhan University, Wuhan, N/A, China)
Zhao, Qinwen (School and Hospital of Stomatology,Wuhan University, Wuhan, N/A, China)
Zhang, Yan (School and Hospital of Stomatology,Wuhan University, Wuhan, N/A, China)
Ji, Wei (School and Hospital of Stomatology,Wuhan University, Wuhan, N/A, China)

Evaluation of Packable and Conventional Hybrid Composite Resins in Restorations

Oper Dent. 2010 Jan-Feb;35(1):11-9. doi: 10.2341/09-027CR.

Evaluation of packable and conventional hybrid resin composites in Class I restorations: three-year results of a randomized, double-blind and controlled clinical trial.

Shi L[1], Wang X, Zhao Q, Zhang Y, Zhang L, Ren Y, Chen Z.

Author information

1 Key Lab for Oral Biomedical Engineering Ministry of Education, Department of Cariology & Endodontics, School & Hospital of Stomatology, Wuhan University, Wuhan, PR China.

Abstract

The clinical performance of packable and conventional hybrid resin composites in Class I restorations for a period of three years was compared using a randomized controlled double-blind clinical trial with self-matching design. A total of 50 pairs of Class I restorations were placed in 32 adult patients by one dentist in a self-matching prospective clinical trial. The paired teeth were divided into the TPH Spectrum/XenoIII (TS) restoration group and the Synergy Compact/One Coat (SC) restoration group according to a random number table. Application of the materials followed the manufacturer's instructions. The restorations were evaluated by two independent evaluators using US Public Health Service (USPHS)-Ryge modified criteria. Statistical analysis was performed using the McNemar's test with Yates' continuity correction. After three years, 40 pairs of restorations were available for evaluation. Four TS and two SC restorations failed due to fracture. Only one TS-restored tooth showed postoperative sensitivity at baseline and the symptom disappeared one week later. Alpha ratings of TS vs SC restorations were as follows: 95% vs 98% for color match, 85% vs 88% for marginal integrity, 88% vs 90% for anatomical form, 85% vs 83% for marginal discoloration, 88% vs 93% for occlusal contact. For both materials, Alpha ratings were 88% for surface texture. The three-year clinical performances of the two restorative materials were satisfactory and not significantly different for each of the parameters evaluated.

PMID: 20166406 DOI: 10.2341/09-027CR

In vivo analysis of enamel wear against ceramic materials

In vivo analysis of enamel wear against ceramic materials

Objective: (1) To test the hypothesis that wear rates of ceramic are equivalent to the wear rates of their enamel antagonists; (2) To test the hypothesis that the wear rates of contralateral teeth are equivalent to the wear rates of ceramic crowns. Methods: Thirty six (36) teeth (from 31 patients selected) needing full coverage crowns were randomly assigned to receive either a metal-ceramic (D'Sign, Ivoclar, Vivadent) or an all-ceramic crown (IPS Empress2 or Eris EXC, Ivoclar, Vivadent). A single unit crown was fabricated from either one of two all-ceramic materials or a metal-ceramic material. A vinyl polysiloxane impression was made of the maxillary and mandibular arches to record the occlusal surfaces of the cemented crown, its antagonist tooth and its contralateral tooth, after one week, 1 year and 2 years, post cementation. Casts were poured in gypsum (GC Fujirock) and scanned using a 3D Laserscanner (Willytec, Germany). Maximum wear was calculated by superimposing the baseline one-week image with first and second year images. Results: The mean maximum wear rates for the ceramic crowns were 48.7±35.1 μm after year 1 and 58.8±36.5 μm for year 2. The mean maximum wear rates for the natural enamel antagonists were 54.9±23.6 μm for year 1 and 69.1±42.9 μm for the year 2. Teeth contralateral to the crowns exhibited a maximum wear of 42.4±23.4 μm maximum wear for the year 1 and 49.5±31.7 μm for year 2. One-way ANOVA (α = 0.05) revealed that there was no significant difference in the maximum wear rates between the ceramic crowns, their natural antagonists and their contralateral teeth. Conclusions: The new breed of ceramics is promising in that in vivo wear rates are within the range of normal enamel wear.
This study was supported by Ivoclar Vivadent.

Division: IADR/AADR/CADR General Session
Meeting: 2009 IADR/AADR/CADR General Session (Miami, Florida)
Location: Miami, Florida
Year: 2009
Final Presentation ID: 1009

Authors

- Esquivel-upshaw, Josephine (University of Florida, Gainesville, FL, USA)
- Rose, William (Univ. of TX, HSC at San Antonio, San Antonio, TX, USA)
- Oliveira, Erica R. (Univ. of TX, HSC at San Antonio, San Antonio, TX, USA)
- Anusavice, Kenneth J. (University of Florida, Gainesville, FL, USA)

Esquivel-upshaw, Josephine (University of Florida, Gainesville, FL, USA)
Rose, William (Univ. of TX, HSC at San Antonio, San Antonio, TX, USA)
Oliveira, Erica R. (Univ. of TX, HSC at San Antonio, San Antonio, TX, USA)
Anusavice, Kenneth J. (University of Florida, Gainesville, FL, USA)

SESSION INFORMATION
Poster Session
Glass Ionomer, Ceramics, Amalgam, Developing Materials
04/02/2009

In vivo analysis of enamel wear against ceramic materials

Dent Mater. 2012 Jun;28(6):615-21. doi: 10.1016/j.dental.2012.02.001. Epub 2012 Mar 10.

Three years in vivo wear: core-ceramic, veneers, and enamel antagonists.

Esquivel-Upshaw JF[1], Rose WF Jr, Barrett AA, Oliveira ER, Yang MC, Clark AE, Anusavice KJ.

Author information

1 Department of Dental Restorative Sciences, University of Florida, Gainesville, FL, USA.

Abstract

OBJECTIVES: Test the hypotheses that there are equivalent wear rates for enamel-versus-enamel and ceramic-versus-enamel, analyzing the in vivo wear of crown ceramics, their natural enamel antagonists, and the corresponding two contralateral teeth; and, that bite force does not correlate with the wear.

METHODS: A controlled, clinical trial was conducted involving patients needing full coverage crowns opposing enamel antagonists. Bite forces were measured using a bilateral gnathodynamometer. Single-unit restorations of metal/ceramic (Argedent 62, Argen Corp/IPS d.SIGN veneer); or, core-ceramic/veneer from either, Empress2/Eris, or e.max Press core/e.max Ceram glaze (ceramics: Ivoclar Vivadent, USA) were randomly assigned, fabricated and cemented. Impressions were made of the ceramic crowns, as well as each maxillary and mandibular quadrant at one week (baseline) and one, two and three years. Resulting models were scanned (3D laser scanner). Maximum wear was calculated by superimposing baseline with annual images.

RESULTS: There were a total of thirty-six crowns required for thirty-one patients. Each restoration had three associated enamel teeth: crown, (1) antagonist, (2) contralateral and (3) contralateral-antagonist. SAS PROC MIXED ($\alpha=0.05$) indicated no statistical significance for mean maximum wear among crown ceramics, enamel antagonists and contralaterals. However, enamel wear was statistically significant in relation to intraoral location ($p=0.04$) and among years ($p<0.02$). Analyzed alone, the enamel contralateral-antagonist exhibited significantly greater wear ($p<0.001$). Considering all wear sites, there was no correlation with bite force ($p=0.15$).

SIGNIFICANCE: The ceramics and their antagonists exhibited in vivo wear rates within the range of normal enamel. Future studies should examine the wear implications of the contralateral-antagonist enamel.

Copyright © 2012 Academy of Dental Materials. Published by Elsevier Ltd. All rights reserved.

PMID: 22410113 PMCID: PMC3570123 DOI: 10.1016/j.dental.2012.02.001

Longitudinal Analysis on Stability of Implants in the Posterior Maxilla

Longitudinal Analysis on Stability of Implants in the Posterior Maxilla

Objectives: To identify longitudinal differences between the stability of immediate, non-functionally loaded and delayed, functionally loaded implants placed in the posterior maxilla as determined by resonance frequency analysis (RFA). Methods: 8 patients with partially edentulous maxilla requiring 2 to 4 implants for screw retained single crowns were included in this study. Implants were randomly assigned to either the Test Group (TG immediate, non-functionally loaded) or the Control Group (CG delayed, functionally loaded). Straumann SLActive 4.1 or 4.8 mm diameter, RN or WN implants of 10 or 12 mm in length were placed (8 test and 8 control). TG received a screw- retained non-functionally loaded acrylic provisional restoration the day of surgery. Final screw-retained crowns were placed at 20 weeks post-implant placement for both TG and CG groups. RFA analysis was performed on all implants utilizing the Osstell Mentor (Integration Diagnostic, Savedalen, Sweden) at implant placement and 6, 12, and 20 weeks post-implant placement. Data was evaluated by One-Way ANOVA. Results: Relative to baseline, both groups showed an increase in ISQ. However, there were no statistically significant differences in ISQ between the groups at baseline or 6, 12 and 20 weeks after implant placement (p=0.35). Conclusion: Preliminary data showed no statistical significant difference in implant stability as measured by RFA between the test and control implants. This finding provides experimental support for the use of an immediate loading protocol on SLActive implants placed in the posterior maxilla. Supported by Straumann AG, Waldenburg, Switzerland.

Division: IADR/AADR/CADR General Session
Meeting: 2009 IADR/AADR/CADR General Session (Miami, Florida)
Location: Miami, Florida
Year: 2009
Final Presentation ID: 2875

Authors

- Atlas, Alan M. (University of Pennsylvania, Philadelphia, PA, USA)
- Ribeiro, Alexandre Lombardi (University of Pennsylvania, Philadelphia, PA, USA)
- Laudenbach, Jay (University of Pennsylvania, Philadelphia, PA, USA)
- Saleh, Najeed (University of Pennsylvania, Philadelphia, PA, USA)
- Blatz, Markus B. (University of Pennsylvania, Philadelphia, PA, USA)
- Kim, Steven (University of Pennsylvania, Philadelphia, PA, USA)
- Korostoff, Jonathan Mark (University of Pennsylvania, Philadelphia, PA, USA)

SESSION INFORMATION
Oral Session
Surgical Interventions and Clinical Investigations
04/04/2009

Atlas, Alan M. (University of Pennsylvania, Philadelphia, PA, USA)
Ribeiro, Alexandre Lombardi (University of Pennsylvania, Philadelphia, PA, USA)
Laudenbach, Jay (University of Pennsylvania, Philadelphia, PA, USA)
Saleh, Najeed (University of Pennsylvania, Philadelphia, PA, USA)
Blatz, Markus B. (University of Pennsylvania, Philadelphia, PA, USA)
Kim, Steven (University of Pennsylvania, Philadelphia, PA, USA)
Korostoff, Jonathan Mark (University of Pennsylvania, Philadelphia, PA, USA)

Clin Oral Implants Res. 2015 Feb;26(2):183-90. doi: 10.1111/clr.12310. Epub 2013 Dec 11.

Resonance frequency analysis as a predictor of early implant failure in the partially edentulous posterior maxilla following immediate nonfunctional loading or delayed loading with single unit restorations.

Kim SJ[1], Ribeiro AL, Atlas AM, Saleh N, Royal J, Radvar M, Korostoff J.

Author information

1 Department of Oral Biology, University of North Carolina at Chapel Hill School of Dentistry, Chapel Hill, NC, USA.

Abstract

OBJECTIVES: To assess the ability of baseline resonance frequency analysis (RFA) measurements to predict early implant failure in the posterior maxilla and to evaluate potential correlations between this measurement with Hounsfield units, bone quality variables, and implant dimension.

MATERIALS AND METHODS: This prospective randomized study involved 46 SLActive Straumann implants placed in the posterior maxillae of 21 subjects. Each patient received at least one control (delayed loading) and one experimental (immediate nonfunctional loading) implant. Each site was evaluated with presurgical computer-assisted tomography (CT) scans, histomorphometric analysis of bone cores, and subjective determination of bone quality. Baseline implant stability quotients (ISQ) were determined by RFA measurements made at the time of fixture placement. Pearson's correlation analysis and Spearman's test were used to identify statistically significant correlations within the resultant data. Receiver operating characteristic (ROC) analysis was used to determine whether baseline ISQ values can accurately predict early implant failure.

RESULTS: The mean baseline ISQ values for the two groups were 66.8 (experimental) and 66.2 (control). The 12-month survival rates were 86.4% (experimental) and 100% (control). There were no statistically significant correlations between baseline ISQ values and early implant failure, bone quality variables, or implant dimension. ROC analysis showed that baseline ISQ values cannot predict early implant failure.

CONCLUSION: Baseline RFA measurements were not able to predict early failure of immediately loaded implants placed in the posterior maxilla and therefore should not be used to determine whether an implant is a candidate for immediate nonfunctional loading in this region of the mouth.

© 2013 John Wiley & Sons A/S. Published by John Wiley & Sons Ltd.

KEYWORDS: immediate loading; implant stability; maxilla; resonance frequency analysis

PMID: 24325547 DOI: 10.1111/clr.12310

Management of Peri-implant Mucositis using Ozone and Hydrogen Peroxide

Management of Peri-implant Mucositis using Ozone and Hydrogen Peroxide

Peri-implant mucositis is a major clinical problem. Huth et al (Effect of aqueous ozone on the NF-kappaB system. J Dent Res. 2007 May;86 (5):451-456) have reported the potential benefits of using Ozone in periodontal treatment. As ozone is the most powerful antimicrobial agent we could use in dentistry it seemed appropriate to assess its efficacy to manage peri-implant mucositis and Ozone is synergistic with hydrogen peroxide (H_2O_2).
Objectives: To assess if ozone/H_2O_2 could manage experimentally produced peri-implant mucositis.

Methods: Twenty subjects (mean age 60) were enrolled in a randomized, double - blind, controlled, single center study after a pre-trial phase to achieve clinically healthy gingivae. Each of these patients had two implants randomly allocated as either test or control. Subgingival administration of ozone (HealOzone, KaVo), 3% H_2O_2 and saline (test implant site) or air and saline (control implant site) was delivered subgingivally for 60 seconds on day 0, 7 and 14. A Bleeding Index (BI) and a Modified Gingival Index (MGI) were recorded by a single operator at 0. 7, 14 and 21 days in this 21 day experimentally induced peri-implant mucositis study. A protective shield was worn over the 2 selected implants whilst the subjects brushed their teeth during this 21 day study.

Results: The (mean +- SE) MGI was zero at baseline and at 21 days was 0.5 +- 0.15 for the ozone/H_2O_2 treated sites and zero and 1.65 +- 0.21 respectively for the air treated sites ($p<0.01$). The BI at 0 and 21 days was 0.05 +- 0.05 and 0.05 +- 0.05 for the ozone/H_2O_2 treated sites and zero and 1.05 +- 0.18 for the air treated sites ($p<0.01$). No adverse side effects were recorded.

Conclusion: Ozone/H_2O_2 treatment of gingivae around implants significantly reduced peri-implant mucositis development during this 21 day experimentally induced peri-implant mucositis study.

Division: IADR/AADR/CADR General Session
Meeting: 2009 IADR/AADR/CADR General Session (Miami, Florida)
Location: Miami, Florida
Year: 2009
Final Presentation ID: 1978

Authors

- Mc Kenna, Dan (Queen's University of Belfast, Belfast, N/A, United Kingdom)
- Lynch, Edward (Queens University Belfast, Belfast, N/A, Northern Ireland, Uk)
- Cunningham, James Leo (Queens University Belfast, Belfast, N/A, Northern Ireland, Uk)
- Grootveld, Martin C. (Bolton University, Bolton, N/A, United Kingdom)

Mc Kenna, Dan (Queen's University of Belfast, Belfast, N/A, United Kingdom)
Lynch, Edward (Queens University Belfast, Belfast, N/A, Northern Ireland, Uk)
Cunningham, James Leo (Queens University Belfast, Belfast, N/A, Northern Ireland, Uk)
Grootveld, Martin C. (Bolton University, Bolton, N/A, United Kingdom)

SESSION INFORMATION
Poster Session
Epidemiology and Health Measurement
04/03/2009

Management of Peri-implant Mucositis using Ozone and Hydrogen Peroxide

☐ The effect of subgingival ozone and/or hydrogen peroxide on the development of peri-implant mucositis: a double-blind randomized controlled trial.
(PMID:24278915)

Abstract | Citations | Related Articles | Data | BioEntities | External Links

McKenna DF, Borzabadi-Farahani A, Lynch E

The International Journal of Oral & Maxillofacial Implants [01 Nov 2013, 28(6):1483-1489]

Type: Randomized Controlled Trial, Journal Article
DOI: 10.11607/jomi.3168

Abstract

PURPOSE: This double-blind randomized controlled trial assessed the effect of subgingival ozone (O_3, gaseous ozone, HealOzone MK II, KaVo) and/or hydrogen peroxide (H_2O_2) on the development of peri-implant mucositis. MATERIALS AND METHODS: Twenty subjects (mean age, 60 ± 7.7 years) with 80 implants (4 implants each) were recruited. First, a 2-week pretrial phase took place to achieve healthy gingiva. Subsequently, partial gum shields were constructed for the experimental area (around the 4 implants); subjects were asked to refrain from brushing in that area by wearing the gum shield. The following treatments were randomly applied (for 60 seconds) to implant sites on days 0, 7, and 14: (1) air (O_2) and saline (0.9% NaCl) (control group), (2) O_2 and H_2O_2 (3%), (3) O_3 and saline, and (4) O_3 and H_2O_2. Plaque, gingival, and bleeding indices were recorded on days 0, 7, 14, and 21. RESULTS: Significant differences were seen among the treatments ($P < .01$) in plaque ($F = 16.68$), modified gingival ($F = 7.86$), and bleeding ($F = 18.42$) indices. O_3 + saline and O_3 + H_2O_2 produced optimum gingival health scores and were equally effective and the most effective in controlling bleeding (mean score = 0.05), while O_2 + saline was the least effective (mean score = 0.56). CONCLUSION: Ozone showed great potential for management of peri-implant mucositis.

Metallic and Translucent Matrices for Class-II Composite Restorations: 4-year Follow-up

Metallic and Translucent Matrices for Class-II Composite Restorations: 4-year Follow-up

Objective: This randomized clinical study evaluated the performance of composite restorations placed with two matrix and wedge systems, 4 years after placement. Methods: Twenty-three patients were selected, and received at least two Class II restorations, one with metallic matrix and wooden wedge and the other with polyester matrix and reflective wedge. One dentist placed the 109 restorations and all cavities were restored using Single Bond and P-60 (3M ESPE) according to manufacturer's instructions. Polymerization was performed from occlusal (metallic matrices) or through the reflective wedge (polyester matrices). Restorations were evaluated at baseline, 1, 2 and 4 years after placement by the modified USPHS criteria together with interproximal radiographs, and data were analyzed with Mann-Whitney and Friedman tests (A=0.05). Results: Fifteen subjects were reassessed after 4 years. There was a significant decrease in the quality of marginal adaptation, marginal staining and roughness for both systems employed ($p<0.05$), while restorations performed with polyester matrix and reflexive wedge also presented decreased quality in color stability and occlusal and proximal contacts ($p<0.05$). However, no significant differences between matrices occurred after 4 years ($p>0.05$). Conclusions: Although the matrix and wedge systems evaluated showed similar clinical performance, the clinical quality of restorations decreased after 4 years of service.

Division: IADR/AADR/CADR General Session
Meeting: 2009 IADR/AADR/CADR General Session (Miami, Florida)
Location: Miami, Florida
Year: 2009
Final Presentation ID: 3250

Authors

- Pereira-cenci, Tatiana (Federal University of Pelotas, Pelotas, N/A, Brazil)
- André, Darvi Almeida (Federal University of Pelotas, Pelotas, N/A, Brazil)
- Barbosa, Renata Pereira Sousa (Federal University of Pelotas, Pelotas, N/A, Brazil)
- Cenci, Maximiliano Sergio (Federal University of Pelotas, Pelotas, N/A, Brazil)
- Piva, E. (Federal University of Pelotas, Pelotas, N/A, Brazil)
- Demarco, Ff (Federal University of Pelotas, Pelotas, N/A, Brazil)

SESSION INFORMATION

Poster Session

Composite and Composite-combination Restorations

04/04/2009

Pereira-cenci, Tatiana (Federal University of Pelotas, Pelotas, N/A, Brazil)
André, Darvi Almeida (Federal University of Pelotas, Pelotas, N/A, Brazil)
Barbosa, Renata Pereira Sousa (Federal University of Pelotas, Pelotas, N/A, Brazil)
Cenci, Maximiliano Sergio (Federal University of Pelotas, Pelotas, N/A, Brazil)
Piva, E. (Federal University of Pelotas, Pelotas, N/A, Brazil)
Demarco, Ff (Federal University of Pelotas, Pelotas, N/A, Brazil)

Metallic and Translucent Matrices for Class-II Composite Restorations: 4-year Follow-u[illegible]

Clin Oral Investig. 2011 Feb;15(1):39-47. doi: 10.1007/s00784-009-0362-5. Epub 2010 Jan 5.

Effects of metallic or translucent matrices for Class II composite restorations: 4-year clinical follow-up findings.

Demarco FF[1], Pereira-Cenci T, de Almeida André D, de Sousa Barbosa RP, Piva E, Cenci MS.

Author information

1 Department of Operative Dentistry, School of Dentistry, Federal University of Pelotas, Rua Gonçalves Chaves, 457, 5th floor, 96015 560 Pelotas, Rio Grande do Sul, Brazil. flavio.demarco@pq.cnpq.br

Abstract

This study evaluated the performance of composite restorations placed with two matrix and wedge systems 4 years after placement. In a split-mouth design, 23 patients were selected and received at least two class II restorations, one with metallic matrix and wooden wedge and the other with polyester matrix and reflective wedge. One dentist placed the 109 restorations, and all cavities were restored using Single Bond and P-60 (3M ESPE) according to the manufacturer's instructions. Polymerization was performed through occlusal (metallic matrices) or through the reflective wedge (polyester matrices). Restorations were evaluated and categorized as alpha (A), bravo (B), charlie (C), and delta (D; modified United States Public Health System criteria) at baseline and 4 years after placement. Both clinical aspects and interproximal radiographs were considered in the evaluation. Data were analyzed with Mann-Whitney and Friedman tests ($\alpha = 0.05$). Fifteen subjects (78 teeth/102 proximal surfaces) were reassessed after 4 years. Considering comparisons within matrices in different evaluation time points, no significant differences were observed ($p > 0.05$). Comparing 4-year to baseline results, the quality of marginal adaptation (40% and 40.4 %, score A), marginal staining (31.3% and 28.8%, score A), and roughness (56% and 46.2%, score A) decreased for metallic and translucent matrices, respectively ($p < 0.05$), while color match (9.6%, score A), occlusal contacts (75%, score A), and proximal contacts (71.7%, score A) also decreased in quality for translucent matrices ($p < 0.001$). Although the matrix and wedge systems evaluated showed similar clinical performance, there was clinical quality loss after 4 years, with most of the restorations being still acceptable, and no intervention was necessary.

PMID: 20049495 DOI: 10.1007/s00784-009-0362-5

Mini-Implants for Vertical Dimension Control in Young Adult Patients

Mini-Implants for Vertical Dimension Control in Young Adult Patients

Objectives: Vertical dimension control has always been a matter of concern in moderate to severe high angle patients showing severe proclination of the anterior teeth. Extrusion of the posterior teeth is an inevitable sequela of orthodontic treatment especially in adult patients often leading to unfavorable esthetic results. The purpose of this randomized controlled trial was to quantify the treatment results in the vertical dimension after en-masse retraction of anterior teeth with mini-implants (MIs) placed between the roots of the second premolar and first molar as anchor units in bialveolar dental protrusion patients undergoing extraction of all 4 first premolars. Methods: 40 patients (mean age 17.5±3.2 years) were randomly assigned to group 1 (G1), space closure with MIs as anchor units, or group 2 (G2), space closure with conventional methods of anchorage. Dento-skeletal and soft-tissue changes were analyzed on lateral cephalograms taken before and after space closure. Statistical analysis was performed by using the Student's paired and unpaired't tests' Results: A statistically significant decrease in the facial vertical dimensions was seen in G1, but the variables in G2 showed no significant differences ($P>0.05$). Extrusion and mesialization of the molar was noted in G2, whereas G1 showed distalization (anchorage gain) and intrusion. Facial convexity angle, nasolabial angle, and lower lip protrusion had greater changes in G1. Conclusions: The biomechanical force system in G1 provided effective control over the posterior dentoalveolar dimension so that dramatic improvement in the facial esthetics could be achieved. Significant autorotation of the mandible was noted leading to increased chin prominence. With accurate diagnosis and treatment planning MI anchorage can be considered as a possible alternative to orthognathic surgery in similar borderline cases. The authors developed a 'mathematical equation' through which the change in chin position can be accurately predicted as related to the molar intrusion.

Division: IADR/AADR/CADR General Session
Meeting: 2009 IADR/AADR/CADR General Session (Miami, Florida)
Location: Miami, Florida
Year: 2009
Final Presentation ID: 273

Authors

- Upadhyay, Madhur (University of Connecticut, Farmington, CT, USA)
- Nanda, Ravindra (University of Connecticut, Farmington, CT, USA)

Upadhyay, Madhur (University of Connecticut, Farmington, CT, USA)
Nanda, Ravindra (University of Connecticut, Farmington, CT, USA)

SESSION INFORMATION
Oral Session
Imaging, Miniscrews
04/02/2009

Am J Orthod Dentofacial Orthop. 2010 Jul;138(1):96-108. doi: 10.1016/j.ajodo.2010.03.014.

Vertical-dimension control during en-masse retraction with mini-implant anchorage.

Upadhyay M[1], Yadav S, Nanda R.

Author information

1 Division of Orthodontics, Department of Craniofacial Sciences, University of Connecticut Health Center, Farmington, CT 06030, USA. madhurup@yahoo.com

Abstract

Vertical dimension control with fixed appliance mechanotherapy has always been a matter of concern in moderate to severe high-angle patients with severe proclination of the anterior teeth. Extrusion of the posterior teeth is an inevitable sequela of conventional mechanics in such patients, especially if they are adults. This article reports on 3 patients who were treated with mini-implants (diameter, 1.3 mm; length, 8 mm) placed between the roots of the second premolar and the first molar for en-masse retraction of the anterior teeth. The biomechanical force system that developed not only maintained absolute anchorage, but also provided effective control over the posterior dentoalveolar dimension so that dramatic improvement in facial esthetics could be achieved. Small but significant autorotation of the mandible was noted; this led to increased chin prominence in all 3 patients. With accurate diagnosis and treatment planning, mini-implant anchorage can be a possible alternative to orthognathic surgery in similar borderline patients.

PMID: 20620840 DOI: 10.1016/j.ajodo.2010.03.014

The German Shortened Dental Arch Study: Quality of Life

The German Shortened Dental Arch Study: Quality of Life

Objectives: A multi-center randomized clinical trial (RCT) is under way at 14 university dental schools in Germany to compare two prosthetic treatment alternatives for the shortened dental arch. One aim of the RCT was to measure the effect of the tested treatment alternatives on the oral health-related quality of life (OHQoL).
Methods: Inclusion criteria of the RCT were: all molars of the test arch were missing but at least both canines and one premolar in each quadrant were present. Two hundred-fifteen participants were randomly assigned to receive either removable dental prostheses including molar replacement (RDP_group) or retain a shortened dental arch (SDA_group). The Oral Health Impact Profile (OHIP-49) were completed by participants before treatment (pre-treatment), six weeks (baseline), 6 month and then annually (12m, 24m, 36m) after treatment.

Results: From the 215 randomized participants 152 participants reach baseline. At the 36m follow-up, OHIP-data of 71 women and 63 men (mean age: 59 years) were available. Medians of the OHIP total-scores were as follows: RDP (n=72), 38.0 (pre-treatment), 27.0 (baseline), 13.0 (12m), 13.5 (24m), 13.0 (36m). SDA (n=61): 40.0 (pre-treatment), 19.0 (baseline), 15.5 (12m), 15.0 (24m), 16.0 (36m). Significant differences ($P \leqslant 0.0001$) were found in both groups between pre-treatment and all follow up sessions; between baseline and 12m/24m/36m only in group RDP ($P \leqslant 0.05$) but not in group SDA ($P>0.05$). However between the follow-ups 12m, 24m and 36m no significant differences were found in both groups ($P>0.05$). There were no significant differences between treatment groups at any time ($P>0.05$).

Conclusion: Within each group, an improvement of OHQoL was observed. This improvement increased within the first year for the RDP group. Thereafter no significant change was observed within the next two years for both groups. No significant differences in OHQoL were found between the two treatment concepts. (Supported by DFG: grant WA 831/2-1-6).

Division: IADR/AADR/CADR General Session
Meeting: 2009 IADR/AADR/CADR General Session (Miami, Florida)
Location: Miami, Florida
Year: 2009
Final Presentation ID: 1716

Authors

- Wolfart, Stefan (RWTH Aachen University, Aachen, N/A, Germany)
- Jahn, Florentine (Friedrich-Schiller-University, Jena, N/A, Germany)
- Gitt, Ingrid (University Leipzig, Leipzig, N/A, Germany)
- Hartmann, Sinsa (Johannes Gutenberg University, Mainz, N/A, Germany)
- Huppertz, J. (Bayrische Julius-Maximilians-Universität, Wuerzburg, N/A, Germany)
- Kern, Matthias (Department of Prosthodontics, Propaedeutics and Dental Materials, Christian-Albrechts University at Kiel, Kiel, N/A, Germany)
- Klein, Vera (Rheinische Friedrich-Wilhelms-University, Bonn, N/A, Germany)
- Marre, Birgit (Technical University of Dresden, Dresden, N/A, Germany)
- Mundt, Torsten (University of Greifswald, Greifswald, N/A, Germany)
- Pospiech, Peter (Saarland University, Homburg, N/A, Germany)
- Richter, Ernst-jürgen (Bayrische Julius-Maximilians-Universität, Wuerzburg, N/A, Germany)
- Heydecke, Guido (University Medical Center Eppendorf, Hamburg, N/A, Germany)
- Wostmann, Bernd (Justus-Leibig-University, Giessen, N/A, Germany)
- Walter, M. (Technical University of Dresden, Dresden, N/A, Germany)
- Luthardt, Ralph G. (Ulm University, Ulm, N/A, Germany)
- Gerss, Joachim (Westfalian Wilhelm University Muenster, Muenster, N/A, Germany)
- Busche, Ekkerhardt (Universität Witten / Herdecke, Witten, N/A, Germany)
- Boening, Klaus (Technical University of Dresden, Dresden, N/A, Germany)
- Eschbach, Stephanie (Department of Prosthodontics, Propaedeutics and Dental Materials, Christian-Albrechts University at Kiel, Kiel, N/A, Germany)
- Aggstaller, Hans (LMU Munich, Munich, N/A, Germany)
- Freesmeyer, Wolfgang B. (Charité-Universitätsmedizin Berlin, Campus Benjamin Franklin, Berlin, N/A, Germany)

SESSION INFORMATION
Oral Session
Outcomes of Prosthodontic Investigations and Biological Investigations
04/03/2009

Clin Oral Investig. 2014;18(2):525-33. doi: 10.1007/s00784-013-0991-6. Epub 2013 May 17.

The randomized shortened dental arch study: oral health-related quality of life.

Wolfart S[1], Müller F, Gerß J, Heyedcke G, Marré B, Böning K, Wöstmann B, Kern M, Mundt T, Hannak W, Brückner J, Passia N, Jahn F, Hartmann S, Stark H, Richter EJ, Gernet W, Luthardt RG, Walter MH.

Author information

1 Department of Prosthodontics and Biomaterials, Medical Faculty, RWTH Aachen University, Pauwelsstraße 30, 52074, Aachen, Germany, swolfart@ukaachen.de.

Abstract

OBJECTIVES: Although the shortened dental arch (SDA) concept is a widely accepted strategy to avoid overtreatment, little is known on its impact on oral health-related quality of life (OHRQoL). This multicenter randomized controlled trial aimed to investigate the OHRQoL for removable partial dental prostheses (RPDP) with molar replacement versus the SDA concept.

MATERIAL AND METHODS: In both groups, missing anterior teeth were replaced with fixed dental prosthesis. Two hundred fifteen patients with bilateral molar loss in at least one jaw were included. The Oral Health Impact Profile (OHIP-49) was completed before; 6 weeks (baseline), 6 months, and 12 months after treatment; and thereafter annually until 5 years.

RESULTS: Of the initial cohort, 81 patients were assigned to the RPDP group and 71 to the SDA group (age, 34 to 86 years). Before treatment, the median OHIP score was similar in both groups (RPDP, 38.0; SDA, 40.0; n.s.). Results indicate marked improvements in OHRQoL in both groups between pretreatment and baseline (RPDP, 27.0; SDA, 19.0; $p \leq 0.0001$) which continued in the RPDP group until the 1-year follow-up ($p = 0.0002$). These significant reductions in OHIP scores are reflected in its subscales. No further differences were seen within and between groups during the remainder observation period.

CONCLUSION: Both treatments show a significant improvement in OHRQoL which continued in the RPDP group until the 1-year follow-up. No significant differences were seen between groups.

CLINICAL RELEVANCE: For improving OHRQoL, it is not necessary to replace missing molars with a RPDP.

PMID: 23680969 DOI: 10.1007/s00784-013-0991-6

Three-year Clinical Evaluation of Resin Composites in Non-carious Cervical Lesions

Three-year Clinical Evaluation of Resin Composites in Non-carious Cervical Lesions

Objective: This randomized controlled clinical trial evaluated 3-year clinical performance of two types of resin composite restored with a one-step self-etch system. Methods: Ninety-eight non-carious cervical lesions in 22 patients (11 men and 11 women) with a mean age of 61.9 years (range 29-78) were involved for the study. Enamel bevel was placed and dentin walls were lightly ground, and restored with a hybrid (AP-X: AP, Kuraray Medical) or a flowable resin composite (Flow FX: FX, Kuraray) in conjunction with S3 Bond (Kuraray). Each patient received both restorative groups randomly. All restorations (48 restorations for AP and 50 restorations for FX) were placed by one dentist. The restorations were blindly evaluated every year using modified USPHS criteria by two examiners. The data were statistically analyzed using the Cochran Q test and Fisher's exact test. Results: All but five restorations (2: extraction due to severe periodontitis, 3: lost) were evaluated after 3 years. Retention rates of AP and FX were 100% and 94%. All retention failures occurred within 6 months. No recurrent caries was detected on any restorations. The only problem was marginal integrity at the enamel margins. Marginal staining increased with time, and its 3-year rates for AP and FX were 23% and 26%, respectively. However, marginal stains were still superficial and clinically acceptable. There was no significant difference in the clinical performance between AP and FX for each variable. Conclusions: Within the limits, the flowable composite in non-carious cervical lesions restored with S3 Bond demonstrated an acceptable clinical performance up to 3 years.

Division: IADR/AADR/CADR General Session
Meeting: 2009 IADR/AADR/CADR General Session (Miami, Florida)
Location: Miami, Florida
Year: 2009
Final Presentation ID: 3246

Authors

- Kubo, Shisei (Nagasaki University, Nagasaki, N/A, Japan)
- Yokota, Hiroaki (Clover Dental Clinic, Fukuoka, N/A, Japan)
- Yokota, Haruka (Clover Dental Clinic, Fukuoka, N/A, Japan)
- Hayashi, Yoshihiko (Nagasaki University, Nagasaki, N/A, Japan)

Kubo, Shisei (Nagasaki University, Nagasaki, N/A, Japan)
Yokota, Hiroaki (Clover Dental Clinic, Fukuoka, N/A, Japan)
Yokota, Haruka (Clover Dental Clinic, Fukuoka, N/A, Japan)
Hayashi, Yoshihiko (Nagasaki University, Nagasaki, N/A, Japan)

SESSION INFORMATION
Poster Session
Composite and Composite-combination Restorations
04/04/2009

J Dent. 2010 Mar;38(3):191-200. doi: 10.1016/j.jdent.2009.10.003. Epub 2009 Oct 17.

Three-year clinical evaluation of a flowable and a hybrid resin composite in non-carious cervical lesions.

Kubo S[1], Yokota H, Yokota H, Hayashi Y.

Author information

1 Center for Clinical Education and Training, Nagasaki University Hospital, 1-7-1 Sakamoto, Nagasaki 852-8588, Japan. kubo@nagasaki-u.ac.jp

Abstract

OBJECTIVES: This randomized controlled clinical trial evaluated the 3-year clinical performance of a hybrid (Clearfil AP-X; AP) and a flowable (Clearfil Flow FX; FX) resin composite in 98 non-carious cervical lesions.

METHODS: Twenty-two patients, 11 males and 11 females (mean age: 61.9 years, range: 29-78 years) regularly visiting the Nagasaki University Hospital, participated in the study. Each patient received both materials randomly. All restorations (48 restorations for AP and 50 restorations for FX) were placed in conjunction with an all-in-one system (Clearfil S(3) Bond) by one dentist. The restorations were blindly evaluated by two examiners at baseline, 6 months, 1, 2 and 3 years using modified USPHS criteria. The data were statistically analyzed using the Cochran's Q-test and Fisher's exact test.

RESULTS: All the patients were examined at each recall. However, five restorations could not be evaluated at 3-year recall as two teeth had been extracted and three restorations had been lost. The only minor problem was the integrity of the enamel margin. The incidence and extent of marginal staining increased with time, but it was still superficial. Marginal staining occurred adjacent to 11 restorations for AP and 12 restorations for FX after 3 years. Neither lesion size nor depth had influence on marginal staining adjacent to each type of resin composite. There were no significant differences in the clinical performances between AP and FX for each variable.

CONCLUSIONS: Under the protocol used in this study, both types of resin composite in conjunction with S(3) Bond demonstrated an acceptable clinical performance up to 3 years.

Copyright 2009 Elsevier Ltd. All rights reserved.

PMID: 19840829 DOI: 10.1016/j.jdent.2009.10.003
