# Supplementary material for: Reporting inconsistency between published conference abstracts and article abstracts of randomised controlled trials in prosthodontics presented at IADR general sessions
Source: PeerJ. 2023 May 5;11:e15303. doi: 10.7717/peerj.15303 (PMC10166077; doi:10.7717/peerj.15303)
Supplement: Supplemental Information 3 [file peerj-11-15303-s003.pdf]

2010

2-Year Clinical Effectiveness of All-in-One Adhesives in Non-carious Cervical Lesions

2-Year Clinical Effectiveness of All-in-One Adhesives in Non-carious Cervical Lesions

Objectives: To evaluate the clinical performance of two one-step self-etch adhesives in a randomized controlled clinical trial. Methods: Twenty-nine patients had 172 non-carious cervical lesions restored with composite (Gradia Direct Anterior; GC) using either the HEMA-rich adhesive Clearfil Tri-S Bond (Kuraray) or the HEMA-free adhesive G-Bond (GC). The restorations were evaluated by two calibrated examiners at baseline and after 6, 12 and 24 months of clinical service regarding retention, caries recurrence, marginal integrity and discoloration, preservation of tooth vitality and post-operative sensitivity. Retention loss, caries recurrence, or any severe marginal defects/discoloration requiring clinical intervention (repair or replacement) were considered as clinical failures. The data were statistically analysed using the Mann-Whitney U and Friedman ANOVA tests ($p<0.05$). Results: The recall rate at 6 and 12 months was 100%, and decreased to 93.1% at 24 months. At the last recall, the retention rate was 98.6% for Clearfil Tri-S Bond and 98.7% for G-Bond. No significant differences were observed between the two adhesives for all the parameters evaluated, irrespective of the recall ($p>0.05$). After 24 months of clinical service, both adhesives presented an increase in the percentage of small, though still clinically acceptable, marginal defects (13.7% for Clearfil Tri-S Bond, and 14.6% for G-Bond) and marginal discoloration (24.7% for Clearfil Tri-S Bond, and 18.7% for G-Bond). For both adhesives, tooth sensitivity was significantly decreased at 6 months as compared to the pre-operative condition. This relative reduction in sensitivity remained stable up to the 24-month recall. At 24 months, the overall clinical success rate was 95.9% and 96% for Clearfil Tri-S Bond and G-Bond, respectively. Conclusion: Both one-step self-etch adhesives presented an equally favorable clinical effectiveness at 24 months.

Division: IADR/PER General Session
Meeting: 2010 IADR/PER General Session (Barcelona, Spain)
Location: Barcelona, Spain
Year: 2010
Final Presentation ID: 694

Authors

- Moretto, Simone Gonçalves (Universidade de São Paulo, São Paulo, N/A, Brazil)
- Russo, Eliza Maria Agueda (Universidade de São Paulo, São Paulo, N/A, Brazil)
- Carvalho, Rubens Corte Real (Universidade de São Paulo, São Paulo, N/A, Brazil)
- Van Landuyt, Kirsten (Katholieke Universiteit Leuven, Leuven, N/A, Belgium)
- Peumans, Marleen (Katholieke Universiteit Leuven, Leuven, N/A, Belgium)
- Meerbeek, Bart Van (Katholieke Universiteit Leuven, Leuven, N/A, Belgium)
- Cardoso, Marcio Vivan (Katholieke Universiteit Leuven, Leuven, N/A, Belgium)

Moretto, Simone Gonçalves (Universidade de São Paulo, São Paulo, N/A, Brazil)
Russo, Eliza Maria Agueda (Universidade de São Paulo, São Paulo, N/A, Brazil)
Carvalho, Rubens Corte Real (Universidade de São Paulo, São Paulo, N/A, Brazil)
Van Landuyt, Kirsten (Katholieke Universiteit Leuven, Leuven, N/A, Belgium)
Peumans, Marleen (Katholieke Universiteit Leuven, Leuven, N/A, Belgium)
Meerbeek, Bart Van (Katholieke Universiteit Leuven, Leuven, N/A, Belgium)
Cardoso, Marcio Vivan (Katholieke Universiteit Leuven, Leuven, N/A, Belgium)

SESSION INFORMATION
Poster Session
Clinical Research: Bonding Agents and Mechanisms
07/15/2010

J Dent. 2013 Aug;41(8):675-82. doi: 10.1016/j.jdent.2013.05.016. Epub 2013 Jun 6.

3-year clinical effectiveness of one-step adhesives in non-carious cervical lesions.

Moretto SG[1], Russo EM, Carvalho RC, De Munck J, Van Landuyt K, Peumans M, Van Meerbeek B, Cardoso MV.

Author information

1 KU Leuven - BIOMAT, Department of Oral Health Sciences, Catholic University of Leuven, Belgium.

Abstract

OBJECTIVES: Despite representing an important component of current dental adhesives, HEMA has been said to negatively influence the long-term stability of adhesion to dentine and enamel. The aim of this randomised clinical trial was to evaluate the 3-year clinical performance of two one-step self-etch adhesives.

METHODS: Thirty patients had 175 non-carious cervical lesions restored with composite (Gradia Direct Anterior, GC) using either the HEMA-rich adhesive Clearfil Tri-S Bond (C3S; Kuraray) or the HEMA-free adhesive G-Bond (GB; GC). The restorations were evaluated by two examiners at baseline, 6, 12, 24 and 36 months regarding retention, caries recurrence, marginal integrity and discoloration and post-operative sensitivity. The data were statistically analysed with GEE and McNemar tests ($p<0.05$).

RESULTS: The recall rate at 6 and 12 months was 100% and decreased to 96.7% at 24 and 36 months. At 3 years, the retention rate was 93.8% for C3S and 98.8% for GB ($p=0.14$). A pairwise comparison showed no significant differences between the two adhesives for all the parameters evaluated, irrespective of the recall ($p>0.05$). After 3 years, both adhesives presented an increase in the percentage of clinically acceptable marginal discoloration (C3S: 32.9% and GB: 26.8%) normally associated to clinically acceptable marginal defects (C3S: 35.8% and GB: 26.5%). Only 1 dentine margin of a GB restoration presented a severe marginal defect (1.2%) and 1 C3S restoration presented caries recurrence. The overall 3-year clinical success rate was 92.6% for C3S and 97.6% for GB ($p=0.16$).

CONCLUSION: Both one-step self-etch adhesives presented an equally favourable clinical effectiveness at 3 years.

CLINICAL SIGNIFICANCE: HEMA is a monomer frequently present in dental adhesives in order to increase their wettability and hydrophilicity. However, this monomer negatively influences hydrolytic stability and durability of the adhesive interface complex. In this 3-year clinical trial no significant difference in bonding effectiveness was noticed between a HEMA-rich and HEMA-free one-step adhesive.

Copyright © 2013 Elsevier Ltd. All rights reserved.

KEYWORDS: Adhesive; Non-carious cervical lesion; Randomized clinical trial; Resin composite; Self-etch

PMID: 23747824 DOI: 10.1016/j.jdent.2013.05.016

6-Month Clinical Performance of a New Glass-Ionomer Restorative System

6-Month Clinical Performance of a New Glass-Ionomer Restorative System

Objective: to evaluate the 6-month clinical performance of a new packable glass-ionomer restorative system used for the restoration of Class II cavities. Methods: Twenty six patients with at least 2 but not more than 4 Class II lesions were enrolled in this study. A total of 60 lesions were randomly divided into two groups according to the restorative systems used (n=30). The lesions in Group 1, were restored with a new glass-ionomer restorative system (EQUIA/ GC) which is a combination of a packable glass-ionomer (Fuji IX GP EXTRA/ GC) and a self-adhesive nano-filled coating (G-Coat PLUS GC); whereas the lesions in Group 2 were restored with a micro-filled composite (Gradia Direct/ GC) in combination with a self-etch adhesive (G-Bond/ GC) according to the manufacturer's instructions. Two independent examiners evaluated the restorations at baseline, after 1 week and 6 months according to the modified USPHS criteria. The differences between two groups were statistically evaluated by Pearson Chi-Square test (p=0, 05). Results: At 6 month recall, 58 restorations were reviewed in 25 patients. Retention rates, anatomic form, recurrent caries, wear, surface texture, post-operative sensitivity and color match were scored as Alpha for all restorations in the two groups. For marginal adaptation, 4 restorations in Group 1 and 5 restorations in Group 2 were scored as Bravo. Besides, 1 restoration in Group 1 and 2 restorations in Group 2 were scored as Bravo for marginal discoloration. However, the differences in terms of marginal adaptation and marginal discoloration were not statistically significant (p>0, 05). Conclusions: At the end of 6 months, the clinical performance of the new glass-ionomer restorative system was as good as the micro-filled composite resin system.

Division: IADR/PER General Session
Meeting: 2010 IADR/PER General Session (Barcelona, Spain)
Location: Barcelona, Spain
Year: 2010
Final Presentation ID: 403

Authors

- Gurgan, Sevil (Hacettepe University, Ankara, N/A, Turkey)
- Cakir, Filiz Yalcin (Hacettepe University, Ankara, N/A, Turkey)
- Firat, Esra (Hacettepe University, Ankara, N/A, Turkey)
- Kutuk, Zeynep Bilge (Hacettepe University, Ankara, N/A, Turkey)
- Ak, Seval (Hacettepe University, Ankara, N/A, Turkey)

Gurgan, Sevil (Hacettepe University, Ankara, N/A, Turkey)
Cakir, Filiz Yalcin (Hacettepe University, Ankara, N/A, Turkey)
Firat, Esra (Hacettepe University, Ankara, N/A, Turkey)
Kutuk, Zeynep Bilge (Hacettepe University, Ankara, N/A, Turkey)
Ak, Seval (Hacettepe University, Ankara, N/A, Turkey)

SESSION INFORMATION
Oral Session
Clinical Research: Glass Ionomers, Endodontic Materials and Equipment
07/15/2010

6-Month Clinical Performance of a New Glass-Ionomer Restorative System

Clin Oral Investig. 2017 Sep;21(7):2335-2343. doi: 10.1007/s00784-016-2028-4. Epub 2016 Dec 20.

Clinical performance of a glass ionomer restorative system: a 6-year evaluation.

Gurgan S[1], Kutuk ZB[2], Ergin E[1], Oztas SS[1], Cakir FY[1].

Author information

Abstract

OBJECTIVES: The aim of this study is to evaluate the long-term clinical performance of a glass ionomer (GI) restorative system in the restoration of posterior teeth compared with a micro-filled hybrid posterior composite.

MATERIALS AND METHODS: A total of 140 (80 Cl1 and 60 Cl2) lesions in 59 patients were restored with a GI system (Equia) or a micro hybrid composite (Gradia Direct). Restorations were evaluated at baseline and yearly during 6 years according to the modified-USPHS criteria. Negative replicas at each recall were observed under SEM to evaluate surface characteristics. Data were analyzed with Cohcran's Q and McNemar's tests ($p < 0.05$).

RESULTS: One hundred fifteen (70 Cl1 and 45 Cl2) restorations were evaluated in 47 patients with a recall rate of 79.6% at 6 years. Significant differences were found in marginal adaptation and marginal discoloration for both restorative materials for Cl1 and Cl2 restorations ($p < 0.05$). However, none of the materials were superior to the other ($p > 0.05$). A significant decrease in color match was observed in Equia restorations ($p < 0.05$). Only one Cl2 Equia restoration was missing at 3 years and another one at 4 years. No failures were observed at 5 and 6 years. Both materials exhibited clinically successful performance after 6 years. SEM evaluations were in accordance with the clinical findings.

CONCLUSIONS: Both materials showed a good clinical performance for the restoration of posterior teeth during the 6-year evaluation.

CLINICAL RELEVANCE: The clinical effectiveness of Equia and Gradia Direct Posterior was acceptable in Cl1 and Cl2 cavities subsequent to 6-year evaluation.

KEYWORDS: Clinical performance; Composite resin; Glass ionomer cement; Posterior teeth

PMID: 28000039 DOI: 10.1007/s00784-016-2028-4

Comparison of Photodynamic Therapy and Antifungal Treatment for Denture Stomatitis

Objectives: This randomized clinical trial compared the clinical and mycological efficacy of Photodynamic Therapy (PDT) with that of topical antifungal therapy (nystatin oral suspension) for the treatment of denture stomatitis (DS). *Methods:* Forty DS patients were randomly assigned to one of two groups of 20 subjects each; NYS group: patients received topical treatment with nystatin (100,000-IU) four times daily for 15 days; PDT group: denture and palate of patients were sprayed with 500-mg/L of Photogem® and, after 30-min of incubation, illuminated by LED light at 455 nm (light fluences of 37.5- and 122-J/cm^2, respectively) three times a week for 15 days. Mycological cultures from oral swabs samples taken from dentures and palates and standard photographs of the palates were performed at baseline (day 0), at the end of the treatment (day 15) and at the follow-up (days 30, 60 and 90). Colonies on Sabouraud Dextrose Agar (37°C, 48-h) were quantified and the ln(CFU/mL) values were statistically analyzed by ANOVA and Tukey tests (α=0.05). The severity of inflammation of the palate in the photographs was evaluated according to Newton's classification. *Results:* Both treatments reduced significantly the CFU/mL values at the end of the treatments (day 15) and on day 30 of the follow-up period ($P<0.05$). NYS and PDT groups showed a rate of clinical success (cure or improvement of the palatal inflammation) of 53 and 45%, respectively, on day 15. At the follow-up period, recurrence of the palatal inflammation was observed in 75 and 78% of the patients who had clinical success respectively in the NYS and PDT groups. *Conclusion:* PDT appears to be an alternative method for the treatment of DS. Grant: FAPESP 2005/02193-4 and 2005/03226-3.

Division: IADR/PER General Session
Meeting: 2010 IADR/PER General Session (Barcelona, Spain)
Location: Barcelona, Spain
Year: 2010
Final Presentation ID: 1787

Authors

- Mima, Ewerton Garcia Oliveira (São Francisco University, Campinas, N/A, Brazil)
- Pavarina, Ana Cláudia (Universidade Est. Paulista Julio Mesquita, Araraquara, N/A, Brazil)
- Silva, Mariana Montenegro (Araraquara Dental School, Sao Paulo State University, Araraquara, N/A, Brazil)
- Ribeiro, Daniela Garcia (Araraquara Dental School, UNESP-Univ Estadual Paulista, Araraquara, N/A, Brazil)
- Dovigo, Lívia Nordi (Araraquara Dental School, UNESP-Univ Estadual Paulista, Araraquara, N/A, Brazil)
- Vergani, Carlos Eduardo (Araraquara Denatl School, UNESP - Univ Estadual Paulista, Department of Dental Materials and Prosthodontics, Araraquara, N/A, Brazil)
- Bagnato, Vanderlei Salvador (Universidade de São Paulo, São Carlos, N/A, Brazil)

Mima, Ewerton Garcia Oliveira (São Francisco University, Campinas, N/A, Brazil)
Pavarina, Ana Cláudia (Universidade Est. Paulista Julio Mesquita, Araraquara, N/A, Brazil)
Silva, Mariana Montenegro (Araraquara Dental School, Sao Paulo State University, Araraquara, N/A, Brazil)
Ribeiro, Daniela Garcia (Araraquara Dental School, UNESP-Univ Estadual Paulista, Araraquara, N/A, Brazil)
Dovigo, Lívia Nordi (Araraquara Dental School, UNESP-Univ Estadual Paulista, Araraquara, N/A, Brazil)
Vergani, Carlos Eduardo (Araraquara Denatl School, UNESP - Univ Estadual Paulista, Department of Dental Materials and Prosthodontics, Araraquara, N/A, Brazil)
Bagnato, Vanderlei Salvador (Universidade de São Paulo, São Carlos, N/A, Brazil)

SESSION INFORMATION
Poster Session
Yeasts and Fungi
07/15/2010

Comparison of Photodynamic Therapy and Antifungal Treatment for Denture Stomatitis

Comparison of Photodynamic Therapy versus conventional antifungal therapy for the treatment of denture stomatitis: a randomized clinical trial

来自Wiley

收藏 引用 批量引用 报错 分享

作者 EG Mima , CE Vergani , AL Machado , EMS Massucato , AL Colombo , ...

摘要 In this randomized clinical trial, the clinical and mycological efficacy of Photodynamic Therapy (PDT) was compared with that of topical antifungal therapy for the treatment of denture stomatitis (DS) and the prevalence of Candida species was identified. Patients were randomly assigned to one of two groups (n = 20 each); in the nystatin (NYT) group patients received topical treatment with nystatin (100 000 IU) four times daily for 15 days and in the PDT group the denture and palate of patients were sprayed with 500 mg/L of Photogem0003, and after 30 min of incubation, were illuminated by light emitting-diode light at 455 nm (37.5 and 122 J/cm2, respectively) three times a week for 15 days. Mycological cultures taken from dentures and palates and standard photographs of the palates were taken at baseline (day 0), at the end of the treatment (day 15) and at the follow-up time intervals (days 30, 60 and 90). Colonies were quantified (CFU/mL) and identified by biochemical tests. Data were analysed by Fisher090005s exact test, analysis of variance and Tukey tests and 0202 test (= 0.05). Both treatments significantly reduced the CFU/mL at the end of the treatments and on day 30 of the follow-up period (p <0.05). The NYT and PDT groups showed clinical success rates of 53% and 45%, respectively. Candida albicans was the most prevalent species identified. PDT was as effective as topical nystatin in the treatment of DS. ▲ 收起

出版源 《Clinical Microbiology & Infection》 , 2012 , 18 (10) :E380-E388

被引量 37

Contingent electrical stimulation effect on jaw muscle activity during sleep

Contingent electrical stimulation effect on jaw muscle activity during sleep

Objective: To determine the effect of contingent electrical stimulation (CES) on jaw muscle activity during sleep in a double-blinded randomized controlled trial (RCT). Methods: Eleven patients with myofascial TMD (mean age 37 years) and with a clinical diagnosis of bruxism were included. EMG activity (Grindcare®) was recorded from the anterior temporalis muscle, online analyzed and the frequency content determined using a Signal Recognition Algorithm (SRA). Jaw muscle activity related to clenching or grinding triggered an electrical square-wave pulse train (450 ms) adjusted to a clear, but non-painful intensity. TMD patients were randomized in two groups: active treatment with CES or no CES (placebo). All patients had baseline EMG recordings for 5-7 consecutive nights; the CES group followed by 6-weeks EMG recordings with the CES turned on; the placebo group followed by 6-weeks without CES, and finally 4 weeks EMG recording without CES for both groups. Numerical Rating Scale (NRS) for pain and hours of sleep were registered for at least 4 night per week during the whole experiment. In order to blind the patients, the device was set to trigger electrical pulses for the first 20 min in the placebo group. ANOVA was used to test the data. Results: The number of EMG episodes/hour sleep was significantly reduced (52 ± 12%) in CES group during the sessions with CES ($P<0.027$) compared to baseline. The placebo group showed no significant differences in the number of EMG episodes/hour sleep during the placebo sessions compared to baseline ($P>0.701$). There were no significant differences in NRS pain scores between groups ($P=0.958$). The average duration of sleep hours during the nights with and without CES was not significantly different ($P=0.456$). Conclusions: These preliminary results showed that there is a significant inhibitory effect of CES on jaw muscle EMG activity during sleep in a RCT design.

Division: IADR/PER General Session
Meeting: 2010 IADR/PER General Session (Barcelona, Spain)
Location: Barcelona, Spain
Year: 2010
Final Presentation ID: 2782

Authors

- Jadidi, Faramarz (Department of Clinical Oral Physiology, Aarhus University, Aarhus, N/A, Denmark)
- Castrillon, Eduardo (Department of Clinical Oral Physiology, Aarhus University, Aarhus, N/A, Denmark)
- Baad-hansen, Lene (Department of Clinical Oral Physiology, Aarhus University, Aarhus, N/A, Denmark)
- Svensson, Peter (Department of Clinical Oral Physiology, Aarhus University, Aarhus, N/A, Denmark)

Jadidi, Faramarz (Department of Clinical Oral Physiology, Aarhus University, Aarhus, N/A, Denmark)
Castrillon, Eduardo (Department of Clinical Oral Physiology, Aarhus University, Aarhus, N/A, Denmark)
Baad-hansen, Lene (Department of Clinical Oral Physiology, Aarhus University, Aarhus, N/A, Denmark)
Svensson, Peter (Department of Clinical Oral Physiology, Aarhus University, Aarhus, N/A, Denmark)

SESSION INFORMATION
Poster Session
Bruxism and Parafunctional Activities
07/16/2010

Contingent electrical stimulation effect on jaw muscle activity during sleep

Acta Odontol Scand. 2013 Sep;71(5):1050-62. doi: 10.3109/00016357.2012.741702. Epub 2012 Nov 13.

Effect of contingent electrical stimulation on jaw muscle activity during sleep: a pilot study with a randomized controlled trial design.

Jadidi F[1], Castrillon EE, Nielsen P, Baad-Hansen L, Svensson P.

Author information

1 Section of Clinical Oral Physiology, Department of Dentistry, HEALTH, Aarhus University, Denmark.

Abstract

OBJECTIVE: To determine the effect of contingent electrical stimulation (CES) on jaw muscle activity during sleep in a double-blinded randomized controlled trial (RCT).

MATERIALS AND METHODS: Eleven patients with myofascial TMD (mean age 37 years) and with a clinical diagnosis of bruxism were included. EMG activity (Grindcare®) was recorded from the anterior temporalis muscle during sleep and analyzed online. Jaw muscle activity related to clenching or grinding triggered an electrical square-wave pulse train (450 ms) adjusted to a clear, but non-painful intensity. TMD patients were randomized into two groups: active treatment with CES or no CES (placebo). Number of EMG episodes/hour sleep was the primary outcome parameter. The following variables were assessed as secondary outcome parameters; number of painful muscles, maximum pain-free jaw opening, characteristic pain intensity, depression scores and Oral Health Impact Profile scores. Numerical Rating Scale scores for self-reported pain and muscle tension were registered for at least 4 nights per week during the experiment.

RESULTS: The number of EMG episodes/hour sleep was significantly reduced (52 ± 12%) in the CES group during the sessions with CES (ANOVA: $p = 0.021$) compared to baseline. There were no significant differences in the secondary outcome parameters (ANOVA: $p > 0.513$) or pain or muscle tension scores between groups ($p = 0.645$). The average duration of sleep hours during the nights with and without CES was not significantly different ($p = 0.646$).

CONCLUSIONS: These results demonstrate a significant inhibitory effect of CES on jaw muscle EMG activity during sleep in a RCT, but with no effects on self-reported pain.

PMID: 23148551 DOI: 10.3109/00016357.2012.741702

Effect of Electromyogram-Biofeedback on Daytime Clenching Behavior in TMD Subjects

Objectives: Although daytime clenching is believed to be one of the oral parafunctions leading to dental problems, a treatment strategy has not yet been devised. Electromyogram (EMG) biofeedback training was performed to ascertain its effect on the regulation of daytime clenching behavior.
Methods: Twenty subjects (mean age, 30.9 ± 5.6 years) who had mild to moderate masticatory muscle pain with daytime clenching behavior were randomly divided into either a biofeedback group (BF) or control group (CO). Subjects were fitted with a hearing-aid-shaped EMG recording and biofeedback apparatus which was used to record EMG data under natural conditions from the temporal muscle, continuously for five hours on four consecutive days. EMG data on Days 1 and 4 were recorded without biofeedback as pre-test and post-test, respectively, and on Days 2 and 3, subjects in the BF group noticed their clenching behaviors via an alert sound from the EMG biofeedback apparatus. No alert sound was given for the CO group throughout the recording sessions.

Results: There was no significant difference in the number of clenching events for five hours between the BF group (4.6 ± 2.5) and CO group (4.6 ± 0.9) on Day 1, however a significant decrease was found in the BF group between Day 1 (4.6 ± 2.5) and Day 4 (2.4 ± 1.7; $P < 0.05$, Bonferroni multiple comparison tests).

Conclusion: Daytime clenching was reduced in the short-term with the help of an EMG biofeedback system under natural circumstances.

Division: IADR/PER General Session
Meeting: 2010 IADR/PER General Session (Barcelona, Spain)
Location: Barcelona, Spain
Year: 2010
Final Presentation ID: 2783

Authors

- Fujisawa Masanori (Meikai University, Sakado, N/A, Japan)
- Watanabe Akira (Iwate Medical University, Morioka, N/A, Japan)
- Kanemura Kiyotaka (Iwate Medical University, Morioka, N/A, Japan)
- Tanabe Norimasa (Iwate Medical University, Morioka, N/A, Japan)
- Iizuka Tomoaki (Meikai University, Sakado, N/A, Japan)
- Sato Masayuki (Meikai University, Sakado, N/A, Japan)
- Iwase Naoki (Meikai University, Sakado, N/A, Japan)
- Ishibashi Kanji (Iwate Medical University, Morioka, N/A, Japan)

SESSION INFORMATION
Poster Session
Bruxism and Parafunctional Activities
07/16/2010

Effect of Electromyogram-Biofeedback on Daytime Clenching Behavior in TMD Subjects

☐ Effect of electromyogram biofeedback on daytime clenching behavior in subjects with masticatory muscle pain.
(PMID:21130060)

Abstract | Citations | Related Articles | Data | BioEntities | External Links

Watanabe A, Kanemura K, Tanabe N, Fujisawa M

Journal of Prosthodontic Research [03 Dec 2010, 55(2):75-81]

Type: Research Support, Non-U.S. Gov't, Randomized Controlled Trial, Journal Article
DOI: 10.1016/j.jpor.2010.09.003

Abstract

PURPOSE: Although daytime clenching is believed to be one of the oral parafunctions leading to dental problems, a treatment strategy has not yet been devised. Electromyogram (EMG) biofeedback training was performed to ascertain its effect on the regulation of daytime clenching behavior. MATERIALS AND METHODS: Twenty subjects (mean age, 30.9±5.6 years) who had mild to moderate masticatory muscle pain with daytime clenching behavior were randomly divided into either a biofeedback group (BF) or control group (CO). Subjects were fitted with a hearing-aid-shaped EMG recording and biofeedback apparatus which was used to record EMG data under natural conditions from the temporal muscle, continuously for five hours on four consecutive days. EMG data on Days 1 and 4 were recorded without biofeedback as pre-test and post-test, respectively, and on Days 2 and 3, subjects in the BF group noticed their clenching behaviors via an alert sound from the EMG biofeedback apparatus. No alert sound was given for the CO group throughout the recording sessions. RESULTS: There was no significant difference in the number of clenching events for five hours between the BF group (4.6±2.5) and CO group (4.6±0.9) on Day 1, however a significant decrease was found in the BF group between Day 1 (4.6±2.5) and Day 4 (2.4±1.7; $P<0.05$). CONCLUSION: Daytime clenching was reduced in the short-term with the help of an EMG biofeedback system under natural circumstances. Further research is needed to confirm a long-lasting effect.

Effectiveness of Prefabricated Occlusal Appliance in TMD Patients with Headache

HOME MEETINGS ADVANCED SEARCH INSTRUCTIONS

Effectiveness of Prefabricated Occlusal Appliance in TMD Patients with Headache

Objectives: To compare the short- and long-term effectiveness of a prefabricated occlusal appliance with a stabilization appliance for the treatment of headache in myofascial pain patients. **Methods:** Sixty-five myofascial pain patients of whom 57 reported headache were included in a randomized controlled trial at two centres for Stomatognathic Physiology in Sweden and Finland. Patients were randomly assigned to a prefabricated appliance (R-group, 27 women, 5 men, mean age 38 years) or a stabilization appliance group (S-group, 31 women 2 men, mean age 37 years). RDC/TMD was used for history-taking and clinical examination. Frequency and intensity of headache were assessed using a verbal scale (1=continuous, 2=recurrent, 3=rarely) and a numeric rating scale (NRS) respectively at baseline, 10-week, 6-and 12-month follow-ups. **Results:** At baseline there were no differences between the groups regarding frequency and intensity of headache. At the follow-ups a statistically significant decrease in both frequency and intensity was observed within the two groups without any differences between the groups. At baseline 72% and 70% in the R and S groups reported recurrent or continuous headache which decreased significantly (p< .01) within both groups to 44% and 31% at 10-week and to 26% and 18% at the 12-month follow-up. Mean intensity (NRS) of headache at baseline decreased within both groups from 6 to 3 at 10-week follow-up and to 2 and 3 in the R and S groups respectively (p< .001) at 12-month follow-up. **Conclusions:** In both short- and long-term the prefabricated appliance seemed to have a similar effectiveness in the treatment of headache in myofascial pain patients.

Division: IADR/PER General Session
Meeting: 2010 IADR/PER General Session (Barcelona, Spain)
Location: Barcelona, Spain
Year: 2010
Final Presentation ID: 3605

Authors

- Doepel Marika (University of Turku, Turku, N/A, Finland)
- Le Bell Yrsa (University of Turku, Turku, N/A, Finland)
- Ekberg Ewacarin (Malmö University, Malmö, N/A, Sweden)
- Nilner Maria (Malmö University, Malmö, N/A, Sweden)

SESSION INFORMATION
Oral Session
Orofacial Pain and TMD: Treatment
07/17/2010

Effectiveness of Prefabricated Occlusal Appliance in TMD Patients with Headache

J Oral Facial Pain Headache. 2014 Spring;28(2):128-37. doi: 10.11607/ofph.1216.

Effectiveness of a prefabricated occlusal appliance in patients with temporomandibular joint pain: a randomized controlled multicenter study.

Christidis N, Doepel M, Ekberg E, Ernberg M, Le Bell Y, Nilner M.

Abstract

AIMS: To evaluate the effectiveness of a prefabricated appliance and compare it to the effectiveness of a stabilization appliance in patients with temporomandibular joint (TMJ) pain.

METHODS: This randomized, controlled multicenter study comprised 48 patients diagnosed with TMJ arthralgia according to the Research Diagnostic Criteria for Temporomandibular Disorders. The effectiveness of a prefabricated appliance (Relax), worn by half of the patients (referred to as the R group), was compared to the effectiveness of a stabilization appliance, worn by the other half of patients (S group). Treatment outcome was assessed according to the recommendations by the Initiative on Methods, Measurement, and Pain Assessment in Clinical Trials (IMMPACT) on an intent-to-treat basis. To analyze the differences between groups, the chi-square test and the Mann-Whitney U test were used, while the Friedman analysis of variance (ANOVA) on ranks was used for the analyses between baseline data and follow-up measurements, all with a significance level set at $P < .05$.

RESULTS: There were no differences between the groups at baseline. A 30% reduction of pain intensity was reported by 62.5% of the R group and 58.3% of the S group at the 10-week follow-up; 58% and 50.3%, respectively, at the 6-month follow-up; and 41.7% in both groups at 12 months. At the 12-month follow-up, pain intensity had decreased and physical function had improved in both groups ($P < .005$ and $P < .016$, respectively), without significant group differences. Emotional function (depression and nonspecific physical symptoms) did not change. Overall improvement of "better" to "symptom-free" was observed in 67% of the R group and 58% of the S group. No side effects occurred.

CONCLUSION: The effectiveness of the prefabricated appliance seems to be similar to that of the stabilization appliance in alleviating TMJ pain. Since the prefabricated appliance requires only one visit for construction, it is convenient for both the general practitioner and for the patient.

PMID: 24822236

Efficacy of mouthwashes on Candida albicans with denture stomatitis patients

Efficacy of mouthwashes on Candida albicans with denture stomatitis patients

Objective: Effective cleaning of dentures is important for the maintenance of good oral hygiene for denture stomatitis patients. Material and methods: The effectiveness of three different brands of alkaline peroxide tablets (Polident, Efferdent, and Fittydent) and two mouthwashes (CloSYS II and Corsodyl) to eliminate Candida albicans on dentures was evaluated in this "in vivo" study. Ninety denture wearers with clinical evidence of denture stomatitis were randomly divided into 6 groups: Five test groups and one control group. For 15, 30, and 60 minute disinfection procedure, each group was further divided into three subgroups. Each test group's prostheses were treated with one of the cleaners and control group's prostheses were treated with distilled water. Swab samples from the palatal surfaces (2cm x 2cm template defining area) of the upper dentures were obtained before and after 15, 30, and 60 minute periods of cleaners use and examined mycologically. Results: The results showed that the reduction in the number of colony-forming units (CFUs) of C. albicans before, and after 15, 30, and 60 minutes' use of CloSYS II and Corsodyl was significantly greater than that of the control group ($p<0.05$). Moreover, there was no difference between Polident, Efferdent, and the control in any of the treatment periods ($p > 0.05$). Denture treated with Fittydent appeared to have a significantly greater reduction in the number of Candida spp only after 60 minutes of treatment . Conclusions: The results of this study showed that the use of mouthwashes significantly reduced the number of micro organisms on dentures.

Division: IADR/PER General Session
Meeting: 2010 IADR/PER General Session (Barcelona, Spain)
Location: Barcelona, Spain
Year: 2010
Final Presentation ID: 2560

Authors

- Uludamar, Altay (Dental Clinic, Ankara, N/A, Turkey)
- Iseri, Ufuk (Yeditepe University, Istanbul, N/A, Turkey)
- Ozkan, Yasemin (Marmara University, Istanbul, N/A, Turkey)

Uludamar, Altay (Dental Clinic, Ankara, N/A, Turkey)
Iseri, Ufuk (Yeditepe University, Istanbul, N/A, Turkey)
Ozkan, Yasemin (Marmara University, Istanbul, N/A, Turkey)

SESSION INFORMATION
Poster Session
Chemical Plaque Control
07/16/2010

Efficacy of mouthwashes on Candida albicans with denture stomatitis patients

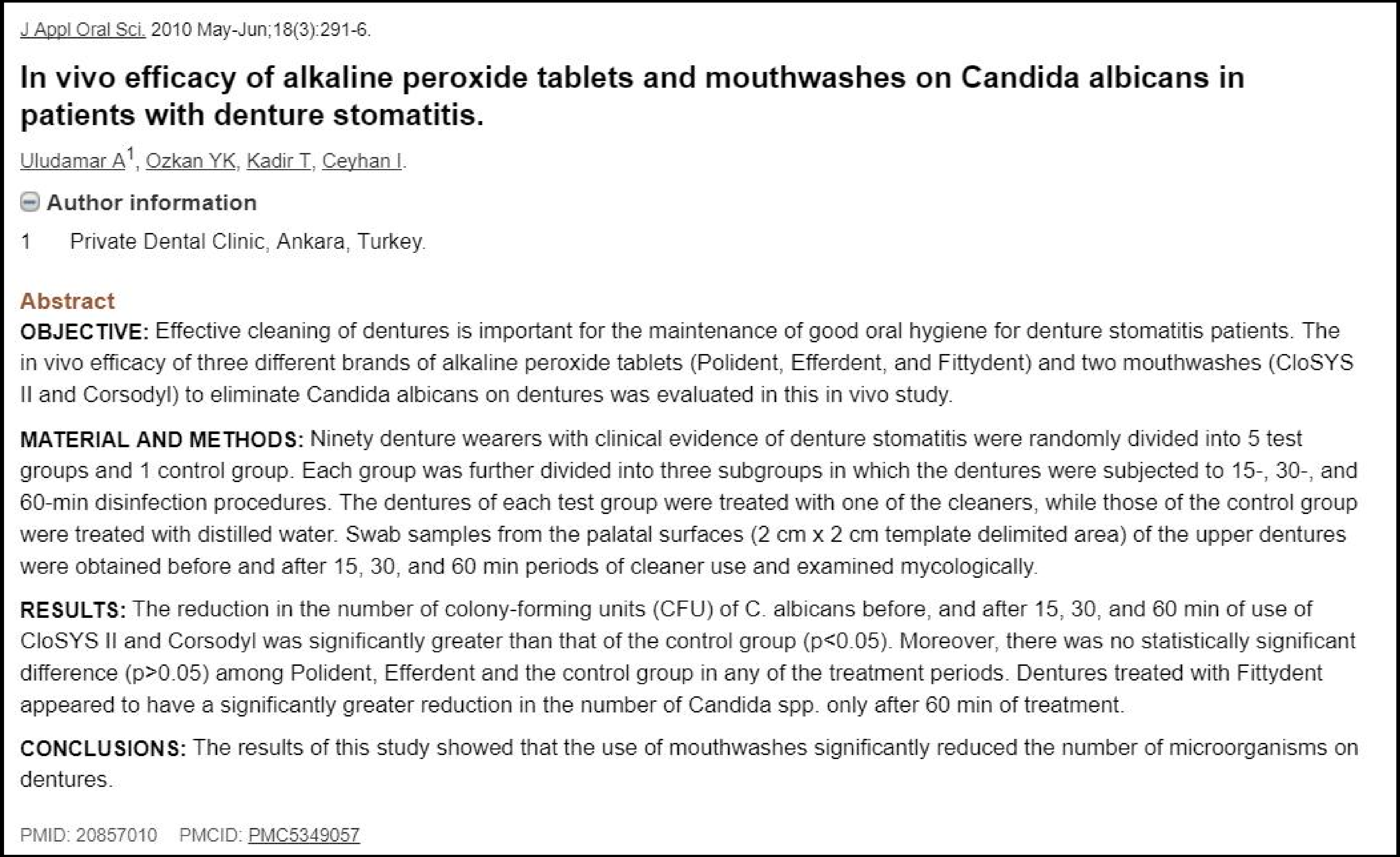
J Appl Oral Sci. 2010 May-Jun;18(3):291-6.

In vivo efficacy of alkaline peroxide tablets and mouthwashes on Candida albicans in patients with denture stomatitis.

Uludamar A[1], Ozkan YK, Kadir T, Ceyhan I.

Author information

1 Private Dental Clinic, Ankara, Turkey.

Abstract

OBJECTIVE: Effective cleaning of dentures is important for the maintenance of good oral hygiene for denture stomatitis patients. The in vivo efficacy of three different brands of alkaline peroxide tablets (Polident, Efferdent, and Fittydent) and two mouthwashes (CloSYS II and Corsodyl) to eliminate Candida albicans on dentures was evaluated in this in vivo study.

MATERIAL AND METHODS: Ninety denture wearers with clinical evidence of denture stomatitis were randomly divided into 5 test groups and 1 control group. Each group was further divided into three subgroups in which the dentures were subjected to 15-, 30-, and 60-min disinfection procedures. The dentures of each test group were treated with one of the cleaners, while those of the control group were treated with distilled water. Swab samples from the palatal surfaces (2 cm x 2 cm template delimited area) of the upper dentures were obtained before and after 15, 30, and 60 min periods of cleaner use and examined mycologically.

RESULTS: The reduction in the number of colony-forming units (CFU) of C. albicans before, and after 15, 30, and 60 min of use of CloSYS II and Corsodyl was significantly greater than that of the control group ($p<0.05$). Moreover, there was no statistically significant difference ($p>0.05$) among Polident, Efferdent and the control group in any of the treatment periods. Dentures treated with Fittydent appeared to have a significantly greater reduction in the number of Candida spp. only after 60 min of treatment.

CONCLUSIONS: The results of this study showed that the use of mouthwashes significantly reduced the number of microorganisms on dentures.

PMID: 20857010 PMCID: PMC5349057

HOME MEETINGS ADVANCED SEARCH INSTRUCTIONS

Efficiency of Occlusal Splint Treatment in Temporomandibular Disorders

Objectives: Temporomandibular disorders (TMD) embrace clinical problems involving the masticatory muscles and/or the temporomandibular joints. Stabilization splints (SS) have been generally used in the treatment of TMD. However, the evidence of their efficiency is scarce, and a need for qualified, well controlled studies exists. The aim of the study was to evaluate the efficiency of the SS treatment on facial pain in a randomized, controlled trial. Methods: The sample consisted of 80 consecutive patients referred to the Oral and Maxillofacial Department, Oulu University Hospital, for treatment of TMD. The patients were randomly allocated to the SS group (n=40) and the control group (n=40). Subjects in both groups received counceling and instructions for masticatory muscle exercises. In addition, subjects in the first group were treated with an SS. Visual analogue scale (VAS) on facial pain was assessed before treatment and one month after treatment. The mean of the changes in VAS scores at the two points of time were compared between the groups using the independent samples t-test. Results: Before treatment, the mean of the VAS was 5.2 (SD 2.8) in the SS group and 4.6 (SD 2.6) in the control group (p=0.323). One month after treatment the mean of the VAS change (indicating pain reduce) was 1.8 (SD 3.3) in the SS group and 0.8 (SD 2.5) in the control group (p=0.122). Conclusions: The efficacy of the SS treatment is not superior to mere muscle exercises in reducing facial pain in a short-time follow-up. The cost-benefit ratio should be considered in the treatment of TMD. Acknowledgements: Study supported by the Finnish Dental Society Apollonia and the Academy of Finland.

Division: IADR/PER General Session
Meeting: 2010 IADR/PER General Session (Barcelona, Spain)
Location: Barcelona, Spain
Year: 2010
Final Presentation ID: 3610

Authors

- Korpela Miia (University of Oulu, Oulu, N/A, Finland)
- Niemelä Katja (University of Oulu, Oulu, N/A, Finland)
- Sipilä Kirsi (University of Oulu, Oulu, N/A, Finland)

SESSION INFORMATION
Oral Session
Orofacial Pain and TMD: Treatment
07/17/2010

Back

Print Friendly

Efficiency of Occlusal Splint Treatment in Temporomandibular Disorders

Journal of Oral Rehabilitation

Efficacy of stabilisation splint treatment on temporomandibular disorders

K. NIEMELÄ, M. KORPELA, A. RAUSTIA, P. YLÖSTALO, K. SIPILÄ

First published: 19 July 2012 | https://doi.org/10.1111/j.1365-2842.2012.02335.x | Cited by: 24

K. Sipilä, Institute of Dentistry, University of Eastern Finland, Kuopio Campus, Box 1627, FIN-70211 Kuopio, Finland.
E-mail: kirsi.sipila@uef.fi

Abstract

Summary The evidence supporting the use of stabilisation splints in the treatment of temporomandibular disorders (TMD) is scarce and a need for well-controlled studies exists. The aim of this randomised, controlled trial study was to assess the efficacy of stabilisation splint treatment on TMD. The sample consisted of 80 consecutive referred patients who were randomly assigned to the splint group ($n = 39$) and the control group ($n = 41$). Subjects in the splint group were treated with a stabilisation splint, whereas subjects in the control group did not receive any treatment except counselling and instructions for masticatory muscle exercises which were given also to the subjects in the splint group. Outcomes were visual analogue scale (VAS) on facial pain intensity and clinical findings for TMD which were measured at baseline and after 1-month follow-up. The differences in change between the groups were analysed using regression models. Facial pain decreased and most of the clinical TMD findings resolved in both of the groups. The differences in changes in VAS or clinical TMD findings between the groups were not statistically significant. The findings of this study did not show that stabilisation splint treatment in combination with counselling and masticatory muscle exercises has additional benefit in relieving facial pain and increasing the mobility of the mandible than counselling and masticatory muscle exercises alone in a short time-interval.

Eight-Year Clinical Evaluation of a Two-Step Self-Etch Adhesive

Eight-Year Clinical Evaluation of a Two-Step Self-Etch Adhesive

Objectives: The objective of this randomized controlled clinical trial was to evaluate the 8-year clinical performance of a mild two-step self-etch adhesive in non-carious Class-V lesions with and without prior selective phosphoric acid-etching of the enamel cavity margins. Methods: A total of 100 non-carious Class-V lesions in 29 patients were restored with Clearfil AP-X (Kuraray). The composite restorations were bonded following two different approaches: 1. application of Clearfil SE (Kuraray) following a self-etch approach (control group; C-SE non-etch); 2. selective phosphoric acid-etching of the enamel cavity margins before application of Clearfil SE (experimental group; C-SE etch). The restorations were evaluated after 6 months, 1, 2, 3, 5 and 8 years of clinical service regarding retention, marginal integrity and discoloration, caries occurrence, preservation of tooth vitality and post-operative sensitivity. Results: The recall rate at 8 years was 76%. Only two restorations, one of the C-SE non-etch group and one of the C-SE etch group, were clinically unacceptable due to loss of retention leading to a retention rate and a clinical success rate of 97% in both groups. Aging of the restorations was characterized by an increase in the percentage of restorations with a clinically acceptable but small marginal defect (C-SE non-etch:92%; C-SE etch:84%) and/or superficial marginal discoloration (C-SE non-etch:44%; C-SE etch:28%). At the enamel side, the presence of small marginal defects (C-SE non-etch:86%; C-SE etch:65%) and superficial marginal discoloration (C-SE non-etch:36%; C-SE etch:11%) was more frequently noticed in the control group than in the experimental group. The difference, however, was only statistically significant for the presence of superficial marginal discoloration (McNemar, p=0.01). Conclusion: After 8 years of clinical functioning, the clinical effectiveness of Clearfil SE remained excellent, with selective acid-etching of the enamel cavity margins only having some minor positive effect on marginal integrity and absence of marginal discoloration at enamel.

Division: IADR/PER General Session
Meeting: 2010 IADR/PER General Session (Barcelona, Spain)
Location: Barcelona, Spain
Year: 2010
Final Presentation ID: 3565

Authors

- Peumans, Marleen (Katholieke Universiteit Leuven, Leuven, N/A, Belgium)
- De Munck, Jan (Katholieke Universiteit Leuven, Leuven, N/A, Belgium)
- Van Landuyt, Kirsten (Katholieke Universiteit Leuven, Leuven, N/A, Belgium)
- Poitevin, André (Katholieke Universiteit Leuven, Leuven, N/A, Belgium)
- Van Meerbeek, Bart (Katholieke Universiteit Leuven, Leuven, N/A, Belgium)

Peumans, Marleen (Katholieke Universiteit Leuven, Leuven, N/A, Belgium)
De Munck, Jan (Katholieke Universiteit Leuven, Leuven, N/A, Belgium)
Van Landuyt, Kirsten (Katholieke Universiteit Leuven, Leuven, N/A, Belgium)
Poitevin, André (Katholieke Universiteit Leuven, Leuven, N/A, Belgium)
Van Meerbeek, Bart (Katholieke Universiteit Leuven, Leuven, N/A, Belgium)

SESSION INFORMATION

Oral Session
Clinical Research: Resin Composites
07/17/2010

Eight-Year Clinical Evaluation of a Two-Step Self-Etch Adhesive

Dent Mater. 2010 Dec;26(12):1176-84. doi: 10.1016/j.dental.2010.08.190. Epub 2010 Oct 13.

Eight-year clinical evaluation of a 2-step self-etch adhesive with and without selective enamel etching.

Peumans M[1], De Munck J, Van Landuyt KL, Poitevin A, Lambrechts P, Van Meerbeek B.

Author information

Abstract

OBJECTIVES: The objective of this randomized controlled clinical trial was to evaluate the 8-year clinical performance of a mild 2-step self-etch adhesive in non-carious Class-V lesions with and without prior selective phosphoric acid-etching of the enamel cavity margins.

METHODS: A total of 100 non-carious Class-V lesions in 29 patients were restored with Clearfil AP-X (Kuraray). The composite restorations were bonded following two different approaches: (1) application of Clearfil SE (Kuraray) following a self-etch approach (control group; C-SE non-etch), (2) selective phosphoric acid-etching of the enamel cavity margins before application of Clearfil SE (experimental group; C-SE etch). The restorations were evaluated after 6 months, 1, 2, 3, 5 and 8 years of clinical service regarding their retention, marginal integrity and discoloration, caries occurrence, preservation of tooth vitality and post-operative sensitivity.

RESULTS: The recall rate at 8 years was 76%. Only two restorations, one of the C-SE non-etch group and one of the C-SE etch group, were clinically unacceptable due to loss of retention leading to a retention rate and a clinical success rate of 97% in both groups. Aging of the restorations was characterized by an increase in the percentage of restorations with a small but clinically acceptable marginal defect (C-SE non-etch: 92%; C-SE etch: 84%) and/or a superficial marginal discoloration (C-SE non-etch: 44%; C-SE etch: 28%). At the enamel side, the presence of small marginal defects (C-SE non-etch: 86%; C-SE etch: 65%) and superficial marginal discoloration (C-SE non-etch: 11%; C-SE etch%) was more frequently noticed in the control group than in the experimental group. The difference, however, was only statistically significant for the presence of superficial marginal discoloration (McNemar, p=0.01).

SIGNIFICANCE: After 8 years of clinical functioning, the clinical effectiveness of Clearfil SE remained excellent, with selective acid-etching of the enamel cavity margins only having some minor positive effect on marginal integrity and absence of marginal discoloration at enamel.

Copyright © 2010 Academy of Dental Materials. Published by Elsevier Ltd. All rights reserved.

PMID: 20947155 DOI: 10.1016/j.dental.2010.08.190

Five-year follow-up of implant-supported all-ceramic FDPs. A randomized clinical trial.

Five-year follow-up of implant-supported all-ceramic FDPs. A randomized clinical trial.

Objectives: The purpose of this study was to evaluate the clinical performance of two- to five-unit implant-supported all-ceramic restorations and to compare the results of two different all-ceramic systems, Denzir® (DZ) and In-Ceram Zirconia® (InZ). Methods: Eighteen patients were treated with a total of 25 two- to five-unit implant-supported fixed dental prostheses. Nine patients were given restorations of the DZ system and the other nine were given restorations of the InZ system. The restorations were cemented with zinc phosphate cement onto customized titanium abutments and were evaluated after one, three and five years. Results: At the five-year follow-up, all restorations were in function; none had fractured. Superficial cohesive (chip-off) fractures were, however, observed in 9 of the 18 patients (11 of 25 restorations). Sixteen units in the DZ group (9 of 13 restorations) and 3 in the InZ group (2 of 12 restorations) had chip-off fractures. The difference between the two groups regarding frequency of chip-off fractures was statistically significant ($P<0.05$ at FDP level and $P<0.001$ at unit level). Conclusion: The results suggest that all-ceramic implant-supported fixed dental prostheses of two- to five-units may be considered a treatment alternative. The DZ system as used in this study, however, exhibits an unacceptable amount of veneering porcelain fractures and thus cannot be recommended for the type of treatment evaluated in this trial. Poor compatibility or problems with the bond mechanisms between veneer and framework could not explain the chip-off fractures. Factors concerning veneering porcelain need to be further evaluated.

Division: IADR/PER General Session
Meeting: 2010 IADR/PER General Session (Barcelona, Spain)
Location: Barcelona, Spain
Year: 2010
Final Presentation ID: 2112

Authors

- Larsson Christel (Malmo University, Malmo, N/A, Sweden)
- Vult Von Steyern Per (Malmo University, Malmo, N/A, Sweden)

SESSION INFORMATION
Oral Session
Clinical Performance: All-ceramic Restorations
07/16/2010

Five-year follow-up of implant-supported all-ceramic FDPs. A randomized clinical trial.

Int J Prosthodont. 2010 Nov-Dec;23(6):555-61.

Five-year follow-up of implant-supported Y-TZP and ZTA fixed dental prostheses. A randomized, prospective clinical trial comparing two different material systems.

Larsson C[1], Vult von Steyern P.

Author information

Abstract

PURPOSE: The aim of this study was to evaluate the clinical performance of two- to five-unit implant-supported all-ceramic restorations and to compare the results of two different all-ceramic systems, Denzir (DZ) and In-Ceram Zirconia (InZ).

MATERIALS AND METHODS: Eighteen patients were treated with a total of 25 two- to five-unit implant-supported fixed dental prostheses. Nine patients were given DZ system restorations and 9 were given InZ system restorations. The restorations were cemented with zinc phosphate cement onto customized titanium abutments and were evaluated after 1, 3, and 5 years.

RESULTS: At the 5-year follow-up, all restorations were in function; none had fractured. However, superficial cohesive (chip-off) fractures were observed in 9 of 18 patients (11 of 25 restorations). Sixteen units in the DZ group (9 of 13 restorations) and 3 in the InZ group (2 of 12 restorations) had chip-off fractures. The difference between the two groups regarding frequency of chip-off fractures was statistically significant ($P < .05$ at the FDP level and $P < .001$ at the unit level).

CONCLUSION: The results suggest that all-ceramic implant-supported fixed dental prostheses of two to five units may be considered a treatment alternative. The DZ system, however, exhibited an unacceptable amount of veneering porcelain fractures and thus cannot be recommended for the type of treatment evaluated in this trial. Poor compatibility or problems with the bond mechanisms between the veneer and framework could not explain the chip-off fractures. Stress distribution, as well as other factors concerning the veneering porcelain, need to be evaluated further.

PMID: 21209993

HOME MEETINGS ADVANCED SEARCH INSTRUCTIONS

Laser stimulation for acupuncture points as temporomandibular chronic symptoms treatment

Traditional acupuncture is a kind of therapies to temporomandibular disorders (TMD) pain control. The low power laser stimulus is an alternative to needles in acupuncture points, however, studies using this feature are still scarce. Objective: this study evaluated the performance of laser acupuncture in combination the occlusal splint (OS) therapy in chronic orofacial pain patients originating from TMD. Methods: according to the Research Diagnostic Criteria for TMD (RDC/TMD) were selected thirty female patients with myofascial pain and arthralgia diagnostic, aged 20 to 40 years. They were divided into 2 groups: the study group received OS and laser acupuncture (infrared laser, 50mW for 90s) while the control group received OS and placebo (non therapeutic LED light). The acupuncture points were selected: S6, SI19, GB20, LI4, L3, SJ3, GB34 and EX-HN3. The clinical evolution of the two groups was compared during extraoral, intraoral muscle and joint palpation, it was registered by the visual analogue scale (VAS). Results: the scores obtained from the study group showed lower final pain threshold compared to the control group. Statistical analysis of variance with repeated measures showed a significant difference (p<0.05) about muscle and joint structures evaluated. Conclusion: the TMD chronic symptoms control was influenced by the synergistic action from laser acupuncture treatment to OC therapy.

Division: IADR/PER General Session
Meeting: 2010 IADR/PER General Session (Barcelona, Spain)
Location: Barcelona, Spain
Year: 2010
Final Presentation ID: 4914

Authors

- Ferreira Luciano Ambrosio (Universidade Federal De Juiz De Fora, Juiz de Fora, Minas Gerais, N/A, Brazil)
- Paula Marcos Vinicius Queiroz (Universidade Federal De Juiz De Fora, Juiz de Fora - Minas Gerais, N/A, Brazil)

SESSION INFORMATION
Poster Session
Temporomandibular Disorders and Sleep Apnea: Treatments
07/17/2010

Back Print Friendly

Laser stimulation for acupuncture points as temporomandibular chronic symptoms treatment

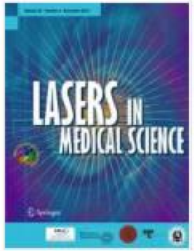

Lasers in Medical Science
November 2013, Volume 28, Issue 6, pp 1549–1558 | Cite as

Laser acupuncture in patients with temporomandibular dysfunction: a randomized controlled trial

Authors | Authors and affiliations

Luciano Ambrosio Ferreira, Rodrigo Guerra de Oliveira, .

Marcos Vinicius Queiroz De Paula

Original Article
First Online: 05 February 2013

4 Shares | 1.2k Downloads | Cit

A prospective, double-blind, randomized, and placebo-controlled trial was conducted in patients with chronic temporomandibular disorder (TMD) to check the analgesic efficacy of infrared low-power GaAlAs diode laser applied to acupuncture points. Forty female subjects, ranging in age from 20 to 40 years, with diagnoses of chronic myofascial pain and arthralgia were randomly allocated to two groups: an experimental group (EG) who received the laser acupuncture as adjunct to reversible occlusal splint therapy and a control group (CG) who received a placebo laser associated with occlusal splint therapy. Both approaches were applied once a week for 3 months. Laser acupuncture was defined by the following parameters: 50-mW continuous radiation for 90 s to acupoints ST6, SI19, GB20, GB43, LI4, LR3, NT3, and EX-HN3; defining 4.5-J energy; 1250-W/cm^2 density point; and 112.5-J/cm^2 total density. The outcome measurements included a symptom evolution assessment carried out by checking spontaneous and palpation pain intensity, which was indicated on a visual analog scale (VAS). All evaluations were made by an assessor who was blind to the treatment. The symptom reduction was significant in both groups (EG: VAS = 0, n = 20; CG: VAS between 2 and 4, n = 18). The measurements showed significantly faster and lower pain intensity values in the EG ($p \leq 0.002$), where there was a higher proportion of patients with remission of symptoms related to the action of laser acupuncture. For patients in whom conservative treatment was adopted, the laser acupuncture is a secure, noninvasive, and effective treatment modality because it improves the chronic pain associated with TMD and has no side effects.

Lingualized occlusion has advantage for severe alveolar ridge resorption

Objectives: To assess the influence of mandibular residual ridge resorption (RRR) on objective masticatory measures of two occlusal schemes: lingualized occlusion (LO) and fully bilateral balanced articulation (FBBA). Methods: The enrolled patients (n = 22) were randomly allocated one set of complete dentures with either LO or FBBA. Maximum occlusal force, masticatory performance (by the MPI), and mandibular movements were measured at 3- and 6-month follow-ups. Mandibular RRR was assessed as the sum of the mandibular bone height at the midline, first premolar region, and least vertical height region, and from the mental foramen to the alveolar crest, measured on panoramic radiographs; the treatment groups were subclassified into severe or moderate RRR subgroups by the value of the sum of individual measurements. Results: Significant differences were observed in the between-subgroup comparisons (Kruskal–Wallis test) of the MPI (3 months, p = 0.01; 6 month, p = 0.04) and linear deviation from intercuspal position (anterior–posterior: 6 months, p = 0.01; inferior–superior: 3 months, p = 0.008; 6 month, p = 0.02). The patients with severe RRR in the FBBA group showed a significant decrease in the MPI and increase in linear inferior deviation from intercuspal position at 3 months (post hoc comparison) as well as a significant increase in the linear posterior and inferior deviation from intercuspal position at 6 months. Conclusions: LO is the preferable occlusal scheme for patients with severe RRR.

Division: IADR/PER General Session
Meeting: 2010 IADR/PER General Session (Barcelona, Spain)
Location: Barcelona, Spain
Year: 2010
Final Presentation ID: 2164

Authors

- Kawai Yasuhiko (Nihon University, Matsudo, Chiba, N/A, Japan)
- Matsumaru Yuichi (Nihon University school of Dentistry at Matsudo, Chiba, N/A, Japan)
- So Kunio (Nihon University school of Dentistry at Matsudo, Chiba, N/A, Japan)
- Gunji Atsuko (Nihon University school of Dentistry at Matsudo, Chiba, N/A, Japan)

SESSION INFORMATION
Poster Session
Clinical Topics in Prosthodontic Research II
07/16/2010

Lingualized occlusion has advantage for severe alveolar ridge resorption

J Prosthodont Res. 2010 Jul;54(3):112-8. doi: 10.1016/j.jpor.2009.11.008. Epub 2010 Jan 19.

Influence of mandibular residual ridge resorption on objective masticatory measures of lingualized and fully bilateral balanced denture articulation.

Matsumaru Y[1].

Author information

Abstract

PURPOSE: To assess the influence of mandibular residual ridge resorption (RRR) on objective masticatory measures of two occlusal schemes: lingualized occlusion (LO) and fully bilateral balanced articulation (FBBA).

METHODS: The enrolled patients (n=22) were randomly allocated one set of complete dentures with either LO or FBBA. Maximum occlusal force, masticatory performance (by the MPI), and mandibular movements were measured at 3- and 6-month follow-ups. Mandibular RRR was assessed as the sum of the mandibular bone height at the midline, first premolar region, and least vertical height region, and from the mental foramen to the alveolar crest, measured on panoramic radiographs; the treatment groups were subclassified into severe or moderate RRR subgroups by the value of the sum of individual measurements.

RESULTS: Significant differences were observed in the between-subgroup comparisons (Kruskal-Wallis test) of the MPI (3 months, p=0.01; 6 months, p=0.04) and linear deviation from intercuspal position (anterior-posterior: 6 months, p=0.01; inferior-superior: 3 months, p=0.008; 6 months, p=0.02). The patients with severe RRR in the FBBA group showed a significant decrease in the MPI and increase in linear inferior deviation from intercuspal position at 3 months (post hoc comparison) as well as a significant increase in the linear posterior and inferior deviation from intercuspal position at 6 months.

CONCLUSIONS: LO is the preferable occlusal scheme for patients with severe RRR.

TRIAL REGISTRATION: ClinicalTrials.gov NCT00959530.

Copyright 2009 Japan Prosthodontic Society. Published by Elsevier Ltd. All rights reserved.

PMID: 20089469 DOI: 10.1016/j.jpor.2009.11.008

Long-Term Efficacy of Resilient Appliance Therapy in TMD Patients

Objectives: To investigate long-term efficacy of a resilient appliance in patients with pain due to temporomandibular disorders (TMD). Methods: A randomized, controlled trial was performed in 80 recruited TMD pain patients. They were randomly allocated to one of two groups: treatment with a resilient appliance or treatment with a hard, palatal, non-occluding appliance. The primary treatment outcome measure was judged positive when patients' characteristic pain intensity decreased by at least 30%. Additional treatment outcomes were physical functioning, emotional functioning and headache. Number needed to treat was measured on the basis of primary treatment outcome at 12 months. Results: At 12 months follow-up 50% of the patients in the treatment group and 42% in the control group had a 30% reduction of characteristic pain intensity, when calculated as intent to treat analysis. Jaw function improved in both groups at 6 and 12 month follow-up. Emotional function improved in both groups at 6 months follow-up; an improvement concerning grade of depression was found in the control group at 12 month. Headache decreased in both groups at both follow-ups. There were no statistically significant differences found regarding primary and additional outcomes between groups at 6 and 12 months follow-up. NNT was 14 when calculated on a 30% reduction of CPI at 12 months follow-up. Conclusion: There was no statistically significant difference between the resilient appliance and the non-occluding control appliance in reducing TMD pain, physical functioning, emotional functioning and headache in a 12 months perspective.

Division: IADR/PER General Session
Meeting: 2010 IADR/PER General Session (Barcelona, Spain)
Location: Barcelona, Spain
Year: 2010
Final Presentation ID: 3606

Authors

- Nilsson Håkan (Malmö University, Faculty of Odontology, Malmö, N/A, Sweden)
- Vallon Danila (Malmö University, Faculty of Odontology, Malmö, N/A, Sweden)
- Ekberg Ewacarin (Malmö University, Faculty of Odontology, Malmö, N/A, Sweden)

SESSION INFORMATION
Oral Session
Orofacial Pain and TMD: Treatment
07/17/2010

Long-Term Efficacy of Resilient Appliance Therapy in TMD Patients

Journal of Oral Rehabilitation

Long-term efficacy of resilient appliance therapy in TMD pain patients: a randomised, controlled trial

H. NILSSON, D. VALLON, E. C. EKBERG

First published: 22 March 2011 | https://doi.org/10.1111/j.1365-2842.2011.02210.x | Cited by: 5

Dr Håkan Nilsson, Department of Stomatognathic Physiology, Faculty of Odontology, Malmö University, SE-205 06 Malmö, Sweden. E-mail: Hakan.Nilsson@mah.se

Abstract

Summary The aim was to investigate long-term efficacy of a resilient appliance in patients with pain due to temporomandibular disorders (TMD). A randomised, controlled trial was performed in 80 recruited TMD pain patients. They were randomly allocated to one of two groups: treatment with a resilient appliance or treatment with a hard, palatal, non-occluding appliance. The primary treatment outcome was judged positive when patients' characteristic pain intensity decreased by at least 30%. Additional treatment outcomes were physical functioning, emotional functioning and headache. At the 12-month follow-up 50% of the patients in the treatment group and 42% in the control group had a 30% reduction of characteristic pain intensity, when calculated in an intent-to-treat analysis. Jaw function improved in both groups at the 6- and 12-month follow-up. Emotional functioning improved in both groups at the 6-month follow-up; an improvement concerning grade of depression was found in the control group at 12 months. Headache decreased in both groups at both follow-ups. There were no statistically significant differences found regarding primary and additional outcomes between groups at the 6- and 12-months follow-up. There was no statistically significant difference between the resilient appliance and the non-occluding control appliance in reducing TMD pain, physical functioning, emotional functioning and headache in a 12 months perspective.

Mandibular 2-Implant Overdentures: The Stability and Magnitude of the Effect

Objectives: There is still controversy regarding the superiority of mandibualr 2-implant overdenture (IODs) over conventional complete dentures (CDs) in terms of quality of life. Furthermore, the magnitude and the stability of the effect remain uncertain. Therefore, this longitudinal study aimed to determine the magnitude and stability of effect of IODs on oral health-related quality of life (OHRQoL). Methods: 172 participants (mean age 71 ± 4.5 years) randomly received CDs or IODs, both opposed by conventional maxillary dentures. OHRQoL was measured using the Oral Health Impact Profile (OHIP-20) at baseline, one and two year post-treatment. Repeated measures general linear models were conducted to assess the effects of time, pre-treatment and socio-demographic factors, as well as their interactions with the type of prosthesis on the total OHIP and its domain scores. Results: Statistically significant improvement in OHRQoL was seen for both treatment groups (Wilks's Lambda = 0.473, F (1,151) = 157.31, p<0.0001). This improvement was maintained over the two years of the assessment. At both follow-ups, participants wearing IODs reported significantly better total OHIP scores than those wearing CDs with 1.5 times larger magnitude of effect. A significant interaction was found between the pre-treatment OHIP score and type of prosthesis (Wilks's Lambda = 0.834, F (1,151) = 31.00, p<0.0001). In the CD, baseline OHIP scores affected significantly the post-treatment scores. This effect was not found in the IOD. Conclusions: The effect of mandibular two-implant overdentures on oral health-related quality of life is stable over the two-year period. The large magnitude of the effect of this treatment supports its clinical relevance.
This study was funded by CIHR Grant #UCR-36052 and Straumann Canada Ltd.

Division: IADR/PER General Session
Meeting: 2010 IADR/PER General Session (Barcelona, Spain)
Location: Barcelona, Spain
Year: 2010
Final Presentation ID: 34

Authors

- Jabbour Zaher (Universite de Montreal, Montreal, QC, Canada)
- Emami Elham (Universite de Montreal, Montreal, QC, Canada)
- De Grandmont Pierre (Universite de Montreal, Montreal, QC, Canada)
- Rompre Pierre (Universite de Montreal, Montreal, QC, Canada)
- Feine Jocelyne (McGill University, Montreal, QC, Canada)

SESSION INFORMATION
Oral Session
Assessing Oral Health for Older Patients
07/14/2010

Mandibular 2-Implant Overdentures: The Stability and Magnitude of the Effect

Clin Oral Implants Res. 2012 Oct;23(10):1205-9. doi: 10.1111/j.1600-0501.2011.02289.x. Epub 2011 Aug 15.

Is oral health-related quality of life stable following rehabilitation with mandibular two-implant overdentures?

Jabbour Z[1], Emami E, de Grandmont P, Rompré PH, Feine JS.

⊕ Author information

Abstract

OBJECTIVES: The superiority of mandibular two-implant overdentures (IODs) over conventional complete dentures (CDs) in terms of quality of life is still questioned. Furthermore, the stability and magnitude of the treatment effect over time remain uncertain. This follow-up study aimed to determine the stability and magnitude of the effect of IODs on oral health-related quality of life (OHRQoL).

MATERIAL AND METHODS: 172 participants (mean age 71 ± 4.5 years) randomly received CDs or IODs, both opposed by conventional maxillary dentures. OHRQoL was measured using the Oral Health Impact Profile (OHIP-20) at baseline, 1 and 2 years post-treatment. Repeated measures ANOVAs were conducted to assess the effects of time and treatment on the total OHIP and its individual domain scores.

RESULTS: A statistically significant improvement in OHRQoL was seen for both treatment groups ($P < 0.001$). This improvement was maintained over the 2 year assessment. At both follow-ups, participants wearing IODs reported significantly better total OHIP scores than those wearing CDs ($P < 0.001$), with a 1.5 times larger magnitude of effect. In the CD group, baseline OHIP scores influenced the post-treatment scores ($P < 0.001$). This effect was not found in the IOD group.

CONCLUSIONS: The effect of mandibular two-IODs on OHRQoL is stable over a 2-year period. The large magnitude of effect of this treatment supports its clinical significance.

© 2011 John Wiley & Sons A/S.

PMID: 22092512 DOI: 10.1111/j.1600-0501.2011.02289.x

Nonsurgical Treatment of Peri-Implantitis: a Controlled Clinical Study

Objectives: The aim of this prospective, parallel group designed, controlled clinical study is to evaluate the effectiveness of an air-powder system for the treatment of peri-implantitis. Methods: Fourteen patients, each of whom displayed at least one implant with initial or moderate peri-implantitis, were randomly instrumented using either (1) an air-powder flow (glycin based powder) (APF) or (2) mechanical debridement using plastic curettes and antiseptic therapy with chlorhexidine digluconate (MDA). The following clinical parameters were measured at baseline and 3 months after treatment: bleeding on probing (BOP), clinical attachment level (CAL). Results: The sites treated with APF revealed a reduction of mean BOP values from 92.8% to 45.2% ($p<0.01$) and a CAL change from 5.1 mm to 4.2 mm ($p<0.01$) at 3 months, respectively. The sites treated with MDA revealed a reduction of mean BOP values from 100% to 49.9% ($p<0.01$) and a CAL change from 5.1 mm to 3.7 mm ($p<0.01$) at 3 months, respectively. Between groups comparisons of mean BOP and CAL values at baseline and after 3 months were statistically non significant ($p>0.05$), respectively. Conclusion: It was concluded that nonsurgical treatment of peri-implantitis using either APF or MDA may result in comparable short-term clinical results.

Division: IADR/PER General Session
Meeting: 2010 IADR/PER General Session (Barcelona, Spain)
Location: Barcelona, Spain
Year: 2010
Final Presentation ID: 1680

Authors

- Sahm Narja (Department of oral surgery Universitätsklinikum Düsseldorf, Düsseldorf, N/A, Germany)
- Santel Thore (Department of oral surgery Universitätsklinikum Düsseldorf, Düsseldorf, N/A, Germany)
- Becker Jürgen (Department of oral surgery Universitätsklinikum Düsseldorf, Düsseldorf, N/A, Germany)
- Schwarz Frank (Department of oral surgery Universitätsklinikum Düsseldorf, Düsseldorf, N/A, Germany)

SESSION INFORMATION
Poster Session
Microbiology
07/15/2010

Nonsurgical Treatment of Peri-Implantitis: a Controlled Clinical Study

Journal of Clinical Periodontology

EFP Official scientific Journal of the European Federation of Periodontology and its member National Societies

Non-surgical treatment of peri-implantitis using an air-abrasive device or mechanical debridement and local application of chlorhexidine: a prospective, randomized, controlled clinical study

Narja Sahm, Jürgen Becker, Thore Santel, Frank Schwarz

First published: 19 July 2011 | https://doi.org/10.1111/j.1600-051X.2011.01762.x | Cited by: 50

Address:
Frank Schwarz
Department of Oral Surgery
Westdeutsche Kieferklinik
Heinrich-Heine-University
Moorenstr.
5 D-40225 Düsseldorf
Germany
E-mail: frank.schwarz@med.uni-duesseldorf.de

Abstract

Objectives: The aim of this prospective, parallel group designed, randomized controlled clinical study was to evaluate the effectiveness of an air-abrasive device (AAD) for non-surgical treatment of peri-implantitis.

Material and Methods: Thirty patients, each of whom displayed at least one implant with initial to moderate peri-implantitis, were enrolled in an oral hygiene program (OHI) and randomly instrumented using either (1) AAD (amino acid glycine powder) or (2) mechanical debridement using carbon curets and antiseptic therapy with chlorhexidine digluconate (MDA). Clinical parameters were measured at baseline, 3 and 6 months after treatment [e.g. bleeding on probing (BOP), probing depth (PD), clinical attachment level (CAL)].

Results: At 6 months, AAD group revealed significantly higher ($p<0.05$; unpaired *t*-test) changes in mean BOP scores when compared with MDA-treated sites (43.5±27.7%*versus* 11.0±15.7%). Both groups exhibited comparable PD reductions (AAD: 0.6±0.6 mm *versus* MDA: 0.5±0.6 mm) and CAL gains (AAD: 0.4±0.7 mm *versus* MDA: 0.5±0.8 mm) ($p>0.05$; unpaired *t*-test, respectively).

Conclusions: Within its limitations, the present study has indicated that (i) both treatment procedures resulted in comparable but limited CAL gains at 6 months, and (ii) OHI+AAD was associated with significantly higher BOP reductions than OHI+MDA.

Occlusal contacts are affected by experimental jaw muscle pain

Objective: Aim of the study was to measure the effect of an experimental jaw muscle pain on number and position of posterior occlusal contacts. Methods: Eleven adult voluntary subjects (nine male, two female, age 25.2±2.3) were enrolled in this randomized cross-over study. Each subject participated in two experimental sessions (separated by 30 days) in which he/she received an injection in the right masseter muscle. Subjects were randomized with respect to the sequence of the substance injected, hypertonic (5.0%) or isotonic saline solution (0.9%), used respectively as painful and control substance. Three occlusal bite records at the maximal intercuspal position were obtained during each experimental session: before (baseline), during and ten minutes after the end of injection. Occlusal records were put on lower casts and posterior contacts were reported by different colors (black for baseline, red for intermediate and green for final contacts) and counted. Difference in color was assumed to be difference in position, and the intermediate contacts were consequently classified as confirmed, disappeared or new contacts. Repeated measures ANOVA was used to compare the overall number of contacts among groups and the number of contacts of different colors. Results: Means and standard deviations of number of contacts are reported in table. No significant difference was found between the overall number of occlusal contacts, but a significant difference (p=.03) was found between contacts according to different colors. That means the overall number of contacts did not change, but the position did. Conclusion: Within the limits of the present study (first of all the small number of subjects), an experimentally induced jaw muscle pain, under controlled conditions, seems to affect the position but not the number of posterior occlusal contacts.

	Baseline	Intermediate	Final	Confirmed	Disappeared	New
Experimental	17.27(0.78)	14.45(1.81)	16.09(0.72)	11.91(2)	5.36(1.53)	2.55(0.41)
Control	17.27(0.78)	17.27(0.75)	17(0.77)	17.09(0.74)	0	0.18(0.12)

Table. Means (sd) of occlusal contacts.

Division: IADR/PER General Session
Meeting: 2010 IADR/PER General Session (Barcelona, Spain)
Location: Barcelona, Spain
Year: 2010
Final Presentation ID: 2793

Authors

- Mobilio Nicola (University of Ferrara, Ferrara, N/A, Italy)
- Catapano Santo (University of Ferrara, Ferrara, N/A, Italy)

SESSION INFORMATION
Poster Session
Orofacial Morphology and Biomechanics in Jaw Motor Function and Dysfunctions
07/16/2010

Occlusal contacts are affected by experimental jaw muscle pain

J Oral Rehabil. 2011 Jun;38(6):404-9. doi: 10.1111/j.1365-2842.2010.02173.x. Epub 2010 Nov 5.

Effect of experimental jaw muscle pain on occlusal contacts.

Mobilio N[1], Catapano S.

Author information

Abstract

The aim of the study was measuring the effect of experimental jaw muscle pain on number and position of posterior occlusal contacts. Eleven adult voluntary subjects were enrolled. A lower impression was taken for each subject and two dental casts were obtained from each impression. The study was carried out in a randomised cross-over fashion. Each subject participated in two experimental sessions (30-day interval) in which he/she received an injection in the central part of the right masseter muscle consisting of 0·5 mL of either hypertonic or isotonic saline. Each subject was asked to rate pain intensity on a visual analogue scale. Three occlusal bite checks (polyvinylsiloxane) at the maximal intercuspal position were obtained during the experimental session: the first before the injection, the second between 60 and 90 s after the injection and the third 15 min after the injection. Evaluation of contacts was performed on dental casts with the use of different colours (black for baseline, red for intermediate and green for final contacts). Repeated-measures analysis of variance was used to compare the overall number of contacts among groups and the number of contacts of different colours. No significant difference was found between the overall number of occlusal contacts ($P>0{\cdot}05$), but significant differences were found between contacts according to different colours: confirmed ($P=0{\cdot}006$), disappeared ($P=0{\cdot}007$) and new ($P<0{\cdot}001$). Assuming different colours as change in contact position, the overall number of contacts did not change, but the position did. Experimentally induced jaw muscle pain affected the pattern of posterior occlusal contacts.

PMID: 21054484 DOI: 10.1111/j.1365-2842.2010.02173.x

Palmitoylethanolamide Vs NSAID In The Treatment Of TMJD Pain

Objectives: The aim of this study was to compare the efficacy of palmitoylethanolamide (PEA) and non-steroidal antinflammatory drugs (NSAID) in the treatment of pain caused by temporomandibular joint disorders (TMJD). PEA is an endogenous agent (fatty acid amide) and its mechanism is an autacoid local inflammation antagonism. It is a regulator of mast cells behavior in the control of both acute and chronic inflammatory disease.
Materials and Methods: A blind randomized clinical trial has been conduced on 25 patients (17 female and 8 male) aged 24-54, selected in a group of 120 subjects referred to Dental Department of the University of Bologna. This group of patients affected by temporomandibular joint's (TMJ) osteoarthritis or synovitis pain (axis I RDC/TMD) was randomly divided in 2 groups. Group A (13 subjects) received PEA (Normast) 300 mg in the morning and 600 mg in the evening for 7 days and then 300 mg twice a day for other 7 days. Group B (12 subject) took ibuprofen 600 mg 3 times a day for 2 weeks. Every patient recorded the intensity of their spontaneous pain using Visual Analogue Scale (VAS) twice a day (in the morning and evening) noting data in a diary. Maximum mouth opening was recorded by a blind operator during the first visit and after 14 days.

Results: Mann-Whitney test was used to compare the course of pain during treatment. Pain decrease after two weeks of treatment was significantly higher in group A (PEA) than in group B (NSAID) (p=0.0001); masticatory function improves more in group A than in Group B (p=0.0001).

Conclusions: Our preliminary data suggest that PEA is an effective tool for treatment of TMJD, in patients with synovitis and osteoarthritis pain. It is a non-gastrolesive effective analgesic with a longer half-life than NSAID (12 versus 4 hours).

Division: IADR/PER General Session
Meeting: 2010 IADR/PER General Session (Barcelona, Spain)
Location: Barcelona, Spain
Year: 2010
Final Presentation ID: 2137

Authors

- Bortolotti Francesco (University of Bologna, Bologna, N/A, Italy)
- Russo Mariagnese (University of Bologna, Bologna, N/A, Italy)
- Bartolucci Maria Lavinia (University of Bologna, Bologna, N/A, Italy)
- Alessandri Bonetti Giulio (University of Bologna, Bologna, N/A, Italy)
- Gatto Maria Rosaria (University of Bologna, Bologna, N/A, Italy)
- Marini Ida (University of Bologna, Bologna, N/A, Italy)

SESSION INFORMATION
Poster Discussion Session
Pharmacology, Therapeutics, & Toxicology (PDS)
07/16/2010

Palmitoylethanolamide Vs NSAID In The Treatment Of TMJD Pain

J Orofac Pain. 2012 Spring;26(2):99-104.

Palmitoylethanolamide versus a nonsteroidal anti-inflammatory drug in the treatment of temporomandibular joint inflammatory pain.

Marini I[1], Bartolucci ML, Bortolotti F, Gatto MR, Bonetti GA.

Author information

Abstract

AIMS: To carry out a randomized clinical trial to compare the effect of palmitoylethanolamide (PEA) versus ibuprofen, a nonsteroidal anti-inflammatory drug (NSAID), for pain relief in temporomandibular joint (TMJ) osteoarthritis or arthralgia. PEA acts as an endogenous agent with an autacoid local inflammation antagonism and modulates mast cell behavior controlling both acute and chronic inflammation.

METHODS: A triple-blind randomized clinical trial was conducted on 24 patients (16 women and 8 men) aged 24 to 54 years and suffering from TMJ osteoarthritis or arthralgia. The patients were enrolled from a group of 120 consecutive patients referred to the University of Bologna's Department of Orthodontics. Patients were randomly divided into two groups: group A (12 subjects) received PEA 300 mg in the morning and 600 mg in the evening for 7 days and then 300 mg twice a day for 7 more days. Group B (12 subjects) received ibuprofen 600 mg three times a day for 2 weeks. Every patient recorded the intensity of spontaneous pain on a visual analog scale twice a day. Maximum mouth opening was recorded by a blind operator during the first visit and again after the 14th day of drug treatment. A t test was used for data comparisons.

RESULTS: Pain decrease after 2 weeks of treatment was significantly higher in group A than in group B (P = .0001); maximum mouth opening improved more in group A than in group B (P = .022).

CONCLUSION: These data suggest that PEA is effective in treating TMJ inflammatory pain.

PMID: 22558609

Randomized Controlled Clinical Trial of Endodontically-treated Teeth Followed-up to 3-

Randomized Controlled Clinical Trial of Endodontically-treated Teeth Followed-up to 3-years

Objectives: Glass-fiber posts are claimed to have more favorable mechanical properties than cast gold post-and-cores when restoring endodontically treated teeth. Aim of this RCT is to investigate whether cast post-and-cores can still be considered the 'gold-standard' to restore endodontically treated teeth or if adhesive post-and-core techniques do perform clinically better.

Methods: 144 patients in need for a restoration on an endodontically treated tooth were followed-up for 7-37 months (mean: 21±9 months). 205 restorations were placed and allocated to one of the following treatment groups: A) cast gold post-and-core (control, Medior 3, Cendres+Métaux); B) prefabricated glass-fiber post (Parapost-FiberLux, Coltène-Whaledent); C) custom-made glass-fiber posts (EverStick, StickTech); and D) composite core without post (Clearfil AP-X, Kuraray). All post-and-cores were covered by an all-ceramic crown (Procera, Nobel-Biocare). Allocation was randomized and based on the remaining tissue available for bonding as follow:

Dental tissue left	Randomization
a. Sufficient (⩾2 walls with 2mm thickness)	Group A/D
b. Insufficient (<2 walls with 2mm thickness)	Group A/B (small root canal)
	C (wide root canal)

Failures were distinguished as 'absolute' in case of fractures of the root or irreparable fractures of the core build-up, as 'relative' in case of loss of post retention or reparable fractures of the core build-up. 'Other' failures included endodontic or periodontal complications. Kaplan-Meier survival curves were drawn.

Results: The recall rate at 3 years was 97.1%. No significant differences could be observed among the 4 groups evaluated, because of few failures. The survival probability was 97.3% for 'absolute' failures due to 2 root fractures, and 94.1% for 'relative' failures due to 3 retention losses of post-and-core and 1 post fracture. One tooth failed because of endodontic complications.

Conclusion: Up to 3-years, both cast gold and adhesive post-and-core techniques performed clinically equally successfully, although longer follow-up times may be needed to determine significant differences.

Division: IADR/PER General Session
Meeting: 2010 IADR/PER General Session (Barcelona, Spain)
Location: Barcelona, Spain
Year: 2010
Final Presentation ID: 405

Authors

- Zicari, Francesca (Katholieke Universiteit Leuven, Leuven, N/A, Belgium)
- Van Meerbeek, B. (Katholieke Universiteit Leuven, Leuven, N/A, Belgium)
- Debels, E. (Katholieke Universiteit Leuven, Leuven, N/A, Belgium)
- Naert, Ignace (Katholieke Universiteit Leuven, Leuven, N/A, Belgium)

Zicari, Francesca (Katholieke Universiteit Leuven, Leuven, N/A, Belgium)
Van Meerbeek, B. (Katholieke Universiteit Leuven, Leuven, N/A, Belgium)
Debels, E. (Katholieke Universiteit Leuven, Leuven, N/A, Belgium)
Naert, Ignace (Katholieke Universiteit Leuven, Leuven, N/A, Belgium)

SESSION INFORMATION
Oral Session
Clinical Research: Glass Ionomers, Endodontic Materials and Equipment
07/15/2010

Randomized Controlled Clinical Trial of Endodontically-treated Teeth Followed-up to 3-years

A randomized controlled clinical trial of restored endodontically treated teeth up to 3 years

来自lirias.kuleuven.be

收藏 引用 批量引用 报错 分享

作者 F Zicari , B Van Meerbeek , E Debels , E Lesaffre , I Naert

摘要 A randomized controlled clinical trial of restored endodontically treated teeth up to 3 yearsZicari, FVan Meerbeek, BDebels, ELesaffre, EmmanuelNaert, IQuintessence Pub. Co.International Journal of Prosthodontics...

出版源 《International Journal of Prosthodontics》 , 2011 , 24 :363-372

被引量 0

找不到摘要

Stratified RCT Comparing Implant Stability of 3 Load Timing Groups

Stratified RCT Comparing Implant Stability of 3 Load Timing Groups

3 year Stratified RCT (SRCT) to evaluate the effect of loading regimen on implant stability and marginal bone height. Objectives: Assign implants into load groups based on ITV (insertion torque value)and measure differences in ISQ (implant stability) over time. Methods:Subjects needing single tooth implants (4mm diameter x 11mm or 13mm Osseospeed®; Astra Tech AB) in the premolar and molar region (N=40) were recruited with IRB approved criteria. Based on implant ITV (Elcomed, W&H) subjects were assigned to one of three loading groups (Immediate, 6 or 12 weeks). Implants placed with > or equal to 20Ncm (High ITV) were randomized to all three load groups, those with 10 to < 20Ncm (Intermediate ITV) were randomized to 6 or 12 week loading and those with < 10Ncm (Low ITV) defaulted to 12 wk loading. Outcomes measured every 2 weeks through 16 weeks included ISQ(Osstell Mentor)in 2 directions, clinical mobility, and tissue depths. At 16 weeks, and every year crestal bone height was measured. Results:

Implant Group	Immediate Load	6 week Load	12 week Load	Totals
ITV 0 to <10 Ncm	-	-	7	7
ITV 10 to <20 Ncm	-	11	2	13
ITV 20 + Ncm	8	6	6	20
Bone Type 1	1	-	-	1
Type 2	5	3	4	12
Type 3	2	8	10	20
Type 4	-	5	2	7

38 patients have completed all 3 years. All 3 Load Group implants increased in stability over 16 weeks. There was no statistically significant difference in stability between the 3 load groups by 4 weeks up to 16 weeks. Wilcoxon Rank Sum Test (p<.05)was used to measure correlation between ITV and bone type and correlation of ISQ (baseline) and bone type. Significant differences in ITV was found for all bone type groups. There was also a significant difference in ISQ(baseline) for bone types 1&2 vs 4, and Type 3 vs 4. A significant but weak correlation existed for ITV and ISQ (r=0.4973) with Spearman Rank Test. There was no difference in bone height between all 3 load groups. Conclusions: Physiologic load timing can be determined based on ITV and evaluated over time with ISQ for Osseospeed implant.

Division: IADR/PER General Session
Meeting: 2010 IADR/PER General Session (Barcelona, Spain)
Location: Barcelona, Spain
Year: 2010
Final Presentation ID: 3682

Authors

- Barewal Reva (Oregon Health & Science University, Happy Valley, OR, USA)
- Stanford Clark (University of Iowa, Iowa City, IA, USA)
- Weesner Ted (Oregon Health & Science University, Happy Valley, OR, USA)

SESSION INFORMATION
Oral Session
Implant Stability
07/17/2010

Stratified RCT Comparing Implant Stability of 3 Load Timing Groups

Int J Oral Maxillofac Implants. 2012 Jul-Aug;27(4):945-56.

A randomized controlled clinical trial comparing the effects of three loading protocols on dental implant stability.

Barewal RM[1], Stanford C, Weesner TC.

⊕ **Author information**

Abstract

PURPOSE: The primary goal of this stratified randomized controlled trial (SRCT) was to compare the stability of dental implants placed under three different loading regimens during the first 16 weeks of healing following implant placement. Implants were loaded immediately, early (6 weeks), or with conventional/delayed timing (12 weeks). Secondary outcomes were to compare marginal bone adaptation for 3 years after placement.

MATERIALS AND METHODS: Single posterior implant sites in the maxilla or mandible were examined. The insertion torque value was the primary determinant of load assignment. Resonance frequency analysis was performed at follow-up appointments for the first 16 weeks (with results provided as implant stability quotients [ISQs]). Marginal bone levels were assessed via radiographs.

RESULTS: Forty patients each received a single 4.0-mm diameter dental implant between 2004 and 2007. One implant failure occurred in Lekholm and Zarb type 4 bone with insertion torque value (ITV) of < 8.1 Ncm; the cumulative success rate was 97.5%. All implants, when classified by bone and loading type, increased in stability over time, with a minor reduction of 1.3 ISQ units seen at 4 weeks in the immediate loading group. The mean marginal bone loss over 3 years was 0.22 mm. The mean ITVs at implant placement for bone types 1 and 2 (grouped together), 3, and 4 were 32, 17, and 10, respectively, and were significantly different ($P < .05$).

CONCLUSIONS: ITV was a good objective measure of bone type. Using an ITV of 20 Ncm as the determinant for immediate loading and an ITV of 10 Ncm or greater as the determinant for early loading provided long-term success for this implant and led to no negative changes in tissue response. All bone type groups and loading groups showed no reduction in stability during the first 4 months of healing.

PMID: 22848898

The randomized shortened dental arch study: 5-year complication rates

The randomized shortened dental arch study: 5-year complication rates

Objectives: Evidence concerning the prosthetic management of patients with bilaterally shortened dental arches is sparse. This multi-center-study aimed at generating data on outcomes and survival rates for two common treatment options, removable dental prostheses for molar replacement or no replacement (funded by German-Research-Association: WA831/2-1-6). Methods: Inclusion criteria of the RCT were: all molars of the study-arch were missing but at least both canines and one premolar in each quadrant were present. Two hundred-fifteen participants were randomly assigned to receive either removable dental prostheses retained by precision attachments (Mini SG, CMSA, Switzerland) including molar replacement (RDP-group) or restorations following the concept of a shortened dental arch (SDA-group). The follow-up was performed for all participants six weeks (baseline), 6 months and then annually after treatment. The follow-up treatment required in the study-arch was categorized as "minimal", "moderate" or "extensive" according to a modified classification of *Struder et al. (1998)* and analyzed by Kaplan-Meier. Results: From the 215 randomized participants 81 participants in the RDP-group (mean age 60±10, 41 female) and 69 participants in the SDA-group (mean age 60±10, 41 female) received allocated intervention. A mean number of 6.9 (RDP-group) and 3.5 (SDA-group) complications occurred during the 5-year observation time per patient. Most frequent events (as a percentage of all events occurred) in the RDP-group were replacement of plastic retention-insert (15.5%), caries (10.9%), relining (9.4%), recementation (7.9%), endodontic complications (7.3%); in the SDA-group caries (22.2%), endodontic complications (10.2%) and periodontitis (9.3%). Distribution of complications after 5-years according to the three categories (minimal / moderate / extensive) was 18%/ 27% / 55% for the RDP-group and 45%/ 29%/ 26% for the SDA-group. The differences between groups were statistically significant ($P \leqslant 0.03$, log-rank-test) for the categories “minimal” and “extensive”. Conclusions: The follow-up treatment required was more extensive for the RDP-group than for the SDA-group.

Division: IADR/PER General Session
Meeting: 2010 IADR/PER General Session (Barcelona, Spain)
Location: Barcelona, Spain
Year: 2010
Final Presentation ID: 3722

Authors

- Wolfart Stefan (Department of Prosthodontics and Dental Materials, Medical Faculty, RWTH Aachen, Germany, Aachen, N/A, Germany)
- Mundt Torsten (University of Greifswald, Greifswald, N/A, Germany)
- Kern Mattias (Christian-Albrechts University, Kiel, N/A, Germany)
- Klein V. (Department of Prosthetic Dentistry, University of Bonn, Bonn, N/A, Germany)
- Passia N. (Department of Prosthetic Dentistry, Albert-Ludwig University of Freiburg, Freiburg, N/A, Germany)
- Pospiech Peter (Saarland University, Homburg/Saar, N/A, Germany)
- Wöstmann Bernd (Justus-Liebig-University, Giessen, N/A, Germany)
- Busche E. (Department of Prosthetic Dentistry, Witten-Herdecke University, Witten, N/A, Germany)
- Gitt I. (2 Department of Prosthetic Dentistry and Dental Material Science, University of Leipzig, Leipzig, N/A, Germany)
- Sturmbaum M. (Department of Prosthetic Dentistry, Ludwig-Maximilians University, München, N/A, Germany)
- Luthardt Ralph (University Ulm, Ulm, N/A, Germany)
- Lazarek K (Carl Gustav Carus, Dresden, N/A, Germany)
- Walter Michael H. (Carl Gustav Carus, Dresden, N/A, Germany)
- Marre B (Carl Gustav Carus, Dresden, N/A, Germany)
- Gerss J. (Department of Medical Informatics and Biomathematics, University of Münster, Muenster, N/A, Germany)
- Hannak Wolfgang B. (Charité Universitätsmedizin Berlin, Berlin, N/A, Germany)
- Heydecke G. (University Medical Center Eppendorf, Department of Prosthodontics, Hamburg, N/A, Germany)
- Hartmann Sinsa (Department of Prosthodontics, University Medical Center of the Johannes Gutenberg-University, Mainz, N/A, Germany)
- Huppertz J. (Department of Prosthetic Dentistry, Julius-Maximilians University of Wuerzburg, Wuerzburg, N/A, Germany)
- Jahn F. (Department of Prosthetic Dentistry and Dental Material Science, Friedrich-Schiller University of Jena, Jena, N/A, Germany)

SESSION INFORMATION
Oral Session
Factors in Clinical Success
07/17/2010

The randomized shortened dental arch study: 5-year complication rates

J Dent Res. 2012 Jul;91(7 Suppl):65S-71S.

The randomized shortened dental arch study: 5-year maintenance.

Wolfart S[1], Marré B, Wöstmann B, Kern M, Mundt T, Luthardt RG, Huppertz J, Hannak W, Reiber T, Passia N, Heydecke G, Reinhardt W, Hartmann S, Busche E, Mitov G, Stark H, Pospiech P, Weber A, Gernet W, Walter MH.

Author information

1 Department of Prosthodontics and Biomaterials, Medical Faculty, RWTH Aachen University, Pauwelsstraße 30, 52074 Aachen, Germany.

Abstract

The scientific evidence concerning prosthodontic care for the shortened dental arch (SDA) is sparse. This randomized multicenter study aimed to compare two common treatment options: removable partial dental prostheses (RPDPs) for molar replacement vs. no replacement (SDA). One of the hypotheses was that the follow-up treatment differs between patients with RPDPs and patients with SDAs during the 5-year follow-up period. Two hundred and fifteen patients with complete molar loss in one jaw were included in the study. Molars were either replaced by RPDPs or not replaced according to the SDA concept. A mean number of 4.2 (RPDP) and 2.8 (SDA) treatments for biological or technical reasons occurred during the 5-year observation time per patient. Concerning the biological aspect, no significant differences between the groups could be shown, whereas treatment arising from technical reasons was significantly more frequent for the RPDP group. When the severity of treatment was analyzed, a change over time was evident. When, at baseline, only follow-up treatment with minimal effort is required, over time there is a continuous increase to moderate and extensive effort observed for both groups (Controlled-trials.com number ISRCTN97265367).

PMID: 22699671 DOI: 10.1177/0022034512447950

2011

24-month clinical evaluation of class-V restorations with two different composites

24-month clinical evaluation of class-V restorations with two different composites

Objectives: Throughout the Nov-07/Jan-08 time period, 60 class-V restorations have been performed. Methods: Patients were to satisfy strict inclusion/exclusion criteria in order to be part of the study. In particular, every patient was to need at least two restorations on vital-teeth, which had to be located on opposite quadrants of the oral cavity. In each patient all the restorations were made with microlayering technique using SwissMasterLight (EMS) curing light (800mW/cm2 for 20s). The test groups were randomly assigned to the class V cavites using composite and adhesive materials as follows: testing material - Venus-Diamond and GlumaComfortBond (Heraeus) competitor - CeramX-Duo and Prime&Bond-NT (Dentsply) A quadrant-based randomization was used to assign to every restoration its filling material and adhesive system: restorations have been performed according to the adhesive dentistry techniques by one expert clinician (>10 years of clinical experience). Each restoration was polished with the 2-step polishing system provided by Heraeus-Kulzer. This system includes a pre-polshier and a high-gloss finisher. The polishing time used is be 20-sec with the pre-polisher and 40 sec with finisher. Results: Clinical evaluations were made by two independent clinicians with more than 80 % of agreement using a visual-loop (4.5x). Two year controls have been executed with USPHS evaluating system. None of the restorations received a Charlie rating. The restorations received 92% (Venus-Diamond) and 85% (CeramX-Duo) Alpha ratings for surface texture. The defects/gaps in marginal adaptation score was recorded as increased. Some restoration was lost, in testing-material 5% (Venus-Diamond) and in competitor-group 12 % (CeramX-Duo). We noticed a 23% in (A) and 25% in (B) rated Bravo for color matching ability. No restoration showed post-operative sensitivity or adverse events were reported. The total absence of post-operative sensibility has to be pointed out. Conclusions: In this clinical trial testing material behave as well as the competitor

Division: IADR/AADR/CADR General Session
Meeting: 2011 IADR/AADR/CADR General Session (San Diego, California)
Location: San Diego, California
Year: 2011
Final Presentation ID: 146

Authors

- Barabanti, Nicola (University of Brescia, Coccaglio, N/A, Italy)
- Madini, Lorenzo (University of Brescia, Cremona, N/A, Italy)
- Cerutti, Francesca (University of Brescia, Pisogne, N/A, Italy)
- Acquaviva, Pier Antonio (University of Brescia, Desenzano del Garda, N/A, Italy)
- Cerutti, Antonio (University of Brescia, Brescia, N/A, Italy)

Barabanti, Nicola (University of Brescia, Coccaglio, N/A, Italy)
Madini, Lorenzo (University of Brescia, Cremona, N/A, Italy)
Cerutti, Francesca (University of Brescia, Pisogne, N/A, Italy)
Acquaviva, Pier Antonio (University of Brescia, Desenzano del Garda, N/A, Italy)
Cerutti, Antonio (University of Brescia, Brescia, N/A, Italy)

SESSION INFORMATION
Oral Session
Clinical Studies: Direct Restorative Materials
03/16/2011

24-month clinical evaluation of class-V restorations with two different composites

24 Months Evaluation Of Class V Restorations With Different Composites

来自Elsevier

收藏 引用 批量引用 报错 分享

作者 P Mondina , N Barabanti , L Madini , PA Acquaviva , A Cerutti , ...

摘要 ABSTRACT Objectives: Study's aim was to test the clinical and esthetic performance of a composite flow resin system in comparison with a microhybrid composite resin system in the treatment of class V lesions in vital teeth Methods: Sixty (60) restorations have been carried out in thirty (30) patients respecting the inclusion-exclusion criteria and randomisation criteria for selection of technique and teeth. In each patient one restoration was made with micro-layering technique using Swiss Master Light (EMS) curing light (800 mW/cm2 for 20 s). The following test groups were randomly assigned to the class V cavites with two different composite materials and the same adhesive system Gluma Comfort Bond (Heraeus): A) Venus Diamond (Heraeus) B) Venus Diamond Flow (Heraeus). Three clinicians took part to this clinical study, one expert clinic operator and two independent evaluators with more than 80 % of clinical agreement using a loop visual (4.5x). Two year controls have been executed with USPHS evaluating system. Results: None of the restorations received a Charlie rating. Some restoration was lost: 5% (Venus-Diamond) and 7 % (Venus-Diamond Flow). The restorations received 92% (Venus-Diamond) and 100% (Venus-Diamond Flow) Alpha ratings for surface texture. The defects/gaps in marginal adaptation score was recorded as Alpha ratings 86% (Venus-Diamond) and 86% (Venus-Diamond Flow). We noticed a 23% in (Venus-Diamond) and 14 % (Venus-Diamond Flow) in (B) rated Bravo for color matching ability. No restoration showed post-operative sensitivity or adverse events were reported. The total absence of post-operative sensibility has to be pointed out. Conclusions: Within the limits of this clinical trial, to be continued with three year controls, the resin composite flow behave better if compared with microhybrid resin composite. We appreciate that color match, margin adapatation and surface texture obtained better scores in class V restorations, confirming a clinical 鈥渇eeling鈥 appreciated in 1 year results.

收起

出版源 Per/iadr Congress , 2012 , 31 :e51

Botulinum toxin type-A for treatment of persistent myofascial TMD pain

Objectives: Although botulinum toxins are used clinically for various pain conditions, evidence of their efficacy is still lacking. In the present randomized, placebo-controlled, cross-over multicenter study the efficacy of botulinum toxin-type A (BTX-A) was investigated in patients with persistent myofascial temporomandibular disorders (TMD). **Methods:** Twenty-one patients with myofascial TMD that had received conventional treatment for at least 6 months without adequate pain relief participated. 50U BTX-A dissolved in 0.5mL isotonic saline or 0.5mL isotonic saline alone were randomly injected into three standardized sites of the painful masseter muscles (maximum dose/patient = 100U). Follow-up was performed after 1 and 3 months, followed by a 1 month wash-out period where after cross-over was performed. Pain intensity at rest assessed on a 0-10 VAS was the primary outcome measure, while physical and emotional function, global improvement as well as side effects were secondary outcome measures. Clinical parameters (pain on palpation, pressure pain threshold, pain-free jaw opening) served as additional outcome measures. **Results:** BTX-A reduced the pain intensity (SD) by 30 (33)% after 1 month and by 23 (30)% after 3 months compared to 11 (40)% and 4 (33)%, respectively for saline (Repeated measures ANOVA; drug: p=0.005, time: p=0.06 interaction: p=0.187), but the number of patients that received a 30% pain reduction was not significantly larger for BTX-A than after saline at any follow-up visit. The number needed-to-treat (NNT) was 11 after 1 months and 7 after 3 months in favor for BTX-A. There were no significant changes after treatment in any other outcome measures, with the exception of pain on palpation that decreased significantly after saline. **Conclusion:** These results only lend weak support of an analgesic effect of BTX-A in patients with persistent myofascial TMD pain. Supported by Allergan Sweden AB.

Division: IADR/AADR/CADR General Session
Meeting: 2011 IADR/AADR/CADR General Session (San Diego, California)
Location: San Diego, California
Year: 2011
Final Presentation ID: 1625

Authors

- Ernberg Malin (Karolinska Institutet, Huddinge, N/A, Sweden)
- Hedenberg-magnusson Britt (Karolinska Institutet, Stockholm, N/A, Sweden)
- List Thomas (Malmo University, Malmoe, N/A, Sweden)
- Svensson Peter (School of Dentistry, Aarhus University, Aarhus C, N/A, Denmark)

SESSION INFORMATION
Oral Session
Keynote Address and Temporomandibular Disorders and Orofacial Pain Treatment
03/18/2011

Botulinum toxin type-A for treatment of persistent myofascial TMD pain

Efficacy of botulinum toxin type A for treatment of persistent myofascial TMD pain: A randomized, controlled, double-blind multicenter study

Malin Ernberg [a], Britt Hedenberg-Magnusson [a, b], Thomas List [c], Peter Svensson [d, e]

[a] Unit of Clinical Oral Physiology, Department of Dental Medicine, Karolinska Institutet, Box 4064, SE 141 04 Huddinge, Sweden
[b] Department of Stomatognathic Physiology, Eastman Institute, Dalagatan 11, SE 113 24 Stockholm, Sweden
[c] Department of Stomatognathic Physiology, Faculty of Dentistry, Malmö University, SE 212 22 Malmö, Sweden
[d] Department of Clinical Oral Physiology, School of Dentistry, Aarhus University, DK 8000 Aarhus, Denmark
[e] Center for Functionally Integrative Neuroscience (CFIN), MindLab, Aarhus University Hospital, Aarhus, Denmark

Received 12 December 2010, Revised 22 March 2011, Accepted 28 March 2011, Available online 22 April 2011.

Abstract

Evidence of an effect by botulinum toxins is still lacking for most pain conditions. In the present randomized, placebo-controlled, crossover multicenter study, the efficacy of botulinum toxin type A (BTX-A) was investigated in patients with persistent myofascial temporomandibular disorders (TMD). Twenty-one patients with myofascial TMD without adequate pain relief after conventional treatment participated. A total of 50 U of BTX-A or isotonic saline (control) was randomly injected into 3 standardized sites of the painful masseter muscles. Follow-up was performed after 1 and 3 months, followed by a 1-month washout period, after which crossover occurred. Pain intensity at rest was the primary outcome measure, while physical and emotional function, global improvement, side effects, and clinical measures were additional outcome measures. There was no main difference between drugs (ANOVA; $P = .163$), but there was a significant time effect ($P < .001$), so BTX-A reduced mean (SD) percent change of pain intensity by 30 (33%) after 1 month and by 23 (30%) after 3 months compared to 11 (40%) and 4 (33%) for saline. The number of patients who received a 30% pain reduction was not significantly larger for BTX-A than after saline at any follow-up visit. The number needed to treat was 11 after 1 month and 7 after 3 months. There were no significant changes after treatment in any other outcome measures, with the exception of pain on palpation, which decreased 3 months after saline injection ($P < .05$). These results do not indicate a clinical relevant effect of BTX-A in patients with persistent myofascial TMD pain.

Clinical efficacy of a castor bean solution as denture cleanser

Objective: This study analyzed the efficacy of a castor bean solution (*Ricinus communis*) as denture cleanser by means of a randomized crossover clinical trial. Methods: Fifty complete denture wearers were enrolled, instructed to brush their dentures 3x/day (specific brush and dentifrice) and immersed their dentures in the following solutions: A - Control: saline (20 min./day). B - alkaline peroxide solution Polident® (05 min/day). C - 1% sodium hypochlorite (20 min/day). D - castor bean (*Ricinus communis*) solution (20 min/day). All solutions were dispensed in white bottles without identification and the tablets were removed from package and dispensed in plastic bags. All volunteers used the four methods of hygiene for a period of 7 days each, according to a random sequence. After using the methods, the internal surface of the dentures was evidenced with 1% neutral red and photographed. The percentage of biofilm coverage area on the internal surface was measured by computer and used as the outcome variable. The data were analyzed by Friedman test (α=0.05). Results: It was observed that there was a significant difference between methods (Fr = 51.67; P<0.001); the method C promoted a lower percentage of biofilm when compared with the others products tested and it was significantly different from product A. By their turn, the methods B and D showed intermediate results. Conclusion: The castor bean solution was capable of reducing biofilm as well a proprietary alkaline peroxide-based denture cleanser, but not as 1% sodium hypochlorite. Results suggest that the experimental solution may be a efficacious denture cleanser, although limited as a disinfecting agent.

Division: IADR/AADR/CADR General Session
Meeting: 2011 IADR/AADR/CADR General Session (San Diego, California)
Location: San Diego, California
Year: 2011
Final Presentation ID: 2419

Authors

- Andrade Ingrid Machado (Universidade de São Paulo, Ribeirao Preto, N/A, Brazil)
- Andrade Kelly Machado (Universidade de São Paulo, Ribeirao Preto, N/A, Brazil)
- Pisani Marina Xavier (Universidade de São Paulo, Ribeirao Preto, N/A, Brazil)
- Davi Letícia Resende (Universidade de São Paulo, Ribeirao Preto, N/A, Brazil)
- Silva-lovato Cláudia Helena (Universidade de São Paulo, Ribeirao Preto, N/A, Brazil)
- Souza Raphael Freitas (Universidade de São Paulo, Ribeirao Preto, N/A, Brazil)
- Paranhos Helena Freitas Oliveira (Faculdade de Odontologia de Ribeirão Preto - Universidade de São Paulo, Ribeirao Preto, N/A, Brazil)

SESSION INFORMATION
Poster Session
Removable Prosthodontics
03/18/2011

Clinical efficacy of a castor bean solution as denture cleanser

Braz Dent J. 2014 Jan-Feb;25(1):43-7.

Trial of an experimental castor oil solution for cleaning dentures.

Andrade IM[1], Andrade KM[2], Pisani MX[1], Silva-Lovato CH[1], de Souza RF[1], Paranhos Hde F[1].

Author information

1 Department of Dental Materials and Prosthetics, School of Dentistry of Ribeirão Preto, University of São Paulo, Ribeirão Preto, SP, Brazil.

2 Department of Restorative Dentistry, School of Dentistry of Ribeirão Preto, University of São Paulo, Ribeirão Preto, SP, Brazil.

Abstract

Denture hygiene is essential because denture biofilm is involved in oral infections and systemic diseases. Although there are chemical agents available on the market, none of them have ideal properties and research on such products is still necessary. The aim of this study was to evaluate the efficacy of a castor bean (Ricinus communis)-based solution for removing denture biofilm, compared to two traditional products (sodium hypochlorite and alkaline peroxide). Fifty maxillary complete denture wearers were instructed to brush their dentures after meals and to immerse their dentures once a day in the following solutions: Saline (20 min; control), Polident alkaline peroxide (3 min), NaOCl (20 min) and 2% castor oil solution (20 min). Participants used each solution for a period of 7 consecutive days, according to a random sequence. After each period, the internal surfaces of maxillary complete dentures were stained with a disclosing solution (1% neutral red), photographed and the disclosed biofilm was quantified with the aid of specific software. The influence of treatments on results was verified by the Friedman test ($\alpha=0.05$). Tested solutions presented significant difference ($Fr=51.67$; $p<0.001$). Saline and NaOCl were significantly different (median: 2.0% and 0.0%) whereas Polident and castor oil presented intermediate results (median: 1.0% and 1.5%, respectively). It can be concluded that the castor oil solution tested in this study was comparable to alkaline peroxide in terms of efficiency in denture biofilm removal.

PMID: 24789291

Clinical Evaluation of a New Simplified Adhesive in Cervical Lesions

Clinical Evaluation of a New Simplified Adhesive in Cervical Lesions

Objectives: A 6-month randomized, controlled prospective study evaluated, in an intra-individual comparison, the clinical performance of two simplified etch-and-rinse adhesives in non-carious cervical lesions [NCCL]. Material and Methods: Thirty-five patients, with at least two similar sized NCCL participated in this study. After sample size calculation, 70 restorations were placed, according to one of the following groups: Adper Single Bond 2 (SB/3MESPE) and Ambar (AM/FGM). The restorations were placed incrementally using a composite resin (Opallis/FGM). The restorations were evaluated at baseline and after 6 months according to the FDI criteria (Hickel et al., J Adhes Dent 2007). The differences in the ratings of the two materials after 6 months were tested with Fisher's exact test (alfa=0.05), and the performance of the materials at baseline and after 6 months was evaluated by McNemar's test (alfa=0.05). Results: All patients attended the 6-month recall. Only two restorations (one from each material) were lost after 6 months. Twelve restorations (5 for Ambar and 7 for Adper Single Bond 2) needed a new polishing procedure after 6 months due to composite fractures at the enamel margins. Conclusions: Both adhesive systems showed acceptable clinical retention rates and fulfilled the ADA partial acceptance criteria for enamel-bonding systems in non-carious cervical lesions.

Division: IADR/AADR/CADR General Session
Meeting: 2011 IADR/AADR/CADR General Session (San Diego, California)
Location: San Diego, California
Year: 2011
Final Presentation ID: 1149

Authors

- Loguercio, Alessandro D. (Universidade Estadual de Ponta Grossa, Ponta Grossa, N/A, Brazil)
- Ferri, Letícia (Universidade Estadual de Ponta Grossa, Ponta Grossa, N/A, Brazil)
- Ferreira, Thays Regina Costa (Universidade Estadual de Ponta Grossa, Ponta Grossa, N/A, Brazil)
- Reis, Alessandra (Universidade Estadual de Ponta Grossa, Ponta Grossa, N/A, Brazil)

Loguercio, Alessandro D. (Universidade Estadual de Ponta Grossa, Ponta Grossa, N/A, Brazil)
Ferri, Letícia (Universidade Estadual de Ponta Grossa, Ponta Grossa, N/A, Brazil)
Ferreira, Thays Regina Costa (Universidade Estadual de Ponta Grossa, Ponta Grossa, N/A, Brazil)
Reis, Alessandra (Universidade Estadual de Ponta Grossa, Ponta Grossa, N/A, Brazil)

SESSION INFORMATION
Poster Session
Clinical Research: Bonding Agents and Mechanisms
03/17/2011

Clinical Evaluation of a New Simplified Adhesive in Cervical Lesions

Journal of Dentistry
Volume 43, Issue 9, September 2015, Pages 1083-1092

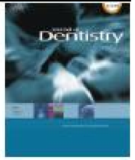

A new universal simplified adhesive: 36-Month randomized double-blind clinical trial

Alessandro D. Loguercio [a], Eloisa Andrade de Paula [b], Viviane Hass [c], Issis Luque-Martinez [d], Alessandra Reis [a], Jorge Perdigão [e]

⊞ Show more

https://doi.org/10.1016/j.jdent.2015.07.005

Get rights and content

Abstract

Statement of the problem

It is still debatable which technique should be used with universal adhesives, either etch-and-rinse (wet or dry) or self-etch strategy (with or without selective enamel etching).

Purpose of the study

To evaluate the 36-month clinical performance of Scotchbond Universal Adhesive (SU, 3M ESPE) in non-carious cervical lesions (NCCLs) using two evaluation criteria.

Methods/materials

Thirty-nine patients participated in this study. Two-hundred restorations were assigned to four groups: ER*m*: etch-and-rinse + moist dentin; ER*d*: etch-and-rinse + dry dentin; *Set*: selective enamel etching; and SE: self-etch. The same composite resin was inserted for all restorations in up to 3 increments. The restorations were evaluated at baseline and at 6-, 18-, and 36-months using both the FDI and the USPHS criteria. Statistical analyses were performed with Friedman repeated measures ANOVA by rank and McNemar test for significance in each pair ($\alpha = 0.05$).

Results

Eight restorations (ER*m*: 1; ER*d*: 1; *Set*: 1 and SE: 5) were lost after 36 months, but only significant for SE when compared with baseline ($p = 0.02$ for either criteria). Marginal staining occurred in 6.8% of the restorations (groups ER*m*, ER*d*, and *Set*) and 17.5% of the restorations (group SE), with significant difference for each group when compared with baseline using the FDI criteria ($p < 0.04$), while statistical significance was reached only for SE when compared with baseline using the USPHS criteria ($p < 0.03$). Twenty-eight and 49 restorations were scored as *bravo* for marginal adaptation using the USPHS and FDI criteria, respectively, with significant difference for each group when compared with baseline ($p < 0.05$).

Conclusions

While there was no statistical difference among bonding strategies when a universal adhesive was used, there were signs of degradation when the universal adhesive was applied in SE mode. The FDI criteria remain more sensitive than the USPHS criteria, especially for the criteria marginal staining and marginal adaptation.

Clinical Evaluation of CAD/CAM-Generated Composite Inlays: Ten-Year Report

Objective: The purpose of this study was to evaluate the clinical performance of composite and porcelain CAD/CAM-generated, adhesive inlays after ten years of clinical service.

Methods: Two clinicians randomly placed 40 porcelain (P=Vita Mark II/Vita) and 40 composite (C=Paradigm MZ100/3M-ESPE) CAD/CAM inlays in 43 patients. A CAD/CAM unit (CEREC 2/Sirona) was used to fabricate all restorations chairside in a single appointment. All inlays were cemented with a total etch technique using Single Bond (3M-ESPE) and dual cured resin cement (RelyX-ARC/3M-ESPE). Restorations were evaluated by two examiners using modified-USPHS criteria at baseline, 6 months, 1, 2, 3, 6, and 10 years.

Results: At ten years, there was no significant difference in margin finish, surface finish, anatomic form, caries, or sensitivity between the two materials with %alpha scores >93%. There was no significant difference in margin discoloration or margin adaptation between the two materials at ten years (Mann-Whitney U test, $p<0.05$). However, both materials showed a significant increase in margin discoloration (%alpha: C=79.3%, P=70.6%) compared to baseline (Wilcoxson signed-rank test (WSRT), $p<0.05$). And both materials showed a significant decrease in margin adaptation (%alpha: C=86.2%, P=70%) compared to baseline (WSRT, $p<0.05$). The composite inlays showed no significant difference in color match at any recall period, while the porcelain inlays had a significant decrease in color match at 6 months (WSRT, $p<0.05$), with no significant color change between 6 months and 10 years. Composite inlays had 5 cusp/tooth fractures and 1 inlay fracture. Porcelain inlays had 2 cusp/tooth fractures and 5 restoration fractures.

Conclusions: After 10 years success rates for the two groups were within acceptable limits (P=79.4% C=80.5%). The composite inlays performed as well as the porcelain inlays with less bulk inlay fracture. This study was supported by a grant from 3M-ESPE.

Division: IADR/AADR/CADR General Session
Meeting: 2011 IADR/AADR/CADR General Session (San Diego, California)
Location: San Diego, California
Year: 2011
Final Presentation ID: 379

Authors

- Fasbinder Dennis J. (University of Michigan, Ann Arbor, MI, USA)
- Dennison Joseph (University of Michigan, Ann Arbor, MI, USA)
- Heys Donald (University of Michigan, Ann Arbor, MI, USA)

SESSION INFORMATION
Oral Session
Keynote Address and Clinical Research: Indirect Restorations and Ceramic-based Materials
03/17/2011

Clinical Evaluation of CAD/CAM-Generated Composite Inlays: Ten-Year Report

**TITLE**	**Clinical Performance of CAD/CAM-Generated Composite Inlays After 10 Years**
**AUTHOR(S)**	Fasbinder, Dennis J.; Neiva, Gisele F.; Dennison, Joseph B.; Heys, Donald R.
**PUB. DATE**	January 2013
**SOURCE**	Journal of Cosmetic Dentistry;Winter2013, Vol. 28 Issue 4, p134
**SOURCE TYPE**	Academic Journal
**DOC. TYPE**	Article

ABSTRACT

A number of porcelain materials have demonstrated clinical reliabil[illegible] placement CAD/CAM technique (e.g., CEREC). The purpose of thi[illegible] to evaluate the longitudinal clinical performance of a composite res[illegible] (Paradigm) compared to a porcelain material (Vita Mark II) for chairside CAD/CAM-generated adhesive inlays. The inlays were evaluated at six months, one year, two years, three years, six years, and 10 years. Composite resin CAD/CAM inlays performed equally as well as porcelain CAD/CAM inlays after 10 years of clinical service, with clinical advantages noted favoring composite inlays for fracture resistance and better color match to the tooth.

READ THE ARTICLE
COURTESY OF YOUR LOCAL LI

ACCESSION # 86139689

Clinical trial of cervical composite restorations with or without bevel

Clinical trial of cervical composite restorations with or without bevel

Objectives: The aim of this double-blind randomized clinical trial was to compare the performance of cervical composite restorations, with and without bevelling the marginal surface. Methods: A total of 25 non-carious cervical lesions, comprising 12 without bevel (butt joint) and 13 with marginal bevelling were selected in 13 patients (6 male, 7 female). All cavities were restored by one clinician with a simplified adhesive system (Adper Single Bond, 3M ESPE, St. Paul, Minnesota, USA) and two resin composite systems: 1) nanofilled - Filtek Z350 (3M ESPE) and 2) microfilled - Durafill (Heraeus-Kulzer). A halogen light-curing unit (XL 3000, 3M ESPE) was used throughout the study. Restorations were polished immediately. Analysis was carried out at baseline and after six months by a calibrated evaluator (kappa), according to FDI criteria. Data were analyzed by Chi-square test, with significance level of 5%. Results: Results showed that nanofilled composite resin (Z350) had better performance than microfilled (Durafill) in relation to translucence and color stability criteria. Beveled and nonbeveled restorations performed similarly after six months in relation to fractures and retention, marginal adaptation, postoperative hypersensitivity, recurrence of caries, surface luster, and anatomic form. There was no statistically significant difference between resins in the 6-month follow-up. Conclusions: It was concluded that both composite resins tested can be used in cervical lesion restorations, regardless bevel presence. After 6-month follow-up, the nanofilled composite (Z350) showed best translucence and color stability than the microfilled system (Durafill).

Division: IADR/AADR/CADR General Session
Meeting: 2011 IADR/AADR/CADR General Session (San Diego, California)
Location: San Diego, California
Year: 2011
Final Presentation ID: 3244

Authors

- Erhardt, Maria Carolina Guilherme (Universidade Federal do Rio Grande do Sul, Porto Alegre, N/A, Brazil)
- Albarello, Janaina Lourença (Universidade Lut. Brasileira, Cachoeira do Sul, N/A, Brazil)
- Cervo, Tainã Bisognin (Universidade Lut. Brasileira, Cachoeira do Sul, N/A, Brazil)
- Conceiçao, Andréa Brito (Universidade Federal do Rio Grande do Sul, Porto Alegre, N/A, Brazil)
- Rolla, Juliana (Universidade Federal do Rio Grande do Sul, Porto Alegre, N/A, Brazil)
- Thomé, Thaís (Universidade Federal do Rio Grande do Sul, Porto Alegre, N/A, Brazil)
- Conceiçao, Ewerton Nocchi (Universidade Federal do Rio Grande do Sul, Porto Alegre, N/A, Brazil)
- Pereira, Charles Da Cunha (Universidade Lut. Brasileira, Cachoeira do Sul, N/A, Brazil)
- Coelho-de-souza, Fábio Herrmann (Universidade Federal do Rio Grande do Sul, Porto Alegre, N/A, Brazil)

Erhardt, Maria Carolina Guilherme (Universidade Federal do Rio Grande do Sul, Porto Alegre, N/A, Brazil)
Albarello, Janaina Lourença (Universidade Lut. Brasileira, Cachoeira do Sul, N/A, Brazil)
Cervo, Tainã Bisognin (Universidade Lut. Brasileira, Cachoeira do Sul, N/A, Brazil)
Conceiçao, Andréa Brito (Universidade Federal do Rio Grande do Sul, Porto Alegre, N/A, Brazil)
Rolla, Juliana (Universidade Federal do Rio Grande do Sul, Porto Alegre, N/A, Brazil)
Thomé, Thaís (Universidade Federal do Rio Grande do Sul, Porto Alegre, N/A, Brazil)
Conceiçao, Ewerton Nocchi (Universidade Federal do Rio Grande do Sul, Porto Alegre, N/A, Brazil)
Pereira, Charles Da Cunha (Universidade Lut. Brasileira, Cachoeira do Sul, N/A, Brazil)
Coelho-de-souza, Fábio Herrmann (Universidade Federal do Rio Grande do Sul, Porto Alegre, N/A, Brazil)

SESSION INFORMATION
Poster Session
Clinical Research: Direct Restorative Materials
03/19/2011

Clinical trial of cervical composite restorations with or without bevel

J Appl Oral Sci. 2012 Mar-Apr;20(2):174-9.

A randomized double-blind clinical trial of posterior composite restorations with or without bevel: 1-year follow-up.

Coelho-De-Souza FH[1], Camargo JC, Beskow T, Balestrin MD, Klein-Júnior CA, Demarco FF.

Author information

1 Department of Conservative Dentistry, Federal University of Rio Grande do Sul, Porto Alegre, RS, Brazil.

Abstract

OBJECTIVE: This randomized double-blind clinical trial compared the performance of posterior composite restorations with or without bevel, after 1-year follow-up.

MATERIAL AND METHODS: Thirteen volunteers requiring at least two posterior composite restorations were selected. Twenty-nine cavities were performed, comprising 14 without bevel (butt joint) and 15 with bevel preparation of the enamel cavosurface angle. All cavities were restored with simplified adhesive system (Adper Single Bond) and composite resin (Filtek P60). A halogen light curing unit was used through the study. Restorations were polished immediately. Analysis was carried out at baseline, after 6 months and after 1 year by a calibrated evaluator (Kappa), according to the FDI criteria. Data were statistically analyzed by Mann-Whitney test ($p < 0.05$).

RESULTS: Beveled and non-beveled cavities performed similarly after 1 year follow-up, regarding to fractures and retention, marginal adaptation, postoperative hypersensitivity, recurrence of caries, surface luster and anatomic form. However, for surface and marginal staining, beveled cavities showed significantly better performance ($p < 0.05$) than butt joint restorations.

CONCLUSIONS: It was concluded that the restorations were acceptable after 1 year, but restorations placed in cavities with marginal beveling showed less marginal staining than those placed in non-beveled cavities.

PMID: 22666833 PMCID: PMC3894759

Color Change Of Relined Dentures After Disinfection: A Clinical Trial

Objective: The purpose of this in vivo study was to evaluate the effect of chemical disinfection (Chlorexidine Digluconate 2% and Sodium Perborate) on the color stability of hard chairside reline resin after 15 days service period. **Methods:** Forty Five adult patients, who required denture reline treatment, were included in this study. Tokuyama Rebase II was used to reline complete maxillary dentures. Adaptation of each denture was examined and the surface to be relined was reduced with of a rotary cutting instrument. The relining material was prepared, poured into the relining area, inserted and adjusted after setting. The edentulous subjects were randomly divided into 3 groups (n=15), according disinfection method: CG (control group) - brushing with coconut soap and soft toothbrush; PG (Perborate group) - brushing according to previous methods and disinfection with sodium perborate (Corega Tabs) for 5 minutes once a day during the evaluation period (15 days) and ChxG (Chlorhexidine Grup) - brushing according to CG and disinfection with chlorhexidine digluconate 2% for 5 minutes once a day during the evaluation period. Color parameters in L*a*b* were recorded with a spectrophotometer. Each relined denture was subjected to baseline evaluation and after 7 and 15 days. Based on the L*a*b* values the color difference (ΔE) was calculated. Data were analyzed using Anova and Tukey tests (p=0.05). Results: The reline material showed color changes (ΔE) from 1.64 to 4.26. Analysis of variance with repeated measures on ΔE identified significant effect of main factor time (p <0.001) but not the method of disinfection (p = 0.298). **The results** demonstrated that the color change values of 7 days after disinfection were significantly lower than those of 15 days. Conclusion: The color of Tokuyama Rebase II was affected independent of the disinfection method used after 7 days observation period. **Grants: FAPESP-2010/00916-7**

Division: IADR/AADR/CADR General Session
Meeting: 2011 IADR/AADR/CADR General Session (San Diego, California)
Location: San Diego, California
Year: 2011
Final Presentation ID: 1990

Authors

- Moffa Eduardo Buozi (Universidade Est. Paulista Julio Mesquita, Araraquara - SP, N/A, Brazil)
- Ribeiro Roberta (Universidade Est. Paulista Julio Mesquita, São Carlos, N/A, Brazil)
- Fernanda Izumida (UNESP, Araraquara- SP, N/A, Brazil)
- Ana Claudia Pavarina (UNESP, Araraquara- SP, N/A, Brazil)
- Eunice Giampaolo (UNESP, Araraquara- SP, N/A, Brazil)
- Vergani Carlos E. (UNESP, Araraquara- SP, N/A, Brazil)

SESSION INFORMATION
Poster Session
Prosthodontics
03/18/2011

Color Change Of Relined Dentures After Disinfection: A Clinical Trial

ELSEVIER

Journal of Dentistry

Volume 39, Supplement 3, December 2011, Pages e65-e71

Colour stability of relined dentures after chemical disinfection. A randomised clinical trial

Eduardo Buozi Moffa, Eunice Teresinha Giampaolo, Fernanda Emiko Izumida, Ana Cláudia Pavarina, Ana Lúcia Machado, Carlos Eduardo Vergani

Department of Dental Materials and Prosthodontics, Araraquara Dental School, UNESP – Univ. Estadual Paulista, Rua Humaita, 1680 CEP: 14801-903, Araraquara, São Paulo, Brazil

Received 1 March 2011, Revised 30 September 2011, Accepted 17 October 2011, Available online 22 October 2011.

Abstract

Background

This randomised clinical study evaluated the effect of chemical disinfection with sodium perborate or chlorhexidine on the colour stability of a hard chairside reline resin during six months.

Methods

Hard chairside reline resin (Tokuyama Rebase Fast II) was used to reline complete dentures. After baseline colour measurements, the patients were randomly divided into 3 groups (n = 15) and allocation was concealed with the use of the BioStat program. The dentures were cleansed according to three methods: CG (control group) – brushing with coconut soap and soft toothbrush, PG (Perborate group) – brushing according to previous methods and disinfection with warmed sodium perborate solution (Corega Tabs) for 5 min, once a day for 6 months and ChxG (Chlorhexidine Group) – brushing according to CG and disinfection with chlorhexidine digluconate 2% for 5 min once a day for 6 months. The data of ΔE^*, ΔL^*, Δa^* and Δb^* were analysed by 2-way repeated-measures ANOVAs and Tukey tests (α = 0.05).

Results

There were significant differences amongst groups for ΔL, Δa and Δb. The time had a significant effect on ΔE and ΔL, for all groups evaluated.

Conclusion

Changes in some colour parameters (ΔL, Δa and Δb) of the reline resin Tokuyama Rebase were observed when the dentures were disinfected by perborate and chlorhexidine digluconate 2% solutions. The colour stability of was also influenced by time, regardless of disinfection or nondisinfection.

Clinical implications

Colour stability of the denture materials is one variable to be considered when choosing disinfection methods. The data in this study will be useful to clinicians when they are selecting disinfectant solutions for disinfection of relined denture.

Conversion of Easy-Bond into a two-step self-etch adhesive: 18-months results

Conversion of Easy-Bond into a two-step self-etch adhesive: 18-months results

Objectives: The purpose of this randomized clinical trial was to evaluate the clinical performance of an one-step self-etch adhesive in non-carious cervical lesions with and without the application of an additional hydrophobic resin coat. Methods: Seventeen patients with non-carious cervical lesions received two or four restorations after being randomly assigned to two adhesive technique protocols: 1) a one-step self-etch adhesive (Adper Easy Bond, 3M ESPE) was applied following manufacturerxs instructions; 2) application of Adper Easy Bond, immediately followed by the application of a hydrophobic resin coat (Scotchbond Multipurpose Bonding Agent, 3M ESPE). All restorations were restored with a hybrid composite (Filtek Z250, 3M ESPE) and cured with a light-curing unit operating at 1200 mW/cm2 (n=32). Clinical effectiveness was recorded in terms of retention, marginal discoloration, marginal integrity, post-operative sensitivity, recurrent caries, periodontal health and pulp vitality. The data were analyzed using Chi-square and Fisher's exact tests at p<0.05. Results: Two restorations of each group were lost after 6 months leading to an overall clinical success rate of 93.8% for both groups. At 18-months evaluation period, no new restoration was lost, but the occurrence of caries at the dentin margin of one restoration of EB group decreased the overall success rate to 90.6% in comparison to 93.8% of EB+B, although this difference was not statistically significant (p=0.500). All lost restorations were placed in lower premolars presenting wear facet and dentin with moderate dentin sclerosis. Conclusion: The application of a hydrophobic resin coat over Easy Bond did not increase bonding effectiveness significantly in non-carious cervical lesions after 18 months.

Division: IADR/AADR/CADR General Session
Meeting: 2011 IADR/AADR/CADR General Session (San Diego, California)
Location: San Diego, California
Year: 2011
Final Presentation ID: 1147

Authors

- Sartori, Neimar (Federal University of Santa Catarina and Case Western Reserve University, Florianopolis/SC, N/A, Brazil)
- Coutinho Guimaraes, Jackeline (Universidade Federal do Espirito Santo, Vitória, N/A, Brazil)
- Dalmagro Peruchi, Lais (Case Western Reserve University, Cleveland, OH, USA)
- Baratieri, Luiz N. (Universidade Federal De Santa Catarina, Florianopolis SC, N/A, Brazil)
- Batalha-silva, Silvana (Universidade Federal De Santa Catarina, Florianópolis, N/A, Brazil)
- Monteiro, Sylvio (Universidade Federal De Santa Catarina, Florianopolis, N/A, Brazil)
- Belli, Renan (University of Erlangen-Nuremberg, Erlangen, N/A, Germany)

Sartori, Neimar (Federal University of Santa Catarina and Case Western Reserve University, Florianopolis/SC, N/A, Brazil)
Coutinho Guimaraes, Jackeline (Universidade Federal do Espirito Santo, Vitória, N/A, Brazil)
Dalmagro Peruchi, Lais (Case Western Reserve University, Cleveland, OH, USA)
Baratieri, Luiz N. (Universidade Federal De Santa Catarina, Florianopolis SC, N/A, Brazil)
Batalha-silva, Silvana (Universidade Federal De Santa Catarina, Florianópolis, N/A, Brazil)
Monteiro, Sylvio (Universidade Federal De Santa Catarina, Florianopolis, N/A, Brazil)
Belli, Renan (University of Erlangen-Nuremberg, Erlangen, N/A, Germany)

SESSION INFORMATION
Poster Session
Clinical Research: Bonding Agents and Mechanisms
03/17/2011

[illegible]ths results

OPERATIVE DENTISTRY

Volume 38, Issue 3 (May/June 2013)

SUMMARY

The purpose of this randomized clinical trial was to evaluate the clinical performance of a one-step self-etch adhesive in noncarious cervical lesions with inclusion of a hydrophobic bonding layer not included in the original bonding system as a test of potentially improved bonding. Patients with noncarious cervical lesions received two or four restorations after being randomly assigned to two adhesive technique protocols (n=32): EB, application of Adper Easy Bond (3M ESPE) following manufacturer's instructions; and EB+B, application of Adper Easy Bond, immediately followed by the application of a hydrophobic resin coat (Scotchbond Multi-Purpose Bonding Agent, 3M ESPE). All restorations were restored with a microhybrid composite (Filtek Z250, 3M ESPE). Clinical effectiveness was recorded in terms of retention, marginal discoloration, marginal integrity, postoperative sensitivity, recurrent caries, periodontal health, and pulpal vitality, according to the modified USPHS criteria, for 18 months. Data were analyzed using chi-square, Fisher exact, and McNemar tests at $\alpha=0.05$. Two restorations of each group were debonded after six months, leading to an overall clinical success rate of 93.8% for both groups. At the 18-month evaluation period, no new restoration was debonded. However, one restoration of the EB group displayed recurrent caries at the dentin margin, decreasing the overall success rate to 90.6% in comparison to 93.8% of EB+B. The success rate between EB and EB+B was not statistically significant ($p=0.5$). The application of a hydrophobic resin coat over EB did not increase bonding effectiveness in noncarious cervical lesions after 18 months.

Cost Effectiveness of Tooth Replacement Strategies for Partially Dentate Elders

Objectives: This study aimed to compare the cost effectiveness of conventional treatment using partial dentures with functionally-orientated treatment based on the shortened dental arch concept to replace missing teeth for partially dentate elders.
Methods: 44 partially dentate patients aged 65 years and older were recruited following routine dental assessment at a university dental hospital. Patients consented to and were randomly assigned to the two treatment arms. The conventional treatment group received a removable partial denture to replace all missing natural teeth. The functionally-orientated group were restored to a shortened dental arch of 10 occluding contacts using resin bonded bridgework. The costs associated with each treatment were recorded including laboratory charges, treatment time and opportunity costs. The impact on quality of life (OHRQoL) was measured using the 14-item Oral Health Impact Profile.

Results: Both groups reported improvements in OHRQoL after completion of treatment. For the conventional group, the mean OHIP-14 score decreased from 12.4 pre-operatively to 3.3 post-operatively ($p<0.001$). In the functionally-orientated group the OHIP-14 score decreased from 11.4 to 1.8 following treatment ($p<0.001$). On average the conventional treatment group required 8.3 clinic visits as compared to 4.4 visits for the functionally-orientated group. The mean total treatment time was 183 minutes 19 seconds for the conventional group versus 124 minutes 8 seconds for the functionally-orientated group. The conventional treatment group had an average of 6.33 teeth replaced at a laboratory cost of 337.31 Euros. The functionally-orientated group had an average of 2.64 teeth replaced at a laboratory cost of 244.05 Euros.

Conclusions: Restoration to a shortened dental arch using functionally-orientated treatment resulted in a similar improvement in OHRQoL with fewer clinic visits, less operative time and at a lower laboratory cost compared with conventional treatment.

Division: IADR/AADR/CADR General Session
Meeting: 2011 IADR/AADR/CADR General Session (San Diego, California)
Location: San Diego, California
Year: 2011
Final Presentation ID: 2913

Authors

- Mckenna Gerald John (University College Cork, Cork, N/A, Ireland)
- Allen Patrick Finbarr (National University of Ireland - Cork, Cork, N/A, Ireland)
- Woods Noel (National University of Ireland, Cork, N/A, Ireland)

SESSION INFORMATION
Oral Session
Elders, Medically Compromised, and Orthodontics
03/19/2011

Cost Effectiveness of Tooth Replacement Strategies for Partially Dentate Elders

Community Dentistry and Oral Epidemiology

Original Article

Cost-effectiveness of tooth replacement strategies for partially dentate elderly: a randomized controlled clinical trial

Gerald McKenna, Finbarr Allen, Noel Woods, Denis O'Mahony, Michael Cronin, Cristiane DaMata, Charles Normand

First published: 20 November 2013 | https://doi.org/10.1111/cdoe.12085 | Cited by: 13

Abstract

Objective

To conduct a cost-effectiveness analysis comparing two different tooth replacement strategies for partially dentate older patients, namely partial removable dental prostheses (RDP) and functionally orientated treatment based on the shortened dental arch concept (SDA).

Methods

Ninety-two partially dentate older patients completed a randomized controlled clinical trial. Patients were randomly allocated to two treatment groups: the RDP group and the SDA group. Treatment effect was measured using impact on oral health–related quality of life (OHrQOL), and the costs involved in providing and maintaining care were recorded for all patients. Patients were followed for 12 months after treatment intervention. All treatment was provided by a single operator.

Results

The total cost of achieving the minimally important clinical difference (MID) in OHrQOL for an average patient in the RDP group was €464.64. For the SDA group, the cost of achieving the MID for an average patient was €252.00. The cost-effectiveness ratio was therefore 1:1.84 in favour of SDA treatment.

Conclusion

With an increasingly ageing population, many patients will continue to benefit from removable prostheses to replace their missing natural teeth. From a purely economic standpoint, the results from this analysis suggest that the treatment of partially dentate older adults should be focused on functionally orientated treatment because it is simply more cost-effective.

Denture microwave disinfection in treating diabetics with denture stomatitis

Denture microwave disinfection in treating diabetics with denture stomatitis

Objectives: this randomized study investigated the efficacy of denture microwave disinfection compared with that of topical antifungal medication in resolving the clinical signs of denture stomatitis (DS) of diabetics. Methods: forty denture wearers well-controlled type 2 diabetics with DS were randomly assigned into two groups (n=20). NYS: patients were treated with Nystatin oral suspension (100.000UI/mL) 4x/daily for 14-days; MW: patients had their dentures immersed in water and microwaved (650W/3min) 3x/weekly for 14-days. Patients were followed up for 3 months. Standardized photographs of the palates of patients were taken at baseline, the end of treatments, and follow up (day-30, day-60, day-90). Two independent observers were engaged to blind-analyze the five photographs from each patient and classify the mucosal characteristics according to Newton's criteria (0, I, II, III). The degrees of correlation and concordance inter-observers were estimated (Kappa and Kendall's rank tests, and coefficient of concordance) and the homogeneity of the mucosal characteristics of patients between the groups at baseline was verified (Fisher's exact test). To analyze the treatment's effect on the evolution of DS over the time, a categorical variable was created, the percentage of patients in each category was determined for each period, and comparisons between the groups were performed (Mann-Whitney test). The percentages of patients cured and with recurrence were compared between the groups (Fisher's exact test). Differences were considered significant at $p<0.05$. Results: at baseline, there was homogeneity between the two groups for the mucosal characteristics of patients ($p=0.6514$). There were no significant differences ($p>0.05$) between the clinical efficacy of NYS and MW treatments in the evolution of DS over the time and in the percentage of patients cured and with recurrence. Conclusion: microwaving dentures is as effective as Nystatin in resolving the clinical signs of DS of diabetic patients. GRANT: FAPESP 2006/02842-5, 2007/03895-8; CNPq 470337/2007-9.

Division: IADR/AADR/CADR General Session
Meeting: 2011 IADR/AADR/CADR General Session (San Diego, California)
Location: San Diego, California
Year: 2011
Final Presentation ID: 2416

Authors

- Sanitá, Paula Volpato (Araraquara Dental School, UNESP- Univ Estadual Paulista, Araraquara, N/A, Brazil)
- Machado, A.l. (Araraquara Dental School, UNESP- Univ Estadual Paulista, Araraquara, N/A, Brazil)
- Dovigo, L.n. (Araraquara Dental School, UNESP- Univ Estadual Paulista, Araraquara, N/A, Brazil)
- Giampaolo, E.t. (Araraquara Dental School, UNESP- Univ Estadual Paulista, Araraquara, N/A, Brazil)
- Pavarina, A.c. (Araraquara Dental School, UNESP- Univ Estadual Paulista, Araraquara, N/A, Brazil)
- Vergani, C.e. (Araraquara Dental School, UNESP- Univ Estadual Paulista, Araraquara, N/A, Brazil)

Sanitá, Paula Volpato (Araraquara Dental School, UNESP- Univ Estadual Paulista, Araraquara, N/A, Brazil)
Machado, A.l. (Araraquara Dental School, UNESP- Univ Estadual Paulista, Araraquara, N/A, Brazil)
Dovigo, L.n. (Araraquara Dental School, UNESP- Univ Estadual Paulista, Araraquara, N/A, Brazil)
Giampaolo, E.t. (Araraquara Dental School, UNESP- Univ Estadual Paulista, Araraquara, N/A, Brazil)
Pavarina, A.c. (Araraquara Dental School, UNESP- Univ Estadual Paulista, Araraquara, N/A, Brazil)
Vergani, C.e. (Araraquara Dental School, UNESP- Univ Estadual Paulista, Araraquara, N/A, Brazil)

SESSION INFORMATION
Poster Session
Removable Prosthodontics
03/18/2011

Denture microwave disinfection in treating diabetics with denture stomatitis

Int J Prosthodont. 2012 May-Jun;25(3):232-44.

Microwave denture disinfection versus nystatin in treating patients with well-controlled type 2 diabetes and denture stomatitis: a randomized clinical trial.

Sanita PV[1], Machado AL, Pavarina AC, Massucato EM, Colombo AL, Vergani CE.

Author information

1 Univ Estadual Paulista, Sao Paulo, Brazil.

Abstract

PURPOSE: The aim of this randomized clinical trial was to compare the effectiveness of microwave denture disinfection and nystatin in the treatment of well-controlled type 2 diabetic patients with denture stomatitis in terms of microbiologic and clinical outcomes.

MATERIALS AND METHOD: Diabetic patients wearing maxillary complete dentures with denture stomatitis ($n = 40$) were divided into two groups: NYS (patients treated with topical nystatin 4 times/day for 14 days) and MW (patients who had their dentures microwaved [650 W for 3 minutes] 3 times/week for 14 days). Mycologic samples were taken from the palates and dentures of the patients for quantification and identification of Candida, and standardized photographs of the palates were taken for clinical analysis. Evaluations were repeated at baseline, the end of treatment (day 14), and throughout follow-up (days 30, 60, and 90). Microbiologic data were evaluated by analysis of variance using a random effects statistical model, Tukey post hoc test, and chi-square test ($\alpha = .05$). Clinical results were analyzed using Mann-Whitney and Fisher exact tests ($\alpha = .05$).

RESULTS: Both treatments were considered successful in reducing the clinical signs of denture stomatitis and significantly reduced the values of colony-forming units/mL from the palates and dentures at days 14 and 30. In addition, 40% of treated patients were cured by the end of treatment. No significant differences in the microbiologic and clinical outcomes were revealed between the two groups ($P > .05$). C albicans was the most predominant species isolated ($P < .01$), followed by C tropicalis and C glabrata.

CONCLUSION: Denture microwave disinfection was as effective as nystatin for the treatment of diabetic patients with denture stomatitis.

PMID: 22545252

Early Loading Maxillary Cresco Full Arch Reconstructions. An RCT trial

Objectives: Appraise the feasibility of interchanging conventional components of a fixed dental prosthesis (FDP) with those of Cresco in two different early loading protocols. Methods: In five centers located in Norway and Sweden patients with an edentulous, fully healed maxilla were recruited to partake in a three-arm blinded randomized controlled trial (RCT). Each patient received 5/6 implants to support a 10/12-unit FDP. The implants used were SLA solid screw two-part implants (Institut Straumann AG, Basel, Switzerland). In test groups 1 and 2 components from Cresco (Cresco Ti Systems, Sarl, Lausanne, Switzerland) were used and the implants loaded 10 days or 6-8 weeks post-implant placement. Group 3 received their FDP fabricated with conventional components 6-8 weeks post-implant placement. Patients were followed up 3 years. Results: Of 36 patients 30 remained after 3 years. The adjusted means and ranges of changes in crestal bone levels were -0.65 mm, -0.5 mm and -0.40 mm in groups 1,2 and 3 respectively. The change from baseline was statistically significant in all treatment groups. Adjusting for the difference in implant depth, there was an expected additional change in bone level of -0.29 mm by each 1 mm the implant was placed deeper. There was no significant difference between the 6-8 weeks post-implant placement loading Cresco group versus and the control group or between the two Cresco groups. Conclusion: The vertical placement has more effect on bone loss overthan the fabrication technique used for the suprastructure and whether the implants were loaded after 10 days versus 6 to 8 weeks. Supported by Institut Straumann, Switzerland.

Division: IADR/AADR/CADR General Session
Meeting: 2011 IADR/AADR/CADR General Session (San Diego, California)
Location: San Diego, California
Year: 2011
Final Presentation ID: 1765

Authors

- Jokstad Asbjorn (University of Toronto Faculty of Dentistry, Toronto, ON, Canada)
- Ellner Stefan (Specialist Dental Care Center, Kalmar County, Sweden, Kalmar, N/A, Sweden)
- Gussgard Anne Margrete (University of Toronto, Toronto, ON, Canada)

SESSION INFORMATION
Oral Session
Keynote Address and Topics in Prosthodontic Research
03/18/2011

Early Loading Maxillary Cresco Full Arch Reconstructions. An RCT trial

CLINICAL ORAL IMPLANTS RESEARCH WILEY

Comparison of two early loading protocols in full arch reconstructions in the edentulous maxilla using the Cresco prosthetic system: a three-arm parallel group randomized-controlled trial

Asbjorn Jokstad, Stefan Ellner, Anne Gussgard

First published:24 March 2011 | https://doi.org/10.1111/j.1600-0501.2010.02156.x | Cited by: 6

Corresponding author:
Asbjorn Jokstad
Prosthodontics Faculty of Dentistry
University of Toronto
124 Edward Street
Toronto
ON Canada M5G 1G6
Tel.: +1 416 979 4930 ext 4427
Fax: +1 416 979 4769
e-mail: a.jokstad@dentistry.utoronto.ca

Abstract

Objectives: Appraise the feasibility of interchanging conventional components of a fixed dental prosthesis (FDP) with those of Cresco in two different early loading protocols.

Material and methods: In five centers patients with an edentulous, fully healed maxilla were recruited to partake in a three-arm blinded randomized-controlled trial (RCT). Each patient received 5/6 implants using a single-stage surgery approach to support a 10/12-unit FDP. The implants used were SLA solid screw two-part implants. In test groups 1 and 2 components from Cresco were used and the implants loaded 10 days or 6–8 weeks post-implant placement. Group 3 received their FDP fabricated with conventional components 6–8 weeks post-implant placement. Patients were followed up 3 years.

Results: Of 36 patients, 30 remained after 3 years. The adjusted means and ranges of changes in crestal bone levels were −0.65, −0.5 and −0.4 mm in groups 1, 2 and 3, respectively. The change from baseline was statistically significant in all treatment groups. Adjusting for the difference in implant depth, there was an expected additional change in bone level of −0.29 mm by each 1 mm the implant was placed deeper. There was no significant difference between the 6-8 weeks post-implant placement loading Cresco group vs. the control group or between the two Cresco groups.

Conclusions: The vertical placement has more effect on bone loss than the fabrication technique used for the suprastructure and whether the implants were loaded after 10 days vs. 6–8 weeks.

To cite this article: Jokstad A, Ellner S, Gussgard A. Comparison of two early loading protocols in full arch reconstructions in the edentulous maxilla using the Cresco prosthetic system: a three-arm parallel group randomized-controlled trial. *Clin. Oral Impl. Res.* **22**, 2011; 455–463. doi: 10.1111/j.1600-0501.2010.02156.x

No Influence of Platform-Switching on Crestal Bone Level Alterations

Introduction: The concept of platform switching has been introduced to implant dentistry based on clinical observations of reduced periimplant crestal bone loss. However, there is a lack of information from randomised clinical trials, and published available data are controversial.
Objective: This study aimed to test the hypothesis of no influence of platform switching on crestal bone level changes.

Materials and methods: In a split-mouth design two implants with a diameter of 4 mm were inserted epicrestally in the posterior mandible of 25 subjects (baseline). After three months of submerged healing, single-tooth crowns were placed, either with (3.3mm platform, test) or without platform switching (4mm platform, control). Patients were followed up at short intervals for monitoring of healing and for oral hygiene control. Standardised radiographs (baseline, 3, 4, 12, 25, and 38 months after implant insertion) were independently evaluated by three calibrated examiners. Statistical analysis for the influence of time and implant type on bone levels were performed with the Brunner-Langer Model and equivalence testing for bone level alterations at test and control implants using a two-sided Wilcoxon signed rank test with an equivalence range of [-0.4mm; +0.4mm].

Results: Three years after implant insertion, the mean radiographic periimplant bone loss at the test implants was 0.69 ±0.43 mm and at the control implant 0.74 ±0.57 mm. The mean difference between the two treatment modalities was 0.05 ±0.58 mm (95%CI: -0.19; 0.29). Crestal bone level alteration depended on time ($p<0.001$) but not on implant type ($p=0.363$) or on the interaction of time and implant type ($p=0.953$). The bone level alterations at test and control implants were equivalent (all $p \leqslant 0.008$).

Conclusion: The present randomised clinical trial could not confirm the hypothesis of a reduced periimplant crestal bone loss, when implants had been restored according to the concept of platform switching.

Division: IADR/AADR/CADR General Session
Meeting: 2011 IADR/AADR/CADR General Session (San Diego, California)
Location: San Diego, California
Year: 2011
Final Presentation ID: 280

Authors

- Enkling Norbert (University of Bern, Bern, N/A, Switzerland)
- Joehren Peter (University of Witten /Herdecke, Bochum, N/A, Germany)
- Mericske-stern Regina (University of Bern, Bern, N/A, Switzerland)
- Bayer Stefan (University of Bonn, Bonn, N/A, Germany)
- Stark Helmut (University of Bonn, Bonn, N/A, Germany)
- Klimberg Victoria (University of Bern, Bern, N/A, Switzerland)
- Jervøe-storm Pia-merete (University of Bonn, Bonn, N/A, Germany)
- Jepsen Soren (University of Bonn, Bonn, N/A, Germany)

SESSION INFORMATION
Oral Session
Biomaterials and Surgical Modalities for Alveolar Bone Regeneration
03/17/2011

No Influence of Platform-Switching on Crestal Bone Level Alterations

Journal of Dental Research
IADR International Association for Dental Research
AADR® American Association for Dental Research

Journal Home | Browse Journal | Submit Paper | About | Subscribe | Searc
Close
d PDF

Influence of Platform Switching on Bone-level Alterations

A Three-year Randomized Clinical Trial

N. Enkling*, P. Jöhren, J. Katsoulis, S. Bayer, P.-M. Jervøe-Storm, R. Mericske-Stern, S. Jepsen

Show le

First Published October 24, 2013 | Research Article | Check for updates
https://doi.org/10.1177/0022034513504953

Abstract

The concept of platform switching has been introduced to implant dentistry based on clinical observations of reduced peri-implant crestal bone loss. However, published data are controversial, and most studies are limited to 12 months. The aim of the present randomized clinical trial was to test the hypothesis that platform switching has a positive impact on crestal bone-level changes after 3 years. Two implants with a diameter of 4 mm were inserted crestally in the posterior mandible of 25 patients. The intraindividual allocation of platform switching (3.3-mm platform) and the standard implant (4-mm platform) was randomized. After 3 months of submerged healing, single-tooth crowns were cemented. Patients were followed up at short intervals for monitoring of healing and oral hygiene. Statistical analysis for the influence of time and platform type on bone levels employed the Brunner-Langer model. At 3 years, the mean radiographic peri-implant bone loss was 0.69 ± 0.43 mm (platform switching) and 0.74 ± 0.57 mm (standard platform). The mean intraindividual difference was 0.05 ± 0.58 mm (95% confidence interval: –0.19, 0.29). Crestal bone-level alteration depended on time ($p < .001$) but not on platform type ($p = .363$). The present randomized clinical trial could not confirm the hypothesis of a reduced peri-implant crestal bone loss, when implants had been restored according to the concept of platform switching (ClinicalTrials.gov NCT01917305).

Keywords

dental implant-abutment connection, dental implant-abutment designs, dental implant-abutment interface, dental implant, single-tooth dental implant, alveolar bone loss

2012

24-Month Clinical Performance of a Glass-Ionomer Restorative System

Objective: The aim of this study was to assess the 24-month clinical performance of a glass-ionomer restorative system used for the restoration of Class II cavities.
Method: Twenty six patients with at least 2 but not more than 4 Class II lesions were included in this study. A total of 60 lesions were randomly divided into two groups according to the restorative systems used (n=30). The lesions in Group 1, were restored with a glass-ionomer restorative system (EQUIA/ GC) which was a combination of a packable glass-ionomer (Fuji IX GP EXTRA/ GC) and a self-adhesive nano-filled coating (G-Coat PLUS GC); whereas the lesions in Group 2 were restored with a micro-filled composite (Gradia Direct/ GC) in combination with a self-etch adhesive (G-Bond/ GC) by two calibrated operators according to the manufacturers' instructions. Two independent examiners evaluated the restorations at baseline, 6-12-18 and 24 months according to the modified USPHS criteria. The differences between two groups were statistically evaluated by Pearson Chi-Square test (p=0. 05).

Result: After 24 months, 53 restorations were evaluated in 23 patients with a recall rate of 88.3%. All the restorations in the two groups were scored as Alpha for retention rate, anatomic form, recurrent caries, surface texture, postoperative sensitivity and color match. For marginal adaptation, 4 restorations (15.4%) in Group 1 and 8 restorations (29.6%) in Group 2 were scored as Bravo. Two restorations (7.6%) in Group 1 and 5 restorations (18.5%) in Group 2 were also scored as Bravo for marginal discoloration. However, the differences in terms of marginal adaptation and marginal discoloration were not statistically significant at the end of 24 months (p>0, 05).

Conclusion: The use of both materials for the restoration of Class II cavities exhibited a similar and clinically acceptable performance after 24-months.

Division: IADR/LAR General Session
Meeting: 2012 IADR/LAR General Session (Iguaçu Falls, Brazil)
Location: Iguaçu Falls, Brazil
Year: 2012
Final Presentation ID: 107

Authors

- Gurgan, Sevil (Hacettepe University, Ankara, N/A, Turkey)
- Yalcin Cakir, Filiz (Hacettepe University, Ankara, N/A, Turkey)
- Firat, Esra (Hacettepe University, Ankara, N/A, Turkey)
- Kutuk, Zeynep (Hacettepe University, Faculty of Dentistry, Ankara, N/A, Turkey)
- Ak Oztas, Sema Seval (Hacettepe University Facuty of Dentistry, Ankara, N/A, Turkey)
- Korkmaz Ceylan, Yonca (University of Texas - Houston/Health Science Center, Houston, TX, USA)

SESSION INFORMATION
Oral Session
Glass-ionomer Restorations
06/20/2012

Gurgan, Sevil (Hacettepe University, Ankara, N/A, Turkey)
Yalcin Cakir, Filiz (Hacettepe University, Ankara, N/A, Turkey)
Firat, Esra (Hacettepe University, Ankara, N/A, Turkey)
Kutuk, Zeynep (Hacettepe University, Faculty of Dentistry, Ankara, N/A, Turkey)
Ak Oztas, Sema Seval (Hacettepe University Facuty of Dentistry, Ankara, N/A, Turkey)
Korkmaz Ceylan, Yonca (University of Texas - Houston/Health Science Center, Houston, TX, USA)

24-Month Clinical Performance of a Glass-Ionomer Restorative System

36-month clinical performance evaluation of a current glass-ionomer restorative system

Esra Ergin, DDS,[a] Sevil Gürgan, DDS, PhD,[a] Zeynep Bilge Kütük, DDS,[a] Filiz Yalçın Çakır, DDS, PhD,[a] Sema Seval Öztaş, DDS[a]

[a]*Hacettepe University, Faculty of Dentistry, Department of Restorative Dentistry, Ankara, Turkey*

A B S T R A C T

Objectives: To evaluate the 36 month clinical performance of a current glass-ionomer restorative system by comparing with a micro-filled resin composite, on Class II cavities.

Materials and Methods: Sixty cavities in 26 patients were randomly divided into two groups according to the restorative systems used (n=30); the cavities in Group 1 were restored with a glass-ionomer restorative system (EQUIA/GC); packable glass-ionomer (Fuji IX GP EXTRA/GC)+self-adhesive nano-filled coating (G-Coat PLUS/GC); whereas the ones in Group 2 were restored with a micro-filled composite (Gradia Direct/GC)+a self-etch adhesive (G-Bond/GC). The restorations were evaluated at 1 week (baseline), 6, 12, 18, 24 and 36 months according to the modified USPHS criteria. The data were statistically evaluated by Pearson Chi-Square test (p=0.05).

Results: Fifty-three restorations were evaluated in 23 patients after 36 months. All the restorations in the two groups were scored as Alpha for recurrent caries, surface texture, postoperative sensitivity and color match. For marginal adaptation, 6 restorations (23.7%) in Group 1 and 8 restorations (29.6%) in Group 2 were scored as Bravo. Two restorations (7.6%) in Group 1 and 5 restorations (18.5%) in Group 2 were scored as Bravo for marginal discoloration. One restoration (3.8%) in Group 1 was scored as Charlie for anatomic form and retention because of marginal fracture within restorative material. However, there were no significant differences between the clinical performances of the materials (p>0.05).

Conclusions: Both materials exhibited a similar and clinically acceptable performance on moderate Class II cavities after 36-months.

Cumhuriyet Dental Journal

Volume 17 Issue 3

doi:10.7126/cdj.58140.1008002560

available at http://dergipark.ulakbim.gov.tr/cumudj/

A New Universal Simplified Adhesive: 6-Month Clinical Evaluation

A New Universal Simplified Adhesive: 6-Month Clinical Evaluation

Objective: A 6-month randomized, controlled prospective study evaluated the clinical performance of Scotchbond Universal Adhesive (3M ESPE) in non-carious cervical lesions (NCCL).

Method: Thirty-nine patients, with at least four similar sized NCCL participated in this study. After sample size calculation, 200 restorations were placed, according to one of the following groups: TE-Dry: total-etch + dry dentin; TE-Moist: total-etch + moist dentin; SE: self-etch; SE-En: selective enamel etching + self-etch. The restorations were placed incrementally using a composite resin (Filtek Supreme Ultra/3M ESPE). The restorations were evaluated at baseline and after 6 months according to both the FDI criteria (Hickel et al., J Adhes Dent 2007) and the classical USPHS criteria. Statistical analyses were performed with Friedman repeated measures analysis of variance by rank and Wilcoxon sign-ranked test for significance in each pair (alfa=0.05).

Result: Only 4 restorations (SE: 3 and TE-Moist: 1) were lost after 6 months (not statistically significant for either criteria). Marginal discoloration occurred in one restoration in SE group (not statistically significant for either criteria). The percentage of *bravo* scores for marginal adaptation at 6 months were 36%, 32%, 46% and 42% for TE-Dry, TE-Moist, SE and SE-En groups, respectively, for FDI criteria (not statistically significant). However, 194/200 restorations were scored as *alfa* for marginal adaptation using the USPHS criteria (one *bravo* for SE and one *bravo* for SE-En, not statistically significant).

Conclusion: All groups showed acceptable clinical retention rates and fulfilled the ADA partial acceptance criteria for dental bonding systems in NCCL. FDI criteria were more sensitive for identifying differences in adhesive restoration of NCCL.

Division: IADR/LAR General Session
Meeting: 2012 IADR/LAR General Session (Iguaçu Falls, Brazil)
Location: Iguaçu Falls, Brazil
Year: 2012
Final Presentation ID: 2011

Authors

- Kose, Carlos (Universidade Estadual De Ponta Grossa, Ponta Grossa, N/A, Brazil)
- De Paula, Eloisa (Universidade Estadual de Ponta Grossa, Ponta Grossa, N/A, Brazil)
- Tay, Lidia Yileng (Universidade Estadual De Ponta Grossa, Ponta Grossa, N/A, Brazil)
- Mena-serrano, Alexandra (Universidade Estadual De Ponta Grossa, Ponta Grossa, N/A, Brazil)
- Reis, Alessandra (Universidade Estadual De Ponta Grossa, Ponta Grossa, N/A, Brazil)
- Perdigao, Jorge (University of Minnesota, Minneapolis, MN, USA)
- Loguercio, Alessandro (Universidade Estadual De Ponta Grossa, Ponta Grossa, N/A, Brazil)

Kose, Carlos (Universidade Estadual De Ponta Grossa, Ponta Grossa, N/A, Brazil)
De Paula, Eloisa (Universidade Estadual de Ponta Grossa, Ponta Grossa, N/A, Brazil)
Tay, Lidia Yileng (Universidade Estadual De Ponta Grossa, Ponta Grossa, N/A, Brazil)
Mena-serrano, Alexandra (Universidade Estadual De Ponta Grossa, Ponta Grossa, N/A, Brazil)
Reis, Alessandra (Universidade Estadual De Ponta Grossa, Ponta Grossa, N/A, Brazil)
Perdigao, Jorge (University of Minnesota, Minneapolis, MN, USA)
Loguercio, Alessandro (Universidade Estadual De Ponta Grossa, Ponta Grossa, N/A, Brazil)

SESSION INFORMATION

Poster Session

Resin-Based Restorative Materials II

06/22/2012

A New Universal Simplified Adhesive: 6-Month Clinical Evaluation

Oper Dent. 2014 Mar-Apr;39(2):113-27. doi: 10.2341/13-045-C. Epub 2013 Jun 26.

A new universal simplified adhesive: 18-month clinical evaluation.

Perdigão J, Kose C, Mena-Serrano AP, De Paula EA, Tay LY, Reis A, Loguercio AD.

Abstract

PURPOSE: To evaluate the 18-month clinical performance of a multimode adhesive (Scotchbond Universal Adhesive, SU, 3M ESPE, St Paul, MN, USA) in noncarious cervical lesions (NCCLs) using two evaluation criteria.

MATERIALS AND METHODS: Thirty-nine patients participated in this study. Two-hundred restorations were assigned to four groups: ERm, etch-and-rinse + moist dentin; ERd, etch-and-rinse + dry dentin; Set, selective enamel etching; and SE, self-etch. The composite resin, Filtek Supreme Ultra (3M ESPE), was placed incrementally. The restorations were evaluated at baseline, and at 18 months, using both the World Dental Federation (FDI) and the United States Public Health Service (USPHS) criteria. Statistical analyses were performed using Friedman repeated-measures analysis of variance by rank and McNemar test for significance in each pair (α=0.05).

RESULTS: Five restorations (SE: 3; Set: 1; and ERm: 1) were lost after 18 months ($p>0.05$ for either criteria). Marginal staining occurred in four and 10% of the restorations evaluated ($p>0.05$), respectively, for USPHS and FDI criteria. Nine restorations were scored as bravo for marginal adaptation using the USPHS criteria and 38%, 40%, 36%, and 44% for groups ERm, ERd, Set, and SE, respectively, when the FDI criteria were applied ($p>0.05$). However, when semiquantitative scores (or SQUACE) for marginal adaptation were used, SE resulted in a significantly greater number of restorations, with more than 30% of the total length of the interface showing marginal discrepancy (28%) in comparison with the other groups (8%, 6%, and 8%, respectively, for ERm, ERd, and Set).

CONCLUSIONS: The clinical retention of the multimode adhesive at 18 months does not depend on the bonding strategy. The only differences between strategies were found for the parameter marginal adaptation, for which the FDI criteria were more sensitive than the USPHS criteria.

PMID: 23802645   DOI: 10.2341/13-045-C

[illegible]

Alkaline Peroxides Versus Sodium Hypochlorite as Overnight Denture Cleansers

Alkaline Peroxides Versus Sodium Hypochlorite as Overnight Denture Cleansers

Objective: This study compared two denture hygiene solutions for overnight immersion (alkaline peroxide and sodium hypochlorite) in terms of biofilm removal, by means of a randomized crossover trial.
Method: Twenty maxillary denture wearers brushed their dentures with a specific brush and neutral liquid soap after meals and soaked them overnight in 200 mL of one of the following media: C-Control: water at room temperature; E1-Experimental 1: one effervescent alkaline peroxide tablet (Corega tabs - GlaxoSmithKline) in warm water ; E2–Experimental 2: 0.5% sodium hypochlorite. The experimental period was 09 weeks; all participants employed each solution thrice for periods of 01 week each (03 repetitions of 07 days), according to a computer-generated random sequence. Maxillary dentures' intaglio surfaces were disclosed with 1% neutral red and photographed on baseline and following each 01 week period. The Image Tool 3.0 software quantified biofilm percentage area for each treatment, which were compared by means of a general linear method using baseline as a covariate followed by the Bonferroni test (α=.05).

Result: Mean results (in %, ±95% CI) were 25,4±7,3 (baseline), 19,3±7,7 (C), 17,1±7,8 (E1), and 8,4±6,9 (E2). The effect of treatments was significant (P=.050), with sodium hypochlorite leading to significantly lower results than the other conditions. Results for control and tablets were similar and no significant effect was found for the covariate or its interaction with treatment.

Conclusion: It was concluded that 0.5% sodium hypochlorite was more effective for an overnight immersion solution than the effervescent tablet tested with regard to denture biofilm removal.

Division: IADR/LAR General Session
Meeting: 2012 IADR/LAR General Session (Iguaçu Falls, Brazil)
Location: Iguaçu Falls, Brazil
Year: 2012
Final Presentation ID: 1385

Authors

- Paranhos Helena (Universidade de São Paulo, Ribeirão Preto, N/A, Brazil)
- Peracini Amanda (Universidade de São Paulo, Ribeirão Preto, N/A, Brazil)
- Andrade Ingrid Machado (Universidade de São Paulo, Ribeirão Preto, N/A, Brazil)
- Oliveira Viviane Cássia (Universidade de São Paulo, Ribeirão Preto, N/A, Brazil)
- Regis Rômulo Rocha (Universidade de São Paulo, Ribeirão Preto, N/A, Brazil)
- Souza Raphael Freitas (Universidade de São Paulo, Ribeirão Preto, N/A, Brazil)
- Silva-lovato Claúdia Helena (Universidade de São Paulo, Ribeirão Preto, N/A, Brazil)

SESSION INFORMATION
Poster Session
Clinical Trials
06/21/2012

Alkaline Peroxides Versus Sodium Hypochlorite as Overnight Denture Cleansers

Alkaline Peroxides Versus Sodium Hypochlorite for Removing Denture Biofilm: a Crossover Randomized Trial

Article *in* Brazilian dental journal 27(6):700-704 · December 2016
DOI: 10.1590/0103-6440201600913

Cite this publication

Amanda Peracini
Rômulo Rocha Regis
Raphael Freitas de Souza
34.51 · McGill University
+ 2
Helena de Freitas Oliveira Paranhos
27.53 · University of São Paulo

Abstract

This study evaluated the efficacy of cleanser solutions on denture biofilm removal by a crossover randomized clinical trial. Thirty two edentulous patients were instructed to brush their dentures (specific brush and liquid soap) three times a day (after breakfast, lunch and dinner) and to soak them (≥ 8 h) in: (C) control -water; (AP): alkaline peroxide; or (SH) 0.5% sodium hypochlorite. Each solution was used for 21 days (three cycles of 7 days). At the end of each cycle, the inner surfaces of maxillary dentures were disclosed (1% neutral red) and photographed (HX1 - Sony). Areas (total and stained biofilm) were measured (Image Tool software) and the percentage of biofilm calculated as the ratio between the area of the biofilm multiplied by 100 and total surface area of the internal base of the denture. Data were compared by means of generalized estimating equation (α=5%) and multiple comparisons (Bonferroni; α=1.67%). Immersion in SH reduced biofilm (%) (8.3 ± 13.3B) compared to C (18.2 ± 14.9A) and AP (18.2 ± 16.6A). The 0.5% sodium hypochlorite solution was the most efficacious for biofilm removal. Alkaline peroxides may not lead to further biofilm removal in patients with adequate denture maintenance habits.

Analgesia of Low Power Laser in TMJ Arthralgia

Analgesia of Low Power Laser in TMJ Arthralgia

Objectives : to evaluate a possible analgesic effect of low-level laser power (LBP) in cases of arthralgia of the temporomandibular joint (TMJ)

with or without drug therapy with Piroxicam.

Methods : We randomly allocated into three treatment groups 32 patients with arthralgia (group III) according to RDC / TMD. The Group A comprised 11 patients, received LBP and Piroxicam placebo. The Group B consisted of 10 patients that received LBP placebo and Piroxicam. And Group C consisted of 11 patients and received both LBP and Piroxicam . The protocol used for the laser was energy density of 100J/cm2, power of 100mW, 2.8 J of energy per point, during 28 seconds. Were irradiated ATM, the masseter and temporal muscles, both bilateral in 2 sessions a week for 2 weeks. Piroxicam was administered at a dose of 20 mg once daily for 10 days. To evaluate the analgesic effect was used visual analogue scale (VAS) of pain before and after each session of laser therapy, and after 30 days of completion of treatment.

Results: The three treatments were effective at reducing the pain reported by the patient, with significant differences between the pain reported before the treatment and after completion of treatment, $p = 0.0038$ for group A, $p = 0.0039$ for group B, $p = 0.0059$ for Group C. However, there was differences between the groups. After 30 days, there was a significant increase ($p = 0.0232$) of pain for patients in group A.

Conclusions: Piroxicam and LBP are effective at reducing pain in patients with TMJ arthralgia, with no differences between them. LBP did not show residual effect after 30 days of completion of treatment.

Division: IADR/LAR General Session
Meeting: 2012 IADR/LAR General Session (Iguaçu Falls, Brazil)
Location: Iguaçu Falls, Brazil
Year: 2012
Final Presentation ID: 2924

Authors

- Lara De Carli Marina (University of São Paulo, São Paulo, N/A, Brazil)
- Buzi Guerra Marcelo (University of São Paulo, São Paulo, N/A, Brazil)
- Borguezan Nunes Thais (University of São Paulo, São Paulo, N/A, Brazil)
- De Luca Carlos (University of São Paulo, São Paulo, N/A, Brazil)
- Canteras Di Matteo Rosana (University of São Paulo, São Paulo, N/A, Brazil)
- Costa Bolzan Marcelo (University of São Paulo, São Paulo, N/A, Brazil)
- Lusvarghi Witzel Andrea (University of São Paulo, São Paulo, N/A, Brazil)

SESSION INFORMATION
Poster Session
Oral Medicine 2
06/22/2012

Analgesia of Low Power Laser in TMJ Arthralgia

J Oral Rehabil. 2013 Mar;40(3):171-8. doi: 10.1111/joor.12022. Epub 2012 Dec 17.

Piroxicam and laser phototherapy in the treatment of TMJ arthralgia: a double-blind randomised controlled trial.

de Carli ML[1], Guerra MB, Nunes TB, di Matteo RC, de Luca CE, Aranha AC, Bolzan MC, Witzel AL.

Author information

Abstract

This study aimed to evaluate the efficacy of piroxicam associated with low-level laser therapy compared with single therapies in 32 patients presenting temporomandibular joint arthralgia in a random and double-blind research design. The sample, divided into laser + piroxicam, laser + placebo piroxicam and placebo laser + piroxicam groups, was submitted to the treatment with infrared laser (830 nm, 100 mW, 28 s, 100 J cm(-2)) at 10 temporomandibular joint and muscle points on each side during four sessions concomitant to take one capsule a day of piroxicam 20 mg during 10 days. The treatment was evaluated throughout four sessions and 30 days follow-up through visual analogue scale (VAS), maximum mouth opening and joint and muscle (temporal and masseter) pain on palpation. The results showed that all the study groups had a significant improvement in the VAS scores ($P < 0·05$), and there were no significant group differences. Piroxicam was effective in the reduction of joint and muscle pain on palpation ($P < 0·05$) and showed the lowest temporal pain ($P = 0·02$) at the 30-day follow-up. The combination of low-level laser therapy and piroxicam was not more effective than single therapies in the treatment of temporomandibular joint arthralgia. The use of piroxicam was more effective in the following 30 days.

PMID: 23252583 DOI: 10.1111/joor.12022

Are TT And TTB Effective In Color Matching?

Are TT And TTB Effective In Color Matching?

Introduction:
As a result of several investigations Toothguide Trainer (TT) and Toothguide Trainingbox (TTB) have a training effect in color matching using the 3D-Master Toothguide.

Aims and Objectives:

The aim of this study was to find out whether TT and TTB show any training effects, independent of the chosen color scale.

Material and Methods:

78 students from the dental schools of Leipzig, Greifswald, Olomouc and Berlin were included in this study. First a color perception check using the Ishihara Test was performed to exclude color deficient students. Participants were randomized into a study, 42 students (age range: 22 to 27 years; 31 % male; 69 % female) and a control group, 36 students (age range: 24 to 30 years; 43 % male; 57 % female). The study group started with a double blind introduction test, followed by the TT and TTB training, finishing with the final test. The control group only passed the initial and after a break the final test. These tests took place under defined light conditions, 5500 K. Eight samples, randomly chosen, seven of the VITA classical guide and one of the 3D Master color scale, were marked by barcodes. The color matching was arranged by the VITA classical scale.

Results:

The results of the initial and final tests of both groups were combined. For every sample the value deltaE was determined. The summation of all eight samples from the introduction and final tests offered a summarised deltaE. The differences between initial and final tests revealed the individual learning success. An advancement of deltaE at more than 2 was classified as clinically relevant. 47.6 % of the study group showed statistically significant better results than the control group, 33 %.

Conclusion:

TT and TTB are statistically effective in color matching independent of the used color scale.

Division: IADR/LAR General Session
Meeting: 2012 IADR/LAR General Session (Iguaçu Falls, Brazil)
Location: Iguaçu Falls, Brazil
Year: 2012
Final Presentation ID: 104

Authors

- Hannak Wolfgang (Charité, Berlin, N/A, Germany)
- Olms Constanze (Leipzig University, Leipzig, N/A, Germany)
- Klinke Thomas (Ernst-Moritz-Arndt University, Greifswald, N/A, Germany)
- Pirek Petr (Palacky University, Olomouc, N/A, Czech Republic)
- Jakstat Holger (Leipzig University, Leipzig, N/A, Germany)

SESSION INFORMATION
Oral Session
Color and Appearance
06/20/2012

Are TT And TTB Effective In Color Matching?

J Dent. 2013 Dec;41(12):1259-63. doi: 10.1016/j.jdent.2013.09.002. Epub 2013 Sep 19.

Randomized multi-centre study on the effect of training on tooth shade matching.

Olms C[1], Klinke T, Pirek P, Hannak WB.

Author information

1 Department of Prosthodontics and Material Science, University of Leipzig, Leipzig, Germany. Electronic address: Constanze.olms@medizin.uni-leipzig.de.

Abstract

OBJECTIVES: The aim of this study was to find out whether Toothguide Trainer, TT, and Toothguide Training Box, TTB, show any training effects, independent of the shade guide chosen.

METHODS: Students from four dental schools (N=78) were included in this study. The participants were randomized into a study, 42 students (age range: 19-27 years; 69% female, 31% male) and a control group of 36 students (age range: 19-30 years; 57% female, 43% male). The study group started with a double blind introduction test, followed by the TT and TTB training, finishing with the final test. The control group only passed the introduction and - after a break - the final test. Eight randomly chosen samples, seven of the Vita classical and one of the 3D-Master colour scale, were marked by barcodes. Colour matching was arranged by the Vita classical scale.

RESULTS: The results of the pre- and final tests of both groups were combined. For every sample, the value ΔE was determined. The summation of all eight samples from the introduction and final tests offered a summarized ΔE value. The differences between introduction and final tests revealed the individual learning success. 47.6% of the study group showed statistically significant better results than the control group, 33% (p=0.031).

CONCLUSION: TT and TTB show a positive effect of training on tooth shade matching independent of the colour scale used.

CLINICAL SIGNIFICANCE: Visual shade taking is the most frequent clinical method for shade determination. To increase better results in visual colour matching, TT and TTB training is used. This is the first study examining the training effect of TT and TTB using Vita classical scale.

Copyright © 2013 Elsevier Ltd. All rights reserved.

KEYWORDS: Aesthetics; Colour matching; Dental education; Prosthodontics; Shade guide; Tooth colour

PMID: 24056366 DOI: 10.1016/j.jdent.2013.09.002

Biofilm Formation After Clinical Disinfection of Relined Dentures

Objective: This study evaluated the effect of two disinfection methods for dentures on the biofilm formation of a hard chairside reline resin in different time period. Method: Hard chairside reline resin (Tokuyama Rebase Fast II) was used to reline complete dentures. Thirty patients, who used upper complete dentures, were randomly divided into 3 groups (n = 15). The dentures were cleansed according to three methods: CG (control group) – brushing with coconut soap and soft toothbrush, PG (Perborate group) – brushing according to previous methods and disinfection with warmed sodium perborate solution (Corega Tabs) for 5 min, once a day for 6 months and ChxG (Chlorhexidine Group) – brushing according to CG and disinfection with chlorhexidine digluconate 2% for 5 min once a day for 6 months. Quantitative microbial cultures were taken from the tissue side of each maxillary denture after 7, 15, 30, 90 and 180 days of dentures disinfection. The data were analyzed by 2-way repeated-measures ANOVAs, followed by Tukey Tests (p = 0.05). Result: CG group showed a significant decrease of micro-organisms in only at 15 days after relining. PG and ChxG groups showed a 100% reduction of cellular viability after 30 days. Conclusion: The disinfection with sodium perborate and chlorhexidine showed effectiveness in inhibiting of micro-organisms, after 30 days of daily disinfection.

Grants: Fapesp 2010/00916-7.

Division: IADR/LAR General Session
Meeting: 2012 IADR/LAR General Session (Iguaçu Falls, Brazil)
Location: Iguaçu Falls, Brazil
Year: 2012
Final Presentation ID: 1393

Authors

- Giampaolo Eunice (UNESP-Univ Estadual Paulista, Araraquara - SP, N/A, Brazil)
- Moffa Eduardo (UNESP-Univ Estadual Paulista, Araraquara - SP, N/A, Brazil)
- Izumida Fernanda (UNESP-Univ Estadual Paulista, São Carlos, N/A, Brazil)
- Jorge Janaina (UNESP-Univ Estadual Paulista, Araraquara, N/A, Brazil)
- Vergani Carlos (UNESP-Univ Estadual Paulista, Araraquara - SP, N/A, Brazil)

SESSION INFORMATION
Poster Session
Clinical Trials
06/21/2012

Biofilm Formation After Clinical Disinfection of Relined Dentures

Am J Dent. 2016 Feb;29(1):15-9.

Effectiveness of chemical disinfection on biofilms of relined dentures: A randomized clinical trial.

Moffa EB, Izumida FE, Jorge JH, Mussi MC, Siqueira WL, Giampaolo ET.

Abstract

PURPOSE: To evaluate the effect of disinfection with sodium perborate or chlorhexidine (when combined with brushing) on the removal of biofilm in relined dentures.

METHODS: Swabs were collected 48 hours after the relining procedure and at the follow-up time intervals of 7, 15, 30, 90, and 180 days. The dentures' surface roughness was measured at the same times. 45 subjects were randomly divided into three groups of 15 subjects each. The control group brushed with coconut soap and a soft toothbrush. The sodium perborate group followed the same procedure and also disinfected with sodium perborate solution for 5 minutes per day. The chlorhexidine group followed the control group procedure and disinfected with 2% chlorhexidine digluconate solution for 5 minutes per day. The number of colony forming units and the surface roughness were evaluated statistically by 2-way repeated-measure ANOVA ($\alpha = 0.05$).

RESULTS: The control group dentures exhibited similar levels of microbial cells throughout the experiment. However, after 15 days, no microbial growth was observed on the dentures for which either disinfection agent was used. There were no statistically significant differences in superficial roughness between the groups ($P = 0.298$). The disinfection agents used, combined with brushing, were able to remove the relined dentures' biofilm after 15 days of disinfection. Roughness was not a predominant factor in CFU reduction.

PMID: 27093771

Clinical evaluation: EDTA in the adhesion with self-etch adhesive system

Clinical evaluation: EDTA in the adhesion with self-etch adhesive system

Objective: To evaluate the clinical effectiveness of a self-etch system in non-carious cervical lesions [NCCL] after conditioning with EDTA in a 6-months randomized clinical study.
Method: Forty-eight patients with two similarly sized NCCL, good general healthy and having at least 20 teeth in occlusion were selected for this study. A total of ninety-six restorations were placed, half using the EDTA conditioning more Adper Easy One Adhesive system (EO+EDTA) and the other half using the same adhesive system according to the manufacturer´s directions. All restorations were placed by two calibrated operators following the manufacturers' instructions, except for the pre-conditioning with the EDTA for the experimental group. Two independent examiners evaluated the restorations at baseline and after six months according to the FDI criteria: fracture / retention, marginal adaptation, postoperative sensitivity, and caries adjacent to restoration.

Result: One restoration was lost, and another was rated with marginal fracture after 6 months, both in the EO group. The retention rates after 6 months for EO+EDTA and EO were 100% and 95.7%, respectively and no statistically significant difference was detected ($p>0.05$). At baseline, 63.5% of patients reported tooth sensitivity, but no patient report it in the immediate postoperative, and just 2.1% in the 6-months evaluation.

Conclusion: The present study demonstrates excellent clinical performance of the adhesive system Adper Easy One with and without the use of EDTA after 6 months.

Division: IADR/LAR General Session
Meeting: 2012 IADR/LAR General Session (Iguaçu Falls, Brazil)
Location: Iguaçu Falls, Brazil
Year: 2012
Final Presentation ID: 2007

Authors

- Luque, Issis (UEPG, Ponta Grossa, N/A, Brazil)
- Muñoz, Miguel Angel (Universidade Estadual De Ponta Grossa, Ponta Grossa- PR, N/A, Brazil)
- Mena-serrano, Alexandra (Universidade Estadual De Ponta Grossa, Ponta Grossa, N/A, Brazil)
- Hass, Viviane (UEPG, Ponta Grossa, N/A, Brazil)
- Reis, Alessandra (Universidade Estadual De Ponta Grossa, Ponta Grossa, N/A, Brazil)
- Loguercio, Alessandro (Universidade Estadual De Ponta Grossa, Ponta Grossa, N/A, Brazil)

Luque, Issis (UEPG, Ponta Grossa, N/A, Brazil)
Muñoz, Miguel Angel (Universidade Estadual De Ponta Grossa, Ponta Grossa- PR, N/A, Brazil)
Mena-serrano, Alexandra (Universidade Estadual De Ponta Grossa, Ponta Grossa, N/A, Brazil)
Hass, Viviane (UEPG, Ponta Grossa, N/A, Brazil)
Reis, Alessandra (Universidade Estadual De Ponta Grossa, Ponta Grossa, N/A, Brazil)
Loguercio, Alessandro (Universidade Estadual De Ponta Grossa, Ponta Grossa, N/A, Brazil)

SESSION INFORMATION

Poster Session
Resin-Based Restorative Materials II
06/22/2012

Clinical evaluation: EDTA in the adhesion with self-etch adhesive system

J Dent. 2015 Sep;43(9):1175-1183. doi: 10.1016/j.jdent.2015.04.013. Epub 2015 May 8.

Effect of EDTA conditioning on cervical restorations bonded with a self-etch adhesive: A randomized double-blind clinical trial.

Luque-Martinez I[1], Muñoz MA[1], Mena-Serrano A[2], Hass V[3], Reis A[3], Loguercio AD[4].

Author information

Abstract

OBJECTIVE: To compare the 18-month retention rates of composite restorations in non-carious cervical lesions [NCCLs] bonded with a self-etch adhesive with and without preliminary conditioning with EDTA.

METHODS: Forty-eight patients with two similar-sized NCCL were selected and randomly allocated to one of two groups. Two calibrated operators placed 96 restorations with a one-step self-etch adhesive (Adper Easy One, 3M ESPE). Half of the restorations were placed according to the manufacturer's instructions while, for the other half, the surfaces of the lesions were conditioned with 17% EDTA for 2 min prior to adhesive application. Two blinded and independent examiners evaluated the restorations at baseline, 6, 12, and 18 months, according to the FDI criteria. The comparison between groups in each period was conducted with the Fisher's exact test, and the performance of each group at the different periods was evaluated by McNemar's test (α=0.05).

RESULTS: After 18 months, significantly higher retention rates (95% CI) were observed for the EDTA group (95.5 [84.9-98.7]) than the control group (79.6% [65.5-88.9]) (p=0.02). Significant deterioration of the marginal adaptation and marginal discoloration were observed for both groups over the 18-month evaluation.

CONCLUSIONS: The preliminary conditioning with EDTA before application of a one-step self-etch adhesive significantly improved the retention rates of composite restorations in cervical lesions.

CLINICAL SIGNIFICANCE: Conditioning with EDTA is an alternative that improves the 18-month retention rate of cervical restorations bonded with a self-etch adhesive.

Copyright © 2015 Elsevier Ltd. All rights reserved.

KEYWORDS: Adhesive; Clinical trial; EDTA; Non-carious cervical lesion; Retention rate

PMID: 25963586 DOI: 10.1016/j.jdent.2015.04.013

Clinical Performance of Silorane-Based Composite Resin in Class II Restorations

Clinical Performance of Silorane-Based Composite Resin in Class II Restorations

Objective: The aim of this randomized, double-blind, controlled study was to compare the clinical performance of a silorane-based composite resin with a methacrylate-based composite resin in Class II restorations, after 18-month follow-up.
Method: After obtaining informed consent, 33 patients received 100 direct Class II composite-resin restorations (n=50) that were randomly allocated into a test group (Filtek P90® / Adhesive System - 3M ESPE) or control group (Filtek P60® /Adper SE Plus® - 3M ESPE). After one week, the restorations were finished and polished. A single operator performed all the restorative procedures. Two calibrated examiners (weight kappa⩾0.7) assessed the restorations at the baseline and after 18 months, according to modified United States Public Health System (USPHS) criteria. The parameters analyzed were marginal discoloration, marginal integrity, surface texture, anatomic form, postoperative sensitivity, secondary caries, proximal contact and radiographic aspect. Data were analyzed with Mann-Whitney U-test, Wilcoxon signed Rank, Kaplan-Meier statistics and Cox regression analysis ($p<0.05$).

Result: At 18-month follow-up, 90% of the restorations were evaluated. No significant difference was observed between the materials at baseline or after 18 months ($p>0.05$). The analysis of each material over the time showed significant difference in the control group for marginal discoloration ($p=0.046$) and surface texture ($p=0.005$). For the test group, there was significant difference for marginal discoloration ($p=0.025$), marginal integrity ($p=0.046$) and surface texture ($p=0.005$). Similar survival rates were observed for the test (92%) and the control (98%) groups ($p=0.168$). There was no significant effect of the restorative system ($p=0.233$), the tooth type ($p=0.668$) nor the number of restored surfaces ($p=0.377$) on the survival curves.

Conclusion: The clinical performance of silorane-based composite resin was similar to the well-known methacrylate systems. The restorations lost quality but remained acceptable after 18 months. The silorane resin system is acceptable to restore Class II cavities.

Division: IADR/LAR General Session
Meeting: 2012 IADR/LAR General Session (Iguaçu Falls, Brazil)
Location: Iguaçu Falls, Brazil
Year: 2012
Final Presentation ID: 2008

Authors

- Gonçalves, Fabiana Santos (Universidade Federal de Minas Gerais, Belo Horizonte, N/A, Brazil)
- Castro, Carolina (Universidade Federal de Minas Gerais, Belo Horizonte, N/A, Brazil)
- Freitas, Amanda Beatriz Dahdah Aniceto (Universidade Federal de Minas Gerais, Belo Horizonte, N/A, Brazil)
- Bueno, Audrey Cristina (Universidade Federal de Minas Gerais, Belo Horizonte, N/A, Brazil)
- Moreira, Allyson Nogueira (Universidade Federal de Minas Gerais, Belo Horizonte, N/A, Brazil)
- Magalhães, Cláudia (Universidade Federal de Minas Gerais, Belo Horizonte, N/A, Brazil)

Gonçalves, Fabiana Santos (Universidade Federal de Minas Gerais, Belo Horizonte, N/A, Brazil)
Castro, Carolina (Universidade Federal de Minas Gerais, Belo Horizonte, N/A, Brazil)
Freitas, Amanda Beatriz Dahdah Aniceto (Universidade Federal de Minas Gerais, Belo Horizonte, N/A, Brazil)
Bueno, Audrey Cristina (Universidade Federal de Minas Gerais, Belo Horizonte, N/A, Brazil)
Moreira, Allyson Nogueira (Universidade Federal de Minas Gerais, Belo Horizonte, N/A, Brazil)
Magalhães, Cláudia (Universidade Federal de Minas Gerais, Belo Horizonte, N/A, Brazil)

SESSION INFORMATION
Poster Session
Resin-Based Restorative Materials II
06/22/2012

Clinical Performance of Silorane-Based Composite Resin in Class II Restorations

J Contemp Dent Pract. 2012 May 1;13(3):251-6.

The short-term clinical performance of a silorane-based resin composite in the proximal contacts of class II restorations.

Goncalves FS[1], Castro CD, Bueno AC, Freitas AB, Moreira AN, Magalhaes CS.

⊕ **Author information**

Abstract

AIM: The aim of this randomized clinical trial was to compare the proximal contact of a silorane-based resin composite with a conventional methacrylate-based resin composite in class II restorations after a 6 months follow-up period.

MATERIALS AND METHODS: After obtaining informed consent, 33 patients were randomly allocated into a test group (Filtek P90/Adhesive System-3M ESPE) or control group (Filtek P60/ Adper SE Plus-3M ESPE), and 100 direct resin composite restorations (n = 50) were placed. A single operator performed the cavities and restorations. After rubber dam placement, a metal matrix and wooden wedge were placed. The restorative systems were applied according to the manufacturer's instructions. After 1 week, the restorations were finished and polished. The proximal contacts were assessed blindly and independently by two calibrated examiners (kW = 0.8) at the baseline and after 6 months according to a three-step grading criteria. Data were analyzed with the Mann-Whitney U-test and Wilcoxon signed Rank tests (a = 0.05).

RESULTS: After 6 months, 96% of the restoration contacts were present for evaluation. The frequencies of restorations classified as Bravo in control and test groups were 6 and 8% at the baseline, and 6.25 and 12.75% after 6 months. No significant difference was found between the restorative materials ($p > 0.05$; Mann-Whitney U-test) neither between baseline and 6 months period ($p > 0.05$; Wilcoxon signed Rank tests).

CONCLUSION: Both materials performed satisfactorily over 6 months follow-up period.

CLINICAL SIGNIFICANCE: The short-term clinical performance of a silorane-based resin composite in the proximal contacts of class II restorations was similar to the well-known methacrylate-based resin composite.

PMID: 22917991

Does inter-implant angulation affect attachment retention in two-implant overdentures?

Does inter-implant angulation affect attachment retention in two-implant overdentures?

Objectives: The purpose of this study was to evaluate the effect of inter-implant angulation on the retention of two attachment systems used in implant overdentures.
Methods: 24 participants (mean age=73.2 years; SD=3.1), wearing mandibular two-implant overdentures opposed by conventional maxillary complete dentures were equally and randomly divided into two groups. Each group received either ball attachments (Retentive Anchor, Straumann Canada Ltd) or cylindrical attachments (Locator, Zest Anchors Company, CA, USA). After one year, new attachments were installed according to a two-by-two crossover study design so that the group that initially had Locators received Retentive Anchors and vice versa. Inter-implant angulations were measured on posterior-anterior and lateral digital radiographs of the skull. The retention of each attachment type was measured by a digital force measurement device at baseline and 1-week, 3, 6 and 12 months post attachment installation. Data were analyzed using a mixed model regression analysis. All statistical hypothesis tests were two-sided and performed at the 0.05 significance level.

Results: Mean inter-implant angulations measured on posterior-anterior and lateral radiographs were 4.64 degrees (range 0.10–11.30) and 3.52 degrees (range 0.10–10.10), respectively. The effect of inter-implant angulation measured on the lateral radiographs was statistically significant on the Locator retention values only. It was found that retention decreases in average 1.1 Newton per degree of inter-implant angulation increase ($p=0.01$, standard error=0.38) after statistically adjusting for baseline retention, side of the mouth, crossover phase and follow-up period. There was no effect of inter-implant angulation on the retention values of Locator and Retentive Anchor attachments for measurements in frontal radiographs.

Conclusion: Increased inter-implant angulation appears to have higher impact on the retention of cylindrical attachments than on ball attachments. Further investigations are needed to assess the effect of larger inter-implant angulations on the amount of loss of retention and its clinical relevance.

Division: IADR/LAR General Session
Meeting: 2012 IADR/LAR General Session (Iguaçu Falls, Brazil)
Location: Iguaçu Falls, Brazil
Year: 2012
Final Presentation ID: 864

Authors

- Jabbour Zaher (McGill University, Montreal, QC, Canada)
- Fromentin Olivier (University Paris 7 Denis Diderot, and Service d'odontologie, Hôtel-Dieu, St. Germain En Laye, N/A, France)
- Lassauzay Claire (Université Clermont 1, UFR Odontologie EA3847, CHU Clermont Ferrand, Service d'Odontologie, Clermont-Ferrand, N/A, France)
- Abi Nader Samer (McGill University, Montreal, QC, Canada)
- Correa José (McGill University, Montreal, QC, Canada)
- Feine Jocelyne S. (McGill University, Montreal, QC, Canada)
- Albuquerque Rubens (McGill University, Montreal, QC, Canada)

SESSION INFORMATION
Poster Session
Implant Prosthodontics
06/21/2012

Does inter-implant angulation affect attachment retention in two-implant overdentures?

Clin Implant Dent Relat Res. 2014 Aug;16(4):565-71. doi: 10.1111/cid.12030. Epub 2013 Jan 10.

Effect of implant angulation on attachment retention in mandibular two-implant overdentures: a clinical study.

Jabbour Z[1], Fromentin O, Lassauzay C, Abi Nader S, Correa JA, Feine J, de Albuquerque Junior RF.

Author information

Abstract

PURPOSE: Attachment wear can affect the performance of mandibular two-implant overdentures (IODs). This prospective clinical study aimed to investigate the effect of interimplant angulation on the retention achieved by two attachment systems at different time points within 1 year of wearing IODs.

MATERIALS AND METHODS: Twenty-four patients (mean age = 73.2 years; standard deviation (SD) = 3.1) wearing IODs opposed by conventional maxillary complete dentures were randomly assigned to two groups in two-by-two crossover design. Retentive Anchor (RA) and Locator (LA) were installed in the IODs of both groups for 1 year, sequentially. Coronal and sagittal interimplant angulation were measured on posterior-anterior and lateral cephalometric radiographs. Retention was measured at baseline, 1 week, 3, 6, and 12 months postattachment installation. Data were analyzed using mixed models with $\alpha = 0.05$.

RESULTS: Mean coronal and sagittal interimplant angulations were 4.6 (SD = 2.9) and 3.5 (SD = 2.6) degrees, respectively. Only with LAs a statistically significant decrease was found in retention (average 1.1 Newton; standard error = 0.38; $p = .007$) per 1 degree increased sagittal interimplant angulation.

CONCLUSIONS: Increased interimplant angulation appears to have higher impact on the retention of LA than of RA attachments. The effect of larger interimplant angulation on the loss of attachment retention and its clinical implications should be further assessed.

© 2013 Wiley Periodicals, Inc.

KEYWORDS: implant attachment; interimplant angulation; mandibular two-implant overdenture; retention

PMID: 23305358 DOI: 10.1111/cid.12030

Early or Delayed Single Implants in Extraction Sockets: 10-year Results

Objectives: To report on the 10-year outcome of single implants inserted early or delayed following tooth extraction.
Methods: 32 patients that have received an implant for single tooth replacement were examined 1 and 10 years after treatment. Implants with a machined/dual acid etched surface (Osseotite; Biomet 3i) had been randomly inserted 10 days (early; n=14) or 3 months (delayed; n=18) post-extraction; 28% of the patients had periodontitis experience (i.e., > 30% bone loss in ⩾ 6 teeth). Dental controls or other treatment, including that related to the implants, were throughout the years provided by patients' own dentist. Clinical and radiographic peri-implant conditions at the 1- and 10-year controls were registered by a calibrated, masked examiner; information on smoking habits was also collected.

Results: At the 1-year control, probing depth (PD) ⩾ 5 mm was observed in 62.5% (8 early / 12 delayed) of the implants and 43.8% of the patients were smokers (i.e., > 5 cigarettes/day). After 10 years, a 100% survival of the implants was observed; peri-implant mucositis (i.e., bleeding on probing) was found in 81.2% (9 early / 15 delayed) of the implants, while 34.4% of the implants showed PD ⩾ 5 mm (4 early / 7 delayed; 5 in patients with previous periodontitis experience; 6 in smokers). Peri-implantitis (i.e., PD ⩾ 5 mm, bleeding on probing, and radiographic bone loss from 1 to 10 years) was observed in only one implant (3.1%); this patient had quit smoking a few years after implant installation.

Conclusion: Peri-implant mucositis was a common finding, while peri-implantitis was a rare event 10 years after implant placement. Implant placement protocol (early / delayed), periodontitis experience, increased probing depth at the 1-year control, and smoking were not associated with an increased risk for biological complications, including peri-implantitis, 10 years after treatment.

Division: IADR/LAR General Session
Meeting: 2012 IADR/LAR General Session (Iguaçu Falls, Brazil)
Location: Iguaçu Falls, Brazil
Year: 2012
Final Presentation ID: 2158

Authors

- Stavropoulos Andreas (Aarhus University, Aarhus C, N/A, Denmark)
- Sculean Anton (University of Berne, Berne, N/A, Switzerland)
- Schropp Lars (Aarhus University, Aarhus C, N/A, Denmark)

SESSION INFORMATION
Poster Session
Clinical Studies and Reviews
06/22/2012

Early or Delayed Single Implants in Extraction Sockets: 10-year Results

Early, delayed, or late single implant placement: 10-year results from a randomized controlled clinical trial.
(PMID:25040354)

Abstract | Citations | Related Articles | Data | BioEntities | External Links

Schropp L[1], Wenzel A, Stavropoulos A

Affiliations

Clinical Oral Implants Research [08 Oct 2013, 25(12):1359-1365]

Research Support, Non-U.S. Gov't, Randomized Controlled Trial, Journal Article

Abstract

The aim of this study was to present the 10-year clinical and radiographic data from a RCT on single-tooth implants placed early, delayed, or late after tooth extraction.Sixty-three patients were randomly allocated to three groups and received an implant on average 10 days (Ea), 3 months (De), or 17 months (La) after tooth extraction. Second-stage surgery was performed after 3 months of submerged healing; metal-ceramic crowns were cemented after one additional month. Standardized periapical radiographs were taken 1 week after implant placement (TP), 1 week (TC) and 1-1.5 year (T1) after crown delivery, and 10 years after implant placement (T10). Pocket depth (PD) and bleeding on probing were registered during controls (TC - T10).Two Ea and one De implants failed to osseointegrate. Seven patients (4 Ea, 1 De, and 2 La) were not available at T10 . No significant differences were found among groups regarding implant survival or radiographic peri-implant marginal bone levels (Ea: 1.15 ± 0.77; De: 1.53 ± 1.06; La: 1.42 ± 1.07) at T10 . Similarly, no differences were observed among groups in the number of implants with PD ≥ 5 mm (Ea: 29%; De: 35%; La: 44%) or the average depth of the sites with PD ≥ 5 mm (Ea: 5.4 ± 0.7; De: 6.1 ± 1.4; La: 5.4 ± 0.5) at T10 . Peri-implant mucositis was found in 70% of the cases; peri-implantitis was diagnosed only in two implants (1 De, 1 La) corresponding to 4.3%.Single-tooth implants placed early or delayed after tooth extraction show high survival rates and limited peri-implant marginal bone resorption or biological complications, similar to what is observed with implants placed according to the conventional (late) protocol.

Effect of Systemic Antibiotics on Implant Therapy- a Clinical Trial

Effect of Systemic Antibiotics on Implant Therapy- a Clinical Trial

Objectives: To determine the effect of various systemic antibiotic regimes on patient-reported outcome measures (PROMs) and post-surgical complications in patients undergoing implant installation.
Methods: 282 healthy adults (131 males, 151 females;19-80 years old), consecutively admitted to 7 study centres for conventional single implant installation were randomly assigned to one of four groups: Oral administration of: 1) 2g amoxicillin 1 hour prior to surgery (Positive Control (PC), n=71), 2) 2g amoxicillin immediately following surgery (Test 1 (T1), n=69), 3) 2g amoxicillin 1 hour prior to and 500mg thrice daily on days 2 and 3 postsurgically (Test 2 (T2),n=71) and 4) 2g of placebo 1 hour presurgically (Negative Control (NC), n=71). Subjects were examined by blinded examiners at Weeks 1, 2 , 4 and 8 for complications. Visual Analogue Scales (VAS) for pain, swelling, bruising and bleeding were obtained from the patients from Day 1 through 7 and Day 14. ANOVA was performed for the VAS. Descriptive statistics was applied to dichotomous data.

Results: At Week 1, 97.18% (PC), 95.59% (T1), 97.14% (T2), 94.37% (NC) of flaps were closed. Subjects with pain and swelling were highest for T2 (20.00%, 25.71% respectively). All flaps were closed with absence of pain at Week 8 for all groups. Suppuration was detected in one subject (T2) at Week 8. All implants were stable at Week 8 except one each from PC and NC. Bleeding, swelling, pain and bruising VAS were low for all groups and decreased from Day 1 to Day 14. There were no significant differences ($p<0.0001$) between various groups at the various time points.

Conclusion: For standard single implant placement, prophylactic systemic antibiotic coverage either before , after or before and after surgery yielded no differences in the incidence of postoperative complications and PROMs and hence, is redundant for routine implant surgery.

Division: IADR/LAR General Session
Meeting: 2012 IADR/LAR General Session (Iguaçu Falls, Brazil)
Location: Iguaçu Falls, Brazil
Year: 2012
Final Presentation ID: 3145

Authors

- Tan Wah Ching (National Dental Centre Singapore, Singapore, Singapore, N/A, Singapore)
- Mattheos Nikolaos (Griffith University, Gold Coast, N/A, Australia)
- Ong Marianne (National Dental Centre Singapore, Singapore, Singapore, N/A, Singapore)
- Lang Niklaus (The University of Hong Kong, Sai Ying Pun, N/A, Hong Kong)
- Wong May C.m. (The University of Hong Kong, Sai Ying Pun, N/A, Hong Kong)
- Meng Huanxin (Peking University, Beijing, N/A, China)
- Han Jie (Peking University, Beijing, N/A, China)
- Pjetursson Bjarni E. (University of Iceland, Reykjavík, N/A, Iceland)
- Sanz Mariano (Universidad Complutense Madrid, Madrid, N/A, Spain)
- Tsai Alex Yi-min (National Taiwan University, Taipei, N/A, Taiwan)

SESSION INFORMATION
Oral Session
Peri-Implantitis/Effect of Systemic Antibiotics/Impression Desinfection
06/23/2012

Effect of Systemic Antibiotics on Implant Therapy- a Clinical Trial

Clin Oral Implants Res. 2014 Feb;25(2):185-93. doi: 10.1111/clr.12098. Epub 2013 Jan 24.

Effect of systemic antibiotics on clinical and patient-reported outcomes of implant therapy - a multicenter randomized controlled clinical trial.

Tan WC[1], Ong M, Han J, Mattheos N, Pjetursson BE, Tsai AY, Sanz I, Wong MC, Lang NP; ITI Antibiotic Study Group.

⊕ Collaborators (26)

⊕ Author information

Abstract

OBJECTIVES: To determine the effect of various systemic antibiotic prophylaxis regimes on patient-reported outcomes and postsurgical complications in patients undergoing conventional implant installation.

MATERIAL AND METHODS: Three hundred and twenty-nine healthy adults in need of conventional implant installation were randomly assigned to one of four groups: (i) preoperatively 2 g of amoxycillin 1 h before surgery (positive control, PC), (ii) postoperatively 2 g of amoxycillin immediately following surgery (test 1, T1), (iii) preoperatively 2 g of amoxycillin 1 h before and 500 mg thrice daily on days 2 and 3 after surgery (test 2, T2), (iv) preoperatively 2 g of placebo 1 h before surgery (negative control, NC). Subjects were examined clinically by blinded examiners over 8 weeks after implant installation. In addition, Visual Analogue Scales (VAS) for pain, swelling, bruising and bleeding were obtained over 14 days. ANOVA was performed for the VAS. Chi-square tests were applied for postsurgical complications.

RESULTS: All VAS scores were low for all groups and decreased over time ($P < 0.001$). There were no significant differences for the VAS scores between the various groups at any time point ($P > 0.05$). There was only a significant difference in flap closure at week 4, where NC had 5% of the subjects not achieving complete wound closure compared to 0% for the three other groups ($P = 0.01$), with no other significant differences for any postsurgical complications ($P > 0.05$).

CONCLUSION: For standard single implant placement, prophylactic systemic antibiotics either before or after, or before and after the surgical procedure do not improve patient-reported outcomes or prevalence of postsurgical complications.

© 2013 John Wiley & Sons A/S. Published by Blackwell Publishing Ltd.

KEYWORDS: complications; dental implants; failures; implant dentistry; patient-reported outcomes; success; survival; systemic antibiotics

PMID: 23347336 DOI: 10.1111/clr.12098

Equivalent bone-level-alterations at implants with platform-switching and implants with matching-platforms

Equivalent bone-level-alterations at implants with platform-switching and implants with matching-platforms

Objective: The study aimed to test the hypothesis of no influence of platform-switching on crestal bone-level-changes.
Method: In a split-mouth design, two bone-level-implants (SICace, SICinvent AG, Basel, CH; diameter 4mm/ length 9.5mm) were interforaminally inserted in 32 edentulous subjects (baseline). Every subject received an implant with (3.3mm platform, test) and one without platform-switching (4mm platform, control). The allocation of test and control and of the loading-protocol, i.e. immediate loading (n=16 patients) or delayed loading after an open healing-period of 3 months (n=16 patients), was randomized before operation by means of a computer generated list. The implants were loaded with a prefabricated bar (SFI-Bar, C+M, Biel, CH). Patients were followed up at short intervals for monitoring of healing and for oral-hygiene-control. Standardized digital radiographs (baseline, 3 months, 6 months, 1 and 2 years after implant insertion) were independently evaluated for bone level alterations by two calibrated examiners. For equivalence-testing of bone-level-alterations at test- and control-implants at every time-point, Wilcoxon-signed-rank-tests with an equivalence-range of [-0.4mm; +0.4mm] were used. Statistical analysis for the influence of time, implant-type (test or control) and loading protocol on bone-levels were performed with the Brunner-Langer model.

Result: 2 years after implant insertion, the mean radiographic periimplant bone-level-alteration at the test-implants was -0.55 ±0.51mm and at the control-implants -0.58±0.54 mm. The median difference between the two treatment modalities was 0.04mm (IQR 0.48mm, adjusted non-parametric 90%CI: -0.08; 0.19). The bone-level-alterations of test- and control-implants were equivalent at every time point (all p<0.001). Crestal bone-level-alteration depended on time (p<0.001), on loading-protocol (p=0.021) and on the interaction of time and loading-protocol (p=0.013), but not on implant-type (p=0.650). Immediate loaded implants showed less bone-loss than delayed loaded.

Conclusion: The present randomized clinical trial proved the hypothesis of an equivalent bone-level-alteration of implants with platform-switching and implants with matching abutments.

Division: IADR/LAR General Session
Meeting: 2012 IADR/LAR General Session (Iguaçu Falls, Brazil)
Location: Iguaçu Falls, Brazil
Year: 2012
Final Presentation ID: 40

Authors

- Enkling Norbert (University of Bern, Bern, N/A, Switzerland)
- Albrecht Dominic (University of Bern, Bern, N/A, Switzerland)
- Gallo Nina (University of Bern, Bern, N/A, Switzerland)
- Dürstler Martina (University of Bern, Bern, N/A, Switzerland)
- Bayer Stefan (University of Bonn, Bonn, N/A, Germany)
- Stark Helmut (University of Bonn, Bonn, N/A, Germany)
- Katsoulis Joannis (University of Bern, Bern, N/A, Switzerland)
- Mericske-stern Regina (University of Bern, Bern, N/A, Switzerland)

SESSION INFORMATION
Oral Session
Implant Design and Platform Switch
06/20/2012

Equivalent bone-level-alterations at implants with platform-switching and implants with matching-platforms

J Dent Res. 2013 Dec;92(12 Suppl):139S-45S. doi: 10.1177/0022034513504953. Epub 2013 Oct 24.

Influence of platform switching on bone-level alterations: a three-year randomized clinical trial.

Enkling N[1], Jöhren P, Katsoulis J, Bayer S, Jervøe-Storm PM, Mericske-Stern R, Jepsen S.

Author information

Abstract

The concept of platform switching has been introduced to implant dentistry based on clinical observations of reduced peri-implant crestal bone loss. However, published data are controversial, and most studies are limited to 12 months. The aim of the present randomized clinical trial was to test the hypothesis that platform switching has a positive impact on crestal bone-level changes after 3 years. Two implants with a diameter of 4 mm were inserted crestally in the posterior mandible of 25 patients. The intraindividual allocation of platform switching (3.3-mm platform) and the standard implant (4-mm platform) was randomized. After 3 months of submerged healing, single-tooth crowns were cemented. Patients were followed up at short intervals for monitoring of healing and oral hygiene. Statistical analysis for the influence of time and platform type on bone levels employed the Brunner-Langer model. At 3 years, the mean radiographic peri-implant bone loss was 0.69 ± 0.43 mm (platform switching) and 0.74 ± 0.57 mm (standard platform). The mean intraindividual difference was 0.05 ± 0.58 mm (95% confidence interval: -0.19, 0.29). Crestal bone-level alteration depended on time ($p < .001$) but not on platform type ($p = .363$). The present randomized clinical trial could not confirm the hypothesis of a reduced peri-implant crestal bone loss, when implants had been restored according to the concept of platform switching.

TRIAL REGISTRATION: ClinicalTrials.gov NCT01917305.

KEYWORDS: alveolar bone loss; dental implant; dental implant-abutment connection; dental implant-abutment designs; dental implant-abutment interface; single-tooth dental implant

PMID: 24158333 PMCID: PMC3860064 DOI: 10.1177/0022034513504953

Ethanol-wet bonding Technique: Six-month Clinical Evaluation

Ethanol-wet bonding Technique: Six-month Clinical Evaluation

Objective: Most of current dental adhesive systems show favorable immediate results in terms of retention and sealing of bonded interface. Despite immediate efficacy, failures in this interface can be observed after a short period of time. The high hydrophilic characteristic of these systems may explain in part such behavior. Therefore, the objective of this split-mouth / randomized study was to determine the clinical performance of adhesive restorations using the ethanol-wet-bonding technique prior to the application of a bonding agent and composite resin in non-carious class V cavities compared to a three-step etch-and-rinse and one-step self-etching techniques.
Method: : 90 restorations (30 for each group) were placed by one operator in 17 patients. No cavity preparation was performed. After 6 months the restorations were assessed by two previously trained examiners using the modified Ryge criteria for marginal adaptation/staining (kappa=0.81) and retention (kappa=1.00). The data were analyzed by Kruskal-Wallis and Fisher's exact tests, respectively.

Result: No significant differences among groups up to 6 months period were observed for marginal adaptation (p=0,204), marginal staining (p=0,382) and retention (p=1,000).

Conclusion: At the time elapsed, restoration placed using the ethanol-wet-bonding technique presented equal performance to the others adhesive techniques employed. However, longer evaluation periods are suggested.

Division: IADR/LAR General Session
Meeting: 2012 IADR/LAR General Session (Iguaçu Falls, Brazil)
Location: Iguaçu Falls, Brazil
Year: 2012
Final Presentation ID: 2014

Authors

- Silva E Souza, Mario (Universidade Federal Do Para, Belém, N/A, Brazil)
- Araújo, Joyce Figueira (Universidade Federal Do Para, Belém, N/A, Brazil)
- Barros, Thais Figueiredo (Universidade Federal Do Para, Belém, N/A, Brazil)
- Braga, Esther Marina (Universidade Federal Do Para, Belém, N/A, Brazil)
- Ferreira, Railson Oliveira (Universidade Federal Do Para, Belém, N/A, Brazil)
- Loretto, Sandro Cordeiro (Universidade Federal Do Para, Belém, N/A, Brazil)

Silva E Souza, Mario (Universidade Federal Do Para, Belém, N/A, Brazil)
Araújo, Joyce Figueira (Universidade Federal Do Para, Belém, N/A, Brazil)
Barros, Thais Figueiredo (Universidade Federal Do Para, Belém, N/A, Brazil)
Braga, Esther Marina (Universidade Federal Do Para, Belém, N/A, Brazil)
Ferreira, Railson Oliveira (Universidade Federal Do Para, Belém, N/A, Brazil)
Loretto, Sandro Cordeiro (Universidade Federal Do Para, Belém, N/A, Brazil)

SESSION INFORMATION
Poster Session
Resin-Based Restorative Materials II
06/22/2012

Ethanol-wet bonding Technique: Six-month Clinical Evaluation

International journal of odontostomatology
On-line version ISSN 0718-381X

Int. J. Odontostomat. vol.10 no.2 Temuco Aug. 2016

http://dx.doi.org/10.4067/S0718-381X2016000200013

Int. J. Odontostomat., 10(2):267-276, 2016.

Services on Demand

Journal

Ethanol-Wet Bonding Technique: 18-month Clinical Evaluation

Técnica de Adhesión Húmeda en Etanol: Evaluación Clínica a los 18 Meses

ABSTRACT: The objective of this randomized clinical trial was to evaluate the clinical performance up to [illegible] months of restorations placed using ethanol-wet bonding technique (EWBT) compared with the three-step etc[illegible] and-rinse (TSER) and one-step self-etching (OSSE) approaches. Ninety-three non-carious cervical lesions (31 [illegible] each group) were restored by one experienced operator in 17 patients under relatively dry conditions using gingi[illegible] retraction cord, cotton rolls and saliva ejector. Each adhesive system was randomly allocated to one of randomiz[illegible] cervical lesions until the three groups were present in the same subject in equal amounts. The restorations we[illegible] evaluated at baseline, 6, 12 and 18 months by two blinded and calibrated examiners using the modified US Pub[illegible] Health Service guidelines (USPHS) for the following outcomes: retention (kappa= 1.00), staining and margi[illegible] adaptation (kappa=0.81) and analyzed by Fisher's exact and Kruskal-Wallis tests, respectively. No significa[illegible] differences were observed among groups after 18 months for any of the assessed criteria (p>0.05). The intr[illegible] group analysis performed by Cochran's test (for retention) and Wilcoxon test (for marginal adaptation/stainin[illegible] revealed significant differences between the time intervals baseline/18 months in marginal adaptation (p= 0.011[illegible] and retention (p= 0.0101) for OSSE and in marginal staining for TSER (0.0051) and EWBT (p= 0.0277) grou[illegible] The survival analysis for retention criteria and the overall clinical success were performed using a log-rank test a[illegible] did not show significant differences among groups (p> 0.05). All three adhesives protocols presented simi[illegible] clinical performance up to 18 months.

KEY WORDS: dentin, dentin-bonding agents, randomized controlled trial.

Thaís Andrade De Figueiredo Barros*; Joyce Figueira Da Araújo*[illegible] Esther Marina França Braga*; Patricia de Almeida Rodrigues Si[illegible] e Souza***; Sandro Cordeiro Loretto**** & Mário Honorato Silva E Souza Júnior******

* Escola Superior da Amazônia, Belém, Brazil.

** Federal University of Maranhão, Maranhão, Brazil.

*** Federal University of Pará, Belém, Brazil.

**** Department of Restorative Dentistry, Dental School, Federal University of Pará, Belém, Brazil.

Share

More

More

Permalink

Implant Overdentures and Dietary Intake: A Randomized Clinical Trial

Objective: We conducted a randomized clinical trial to determine whether providing simple implant-retained mandibular dentures to elderly individuals gives them a significantly better nutritional profile than those who receive conventional dentures.
Method: Two hundred-fifty five edentate people over the age of 65 were randomly assigned to receive mandibular two implant overdentures (n=127; mean age: 70.5years, SD: 5.0) or conventional dentures (n=128; mean age: 69.6, SD: 4.6) and maxillary conventional dentures. Dietary information was obtained by means of a 24-hour recall anda dietary intake questionnaire Six-month and one-year post-treatment outcomes measured were differences in dietary intake

Result:

Significant reductions in caloric intake were observed at 6 and 12 months post treatment in the conventional group (baseline: mean: 1551.2 kcal (SD:450.8), 6-month: mean: 1424.6 kcal (SD:300.1) and 12 months mean: 1462.5 kcal (SD: 273.5); p<0.05) but not in the implant group (baseline: mean: 1494.3 kcal (SD:445.1), 6 months: 1452.7 kcal (270.1) and 12 months mean: 1467.0 kcal (SD:302.6) p>0.05). After adjustment for age, sex, and baseline values, folate consumption tended to be higher in the implant, compared to the conventional, group (*B*=13.5, 95% CI: -0.8, 27.7, p=0.06). No between group differences were observed in caloric intake, protein, carbohydrates, fat, vitamin B6 or vitamin B12. Implant group participants reported fewer difficulties chewing pieces of beef, vegetables, fruits, bread crust than did those in the conventional group (p<0.0008); They were also significantly less likely to avoid certain foods because of their prosthesis or to cut fruits and vegetables into small pieces for chewing (p<0.05).

Conclusion:

This study suggests that the provision of implant overdentures did not lead to changes in dietary intake compared to new conventional dentures. Implant overdentures were associated with significant improvements in ability to chew and food habits.

Funded by the Canadian Institutes of Health Research and Straumann Canada Ltd.

Division: IADR/LAR General Session
Meeting: 2012 IADR/LAR General Session (Iguaçu Falls, Brazil)
Location: Iguaçu Falls, Brazil
Year: 2012
Final Presentation ID: 3017

Authors

- Awad Manal (University of Sharjah, Sharjah, N/A, United Arab Emirates)
- Gray-donald Katherine (McGill University, Montreal, QC, Canada)
- Morais Jose A. (McGill University, Montreal, N/A, Canada)
- Wollin Stephanie (McGill University, Montreal, QC, Canada)
- Johnson-down Louise (McGill University, Montreal, QC, Canada)
- Feine Jocelyne S. (McGill University, Montreal, QC, Canada)

SESSION INFORMATION
Oral Session
Oral Health Challenges in the Aging Population
06/23/2012

Implant Overdentures and Dietary Intake: A Randomized Clinical Trial

J Dent Res. 2013 Dec;92(12 Suppl):146S-53S. doi: 10.1177/0022034513504948. Epub 2013 Oct 24.

Do implant overdentures improve dietary intake? A randomized clinical trial.

Hamdan NM[1], Gray-Donald K, Awad MA, Johnson-Down L, Wollin S, Feine JS.

⊕ Author information

Abstract

People wearing mandibular two-implant overdentures (IOD) chew food with less difficulty than those wearing conventional complete dentures (CD). However, there is still controversy over whether or not this results in better dietary intake. In this randomized clinical trials (RCT), the amounts of total dietary fiber (TDF), macronutrients, 9 micronutrients, and energy in diets consumed by persons with IOD and CD were compared. Male and female edentate patients ≥ 65 yrs (n = 255) were randomly divided into 2 groups and assigned to receive a maxillary CD and either a mandibular IOD or a CD. One year following prosthesis delivery, 217 participants (CD = 114, IOD = 103) reported the food and quantities they consumed to a registered dietician through a standard 24-hour dietary recall method. The mean and median values of TDF, macro- and micronutrients, and energy consumed by both groups were calculated and compared analytically. No significant between-group differences were found (ps > .05). Despite quality-of-life benefits from IODs, this adequately powered study reveals no evidence of nutritional advantages for independently living medically healthy edentate elders wearing two-implant mandibular overdentures over those wearing conventional complete dentures in their dietary intake at one year following prosthesis delivery.

KEYWORDS: dental implant(s); edentulous/edentulism; geriatric dentistry; nutrition/nutritional sciences; prostheses; removable prosthodontics.

Comment in

Implant-retained overdentures did not have a significant improvement in dietary intake. [Evid Based Dent. 2014]

PMID: 24158335 PMCID: PMC3860059 DOI: 10.1177/0022034513504948

Masticatory Efficiency of Complete Denture Wearers with Reduced

Masticatory Efficiency of Complete Denture Wearers with Reduced Dental Arch

One obstacle when placing posterior artificial teeth during the manufacturing of complete dentures is the reduction of spatial relationship of the maxillae to the mandible. It affects the work time of the technician, once it requires abrasion of the denture base and the artificial tooth itself. Occasionally, the placement of the second molars is suppressed, for it does not affect aesthetics, phonetics and comfort. There are no reports in literature on this subject; despite studies involving shortened dental arches and dentures masticatory performance. Objective: The aim of this study was to compare masticatory efficiency of maxillomandibular complete denture wearers with reduced dental arch (without superior and inferior second molars) and complete dental arch. Method: Twenty subjects were divided in two groups randomly and received new dentures. Group 1 was given complete dentures without second molars and group 2 was given dentures with second molars. After post-insertion consults, the first masticatory efficiency test was taken with Optocal. Fifteen days after the first test, a new one was taken, in which second molars were positioned in group 1 and removed from group 2. Comminuted material was treated and sieved on a stack of sieves under vibration. Result: Mean and standard deviation of subjects' masticatory efficiency with complete dental arch was 4 and 0.68, respectively. While on the tests without second molars, mean and standard deviation were 4.22 and 0.92, respectively. Analyzing the moment of removal of second molars from the dental arch, mean of group 1 was 4.22 and standard deviation 0,63 and, group 2 3.78 and 0.72, respectively. Conclusion: According to the statistical analysis applied to this study (p<0,05), there were no differences on masticatory efficiency in complete dentures with or without second molars. Therefore, placing artificial teeth until first molars can be done when needed, without compromising masticatory efficiency.

Division: IADR/LAR General Session
Meeting: 2012 IADR/LAR General Session (Iguaçu Falls, Brazil)
Location: Iguaçu Falls, Brazil
Year: 2012
Final Presentation ID: 352

Authors

- Iegami Carolina (Universidade de Sao Paulo, Sao Paulo, N/A, Brazil)
- Barbosa Wallace Ferreira (Universidade de Sao Paulo, Sao Paulo, N/A, Brazil)
- Furuyama Ricardo Jun (Universidade de Sao Paulo, Sao Paulo, N/A, Brazil)
- Minagi Shogo (Okayama University, Okayama, N/A, Japan)
- Sugimoto Kyoko (Okayama University, Okayama, N/A, Japan)
- Lopes Danilo Melo (Universidade de Sao Paulo, Sao Paulo, N/A, Brazil)
- Campos Tomie Toyota (Universidade de Sao Paulo, Sao Paulo, N/A, Brazil)
- Tamaki Regina (Universidade de Sao Paulo, Sao Paulo, N/A, Brazil)

SESSION INFORMATION
Poster Session
Clinical Interventional Removable Prosthesis Studies
06/21/2012

Masticatory Efficiency of Complete Denture Wearers with Reduced Dental Arch

J Oral Rehabil. 2014 Aug;41(8):619-23. doi: 10.1111/joor.12179. Epub 2014 Apr 29.

Masticatory efficiency in complete denture wearers with reduced dental arches--a randomised cross-over study.

Iegami CM[1], Barbosa WF, Furuyama RJ, Lima JR, de Campos TT, Minagi S, Tamaki R.

Author information

Abstract

One obstacle to placing artificial posterior teeth in manufacturing complete dentures is a reduction of the space between the maxilla and the mandible. Occasionally, second molar placement is not performed, as it does not affect aesthetics, phonetics or comfort. The aim of this study was to compare the masticatory efficiency between patients wearing maxillary and mandibular complete dentures with reduced dental arches (without second molars) (WSM) and with full dental arches (FDA). Twenty subjects were divided into two groups and randomly received new complete dentures. Patients in Group 1 were given dentures WSM, and those in Group 2 were given dentures with FDA. After the post-placement visits, an initial masticatory efficiency test was performed with Optocal, an artificial test food. Fifteen days later, second molars were placed in Group 1 and removed from Group 2, and a new test was performed. Comminuted material was treated and sieved under vibration. The mean and standard deviation of masticatory efficiency with FDA were 10.4 and 8.1, respectively. In the tests WSM, the mean and standard deviation were 8.4 and 3.3, respectively. After removing the second molars in Group 2 and adding them in Group 1, the mean and standard deviation were 15.7 and 14.7 for Group 1 and 12.5 and 10.4 for Group 2, respectively. Within the limitations of this study, placing artificial teeth up to the first molars can be performed when needed without compromising masticatory efficiency.

KEYWORDS: artificial tooth; complete denture; mastication

PMID: 24779746 DOI: 10.1111/joor.12179

Peri-implant tissue response around Morse tapper and External hexagon implants

Peri-implant tissue response around Morse tapper and External hexagon implants

Objective: The aim of this study was to compare the clinical and radiographic tissue response after placement of two different dental implant designs used in immediately loaded mandibular implant-supported prostheses
Method: A total of 12 edentulous patients received 4 implants, two Morse Tapper (Neodent, Curitiba, Brazil) and two External Hexagon (Neodent, Curitiba, Brazil) in the anterior region of the mandible following a randomized split-mouth design. The distal implants were tilted and the central implants axially positioned in relation to the alveolar crest. Standardized intraoral radiographs were taken immediately after implant placement and in the follow-up visits after 4 months to evaluate bone response. Periodontal parameters consisting of probing depth and tissue width and height were also recorded in the same times

Result: It was observed statistically significant marginal bone loss, mainly around the tilted implants, both in mesial and distal faces of CM implants (-1.15mm and -0.91mm, respectively) and of HE implants (-0.84mm and -0.77mm). Around the central implants there was also bone loss, but lower in the CM implants when compared to HE. Probing depth showed increase in the mesial face, higher around the tilted implants CM (0.45mm) and HE (0.40mm) and decrease in the buccal and lingual faces whatever implant design and position. The distance from the abutment to the gingival margin showed a tendency to decrease in the CM implants and increase in the HE ones, but with no statistically significant difference. Finally there was no correlation between height and width of keratinized tissue and bone behavior

Conclusion: The data from this study show no difference between Morse taper and External hexagon implants, tilted or not, for rehabilitation with implant supported prosthesis fixed in the mandible when immediate loading protocol is used

Division: IADR/LAR General Session
Meeting: 2012 IADR/LAR General Session (Iguaçu Falls, Brazil)
Location: Iguaçu Falls, Brazil
Year: 2012
Final Presentation ID: 1516

Authors

- Sumiyassu Sueli (IMPPAR Odontologia, Londrina, N/A, Brazil)
- Sartori Ivete (ILAPEO - Instituto Latino Americano de Pesquisa e Ensino Odontológico, Curitiba, N/A, Brazil)
- Kuabara Marcos (IMPPAR Odontologia, Londrina, N/A, Brazil)
- Albuquerque Stella (IMPPAR Odontologia, Londrina, N/A, Brazil)
- Ferreira Edilson (IMPPAR Odontologia, Londrina, N/A, Brazil)
- Moreira Ana (ILAPEO - Instituto Latino Americano de Pesquisa e Ensino Odontológico, Curitiba, N/A, Brazil)

SESSION INFORMATION
Poster Session
Implant and Abutment Design and Interfaces
06/21/2012

Peri-implant tissue response around Morse tapper and External hexagon implants

Tissue response around Morse taper and external hexagon implants: preliminary results of a randomized split-mouth design

Autor: Sumiyassu, Sueli; Melo, Ana Cláudia Moreira; Sartor, Ivete Aparecida de Mattias; Fontão, Flávia Noemi Gasparini Kiatake; Ferreira, Edilson José; Thomé, Geninho.

Título: Tissue response around Morse taper and external hexagon implants: preliminary results of a randomized split-mouth design / Resposta tecidual ao redor de implantes cone Morse e hexágono externo: resultados preliminares de um estudo em boca dividida

Fonte: Salusvita;32(1), 2013. ilus, tab.

Idioma: pt.

Resumo: Introduction: the rehabilitation of edentulous mandible by four interforaminal implants with the distal ones inserted tilted in order to avoid proximity with the mentual foramen as well as improving prosthesis support have been argued as an adequate design for implant supported fixed prosthesis. Objective: the aim of this study was to compare tissue response around immediately loaded mandibular dental implants with two different prosthetic connections. Methods: a total of 48 implants were inserted in the anterior region of the mandible of 12 edentulous patients following a randomized split-mouth design. Morse Taper and External Hexagon implants were equally divided into each patient. Distal implants were tilted and central implants axially positioned in relation to the alveolar crest. Standardized intraoral radiographs were taken immediately after implant placement and after 6 months. Periodontal parameters (probing depth and keratinized tissue width and height) were recorded at the same times. Wilcoxon test was used. Results and Discussion: It was observed stability of the gingival margin and decrease in probing depth around Morse taper implants and increase in external hexagon implants. There was marginal bone increase in the mesial face (0.27 mm) and decrease at the distal face (-0.87 mm) of Morse taper and at both proximal faces of external hexagonimplants (-1.06 mm and -0.80 mm, respectively). Morse taper tilted implants showed maintenance of bone height (0.03 mm and-0.02mm, mesial and distal) while external hexagon implants showed resorption (-1.82 mm and -0.75 mm, mesial and distal). Axially positioned implants showed bone loss, either Morse taper (-0.72 and-0.67mm, mesial and distal) or external hexagon (-0.69 and -0.83mm).

Prophylaxis Vs Antimicrobial Therapy in Patients Undergoing Oral Implants

Prophylaxis Vs Antimicrobial Therapy in Patients Undergoing Oral Implants ORLANDO RIAÑO DDS, GERMAN DURAN DDS, IVONNE GASCA DDS, BEATRIZ CEPEDA MD MS Introduction: Controversy persists about the superiority of prophylaxis versus therapy to prevent postsurgical infection. Objective: To compare the control of bacterial infection prophylaxis vs antimicrobial therapy in patients receiving implants. Methods: Thirty patients between 30 and 70 years were randomly assigned to two groups. Fifteen patients received prophylaxis (amoxicillin 2g PO, 30min before surgery). The remainder received therapy (amoxicillin 500mg BID for seven days). Signs of infection were assessed: pain, redness, swelling and discharge, at 3, 8 and 15 days after the procedure. The number of leukocytes, neutrophils and lymphocytes was determined one week after. Categorical variables were compared using X2 and likelihood ratio. Numerical tests are performed by T or U tests, according to the validation of the assumptions of normality and homoscedasticity. Results: The infection rate was significantly lower in the prophylaxis group compared with group therapy. The prophylaxis group showed a lower proportion of signs and symptoms of infection (6.7% versus 13.3%), without reaching statistical significance (p=0.27). the average fraction of leukocytes, lymphocytes and neutrophils in the blood picture were within normal range in both groups (p = 0.79, 0.91 and 0.82, respectively) Conclusions: The lower infection rate confirms previously published results, which support the superiority of prophylactic antibiotics on therapy to prevent postoperative infections.

Division: IADR/LAR General Session
Meeting: 2012 IADR/LAR General Session (Iguaçu Falls, Brazil)
Location: Iguaçu Falls, Brazil
Year: 2012
Final Presentation ID: 2862

Authors

- Riano Orlando (CIEO University, Bogota, N/A, Colombia)
- Duran German (CIEO University, Bogota, N/A, Colombia)
- Gasca Ivonne (CIEO University, Bogota, N/A, Colombia)
- Cepeda Beatriz (CIEO University, Bogota, N/A, Colombia)

SESSION INFORMATION
Poster Session
Peri-implant Disease/Compromised Bone/Failure
06/22/2012

Prophylaxis Vs Antimicrobial Therapy in Patients Undergoing Oral Implants

Profilaxis versus tratamiento antimicrobiano en pacientes sometidos a implantes orales Antibiotic prophylaxis versus antimicrobial therapy in patients undergoing dental implants

Revista Colombiana de Cirugía 2012 0

Profilaxis versus tratamiento antimicrobiano en pacientes sometidos a implantes orales Antibiotic prophylaxis versus antimicrobial therapy in patients undergoing dental implants

Beatriz Cepeda,Orlando Ria ? o,Germán Durán,Ivonne Gasca

Keywords: implante dental , infección , control de infección dental , profilaxis antibiótica , dental implant , infection , infection control , dental , Antibiotic Prophylaxis

Abstract:

Introduccion. Persiste la controversia en cuanto a la superioridad de la profilaxis sobre el tratamiento antimicrobiano para evitar la infeccion posquirurgica. Objetivo. Comparar el control de la infeccion bacteriana mediante el uso de profilaxis con el tratamiento antimicrobiano en pacientes sometidos a implantes. Materiales y metodos. Los pacientes de la muestra (n=30), con edades entre los 30 y los 70 anos de edad, se asignaron aleatoriamente en dos grupos. Quince recibieron profilaxis de 2 g de amoxicilina 30 minutos antes de la cirugia y los restantes recibieron tratamiento de 500 mg de amoxicilina cada ocho horas por siete dias. Se valoraron los signos de infeccion .dolor, rubor, tumefaccion y exudacion. a los 3, 8 y 15 dias despues del procedimiento. Una semana despues se determino el numero de leucocitos, neutrofilos y linfocitos. Las variables categoricas se compararon mediante la prueba de ji al cuadrado y razon de verosimilitud. Los contrastes numericos se valoraron mediante la prueba t de Student, segun la validacion de los supuestos de normalidad y homocedasticidad (=0,05, en todos los casos). Resultados. La proporcion de infeccion fue menor en el grupo de profilaxis que en el grupo de tratamiento. El grupo de profilaxis tuvo una menor proporcion de signos y sintomas de infeccion (6,7 % Vs. 13,3 %), sin alcanzar significancia estadistica (p=0,27). El promedio de la fraccion de leucocitos, linfocitos y neutrofilos en el cuadro hematico, estuvo dentro del rango de normalidad en los dos grupos (p=0,79, p=0,91 y p=0,82, respectivamente). Conclusiones. La menor proporcion de infeccion confirma los resultados previamente publicados, que sustentan la superioridad de la profilaxis antibiotica sobre el tratamiento antibiotico para prevenir las infecciones posquirurgicas. Introduction: There is persistent controversy over the superiority of antibiotic prophylaxis versus antimicrobial therapy in avoiding postoperative infection following dental implants. Objective: To compare the use of antibiotic prophylaxis versus antimicrobial therapy in the control of infection in patients undergoing dental implant surgery. Method: The sample consisted of thirty patients, ages between 30 and 70 years that were randomly divided into two groups, 15 patients each. The average age in the prophylaxis group was 52 years and 59 in the therapy group, and the average was 2 implants placed per patient. One group

Repositioning occlusal splints and NTI-tss in Temporomandibular Joint arthralgia

Repositioning occlusal splints and NTI-tss in Temporomandibular Joint arthralgia

Objectives: The aim of the study was to test the hypothesis that the treatment with intraoral devices (occlusal splints and NTI-tss device) is effective in the control of Temporomandibular joint (TMJ) arthralgia. **Methods:** Sixty individuals with TMJ arthralgia (Research Diagnostic Criteria for Temporomandibular Disorders - RDC/TMD) were randomly divided into three groups, according to the treatment received: a) repositioning occlusal splint and counseling; b) NTI-tss device and counseling and c) only counseling for behavioral changes (control). The devices were used in partial time (only during sleep), and patients were evaluated for pain intensity (Visual Analogue Scale), presence of joint noises, active mouth opening, TMJ pressure pain threshold (PPT) and level of comfort of intraoral devices after 2, 6 and 12 weeks. The data were analyzed with Kruskal-Wallis and ANOVA (significance level of 5%). **Results:** All three groups showed a reduction in pain intensity ($p<0.05$), although no differences were detected for PPT and joint noises. Higher level of comfort and an earlier effect on pain reduction were found in the occlusal appliance group ($p<0.05$). **Conclusion:** Counseling and occlusal devices seems to be useful in the reduction of TMJ pain in a short-term analysis. Long-term studies with the NTI device are needed to establish its effectiveness and absence of adverse dental effects.

Division: IADR/LAR General Session
Meeting: 2012 IADR/LAR General Session (Iguaçu Falls, Brazil)
Location: Iguaçu Falls, Brazil
Year: 2012
Final Presentation ID: 2222

Authors

- Cunha Carolina (Bauru School of Dentistry - University of Sao Paulo, Bauru, N/A, Brazil)
- Correa Ana Silvia (Bauru School of Dentistry - University of Sao Paulo, Bauru, N/A, Brazil)
- Pinto Livia Maria (Bauru School of Dentistry - University of Sao Paulo, Bauru, N/A, Brazil)
- Alencar Eloisa (Bauru School of Dentistry - University of Sao Paulo, Bauru, N/A, Brazil)
- Bonjardim Leonardo (Professor of Federal University of Sergipe and Post-doctoral Student of Bauru School of Dentistry - University of São Paulo, Bauru, N/A, Brazil)
- Conti Paulo (Bauru School of Dentistry University of Sao Paulo, Bauru, N/A, Brazil)

SESSION INFORMATION
Poster Session
Orofacial Pain: Epidemiology, Etiology, Assessment, and Treatment
06/22/2012

Repositioning occlusal splints and NTI-tss in Temporomandibular Joint arthralgia

Journal of Applied Oral Science

Print version ISSN 1678-7757*On-line version* ISSN 1678-7765

J. Appl. Oral Sci. vol.23 no.5 Bauru Sept./Oct. 2015 Epub July 21, 2015

http://dx.doi.org/10.1590/1678-775720140438

ORIGINAL ARTICLES

Management of painful temporomandibular joint clicking with different intraoral devices and counseling: a controlled study

Paulo César Rodrigues CONTI[1]

Ana Silvia da Mota CORRÊA[1]

José Roberto Pereira LAURIS[2]

Juliana STUGINSKI-BARBOSA[1]

[1]Department of Prosthodontics, Bauru School of Dentistry, University of São Paulo, Bauru, SP, Brazil.
[2]Department of Department of Pediatric Dentistry, Orthodontics and Community Health, Bauru School of Dentistry, University of São Paulo, Bauru, SP, Brazil.

Permalink

ABSTRACT

Objective

The benefit of the use of some intraoral devices in arthrogenous temporomandibular disorders (TMD) patients is still unknown. This study assessed the effectiveness of the partial use of intraoral devices and counseling in the management of patients with disc displacement with reduction (DDWR) and arthralgia.

Materials and Methods

A total of 60 DDWR and arthralgia patients were randomly divided into three groups: group I (n=20) wore anterior repositioning occlusal splints (ARS); group II (n=20) wore the Nociceptive Trigeminal Inhibition Clenching Suppression System devices (NTI-tss); and group III (n=20) only received counseling for behavioral changes and self-care (the control group). The first two groups also received counseling. Follow-ups were performed after 2 weeks, 6 weeks and 3 months. In these sessions, patients were evaluated by means of a visual analogue scale, pressure pain threshold (PPT) of the temporomandibular joint (TMJ), maximum range of motion and TMJ sounds. Possible adverse effects were also recorded, such as discomfort while using the device and occlusal changes. The results were analyzed with ANOVA, Tukey's and Fisher Exact Test, with a significance level of 5%.

Results

Groups I and II showed improvement in pain intensity at the first follow-up. This progress was recorded only after 3 months in Group III. Group II showed an increased in joint sounds frequency. The PPT values, mandibular range of motion and the number of occlusal contacts did not change significantly.

Conclusion

The simultaneous use of intraoral devices (partial time) plus behavioral modifications seems to produce a more rapid pain improvement in patients with painful DDWR. The use of NTI-tss could increase TMJ sounds. Although intraoral devices with additional counseling should be considered for the management of painful DDWR, dentists should be aware of the possible side effects of the intraoral device's design.

Key words: Temporomandibular joint disorders; Temporomandibular joint disc; Arthralgia; Occlusal splints; Behavioral control

Silorane-Based Composite in Class I Restorations: A Split-Mouth Clinical Trial

Silorane-Based Composite in Class I Restorations: A Split-Mouth Clinical Trial

Objective: The aim of this split-mouth, double-blind clinical trial was to compare the performance of a silorane-based composite-resin with a methacrylate-based composite-resin in Class I restorations, after 1-year follow-up.
Method: This study received approval from the local ethics committee. After obtaining the informed consent, the subjects (n=35) received at least one pair of restorations, which were randomly allocated into test (Filtek™ P90/P90 Adhesive System, 3M/ESPE) and control (Filtek™ P60/Adper™ SE Plus, 3M/ESPE) groups. A single operator performed all the restorations, following the clinical protocols and manufacturer's instructions. After one week, they were finished, polished and independently assessed by two trained examiners (weight kappa ⩾ 0.7) using modified United States Public Health System criteria. After one year, the same examiners reassessed the restorations. Wilcoxon test compared the frequencies of Alfa (A), Bravo (B) and Charlie (C) scores in test and control groups and between baseline and one-year recall.

Result: All patients were present at baseline and 31 patients (88.6%) returned at recall. The frequencies (%) of restorations scored A/B/C at baseline, for test and control groups were, respectively: Marginal Discoloration (100/0/0), (100/0/0); Marginal Integrity (97.1/2.9/0), (100/0/0); Surface Texture (100/0/0), (100/0/0); Anatomic Contour (94.3/5.7/0), (100/0/0); Postoperative Sensitivity (97.2/2.8/0), (97.2/2.8/0); Recurrent Caries (100/0/0), (100/0/0). At recall, frequencies (%) of A/B/C, for test and control groups were, respectively: Marginal Discoloration (100/0/0), (100/0/0); Marginal Integrity (93.6/6.4/0), (100/0/0); Surface Texture (96.8/3.2/0), (87.1/12.9/0); Anatomic Contour (100/0/0), (100/0/0); Postoperative Sensitivity (96.8/3.2/0), (100/0/0); Recurrent Caries (100/0/0), (100/0/0). No statistically significant differences were found between groups at baseline and at recall (p>0.05). No significant differences were found when each group was compared through time (p>0.05).

Conclusion: The clinical performance of silorane-based composite-resin proved similar to that achieved with methacrylate resin. A silorane-based composite-resin could be a promising alternative for Class I restorations.

Division: IADR/LAR General Session
Meeting: 2012 IADR/LAR General Session (Iguaçu Falls, Brazil)
Location: Iguaçu Falls, Brazil
Year: 2012
Final Presentation ID: 2017

Authors

- Castro, Carolina (Universidade Federal de Minas Gerais, Belo Horizonte, N/A, Brazil)
- Gonçalves, Fabiana (Universidade Federal de Minas Gerais, Belo Horizonte, N/A, Brazil)
- Bueno, Audrey (Universidade Federal de Minas Gerais, Belo Horizonte, N/A, Brazil)
- Freitas, Amanda (Universidade Federal de Minas Gerais, Belo Horizonte, N/A, Brazil)
- Moreira, Allyson (Universidade Federal de Minas Gerais, Belo Horizonte, N/A, Brazil)
- Magalhães, Cláudia (Universidade Federal de Minas Gerais, Belo Horizonte, N/A, Brazil)

SESSION INFORMATION
Poster Session
Resin-Based Restorative Materials II
06/22/2012

Castro, Carolina (Universidade Federal de Minas Gerais, Belo Horizonte, N/A, Brazil)
Gonçalves, Fabiana (Universidade Federal de Minas Gerais, Belo Horizonte, N/A, Brazil)
Bueno, Audrey (Universidade Federal de Minas Gerais, Belo Horizonte, N/A, Brazil)
Freitas, Amanda (Universidade Federal de Minas Gerais, Belo Horizonte, N/A, Brazil)
Moreira, Allyson (Universidade Federal de Minas Gerais, Belo Horizonte, N/A, Brazil)
Magalhães, Cláudia (Universidade Federal de Minas Gerais, Belo Horizonte, N/A, Brazil)

Silorane-Based Composite in Class I Restorations: A Split-Mouth Clinical Trial

J Contemp Dent Pract. 2012 May 1;13(3):251-6.

The short-term clinical performance of a silorane-based resin composite in the proximal contacts of class II restorations.

Goncalves FS[1], Castro CD, Bueno AC, Freitas AB, Moreira AN, Magalhaes CS.

Author information

Abstract

AIM: The aim of this randomized clinical trial was to compare the proximal contact of a silorane-based resin composite with a conventional methacrylate-based resin composite in class II restorations after a 6 months follow-up period.

MATERIALS AND METHODS: After obtaining informed consent, 33 patients were randomly allocated into a test group (Filtek P90/Adhesive System-3M ESPE) or control group (Filtek P60/ Adper SE Plus-3M ESPE), and 100 direct resin composite restorations (n = 50) were placed. A single operator performed the cavities and restorations. After rubber dam placement, a metal matrix and wooden wedge were placed. The restorative systems were applied according to the manufacturer's instructions. After 1 week, the restorations were finished and polished. The proximal contacts were assessed blindly and independently by two calibrated examiners (kW = 0.8) at the baseline and after 6 months according to a three-step grading criteria. Data were analyzed with the Mann-Whitney U-test and Wilcoxon signed Rank tests (a = 0.05).

RESULTS: After 6 months, 96% of the restoration contacts were present for evaluation. The frequencies of restorations classified as Bravo in control and test groups were 6 and 8% at the baseline, and 6.25 and 12.75% after 6 months. No significant difference was found between the restorative materials ($p > 0.05$; Mann-Whitney U-test) neither between baseline and 6 months period ($p > 0.05$; Wilcoxon signed Rank tests).

CONCLUSION: Both materials performed satisfactorily over 6 months follow-up period.

CLINICAL SIGNIFICANCE: The short-term clinical performance of a silorane-based resin composite in the proximal contacts of class II restorations was similar to the well-known methacrylate-based resin composite.

PMID: 22917991

Simplified Versus Conventional Method for Complete Denture Fabrication: Cost Analysis

Simplified Versus Conventional Method for Complete Denture Fabrication: Cost Analysis.

Complete denture fabrication involves a series of complex technical procedures. Nevertheless, simplified methods may be as effective as conventional ones albeit the lower use of time and resources.
Objective: The aim of this study was to quantify the costs of complete denture fabrication by means of a simplified method compared with a conventional protocol

Method: A sample of edentulous patients needing conventional maxillary and mandibular complete dentures was randomly divided into: group S, which received dentures fabricated by means of a simplified method, and group C, which received conventionally fabricated dentures. We calculated direct and indirect costs for each participant including unscheduled procedures. This study assessed 19 and 20 participants allocated in groups S and C, respectively, and comparisons between groups were conducted by means of the Mann-Whitney and Student's t test (α=0,05)

Result: Complete denture fabrication demanded median time periods of 173,2 and 284,5 min from the operator for groups S and C respectively, and 46.6 and 61.7 min from the dental assistant (significant differences, $P<0.05$). There was no difference between groups regarding post-insertion adjustments. Group S also showed lower values than C during the fabrication stage, but not during adjustments, for costs with materials and time spent by patients. In summary, the simplified method reduced direct treatment costs in 34.9%.

Conclusion: It can be concluded that the simplified method is less costly for patients and health system when compared with a conventional protocol for the rehabilitation of edentulous patients.

Division: IADR/LAR General Session
Meeting: 2012 IADR/LAR General Session (Iguaçu Falls, Brazil)
Location: Iguaçu Falls, Brazil
Year: 2012
Final Presentation ID: 344

Authors

- Della Vecchia Maria (University of São Paulo, Ribeirão Preto, N/A, Brazil)
- Cunha Tatiana Ramirez (University of São Paulo, Ribeirão Preto, N/A, Brazil)
- Regis Rômulo Rocha (University of São Paulo, Ribeirão Preto, N/A, Brazil)
- Ribeiro Adriana (University of São Paulo, Ribeirão Preto, N/A, Brazil)
- Lovato Da Silva Cláudia Helena (University of São Paulo, Ribeirão Preto, N/A, Brazil)
- Souza Raphael (University of São Paulo, Ribeirão Preto, N/A, Brazil)

SESSION INFORMATION
Poster Session
Clinical Interventional Removable Prosthesis Studies
06/21/2012

Simplified Versus Conventional Method for Complete Denture Fabrication: Cost Analysis.

Journal of Prosthodontics
Implant, Esthetic, and Reconstructive Dentistry

ACP Your smile. Our specialty.

Original Article

A Randomized Trial on Simplified and Conventional Methods for Complete Denture Fabrication: Cost Analysis

Maria Paula Della Vecchia DDS, Rômulo Rocha Regis DDS, Tatiana Ramirez Cunha DDS, Ingrid Machado de Andrade DDS, PhD, Julio César Souza da Matta CDT, Raphael Freitas de Souza DDS, PhD

First published: 26 July 2013 | https://doi.org/10.1111/jopr.12090 | Cited by: 7

Abstract

Purpose

This study aimed to quantify the costs of complete denture fabrication by a simplified method compared with a conventional protocol.

Materials and Methods

A sample of edentulous patients needing conventional maxillary and mandibular complete dentures was randomly divided into group S, which received dentures fabricated by a simplified method, and group C, which received conventionally fabricated dentures. We calculated direct and indirect costs for each participant including unscheduled procedures. This study assessed 19 and 20 participants allocated into groups S and C, respectively, and comparisons between groups were conducted by the Mann-Whitney and Student's *t*-test ($\alpha = 0.05$).

Results

Complete denture fabrication demanded median time periods of 173.2 and 284.5 minutes from the operator for groups S and C respectively, and 46.6 and 61.7 minutes from the dental assistant (significant differences, $p < 0.05$). There was no difference between groups regarding postinsertion adjustments. Group S showed lower values for costs with materials and time spent by patients than group C during the fabrication stage, but not during adjustments.

Conclusions

The median direct cost of complete denture treatment was 34.9% lower for the simplified method. It can be concluded that the simplified method is less costly for patients and the health system when compared with a conventional protocol for the rehabilitation of edentulous patients.

The Effect of Overnight Storage on Removable Denture Biofilm

Objective: The aim of this study was to evaluate the effect of 3 different overnight storage modes of removable full dentures on microbial growth on the dentures.
Method: 60 elderly edentulous persons with 2 removable full dentures were included in the study. All dentures were disinfected using chlorhexidine (PerioAid gel, Dentaid, The Netherlands) prior to the study. Biofilm formation was allowed during one week on 2 contralateral 5-mm-diameter measuring areas (1+2) on the lower dentures. A custom-made mold assured the reproducibility of the measuring areas where the samples were taken. After one week (=baseline), all plaque was removed from measuring area 1 (1-week biofilm baseline), after which the dentures were disinfected again, except for measuring area 2. At this point, the patients were randomly divided in 3 test groups according to the following overnight storage modes: A) dry, B) in water, and C) in water with a cleansing tablet (Corega antibacterie, GlaxoSmithKline, UK). After another week, plaque samples were taken from measuring area 1 (1-week biofilm) and measuring area 2 (2-weeks biofilm). The plaque samples were used for quantitative microbial analyses using PCR of 20 oral bacteria and Candida Albicans (ADD Clinident, The Netherlands).

Result: The amount of Candida Albicans and the total amount of bacteria in the 1-week biofilm baseline was equal for all 3 test groups. The 1-week and 2-weeks biofilm, however, showed a significantly lower amount of Candida Albicans and total amount of bacteria in test group C compared to B. The total amount of bacteria in the 1-week biofilm was also significantly lower for test group C compared to A.

Conclusion: Lower amounts of Candida Albicans and bacteria were found on dentures that were stored in water with a cleansing tablet compared to storage in water or dry.

Division: IADR/LAR General Session
Meeting: 2012 IADR/LAR General Session (Iguaçu Falls, Brazil)
Location: Iguaçu Falls, Brazil
Year: 2012
Final Presentation ID: 1096

Authors

- Duyck Joke (Catholic University of Leuven, Leuven, N/A, Belgium)
- Muller Peter (Katholieke Universiteit Leuven, 3000 Leuven, N/A, Belgium)
- Teughels Wim (Catholic University of Leuven, Leuven, N/A, Belgium)

SESSION INFORMATION
Poster Session
Oral Health of Older People
06/21/2012

The Effect of Overnight Storage on Removable Denture Biofilm

J Dent. 2013 Dec;41(12):1281-9. doi: 10.1016/j.jdent.2013.08.002. Epub 2013 Aug 13.

Overnight storage of removable dentures in alkaline peroxide-based tablets affects biofilm mass and composition.

Duyck J[1], Vandamme K, Muller P, Teughels W.

Author information

Abstract

BACKGROUND: Clinical guidelines for denture care are available, but evidence for optimal nocturnal storage is scarce. The aim of the study was to compare the role of the overnight storage state on plaque growth and composition on acrylic removable dentures.

METHODS: In a parallel-group randomized controlled trial of 51 institutionalized participants, 3 denture overnight preservation methods were considered: (i) in water, (ii) dry or (iii) in water with added alkaline peroxide-based cleansing tablet. Biofilm samples were taken on day 7 (developing biofilm - dBF) and day 14 (maturing biofilm - mBF) from a mechanically uncleaned, standardized region, situated distally to the second lower premolars. Total and individual levels of selected perio-pathogenic and commensal species (n=20), and of Candida albicans were calculated by PCR. Differences between storage conditions (water/dry/tablet) and between the samples (dBF/mBF) were assessed by means of unpaired and paired t-tests respectively, with α=5%.

RESULTS: Overnight denture storage with cleansing tablet significantly decreased the total bacterial level of dBF and mBF up to 13.8%. Fn, Ec, Cs, Sc, Ao and Vp counts were particularly affected by tablet care. Significant lower amounts of Candida albicans for tablet storage compared to water preservation were recorded in dBF and mBF (-69.3 ± 3.8% and -75.9 ± 3.2% respectively). The mass and pathogenicity of dBF and mBF was equal, irrespective of the overnight storage intervention.

CONCLUSIONS: The use of cleansing tablets for acrylic removable denture overnight storage reduces denture biofilm mass and pathogenicity compared to dry and water preservation, and may contribute to the overall systemic health.

CLINICAL SIGNIFICANCE: Evidence-based clinical guidelines for overnight storage of removable acrylic dentures are lacking. The findings of this study indicate that alkaline peroxide-based cleansing tablets decrease bacterial and Candida levels in denture biofilms in case of poor oral hygiene. This provides evidence for a clinical guideline to minimize microbial load of dentures, thereby reducing associated systemic health risks.

Copyright © 2013 The Authors. Published by Elsevier Ltd.. All rights reserved.

KEYWORDS: Acrylic removable denture; Candida albicans; Clinical trial; Microbiology; Nocturnal storage; Randomized
