# Supplementary material for: Reporting inconsistency between published conference abstracts and article abstracts of randomised controlled trials in prosthodontics presented at IADR general sessions
Source: PeerJ. 2023 May 5;11:e15303. doi: 10.7717/peerj.15303 (PMC10166077; doi:10.7717/peerj.15303)
Supplement: Supplemental Information 4 [file peerj-11-15303-s004.pdf]

2013

18-month Clinical Evaluation of New Etch-and-Rinse Adhesive in Cervical Lesions

18-month Clinical Evaluation of New Etch-and-Rinse Adhesive in Cervical Lesions

Objective: An 18-month randomized, controlled prospective study evaluated, in an intra-individual comparison, the clinical performance of two simplified etch-and-rinse adhesives in non-carious cervical lesions [NCCL]
Method: Thirty-five patients, with at least two similar sized NCCL participated in this study. After sample size calculation, 70 restorations were placed, according to one of the following groups: Adper Single Bond 2 (SB/3MESPE) and Ambar (AM/FGM). The restorations were placed incrementally using a composite resin (Opallis/FGM). The restorations were evaluated at baseline and after 6 and 18 months according to the FDI criteria (Hickel et al., J Adhes Dent 2007). The differences in the ratings of the two materials after 6 and 18 months were tested with Fisher exact´s test (alfa=0.05), and the performance of the each materials at baseline and after 6 and 18 months was evaluated by Wilcoxon test (alfa=0.05).

Result: All patients attended the 18-month recall. No significant differences were observed between the materials for any criteria evaluated. Only four restorations (two from each material) were lost after 18 months. Thus, the retention rates of both materials at 18 months were 94.2% (95% CI 81 - 98%). Nine restorations (4 for Ambar and 5 for Adper Single Bond 2) showed descoloration marginal classified as B or C, and it was possible to solve this problem with a new polishing procedure.

Conclusion: Both adhesive systems showed acceptable clinical retention rates and fulfilled the ADA seal for full acceptance.

Division: IADR/AADR/CADR General Session
Meeting: 2013 IADR/AADR/CADR General Session (Seattle, Washington)
Location: Seattle, Washington
Year: 2013
Final Presentation ID: 596

Authors

- Loguercio, Alessandro Dourado (Universidade Estadual de Ponta Grossa, Ponta Grossa, PR, , Brazil)
- Ferri, Letícia (Universidade Estadual De Ponta Grossa, Ponta Grossa, N/A, Brazil)
- Costa, Thays Rf (Universidade Estadual de Ponta Grossa, Ponta Grossa - Paraná, N/A, Brazil)
- Reis, Alessandra (Universidade Estadual de Ponta Grossa, Ponta Grossa, PR, , Brazil)

Loguercio, Alessandro Dourado (Universidade Estadual de Ponta Grossa, Ponta Grossa, PR, , Brazil)
Ferri, Letícia (Universidade Estadual De Ponta Grossa, Ponta Grossa, N/A, Brazil)
Costa, Thays Rf (Universidade Estadual de Ponta Grossa, Ponta Grossa - Paraná, N/A, Brazil)
Reis, Alessandra (Universidade Estadual de Ponta Grossa, Ponta Grossa, PR, , Brazil)

SESSION INFORMATION
Poster Session
Clinical Trials II
03/21/2013

18-month Clinical Evaluation of New Etch-and-Rinse Adhesive in Cervical Lesions

Am J Dent. 2014 Dec;27(6):312-7.

Eighteen-month randomized clinical trial on the performance of two etch-and-rinse adhesives in non-carious cervical lesions.

da Costa TR, Ferri LD, Loguercio AD, Reis A.

Abstract

PURPOSE: An 18-month randomized, controlled prospective study evaluated, in an intra-individual comparison, the clinical performance of two-step etch-and-rinse adhesives in non-carious cervical lesions (NCCL).

METHODS: 35 subjects, with at least two similar sized NCCL participated in this study. After sample size calculation, 70 restorations were placed, according to one of the following groups: Adper Single Bond 2 (SB) and Ambar (AM). The restorations were placed incrementally using a resin composite (Opallis). The restorations were evaluated at baseline and after 6 and 18 months according to the FDI criteria. The differences in the ratings of the two materials after 6 and 18 months were tested with Fisher's exact test ($\alpha = 0.05$), and the performance of the each material at baseline and after 6 and 18 months was evaluated by Wilcoxon test ($\alpha = 0.05$).

RESULTS: All subjects attended the 18-month recall. No significant differences were observed between the materials for any criteria evaluated. Only four restorations (two from each material) were lost after 18 months. Thus, the retention rates of both materials at 18 months were 94.2% (95% CI 81-98%). Nine restorations (four Ambar and five Adper Single Bond 2) showed marginal discoloration which was solved with a polishing procedure. Both adhesive systems showed acceptable clinical retention rates after 18 months.

PMID: 25707085

6-Months Clinical Comparison of Two Resin Composites on

6-Months Clinical Comparison of Two Resin Composites on Diastema Closure

Objective: To evaluate the esthetic, functional and biological clinical performance of two nano-hybrid resin composite systems used for anterior diastema closure at 6-months. Methods: Twenty-three patients with anterior midline or multi diastema problem were enrolled in this study. Nano-hybrid resin composite systems to be used on each patient were randomly selected. Thirty seven teeth of 10 patients were restored with Filtek-Z550 (3M/ESPE) in combination with Adper™ Single Bond 2 (3M/ESPE) etch&rinse adhesive; whereas 39 teeth of 13 patients were restored with Charisma-Diamond (Heraeus Kulzer) in combination with Gluma 2 Bond (Heraeus Kulzer) etch&rinse adhesive by two operators. Esthetic *(Surface Luster, Surface/Margin Staining, Color Match and Translucency, Esthetic Anatomical Form)*, Functional *(Fracture of Material and Retention, Marginal Adaptation, Patient's View)* and Biological (*Periodontal Response, Adjacent Mucosa, Oral&General Health*) properties of the restorations were evaluated at baseline and 6-months using FDI Criteria establishing a score-range of 1-5 (1-Clinically excellent/very good, 2-Clinically good, 3-Clinically sufficient/satisfactory, 4-Clinically unsatisfactory and 5-Clinically poor) by two independent examiners. The results were evaluated using the Pearson Chi-Square (p=0.05).

Results: At 6-months, 23 patients were evaluated. Two Filtek-Z550 restorations (5.4%) and 5 Charisma-Diamond (12.8%) restorations exhibited minor surface staining (Score 2). The surface luster of 3 Charisma-Diamond restorations were scored as 2. Three Filtek-Z550 restorations (8.1%) and 1 Charisma-Diamond restoration (2.6%) were scored as 2 with minor irregularities in marginal adaptation. However, there were no significant differences between two restorative materials for surface/marginal staining, surface luster and marginal adaptation (p>0.05). All the restorations in both groups were clinically excellent (Score 1) for the rest of the esthetic, functional and biological properties assessed.

Conclusions: Both nano-hybrid resin composite systems revealed esthetically, functionally, and biologically good clinical performance when used for diastema closure at 6-months.

Division: IADR/AADR/CADR General Session
Meeting: 2013 IADR/AADR/CADR General Session (Seattle, Washington)
Location: Seattle, Washington
Year: 2013
Final Presentation ID: 599

Authors

- Firat, Esra (Hacettepe University, Ankara, N/A, Turkey)
- Kutuk, Zeynep Bilge (Hacettepe University, Ankara, N/A, Turkey)
- Oztas, Sema Seval (Hacettepe University, Ankara, N/A, Turkey)
- Gurgan, Sevil (Hacettepe University, Ankara, N/A, Turkey)
- Yalcin Cakir, Filiz (Hacettepe University, Ankara, , Turkey)

Firat, Esra (Hacettepe University, Ankara, N/A, Turkey)
Kutuk, Zeynep Bilge (Hacettepe University, Ankara, N/A, Turkey)
Oztas, Sema Seval (Hacettepe University, Ankara, N/A, Turkey)
Gurgan, Sevil (Hacettepe University, Ankara, N/A, Turkey)
Yalcin Cakir, Filiz (Hacettepe University, Ankara, , Turkey)

SESSION INFORMATION
Poster Session
Clinical Trials II
03/21/2013

6-Months Clinical Comparison of Two Resin Composites on

Nigerian Journal of Clinical Practice

Abstract

Aims: This aim of this study is to evaluate and to compare the clinical performances of two nanohybrid composite resin systems used for diastema closure and tooth reshaping at 4 years.

Subjects and Methods: Twenty-three patients with midline or multidiastema problem were enrolled in this study. Nanohybrid resin composite systems to be used on each patient were randomly selected. Thirty seven teeth of 10 patients were restored with Filtek-Z550 (3M/ESPE) in combination with Adper™ Single Bond 2 (3M/ESPE) etch and rinse adhesive in Group 1 whereas 39 teeth of 13 patients were restored with Charisma-Diamond (Heraeus Kulzer) in combination with Gluma2 Bond (Heraeus Kulzer) etch and rinse adhesive in Group 2, by two operators. Esthetic, functional, and biological properties of the restorations were evaluated at baseline, 1, 2, 3, and 4 years using foreign direct investment criteria by two independent examiners. Statistical Analysis Used: The data were evaluated using Fisher's Chi-Square ($P = 0.05$).

Results: Fifty-eight restorations (19 patients) with a mean service time of 43.4 months were evaluated (recall rate 82.6%). One Filtek-Z550 and two Charisma-Diamond restorations were repaired due to partial fracture (Score 4). Survival rates of Group 1 and Group 2 were 96.3% and 93.5%, respectively (Kaplan–Meier) ($P > 0.05$). Qualitative deteriorations were observed within each group according to baseline regarding surface luster, surface/marginal staining, marginal adaptation, patient's view, and periodontal response ($P < 0.05$). However, there were no significant differences between two restorative materials for any of the criteria assessed ($P > 0.05$).

Conclusions: Both nanohybrid composite resin systems revealed esthetically, functionally, and biologically acceptable clinical performance when used for diastema closure and tooth reshaping at 4 years.

Keywords: Clinical performance, composite resin buildup, diastema closure

Anti-infective therapy of periimplantitis:Outcomes of a 12-month RCT

Anti-infective therapy of periimplantitis:Outcomes of a 12-month RCT

Objective: to compare the adjunctive effects in the non-surgical treatment of periimplantitis with either local drug delivery (LDD) or photodynamic therapy (PDT).
Method: Forty subjects with initial periimplantitis, i.e. pocket probing depths (PPD) 4–6 mm with bleeding on probing (BoP) and crestal bone loss 0.5-2 mm were randomly assigned to two treatment groups. After mechanical debridement, test implant sites (N=20) received adjunctive PDT, whereas minocycline microspheres were locally delivered at sites of control implants (N=20). At sites with residual BoP, treatment was repeated after 3, 6 and 9 months. The primary outcome variable was the change in the number of sites with BoP. Secondary outcome variables were changes in PPD, clinical attachment level (CAL), mucosal recession (REC) and changes in microbiological and host-derived parameters.

Result: After 12 months, the number of BoP-positive sites decreased statistically significantly ($p<0.05$) from baseline in the PDT (4.03 ± 1.66 to 1.74 ± 1.37) and LDD (4.41 ± 1.47 to 1.55 ± 1.26) group, respectively. A statistically significant ($p<0.05$) decrease in PPD from baseline was observed at PDT-treated sites up to 9 months (4.19 ± 0.55 mm to 3.89 ± 0.68 mm) and up to 12 months at LDD-treated sites (4.39 ± 0.77 mm to 3.83 ± 0.85 mm). The presence of *Porphyromonas gingivalis* and *Tannerella forsythia* decreased statistically significantly ($p<0.05$) from baseline to 9 months in the PDT group and up to 12 months in the LDD group. After 12 months, the concentration in the crevicular fluid of interleukin-1β decreased statistically significantly ($p<0.05$) from baseline in both groups. No statistically significant differences were observed between both groups after 12 months with respect to clinical, microbiological and host-derived parameters.

Conclusion: Non-surgical mechanical debridement with adjunctive use of PDT was equally effective as the adjunctive use of LDD in the treatment of initial periimplantitis up to 12 months.

Division: IADR/AADR/CADR General Session
Meeting: 2013 IADR/AADR/CADR General Session (Seattle, Washington)
Location: Seattle, Washington
Year: 2013
Final Presentation ID: 430

Authors

- Salvi Giovanni E. (University of Bern, Bern, N/A, Switzerland)
- Bassetti Mario (University of Bern, Bern, N/A, Switzerland)
- Schär Dorothee (University of Bern, Bern, N/A, Switzerland)
- Ramseier Christoph (University of Bern, Bern, N/A, Switzerland)
- Eick Sigrun (School of Dental Medicine, University of Bern, Bern, , Switzerland)
- Arweiler Nicole (University of Marburg, Marburg, , Germany)
- Sculean Anton (School of Dental Medicine, University of Bern, Bern, , Switzerland)

Salvi Giovanni E. (University of Bern, Bern, N/A, Switzerland)
Bassetti Mario (University of Bern, Bern, N/A, Switzerland)
Schär Dorothee (University of Bern, Bern, N/A, Switzerland)
Ramseier Christoph (University of Bern, Bern, N/A, Switzerland)
Eick Sigrun (School of Dental Medicine, University of Bern, Bern, , Switzerland)
Arweiler Nicole (University of Marburg, Marburg, , Germany)
Sculean Anton (School of Dental Medicine, University of Bern, Bern, , Switzerland)

SESSION INFORMATION
Oral Session
Keynote Address; Dental Biofilm and Implants
03/21/2013

Anti-infective therapy of periimplantitis:Outcomes of a 12-month RCT

Clin Oral Implants Res. 2013 Jan;24(1):104-10. doi: 10.1111/j.1600-0501.2012.02494.x. Epub 2012 May 9.

Anti-infective therapy of peri-implantitis with adjunctive local drug delivery or photodynamic therapy: six-month outcomes of a prospective randomized clinical trial.

Schär D[1], Ramseier CA, Eick S, Arweiler NB, Sculean A, Salvi GE.

⊕ Author information

Abstract

OBJECTIVE: To compare the adjunctive clinical effects in the non-surgical treatment of peri-implantitis with either local drug delivery (LDD) or photodynamic therapy (PDT).

MATERIAL AND METHODS: Forty subjects with initial peri-implantitis, i.e. pocket probing depths (PPD) 4-6 mm with concomitant bleeding on probing (BoP) and marginal bone loss ranging from 0.5 to 2 mm between delivery of the reconstruction and pre-screening appointment were randomly assigned to two treatment groups. All implants underwent mechanical debridement with titanium curettes, followed by a glycine-based powder airpolishing. Implants in the test group (n = 20) received adjunctive PDT, whereas minocycline microspheres were locally delivered into the peri-implant pockets of control implants (n = 20). At sites with residual BoP, treatment was repeated after 3 and 6 months. The primary outcome variable was the change in the number of sites with BoP. Secondary outcome variables were changes in PPD, in clinical attachment level (CAL), and in mucosal recession (REC).

RESULTS: After 3 months, implants of both groups yielded a statistically significant reduction ($P < 0.0001$) in the number of BoP-positive sites compared with baseline (LDD: from 4.41 ± 1.47 to 2.20 ± 1.28, PDT: from 4.03 ± 1.66 to 2.26 ± 1.28). After 6 months, complete resolution of mucosal inflammation was obtained in 15% of the implants in the control group and in 30% of the implants in the test group ($P = 0.16$). After 3 months, changes in PPD, REC, and modified Plaque Index (mPlI) were statistically significantly different from baseline ($P < 0.05$). No statistically significant changes ($P > 0.05$) occurred between 3 and 6 months. CAL measurements did not yield statistically significant changes ($P > 0.05$) in both groups during the 6-month observation time. Between-group comparisons revealed no statistically significant differences ($P > 0.05$) at baseline, 3 and 6 months with the exception of the mPlI after 6 months.

CONCLUSIONS: In cases of initial peri-implantitis, non-surgical mechanical debridement with adjunctive use of PDT is equally effective in the reduction of mucosal inflammation as with the adjunctive use of minocycline microspheres up to 6 months. Adjunctive PDT may represent an alternative treatment modality in the non-surgical management of initial peri-implantitis. Complete resolution of inflammation, however, was not routinely achieved with either of the adjunctive therapies.

© 2012 John Wiley & Sons A/S.

PMID: 22568744 DOI: 10.1111/j.1600-0501.2012.02494.x

Bacterial Adhesion on Titanium and Zirconia: DNA Checkerboard Clinical Trial

Bacterial Adhesion on Titanium and Zirconia: DNA Checkerboard Clinical Trial

Objective: Bacterial adhesion and colonization of dental implants abutments may be influenced by the surface and composition of the material used in their fabrication. The aim of this *in vivo* investigation was to evaluate bacterial biofilm formation on the surface of machined titanium 6Al-4V (MPT), cast titanium 6Al-4V (CPT), and machined zirconia (MZc) abutments. Methods: Six health subjects were enrolled in this randomized *crossover* clinical investigation. The study was conducted in three phases according to the abutment surface evaluated. Each subject wore an removable oral splint containing 4 discs of the same tested substrate, 2 located in the anterior and 2 in the posterior region of the mouth, totalling 12 specimens per subject. Participants were asked to use the intraoral splint during 24 h consecutively. The DNA Checkerboard hybridization method was used to identify and quantify bacteria in the biofilm formed on the abutment substrates, based on 38 target species selected. The results were compared with Kruskal-Wallis and Dunn's post-hoc tests. *Results:* Both pathogens and non-pathogens species were found on the surfaces of the three substrates. Overall, there was a significant difference in bacterial indices between the three groups ($p<0.01$). Thirty species were significantly different when compared individually between-groups ($p<0.01$). CPT presented the higher mean index, followed by MPT and MZc. The highest indices ($x10^5$, ±SEM) were recorded for *P. endodontalis* (2.11 ±0.45) and *S. pasteuri* (1.84 ±0.47) in MPT group, *S. mitis* (3.17 ±0.68) and *S. mutans* (2.42 ±0.47) in MZc, and *A. actynomicetemcomitans a* (4.98 ±0.11) and *P. melaninogenica* (4.66 ±0.28) in CPT. No difference in bacterial adhesion was found between the anterior and posterior locations of the discs in the mouth. *Conclusions:* Changes in the physicochemical properties of the abutment materials caused by casting procedures may have higher impact on bacterial adhesion than their chemical composition.

Division: IADR/AADR/CADR General Session
Meeting: 2013 IADR/AADR/CADR General Session (Seattle, Washington)
Location: Seattle, Washington
Year: 2013
Final Presentation ID: 3560

Authors

- Nascimento, Cassio (University of São Paulo, Ribeirão Preto, N/A, Brazil)
- Pita, Murillo (University of São Paulo, São José do Rio Preto, N/A, Brazil)
- Calefi, Paulo (University of São Paulo, Ribeirão Preto, , Brazil)
- Albuquerque, Rubens (McGill University, Montreal, QC, Canada)
- Pedrazzi, Vincius (University of São Paulo, Ribeirão Preto, N/A, Brazil)
- Ribeiro, Ricardo Faria (University of São Paulo, Ribeirão Preto, N/A, Brazil)

Nascimento, Cassio (University of São Paulo, Ribeirão Preto, N/A, Brazil)
Pita, Murillo (University of São Paulo, São José do Rio Preto, N/A, Brazil)
Calefi, Paulo (University of São Paulo, Ribeirão Preto, , Brazil)
Albuquerque, Rubens (McGill University, Montreal, QC, Canada)
Pedrazzi, Vincius (University of São Paulo, Ribeirão Preto, N/A, Brazil)
Ribeiro, Ricardo Faria (University of São Paulo, Ribeirão Preto, N/A, Brazil)

SESSION INFORMATION
Poster Session
Adhesion - Leakage/Margin Assessments II
03/23/2013

Clin Oral Implants Res. 2014 Mar;25(3):337-343. doi: 10.1111/clr.12093. Epub 2013 Jan 14.

Bacterial adhesion on the titanium and zirconia abutment surfaces.

Nascimento CD[1], Pita MS[1], Fernandes FHNC[1], Pedrazzi V[1], de Albuquerque Junior RF[1], Ribeiro RF[1].

Author information

1 Department of Dental Materials and Prosthodontics, Faculty of Dentistry of Ribeirão Preto, University of São Paulo, São Paulo, Brazil.

Abstract

OBJECTIVE: Microorganisms harboring the oral cavity, mainly those related to periodontal diseases, are the most potential etiologic factor of failure in long-term implant treatment. The material used for abutment components may influence the adhesion and colonization of microbial species. The aim of this in vivo investigation was to evaluate the biofilm formation on machined (MPT) or cast titanium (CPT) and zirconia abutments (Zc).

METHODS: Six healthy subjects were enrolled in this randomized crossover clinical investigation. The study was conducted in three phases according to abutment surface evaluated. Each subject used an individual oral splint containing four disks of the same tested substrate, two located in the anterior and two in the posterior region, totalizing 12 specimens for subject. Participants were asked to use the removable intraoral splint during 24 h. DNA checkerboard hybridization method was used to identify and quantify 38 bacterial species colonizing formed biofilm on the abutment substrates.

RESULTS: Pathogens and non-pathogens species were found colonizing the three substrates surfaces. Fusobacterium nucleatum, Neisseria mucosa, Porphyromonas aeruginosa, Peptostreptococcus anaerobios, Staphylococcus aureus, Streptococcus gordonii, Streptococcus parasanguinis, and Tanerella forsythia were the only species with no significant differences over the tested materials ($P > 0.05$). All the other target species presented significant differences sought by Friedman test ($P < 0.0001$).

CONCLUSIONS: There was a significant difference in the total bacterial count between the three groups. CPT presented the higher mean counts, followed by MPT and Zc. CPT group also showed a higher mean incidence of species than MPT and Zc. The anterior or posterior region of disks placement did not show significant differences in relation to bacterial adhesion.

© 2013 John Wiley & Sons A/S. Published by Blackwell Publishing Ltd.

KEYWORDS: biomaterials; clinical assessment; clinical research; diagnosis; microbiology; prosthodontics

PMID: 23316996 DOI: 10.1111/clr.12093

Contingent Electrical Stimulation Reduces Jaw Muscle Activity, not Pain Perception

Contingent Electrical Stimulation Reduces Jaw Muscle Activity, not Pain Perception

Objective: To investigate the effect of contingent electrical stimulation (CES) on pain intensity (PI), pressure pain threshold (PPT) and electromyography (EMG) activity in self-reported bruxers with myofascial pain.
Method: 15 patients with myofascial pain according to RDC/TMD and self-reported sleep bruxism, according to the International Classification of Sleep Disorders, participated in this study. Patients were randomly allocated into two groups: CES (n=7; 37.3±8.9 years) and control (n=8; 31.9±12.3 years). The study was performed in three phases for 4 weeks, as follows: baseline (T1) 1 week without CES; treatment (T2) 2 weeks period with CES activated (CES group) or not (control group); and follow-up (T3) 1 week without CES. CES was individually adjusted for each patient to a clear and distinct, but nonpainful electrical stimulation. EMG during sleep was recorded with a portable EMG device (Grindcare®, Medotech A/S) in all phases. After each phase, PI was recorded with a 0-10cm Visual Analogue Scale. PPTs were measured on masseter (PPTm) and anterior temporalis (PPTat). ANOVA models were used for comparisons, considering a 5% level of significance.

Result: The average EMG activity at T1 was 33.5±23.5 and 24.8±14.8 grinds/hour respectively for the CES and control groups. EMG activity was significant different between phases (p=0.003) with lower values during T2 and T3 only in the CES group. The CES group patients demonstrated 35% lower EMG activity in T2 and 38.4% in T3. There were no significant differences between groups in PI and PPT during the different phases.

Conclusion: CES reduced EMG activity related to tooth-clenching/grinding in myofascial pain patients, however, this decrease was not associated with changes in self-reported pain or mechanical sensitivity. These findings suggest that other factors must be involved in the pain experience of myofascial TMD patients, including the contribution of the central nervous system nociceptive processing and psychological variables.

Division: IADR/AADR/CADR General Session
Meeting: 2013 IADR/AADR/CADR General Session (Seattle, Washington)
Location: Seattle, Washington
Year: 2013
Final Presentation ID: 3910

Conti, Paulo Cesar Rodrigues (Bauru School of Dentistry - University of São Paulo, Bauru, N/A, Brazil)
Bonjardim, Leonardo Rigoldi (Universidade Federal De Sergipe, Aracaju, N/A, Brazil)
Stuginski-barbosa, Juliana (Bauru School of Dentistry, Bauru, N/A, Brazil)
Svensson, Peter (Aarhus University, Aarhus, N/A, Denmark)

Authors

- Conti, Paulo Cesar Rodrigues (Bauru School of Dentistry - University of São Paulo, Bauru, N/A, Brazil)
- Bonjardim, Leonardo Rigoldi (Universidade Federal De Sergipe, Aracaju, N/A, Brazil)
- Stuginski-barbosa, Juliana (Bauru School of Dentistry, Bauru, N/A, Brazil)
- Svensson, Peter (Aarhus University, Aarhus, N/A, Denmark)

SESSION INFORMATION
Poster Session
Bruxism and Jaw Muscle Activity
03/23/2013

Contingent Electrical Stimulation Reduces Jaw Muscle Activity, not Pain Perception

Oral Surg Oral Med Oral Pathol Oral Radiol. 2014 Jan;117(1):45-52. doi: 10.1016/j.oooo.2013.08.015. Epub 2013 Oct 31.

Contingent electrical stimulation inhibits jaw muscle activity during sleep but not pain intensity or masticatory muscle pressure pain threshold in self-reported bruxers: a pilot study.

Conti PC[1], Stuginski-Barbosa J[2], Bonjardim LR[3], Soares S[4], Svensson P[5].

Author information

1 Professor, Department of Prosthodontics, Bauru School of Dentistry, University of São Paulo, Bauru, São Paulo, Brazil. Electronic address: pcconti@fob.usp.br.

2 Doctorate Student, Department of Prosthodontics, Bauru School of Dentistry, University of São Paulo, Bauru, São Paulo, Brazil.

3 Professor, Departament of Biological Sciences, Bauru School of Dentistry, University of São Paulo, Bauru, São Paulo, Brazil.

4 Professor, Department of Prosthodontics, Bauru School of Dentistry, University of São Paulo, Bauru, São Paulo, Brazil.

5 Professor, Section of Clinical Oral Physiology, Department of Dentistry, Aarhus University, Aarhus, Denmark; Center for Functionally Integrative Neuroscience (CFIN), MindLab, Aarhus University Hospital, Aarhus, Denmark.

Abstract

OBJECTIVE: This study investigated the effect of contingent electrical stimulation (CES) on present pain intensity (PI), pressure pain threshold (PPT), and electromyographic events per hour of sleep (EMG/h) on probable bruxers with masticatory myofascial pain.

STUDY DESIGN: The study enrolled 15 probable bruxers with masticatory myofascial pain in 3 phases: (1) baseline EMG/h recording, (2) biofeedback treatment using a CES paradigm (active group, n = 7) or inactive device (control group, n = 8), and (3) posttreatment EMG/h recording. PI and PPT were assessed after each phase. Analysis of variance models were used to compare results at a 5% significance level.

RESULTS: Patients in the active group had 35% lower EMG/h in P2 and 38.4% lower EMG/h in P3, when compared with baseline. There were no differences in PI or PPT levels at any phase.

CONCLUSIONS: CES could reduce EMG activity associated with sleep bruxism in patients with masticatory myofascial pain but did not influence perceived pain.

Cross-over clinical trial of biofilm formation influenced by adhesive usage

Cross-over clinical trial of biofilm formation influenced by adhesive usage

Objectives: The objective of this *in vivo* study was to investigate the influence of adhesive usage (Corega cream; Glaxo Smith Kline, U.K.) on biofilm formation on the internal surfaces of complete dentures.

Methods: Twenty edentulous patients received a new set of complete dentures. After the intraoral adjustments and adaptation period, the participants were enrolled in the trial and randomly assigned to 2 protocols: protocol 1 – adhesive usage during the first 15 days, followed by no use of adhesive over the next 15 days; protocol 2- no use of adhesive during the first 15 days, followed by adhesive usage over the next 15 days. After each period of 15 days, the internal surfaces of the maxillary and mandibular complete dentures were stained and photographed with standard distance and 45° inclination. The photographs were processed and the areas (total internal surface and surface stained with biofilm) quantified (software Image Tool 3.00). The percentage of the biofilm was calculated by the ratio between the biofilm area multiplied by 100 and the total area of the internal surface of the complete dentures. Data were analyzed by nonparametric paired sample Wilcoxon test ($\alpha < .05$).

Results: Similar biofilm formation (%) was found with or without adhesive usage for maxillary ($p=.225$) and mandibular complete dentures ($p=.433$).

Conclusions: Adhesive usage did not influence the biofilm formation on the internal surfaces of maxillary and mandibular complete dentures during the 15-day trial period.

Division: IADR/AADR/CADR General Session
Meeting: 2013 IADR/AADR/CADR General Session (Seattle, Washington)
Location: Seattle, Washington
Year: 2013
Final Presentation ID: 1048

Authors

- Pero, Ana Carolina (Universidade Estadual Paulista Júlio de Mesquita Filho, Araraquara, N/A, Brazil)
- Leite, Andressa Rosa Perin (Universidade Estadual Paulista Júlio de Mesquita Filho, Araraquara, N/A, Brazil)
- Marin, Danny Omar Mendoza (Universidade Estadual Paulista Júlio de Mesquita Filho, Araraquara, N/A, Brazil)
- Paleari, André Gustavo (Universidade Estadual Paulista Júlio de Mesquita Filho, Araraquara, N/A, Brazil)
- Rodriguez, Larissa Santana (Universidade Estadual Paulista Júlio de Mesquita Filho, Araraquara, N/A, Brazil)
- Giro, Gabriela (Universidade Estadual Paulista Júlio de Mesquita Filho, Araraquara, N/A, Brazil)
- Compagnoni, Marco Antonio (Universidade Estadual Paulista Júlio de Mesquita Filho, Araraquara, N/A, Brazil)

Pero, Ana Carolina (Universidade Estadual Paulista Júlio de Mesquita Filho, Araraquara, N/A, Brazil)
Leite, Andressa Rosa Perin (Universidade Estadual Paulista Júlio de Mesquita Filho, Araraquara, N/A, Brazil)
Marin, Danny Omar Mendoza (Universidade Estadual Paulista Júlio de Mesquita Filho, Araraquara, N/A, Brazil)
Paleari, André Gustavo (Universidade Estadual Paulista Júlio de Mesquita Filho, Araraquara, N/A, Brazil)
Rodriguez, Larissa Santana (Universidade Estadual Paulista Júlio de Mesquita Filho, Araraquara, N/A, Brazil)
Giro, Gabriela (Universidade Estadual Paulista Júlio de Mesquita Filho, Araraquara, N/A, Brazil)
Compagnoni, Marco Antonio (Universidade Estadual Paulista Júlio de Mesquita Filho, Araraquara, N/A, Brazil)

SESSION INFORMATION
Poster Session
Clinical Studies, Edentate Patients
03/21/2013

Cross-over clinical trial of biofilm formation influenced by adhesive usage

The Journal of Prosthetic Dentistry

Volume 112, Issue 2, August 2014, Pages 349-356

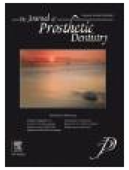

Crossover clinical trial of the influence of the use of adhesive on biofilm formation

Andressa R.P. Leite DDS [a], Danny O. Mendoza-Marin DDS [b], André G. Paleari DDS, PhD [c], Larissa S. Rodriguez DDS, MSc [d], Andréia A. Roccia [e], Vivian B. Policastro [f], Marco A. Compagnoni DDS, PhD [g], Raphael F. de Souza DDS, PhD [h], Ana C. Pero DDS, PhD [i]

⊞ **Show more**

https://doi.org/10.1016/j.prosdent.2013.11.003 Get rights and content

Statement of problem

Contrasting results have been reported regarding the influence of the use of adhesive on biofilm formation.

Purpose

The purpose of this study was to evaluate the influence of the use of adhesive on the formation of biofilm on the internal surface of complete dentures and the palatal mucosa of denture wearers.

Material and methods

Thirty participants with well-fitting complete dentures were randomly divided according to the experimental design: protocol 1, adhesive use during the first 15 days, followed by no use of adhesive over the next 15 days; protocol 2, no use of adhesives during the first 15 days, followed by adhesive use over the next 15 days. After each period, material from the mucosa and intaglio of the maxillary dentures was collected. Replicate aliquots were plated onto Petri dishes containing selective media for *Candida* spp, *Streptococcus mutans*, and a nonselective culture medium. Colony-forming units were expressed as log (CFU+1)/mL. In addition, the internal surfaces of the maxillary and mandibular complete dentures were stained and photographed. From the photographs, the total internal surface and the surface stained with biofilm were quantified (software ImageTool 3.00), and the percentage of the biofilm-covered area (%) on the maxillary and mandibular dentures was calculated and compared with 2-way ANOVA. For the nonselective culture medium, data were compared with the paired-sample *t* test, and the Wilcoxon signed rank test was performed to compare the colony counts of *Candida* spp and *Streptococcus mutans* (α=.05).

Results

Similar colony counts were found with or without the use of adhesive for the mucosa and internal surfaces of maxillary dentures, irrespective of the culture medium (P>.05). The area of dentures covered with biofilm was influenced by the use of adhesive (P=.025), regardless of the type of denture (P=.121).

Conclusions

The use of adhesive did not alter the colony counts of microorganisms from the palatal mucosa and maxillary dentures of complete denture wearers during the 15-day period, but it did influence the area covered with biofilm on the internal surfaces of the complete dentures.

Denture Adhesive Influence on Masticatory Function of Complete Denture Wearers

Denture Adhesive Influence on Masticatory Function of Complete Denture Wearers

Objectives: This study aimed to determine the effect of two denture adhesives (DA) on mastication of complete denture wearers.
Methods: A cross-sectional clinical trial was conducted and 20 volunteers (mean age 67.4 ± 8.3 years) with upper and lower total edentulism were selected. All subjects have received new complete dentures, which were adjusted to bilateral balanced occlusion. After 3 months of adaptation to new dentures, masticatory performance was assessed in three situations: *(1)* without DA; *(2)* using cream DA; and *(3)* using strip DA. The sequence was randomly established and adhesives were applied on lower denture. Masticatory performance was evaluated by chewing an artificial test material Optocal using a sieving method and it was defined as the median particle size (X50). Data were submitted to exploratory analyzes and showed normal distribution. PROC GLM procedure of the SAS statistical program was applied and multiple comparisons were accomplished by the Tukey-Kramer test with a significance level of 5%.

Results: Regardless of the adhesive type ($p = 0.072$), consistent improvement was observed in masticatory performance when adhesives were used ($p < 0.001$).

Conclusions: The results of this study provide evidence that the use of DA can enhance mastication of complete denture wearers.

Division: IADR/AADR/CADR General Session
Meeting: 2013 IADR/AADR/CADR General Session (Seattle, Washington)
Location: Seattle, Washington
Year: 2013
Final Presentation ID: 3525

Authors

- Goncalves Thais Marques Simek Vega (UNICAMP, Campinas, N/A, Brazil)
- Viu Flávia (UNICAMP, Piracicaba, N/A, Brazil)
- Goncalves Leticia Machado (UNICAMP, Piracicaba-SP, N/A, Brazil)
- Rodrigues Garcia Renata (UNICAMP, Piracicaba, SP, N/A, Brazil)

Goncalves Thais Marques Simek Vega (UNICAMP, Campinas, N/A, Brazil)
Viu Flávia (UNICAMP, Piracicaba, N/A, Brazil)
Goncalves Leticia Machado (UNICAMP, Piracicaba-SP, N/A, Brazil)
Rodrigues Garcia Renata (UNICAMP, Piracicaba, SP, N/A, Brazil)

SESSION INFORMATION
Poster Session
Occlusion & Mastication
03/23/2013

Denture Adhesive Influence on Masticatory Function of Complete Denture Wearers

Int J Prosthodont. 2014 Mar-Apr;27(2):140-6. doi: 10.11607/ijp.3674.

Denture adhesives improve mastication in denture wearers.

Gonçalves TM, Viu FC, Gonçalves LM, Garcia RC.

Abstract

PURPOSE: This clinical trial evaluated the influence of denture adhesive (DA) use on masticatory function in denture wearers according to their denture-bearing ridge status.

MATERIALS AND METHODS: Thirty edentulous subjects, wearing new well-fitting dentures, were classified as having either a normal or resorbed ridge. Mastication was evaluated in patients who completed chewing tests with and without two DA substances (cream or strips), which were randomly assigned. A chewing test with a sieve method analyzed masticatory performance. A kinesiographic device evaluated chewing cycle, and a visual analog scale measured masticatory ability. Data were submitted to Mauchly's sphericity test, and PROC MIXED procedures were conducted on repeated measures. Tukey-Kramer tests performed appropriate statistical comparisons ($P \leq .05$).

RESULTS: DA use increased masticatory performance and ability in patients with both ridge types ($P < .05$). Subjects with resorbed ridges showed the best masticatory performance ($P < .001$) and lowest chewing cycle time ($P < .001$) with DA cream, followed by DA strips and the nonadhesive trial. For normal ridge subjects, decreases in ×50 values were only significant with DA use ($P < .05$), regardless of DA type. The denture-bearing ridge status alone did not alter masticatory function in any of the parameters evaluated.

CONCLUSION: DAs improve mastication by shortening the chewing cycle and by enhancing chewing ability and performance.

PMID: 24596911

Effect of adhesive usage on satisfaction of complete denture wearers

Effect of adhesive usage on satisfaction of complete denture wearers

Objectives: The objective of this study was to evaluate the effect of a denture adhesive (DA) (Corega cream; Glaxo Smith Kline, U.K.) in patients' satisfaction with new well-fitting complete dentures by a cross-over clinical trial.

Methods: Forty-four subjects were randomized and assigned to 2 protocols with alternating periods and sequence of 15 days, as follow: *protocol 1*- DA application during the first 15 days, followed by the absence of DA over the next 15 days; *protocol 2*-absence of DA during the first 15 days, followed by application of DA over the next 15 days. The Brazilian version of a questionnaire for satisfaction with complete dentures was used to evaluate subjectively patients' satisfaction with and without DA after each period of 15 days. Patients' satisfaction graded of their complete dentures by using scores from 0 to 2 (0 = unsatisfactory, 1 = regular, 2 = good). Data were analyzed using the nonparametric "paired sample Wilcoxon test" ($\alpha = .05$).

Results: The use of DA improved the overall satisfaction of complete denture wearers ($p<.05$). Significant differences for general satisfaction (96.7%) and chewing (83.3%) were observed with the use of DA, which also improved significantly the patients' satisfaction with respect to retention (83.3%) and comfort (76.7%) of the mandibular denture. However, there was no significant improvement in patients' satisfaction with maxillary denture ($p> .05$). Furthermore, aesthetics and phonetics were not influenced by the DA.

Conclusions: Denture adhesive use improved the degree of satisfaction of complete denture wearers, influenced mainly by the increase in the general satisfaction, chewing, retention and comfort of mandibular denture.

Division: IADR/AADR/CADR General Session
Meeting: 2013 IADR/AADR/CADR General Session (Seattle, Washington)
Location: Seattle, Washington
Year: 2013
Final Presentation ID: 1040

Marin Danny Omar Mendoza (Universidade Estadual Paulista Júlio de Mesquita Filho, Araraquara, N/A, Brazil)
Leite Andressa Rosa Perin (Universidade Estadual Paulista Júlio de Mesquita Filho, Araraquara, N/A, Brazil)
Paleari André Gustavo (Universidade Estadual Paulista Júlio de Mesquita Filho, Araraquara, N/A, Brazil)
Rodriguez Larissa Santana (Universidade Estadual Paulista Júlio de Mesquita Filho, Araraquara, N/A, Brazil)
Oliveira Júnior Norberto Martins (Universidade Estadual Paulista Júlio de Mesquita Filho, Araraquara, N/A, Brazil)
Pero Ana Carolina (Universidade Estadual Paulista Júlio de Mesquita Filho, Araraquara, N/A, Brazil)
Compagnoni Marco Antonio (Universidade Estadual Paulista Júlio de Mesquita Filho, Araraquara, N/A, Brazil)

Authors

- Marin Danny Omar Mendoza (Universidade Estadual Paulista Júlio de Mesquita Filho, Araraquara, N/A, Brazil)
- Leite Andressa Rosa Perin (Universidade Estadual Paulista Júlio de Mesquita Filho, Araraquara, N/A, Brazil)
- Paleari André Gustavo (Universidade Estadual Paulista Júlio de Mesquita Filho, Araraquara, N/A, Brazil)
- Rodriguez Larissa Santana (Universidade Estadual Paulista Júlio de Mesquita Filho, Araraquara, N/A, Brazil)
- Oliveira Júnior Norberto Martins (Universidade Estadual Paulista Júlio de Mesquita Filho, Araraquara, N/A, Brazil)
- Pero Ana Carolina (Universidade Estadual Paulista Júlio de Mesquita Filho, Araraquara, N/A, Brazil)
- Compagnoni Marco Antonio (Universidade Estadual Paulista Júlio de Mesquita Filho, Araraquara, N/A, Brazil)

SESSION INFORMATION
Poster Session
Clinical Studies, Edentate Patients
03/21/2013

Effect of adhesive usage on satisfaction of complete denture wearers

Braz Dent J. 2014 Sep-Oct;25(5):391-8. doi: 10.1590/0103-6440201302409.

Effect of a denture adhesive on the satisfaction and kinesiographic parameters of complete denture wearers: a cross-over randomized clinical trial.

Marin DO[1], Leite AR[1], Paleari AG[1], Rodriguez LS[1], Oliveira Junior NM[1], Pero AC[1], Compagnoni MA[1].

Author information

Abstract

The aim of the present study was to assess the effect of a denture adhesive (DA) on patient satisfaction and kinesiographic parameters of complete denture wearers by a cross-over study. Fifty edentulous patients received a set of new complete dentures. After an adaptation period, the participants were enrolled in the trial and randomized to receive a sequence of treatment protocols: Protocol 1- DA use during the first 15 days, followed by no DA for the next 15 days; Protocol 2- no DA during the first 15 days, followed by use of DA for the next 15 days. Outcomes were assessed after 15 days of each sequence of treatment. A questionnaire was used to assess the patients' satisfaction. A kinesiograph was used to record mandible movements and patterns of maxillary complete denture movement during chewing. The Wilcoxon test ($\alpha=0.05$) and a paired sample t-test ($\alpha=0.05$) were used to compare satisfaction levels and kinesiographic data, respectively. Use of DA improved the overall level of patient satisfaction ($p<0.001$). The kinesiographic recordings revealed a significant increase (1.7 mm) in vertical mandible movements ($p<0.001$) during chewing and a lower (0.3 mm) vertical intrusion of the maxillary complete dentures ($p=0.002$) during chewing after using the DA. Use of DA in complete denture wearers improved the patients' satisfaction and altered mandible movements, with increases in vertical movements during chewing and less intrusion of maxillary complete dentures.

PMID: 25517773 DOI: 10.1590/0103-6440201302409

Effect of denture adhesive on kinesiographic parameters in complete denture

Effect of denture adhesive on kinesiographic parameters in complete denture

Objectives: The purpose of this cross over clinical trial was to evaluate the effect of a denture adhesive (DA) (Corega cream; Glaxo Smith Kline, U.K.) on the kinesiographic parameters of new well-fitting complete dentures wearers.
Methods: Edentulous patients (n= 44) received one set of new complete dentures. After adaptation period (30 days), the subjects were randomized and assigned to 2 protocols, as follow: *protocol 1*- DA use during the first 15 days, followed by the no use of DA over the next 15 days; *protocol 2*- no use of DA during the first 15 days, followed by use of DA over the next 15 days. The kinesiograph instrument K7-I (Myotronics Research Inc., Seattle, WA) was used to record opening and closure movement limits, freeway space, chewing cycle and pattern of maxillary complete denture movement during chewing after each period of 15 days. Data were analyzed using the parametric "paired sample t-Student" ($\alpha = .05$).

Results: There were no significant differences for opening and closure movement limits and freeway space with or without DA use. However, DA use produced an increase (1.7 mm) on mandibular vertical movement during chewing and a lower intrusion (.3 mm) of the maxillary complete denture during chewing (paired t test; $p < .05$).

Conclusions: It can be concluded that the use of a DA in complete dentures wearers changes mandible movements, with increases in vertical mandibular movements and lower intrusion of maxillary complete denture during chewing.

Division: IADR/AADR/CADR General Session
Meeting: 2013 IADR/AADR/CADR General Session (Seattle, Washington)
Location: Seattle, Washington
Year: 2013
Final Presentation ID: 3530

Authors

- Compagnoni Marco Antonio (Universidade Estadual Paulista Júlio de Mesquita Filho, Araraquara, N/A, Brazil)
- Marin Danny Omar Mendoza (Universidade Estadual Paulista Júlio de Mesquita Filho, Araraquara, N/A, Brazil)
- Leite Andressa Rosa Perin (Universidade Estadual Paulista Júlio de Mesquita Filho, Araraquara, N/A, Brazil)
- Paleari André Gustavo (Universidade Estadual Paulista Júlio de Mesquita Filho, Araraquara, N/A, Brazil)
- Rodriguez Larissa Santana (Universidade Estadual Paulista Júlio de Mesquita Filho, Araraquara, N/A, Brazil)
- Oliveira Júnior Norberto Martins (Universidade Estadual Paulista Júlio de Mesquita Filho, Araraquara, N/A, Brazil)
- Pero Ana Carolina (Universidade Estadual Paulista Júlio de Mesquita Filho, Araraquara, N/A, Brazil)

Compagnoni Marco Antonio (Universidade Estadual Paulista Júlio de Mesquita Filho, Araraquara, N/A, Brazil)
Marin Danny Omar Mendoza (Universidade Estadual Paulista Júlio de Mesquita Filho, Araraquara, N/A, Brazil)
Leite Andressa Rosa Perin (Universidade Estadual Paulista Júlio de Mesquita Filho, Araraquara, N/A, Brazil)
Paleari André Gustavo (Universidade Estadual Paulista Júlio de Mesquita Filho, Araraquara, N/A, Brazil)
Rodriguez Larissa Santana (Universidade Estadual Paulista Júlio de Mesquita Filho, Araraquara, N/A, Brazil)
Oliveira Júnior Norberto Martins (Universidade Estadual Paulista Júlio de Mesquita Filho, Araraquara, N/A, Brazil)
Pero Ana Carolina (Universidade Estadual Paulista Júlio de Mesquita Filho, Araraquara, N/A, Brazil)

SESSION INFORMATION
Poster Session
Occlusion & Mastication
03/23/2013

Effect of denture adhesive on kinesiographic parameters in complete denture

Braz Dent J. 2014 Sep-Oct;25(5):391-8. doi: 10.1590/0103-6440201302409.

Effect of a denture adhesive on the satisfaction and kinesiographic parameters of complete denture wearers: a cross-over randomized clinical trial.

Marin DO[1], Leite AR[1], Paleari AG[1], Rodriguez LS[1], Oliveira Junior NM[1], Pero AC[1], Compagnoni MA[1].

⊕ Author information

Abstract

The aim of the present study was to assess the effect of a denture adhesive (DA) on patient satisfaction and kinesiographic parameters of complete denture wearers by a cross-over study. Fifty edentulous patients received a set of new complete dentures. After an adaptation period, the participants were enrolled in the trial and randomized to receive a sequence of treatment protocols: Protocol 1- DA use during the first 15 days, followed by no DA for the next 15 days; Protocol 2- no DA during the first 15 days, followed by use of DA for the next 15 days. Outcomes were assessed after 15 days of each sequence of treatment. A questionnaire was used to assess the patients' satisfaction. A kinesiograph was used to record mandible movements and patterns of maxillary complete denture movement during chewing. The Wilcoxon test ($\alpha=0.05$) and a paired sample t-test ($\alpha=0.05$) were used to compare satisfaction levels and kinesiographic data, respectively. Use of DA improved the overall level of patient satisfaction ($p<0.001$). The kinesiographic recordings revealed a significant increase (1.7 mm) in vertical mandible movements ($p<0.001$) during chewing and a lower (0.3 mm) vertical intrusion of the maxillary complete dentures ($p=0.002$) during chewing after using the DA. Use of DA in complete denture wearers improved the patients' satisfaction and altered mandible movements, with increases in vertical movements during chewing and less intrusion of maxillary complete dentures.

PMID: 25517773 DOI: 10.1590/0103-6440201302409

Ethanol-Wet Bonding Technique: One Year Randomized Clinical Trial

Ethanol-Wet Bonding Technique: One Year Randomized Clinical Trial

The clinical durability of adhesive restorations can be compromised by the incomplete penetration of hydrophilic monomers of adhesive systems that are necessary to promote their bonding to dentin, which is a naturally moist substrate. An adhesive technique called ethanol-wet bonding is based on the application of hydrophobic monomers to ethanol-saturated dentin; this technique has been extensively analyzed in the laboratory and demonstrates some advantages.
Objective: this randomized clinical trial aimed to evaluate the clinical performance of adhesive restorations using the ethanol-wet bonding technique (G3) prior to the application of a bonding agent and composite resin in non-carious cervical lesions and to compare the performance with that of the three-step etch-and-rinse (G1) and one-step self-etching (G2) techniques.

Method: ninety-three restorations (31 for each group) were performed in 17 patients by a single operator. No cavity preparation was performed. After 6 and 12 months, the restorations were assessed by 2 previously trained examiners using a modified Ryge criteria for marginal adaptation/staining (kappa=0.81) and retention (kappa=1.00) and analyzed by the Kruskal-Wallis test and Fisher's exact test, respectively.

Result: we did not observe any significant differences among groups at the 6- and 12-month time points for any of the assessed criteria ($p \geqslant 0.05$). The intra-group analysis revealed significant differences between the time intervals baseline/12-months in marginal adaptation in G2 ($p=0.0180$) and in marginal staining in G1 ($p=0.0117$).

Conclusion: overall, restorations placed using the ethanol-wet bonding technique performed equally as well as the other adhesive techniques employed.

Division: IADR/AADR/CADR General Session
Meeting: 2013 IADR/AADR/CADR General Session (Seattle, Washington)
Location: Seattle, Washington
Year: 2013
Final Presentation ID: 3107

Authors

- Araujo, Joyce (Universidade Federal Do Para, Belém, N/A, Brazil)
- Figueiredo Barros, Thais Andrade (Universidade Federal Do Para, Belém, N/A, Brazil)
- França Braga, Esther Marina (Universidade Federal Do Para, Belém, , Brazil)
- Silva E Souza, Patricia Almeida (Universidade Federal Do Para, Belém, N/A, Brazil)
- Loretto, Sandro Cordeiro (Universidade Federal Do Para, Belém, N/A, Brazil)
- Da Silva E Souza Jr, Mario (Universidade Federal Do Para, Belém, , Brazil)

SESSION INFORMATION
Poster Session
Clinical Trials IV
03/23/2013

Araujo, Joyce (Universidade Federal Do Para, Belém, N/A, Brazil)
Figueiredo Barros, Thais Andrade (Universidade Federal Do Para, Belém, N/A, Brazil)
França Braga, Esther Marina (Universidade Federal Do Para, Belém, , Brazil)
Silva E Souza, Patricia Almeida (Universidade Federal Do Para, Belém, N/A, Brazil)
Loretto, Sandro Cordeiro (Universidade Federal Do Para, Belém, N/A, Brazil)
Da Silva E Souza Jr, Mario (Universidade Federal Do Para, Belém, , Brazil)

Ethanol-Wet Bonding Technique: One Year Randomized Clinical Trial

International journal of odontostomatology
On-line version ISSN 0718-381X

Int. J. Odontostomat. vol.10 no.2 Temuco Aug. 2016

http://dx.doi.org/10.4067/S0718-381X2016000200013

Int. J. Odontostomat., 10(2):267-276, 2016.

Ethanol-Wet Bonding Technique: 18-month Clinical Evaluation

Técnica de Adhesión Húmeda en Etanol: Eval [illegible] Clínica a los 18 Meses

Thaís Andrade De Figueiredo Barros*; Joyce Figueira D [illegible] Esther Marina França Braga*; Patricia de Almeida Ro [illegible] e Souza***; Sandro Cordeiro Loretto**** & Mário Hon [illegible] Souza Júnior******

* Escola Superior da Amazônia, Belém, Brazil.

** Federal University of Maranhão, Maranhão, Brazil.

*** Federal University of Pará, Belém, Brazil.

**** Department of Restorative Dentistry, Dental School, Fe [illegible]

ABSTRACT: The objective of this randomized clinical trial was to evaluate the clinical performance up to 18 months of restorations placed using ethanol-wet bonding technique (EWBT) compared with the three-step etch-and-rinse (TSER) and one-step self-etching (OSSE) approaches. Ninety-three non-carious cervical lesions (31 for each group) were restored by one experienced operator in 17 patients under relatively dry conditions using gingival retraction cord, cotton rolls and saliva ejector. Each adhesive system was randomly allocated to one of randomized cervical lesions until the three groups were present in the same subject in equal amounts. The restorations were evaluated at baseline, 6, 12 and 18 months by two blinded and calibrated examiners using the modified US Public Health Service guidelines (USPHS) for the following outcomes: retention (kappa= 1.00), staining and marginal adaptation (kappa=0.81) and analyzed by Fisher's exact and Kruskal-Wallis tests, respectively. No significant differences were observed among groups after 18 months for any of the assessed criteria ($p>0.05$). The intra-group analysis performed by Cochran's test (for retention) and Wilcoxon test (for marginal adaptation/staining) revealed significant differences between the time intervals baseline/18 months in marginal adaptation ($p= 0.0117$) and retention ($p= 0.0101$) for OSSE and in marginal staining for TSER (0.0051) and EWBT ($p= 0.0277$) groups. The survival analysis for retention criteria and the overall clinical success were performed using a log-rank test and did not show significant differences among groups ($p> 0.05$). All three adhesives protocols presented similar clinical performance up to 18 months.

KEY WORDS: dentin, dentin-bonding agents, randomized controlled trial.

Randomized Shortened Dental Arch Study: Periodontal Health Over Five Years

Randomized Shortened Dental Arch Study: Periodontal Health Over Five Years

Objective: The randomized shortened dental arch study was designed to provide relevant clinical outcome data for non-implant treatments with and without molar replacement. The objective of this current analysis was to evaluate the influence of these different treatments on periodontal health.
Method: : In a multicenter trial, patients with complete molar loss in one jaw received either a partial removable dental prosthesis (PRDP) with precision attachments or were treated according to the shortened dental arch (SDA) concept aiming at a premolar occlusion. Chi-square tests, Fisher's exact tests, regression analyses and analyses of variance were conducted for group comparisons.

Result: One hundred thirty two of 152 treated patients reached the five year examination. For the dentition as a whole, minor but significant increases of probing pocket depth and vertical clinical attachment loss were found in the PRDP group. The estimated rise of vertical clinical attachment loss over five years was 0.37 mm. No such changes were detected in the SDA group. Also at the distal sites of the posterior-most teeth of the jaw that received the study treatment, the plaque index according to Silness and Löe, probing pocket depth, vertical clinical attachment loss and bleeding on probing showed a more unfavorable development in the PRDP group. Here, the estimated increases of vertical clinical attachment loss over five years were 0.42 mm (disto-buccal) and 0.60 mm (disto-oral) in the maxilla and 0.36 mm (disto-buccal) and 0.48 mm (disto-oral) in the mandible.

Conclusion: It can be concluded that even in a well maintained patient group significant although relatively small detrimental effects of PRDPs on periodontal health are measurable. In view of these findings, periodontal risks and potential benefits of removable prostheses have to be carefully weighed against each other in prosthetic rehabilitation planning. Funded by the German Research Association (grant WA 831/2-1-6).

- Luthardt Ralph Gunnar (University Ulm, Ulm, N/A, Germany)
- Strub Joerg (University Hospital Freiburg, Freiburg, N/A, Germany)
- Mundt Torsten (University of Greifswald, Greifswald, N/A, Germany)
- Wöstmann Bernd (Justus Liebig University, Giessen, N/A, Germany)
- Pospiech Peter (University Hospital Carl Gustav Carus, Dresden, N/A, Germany)
- Wolfart Stefan (RWTH Aachen University, Aachen, N/A, Germany)
- Jahn Florentine (Friedrich Schiller Universität Jena, Jena, N/A, Germany)
- Kern Mathias (Christian-Albrechts University, Kiel, , Germany)
- Huppertz Jan (Julius-Maximilians University of Würzburg, Würzburg, N/A, Germany)
- Hannak Wolfgang Bernd (Charité-Universitätsmedizin Berlin, Berlin, N/A, Germany)
- Range Ursula (Technische Universität Dresden, DResden, N/A, Germany)
- Marré Birgit (Technische Universität Dresden, Dresden, , Germany)
- Gernet Wolfgang (University of Munich, Munich, N/A, Germany)
- Boening Klaus (Technical University of Dresden, Dresden, N/A, Germany)
- Hartmann Sinsa (Johannes Gutenberg University, Mainz, N/A, Germany)
- Gitt Ingeborg (University Leipzig, Leipzig, N/A, Germany)
- Heydecke Guido (University Medical Center Hamburg-Eppendorf, Hamburg, N/A, Germany)
- Stark Helmut (Zentrum fur Zahn-Mund und Kieferheikunde der Universitat, Bonn, N/A, Germany)

SESSION INFORMATION
Oral Session
Clinical Interventional Studies; Removable Prostheses [CLINICIAN TRACK]
03/23/2013

[illegible]
Mundt Torsten (University of Greifswald, Greifswald, N/A, Germany)
Wöstmann Bernd (Justus Liebig University, Giessen, N/A, Germany)
Pospiech Peter (University Hospital Carl Gustav Carus, Dresden, N/A, Germany)
Wolfart Stefan (RWTH Aachen University, Aachen, N/A, Germany)
Jahn Florentine (Friedrich Schiller Universität Jena, Jena, N/A, Germany)
Kern Mathias (Christian-Albrechts University, Kiel, , Germany)
Huppertz Jan (Julius-Maximilians University of Würzburg, Würzburg, N/A, Germany)
Hannak Wolfgang Bernd (Charité-Universitätsmedizin Berlin, Berlin, N/A, Germany)
Range Ursula (Technische Universität Dresden, DResden, N/A, Germany)
Marré Birgit (Technische Universität Dresden, Dresden, , Germany)
Gernet Wolfgang (University of Munich, Munich, N/A, Germany)
Boening Klaus (Technical University of Dresden, Dresden, N/A, Germany)
Hartmann Sinsa (Johannes Gutenberg University, Mainz, N/A, Germany)
Gitt Ingeborg (University Leipzig, Leipzig, N/A, Germany)
Heydecke Guido (University Medical Center Hamburg-Eppendorf, Hamburg, N/A, Germany)
Stark Helmut (Zentrum fur Zahn-Mund und Kieferheikunde der Universitat, Bonn, N/A, Germany)

Randomized Shortened Dental Arch Study: Periodontal Health Over Five Years

J Oral Rehabil. 2014 Jul;41(7):515-22. doi: 10.1111/joor.12160. Epub 2014 Mar 27.

Management of shortened dental arches and periodontal health: 5-year results of a randomised trial.

Walter MH[1], Marré B, Vach K, Strub J, Mundt T, Stark H, Pospiech P, Wöstmann B, Heydecke G, Kern M, Hartmann S, Luthardt R, Huppertz J, Wolfart S, Hannak W.

Author information

Abstract

In a multicentre randomised trial (German Research Association, grants DFG WA 831/2-1 to 2-6, WO 677/2-1.1 to 2-2.1.; controlled-trials.com ISRCTN97265367), patients with complete molar loss in one jaw received either a partial removable dental prosthesis (PRDP) with precision attachments or treatment according to the SDA concept aiming at pre-molar occlusion. The objective of this current analysis was to evaluate the influence of different treatments on periodontal health. Linear mixed regression models were fitted to quantify the differences between the treatment groups. The assessment at 5 years encompassed 59 patients (PRDP group) and 46 patients (SDA group). For the distal measuring sites of the posterior-most teeth of the study jaw, significant differences were found for the plaque index according to Silness and Löe, vertical clinical attachment loss (CAL-V), probing pocket depth (PPD) and bleeding on probing. These differences were small and showed a slightly more unfavourable course in the PRDP group. With CAL-V and PPD, significant differences were also found for the study jaw as a whole. For CAL-V, the estimated group differences over 5 years amounted to 0.27 mm (95% CI 0.05; 0.48; P = 0.016) for the study jaw and 0.25 mm (95% CI 0.05; 0.45; P = 0.014) for the distal sites of the posterior-most teeth. The respective values for PPD were 0.22 mm (95% CI 0.03; 0.41; P = 0.023) and 0.32 mm (95% CI 0.13; 0.5; P = 0.001). It can be concluded that even in a well-maintained.patient group statistically significant although minor detrimental effects of PRDPs on periodontal health are measurable.

Simplified versus conventional method for complete denture fabrication

Objectives: The purpose of this study was to compare a simplified method for complete denture fabrication to a conventional protocol. We will present final results for a randomized controlled trial that considered oral health quality of life (OHRQoL), patient satisfaction, denture quality, masticatory performance and ability, and costs as outcomes.
Methods: A sample of edentulous adult participants was randomly allocated into two groups: Group S (n=19), which received dentures fabricated by a simplified method, and Group C (n=20), which received conventionally fabricated dentures. Before the interventions and after 3 and 6 months following insertion, OHRQoL was evaluated by means of OHIP-EDENT questionnaire and patient satisfaction by a specific instrument. Denture quality and masticatory performance were assessed three months after delivery by a clinician and masticatory ability by a proper questionnaire. Direct and indirect costs were quantified since clinical exam to the last adjustment. Groups were compared by means of statistical tests suitable for the distribution of data (α=5%).

Results: Groups presented no difference for OHRQoL, denture quality and patient general satisfaction. Differences regarding to patient satisfaction with specific aspects of the dentures were found after 3 months, but were insignificant at 6 months. Mastication was similar for both groups; however, the simplified method has led to a direct cost reduction of 34.9% and less time demanded from patients.

Conclusions: The simplified method is able to produce complete dentures of a quality comparable to those produced by the conventional protocol, influencing the OHQoL, satisfaction of their wearers and mastication similarly, but associated with an important saving of resources.

Division: IADR/AADR/CADR General Session
Meeting: 2013 IADR/AADR/CADR General Session (Seattle, Washington)
Location: Seattle, Washington
Year: 2013
Final Presentation ID: 2867

Authors

- De Souza Raphael (University of São Paulo, Ribeirão Preto, , Brazil)
- Regis Rr (University of São Paulo, Ribeirão Preto, N/A, Brazil)
- Della Vecchia Mp (University of São Paulo, Ribeirão Preto, N/A, Brazil)
- Cunha Tr (University of São Paulo, Ribeirão Preto, N/A, Brazil)
- Ribeiro Adriana (Ribeirão Preto Dental School - University of São Paulo, Ribeirão Preto, , Brazil)
- Muglia Va (University of São Paulo, Ribeirão Preto, N/A, Brazil)
- Mestriner Wilson (University of São Paulo, Ribeirão Preto, N/A, Brazil)
- Silva-lovato Claudia (University of São Paulo, Ribeirão Preto, N/A, Brazil)

De Souza Raphael (University of São Paulo, Ribeirão Preto, , Brazil)
Regis Rr (University of São Paulo, Ribeirão Preto, N/A, Brazil)
Della Vecchia Mp (University of São Paulo, Ribeirão Preto, N/A, Brazil)
Cunha Tr (University of São Paulo, Ribeirão Preto, N/A, Brazil)
Ribeiro Adriana (Ribeirão Preto Dental School - University of São Paulo, Ribeirão Preto, , Brazil)
Muglia Va (University of São Paulo, Ribeirão Preto, N/A, Brazil)
Mestriner Wilson (University of São Paulo, Ribeirão Preto, N/A, Brazil)
Silva-lovato Claudia (University of São Paulo, Ribeirão Preto, N/A, Brazil)

SESSION INFORMATION
Oral Session
Clinical Interventional Studies; Removable Prostheses [CLINICIAN TRACK]
03/23/2013

Simplified versus conventional method for complete denture fabrication

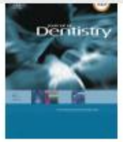

ELSEVIER

Journal of Dentistry
Volume 41, Issue 2, February 2013, Pages 133-142

A randomised trial on simplified and conventional methods for complete denture fabrication: Masticatory performance and ability ☆

T.R. Cunha, M.P. Della Vecchia, R.R. Regis, A.B. Ribeiro, V.A. Muglia, W. Mestriner Jr, R.F. de Souza

University of São Paulo, Ribeirão Preto Dental School, Av. do Café s/n, 14040-904 Ribeirão Preto, SP, Brazil

Received 15 June 2012, Revised 27 August 2012, Accepted 13 September 2012, Available online 19 September 2012.

⊟ Show less

https://doi.org/10.1016/j.jdent.2012.09.008 Get rights and content

Abstract

Objectives

To compare a simplified method to a conventional protocol for complete denture fabricatio regarding masticatory performance and ability.

Methods

A sample was formed by edentulous patients requesting treatment with maxillary and mandibular complete dentures. Participants were randomly divided into two groups: Group S, which received dentures fabricated by a simplified method, and Group C (n = 21 each), which received conventionally fabricated dentures. After three months following insertion, masticatory performance was evaluated by a colorimetric assay based on chewing two capsules as test food during twenty and forty cycles. Masticatory ability was assessed by a questionnaire with binary answers and a single question answered by means of a 0–10 scale. A third group (DN) formed by seventeen dentate volunteers served as an external comparator. Groups were compared by statistical tests suitable for data distribution ($\alpha = 0.05$).

Results

Thirty-nine participants were assessed for three months (twenty from Group C and nineteen from Group S). Groups C and S presented similar masticatory performance which corresponded to approximately 30% of Group DN. Results for masticatory ability showed similarity between S and C, regardless of the assessment method, although an isolate questionnaire item showed more favourable results for the first group.

Conclusions

The simplified method for complete denture fabrication is able to restore masticatory function to a level comparable to a conventional protocol, both physiologically and according to patient's perceptions.

Clinical significance

Although masticatory function is impaired by the loss of natural teeth and dentures can restore only a fraction of such function, patients can benefit from a simplified protocol for complete denture fabrication to the same extent they would by conventional techniques.

2014

12-Months Clinical Comparison of Two Resin Composites on Diastema Closure

12-Months Clinical Comparison of Two Resin Composites on Diastema Closure

Objective: To evaluate the esthetic, functional and biological clinical performance of two nano-hybrid resin composite systems used for anterior diastema closure at 12-months.

Method: Twenty-three patients with anterior midline or multi-diastema problem were enrolled in this study. Nano-hybrid resin composite systems to be used on each patient were randomly selected. Thirty seven teeth of 10 patients were restored with Filtek-Z550 (3M/ESPE) in combination with Adper™ Single Bond 2 (3M/ESPE) etch&rinse adhesive; whereas 39 teeth of 13 patients were restored with Charisma-Diamond (Heraeus Kulzer) in combination with Gluma2 Bond (Heraeus Kulzer) etch&rinse adhesive by two operators. Esthetic (Surface Luster, Surface/Margin Staining, Color Match and Translucency, Esthetic Anatomical Form), Functional (Fracture of Material and Retention, Marginal Adaptation, Patient's View) and Biological (Periodontal Response, Adjacent Mucosa, Oral&General Health) properties of the restorations were evaluated at baseline, 6- and 12-months using FDI Criteria establishing a score-range of 1-5 (1-Clinically excellent/very good, 2-Clinically good, 3-Clinically sufficient/satisfactory, 4-Clinically unsatisfactory and 5-Clinically poor) by two independent examiners. The results were evaluated using the Pearson Chi-Square (p=0.05).

Result: At 12-months, 23 patients were evaluated. Two Filtek-Z550 restorations (5.4%) and 5 Charisma-Diamond (12.8%) restorations exhibited minor surface staining (Score 2). The surface luster of 3 Charisma-Diamond restorations was scored as 2. Three Filtek-Z550 restorations (8.1%) and 1 Charisma-Diamond restoration (2.6%) were scored as 2 with minor irregularities in marginal adaptation. However, there were no significant differences between two restorative materials for surface/marginal staining, surface luster and marginal adaptation (p>0.05). All the restorations in both groups were clinically excellent (Score 1) for the rest of the esthetic, functional and biological properties assessed.

Conclusion: Both nano-hybrid resin composite systems revealed esthetically, functionally and biologically good clinical performance when used for diastema closure at 12-months.

Division: IADR/AMER General Session
Meeting: 2014 IADR/AMER General Session (Cape Town, South Africa)
Location: Cape Town, South Africa
Year: 2014
Final Presentation ID: 1154

Authors

- Kutuk, Zeynep (Hacettepe University, Ankara, , Turkey)
- Ergin, Esra (Hacettepe University, School of Dentistry, Ankara, , Turkey)
- Gurgan, Sevil (Hacettepe University, ankara, , Turkey)
- Yalcin Cakir, Filiz (Hacettepe University, Ankara, , Turkey)
- Oztas, Sema (Hacettepe University, Ankara, , Turkey)

Kutuk, Zeynep (Hacettepe University, Ankara, , Turkey)
Ergin, Esra (Hacettepe University, School of Dentistry, Ankara, , Turkey)
Gurgan, Sevil (Hacettepe University, ankara, , Turkey)
Yalcin Cakir, Filiz (Hacettepe University, Ankara, , Turkey)
Oztas, Sema (Hacettepe University, Ankara, , Turkey)

SESSION INFORMATION
Poster Session
Adhesives and Composite Clinical Trials
06/27/2014

12-Months Clinical Comparison of Two Resin Composites on Diastema Closure

Nigerian Journal of Clinical Practice

JOURNAL HOME | ABOUT THIS JOURNAL | ADVANCED SEARCH | CURRENT ISSUE | ARCHIVES

Journal Home > Vol 21, No 9 (2018) >

Log in or Register to get access to full text downloads.

Username Password Remember me Login or Register

Comparison of two different compos[illegible]
closure in a 4-year follow-up

E Ergin, Z.B. Kutuk, F.Y. Cakir, S Gurgan

Abstract

Aims: This aim of this study is to evaluate and to compare the clinical performances of two nanohybrid composite resin systems used for diastema closure and tooth reshaping at 4 years.

Subjects and Methods: Twenty-three patients with midline or multidiastema problem were enrolled in this study. Nanohybrid resin composite systems to be used on each patient were randomly selected. Thirty seven teeth of 10 patients were restored with Filtek-Z550 (3M/ESPE) in combination with Adper™ Single Bond 2 (3M/ESPE) etch and rinse adhesive in Group 1 whereas 39 teeth of 13 patients were restored with Charisma-Diamond (Heraeus Kulzer) in combination with Gluma2 Bond (Heraeus Kulzer) etch and rinse adhesive in Group 2, by two operators. Esthetic, functional, and biological properties of the restorations were evaluated at baseline, 1, 2, 3, and 4 years using foreign direct investment criteria by two independent examiners. Statistical Analysis Used: The data were evaluated using Fisher's Chi-Square ($P = 0.05$).

Results: Fifty-eight restorations (19 patients) with a mean service time of 43.4 months were evaluated (recall rate 82.6%). One Filtek-Z550 and two Charisma-Diamond restorations were repaired due to partial fracture (Score 4). Survival rates of Group 1 and Group 2 were 96.3% and 93.5%, respectively (Kaplan–Meier) ($P > 0.05$). Qualitative deteriorations were observed within each group according to baseline regarding surface luster, surface/marginal staining, marginal adaptation, patient's view, and periodontal response ($P < 0.05$). However, there were no significant differences between two restorative materials for any of the criteria assessed ($P > 0.05$).

Conclusions: Both nanohybrid composite resin systems revealed esthetically, functionally, and biologically acceptable clinical performance when used for diastema closure and tooth reshaping at 4 years.

Keywords: Clinical performance, composite resin buildup, diastema closure

60-Month Clinical Performance Of A Glass-Ionomer Restorative System

60-Month Clinical Performance Of A Glass-Ionomer Restorative System

Objective: The aim of this study was to evaluate the clinical performance of a glass ionomer restorative system compared with a micro-filled hybrid posterior composite in a 60 month randomized clinical trial.
Method: A total of 140 (80 Cl1 and 60 Cl2) lesions in 59 patients were either restored with a glass-ionomer restorative system (EQUIA/ GC) which was a combination of a packable glass-ionomer (Equia Fil/ GC) and a self-adhesive nano-filled coating (Equia Coat/GC) or with a micro-filled hybrid composite (Gradia Direct Posterior/ GC) in combination with a self-etch adhesive (G-Bond/ GC) by two experienced operators according to the manufacturer's instructions. Two independent examiners evaluated the restorations at baseline, 12- 24-36-48 and 60 months according to the modified United States Public Health Service criteria. The differences between two groups were statistically evaluated by McNemar, Cochron's Q and Chi-Square tests ($p<0.05$).

Result: After 60 months 126 (76 Cl1 and 50 Cl2) restorations were evaluated in 52 patients with a recall rate of 88.1%. None of the restorations showed trends to downgrade in anatomical form, secondary caries, surface texture, postoperative sensitivity and color match ($p>0.05$). Significant differences in marginal adaptation and discoloration were found at 60 months compared to baseline for both restorative materials for Cl1 and Cl2 restorations ($p<0.05$). Only 1 Cl2 Equia restoration was missing at 36 months (3.9%), and another one at 48 months (7.7%) ($p>0.05$). No change was observed at 60 month recall.

Conclusion: The use of both materials for the restoration of posterior teeth exhibited a similar and clinically successful performance after 60 months.

Division: IADR/AMER General Session
Meeting: 2014 IADR/AMER General Session (Cape Town, South Africa)
Location: Cape Town, South Africa
Year: 2014
Final Presentation ID: 89

Authors

- Gurgan, Sevil (Hacettepe University, Ankara, , Turkey)
- Kutuk, Zeynep (Hacettepe University, Ankara, , Turkey)
- Firat, Esra (Hacettepe University, Ankara, N/A, Turkey)
- Çakır, Filiz (Hacettepe University, Ankara, , Turkey)
- Oztas, Serap (Hacettepe University, Ankara, , Turkey)

SESSION INFORMATION
Oral Session
Keynote Address; Restorative Clinical Trials
06/25/2014

Gurgan, Sevil (Hacettepe University, Ankara, , Turkey)
Kutuk, Zeynep (Hacettepe University, Ankara, , Turkey)
Firat, Esra (Hacettepe University, Ankara, N/A, Turkey)
Çakır, Filiz (Hacettepe University, Ankara, , Turkey)
Oztas, Serap (Hacettepe University, Ankara, , Turkey)

60-Month Clinical Performance Of A Glass-Ionomer Restorative System

Oper Dent. 2015 Mar-Apr;40(2):134-43. doi: 10.2341/13-239-C. Epub 2014 Oct 9.

Four-year randomized clinical trial to evaluate the clinical performance of a glass ionomer restorative system.

Gurgan S, Kutuk ZB, Ergin E, Oztas SS, Cakir FY.

Abstract

OBJECTIVE: The aim of this study was to evaluate the clinical performance of a glass ionomer restorative system compared with a microfilled hybrid posterior composite in a four-year randomized clinical trial.

METHODS: A total of 140 (80 Class 1 and 60 Class 2) lesions in 59 patients were either restored with a glass ionomer restorative system (Equia, GC, Tokyo, Japan), which was a combination of a packable glass ionomer (Equia Fil, GC) and a self-adhesive nanofilled coating (Equia Coat, GC), or with a microfilled hybrid composite (Gradia Direct Posterior, GC) in combination with a self-etch adhesive (G-Bond, GC) by two experienced operators according to the manufacturer's instructions. Two independent examiners evaluated the restorations at baseline and at one, two, three, and four years postrestoration according to the modified US Public Health Service criteria. Polyvinyl siloxane impression negative replicas at each recall were observed under scanning electron microscopy (SEM) to evaluate surface characteristics. The statistical analyses were carried out with McNemar, Pearson Chi-square, and Cochran Q-tests ($p<0.05$).

RESULTS: After four years, 126 (76 Class 1 and 50 Class 2) restorations were evaluated in 52 patients, with a recall rate of 88.1%. None of the restorations showed trends to downgrade in anatomical form, secondary caries, surface texture, postoperative sensitivity, and color match ($p>0.05$). Significant differences in marginal adaptation and discoloration were found at four years compared to baseline for both restorative materials for Class 1 and Class 2 restorations ($p<0.05$). Only one Class 2 Equia restoration was missing at three years (3.9%), and another one was missing at four years (7.7%) ($p>0.05$). SEM evaluations were in accordance with the clinical findings.

CONCLUSIONS: The use of both materials for the restoration of posterior teeth exhibited a similar and clinically successful performance after four years.

PMID: 25299703 DOI: 10.2341/13-239-C

Antimicrobial Action and Denture Stomatitis Remission: Effect of Denture Cleansers

Antimicrobial Action and Denture Stomatitis Remission: Effect of Denture Cleansers

Objective: This study reports a cross-over trial about the efficacy of two denture cleansers (sodium hypochlorite and Castor oil solution) in terms of antimicrobial action against *Candida sp* and Denture Stomatitis remission.
Method: Forty-five denture wearers were instructed to brush their dentures (specific brush and liquid soap) three times a day and to soak them (twenty minutes/fifteen days) in the following solutions: (A) 0.20% Sodium Hypochlorite, (B) 0.10% Sodium Hypochlorite, (C) 8% Castor oil solution, (D,control) Saline. After each product use, the internal surfaces of maxillary dentures were brushed (two minutes) with saline solution and the diluted resultant solutions (10^0 to 10^3) were cultured (10 μL aliquots) inside Sabourraud dextrose agar for *Candida* sp. After the incubation, the characteristic colonies were counted by a stereoscopic magnifier and the colony forming units (CFU) were counted and transformed in log10+1. The patients´ palate photos were treated (Microsoft PowerPoint 2010) and scored (Newton´s index). The statistical tests used were Friedman test (α=0.05) followed by multiple comparisons - Wilcoxon test, corrected by Bonferroni (α=0.005) for CFU and Kruskal-Wallis test (α=0.05) followed by Dunn test for denture-related Stomatitis remission.

Result: Sodium hypochlorite resulted in UFC means [(A) μ=1.58±2.33; (B) μ=2.06±2.44] significantly lower than others treatments [(D) μ=4.14±1.92; (C) μ=3.94±2.19]. There was no difference between treatments (C) and (D). For denture-related Stomatitis, both sodium hypochlorite and 8% Castor oil solution were similar [(B) μ=1.38±0.86; (A) μ=1.44±1.01; (C) μ=1.53±0.79] and better than (D) μ=1.82±0.89.

Conclusion: Sodium hypochlorite solutions (0.20% and 0.10%) were effective in the reduction of Candida sp. and Denture Stomatitis. Although 8% Castor oil solution has been effective in the remission of stomatitis, she did not cause changes in the composition of the prosthetic biofilm, when considering such microbial group highly relevant to the oral health of denture wearers.

Division: IADR/AMER General Session
Meeting: 2014 IADR/AMER General Session (Cape Town, South Africa)
Location: Cape Town, South Africa
Year: 2014
Final Presentation ID: 281

Authors

- Arruda, Carolina (Dental School of Ribeirão Preto, Ribeirão Preto - SP, , Brazil)
- Salles, Marcela (University of São Paulo, Ribeirão Preto, , Brazil)
- Badaró, Maurício (Ribeirão Preto Dental School, University of São Paulo, Ribeirão Preto, , Brazil)
- Sorgini, Danilo (Dental School of Ribeirão Preto, Ribeirão Preto, , Brazil)
- Oliveira, Viviane Cássia (University of São Paulo, Ribeirão Preto, N/A, Brazil)
- Silva-lovato, Claudia (University of São Paulo, Ribeirão Preto, N/A, Brazil)
- Paranhos, Helena (University of São Paulo, Ribeirão Preto, N/A, Brazil)

Arruda, Carolina (Dental School of Ribeirão Preto, Ribeirão Preto - SP, , Brazil)
Salles, Marcela (University of São Paulo, Ribeirão Preto, , Brazil)
Badaró, Maurício (Ribeirão Preto Dental School, University of São Paulo, Ribeirão Preto, , Brazil)
Sorgini, Danilo (Dental School of Ribeirão Preto, Ribeirão Preto, , Brazil)
Oliveira, Viviane Cássia (University of São Paulo, Ribeirão Preto, N/A, Brazil)
Silva-lovato, Claudia (University of São Paulo, Ribeirão Preto, N/A, Brazil)
Paranhos, Helena (University of São Paulo, Ribeirão Preto, N/A, Brazil)

SESSION INFORMATION
Poster Session
Removable and Implant Prosthodontics
06/26/2014

Effect of sodium hypochlorite and Ricinus communis solutions on control of denture biofilm: A randomized crossover clinical trial

来自ResearchGate

收藏 引用 批量引用 报错 分享

作者 CNFD Arruda , MM Salles , MM Badaró , VDC Oliveira , AP Macedo , ...

摘要 The prevalence of complete edentulism remains high in the elderly, and previous data have shown that poor denture hygiene is common among edentulous patients. The purpose of this randomized crossover trial was to evaluate the efficacy of denture cleansers in terms of biofilm removal, antimicrobial action, and the remission of denture stomatitis. Fifty denture wearers with denture stomatitis were instructed to brush their dentures (brush and soap) and to soak them (20 minutes/14 days) in 4 solutions, as follows: C (control), 0.85% saline; SH1, 0.1% sodium hypochlorite; SH2, 0.2% sodium hypochlorite; and RC, 8%Ricinus communis.The biofilm in the intaglio surface of maxillary dentures was stained, photographed, and quantified by software (Image Tool). It was then collected (brushed with saline solution), and the obtained suspension was diluted (100to 10-3) and seeded (50 渭L) in CHROMagar forCandidaspp. After incubation, colony-forming units per milliliter values were calculated. Denture stomatitis remission was classified according to the Newton classification. Data were analyzed by Friedman (伪=.05) and Wilcoxon tests and corrected by the Bonferroni test (伪=.005). SH1 (mean rank [MR]=1.98) and SH2 (MR=1.64) showed lower biofilm coverage than C (MR=3.73) that was similar to RC (MR=2.92). SH1 (MR=2.43) and SH2 (MR=2.10) showed antimicrobial action forCandidaspp, and RC (MR=3.36) showed similar results to C (MR=3.51) and baseline (MR=3.50). Clinical signs of denture stomatitis were reduced by SH1 (MR=2.44), while SH2 (MR=2.56) and RC (MR=2.74) showed intermediate results. The 2 sodium hypochlorite solutions were the most effective means of biofilm control. All tested solutions were effective in reducing the signs of denture stomatitis.

收起

出版源 《Journal of Prosthetic Dentistry》 , 2017 , 117 (6)

被引量 3

Caries incidence following tooth replacement for partially dentate elders

Caries incidence following tooth replacement for partially dentate elders

Objective:

To compare caries incidence following two different tooth replacement strategies for partially dentate older patients; namely functionally orientated treatment according to the principles of the Shortened Dental Arch (SDA) and conventional treatment using Removable Partial Dentures (RPDs).

Method:

A randomised controlled clinical trial (RCT) was conducted of partially dentate patients aged 65 years and older. Patients were randomly allocated to two different treatment groups: the RPD group and the SDA group. Each member of the RPD group was restored to complete arches with cobalt-chromium RPDs used to replace missing teeth. Patients in the SDA group were restored to a shortened arch of 10 occluding pairs of natural and replacement teeth using adhesive bridgework. All of the operative treatment was completed by a single operator. Caries incidence was measured over a 2-year period following treatment intervention and recorded using the International Caries and Detection System (ICDAS).

Result:

In total, 89 patients completed the RCT (45 SDAs and 44 RPDs). Patients in the RPD group recorded a significantly higher incidence of new carious lesions ($p<0.001$) and recurrent carious lesions ($p<0.001$) compared to the SDA group. A mixed model of covariance (ANCOVA) revealed that treatment group ($p<0.001$) and co-morbidity ($p<0.001$) were significant predictors of caries incidence.

Conclusion:

Two years after provision of prosthodontic treatment there was a significantly higher incidence of new and recurrent caries lesions in subjects provided with RPDs compared with SDA treatment. This will have a significant impact on the ongoing maintenance costs for these two treatment groups.

Division: IADR/AMER General Session
Meeting: 2014 IADR/AMER General Session (Cape Town, South Africa)
Location: Cape Town, South Africa
Year: 2014
Final Presentation ID: 328

Authors

- Mckenna, Gerald (University College Cork, Cork, , Ireland)
- Allen, Finbarr (University College Cork, Cork, , Ireland)
- O'mahony, Denis (University College Cork, Cork, , Ireland)
- Cronin, Michael (University College Cork, Cork, , Ireland)
- Da Mata, Cristiane Mendonca (University College Cork, Cork, , Ireland)
- Woods, Noel (University College Cork, Cork, , Ireland)

Mckenna, Gerald (University College Cork, Cork, , Ireland)
Allen, Finbarr (University College Cork, Cork, , Ireland)
O'mahony, Denis (University College Cork, Cork, , Ireland)
Cronin, Michael (University College Cork, Cork, , Ireland)
Da Mata, Cristiane Mendonca (University College Cork, Cork, , Ireland)
Woods, Noel (University College Cork, Cork, , Ireland)

SESSION INFORMATION
Poster Session
The J. Morita Award Poster Competition in Geriatric Oral Health Research
06/26/2014

Caries incidence following tooth replacement for partially dentate elders

COMMUNITY DENTISTRY AND ORAL EPIDEMIOLOGY

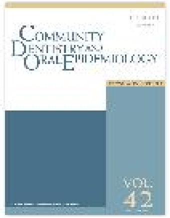

Volume 42, Issue 4
August 2014

Original Article | Full Access

Cost-effectiveness of tooth replacement strategies for partially dentate elderly: a randomized controlled clinical trial

Gerald McKenna, Finbarr Allen, Noel Woods, Denis O'Mahony, Michael Cronin, Cristiane DaMata, Charles Normand

First published: 20 November 2013 | https://doi.org/10.1111/cdoe.12085 | Cited by: 13

Advertisement
Wiley Ed
Ensure your
ready for su

Abstract

Objective

To conduct a cost-effectiveness analysis comparing two different tooth replacement strategies for partially dentate older patients, namely partial removable dental prostheses (RDP) and functionally orientated treatment based on the shortened dental arch concept (SDA).

Methods

Ninety-two partially dentate older patients completed a randomized controlled clinical trial. Patients were randomly allocated to two treatment groups: the RDP group and the SDA group. Treatment effect was measured using impact on oral health–related quality of life (OHrQOL), and the costs involved in providing and maintaining care were recorded for all patients. Patients were followed for 12 months after treatment intervention. All treatment was provided by a single operator.

Results

The total cost of achieving the minimally important clinical difference (MID) in OHrQOL for an average patient in the RDP group was €464.64. For the SDA group, the cost of achieving the MID for an average patient was €252.00. The cost-effectiveness ratio was therefore 1:1.84 in favour of SDA treatment.

Conclusion

With an increasingly ageing population, many patients will continue to benefit from removable prostheses to replace their missing natural teeth. From a purely economic standpoint, the results from this analysis suggest that the treatment of partially dentate older adults should be focused on functionally orientated treatment because it is simply more cost-effective.

Clinical Implications from a RCT of Complete Denture Impression Materials

Clinical Implications from a RCT of Complete Denture Impression Materials

Objective:

There is a continuing need for non-implant prosthodontic treatment yet there is a paucity of high quality randomised controlled trial (RCT) evidence for best practice. The aim was to provide RCT evidence for prosthodontic impressions. Here we report all clinical outcomes.

Method:

This was a double-blind, randomised, crossover trial comparing two impression materials for complete dentures. Using eligibility criteria, 85 patients were recruited at Leeds Dental Institute, UK. Each patient received two sets of dentures made using either alginate or silicone impressions. Unadjusted dentures were worn alternately over 2 weeks, adjusted over 2 sequential periods of 8 weeks then worn side-by-side for a further two weeks. The order of construction and wear of the dentures was randomised. The primary outcome was patient preference for unadjusted dentures. Secondary outcomes were: patients' quality of life (OHIP-EDENT); patient-centred rating of comfort, stability and chewing efficiency before and after adjustment; patient assessment and preference of the impression materials.

Result:

78 (91.8%) patients completed the primary assessment. There was a 50% difference in preference rates (in favour of silicone) (95%CI 32.7% to 67.3%, $p<0.0001$). The unadjusted dentures made from silicone impressions were rated more comfortable ($p=0.0039$), more stable ($p=0.0047$) and more efficient ($p<0.0001$). During the two 8 week Adjustment Periods, the OHIP-EDENT score was lower wearing dentures made from silicone impressions ($p=0.0014$). After the 'confirmation' period there was significant evidence that dentures made from silicone impressions were rated as more stable ($p=0.0066$) and more efficient ($p=0.0010$) but following adjustment of the dentures there was no difference in comfort between the dentures ($p=0.5417$). At the end of the trial there was a 33.8% difference in preference rates for the adjusted dentures (in favour of silicone) (95%CI 14.3% to 53.3%, $p=0.0016$).

Conclusion:

There is significant evidence that dentures made from silicone impressions were preferred by patients.

Division: IADR/AMER General Session
Meeting: 2014 IADR/AMER General Session (Cape Town, South Africa)
Location: Cape Town, South Africa
Year: 2014
Final Presentation ID: 898

Authors

- Hyde T. Paul (University of Leeds, Leeds, N/A, England)
- Brunton Paul A. (University of Leeds, Leeds, N/A, England)
- Craddock Helen (University of Aberdeen, Aberdeen, N/A, Scotland)
- Gray Janine (University of Leeds, Leeds, N/A, England)
- Pavitt Sue (University of Leeds, Leeds, N/A, England)
- Hulme Claire (University of Leeds, Leeds, N/A, England)
- Godfrey Mary (University of Leeds, Leeds, , England)
- Brown Sarah (University of Leeds, Leeds, N/A, England)
- Fernandez Catherine (University of Leeds, Leeds, N/A, England)
- Wright Jonathan (University of Leeds, Leeds, N/A, England)

SESSION INFORMATION
Poster Session
Senior-Clinical Research
06/27/2014

Hyde T. Paul (University of Leeds, Leeds, N/A, England)
Brunton Paul A. (University of Leeds, Leeds, N/A, England)
Craddock Helen (University of Aberdeen, Aberdeen, N/A, Scotland)
Gray Janine (University of Leeds, Leeds, N/A, England)
Pavitt Sue (University of Leeds, Leeds, N/A, England)
Hulme Claire (University of Leeds, Leeds, N/A, England)
Godfrey Mary (University of Leeds, Leeds, , England)
Brown Sarah (University of Leeds, Leeds, N/A, England)
Fernandez Catherine (University of Leeds, Leeds, N/A, England)
Wright Jonathan (University of Leeds, Leeds, N/A, England)

Clinical Implications from a RCT of Complete Denture Impression Materials

Journal of Dentistry
Volume 42, Issue 8, August 2014, Pages 895-901

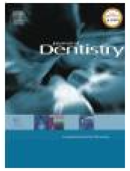

A Randomised Controlled Trial of complete denture impression materials

T.P. Hyde [a], H.L. Craddock [b], J.C. Gray [c], S.H. Pavitt [d], C. Hulme [e], M. Godfrey [f], C. Fernandez [c], N. Navarro-Coy [g], S. Dillon [a], J. Wright [g], S. Brown [c], G. Dukanovic [h], P.A. Brunton [a, i]

⊞ Show more

https://doi.org/10.1016/j.jdent.2014.02.005 Get rights and conten

Open Access funded by Department of Health UK

Under a Creative Commons license open access

Abstract

Objectives

There is continuing demand for non-implant prosthodontic treatment and yet there is a paucity of high quality Randomised Controlled Trial (RCT) evidence for best practice. The aim of this research was to provide evidence for best practice in prosthodontic impressions by comparing two impression materials in a double-blind, randomised, crossover, controlled, clinical trial.

Methods

Eighty-five patients were recruited, using published eligibility criteria, to the trial at Leeds Dental Institute, UK. Each patient received two sets of dentures; made using either alginate or silicone impressions. Randomisations determined the order of assessment and order of impressions. The primary outcome was patient blinded preference for unadjusted dentures. Secondary outcomes were patient preference for the adjusted dentures, rating of comfort, stability and chewing efficiency, experience of each impression, and an OHIP-EDENT questionnaire.

Results

Seventy-eight (91.8%) patients completed the primary assessment. 53(67.9%) patients preferred dentures made from silicone impressions while 14(17.9%) preferred alginate impressions. 4(5.1%) patients found both dentures equally satisfactory and 7 (9.0%) found both equally unsatisfactory. There was a 50% difference in preference rates (in favour of silicone) (95%CI 32.7–67.3%, $p < 0.0001$).

Conclusion

There is significant evidence that dentures made from silicone impressions were preferred by patients.

Clinical significance

Given the strength of the clinical findings within this paper, dentists should consider choosing silicone rather than alginate as their material of choice for secondary impressions for complete dentures.

Trial Registration: ISRCTN 01528038.

Efficacy of Stabilization Splint Treatment on Facial Pain-One-year Follow-up

Efficacy of Stabilization Splint Treatment on Facial Pain-One-year Follow-up

Objective:

There are contradicting results on the efficacy of stabilization splint treatment on temporomandibular disorders (TMD). The aim of this randomized control trial was to assess the efficacy of stabilization splint treatment on TMD-related facial pain during a one-year follow-up.

Method:

The sample consisted of 80 TMD patients who were randomly assigned to two groups; splint group (n=39) and control group (n=41). The patients in the splint group were treated with a stabilization splint and received counseling and instructions for masticatory muscle exercises. The controls received only counseling and instructions for masticatory muscles exercises.

The outcome variables were the change in the intensity of facial pain (as measured with VAS, visual analogue scale) between baseline and one-year follow-up as well as the patients' subjective estimate of treatment outcome. The differences in VAS changes between the groups were analyzed using variance analysis and linear regression models.

Result:

Facial pain decreased in both groups but the difference in VAS change between the groups was not statistically significant. In the linear regression analysis the group status did not associate with the change in VAS after adjustment for baseline VAS, gender, age, length of treatment and general health status. After one-year follow-up, 27.6% of the patients in the splint group and 37.5% of the patients in the control group reported "very good" treatment effects.

Conclusion:

The findings of this study did not show stabilization splint treatment to be more effective in decreasing facial pain than masticatory muscle exercises and counseling alone in treatment of TMD over a one-year follow-up.

[illegible]

Authors

- Qvintus, Veera (University of Eastern Finland, Kuopio, , Finland)
- Suominen, Anna Liisa (University of Eastern Finland, Kuopio, , Finland)
- Huttunen, Jussi (University of Eastern Finland, Kuopio, , Finland)
- Raustia, Aune (University of Oulu, Oulu, , Finland)
- Ylöstalo, Pekka (University of Eastern Finland, Kuopio, , Finland)
- Sipila, Kirsi (University of Eastern Finland, Kuopio, , Finland)

SESSION INFORMATION

Poster Session

Physiology and Pathology of TMJ and Muscles of Mastication

06/28/2014

Qvintus, Veera (University of Eastern Finland, Kuopio, , Finland)
Suominen, Anna Liisa (University of Eastern Finland, Kuopio, , Finland)
Huttunen, Jussi (University of Eastern Finland, Kuopio, , Finland)
Raustia, Aune (University of Oulu, Oulu, , Finland)
Ylöstalo, Pekka (University of Eastern Finland, Kuopio, , Finland)
Sipila, Kirsi (University of Eastern Finland, Kuopio, , Finland)

J Oral Rehabil. 2015 Jun;42(6):439-46. doi: 10.1111/joor.12275. Epub 2015 Jan 17.

Efficacy of stabilisation splint treatment on facial pain - 1-year follow-up.

Qvintus V[1], Suominen AL, Huttunen J, Raustia A, Ylöstalo P, Sipilä K.

Author information

1 Institute of Dentistry, University of Eastern Finland, Kuopio, Finland.

Abstract

The aim of this randomised controlled trial was to assess the efficacy of stabilisation splint treatment on TMD-related facial pain during a 1-year follow-up. Eighty patients were randomly assigned to two groups: splint group (n = 39) and control group (n = 41). The patients in the splint group were treated with a stabilisation splint and received counselling and instructions for masticatory muscle exercises. The controls received only counselling and instructions for masticatory muscles exercises. The outcome variables were the change in the intensity of facial pain (as measured with visual analogue scale, VAS) as well as the patients' subjective estimate of treatment outcome. The differences in VAS changes between the groups were analysed using variance analysis and linear regression models. The VAS decreased in both groups, the difference between the groups being not statistically significant. The group status did not significantly associate with the decrease in VAS after adjustment for baseline VAS, gender, age, length of treatment and general health status. The only statistically significant predicting factor was the baseline VAS, which was also confirmed by the mixed-effect linear model. After 1-year follow-up, 27.6% of the patients in the splint group and 37.5% of the patients in the control group reported 'very good' treatment effects. The findings of this study did not show stabilisation splint treatment to be more effective in decreasing facial pain than masticatory muscle exercises and counselling alone in the treatment of TMD-related facial pain over a 1-year follow-up.

KEYWORDS: RCT; facial pain; stabilisation splint; temporomandibular disorders; treatment

PMID: 25644634 DOI: 10.1111/joor.12275

Implant Maintenance with a Chitosan Brush –A Randomized Clinical Trial

Implant Maintenance with a Chitosan Brush –A Randomized Clinical Trial

Objective:

Peri-implant mucositis is common and it is reported that long term mucositis may induce peri-implantitis. Treating mucositis and to develop easily applied methods for maintenance of dental implants is thus crucial. It is also important to avoid leaving instrument remnants potentially causing a foreign body reaction or to damage the titanium surface when debriding the implant. Chitosan, a natural biopolymer, has been demonstrated to be biocompatible and biodegradable. Chitosan has also been suggested to have anti-inflammatory and antimicrobial properties. In this study a rotating chitosan brush was evaluated

Method:

This was a randomized, split mouth, examiner blinded, clinical trial of 6 months duration including 12 patients diagnosed with peri-implant mucositis. The study had been approved by the regional ethics committee. Implants were randomized to either treatment with a rotating chitosan brush (BioClean, LABRIDA AS, Oslo Norway) using a slow speed dental handpiece or titanium curettes (Langer and Langer, Rønvig, Denmark). The treatment was repeated at three months. Examinations including probing pocket depths (PPD), bleeding on probing (BoP) and radiographs were performed by two board certified periodontists, blinded to treatment allocation. Differences between groups in change in clinical parameters were compared at 2 weeks, 4 weeks and 6 months. A Mann-Whitney U test with an alpha level of 0.05 was used for the statistical analyses

Result:

Both groups demonstrated significant reductions in clinical parameters between baseline and 4 weeks. The test implants treated with the chitosan brush had a better improvement in BoP at 2 weeks and a better improvement in PPD at 2 weeks and 4 weeks as compared with the implants treated with the titanium curettes. None of the implants demonstrated progression in bone-loss during the course of the study.

Conclusion:

A chitosan brush seems to be a safe and efficient device for maintenance of dental implants.

- Aass Anne (University of Oslo, Oslo, , Norway)
- Lyngstadaas Ståle (University of Oslo, Oslo, , Norway)
- Koldsland Odd (University of Oslo, Oslo, , Norway)

SESSION INFORMATION
Poster Session
Tissue Engineering/Wound Healing and Regeneration/Peri-implant Diseases
06/26/2014

Norway)
Aass Anne (University of Oslo, Oslo, , Norway)
Lyngstadaas Ståle (University of Oslo, Oslo, , Norway)
Koldsland Odd (University of Oslo, Oslo, , Norway)

Int J Implant Dent. 2017 Dec;3(1):38. doi: 10.1186/s40729-017-0098-y. Epub 2017 Aug 3.

A novel non-surgical method for mild peri-implantitis- a multicenter consecutive case series.

Wohlfahrt JC[1], Evensen BJ[2], Zeza B[3], Jansson H[4], Pilloni A[3], Roos-Jansåker AM[5,6], Di Tanna GL[7], Aass AM[8], Klepp M[9], Koldsland OC[8].

Author information

Abstract

AIM: The aim of the present study was to evaluate the effect on peri-implant mucosal inflammation from the use of a novel instrument made of chitosan in the non-surgical treatment of mild peri-implantitis across several clinical centers.

MATERIALS AND METHODS: In this 6-month multicenter prospective consecutive case series performed in six different periodontal specialist clinics, 63 implants in 63 patients were finally included. The subjects had mild peri-implantitis defined as radiographic bone loss of 1-2 mm, pocket probing depth (PPD) ≥4 mm and a positive bleeding on probing (mBoP) score. The patients were clinically examined at baseline and after 2, 4, 12 and 24 weeks, and radiographs were taken at baseline and at 3 and 6 months. Treatment of the implants with the chitosan brush seated in an oscillating dental drill piece was performed at baseline and at 3 months. Reductions in the clinical parameters (PPD and mBoP) were compared between baseline and the later examination time points.

RESULTS: Significant reductions in both PPD and mBoP were observed at all time points compared with the baseline clinical measurements ($p < 0.001$). The mean PPD and mBoP at baseline were 5.15 mm (4.97; 5.32) and 1.86 (1.78; 1.93), respectively, whereas the mean PPD and mBoP at 6 months were 4.0 mm (3.91; 4.19) and 0.64 (0.54; 0.75), respectively. Stable reductions in PPD and mBoP were evident up to 6 months after the initial treatment and 3 months after the second treatment. All 63 implants were reported to have stable radiographic levels of osseous support.

CONCLUSIONS: This case series demonstrated that an oscillating chitosan brush is safe to use and seems to have merits in the non-surgical treatment of dental implants with mild peri-implantitis. To measure the effectiveness of the method, a multicenter randomized clinical trial needs to be undertaken.

KEYWORDS: Chitosan; Clinical study; Dental implants; Non-surgical treatment; Peri-implantitis

PMID: 28776288 PMCID: PMC5543013 DOI: 10.1186/s40729-017-0098-y

Marginal Bone Level Changes and implant neck configuration. Split-mouth RCT

Marginal Bone Level Changes and implant neck configuration. Split-mouth RCT

Objective:
This research presents 3-year results from a previously 1-year randomized, controlled, split-mouth, clinical trial, comparing the clinical and radiological outcomes of two implant designs with different prosthetic interfaces and neck configurations.

Method: Thirty-four partially edentulous patients randomly received at least one implant with back-tapered collar, internal conical connection, platform shifting design, and one external hexagon implant with flat-to-flat implant-abutment interface. Primary endpoints: the implant and prosthetic success rates, all surgical and prosthetic complications occurring during the entire follow-up period. Secondary outcome measures: horizontal and vertical marginal bone level (MBL) changes, resonance frequency analysis values, periodontal parameters: sulcus bleeding index (SBI), plaque score (PS) papilla index.

Result: All patients were followed for 3-years. No drop-outs occurred. No implants and prostheses failures, yielding a 100% cumulative survival rate. MBL showed statistically significant difference with better results for the internal conical connection for both horizontal and vertical measurements ($P<.05$). After 3 years of loading, the conical connection implants lost a mean of vertical MBL of 0.67 mm compared with 1.24 mm for conventional external hexagon implants ($P=.000$); the mean horizontal MBL was 0.20 mm for the conical connection implants and 0.60 mm for the external hexagon ones ($P=.000$). A high ISQ value was found for both implants, no statistically significant difference was found for ISQ mean values between interventions ($P>.05$). All implants showed good periodontal outcomes, with no significant differences between groups.

Conclusion: The results suggest that in well maintained patients the marginal bone level changes could be affected by the prosthetic interface and implant neck configuration. Both implants provided good results. However, both horizontal and vertical marginal bone loss were statistically significant differences, with lower values in the back-tapered neck configuration with conical connection and built-in platform shifting, compared to the straight neck configuration, flat-to-flat implant-abutment interface and external hexagonal connection.

Division: IADR/AMER General Session
Meeting: 2014 IADR/AMER General Session (Cape Town, South Africa)
Location: Cape Town, South Africa
Year: 2014
Final Presentation ID: 39

Authors

- Pozzi Alessandro (Researcher University of Tor Vergata, Rome, , Italy)
- Tallarico Marco (Lecturer University of Tor Vergata, Rome, , Italy)
- Moy Peter (West Coast Oral and Maxillofacial Surgery Center, University of California, Los Angeles, CA, USA)

SESSION INFORMATION
Oral Session
Keynote Address; Surfaces/Materials/Design/Treatment Planning
06/25/2014

Pozzi Alessandro (Researcher University of Tor Vergata, Rome, , Italy)
Tallarico Marco (Lecturer University of Tor Vergata, Rome, , Italy)
Moy Peter (West Coast Oral and Maxillofacial Surgery Center, University of California, Los Angeles, CA, USA)

Marginal Bone Level Changes and implant neck configuration. Split-mouth RCT

CLINICAL IMPLANT DENTISTRY
and Related Research

ARTICLE

Clinical and Radiological Outcomes of Two Implants with Different Prosthetic Interfaces and Neck Configurations: Randomized, Controlled, Split-Mouth Clinical Trial

Alessandro Pozzi DDS, PhD, Enrico Agliardi MD, DDS, Marco Tallarico DDS,
Alberto Barlattani MD, DDS, PhD

First published: 01 June 2012 | https://doi.org/10.1111/j.1708-8208.2012.00465.x | Cited by: 18

Conflict of interest: None.

Read the full text >

PDF TOOLS SHARE

ABSTRACT

Background: Peri-implant bone loss seems to occur following implant placement/loading regardless of all the efforts to eliminate it. Several factors, including surgical trauma, biologic width establishment, lack of passive fit of the superstructures, implant-abutment microgap, and occlusal overloading, may increase peri-implant bone loss. Over the years, new interface designs were introduced and clinical studies suggest that internal conical connection and platform shifting may be advantageous for marginal bone preservation.

Purpose: To compare clinical and radiological outcomes of two implant designs with different prosthetic interfaces and neck configurations in a randomized, controlled, split-mouth clinical trial.

Materials and Methods: Thirty-four partially edentate patients randomly received at least one internal conical connection with back-tapered collar and platform shifting design or external-hexagon implants with flat-to-flat implant-abutment interface. Primary end point was peri-implant bone level changes at different time points, failures of implants and/or prosthesis, any complications, implant stability quotient (ISQ) values, and periodontal parameters.

Results: No dropout occurred. Marginal bone changes were statistically significantly different with better results for the internal conical connection. No implants and prosthesis failures have been observed, yielding a cumulative survival rate of 100%. A high ISQ value was found for both implants, and no statistically significant difference was found for ISQ mean values between interventions at each time point ($p > .05$). All implants showed no bleeding on probing and a very slight amount of plaque at the 1-year-in-function visit.

Conclusions: Both implant designs investigated performed similarly in terms of failure rates, providing successful results up to 1 year after loading. The back-tapered neck configuration with conical connection and built-in platform shifting showed statistically lower marginal bone loss than straight neck configuration with flat-to-flat implant-abutment interface and external-hexagonal connection.

R.communis and sodium hypochlorite's clinical evaluation for denture cleansing

R.communis and sodium hypochlorite's clinical evaluation for denture cleansing

Objective: This clinical trial evaluated the efficacy of an experimental solution (*Ricinus communis)*for cleaning dentures, comparing it to sodium hypochlorite. The variables were the effectiveness of biofilm removal from the inner surface of maxillary dentures and the patient's satisfaction.
Method: 64 upper denture users were instructed to brush the prosthesis for 3 minutes, three times a day and to immerse them for 20 minutes in the solutions: S1-Saline; S2/S3-0,25 and 0,5% Sodium Hypochlorite; S4-10% *Ricinus communis*. They were used in a randomized sequence and crossed form for 07 days, with washout of 07 days. The biofilm quantification was performed by the photographic and computerized method (ImageTool 3.0). A questionnaire was used to check the patient's satisfaction regarding cleanliness, smell, taste, ease of use, the possibility of daily use and indication of use. The ability of biofilm removal was evaluated by General Linear Model and Tukey-LSD test ($p<0.05$). The analysis of the patients' satisfaction was performed descriptively.

Result: There were differences among the solutions, having S2 and S3 promoted the lowest average biofilm (4.41±7.98 and 2.93±5.23), followed by *R. communis*(6.95±10.93) and Saline (11.07±11.99). The satisfaction questionnaire indicated no difference among the solutions.

Conclusion: It was concluded that there is feasibility for using Sodium hypochlorite at lower concentrations and *R. communis*for the removal of biofilm. As to satisfaction, all products were accepted for over 90% of patients.

Division: IADR/AMER General Session
Meeting: 2014 IADR/AMER General Session (Cape Town, South Africa)
Location: Cape Town, South Africa
Year: 2014
Final Presentation ID: 282

Authors

- Badaró Maurício (Ribeirão Preto Dental School, University of São Paulo, Ribeirão Preto, , Brazil)
- Salles Marcela (University of São Paulo, Ribeirão Preto, , Brazil)
- Leite Vanessa (Ribeirao Preto School of Dentistry, University of Sao Paulo, Ribeirao Preto, , Brazil)
- Arruda Carolina (Dental School of Ribeirão Preto, Ribeirão Preto - SP, , Brazil)
- Oliveira Viviane Cássia (University of São Paulo, Ribeirão Preto, N/A, Brazil)
- De Souza Raphael (Ribeirão Preto Dental School - University of São Paulo, Ribeirão Preto, , Brazil)
- Paranhos Helena (University of São Paulo, Ribeirão Preto, N/A, Brazil)
- Silva-lovato Claudia (University of São Paulo, Ribeirão Preto, N/A, Brazil)

SESSION INFORMATION
Poster Session
Removable and Implant Prosthodontics
06/26/2014

Badaró Maurício (Ribeirão Preto Dental School, University of São Paulo, Ribeirão Preto, , Brazil)
Salles Marcela (University of São Paulo, Ribeirão Preto, , Brazil)
Leite Vanessa (Ribeirao Preto School of Dentistry, University of Sao Paulo, Ribeirao Preto, , Brazil)
Arruda Carolina (Dental School of Ribeirão Preto, Ribeirão Preto - SP, , Brazil)
Oliveira Viviane Cássia (University of São Paulo, Ribeirão Preto, N/A, Brazil)
De Souza Raphael (Ribeirão Preto Dental School - University of São Paulo, Ribeirão Preto, , Brazil)
Paranhos Helena (University of São Paulo, Ribeo, N/A, Brazil)
Silva-lovato Claudia (University of São Paulo, Ribeirão Preto, N/A, Brazil)

R.communis and sodium hypochlorite's clinical evaluation for denture cleansing

J Appl Oral Sci. 2017 May-Jun; 25(3): 324–334.
doi: 10.1590/1678-7757-2016-0222
PMCID: PMC5482256
PMID: 28678952

Clinical trial for evaluation of *Ricinus communis* and sodium hypochlorite as denture cleanser

Maurício Malheiros BADARÓ,[1] Marcela Moreira SALLES,[1] Vanessa Maria Fagundes LEITE,[1] Carolina Noronha Ferraz de ARRUDA,[1] Viviane de Cássia OLIVEIRA,[1] Cássio do NASCIMENTO,[1] Raphael Freitas de SOUZA,[1] Helena de Freitas de Oliveira PARANHOS,[1] and Cláudia Helena SILVA-LOVATO[1]

Author information ► Article notes ► Copyright and License information ► Disclaimer

Abstract

Go to:

The development of opportunistic infections due to poor denture hygiene conditions justified the search for effective hygiene protocols for controlling denture biofilm.

Objective

Go to:

This study evaluated *Ricinus communis* and sodium hypochlorite solutions in terms of biofilm removal ability, remission of candidiasis, antimicrobial activity, and participant satisfaction.

Material and Methods

Go to:

It was conducted a controlled clinical trial, randomized, double-blind, and crossover. Sixty-four denture wearers with (n=24) and without candidiasis (n=40) were instructed to brush (3 times/day) and immerse their dentures (20 min/day) in different storage solutions (S1 / S2: 0.25% / 0.5% sodium hypochlorite; S3: 10% *R. communis*; S4: Saline).The trial period for each solution was seven days and a washout period of seven days was used before starting the use of another solution. The variables were analyzed at baseline and after each trial period. The biofilm of inner surfaces of maxillary dentures was disclosed, photographed, and total and dyed areas were measured (Image Tool software). The percentage of biofilm was calculated. Remission of candidiasis was assessed by visual scale and score were attributed. Antimicrobial activity was assessed by the DNA-Checkerboard hybridization method. Patient satisfaction was measured using a questionnaire.

Results

Go to:

S1 (4.41±7.98%) and S2 (2.93±5.23%) were more effective then S3 (6.95±10.93%) in biofilm remotion($P<0.0001$). All solutions were different from the control (11.07±11.99%). S3 was the most effective solution in remission of candidiasis (50%), followed by S1 (46%). Concerning antimicrobial action, S1/S2 were similar and resulted in the lowest microorganism mean count ($P=0.04$), followed by S3. No significant differences were found with patient's satisfaction.

Conclusions

Go to:

10% *R. communis* and 0.25% sodium hypochlorite were effective in biofilm removal, causing remission of candidiasis and reducing the formation of microbial colonies in denture surfaces. All solutions were approved by patients.

Keywords: Denture, Biofilms, Candidiasis, Disinfection

Randomized shortened dental arch study: tooth loss over eight years

Randomized shortened dental arch study: tooth loss over eight years

Objectives: This multicenter randomized controlled clinical trial was designed to provide clinical outcome data for two treatments of the shortened dental arch (SDA).
Methods: Patients with complete molar loss in one jaw were provided with either a partial removable dental prosthesis (PRDP) retained with precision attachments or were treated according to SDA concept aiming at a premolar occlusion without molar replacement. Implant treatments were excluded. Primary outcome was tooth loss. For evaluation, patient related Kaplan-Meier survival analyses were performed.

Results: Of 215 enrolled patients, 152 were actually treated. Over 8 years, 56 patients experienced tooth loss. For the primary outcome tooth loss regardless of the jaw, the 8-year Kaplan-Meier survival rates were .540 (SE .067) in the PRDP group and .634 (SE .066) in the SDA group. A further analysis was conducted for tooth loss in the study jaw, i.e. the jaw in which the study treatment had been delivered. The respective survival rates were .678 (SE .061) in the PRDP group and .671 (SE .066) in the SDA group. With both analyses, the survival functions showed a steady, almost linear decline over time in both groups. No significant group differences were found (Log Rank test, P=.05).

Conclusion: The overall treatment goals of a sustainable oral rehabilitation and the avoidance of further tooth loss over longer periods were not reliably achievable. No influence of the type of prosthetic treatment on tooth loss was found. In view of this result, patient preferences gain further importance in clinical decision making.

Division: IADR/AMER General Session
Meeting: 2014 IADR/AMER General Session (Cape Town, South Africa)
Location: Cape Town, South Africa
Year: 2014
Final Presentation ID: 1373

Authors

- Walter Michael (University of Dresden, Dresden, , Germany)
- Jahn Florentine (Friedrich Schiller Universität Jena, Jena, N/A, Germany)
- Strub Joerg R. (University Hospital, School of Dentistry, Freiburg, N/A, Germany)
- Pospiech Peter (Julius-Maximilian University of Würzburg, Würzburg, , Germany)
- Brückner Julian (University of Leipzig, Leipzig, , Germany)
- Wolfart Stefan (University Hospital RWTH Aachen, Aachen, , Germany)
- Busche Eckhard (Witten/Herdecke University, Faculty of Health, Witten, N/A, Germany)
- Luthardt Ralph (Ulm University, Center of Dentistry, Ulm, , Germany)
- Heydecke Guido (University Medical Center Hamburg-Eppendorf, Hamburg, N/A, Germany)
- Marré Birgit (Technische Universität Dresden, Dresden, , Germany)
- Hannak Wolfgang Bernd (Charité-Universitätsmedizin Berlin, Berlin, N/A, Germany)
- Kern Matthias (Christian-Albrechts University, Kiel, , Germany)
- Mundt Torsten (University of Greifswald, Greifswald, N/A, Germany)
- Gernet Wolfgang (University of Munich, Munich, N/A, Germany)
- Wöstmann Bernd (Justus-Liebig-University Giessen, Giessen, , Germany)
- Stark Helmut (Zentrum fur Zahn-Mund und Kieferheikunde der Universitat, Bonn, N/A, Germany)
- Huppertz Jan (Julius-Maximilians University of Würzburg, Würzburg, N/A, Germany)
- Hartmann Sinsa (Johannes Gutenberg University of Mainz, Mainz, , Germany)

SESSION INFORMATION
Oral Session
Clinical Studies in Prosthodontics
06/28/2014

Walter Michael (University of Dresden, Dresden, , Germany)
Jahn Florentine (Friedrich Schiller Universität Jena, Jena, N/A, Germany)
Strub Joerg R. (University Hospital, School of Dentistry, Freiburg, N/A, Germany)
Pospiech Peter (Julius-Maximilian University of Würzburg, Würzburg, , Germany)
Brückner Julian (University of Leipzig, Leipzig, , Germany)
Wolfart Stefan (University Hospital RWTH Aachen, Aachen, , Germany)
Busche Eckhard (Witten/Herdecke University, Faculty of Health, Witten, N/A, Germany)
Luthardt Ralph (Ulm University, Center of Dentistry, Ulm, , Germany)
Heydecke Guido (University Medical Center Hamburg-Eppendorf, Hamburg, N/A, Germany)
Marré Birgit (Technische Universität Dresden, Dresden, , Germany)
Hannak Wolfgang Bernd (Charité-Universitätsmedizin Berlin, Berlin, N/A, Germany)
Kern Matthias (Christian-Albrechts University, Kiel, , Germany)
Mundt Torsten (University of Greifswald, Greifswald, N/A, Germany)
Gernet Wolfgang (University of Munich, Munich, N/A, Germany)
Wöstmann Bernd (Justus-Liebig-University Giessen, Giessen, , Germany)
Stark Helmut (Zentrum fur Zahn-Mund und Kieferheikunde der Universitat, Bonn, N/A, Germany)
Huppertz Jan (Julius-Maximilians University of Würzburg, Würzburg, N/A, Germany)
Hartmann Sinsa (Johannes Gutenberg University of Mainz, Mainz, , Germany)

Clin Oral Investig. 2014;18(2):525-33. doi: 10.1007/s00784-013-0991-6. Epub 2013 May 17.

The randomized shortened dental arch study: oral health-related quality of life.

Wolfart S[1], Müller F, Gerß J, Heyedcke G, Marré B, Böning K, Wöstmann B, Kern M, Mundt T, Hannak W, Brückner J, Passia N, Jahn F, Hartmann S, Stark H, Richter EJ, Gernet W, Luthardt RG, Walter MH.

⊕ Author information

Abstract

OBJECTIVES: Although the shortened dental arch (SDA) concept is a widely accepted strategy to avoid overtreatment, little is known on its impact on oral health-related quality of life (OHRQoL). This multicenter randomized controlled trial aimed to investigate the OHRQoL for removable partial dental prostheses (RPDP) with molar replacement versus the SDA concept.

MATERIAL AND METHODS: In both groups, missing anterior teeth were replaced with fixed dental prosthesis. Two hundred fifteen patients with bilateral molar loss in at least one jaw were included. The Oral Health Impact Profile (OHIP-49) was completed before; 6 weeks (baseline), 6 months, and 12 months after treatment; and thereafter annually until 5 years.

RESULTS: Of the initial cohort, 81 patients were assigned to the RPDP group and 71 to the SDA group (age, 34 to 86 years). Before treatment, the median OHIP score was similar in both groups (RPDP, 38.0; SDA, 40.0; n.s.). Results indicate marked improvements in OHRQoL in both groups between pretreatment and baseline (RPDP, 27.0; SDA, 19.0; $p \leq 0.0001$) which continued in the RPDP group until the 1-year follow-up ($p = 0.0002$). These significant reductions in OHIP scores are reflected in its subscales. No further differences were seen within and between groups during the remainder observation period.

CONCLUSION: Both treatments show a significant improvement in OHRQoL which continued in the RPDP group until the 1-year follow-up. No significant differences were seen between groups.

CLINICAL RELEVANCE: For improving OHRQoL, it is not necessary to replace missing molars with a RPDP.

PMID: 23680969 DOI: 10.1007/s00784-013-0991-6

2015

3-year Randomized Controlled Prospective Clinical Trial on Different CAD-CAM Implant Abutments

Objectives: The study aimed at assessing the survival of implant crowns supported by CAD-CAM abutments after 3 years of service. The null hypothesis stated that there were no differences in the survival rates.
Methods: Fourty-eight patients were selected for this prospective clinical study (Clinicaltrials.gov #NCT02090647).
Each patient received at least 1 titanium dental implant (Osseospeed, Astra Tech Dental) for a total of 90 fixtures. A two-stage surgical technique and no additional soft or hard tissue graft were planned. The surgical exposure of the implants was carried on between 3-6 months after fixture insertion and a trans-mucosal healing abutment was screwed onto each implant.
The implants were randomly divided into 3 groups of 30 samples receiving different abutments (Atlantis, Astra Tech Dental), as follows: Group 1. titanium; Group 2, titanium nitride; Group 3, zirconia. The abutments were customized by a CAD-CAM system.
Zirconia (Aadva, GC) or metal-ceramic crowns were used as final restorations.
Cementation was the baseline and the restorations were checked after 6 months, 1, 2 and 3 years, assessing any mechanical complication. The 3-year success rate was statistically analyzed.
Results: Five failures were reported in the zirconia group; all the failed restorations showed fractures of the abutment connection. Four failures occurred in posterior restorations. One more fracture occurred while screwing the abutment onto the fixture.
The statistical analysis showed that titanium and titanium nitride abutments had a significantly higher 3-year success rate than zirconia abutments.
Conclusions: Titanium and titanium nitride abutments showed optimal clinical performances after 3 years of clinical service. Conversely, zirconia abutments should be avoided to restore posterior regions.

Division: IADR/AADR/CADR General Session
Meeting: 2015 IADR/AADR/CADR General Session (Boston, Massachusetts)
Location: Boston, Massachusetts
Year: 2015
Final Presentation ID: 0453

Authors

- Ferrari Marco (Tuscan School of Dental Medicine , Livorno Livorno , Italy)
- Cagidiaco Mc (Tuscan School of Dental Medicine , Livorno Livorno , Italy)
- Nappo Antonio (Tuscan School of Dental Medicine , Livorno Livorno , Italy)
- Sorrentino Roberto (University "Federico II" , Napoli Napoli , Italy ; Second University of Naples , Aversa Aversa , Italy)
- Zarone Fernando (University "Federico II" , Napoli Napoli , Italy)
- Gherlone Enrico (University Vita Salute San Raffaele , Milano Milano , Italy)
- Goracci Cecilia (Tuscan School of Dental Medicine , Livorno Livorno , Italy)
- Vichi Alessandro (Tuscan School of Dental Medicine , Livorno Livorno , Italy)

Financial Interest Disclosure: NONE

SESSION INFORMATION
Oral Session
Clinical Prosthodontics Research
Thursday, 03/12/2015 , 08:00AM - 09:30AM

3-year Randomized Controlled Prospective Clinical Trial on Different CAD-CAM Implant Abutments

CLINICAL IMPLANT DENTISTRY
and Related Research

ARTICLE

3-Year Randomized Controlled Prospective Clinical Trial on Different CAD-CAM Implant Abutments

Marco Ferrari MD, DDS, PhD, Maria Gabriella Tricarico DDS, Maria Crysanti Cagidiaco MD, DDS, PhD, Alessandro Vichi DDS, MSc, PhD, Enrico Felice Gherlone MD, DDS, Fernando Zarone MD, DDS, Roberto Sorrentino DDS, MSc, PhD

First published: 14 March 2016 | https://doi.org/10.1111/cid.12418 | Cited by: 4

Read the full text >

PDF TOOLS SHARE

Abstract

Background

Zirconia abutments were introduced to restore esthetic regions and showed sufficient stability to support implant restorations. Nonetheless, to date the observation periods are shorter than those of titanium abutments.

Purpose

To assess the survival of implant crowns supported by computer aided design-computer aided manufacturing (CAD-CAM) abutments after 3 years.

Materials and Methods

Fifty-six patients were selected for this prospective clinical study. Each patient received at least 1 titanium implant for a total of 89 fixtures. A two-stage surgical technique and no additional soft or hard tissue graft were used. The implants were randomly divided into 3 groups receiving different CAD-CAM abutments: titanium, titanium nitride, and zirconia. Zirconia or metal-ceramic crowns were used as final restorations. Cementation was the baseline and the restorations were checked after 6 months, 1, 2, and 3 years, assessing any mechanical complication. Statistical analyses were performed to evaluate the 3-year success rates.

Results

Five failures were reported in the zirconia group; all the failed restorations showed fractures of the abutment connection. Four failures occurred in posterior regions and one more occurred while screwing the abutment. Titanium and titanium nitride abutments had significantly higher 3-year success rates than zirconia abutments ($p < .05$).

Conclusions

Atlantis titanium and titanium nitride abutments showed optimal clinical performances after 3 years. Conversely, Atlantis zirconia abutments should be avoided to restore posterior regions.

A Fractographic Study of Clinically Retrieved Zirconia-Ceramic and Metal-Ceramic FPDs

Objectives: A recent 3-year clinical study of randomized controlled three- to five-unit metal-ceramic and zirconia-ceramic fixed partial dentures (FPDs) revealed that veneer chipping and fracture in zirconia-ceramic systems occurred more frequently than those in metal-ceramic systems. This study seeks to elucidate the underlying mechanisms responsible for this clinical observation using a quantitative fractographic analysis.

Methods: Thirty-six zirconia-ceramic and thirty-one metal-ceramic FPDs were randomly placed in fifty-nine patients who had good general and periodontal health with no obvious signs of bruxism. Impressions of FPDs were taken from the patients at the end of a mean observation period of 40.3 ± 2.8 months. Epoxy replicas were produced from these impressions. All replicas were gold coated, and inspected under the optical microscope and scanning electron microscope (SEM). Fracture origin, fracture path, damage modes, and stress states were determined. Critical load for the onset of fracture was estimated based on the principles of fracture mechanics.

Results: Among 31 metal-ceramic FPDs, 25 survived, 5 had small chippings and 1 had large chipping. Out of 36 zirconia-ceramic FPDs, 24 survived, 2 had small chippings, 9 had large chippings, and 1 exhibited delamination. Detailed SEM examinations revealed that fracture initiated from the wear facet at the occlusal surface in all cases, irrespective of the type of restoration.

Conclusions: Metal-ceramic and zirconia-ceramic FPDs all fractured from veneer chipping with the origin of fracture located in an occlusal wear facet. The relatively low fracture toughness and high residual tensile stress in the porcelain veneer of zirconia-based restorations contributed to the higher chipping rate and larger chip size in zirconia-ceramic FPDs relative to their metal-ceramic counterparts. The low veneer/core interfacial toughness of porcelain-veneered zirconia resulted in a higher incidence of delamination in zirconia-ceramic FPDs relative to metal-ceramic FPDs.

Division: IADR/AADR/CADR General Session
Meeting: 2015 IADR/AADR/CADR General Session (Boston, Massachusetts)
Location: Boston, Massachusetts
Year: 2015
Final Presentation ID: 3535

Authors

- Pang Zhen (New York University , New York New York , New York , United States)
- Chughtai Asima (New York University , New York New York , New York , United States)
- Sailer Irena (University of Geneva , Geneva Geneva , Switzerland)
- Zhang Yu (New York University , New York New York , New York , United States)

Pang Zhen (New York University , New York New York , New York , United States)
Chughtai Asima (New York University , New York New York , New York , United States)
Sailer Irena (University of Geneva , Geneva Geneva , Switzerland)
Zhang Yu (New York University , New York New York , New York , United States)

Support Funding Agency/Grant Number: NIH/NIDCR 2R01 DE017925
Financial Interest Disclosure: None

SESSION INFORMATION
Poster Session
Clinical Aspects of Ceramic Restorations
Saturday, 03/14/2015 , 02:00PM - 03:15PM

A Fractographic Study of Clinically Retrieved Zirconia-Ceramic and Metal-Ceramic FPDs

ELSEVIER

Dental Materials

Volume 31, Issue 10, October 2015, Pages 1198-1206

A fractographic study of clinically retrieved zirconia–ceramic and metal–ceramic fixed dental prostheses

Zhen Pang [a], Asima Chughtai [a], Irena Sailer [b], Yu Zhang [a]

⊞ Show more

https://doi.org/10.1016/j.dental.2015.07.003

Get rights and content

Abstract

Objectives

A recent 3-year randomized controlled trial (RCT) of tooth supported three- to five-unit zirconia–ceramic and metal–ceramic posterior fixed dental prostheses (FDPs) revealed that veneer chipping and fracture in zirconia–ceramic systems occurred more frequently than those in metal–ceramic systems [1]. This study seeks to elucidate the underlying mechanisms responsible for the fracture phenomena observed in this RCT using a descriptive fractographic analysis.

Methods

Vinyl-polysiloxane impressions of 12 zirconia–ceramic and 6 metal–ceramic FDPs with veneer fractures were taken from the patients at the end of a mean observation of 40.3 ± 2.8 months. Epoxy replicas were produced from these impressions [1]. All replicas were gold coated, and inspected under the optical microscope and scanning electron microscope (SEM) for descriptive fractography.

Results

Among the 12 zirconia–ceramic FDPs, 2 had small chippings, 9 had large chippings, and 1 exhibited delamination. Out of 6 metal–ceramic FDPs, 5 had small chippings and 1 had large chipping. Descriptive fractographic analysis based on SEM observations revealed that fracture initiated from the wear facet at the occlusal surface in all cases, irrespective of the type of restoration.

Significance

Zirconia–ceramic and metal–ceramic FDPs all fractured from microcracks that emanated from occlusal wear facets. The relatively low fracture toughness and high residual tensile stress in porcelain veneer of zirconia restorations may contribute to the higher chipping rate and larger chip size in zirconia–ceramic FDPs relative to their metal–ceramic counterparts. The low veneer/core interfacial fracture energy of porcelain-veneered zirconia may result in the occurrence of delamination in zirconia–ceramic FDPs.

Abutment Color and Gingival Thickness Influence on Soft Tissue Color

Objectives: Zirconia (Zir) and Titanium nitride (TiN) prosthetic abutments have been introduced mainly for aesthetic purposes, as Titanium gray color could be seen through the gingival tissue. Aim of the study was to assess whether Zir and TiN abutments yielded better aesthetics of gingival tissues in comparison with Titanium (Ti) abutments.
Methods: Ninety patients were included in the study. Each patient received an implant (Osseospeed, Astra Tech). A two-stage surgical technique was performed. Six months later re-entry was performed. After 1 week, a temporary restoration was placed. After 8 weeks, an implant level impression was taken and soft tissue dimensions was recorded, ranking thickness into thin (⩽2mm) and thick (>2mm). Patients were randomly allocated to one of the three groups, based on the abutment type: TiN,Ti,Zir. After 15 weeks, definitive restoration was applied. Gingival area of abutment was measured for color by the use of a Clinical Spectrophotomer (Easyshade, VITA). Measurement of contralateral area with natural dentition was performed at the same time. Data were collected in CIELab* color system. ΔE was calculated between implant and contralateral soft tissue. A critical threshold of ΔE=3.7 was selected. The Chi-square test was applied to identify statistically significant differences in ΔE between thin and thick gingiva and among the abutment types.
Results: 3 patients were lost to follow-up. No statistically significant differences in DE emerged among the abutment types (p=0.966). A statistically significant difference in ΔE emerged between thick and thin gingiva (p<0.001).
Conclusions: The three abutment types performed similarly in terms of influence on soft tissue color. Concerning with gingival thickness, when it was ⩽ 2mm all the measurements of the three types of abutments influenced the color of the gingival tissue. Conversely, when gingival tissue thickness was >2mm, only 2 of 64 measurements influenced the color beyond ΔE clinical threshold.

Division: IADR/AADR/CADR General Session
Meeting: 2015 IADR/AADR/CADR General Session (Boston, Massachusetts)
Location: Boston, Massachusetts
Year: 2015
Final Presentation ID: 0107

Authors

- Vichi Alessandro (University of Siena , Siena Siena , Italy)
- Carrabba Michele (University of Siena , Siena Siena , Italy)
- Nappo Antonio (University of Siena , Siena Siena , Italy)
- Goracci Cecilia (University of Siena , Siena Siena , Italy)
- Ferrari Marco (University of Siena , Siena Siena , Italy)

Financial Interest Disclosure: NONE

SESSION INFORMATION
Oral Session
Gingiva and Dental Materials
Wednesday, 03/11/2015 , 01:30PM - 03:00PM

Int J Oral Maxillofac Implants. 2017 Mar/Apr;32(2):393–399. doi: 10.11607/jomi.4794. Epub 2016 Aug 15.

Influence of Abutment Color and Mucosal Thickness on Soft Tissue Color.

Ferrari M, Carrabba M, Vichi A, Goracci C, Cagidiaco MC.

Abstract

PURPOSE: Zirconia (ZrO_2) and titanium nitride (TiN) implant abutments were introduced mainly for esthetic purposes, as titanium's gray color can be visible through mucosal tissues. This study was aimed at assessing whether ZrO_2 and TiN abutments could achieve better esthetics in comparison with titanium (Ti) abutments, regarding the appearance of soft tissues.

MATERIALS AND METHODS: Ninety patients were included in the study. Each patient was provided with an implant (OsseoSpeed, Dentsply Implant System). A two-stage surgical technique was performed. Six months later, surgical reentry was performed. After 1 week, provisional restorations were screwed onto the implants. After 8 weeks, implant-level impressions were taken and soft tissue thickness was recorded, ranking thin (≤ 2 mm) or thick (≥ 2 mm). Patients were randomly allocated to three experimental groups, based on abutment type: (1) Ti, (2) TiN, and (3) ZrO_2. After 15 weeks, the final restorations were delivered. The mucosal area referring to each abutment was measured for color using a clinical spectrophotometer (Easyshade, VITA); color measurements of the contralateral areas referring to natural teeth were performed at the same time. The data were collected using the Commission Internationale de l'Eclairage (CIE) L*a*b* color system, and ΔE was calculated between peri-implant and contralateral soft tissues. A critical threshold of $\Delta E = 3.7$ was selected. The chi-square test was used to identify statistically significant differences in ΔE between thin and thick mucosal tissues and among the abutment types.

RESULTS: Three patients were lost at follow-up. No statistically significant differences were noticed as to the abutment type ($P = .966$). Statistically significant differences in ΔE were recorded between thick and thin peri-implant soft tissues ($P < .001$). Only 2 out of 64 patients with thick soft tissues showed a ΔE higher than 3.7: 1 in the TiN group and 1 in the ZrO_2 group. All the patients with thin soft tissues reported color changes that exceeded the critical threshold.

CONCLUSION: The different abutment materials showed comparable results in terms of influence on soft tissue color. Regarding peri-implant soft tissue thickness, the influence of the tested abutments on soft tissue color became clinically relevant for values ≤ 2 mm.

PMID: 27525517 DOI: 10.11607/jomi.4794

Clinical Comparison of a Flowable to Conventional Resin Composite

Clinical Comparison of a Flowable to Conventional Resin Composite

Objectives: To evaluate the 2-year clinical performance and volumetric wear of a flowable resin composite to conventional highly filled composite resin in Class I restorations.
Methods: In this single-center, single-blinded, comparator-controlled clinical study (IRB approved), 120 carious teeth distributed in 60 patients were randomly assigned to 4 calibrated practitioners who placed occlusal restorations (n=60 Filtek Supreme Ultra Flowable; n=60 Filtek Supreme Ultra Universal, 3M ESPE Dental Products). Following Class I preparation and 15s etch, Adper Single Bond Plus Adhesive (3M ESPE) was applied, followed by 2mm composite increments cured for 20 seconds/increment with rubber dam isolation. Direct and indirect assessment at baseline, 6-months, 1-year, and 2-years occurred evaluating anatomical form, color match, marginal adaptation, marginal discoloration, surface integrity, secondary caries, and occlusal/cold sensitivity. Volumetric wear was determined by superimposition of 3D light profilometer scans (Proscan 2000, Scantron Industrial Products, Taunton, England) of baseline and 2-year casts. After 2 years, 94 restorations remained in the study. Groups were compared with repeated measures ANOVA (alpha=.05) with Fishers Exact test and volumetric wear was compared with a Mann-Whitney test (alpha=.05).
Results: At 2 years, there was no significant difference in anatomic form (p=.80), color match (p=.08), marginal adaptation (p=.89), marginal discoloration (p=.79), surface integrity (p=.18), secondary caries (p=.66), cold sensitivity (p=.522), occlusal sensitivity (p=.818), or volumetric wear (p=.661) between materials. Both materials showed a decrease all criteria except secondary caries (p=.95) over time. Two-year mean volumetric wear was 3.16 ± 2.38 mm^3 for the flowable composite and 3.43 ± 2.50 mm^3 for the universal composite.
Conclusions: The flowable and universal composites used in this study have similar clinical efficacy after 2 years of service when placed as Class I occlusal restorations having isthmus widths less than one-half the intercuspal distance.

This study was supported in part by 3M ESPE Dental Products, Minneapolis, MN.

Year: 2015
Final Presentation ID: 3269

Authors

- Givan, Daniel (University of Alabama at Birmingham , Birmingham Birmingham , Alabama , United States)
- Lawson, Nathaniel (University of Alabama at Birmingham , Birmingham Birmingham , Alabama , United States)
- Radhakrishnan, Rashmi (University of Alabama at Birmingham , Birmingham Birmingham , Alabama , United States)
- Fu, Chin-chaun (University of Alabama at Birmingham , Birmingham Birmingham , Alabama , United States)
- Robles, Augusto (University of Alabama at Birmingham , Birmingham Birmingham , Alabama , United States)
- Ramp, Lance (University of Alabama at Birmingham , Birmingham Birmingham , Alabama , United States)
- Cakir, Deniz (University of Alabama at Birmingham , Birmingham Birmingham , Alabama , United States)
- Burgess, John (University of Alabama at Birmingham , Birmingham Birmingham , Alabama , United States)

Givan, Daniel (University of Alabama at Birmingham , Birmingham Birmingham , Alabama , United States)
Lawson, Nathaniel (University of Alabama at Birmingham , Birmingham Birmingham , Alabama , United States)
Radhakrishnan, Rashmi (University of Alabama at Birmingham , Birmingham Birmingham , Alabama , United States)
Fu, Chin-chaun (University of Alabama at Birmingham , Birmingham Birmingham , Alabama , United States)
Robles, Augusto (University of Alabama at Birmingham , Birmingham Birmingham , Alabama , United States)
Ramp, Lance (University of Alabama at Birmingham , Birmingham Birmingham , Alabama , United States)
Cakir, Deniz (University of Alabama at Birmingham , Birmingham Birmingham , Alabama , United States)
Burgess, John (University of Alabama at Birmingham , Birmingham Birmingham , Alabama , United States)

Support Funding Agency/Grant Number: 3M ESPE Dental Products
Financial Interest Disclosure: None

SESSION INFORMATION
Oral Session
Clinical Trials: Adhesives and Composites
Saturday, 03/14/2015 , 08:00AM - 09:30AM

Clinical Comparison of a Flowable to Conventional Resin Composite

Oper Dent. 2015 Nov-Dec;40(6):594-602. doi: 10.2341/15-038-C. Epub 2015 Aug 3.

Two-year Randomized, Controlled Clinical Trial of a Flowable and Conventional Composite in Class I Restorations.

Lawson NC, Radhakrishnan R, Givan DA, Ramp LC, Burgess JO.

Abstract

OBJECTIVES: This study evaluated the two-year clinical performance and volumetric wear of a flowable resin composite compared to a conventional highly filled composite resin in Class I restorations.

METHODS AND MATERIALS: In this single-center, single-blinded, comparator-controlled clinical study (Institutional Review Board approved), 120 carious teeth distributed in 60 patients were randomly assigned to four calibrated practitioners who placed occlusal restorations (n=60 flowable and n=60 conventional composite). Direct and indirect assessment at baseline, six months, one year, and two years occurred during which the modified Cvar and Ryge criteria were evaluated. Volumetric wear was determined by superimposition of profilometer scans of baseline and two-year casts.

RESULTS: At two years, there was no significant difference in anatomic form (p=0.80), color match (p=0.08), marginal adaptation (p=0.89), marginal discoloration (p=0.79), surface integrity (p=0.18), secondary caries (p=0.66), cold sensitivity (p=0.522), occlusal sensitivity (p=0.818), or volumetric wear (p=0.661) between materials. Both materials showed a decrease in all criteria except secondary caries (p=0.95) over time. Two-year mean volumetric wear was 3.16 ± 2.38 mm(3) for the flowable composite and 3.43 ± 2.50 mm(3) for the conventional composite.

CONCLUSIONS: The flowable and conventional composites used in this study have similar clinical efficacy after two years of service when placed as Class I occlusal restorations having isthmus widths less than one-half the intercuspal distance.

PMID: 26237643 DOI: 10.2341/15-038-C

Complications of Zirconia Single Crowns: a 3-year Randomised Prospective Clinical Trial

Objectives: The present randomised prospective clinical trial aimed at evaluating the influence of the coping design on the fracture resistance of CAD-CAM zirconia-ceramic crowns onto teeth after 3 years of service.

Methods: Fifty-six patients in need to receive at least 1 posterior zirconia single crown were selected (Clinicaltrials.gov #NCT020906567) for a total of 90 abutments. Standardized tooth preparations with a supragingival chamfer finish line were performed. Precision impressions were taken using vinyl-polyether-silicone materials (EXA'lence, GC).

The teeth were randomly divided into 3 groups of 30 samples each and received different coping designs, as follows: Group 1, flat design (FD); Group 2, porcelain-fused-to-metal-like design (PFM); Group 3, anatomically-guided design (AG). The wax-up of each coping was scanned with a CAD-CAM software (Aadva, GC) to produce the zirconia cores that were layered with a dedicated ceramic.

The crowns were cemented with resin cement (G-CEM, GC). Cementation was the baseline and mechanical complications were evaluated after 6 months, 1, 2 and 3 years. The 3-year success rate was statistically analyzed.

Results: After 3 years, Group 1 showed 6 chippings (80% success rate) while no complication was recorded in Groups 2 and 3 (100% success rate). No chipping impaired function and no crown was replaced.

The statistical analyses showed significantly higher 3-year success rates in Groups 2 and 3.

Conclusions: Zirconia copings with PFM and AG designs performed clinically better than FD crowns. A proper support to the veneering ceramic by a correct coping design could reduce the risk of chipping during function. The FD should be avoided in daily practice.

Division: IADR/AADR/CADR General Session
Meeting: 2015 IADR/AADR/CADR General Session (Boston, Massachusetts)
Location: Boston, Massachusetts
Year: 2015
Final Presentation ID: 0451

Authors

- Ferrari Marco (Tuscan School of Dental Medicine , Siena Siena , Italy)
- Cagidiaco Mc (Tuscan School of Dental Medicine , Siena Siena , Italy)
- Nappo Antonio (Tuscan School of Dental Medicine , Siena Siena , Italy)
- Sorrentino Roberto (University "Federico II" , Napoli Napoli , Italy ; Second University of Naples , Aversa Aversa , Italy)
- Zarone Fernando (University "Federico II" , Napoli Napoli , Italy)
- Bosco Mario (University of Pavia , Pavia Pavia , Italy)
- Goracci Cecilia (Tuscan School of Dental Medicine , Siena Siena , Italy)
- Vichi Alessandro (Tuscan School of Dental Medicine , Siena Siena , Italy)

Ferrari Marco (Tuscan School of Dental Medicine , Siena Siena , Italy)
Cagidiaco Mc (Tuscan School of Dental Medicine , Siena Siena , Italy)
Nappo Antonio (Tuscan School of Dental Medicine , Siena Siena , Italy)
Sorrentino Roberto (University "Federico II" , Napoli Napoli , Italy ; Second University of Naples , Aversa Aversa , Italy)
Zarone Fernando (University "Federico II" , Napoli Napoli , Italy)
Bosco Mario (University of Pavia , Pavia Pavia , Italy)
Goracci Cecilia (Tuscan School of Dental Medicine , Siena Siena , Italy)
Vichi Alessandro (Tuscan School of Dental Medicine , Siena Siena , Italy)

Financial Interest Disclosure: NONE

SESSION INFORMATION
Oral Session
Clinical Prosthodontics Research
Thursday, 03/12/2015 , 08:00AM - 09:30AM

Complications of Zirconia Single Crowns: a 3-year Randomised Prospective Clinical Trial

Am J Dent. 2015 Aug;28(4):235-40.

Short-term clinical performance of zirconia single crowns with different framework designs: 3-year clinical trial.

Ferrari M, Sorrentino R, Cagidiaco C, Goracci C, Vichi A, Gherlone E, Zarone F.

Abstract

PURPOSE: The present prospective clinical study evaluated the influence of coping design on the fracture resistance of CAD/CAM zirconia single crowns layered with dedicated ceramics.

METHODS: 56 subjects were provided with 90 zirconia single crowns in posterior regions. Tooth preparations were standardized and the abutment teeth were randomly distributed into three groups, according to three different coping designs (flat design, FD; porcelain-fused-to-metal-like crowns, PFM; anatomically-guided, AG). The zirconia cores were produced using a CAD/CAM software and then were hand-layered with dedicated ceramics. All crowns were cemented with a self-adhesive resin luting agent and the patients were recalled for follow-up visits after 1 month, 6 months, 1, 2 and 3 years of clinical service. The function, esthetics and marginal adaptation of the restorations were evaluated. Statistical analyses were performed to evaluate survival and success of the restorations.

RESULTS: Success rates of 100% were reported in Group 2 and Group 3 while the percentage was 80% in Group 1. Three chippings were noticed in Group 1 (FD) and two crowns needed to be replaced after 3 years, resulting in a survival rate of 93.3%. Group 2 and Group 3 had significantly higher 3-year success rates than Group 1 ($P < 0.05$). Based on the present clinical results, the following conclusions were drawn: the porcelain-fused-to-metal-like and the anatomically-guided frameworks for zirconia single crowns performed better clinically than the flat designed cores in posterior regions after 3 years; standardized tooth preparations achieved even thicknesses of the bilayered restorations; the proper support given to the veneering ceramic by the correct design of the zirconia framework could significantly reduce the risk of chipping during function.

PMID: 26437506

Crown vs. Composite for Post-retained Restorations: up to 59-month Trial

Crown vs. Composite for Post-retained Restorations: up to 59-month Trial

Objectives: This randomized clinical trial compared the survival of composite resin restorations and metal-ceramic crowns on endodontically treated teeth that received a glass fiber post.

Methods: Forty-seven patients (age 42.5±11.5) with fifty-seven endodontically treated teeth severely damaged but with at least one entire wall were randomly allocated into two groups according to the type of coronal restoration: metal-ceramic crown or composite resin (Z250). A glass fiber post (White Post DC #1) was previously cemented in all teeth with regular (RelyX ARC) or self-adhesive (RelyX U100) resin cement. Descriptive analysis through FDI clinical criteria was performed and the longevity of restorations and teeth were analyzed using Kaplan-Meier statistics and log-rank tests.

Results: The recall rate was 100% for a time up to 59 months. From 30 composite restorations and 27 metal-ceramic crowns, one tooth was extracted after 11 months due to root fracture (composite group). Eight composite restorations and one crown had reparable failures, all due to secondary caries or restoration fracture. The general annual failure rate (AFR) was 0.92% after 50 months for success of the restorations, with 1.83% for the composite group and 0.26% for the metal-ceramic crown group. The log-rank test showed no difference for survival according to the type of restoration ($p=0.344$). However, for success rates, metal-ceramic crowns demonstrated better performance ($p=0.022$).

Conclusions: Indirect restorations provided higher acceptable clinical performance and lower need for reintervention after the follow-up time. Still, both types of restorations presented good survival rates. (NCT01461239)

Division: IADR/AADR/CADR General Session
Meeting: 2015 IADR/AADR/CADR General Session (Boston, Massachusetts)
Location: Boston, Massachusetts
Year: 2015
Final Presentation ID: 0514

Authors

- Skupien Jovito (Federal University of Pelotas , Pelotas Pelotas , RS , Brazil)
- Cenci Maximiliano (Federal University of Pelotas , Pelotas Pelotas , RS , Brazil)
- Opdam N.j.m. (Radboud University Nijmegen Medical Centre , Nijmegen Nijmegen , Netherlands)
- Kreulen Cees (Radboud University Nijmegen Medical Centre , Nijmegen Nijmegen , Netherlands)
- Huysmans Marie (Radboud University Nijmegen Medical Centre , Nijmegen Nijmegen , Netherlands)
- Pereira Cenci Tatiana (Federal University of Pelotas , Pelotas Pelotas , RS , Brazil)

Skupien Jovito (Federal University of Pelotas , Pelotas Pelotas , RS , Brazil)
Cenci Maximiliano (Federal University of Pelotas , Pelotas Pelotas , RS , Brazil)
Opdam N.j.m. (Radboud University Nijmegen Medical Centre , Nijmegen Nijmegen , Netherlands)
Kreulen Cees (Radboud University Nijmegen Medical Centre , Nijmegen Nijmegen , Netherlands)
Huysmans Marie (Radboud University Nijmegen Medical Centre , Nijmegen Nijmegen , Netherlands)
Pereira Cenci Tatiana (Federal University of Pelotas , Pelotas Pelotas , RS , Brazil)

Support Funding Agency/Grant Number: CAPES/NUFFIC 026/11
Financial Interest Disclosure: none

SESSION INFORMATION
Oral Session
Clinical Trials: Miscellaneous
Thursday, 03/12/2015 , 10:45AM - 12:15PM

Crown vs. Composite for Post-retained Restorations: up to 59-month Trial

Journal of Dentistry
Volume 48, May 2016, Pages 34-39

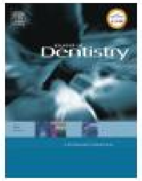

Crown vs. composite for post-retained restorations: A randomized clinical trial ☆

Jovito Adiel Skupien [a], Maximiliano Sérgio Cenci [a], Niek Johannes Opdam [b], C.M. Kreulen [c], Marie-Charlotte Huysmans [b], Tatiana Pereira-Cenci [a]

Show more

https://doi.org/10.1016/j.jdent.2016.03.007

Get rights and content

Abstract

Objectives

This randomized clinical trial compared the survival of composite resin restorations and metal-ceramic crowns on endodontically treated teeth that received a glass fiber post using 2 different cementation methods.

Methods

Forty-seven patients (age 42.5 ± 11.5) with fifty-seven endodontically treated teeth with extensive coronal damage but always with one intact surface were randomly allocated according to the type of coronal restoration: metal-ceramic crown or composite resin. In case of crown restoration, a core buildup was performed with microhybrid composite resin. The dentin bonding agent and composite resin used were the same for both direct and indirect restorations. Descriptive analysis was performed using FDI clinical criteria and survival of restorations/teeth analyzed using Kaplan-Meier statistics and log-rank tests.

Results

57 restorations (30 composite resin and 27 crowns) were made in 47 patients. The recall rate was 100% and follow up time ranged between 1 and 5 years. One tooth was extracted 11 months post-restoration due to root fracture (composite group). Eight composite restorations and one crown had reparable failures, all due to secondary caries or restoration fracture. The overall annual failure rate (AFR) was 0.92% after 50 months for success of the restorations, with 1.83% for the composite group and 0.26% for the metal-ceramic crown group. The log-rank test showed no difference for survival according to the type of restoration ($p = 0.344$). However, for success rates, metal-ceramic crowns demonstrated better performance ($p = 0.022$).

Conclusions

Indirect restorations provided higher acceptable clinical performance and lower need for re-intervention, but both types of restorations presented good survival rates. (NCT01461239).

Clinical significance

When endodontically treated teeth with at least one intact surface must be restored, composite resin restorations and metal-ceramic crows are acceptable alternatives to achieve good survival and success rates.

Effect of Different Prosthetic Abutments on Peri-Implant Soft Tissue: A Randomized Clinical Trial

Effect of Different Prosthetic Abutments on Peri-Implant Soft Tissue: A Randomized Clinical Trial

Objectives: This randomized clinical trial was aimed at assessing the effect of three different prosthetic abutments (titanium, titanium nitrurate, zirconia) on peri-implant soft tissue two years after treatment in partially edentulous patients.
Methods: A sample of 37 patients (17 males, 20 females) with a total of 71 implants was collected. Patients were randomly allocated to receive one of the three abutment types: Group 1 (n=9): titanium; Group 2 (n=20): titanium nitrurate; Group 3 (n=8): zirconia (Atlantis, Dentsply). Before abutment connection the following clinical measurements were taken: keratinized tissue thickness of the buccal peri-implant soft tissue at the level of the implant neck, soft tissue thickness above the bone crest, depth/length of transmucosal pathway, periodontal biotype at adjacent teeth. The final prosthetic restoration was an implant-supported single crown or bridge cemented on a customized abutment (Atlantis). At the time of placement of definitive restoration the following clinical measurements were taken: recession depth at the central buccal site, probing depth at 6 points/tooth, keratinized tissue thickness at the middle buccal point. Measurements were repeated after 2 years of clinical service. A two-level (patient, implant) statistical model was applied.
Results: At the 2-year clinical observation recession of the gingival margin was observed only at 13% of implants irrespective of the type of abutment. No significant correlation between periodontal biotype at adjacent teeth and peri-implant biotype was observed. None of the investigated variables at patient level (age, gender, implant type, periodontal biotype) or at implant level (keratinized tissue thickness, probing depth, soft tissue thickness) was identified as significant predictor of recession.
Conclusions: Abutment type did not influence peri-implant variables after 2 years. Caution should be used in considering periodontal biotype at patient level as a possible indicator of the future peri-implant biotype.

Division: IADR/AADR/CADR General Session
Meeting: 2015 IADR/AADR/CADR General Session (Boston, Massachusetts)
Location: Boston, Massachusetts
Year: 2015
Final Presentation ID: 0985

Authors

- Ferrari Marco (University of Siena , Siena Siena , Italy)
- Cagidiaco Maria Crysanti (University of Siena , Siena Siena , Italy)
- Nappo Antonio (University of Siena , Siena Siena , Italy)
- Goracci Cecilia (University of Siena , Siena Siena , Italy)
- Cairo Francesco (University of Siena , Siena Siena , Italy)

Ferrari Marco (University of Siena , Siena Siena , Italy)
Cagidiaco Maria Crysanti (University of Siena , Siena Siena , Italy)
Nappo Antonio (University of Siena , Siena Siena , Italy)
Goracci Cecilia (University of Siena , Siena Siena , Italy)
Cairo Francesco (University of Siena , Siena Siena , Italy)

Financial Interest Disclosure: NONE

SESSION INFORMATION
Poster Session
Clinical and Biological Research, Implant Prosthodontics
Thursday, 03/12/2015 , 03:30PM - 04:45PM

Effect of Different Prosthetic Abutments on Peri-Implant Soft Tissue: A Randomized Clinical Trial

Am J Dent. 2015 Apr;28(2):85-9.

Effect of different prosthetic abutments on peri-implant soft tissue. A randomized controlled clinical trial.

Ferrari M, Cagidiaco MC, Garcia-Godoy F, Goracci C, Cairo F.

Abstract

PURPOSE: This randomized clinical trial assessed the effect of three different prosthetic abutments (titanium, gold-hue titanium and zirconia) on peri-implant soft tissue 2 years after treatment in partially edentulous subjects.

METHODS: Baseline data concerning (1) thickness of the buccal peri-implant soft tissue, (2) soft tissue thickness above the bone crest, (3) depth/length of transmucosal pathway, and (4) periodontal biotype at adjacent teeth were collected. The final sample consisted of 47 subjects (21 males, 26 females) with a total of 97 implants. A two-level (patient, implant) statistical model was applied.

RESULTS: At the 2-year clinical observation, recession of the gingival margin was observed only at 13% of implants irrespective of the type of abutment. No significant correlation between periodontal biotype at adjacent teeth and peri-implant biotype was observed. Furthermore, none of the investigated variables at patient level (age, gender, implant type, periodontal biotype) or at implant level (keratinized tissue thickness, probing depth, soft tissue thickness) was identified as a significant predictor of recession. In conclusion, this study pointed out that (1) abutment type was not able to influence peri-implant variables after 2 years, and (2) caution should be used in considering periodontal biotype at patient level as a possible indicator of the future peri-implant biotype.

PMID: 26087573

Flap vs. Flapless Immediate Implant Placement- A Randomized Controlled Trial

Flap vs. Flapless Immediate Implant Placement- A Randomized Controlled Trial

Objectives: We carried out a randomized controlled trial of 6-month duration to compare gingival margin location (GM), buccal horizontal ridge dimensions, and interproximal crestal bone levels following two surgical approaches for immediate placement of implants in the esthetic zone: one involving flap elevation (FE) and another using a flapless protocol (FL).
Methods: Thirty-nine patients with a single non-restorable tooth in the anterior maxilla had their tooth extracted. Patients having an intact buccal plate or < 5mm fenestration post-extraction were randomized to receive a dental implant using either a FE or a FL protocol. We used clinical measurements to assess GM changes from fixed reference points, obtained standardized radiographs using bite-plates to assess bone changes, and used digitized cast overlays to assess buccal ridge dimensional changes.
Results: The mean change in GM between the groups at 6 months (follow-up minus baseline, FL vs. FE, in mm) was 0.25 vs. -0.13 at the mid-buccal site (p=0.031), 0.50 vs. -0.05 at the mesio-buccal site (p=0.003), and 0.25 vs. 0.03 at the disto-buccal site (p=0.031).
Interproximal bone level changes at 6 months were -0.31 vs. -0.37mm at the mesial surface (p=0.794), and -0.44 vs. -0.82mm at the distal surface (p=0.178), in the FL and FE groups, respectively.
Buccal horizontal ridge dimensional changes (follow-up minus pre-extraction) were -0.64 vs. -0.54mm during the first 3 months (p=0.617) followed by an additional -0.18 vs. -0.19mm during the subsequent 3 months (p=0.968) in the FL and FE groups, respectively.
Conclusions: Flap elevation resulted in <0.2mm mean recession of the gingival margin at the mid-buccal and mesio-buccal aspects of the implants at 6 months. No statistically significant differences in buccal ridge dimensions or interproximal crestal bone remodeling were detected between the groups. From a clinical standpoint, the differences between the groups were of questionable significance.

Division: IADR/AADR/CADR General Session
Meeting: 2015 IADR/AADR/CADR General Session (Boston, Massachusetts)
Location: Boston, Massachusetts
Year: 2015
Final Presentation ID: 0264

Authors

- Stoupel Janet (Columbia University , New York New York , New York , United States)
- Lee Chun-teh (Columbia University , New York New York , New York , United States)
- Glick Jaclyn (Columbia University , New York New York , New York , United States)
- Sanz Miralles Elena (Columbia University , New York New York , New York , United States)
- Chiuzan Corduta (Columbia University Mailman School of Public Health , New York New York , New York , United States)
- Papapanou Panos (Columbia University , New York New York , New York , United States)

Stoupel Janet (Columbia University , New York New York , New York , United States)
Lee Chun-teh (Columbia University , New York New York , New York , United States)
Glick Jaclyn (Columbia University , New York New York , New York , United States)
Sanz Miralles Elena (Columbia University , New York New York , New York , United States)
Chiuzan Corduta (Columbia University Mailman School of Public Health , New York New York , New York , United States)
Papapanou Panos (Columbia University , New York New York , New York , United States)

Financial Interest Disclosure: None of the authors have any conflict of interest in conjunction with this study.

SESSION INFORMATION
Oral Session
Implant Surgery Factors
Wednesday, 03/11/2015 , 03:15PM - 04:45PM

J Clin Periodontol. 2016 Dec;43(12):1171-1179. doi: 10.1111/jcpe.12610. Epub 2016 Oct 17.

Immediate implant placement and provisionalization in the aesthetic zone using a flapless or a flap-involving approach: a randomized controlled trial.

Stoupel J[1], Lee CT[1], Glick J[1], Sanz-Miralles E[1], Chiuzan C[2], Papapanou PN[1].

⊕ Author information

Abstract

AIM: We conducted a randomized controlled trial to compare the effect of flapless (FLS) or flap-involving (F) immediate placement and provisionalization of single-tooth implants in the aesthetic zone.

MATERIALS AND METHODS: Thirty-nine patients were randomized following extraction of a non-restorable tooth to a FLS or F group. All implants were immediately placed and provisionalized. We monitored prospectively changes in the peri-implant mucosal margin, the interproximal bone and buccal horizontal ridge at 3, 6 and 12 months.

RESULTS: At 3 months post-surgery, the mean ± SD [median (interquartile range)] mesiobuccal peri-implant gingival margin recession from the pre-surgical soft tissue position amounted to 0.11 ± 0.32 mm [0 (0, 0.5)] in the FLS treatment arm versus 0.43 ± 37 mm [0.5 (0, 0.5)] in the F treatment arm ($p = 0.02$); corresponding values at the distobuccal surface were 0.11 ± 32 mm [0 (0, 0)] in the FLS arm versus 0.48 ± 0.44 mm [0.5 (0, 1)] in the F arm ($p = 0.01$). No other significant differences in soft or hard tissue remodelling between the treatment arms were observed at 3, 6 or 12 months.

CONCLUSIONS: Flapless and a flap-involving immediate implant placement and provisionalization in the aesthetic zone resulted in comparable remodelling of the peri-implant mucosa, interproximal bone and buccal ridge at 6 and 12 months.

© 2016 John Wiley & Sons A/S. Published by John Wiley & Sons Ltd.

KEYWORDS: aesthetics; alveolar process; dental implants; gingival recession; surgical flap

PMID: 27501953 DOI: 10.1111/jcpe.12610

High-Definition Non-Invasive Brain Modulation of Sensorimotor Dysfunction in Chronic TMD

Objectives: Temporomandibular disorders (TMD) have a relatively high prevalence and in many patients pain and masticatory dysfunction persist despite a range of treatments. New non-invasive brain neuromodulatory methods, such as transcranial direct current stimulation (tDCS), can now safely provide relatively long-lasting pain relief in chronic pain patients. The objective of this study was to define the neuromodulatory effect of five daily 2x2 motor cortex high-definition tDCS (HD-tDCS) sessions on clinical sensorimotor measures in chronic TMD patients.
Methods: Twenty-four female patients with myofascial TMD diagnosis were enrolled in this randomized, placebo-controlled, single-blind, parallel-group study. Baseline values were obtained one-week prior to treatment, and then subjects participated in five daily sessions of either active or sham 2 milliamps HD-tDCS for 20 minutes. A novel 2 x 2 HD-tDCS montage was developed and applied unilaterally over the putative primary motor cortical (M1) region, guided by combined electroencephalography and computational modeling systems. Follow-up sessions were held one-week and four-weeks post treatment. Measurable outcomes included pain-free mouth opening, sectional sensory-discriminative pain measures (e.g., area and intensity) tracked by mobile application developed in-house (Pain*Trek)*, McGill Pain Questionnaire, and positive and negative affect scale (PANAS).
Results: There were statistically significant improvements for clinical sensorimotor measurements in the active HD-tDCS TMD group compared to the placebo group for: responders with pain relief above 50% in the visual analogue scale at four-week follow-up ($p=0.04$); pain-free mouth opening at one-week follow-up ($p<0.01$); and sectional pain area, intensity and their sum measures contralateral to putative M1 stimulation during the treatment week ($p<0.01$). No changes in emotional values were noticed between groups after M1 HD-tDCS sessions as measured with PANAS.
Conclusions: Putative M1 stimulation by HD-tDCS selectively improved meaningful clinical sensorimotor measures during stimulation and up to four weeks post treatment in chronic myofascial TMD pain patients.

- Gupta Vikas (University of Michigan , Ann Arbor Ann Arbor , Michigan , United States)
- Zieba Tina (University of Michigan , Ann Arbor Ann Arbor , Michigan , United States)
- Truong Dennis (City College of New York , New York New York , New York , United States)
- Bikson Marom (City College of New York , New York New York , New York , United States)
- Datta Abhi (Soterix , New York New York , New York , United States)
- Bellile Emily (University of Michigan , Ann Arbor Ann Arbor , Michigan , United States)

Gupta Vikas (University of Michigan , Ann Arbor Ann Arbor , Michigan , United States)
Zieba Tina (University of Michigan , Ann Arbor Ann Arbor , Michigan , United States)
Truong Dennis (City College of New York , New York New York , New York , United States)
Bikson Marom (City College of New York , New York New York , New York , United States)
Datta Abhi (Soterix , New York New York , New York , United States)
Bellile Emily (University of Michigan , Ann Arbor Ann Arbor , Michigan , United States)

Support Funding Agency/Grant Number: American Academy of Orofacial Pain
Financial Interest Disclosure: The City University of New York has patents on brain stimulation with Marom Bikson and Abhishek Datta listed as inventors. Marom Bikson and Abhishek Datta have equity in Soterix Medical Inc.

SESSION INFORMATION
Oral Session
Sleep Disorders and Neuroimaging
Friday, 03/13/2015 , 08:00AM - 09:30AM

Brain Stimul. 2015 Nov-Dec;8(6):1085-92. doi: 10.1016/j.brs.2015.06.008. Epub 2015 Jun 23.

High-Definition and Non-invasive Brain Modulation of Pain and Motor Dysfunction in Chronic TMD.

Donnell A[1], D Nascimento T[2], Lawrence M[2], Gupta V[2], Zieba T[2], Truong DQ[3], Bikson M[3], Datta A[4], Bellile E[5], DaSilva AF[6].

⊕ Author information

Abstract

BACKGROUND: Temporomandibular disorders (TMD) have a high prevalence and in many patients pain and masticatory dysfunction persist despite a range of treatments. Non-invasive brain neuromodulatory methods, namely transcranial direct current stimulation (tDCS), can provide relatively long-lasting pain relief in chronic pain patients.

OBJECTIVE: To define the neuromodulatory effect of five daily 2x2 motor cortex high-definition tDCS (HD-tDCS) sessions on clinical pain and motor measures in chronic TMD patients. It is predicted that M1 HD-tDCS will selectively modulate clinical measures, by showing greater analgesic after-effects compared to placebo, and active treatment will increase pain free jaw movement more than placebo.

METHODS: Twenty-four females with chronic myofascial TMD pain underwent five daily, 20-min sessions of active or sham 2 milliamps (mA) HD-tDCS. Measurable outcomes included pain-free mouth opening, visual analog scale (VAS), sectional sensory-discriminative pain measures tracked by a mobile application, short form of the McGill Pain Questionnaire, and the Positive and Negative Affect Schedule. Follow-up occurred at one-week and four-weeks post-treatment.

RESULTS: There were significant improvements for clinical pain and motor measurements in the active HD-tDCS group compared to the placebo group for: responders with pain relief above 50% in the VAS at four-week follow-up ($P = 0.04$); pain-free mouth opening at one-week follow-up ($P < 0.01$); and sectional pain area, intensity and their sum measures contralateral to putative M1 stimulation during the treatment week ($P < 0.01$). No changes in emotional values were shown between groups.

CONCLUSION: Putative M1 stimulation by HD-tDCS selectively improved meaningful clinical sensory-discriminative pain and motor measures during stimulation, and up to four-weeks post-treatment in chronic myofascial TMD pain patients.

Copyright © 2015 Elsevier Inc. All rights reserved.

KEYWORDS: Clinical trial; Pain; PainTrek; Temporomandibular disorder; Transcranial direct current stimulation

Mandibular Overdentures Retained by One or Two Implants: Kinesiographic Study

Mandibular Overdentures Retained by One or Two Implants: Kinesiographic Study

Objectives: The purpose of this study was to evaluate the effect of mandibular overdentures retained by one and two implants on the kinesiographic parameters of new well-fitting complete dentures wearers.
Methods: Edentulous patients (n=16) received one set of new complete dentures. After adaptation period (30 days), the subjects were randomized and assigned to two groups, as follow: G1- One implant in the anterior region of the mandible; G2- Two implants in the anterior region of the mandible. After a healing period (04 months), o´ring attachments were installed on the implants and the matrix attachments were connected in the mandibular dentures. The kinesiograph instrument K7-I - Scan 8 - was used to record chewing cycle in a vertical and horizontal planes before the surgery (T0) and 06 months (T1) after the insertion of the overdentures. Data were analyzed using the parametric "t-Student" test ($\alpha = .05$).
Results: After 06 months, the chewing cycles in mandibular overdentures retained by one implant (VER: μ=15.37±2.10; HOR: μ=7.37±3.33) and two implants (VER: μ=14.60±1.17; HOR: μ=6.27±1.93) showed a significant increased on mandibular vertical and horizontal movements ($p<.05$) when compared with T0 (one implant: VER: μ=11.44±1.78; HOR: μ=2.75±1.38 – two implant: VER: μ=12.54±1.90; HOR: μ=2.76±2.59). When mandibular overdentures retained by one and two implants were compared between them, after 06 months, no significant difference was found for vertical (p=.380) and horizontal (p=.433) movements.
Conclusions: It can be concluded that mandibular overdentures retained by one and two implants increased vertical and horizontal mandibular movements during chewing when compared with conventional complete dentures. Both treatments showed similar amplitude of the movements during chewing after 06 months of the treatment.

Division: IADR/AADR/CADR General Session
Meeting: 2015 IADR/AADR/CADR General Session (Boston, Massachusetts)
Location: Boston, Massachusetts
Year: 2015
Final Presentation ID: 0979

Authors

- Mendoza-marin Danny (Univ Estadual Paulista , Araraquara Araraquara , São Paulo , Brazil)
- Paleari André (Univ Estadual Paulista , Araraquara Araraquara , São Paulo , Brazil)
- Rodriguez Larissa (Univ Estadual Paulista , Araraquara Araraquara , São Paulo , Brazil)
- Oliveira-júnior Norberto (Univ Estadual Paulista , Araraquara Araraquara , São Paulo , Brazil)
- Policastro Vivian (Univ Estadual Paulista , Araraquara Araraquara , São Paulo , Brazil)
- Arioli Filho João (Univ Estadual Paulista , Araraquara Araraquara , São Paulo , Brazil)
- Pero Ana (Univ Estadual Paulista , Araraquara Araraquara , São Paulo , Brazil)
- Compagnoni Marco (Univ Estadual Paulista , Araraquara Araraquara , São Paulo , Brazil)

Mendoza-marin Danny (Univ Estadual Paulista , Araraquara Araraquara , São Paulo , Brazil)
Paleari André (Univ Estadual Paulista , Araraquara Araraquara , São Paulo , Brazil)
Rodriguez Larissa (Univ Estadual Paulista , Araraquara Araraquara , São Paulo , Brazil)
Oliveira-júnior Norberto (Univ Estadual Paulista , Araraquara Araraquara , São Paulo , Brazil)
Policastro Vivian (Univ Estadual Paulista , Araraquara Araraquara , São Paulo , Brazil)
Arioli Filho João (Univ Estadual Paulista , Araraquara Araraquara , São Paulo , Brazil)
Pero Ana (Univ Estadual Paulista , Araraquara Araraquara , São Paulo , Brazil)
Compagnoni Marco (Univ Estadual Paulista , Araraquara Araraquara , São Paulo , Brazil)

Support Funding Agency/Grant Number: FAPESP Grant Number: 2012/03809-2
Financial Interest Disclosure: NONE

SESSION INFORMATION
Poster Session
Clinical and Biological Research, Implant Prosthodontics
Thursday, 03/12/2015 , 03:30PM - 04:45PM

Mandibular Overdentures Retained by One or Two Implants: Kinesiographic Study

A Randomized Clinical Trial of Oral Health-Related Quality of Life, Peri-Implant and Kinesiogr Overdentures (PMID:29679427)

Abstract Citations

Policastro VB[1], Paleari
Pero AC[1]

Affiliations

1. Department of Dent UNESP, Univ Estadual P
2. Department of Resto Alfenas, Brazil.
3. Department of Perio Gainesville, FL.

Close affiliations ✖

Journal of Prosthodont
Type: Journal Article
DOI: 10.1111/jopr.127

Abstract

The present randomized clinical trial compared the oral health-related quality of life (OHRQoL), peri-implant parameters, mandible movements, and maxillary complete denture movement during chewing between wearers of single- (1-IOD) and wearers of two-implant overdentures (2-IODs) for a period of 12 months.Twenty-one complete denture wearers were randomly allocated into two parallel groups: 1-IOD (n = 11) or 2-IODs (n = 10). The validated Brazilian version of the OHIP-EDENT was used to evaluate the OHRQoL. A kinesiograph recorded maxillary complete denture movement during chewing of hard food testing (polysulphide impression material) and soft food testing (bread). Peri-implant parameters were also recorded: plaque index (PI), bleeding on probing (BOP), and probing depth (PD). The Friedman test was used to compare the OHRQoL data and peri-implant parameters among periods; the Mann-Whitney test was performed to compare the groups (1- and 2-IODs). One-way ANOVA and the Bonferroni test were used to compare mandible movement during chewing among periods, and the t-test for independent samples was used to compare the groups. Maxillary complete denture movement was analyzed using three-way ANOVA followed by the Bonferroni test. All statistical analyses were performed at $\alpha = 0.05$.Both treatments led to better general OHRQoL in comparison to conventional complete dentures ($p < 0.001$). Better OHRQoL was observed among 2-IOD patients at the 12-month follow up ($p = 0.034$). Peri-implant parameters were similar irrespective of the group and follow-up period. Vertical opening was significantly higher among 1-IOD patients at 3 months ($p = 0.038$). Decreased maxillary denture vertical intrusions were observed with complete dentures in comparison with overdentures ($p = 0.006$), regardless of the food test ($p = 0.251$); however, vertical intrusion was significantly higher among 1-IOD patients ($p = 0.043$).This study suggested that 1-IOD can improve patient OHRQoL and may be similar to 2-IODs in preservation of both peri-implant parameters and masticatory movements.

Masticatory Performance of Overdentures Retained by Mini or Conventional Implants

Objectives: This study aimed to compare the masticatory performance of overdentures retained by mini or conventional implants as a treatment modality for adult edentulous patients.

Methods: Fifty four edentulous patients requesting treatment with implants because of difficulties during speech and chewing with the mandibular denture were randomly allocated into three groups according to one of three possible implant treatment planning options for the mandible to support overdentures: Group 1 – received four mini implants; Group 2 - two mini implants; and Group 3 – two conventional implants. A period of three months was respected prior to the installation of o'ring matrices in the mandibular denture base for all groups. After 3 months following the matrices insertion, masticatory performance was evaluated by means of a color-changeable gum chewed during sixty cycles. Changes in color were assessed using a colorimeter, and the calculated color difference (ΔE) was used as a measure of masticatory performance. The results were compared by means of analysis of variance using the software SPSS 22.0 (α=5%).

Results: Mean values (95% confidence interval) of ΔE for Group 1, Group 2 and Group 3 were, respectively, 18.24 (15.31-21.17), 17.65 (14.18-21.12) and 18.89 (16.21-21.57). The three groups showed similar masticatory performance with no significant statistical difference (P = 0.832).

Conclusions: It can be concluded that overdentures retained by two or four mini dental implants are able to provide masticatory function to a level comparable to the conventional protocol. The results of this study also suggest that the mandibular overdenture retained by both two or four mini dental implants can provide edentulous patients with satisfactory masticatory performance.

Division: IADR/AADR/CADR General Session
Meeting: 2015 IADR/AADR/CADR General Session (Boston, Massachusetts)
Location: Boston, Massachusetts
Year: 2015
Final Presentation ID: 0981

Authors

- Cunha Tatiana (University of São Paulo , Ribeirão Preto Ribeirão Preto , São Paulo , Brazil)
- Ribeiro Adriana (University of São Paulo , Ribeirão Preto Ribeirão Preto , São Paulo , Brazil)
- Vecchia Maria Paula (University of São Paulo , Ribeirão Preto Ribeirão Preto , São Paulo , Brazil)
- Sorgini Danilo (University of São Paulo , Ribeirão Preto Ribeirão Preto , São Paulo , Brazil)
- Reis Andréa (University of São Paulo , Ribeirão Preto Ribeirão Preto , São Paulo , Brazil)
- Muglia Valdir (University of São Paulo , Ribeirão Preto Ribeirão Preto , São Paulo , Brazil)
- De Souza Raphael (University of São Paulo , Ribeirão Preto Ribeirão Preto , São Paulo , Brazil)
- Albuquerque-junior Rubens (University of São Paulo , Ribeirão Preto Ribeirão Preto , São Paulo , Brazil)

Cunha Tatiana (University of São Paulo , Ribeirão Preto Ribeirão Preto , São Paulo , Brazil)
Ribeiro Adriana (University of São Paulo , Ribeirão Preto Ribeirão Preto , São Paulo , Brazil)
Vecchia Maria Paula (University of São Paulo , Ribeirão Preto Ribeirão Preto , São Paulo , Brazil)
Sorgini Danilo (University of São Paulo , Ribeirão Preto Ribeirão Preto , São Paulo , Brazil)
Reis Andréa (University of São Paulo , Ribeirão Preto Ribeirão Preto , São Paulo , Brazil)
Muglia Valdir (University of São Paulo , Ribeirão Preto Ribeirão Preto , São Paulo , Brazil)
De Souza Raphael (University of São Paulo , Ribeirão Preto Ribeirão Preto , São Paulo , Brazil)
Albuquerque-junior Rubens (University of São Paulo , Ribeirão Preto Ribeirão Preto , São Paulo , Brazil)

Support Funding Agency/Grant Number: FAPESP, grant 2011/00688-7 ; CNPq, scholarship.
Financial Interest Disclosure: NONE

SESSION INFORMATION
Poster Session
Clinical and Biological Research, Implant Prosthodontics
Thursday, 03/12/2015 , 03:30PM - 04:45PM

Masticatory Performance of Overdentures Retained by Mini or Conventional Implants

Journal of Dental Research

IADR International Association for Dental Research

AADR® American Association for Dental Research

Mini vs. Standard Implants for Mandibular Overdentur

A Randomized Trial

R.F. de Souza, A.B. Ribeiro, M.P. Della Vecchia, more...

First Published August 20, 2015 | Research Article | Check for updates

https://doi.org/10.1177/0022034515601959

Article information

Abstract

A mandibular implant-retained overdenture is considered a first-choice treatment for edentulism. However, some aspects limit the use of standard implants—for example, the width of edentulous ridges, chronic diseases, fear, or costs. This randomized trial compared mandibular overdentures retained by 2 or 4 mini-implants with standard implants, considering oral health–related quality of life (OHRQoL), patient satisfaction, and complications such as lost implant. In sum, 120 edentulous men and women (mean age, 59.5 ± 8.5 y) randomly received 4 mini-implants, 2 mini-implants, or 2 standard implants. Participants provided data regarding OHRQoL and satisfaction until 12 mo. Clinical parameters, including implant survival rate, were also recorded. Both 2 and 4 mini-implants led to better OHRQoL, compared with 2 standard implants. Treatment with 4 mini-implants was more satisfying than 2 standard implants, with 2 mini-implants presenting intermediate results. Implant survival rate was 89%, 82%, and 99% for 4 mini-implants, 2 mini-implants, or 2 standard implants, respectively. Overdentures retained by 4 or 2 mini-implants can achieve OHRQoL and satisfaction at least comparable with that of 2 standard implants. However, the survival rate of mini implants is not as high as that of standard implants (ClinicalTrials.gov NCT01411683).

Keywords

complete denture, edentulous mouth, minimally invasive surgical procedures, patient outcome assessment, patient satisfaction, quality of life

Patient Satisfaction with Electroplated Telescopic Prostheses on Zirconia Primary Crowns

Patient Satisfaction with Electroplated Telescopic Prostheses on Zirconia Primary Crowns

Objectives: Aim of this investigation was to evaluate the effect of electroplated telescopic removable dental prostheses (E-RDPs) with zirconia primary crowns on oral health-related quality of life (OHRQoL) and to compare the data to E-RDPs with metal primary crowns.

Methods: Fifty-six participants in need of 60 RDPs were randomly assigned to receive primary crowns made from either cobalt-chromium alloy or zirconia (NobelProcera). Electroplating was used to form precisely fitting gold copings on the telescopic primary crowns. These copings were intra-orally bonded to the denture framework. OHRQoL was assessed using the 49-item Oral Health Impact Profile (OHIP) and additional patient self-ratings at baseline before treatment, after 6 and 12 months. Statistical analyses were performed with one- and two-sample t-tests and analyses of covariance.

Results: After 6 months, mean OHIP sum score was 20 (SD: 26, 95%CI: 13-27.1) and after 12 months 16.4 (SD: 17.9, 95%CI: 11.6–21.2). This was a significant decrease in comparison to the mean baseline value of 53.4 (SD: 37.4, 95%CI: 41.3-62). More specifically, the mean reduction in OHIP sum score after 12 months was 25 (SD: 31.2, 95%CI: 13.1-36.9) for cobalt-chromium alloy und 44.4 (SD: 32.3, 95%CI: 31.1–57.8) for zirconia. Concerning self-rated patient satisfaction, significant improvements were found after 6 and 12 months compared to baseline. However, the two materials did not show a statistically significant difference, neither for OHIP change nor for changes in patient self-ratings.

Conclusions: OHRQoL is improved using both, cobalt-chromium alloy and zirconia primary crowns for E-RDPs; but there were no statistically significant post-treatment differences between the groups. E-RDPs based on zirconia primary crowns remain appealing because of the beneficial material properties of zirconia and the efficient CAD/CAM-based manufacturing.

Division: IADR/AADR/CADR General Session
Meeting: 2015 IADR/AADR/CADR General Session (Boston, Massachusetts)
Location: Boston, Massachusetts
Year: 2015
Final Presentation ID: 2045

Authors

- Schwindling Franz (University clinics Heidelberg , Heidelberg Heidelberg , Germany)
- Deisenhofer Ulrich (University clinics Heidelberg , Heidelberg Heidelberg , Germany)
- Séché Anne-christiane (University clinics Heidelberg , Heidelberg Heidelberg , Germany)
- Lehmann Franziska (University clinics Heidelberg , Heidelberg Heidelberg , Germany)
- Terebesi Sophia (University clinics Heidelberg , Heidelberg Heidelberg , Germany)
- Rammelsberg Peter (University clinics Heidelberg , Heidelberg Heidelberg , Germany)
- Stober Thomas (University clinics Heidelberg , Heidelberg Heidelberg , Germany)

Schwindling Franz (University clinics Heidelberg , Heidelberg Heidelberg , Germany)
Deisenhofer Ulrich (University clinics Heidelberg , Heidelberg Heidelberg , Germany)
Séché Anne-christiane (University clinics Heidelberg , Heidelberg Heidelberg , Germany)
Lehmann Franziska (University clinics Heidelberg , Heidelberg Heidelberg , Germany)
Terebesi Sophia (University clinics Heidelberg , Heidelberg Heidelberg , Germany)
Rammelsberg Peter (University clinics Heidelberg , Heidelberg Heidelberg , Germany)
Stober Thomas (University clinics Heidelberg , Heidelberg Heidelberg , Germany)

Financial Interest Disclosure: This study was supported by a grant (code 2005-362) provided by Nobel Biocare Services AG, Kloten (ZH), Switzerland

SESSION INFORMATION
Oral Session
Keynote Address; Achievable Outcomes in Prosthodontic Care
Friday, 03/13/2015 , 10:45AM - 12:15PM

Patient Satisfaction with Electroplated Telescopic Prostheses on Zirconia Primary Crowns

Clin Oral Investig. 2017 May;21(4):1157-1163. doi: 10.1007/s00784-016-1869-1. Epub 2016 Jun 9.

Randomized trial investigating zirconia electroplated telescopic retainers: quality of life outcomes.

Schwindling FS[1], Deisenhofer UK[2], Séché AC[3], Lehmann F[4], Rammelsberg P[2], Stober T[2].

Author information

Abstract

OBJECTIVES: The study aims to evaluate the effect of electroplated telescopic removable dental prostheses (E-RDPs) with zirconia primary crowns on oral-health-related quality of life (OHRQoL).

MATERIALS AND METHODS: For E-RDPs, electroplating is used to produce precisely fitting gold copings on telescopic primary crowns. These copings are bonded intra-orally to the denture framework. Fifty-six participants in need of 60 removable restorations were randomly allocated one of two materials for the primary crowns: cobalt-chromium alloy or zirconia. OHRQoL was assessed by use of the 49-item Oral Health Impact Profile (OHIP-49) and by additional patient self-rating at baseline before treatment, and after 6 and 12 months. Statistical analysis was performed by use of one- and two-sample t-tests and analysis of covariance.

RESULTS: Mean OHIP sum score at baseline was 53.4 (SD 37.4, 95 % CI 41.3-62). At follow-ups, it decreased significantly (after 6 months: mean 20, SD 26, 95 % CI 13-27.1; after 12 months: mean 16.4, SD 17.9, 95 % CI 11.6-21.2). The mean reduction in OHIP sum score after 12 months was 25 (SD 31.2, 95 % CI 13.1-36.9) for cobalt-chromium alloy and 44.4 (SD 32.3, 95 % CI 31.1-57.8) for zirconia. However, no statistically significant difference of the two materials on OHIP change or patient self-rating was detected.

CONCLUSIONS: Although OHRQoL was improved by using both cobalt-chromium alloy and zirconia primary crowns for E-RDPs, post-treatment differences between the groups were not statistically significant.

CLINICAL RELEVANCE: Zirconia E-RDPs enhance OHRQoL. However, zirconia primary crowns do not outperform cobalt-chromium alloy crowns regarding patient satisfaction-despite their tooth-like color.

KEYWORDS: Cobalt–chromium; Electroplating; OHRQoL; Patient satisfaction; Removable dental prostheses; Zirconia

PMID: 27277518 DOI: 10.1007/s00784-016-1869-1

Surgical Reconstruction of Peri-implant Osseous Defects: a Multicenter RCT

Surgical Reconstruction of Peri-implant Osseous Defects: a Multicenter RCT

Objectives: There is a paucity of data for the effectiveness of reconstructive procedures in the treatment of peri-implantitis. Porous titanium granules (PTG) have been introduced as an osteoconductive bone substitute. The aim was to compare reconstructive surgery of peri-implant defects with PTG to open flap debridement (OFD).
Methods: In a multi-national, multicenter randomized controlled trial of 12 months duration, 63 patients (36 female, 27 male; mean age: 58.4 ± 12.3 years), each contributing one 3 or 4 wall peri-implant osseous defect (circumferential ≥ 270 degrees) were included using a parallel group design. Subjects had received non-surgical periodontal/peri-implant pre-treatment. All defects were surgically treated by OFD using a titanium brush for debridement and 33 patients received PTG. Implants were not submerged and all patients were prescribed adjunctive systemic antibiotics (amoxicillin and metronidazole) for 7 days. Radiographic outcomes (change of vertical defect depth and of marginal bone level) were assessed by 2 examiners on digitized intraoral radiographs obtained in a standardized way. Statistical analysis was performed by ANCOVA.
Results: Healing was uneventful in both groups with no adverse events. After 12 months the PTG group showed a mean reduction in vertical radiographic defect depth (mesial/distal) of 3.61 mm (SD 1.96) /3.56 mm (SD 2.07) compared to 1.05 mm (SD 1.42)/1.04 (SD 1.34) in the OFD group. Furthermore, in the PTG group a mean gain in marginal radiographic bone level (mesial/distal) of 3.58 mm (SD 2.05)/ 3.45 mm (SD 2.16) was observed compared to 0.96 mm (SD 1.35) /0.84 mm (SD 1.14) in the OFD group. All differences were statistically significant in favor of the test group ($p < 0.05$).
Conclusions: Improvements were obtained following both treatment modalities, however, reconstructive surgery using PTG resulted in significantly enhanced radiographic peri-implant defect fill compared with controls.

Division: IADR/AADR/CADR General Session
Meeting: 2015 IADR/AADR/CADR General Session (Boston, Massachusetts)
Location: Boston, Massachusetts
Year: 2015
Final Presentation ID: 0543

Authors

- Jepsen Karin (University of Bonn , Bonn Bonn , Germany)
- Ortiz-vigon Alberto (Universidad Complutense Madrid , Madrid Madrid , Spain)
- Jansåker Ann-marie (Kristianstad University , Kristianstad Kristianstad , Sweden)
- Renvert Stefan (Kristianstad University , Kristianstad Kristianstad , Sweden)
- Jepsen Søren (University of Bonn , Bonn Bonn , Germany)
- Loos Bruno (Acta , Amsterdam Amsterdam , Netherlands)
- Laine Marja (Acta , Amsterdam Amsterdam , Netherlands)
- Anssari Moin David (Acta , Amsterdam Amsterdam , Netherlands)
- Wismeijer Daniel (Acta , Amsterdam Amsterdam , Netherlands)
- Pilloni Andrea (University of Rome, Sapienza , Rome Rome , Italy)
- Zeza Blerina (University of Rome, Sapienza , Rome Rome , Italy)
- Sanz Mariano (Universidad Complutense Madrid , Madrid Madrid , Spain)

Jepsen Karin (University of Bonn , Bonn Bonn , Germany)
Ortiz-vigon Alberto (Universidad Complutense Madrid , Madrid Madrid , Spain)
Jansåker Ann-marie (Kristianstad University , Kristianstad Kristianstad , Sweden)
Renvert Stefan (Kristianstad University , Kristianstad Kristianstad , Sweden)
Jepsen Søren (University of Bonn , Bonn Bonn , Germany)
Loos Bruno (Acta , Amsterdam Amsterdam , Netherlands)
Laine Marja (Acta , Amsterdam Amsterdam , Netherlands)
Anssari Moin David (Acta , Amsterdam Amsterdam , Netherlands)
Wismeijer Daniel (Acta , Amsterdam Amsterdam , Netherlands)
Pilloni Andrea (University of Rome, Sapienza , Rome Rome , Italy)
Zeza Blerina (University of Rome, Sapienza , Rome Rome , Italy)
Sanz Mariano (Universidad Complutense Madrid , Madrid Madrid , Spain)

Support Funding Agency/Grant Number: partially sponsored by Tigran Technologies, Sweden
Financial Interest Disclosure: The authors declare that they have no conflict of interest. This study was supported by a research grant from Tigran Technologies, Sweden.

SESSION INFORMATION
Oral Session
Keynote Address; Peri-implant Infections-Interventions
Thursday, 03/12/2015 , 10:45AM - 12:15PM

Surgical Reconstruction of Peri-implant Osseous Defects: a Multicenter RCT

Journal of Dental Research

IADR International Association for Dental Research

AAI American Asso… for Dental Res…

Journal Home | Browse Journal | Submit Paper | About | Subscribe

Close

PDF

Reconstruction of Peri-implant Osseous Defects

A Multicenter Randomized Trial

K. Jepsen, S. Jepsen, M.L. Laine, D. Anssari Moin, A. Pilloni, B. Zeza, M. Sanz, A. Ortiz-Vigon, A.M. Roos-Jansåker, S. Renvert

First Published October 8, 2015 | Research Article | Check for updates

https://doi.org/10.1177/0022034515610056

Abstract

There is a paucity of data for the effectiveness of reconstructive procedures in the treatment of peri-implantitis. The objective of this study was to compare reconstruction of peri-implant osseous defects with open flap debridement (OFD) plus porous titanium granules (PTGs) compared with OFD alone. Sixty-three patients (36 female, 27 male; mean age 58.4 y [SD 12.3]), contributing one circumferential peri-implant intraosseous defect, were included in a multinational, multicenter randomized trial using a parallel-group design. After OFD and surface decontamination using titanium brushes and hydrogen peroxide, 33 defects received PTGs. The implants were not submerged. All patients received adjunctive perioperative systemic antibiotics. The primary outcome variable (defect fill) was assessed on digitalized radiographs. Clinical measurements of probing depth (PPD), bleeding on probing (BoP), suppuration, and plaque were taken by blinded examiners. After 12 mo, the test group (OFD plus PTG) showed a mean radiographic defect fill (mesial/distal) of 3.6/3.6 mm compared with 1.1/1.0 in the control group (OFD). Differences were statistically significant in favor of the test group ($P < 0.0001$). The OFD plus PTG group showed a mean reduction in PPD of 2.8 mm compared with 2.6 mm in the OFD group. BoP was reduced from 89.4% to 33.3% and from 85.8% to 40.4% for the test and control groups, respectively. There was no significant difference in complete resolution of peri-implantitis (PPD ≤4 mm and no BoP at six implant sites and no further bone loss), because this finding was accomplished at 30% of implants in the test group and 23% of implants in the control group. Reconstructive surgery using PTGs resulted in significantly enhanced radiographic defect fill compared with OFD. However, limitations in the lack of ability to discern biomaterial from osseous tissue could not be verified to determine new bone formation. Similar improvements according to clinical measures were obtained after both surgical treatment modalities (ClinicalTrials.gov NCT02406001).

Tooth Bleaching Effects on the Adhesive Interface of Composite Restorations

Objectives: Teeth that will be bleached may have proximal composite restorations, which can be preserved since the difference in color of the bleached tooth and the restoration is indistinguishable. However, the question remains whether whitening will affect the adhesion of restorative materials to the tooth. The objective was to evaluate the effects of different bleaching techniques on the tooth-restoration interface of composite restorations.

Methods: Cavities (3x3x2mm) were prepared in 100 bovine incisor fragments, which were etched with a conventional adhesive system and restored with a micro-hybrid composite. Those fragments were randomly divided into five groups (n=20): Control (no bleaching), At-Home Bleaching (10% hydrogen peroxide), In-Office Bleaching (35% hydrogen peroxide), LED Activated Bleaching (35% hydrogen peroxide activated by LED), and Laser Activated Bleaching (35% hydrogen peroxide activated by diode laser, λ=808nm). After bleaching, ten samples per group were thermocycled (500 cycles, 5–55^{0}C), immersed in 50% silver nitrate solution, sectioned, evaluated under a stereomicroscope, and scored for microleakage. The other samples were pH cycled for 14 consecutive days, sectioned, and the enamel adjacent to the adhesive interface was assessed by cross-sectional Knoop hardness. The data were compared using one-way ANOVA (α=0.05).

Results: No differences between the microleakage indexes found for the control group and experimental groups were observed. The enamel of the bleached groups located near the adhesive interface presented the same Knoop hardness numbers as the samples of the control group.

Conclusions: Tooth bleaching does not damage the tooth-restoration interface of composite restorations.

Division: IADR/AADR/CADR General Session
Meeting: 2015 IADR/AADR/CADR General Session (Boston, Massachusetts)
Location: Boston, Massachusetts
Year: 2015
Final Presentation ID: 0843

Lancellotti, Ailla (Piracicaba Dental School, University of Campinas-UNICAMP , Piracicaba Piracicaba , Sao Paulo , Brazil)
Silva, Lorena (Uberaba University , Uberaba Uberaba , Brazil)
Nogueira, Ruchele (Uberaba University , Uberaba Uberaba , Brazil)
Geraldo-martins, Vinicius (Uberaba University , Uberaba Uberaba , Brazil)

Authors

- Lancellotti, Ailla (Piracicaba Dental School, University of Campinas-UNICAMP , Piracicaba Piracicaba , Sao Paulo , Brazil)
- Silva, Lorena (Uberaba University , Uberaba Uberaba , Brazil)
- Nogueira, Ruchele (Uberaba University , Uberaba Uberaba , Brazil)
- Geraldo-martins, Vinicius (Uberaba University , Uberaba Uberaba , Brazil)

Financial Interest Disclosure: NONE

SESSION INFORMATION
Poster Session
Restorative Materials and Technique Effects upon Interfacial Adaptation II
Thursday, 03/12/2015 , 03:30PM - 04:45PM

Tooth Bleaching Effects on the Adhesive Interface of Composite Restorations

Int J Esthet Dent. 2017;12(1):96-106.

Tooth bleaching effects on the adhesive interface of composite restorations.

Silva L, Thedei G Jr, Menezes-Oliveira MA, Nogueira RD, Geraldo-Martins V.

Abstract

The objective of this study was to evaluate the effects of different bleaching techniques on the tooth-restoration interface of composite restorations. Cavities (3 x 3 x 2 mm) were prepared in 100 bovine incisor fragments, which were etched with a conventional adhesive system and restored with a nanocomposite. The fragments were randomly divided into five groups (n = 20): Control (no bleaching), At-home bleaching (HB) (10% hydrogen peroxide [HP]), In-office bleaching (OB) (35% HP), LED-activated bleaching (LB) (35% HP activated by LED), and Laser-activated bleaching (LaB) (35% HP activated by diode laser, λ = 880 nm). After bleaching, 10 samples per group were thermocycled (500 cycles, 5°C to 55°C), immersed in 50% silver nitrate solution, sectioned, evaluated under a stereomicroscope, and scored for microleakage. The other samples were pH cycled for 14 consecutive days, sectioned, and the enamel adjacent to the adhesive interface assessed by cross-sectional Knoop hardness. The data were compared using the one-way ANOVA ($\alpha = 0.05$). No differences between the microleakage indexes found for the control and experimental groups were observed. The enamel of the bleached groups located near the adhesive interface presented the same Knoop hardness numbers as the samples of the control group. Tooth bleaching does not damage the tooth-restoration interface of composite restorations.

PMID: 28117858

Two-year Success, Clinical Performance and Patient Satisfaction of Fiber-reinforced-composite Fixed-partial-dentures

Two-year Success, Clinical Performance and Patient Satisfaction of Fiber-reinforced-composite Fixed-partial-dentures

Objectives: The purpose of the study is to evaluate the success, clinical performance, and patient satisfaction of direct placed fiber reinforced composite (FRC) fixed partial dentures (FPDs) in a two-year follow up period.
Methods: 120 subjects who met the inclusion criteria were enrolled. 167 FRC-FPDs were directly fabricated to restore single missing tooth by six Advanced Education in General Dentistry (AEGD) residents. The FRC FPDs were randomized into groups dependent on missing tooth location, retention type (surface, inlay, and hybrid) and fiber materials (pre-impregnated glass fiber (StickTech) and polyethylene fiber (Ribbond)). The prostheses clinical performances were evaluated at the baseline (2-weeks), 6, 12, and 24-month by two calibrated evaluators for prosthesis adaptation, color match, marginal discoloration, surface roughness, caries, and post-operative sensitivity using modified USPHS criteria. Patients were requested to evaluate the prosthesis appearance, color, chewing ability and overall satisfaction using visual analogue scale (VAS). Kaplan-Meier success curve was plotted using IBM-SPSS.
Results: During 2-year follow up, 94 patients with 137 FRC-FPDs returned which resulted in an attrition rate of 21.67% for study subjects and 17.94% for FRC-FPDs. 17 FRC-FPDs failed due to one-end (4), two-ends (4) debonding or pontic fracture (9). For an accumulative 2-year success rate of 84.32%, there is no statistical difference between groups of different missing tooth location, retention type or fiber materials (P>0.05). The FRC-FPDs demonstrated satisfied prosthesis adaptation, color match, marginal discoloration and surface roughness at 24-month. The patient satisfaction was rated high on the VAS scale (Mean>80) for all criteria at baseline, 6, 12, and 24-month.
Conclusions: In this study, the FRC-FPDs (restoring single tooth) fabricated by AEGD residents achieved 84.32% success rate in a 2-year follow up period. The FRC-FPDs success was not affected by missing tooth location, retention type or fiber materials. Long-term follow up is desirable in future study.

Division: IADR/AADR/CADR General Session
Meeting: 2015 IADR/AADR/CADR General Session (Boston, Massachusetts)
Location: Boston, Massachusetts
Year: 2015
Final Presentation ID: 3701

Xiao Jin (Eastman Institute for Oral Health, University of Rochester , Rochester Rochester , New York , United States)
Dellanzo-savu Alina (Eastman Institute for Oral Health, University of Rochester , Rochester Rochester , New York , United States)
Jabeen Ayesha (Eastman Institute for Oral Health, University of Rochester , Rochester Rochester , New York , United States)
Romero Mario (Georgia Regents University College of Dental Medicine , Augusta Augusta , Georgia , United States)
Huang Jinwei (Eastman Institute for Oral Health, University of Rochester , Rochester Rochester , New York , United States ; Peking University , Beijing Beijing , China)
Yunkeer Michael (Eastman Institute for Oral Health, University of Rochester , Rochester Rochester , New York , United States)
Ren Yan-fang (Eastman Institute for Oral Health, University of Rochester , Rochester Rochester , New York , United States)
Malmstrom Hans (Eastman Institute for Oral Health, University of Rochester , Rochester Rochester , New York , United States)

Authors

- Xiao Jin (Eastman Institute for Oral Health, University of Rochester , Rochester Rochester , New York , United States)
- Dellanzo-savu Alina (Eastman Institute for Oral Health, University of Rochester , Rochester Rochester , New York , United States)
- Jabeen Ayesha (Eastman Institute for Oral Health, University of Rochester , Rochester Rochester , New York , United States)
- Romero Mario (Georgia Regents University College of Dental Medicine , Augusta Augusta , Georgia , United States)
- Huang Jinwei (Eastman Institute for Oral Health, University of Rochester , Rochester Rochester , New York , United States ; Peking University , Beijing Beijing , China)
- Yunkeer Michael (Eastman Institute for Oral Health, University of Rochester , Rochester Rochester , New York , United States)
- Ren Yan-fang (Eastman Institute for Oral Health, University of Rochester , Rochester Rochester , New York , United States)
- Malmstrom Hans (Eastman Institute for Oral Health, University of Rochester , Rochester Rochester , New York , United States)

Financial Interest Disclosure: None

SESSION INFORMATION
Poster Session
Clinical Trials III
Saturday, 03/14/2015 , 03:30PM - 04:45PM

Two-year Success, Clinical Performance and Patient Satisfaction of Fiber-reinforced-composite Fixed-partial-dentures

Journal of Oral Rehabilitation

Original Article | Full Access

Success, clinical performance and patient satisfaction of direct fibre-reinforced composite fixed partial dentures – a two-year clinical study

H. Malmstrom, A. Dellanzo-Savu, J. Xiao, C. Feng, A. Jabeen, M. Romero, J. Huang, Y. Ren, M. A. Yunker

First published: 14 July 2015 | https://doi.org/10.1111/joor.12327 | Cited by: 4

Summary

To evaluate the success, clinical performance and patient satisfaction of directly placed fibre-reinforced composite (FRC) fixed partial dentures (FPDs) in 2 years. One hundred sixty-seven FRC FPDs (120 subjects) were directly fabricated to restore a single missing tooth by six Advanced Education in General Dentistry (AEGD) residents. The FRC FPDs recipients were rando-mised into two groups according to the fibre materials (pre-impregnated glass or polyethylene). Clinical performance was evaluated at baseline (2 weeks), 6, 12 and 24 months by two calibrated evaluators for prosthesis adaptation, colour match, marginal discoloration, surface roughness, caries and post-operative sensitivity using modified United State Public Health Service (USPHS) criteria. Prosthesis appearance, colour, chewing ability and overall satisfaction were evaluated by patients using a visual analogue scale (VAS). Kaplan–Meier estimation was used to estimate the prosthesis success. Ninety-four patients with 137 FRC FPDs returned (21·67% attrition rate for study subjects, 17·94% for FRC FPDs). Seventeen FRC FPDs failed, due to one-end (n = 4) or two-ends (n = 4) debonding or pontic fracture (n = 9). The cumu-lative 2-year success rate was 84·32% and survival rate was 92·7%; there were no statistically significant differences between the groups according to different missing tooth location, retention type or fibre materials (P > 0·05). Patient satisfaction regarding prosthesis appearance, col-our, chewing ability and overall satisfaction was rated high on the VAS (mean >80 mm) for all criteria at all time points. The FRC FPDs (restoring single tooth) fabricated by AEGD residents achieved acceptable success and survival rates in a 2-year follow-up.
